# The Effects on Parapatric Divergence of Linkage between Preference and Trait Loci versus Pleiotropy

**DOI:** 10.3390/genes9040217

**Published:** 2018-04-17

**Authors:** Maria R. Servedio, Reinhard Bürger

**Affiliations:** 1 Department of Biology, University of North Carolina, CB#3280, Coker Hall, Chapel Hill, NC 27599, USA; 2 Department of Mathematics, University of Vienna, Oskar-Morgenstern-Platz 1, 1090 Vienna, Austria; reinhard.buerger@univie.ac.at

**Keywords:** assortative mating, inversion, linkage, mate choice, pleiotropy, phenotype matching, preference, speciation

## Abstract

Attempts to uncover the genetic basis of female mating preferences and male signals involved in reproductive isolation have discovered intriguing cases in which loci contributing to these traits co-localize in their chromosomal positions. Such discoveries raise the question of whether alleles at certain loci contribute pleiotropically to male and female components of premating reproductive isolation, versus whether these loci are merely tightly linked. Here we use population genetic models to assess the degree to which these alternatives affect both short term and equilibrium patterns of trait (signal) and preference divergence. We take advantage of the fact that in the case of secondary contact between populations exchanging migrants, patterns of divergence across the range of preference strengths differ markedly when preferences and traits are controlled by the same locus (the case of phenotype matching) versus when they are on separate chromosomes. We find that tight linkage between preference and trait loci can mimic the pleiotropic pattern for many generations (roughly the reciprocal of the recombination rate), but that any recombination ultimately results in equilibrium patterns of divergence far more similar to those found when preferences and traits are on separate chromosomes. In general, our finding that pleiotropy results in quite different long-term patterns from tight linkage highlights the importance of distinguishing between these possibilities in empirical systems.

## 1. Introduction

Premating isolation is thought to be an unusually powerful mechanism of reproductive isolation [1], as its early position in the life cycle preempts the action of low hybrid fitness in restricting or preventing gene flow [2,3]. A common source of premating isolation in animals is mate choice, which can occur by different mechanisms depending on the genes that underlie its components. In some cases, alleles at loci for a mating preference in one sex, usually the female, determine the probability of choosing a mate, based on a mating trait or cue controlled by different loci in the opposite sex (a “preference/trait” mechanism). If incipient species are characterized by divergent preferences and traits, mate choice by this mechanism would lead to assortative mating and hence premating isolation. In other cases, called “matching” mechanisms, a female instead sets her preference to match the trait phenotype that she herself expresses or the trait allele that she carries. In this case, a mechanism that would lead to such matching, such as self-reference (for other matching mechanisms see [4]), may either already be established, or may evolve to affect how choosy females are, but the mean or direction of a female’s preference is set by the alleles that she carries at her own trait loci. Thus if a matching mechanism were present, divergence of a trait alone would be enough to cause assortative mating. Preference/trait and matching mechanisms differ in several important theoretical properties that affect their action during the speciation process [4].

Mate choice by females will almost universally lead to sexual selection, or the differential mating success of males. However, both the way in which this sexual selection is generated and its effect on population divergence can differ between preference/trait and matching mechanisms. This in turn can have important consequences on the evolution and maintenance of trait divergence, and hence premating isolation (reviewed in [4,5,6]).

In the case of preference/trait mechanisms, the frequency of different preference alleles in a population will determine the direction, and along with other loci controlling “choosiness”, the strength of sexual selection on the trait. However, unless preferences are themselves under divergent selection, preference differentiation within or across populations is difficult to maintain—theoretical studies show that preferences will tend to homogenize across differentiated areas when they are selectively neutral [7,8,9]. In turn, these homogenized preferences can cause sexual selection to tend to homogenize traits (this is true of preferences for a specific trait value, i.e., unimodal or “absolute” preferences, but not necessarily for all other types of preference functions, see [10]). Interestingly, stronger preferences will in some circumstances exacerbate this reduction of trait divergence [9,10,11].

In contrast, such homogenization of preferences does not occur under matching mechanisms because the preference is simply based upon the trait itself. Under matching, the frequency of the trait, not a separate preference, in females thus determines the direction (and again along with alleles that control choosiness, the strength) of sexual selection, on this same trait in males. If a trait is more common in a population, more females will possess it, and it will thus be favored by sexual selection. The sexual selection caused by phenotype matching is therefore positive frequency-dependent, based on the frequency of the trait itself. This has contrasting effects on speciation, depending on the geographic scenario and existing trait divergence (reviewed in [5,6]). During sympatric speciation, for example, positive frequency-dependence can cause sexual selection to be stabilizing if trait distributions are initially unimodal (e.g., [12,13,14]). In contrast, during the late stages of sympatric speciation or migration between already partially isolated populations, when traits are already largely differentiated, positive frequency-dependent sexual selection can accentuate this divergence [13,15]. Furthermore, extremely strong preferences guarantee that females with rare traits will wait to mate with a rare matching partner, thereby increasing the mating success of rare males and paradoxically reducing frequency-dependent sexual selection, regardless of the speciation stage or geographic scenario. Thus, intermediate preference strengths lead to the most trait divergence between spatially separated populations under this mechanism [11,15,16].

Identifying whether a pair of incipient species has premating isolation controlled by a preference/trait versus a matching mechanism is not necessarily easy, either behaviorally or genetically (reviewed in [4]). Because during matching a female’s preference is determined by the trait that she carries, the alleles at each trait locus can be thought of as having dual functions, both determining the trait phenotype and determining the preference phenotype; they would thus be technically pleiotropic. In empirical systems, genetic mapping can potentially identify whether preferences are controlled by separate loci from traits, but when preference and trait quantitative trait loci (QTL) co-localize, it is difficult to distinguish between pleiotropy and tightly linked preference and trait loci using mapping techniques (though these possibilities could potentially be differentiated using gene editing with specific alleles). Co-localization of preference and trait has been found in species pairs of interest in speciation research. Evidence from *Laupala*, for example, finds that both pulse rate of song and preference for pulse rate map in part to the same QTL [17,18]; the same situation exists for fore-wing color and color preference in two species of *Heliconius* [19,20,21]. The question then arises, that if pleiotropy between preference and trait alleles is a form of matching, and is thus expected to have the effects on trait divergence which models of matching predict, would the same be true of tight linkage between preferences and traits? Or would these behave entirely differently, following the predictions of a preference/trait model?

We address these questions using a model that assesses preference and trait frequencies in spatially separated populations with gene flow, allowing both tight linkage and pleiotropy. We focus in particular on the case of secondary contact, assessing the differentiation in trait and preference frequencies between populations, or “divergence”, that can be maintained between incipient species. This case, when combined with the assumption of spatial separation, sets up a particularly interesting contrast; as discussed above, in this situation, sexual selection tends to erode trait divergence under a preference/trait mechanism [9,11] whereas it instead promotes trait divergence, especially at intermediate preference strengths, under a matching mechanism [11,15]. The effects of the two mechanisms can thus be easily distinguished. We examine both the equilibria for trait and preference divergence as well as the temporal dynamics, which are of interest because empirical systems in which speciation or reinforcement is studied are often newly-emerging or recently in contact. We find that the distinction between pleiotropy and tight linkage is critical. When preferences and traits are tightly linked, they will eventually reach the same, or very similar, equilibria as would be present if they were instead on separate chromosomes, or in some cases divergence will be lost. However, during shorter time intervals, tight linkage may indeed mimic matching, which may give the misleading impression that sexual selection may ultimately promote divergence in the system.

## 2. Materials and Methods

We consider a population genetic model of secondary contact with two diallelic loci, one for a preference (P) and one for a trait (T) in a polygynous mating system, following [9,11]. We generally assume that divergence in preferences and traits has occurred in allopatry, such that a female preference P_1_ for a male trait T_1_ is initially at a high frequency in population 1, while preference P_2_ for trait T_2_ is initially at a high frequency in population 2, though we examine broader starting conditions as well. We consider the case in which the traits are both locally adapted and serve as mating cues. These “magic traits” (in the terminology of [22]) are commonly considered a “best case” for promoting speciation. We assume that upon secondary contact the two populations retain geographic separation but exchange migrants.

Specifically, we write the frequency of the genotypes P_1_T_1_, P_1_T_2_, P_2_T_1_, and P_2_T_2_ in deme *k* as *x*_1,*k*_ through *x*_4,*k*_, the allele frequency of allele P_*n*_ and T_*n*_ in population *k* as *p*_*n*,*k*_ and *t*_*n*,*k*_ respectively, and the linkage disequilibrium in population *k* as *D_k_*. The life cycle in our model begins with migration, which occurs between the populations at arbitrary rates *m_k_* (the proportion of population *k* that consists of migrants right after migration has occurred), such that:

(1) $$x_{i,k}^{mig}=(1-m_{k})x_{i,k}+m_{k}x_{i,l}.$$

Here the index *k* in the subscript represents population 1 when *l* represents population 2 and vice versa. We assume that *m*_1_ is always smaller than or equal to *m*_2_, such that when *m*_1_ = 0, population 1 behaves as if it is a continent in a continent-island model, and when *m*_1_ = *m*_2_, the populations are undergoing symmetric migration.

After migration, selection occurs, so that the frequencies of each genotype in males (represented by the third subscript *m*) are:

(2) $$x_{i,k,m}^{vs}=\frac{(1+d_{i}s_{k})x_{i,k}^{mig}}{1+s_{k}t_{k,k}^{mig}},$$

where *d_i_* = 1 if the allele T_*k*_ is present in genotype *i*, and *d_i_* = 0 otherwise. This has the effect of giving the selective advantage *s_k_* to the “local” trait allele. Here $t_{k,k}^{mig}$ is the frequency of allele T_*k*_ in population *k* after migration. We primarily consider the situation where *s*_1_ = *s*_2_ = *s*. We examine both a version of the model in which both sexes express the trait (and thus undergo viability selection), such that females, represented by subscript *f*, have the same genotype frequencies as do males ($x_{i,k,f}^{vs}=x_{i,k,m}^{vs}$), and also a version in which males alone express the trait ($x_{i,k,f}^{vs}=x_{i,k}^{mig}$).

Mating and sexual selection follow viability selection. P_1_ females always prefer T_1_ males, and P_2_ females prefer T_2_ males, regardless of location. These preferences occur such that P_*n*_ females are 1 + *α_n_* times more likely to mate with a T_*n*_ male than a mismatched male, if she were to encounter one of each. The parameter *α* can thus be thought of as a measure of the strength of choosiness. The frequency of matings between a female of genotype *j* and a male of genotype *i* is therefore, in both populations:

(3) $$M_{ij,k}=\frac{x_{i,k,m}^{vs}x_{j,k,f}^{vs}(1+b_{ij}\alpha_{1})(1+c_{ij}\alpha_{2})}{\sum_{i}x_{i,k,m}^{vs}(1+b_{ij}\alpha_{1})(1+c_{ij}\alpha_{2})}$$

where *b_ij_* = 1 when *i* is odd, and *j* = 1 or 2 (P_1_ females and T_1_ males), and *b_ij_* = 0 otherwise, *c_ij_* = 1 when *i* is even and *j* = 3 or 4 (P_2_ females and T_2_ males), and *c_ij_* = 0 otherwise, and the denominator is a normalization that assures that females have equal mating success regardless of their genotype (strict polygyny, e.g., [23]). As with viability selection, we primarily consider the situation where the mating strengths of P_1_ and P_2_ females are the same, such that *α*_1_ = *α*_2_ = *α*.

Mating is followed by recombination, which occurs at rate *r* between the loci, following the standard equations for haploid genotypes. The case in which *r* = 0 and the preference and trait loci start out in full linkage disequilibrium (*x*_2,*k*_ = *x*_3,*k*_ = 0) corresponds to pleiotropy between the preference and trait, or phenotype matching, exactly matching [15]. Because we are specifically addressing a case where the alternate alleles T_1_ and T_2_ at the trait locus are both pleiotropic in their effects on preferences and traits, we sometimes use the shorthand language that the “locus” is pleiotropic. Mathematica files performing numerical iterations of the exact recursion equations (which constitute our simulations), and other analyses described below, can be found in Supplementary Materials Files S1 and S2.

## 3. Results

### 3.1. Trait Expressed in Both Sexes

We first assume that both sexes fully express the trait that is under divergent ecological selection and used, in males, as a mating cue. An example of such a trait might be body size. We begin by concentrating on the case of secondary contact after a period of almost complete divergence in allopatry, to ask how preference strength and physical linkage will determine the trait and preference divergence that can be maintained after the commencement of gene flow. For this initial analysis, we assume that the preference and trait are in complete linkage disequilibrium at the start of secondary contact; this allows us to equate the case of no recombination (*r* = 0) between the preference and trait loci with phenotype matching.

**Figure 1:**
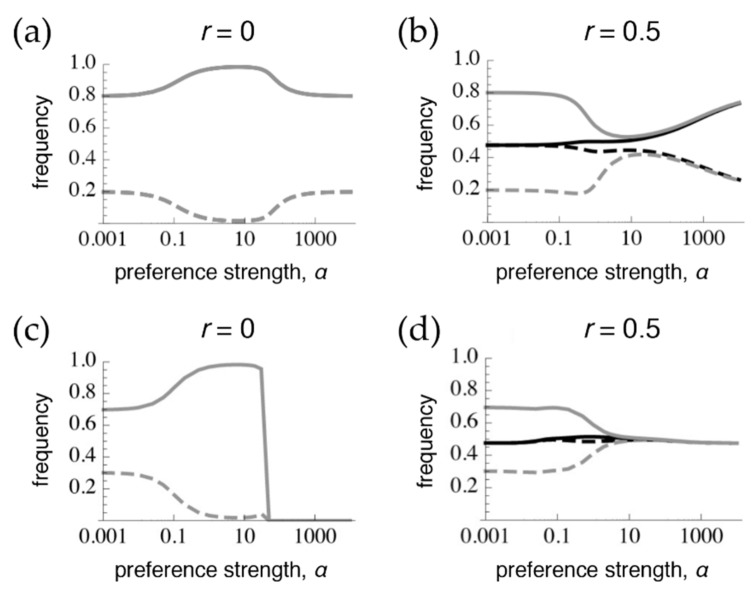
Preference and trait frequencies 1 million generations after secondary contact via the onset of migration. Starting conditions are population 1: *p*_1_ = *t*_1_ = 1.0, population 2: *p*_2_ = *t*_2_ = 0.95 with full linkage disequilibrium. Grey: frequency of the trait T_2_, black: frequency of the preference P_2_, solid: frequencies in population 2, dashed: frequencies in population 1. The difference between the solid and dashed lines for the trait (preference) can be thought of as the amount of trait (preference) divergence. Lines are extrapolations between discrete data points obtained by numerical simulations. The symmetrical migration rate *m* = 0.01 and the strength of selection for local adaptation in both populations *s* = 0.038. The trait is expressed in both sexes in the top row in (**a**,**b**), and is expressed only in males in (**c**,**d**). Note that, as discussed in the main text, slightly different starting conditions will lead to slightly different curves at 1 million generations (in (**a**,**b**) because there is a line of equilibrium present, and in (**c**,**d**) because of extremely slow convergence to an equilibrium point determined by *α*). Sufficiently different starting conditions can also lead to the loss of polymorphism at the T or P loci altogether in any of the panels.

In order to assess whether tight linkage between the preference and trait can mimic pleiotropy, we first obtain, for comparison under these parameter values and starting conditions, the divergence that results after 1 million generations (at or close to equilibrium) at the extremes of pleiotropy (Figure 1a, following [11,15]) versus the case where preferences and traits are on different chromosomes (Figure 1b, following [9]). The case of pleiotropy, or phenotype matching, results in the trait being at migration-selection balance both when preferences are very weak (low *α*) and when preferences are very strong (high *α*), such that all rare males will be chosen by a matching, rare female (Figure 1a). However, at intermediate preference strengths (moderate *α*), sexual selection, which is positive frequency-dependent, drives further divergence between populations than would exist due to viability selection alone. In contrast, when preferences and traits are on different chromosomes (Figure 1b), with weak preferences the preference frequencies are homogenized between populations and the trait is at migration-selection balance. With moderate preferences, the preference alleles are still at only slightly divergent frequencies and so exert sexual selection on the traits that draws their frequencies closer together. With strong preferences, preference frequencies are able to build up more divergence, leading to more divergence in trait frequencies as well [9,11]. Given that preference divergence is never greater than trait divergence, sexual selection generally tends to homogenize trait frequencies across the populations (at least within *α* < 10,000, which would arguably already correspond to separate species).

We next assess the effects of reduced recombination between preferences and traits (*r* = 0.001, 0.01, 0.1). With these starting conditions, the results at equilibrium are very similar, to the point of being visually indistinguishable, to those shown in Figure 1b. In other words, recombination rates as low as *r* = 0.001, which would be infeasible to distinguish from pleiotropy using mapping techniques, lead to equilibrium preference and trait frequencies that are far more similar to those of preference and trait loci on separate chromosomes, than they are to the pattern of matching occurring under pleiotropy (*r* = 0, Figure 1a).

**Figure 2:**
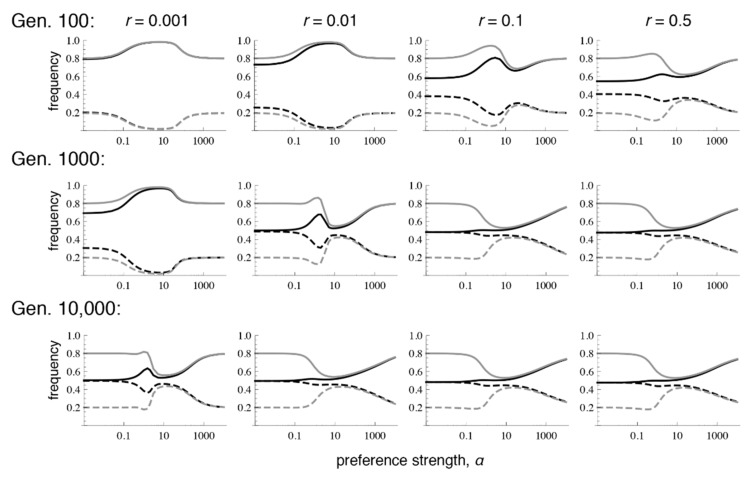
Preference and trait divergence at 100, 1000, and 10,000 generations (Gen) after secondary contact when the trait is expressed in both sexes. The parameter values and legend follow Figure 1. Note that with *r* = 0.001, equilibrium has not yet been reached at 10,000 generations (at equilibrium, this case will resemble the rest of the row).

Patterns of differentiation that depend conspicuously on the recombination rate emerge, however, at lower numbers of generations after contact. Each row of Figure 2 shows the amount of preference and trait divergence that would be observed for a given preference strength if the populations were assessed at a certain number of generations after the onset of migration (assuming the same allele frequencies at secondary contact as in Figure 1). As can be seen in the figure, when *r* = 0.5, the equilibrium pattern is approached relatively quickly. However, with lower recombination rates, the pattern of divergence can look very similar to the pattern expected from phenotype matching for many generations. This can inflate the preference and trait divergence far from that which will be reached at equilibrium, especially with moderate preference strengths, which may be the case most commonly of interest for empirical studies of speciation (i.e., there would be some premating isolation, but it would not be complete). More specifically, at these intermediate preference strengths, because preference divergence can remain inflated for a long time with tight linkage, sexual selection can temporarily allow trait divergence to remain above migration-selection balance. This has the potential to lead to the mistaken impression that sexual selection is “promoting” speciation, whereas when equilibrium is eventually reached, sexual selection will actually have inhibited trait divergence (e.g., the dip in trait frequencies that occurs with intermediate preferences in the bottom row).

Next, we obtain a more thorough picture of the equilibrium conditions by expanding our analysis to consider a broader range of starting conditions, concentrating on the case where population 1 is fixed for T_1_ and P_1_ at the start of contact, while population 2 is polymorphic, and there is a moderately high strength of mating preference. We have shown elsewhere [9] that with separate preference and trait loci (and thus *r* > 0), symmetric starting conditions between the two populations (i.e., *p*_2,1_ = 1 − *p*_2,2_, *t*_2,1_ = 1 − *t*_2,2_, and *D*_1_ = *D*_2_) lead to the existence of a stable polymorphic equilibrium that is independent of the recombination rate *r* and the starting conditions. With non-symmetric starting conditions, this is not the case.

**Figure 3:**
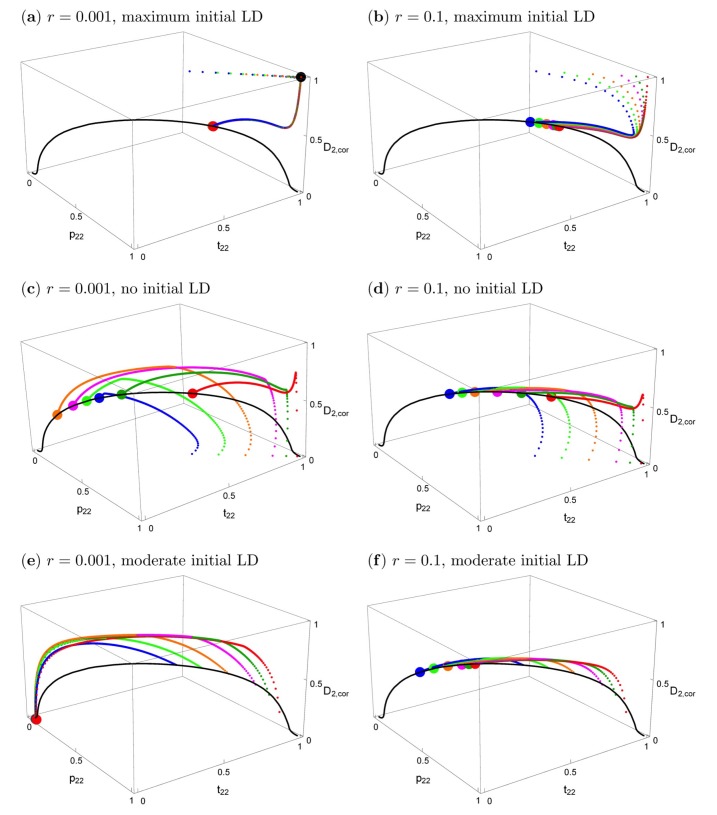
The effects of initial conditions and recombination rates on evolutionary trajectories when the trait is expressed in both sexes and migration is symmetric. Left column: *r* = 0.001; right column: *r* = 0.1. The other parameters are *s*_1_ = *s*_2_ = 0.2, *α*_1_ = *α*_2_ = 10, *m*_1_ = *m*_2_ = 0.01. The black curve is the curve of all stable equilibria. Small dots in different colors show evolutionary trajectories in population 2, starting from different initial values (see below). The bigger colored dots show the equilibrium that is reached. The big black symbol in panel (**a**) indicates the stable polymorphic equilibrium under phenotype matching (*r* = 0). The vertical axis shows the correlation coefficient $D_{2,cor}=D_{2}/\sqrt{p_{2,2}(1-p_{2,2})t_{2,2}(1-t_{2,2})}$. In all panels, population 1 is initially fixed for P_1_ and T_1_, and the trait allele T_2_ has the following initially frequencies in population 2: *t*_2,2_ = 0.99 (red), 0.95 (dark green), 0.90 (magenta), 0.80 (orange), 0.70 (light green), 0.60 (blue). In the top and middle panels, P_2_ has the same initial frequency as T_2_; there is maximum initial linkage disequilibrium (D_2,cor_ = 1) in panels (**a**,**b**), and no initial linkage disequilibrium (D_2,cor_ = 0) in (**c**,**d**). In (**e**,**f**), the initial values of the preference allele and the linkage disequilibrium are taken from the curve of stable equilibria that exists in allopatry (see main text). They are (*p*_2,2_, *t*_2,2_, D_2_) = (0.8695, 0.99, 0.0048) (red), (0.8279, 0.95, 0.0241) (dark green), (0.7766, 0.9, 0.0471) (magenta), (0.6768, 0.8, 0.0471) (orange), (0.5814, 0.7, 0.1123) (light green), (0.4909, 0.6, 0.1261) (blue). Abbreviations: LD, linkage disequilibrium.

Figure 3 shows the effects of the starting conditions and recombination rate on the evolutionary trajectories. In all panels, the black solid curve represents the stable equilibria. The particular equilibrium point that is reached depends on *r* and on the starting conditions, but the curve is the same for every *r* > 0. Small deviations from symmetric initial conditions lead to small deviations from the symmetric equilibrium (Supplementary Materials File S3, Section 3.2), which essentially coincides with the big red dot in Figure 3a,b, the deviation being too small to be visible.

In the top row of Figure 3, the starting conditions are consistent with what would be expected under phenotype matching, i.e., *p*_2,*k*_ = *t*_2,*k*_, *D_k_* = *t*_2,*k*_(1 − *t*_2,*k*_), *k* = 1,2. Therefore, initially, only the two extreme gametes are present (maximum linkage disequilibrium) and the loci are completely correlated. Figure 3a shows that with tight linkage and after secondary contact, the evolutionary trajectories first approach the phenotype-matching equilibrium (the big black dot) for about 20–100 generations, i.e., divergence between the two population increases. However, in contrast to phenotype matching, or pleiotropy (*r* = 0), eventually very slow convergence to a very different equilibrium (the big red dot) occurs (on the order of 10,000–50,000 generations for the given parameters). This equilibrium depends very slightly on initial conditions (hardly visible in this figure because the deviations are so small). A similar evolutionary pattern occurs even for loosely linked loci (Figure 3b). First, there is again some tendency to increase divergence, but after a few generations, evolution takes a different path and convergence to equilibria on the black curve occurs. It is clearly visible that the final evolutionary outcome with very tightly linked loci (*r* = 0.001, red dot in Figure 3a) is much closer to that for separate preference and trait loci (*r* = 0.1, colored dots in Figure 3b) than to phenotype matching (black dot in Figure 3a). A detailed time course of a typical evolutionary trajectory with tightly linked loci is shown in Figure S1a.

The two panels in the second row of Figure 3 are parallel to those in the top row. All parameters and the initial allele frequencies are the same, but here, linkage disequilibrium is initially absent in population 2 at the time of secondary contact, instead of maximal. All evolutionary trajectories converge to the curve of equilibria. There is not an initial increase of trait divergence in this case (for a detailed time course, see Figure S1b). Again, the final evolutionary outcome for very tightly linked versus loosely linked loci is quite similar. Interestingly, the latter case leads to somewhat higher equilibrium frequencies of *p*_2,2_ and *t*_2,2_, and also to slightly higher divergence between the two subpopulations (Supplementary Materials File S3, Section 3.1.2). Although the allele frequencies in the two populations differ from one another and are thus technically “divergent”, it can be seen in Figure 3c,d that the frequencies of T_2_ and P_2_ in population 2 are often below 0.5, and in population 1 they are even lower. Therefore, the same alleles (P_1_ and T_1_) are characteristic of both populations at equilibrium under such starting conditions.

Finally, we considered populations that were at equilibrium when isolated in allopatry. Note that the assumptions of our model lead to a line of polymorphic equilibria for preference and trait frequencies within each allopatric population, as was found in [23]). For a given equilibrium frequency of an ecologically favored trait T_2_ that is prevalent in a population (${\hat{t}}_{2}>0.5$), the equilibrium frequency of the preference allele P_2_ is lower than that of the trait (${\hat{p}}_{2}<{\hat{t}}_{2}$) and is independent of the recombination rate *r* (Supplementary Materials File S3, Section 2.4; Figure S2; see similar model in [23]). In other words, there is a curve of polymorphic equilibria in such a population where, counterintuitively, the equilibrium preference frequency ${\hat{p}}_{2}$ will sometimes be so low that it is below 0.5; this is especially the case when preferences are weak (low *α*, Figure S2). When two populations that are highly divergent in their trait frequencies but are at equilibrium in allopatry contact one another, we observe equilibrium patterns similar to Figure 1b, but without the misleading initial approach to the phenotype matching pattern that is apparent in Figure 2 (see Figure S2).

The two panels in the bottom row of Figure 3 focus on the case where population 1 is fixed for its characteristic trait and preference, and population 2 is initially at an equilibrium on this curve of allopatric equilibrium values. Otherwise, the two panels are parallel to those in the top and middle row of Figure 3. The trait and preference alleles eventually reach low frequencies or are lost; an outcome that is determined by the recombination rate (compare Figure 3e,f). Trait and preference variation is likely to be lost altogether when *r* is very low. This shows that the initially more frequent preference allele (across the total of both populations), here P_1_, may drag “its” trait allele, here T_1_, to fixation.

When populations at equilibrium in allopatry are instead fully symmetrical in their frequencies, we see convergence to the same point regardless of the amount of initial trait divergence, provided that *r* > 0 (Figure S3). Comparison of Figure 3 with Figure S3 shows that symmetric initial conditions (as assumed in [9]) are indeed most conducive to maintaining divergence and local adaptation, i.e., asymmetric initial allele frequencies of the two populations at secondary contact usually lead to reduced divergence and often even to loss of local adaptation in one population, such that maladapted alleles are more common than adaptive ones, or even reach fixation.

With migration rates that are an order of magnitude smaller than above, the evolutionary dynamics are much slower. Both the period of initial approach to the phenotype matching pattern, or with moderate or no initial linkage disequilibrium the period of near constancy of allele frequencies, and the period of final convergence to the true equilibrium, may be elongated by up to an order of magnitude (see Figure S1c,d and Figure S4 which is otherwise parallel to Figure 3).

### 3.2. Trait Expressed Only in Males

Next, we turn to the case in which the mating trait is expressed only in males. Such traits, which include sexually dimorphic ornaments, courtship song, or pheromones, are more commonly the focus of sexual selection research than are traits expressed in both sexes, and thus may often be the subjects of empirical studies of the effects of sexual selection on speciation. Importantly, even when a trait is only expressed in males, preference/trait pleiotropy is still possible; in *Laupala*, for example, there is potential evidence of pleiotropy even though only males produce a song [17,18]. Such cases do not fit a literal reading of the term “phenotype matching” because females do not express the phenotype, but they do fit the technical definition of phenotype matching as a category of models because the preference direction and the trait are determined by a single locus.

When the trait is expressed only in males, trait divergence is lost at high preference strengths both during phenotype matching (T_2_ is lost across both populations in Figure 1c), and with the preference and trait on separate chromosomes (the traits have homogenized across populations in Figure 1d). The loss of one of the alleles with very strong preferences during phenotype matching (Figure 1c) occurs for the following reason: When selection on the trait only occurs in males, females have less divergent trait frequencies than males at the start of the sexual selection stage of the life cycle (immediately after divergent viability selection). Therefore, females that have very strong preferences for matching male phenotypes will generate sexual selection on males that will tend to initially cause the male traits to converge between the populations. This sexual selection can also lead to a (very slow) loss of polymorphism when frequencies are not sufficiently symmetrical between populations. Note that these effects contrast with those that occur when viability selection acts on both sexes, and when all males, regardless of their identity, have a matching female that strongly prefers them.

Given starting conditions that resemble phenotype matching, separate preference and trait loci with free recombination will homogenize across populations much more so when traits are expressed only in males than when females also express the trait (compare Figure 1d with Figure 1b). This occurs because when the trait is expressed in both sexes, females themselves undergo ecological differentiation, increasing trait divergence and subsequently preference divergence (through linkage disequilibrium), right before sexual selection acts in the life cycle (see [9]). Preferences are thus more homogenized at the time of sexual selection when females do not express the trait, and consequently sexual selection has a more inhibitory effect on trait divergence in this case (note that this is not true with all starting conditions; see below). Although sexual selection does not promote trait differentiation with strong preferences in either the cases of matching or separate chromosomes, this inhibition occurs by different mechanisms (loss of one trait allele versus homogenization of the traits, respectively), so the patterns are still easily distinguishable from each other.

**Figure 4:**
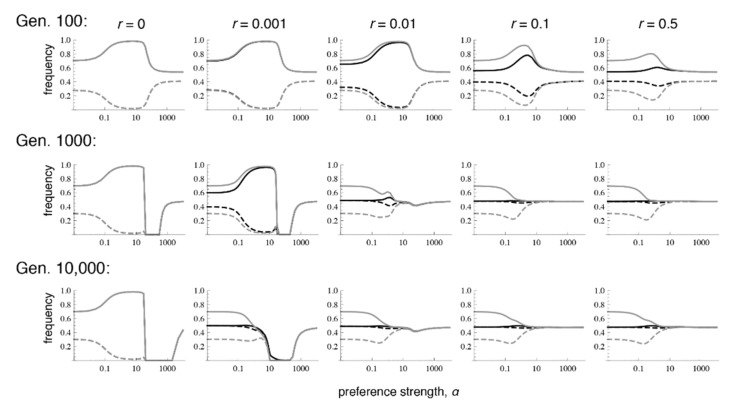
Preference and trait divergence at 100, 1000, and 10,000 generations after secondary contact when the trait is expressed in only in males. The parameter values and legend follow Figure 1. Convergence to equilibrium is much slower when selection on the trait occurs only in males, versus when it occurs in both sexes.

**Figure 5:**
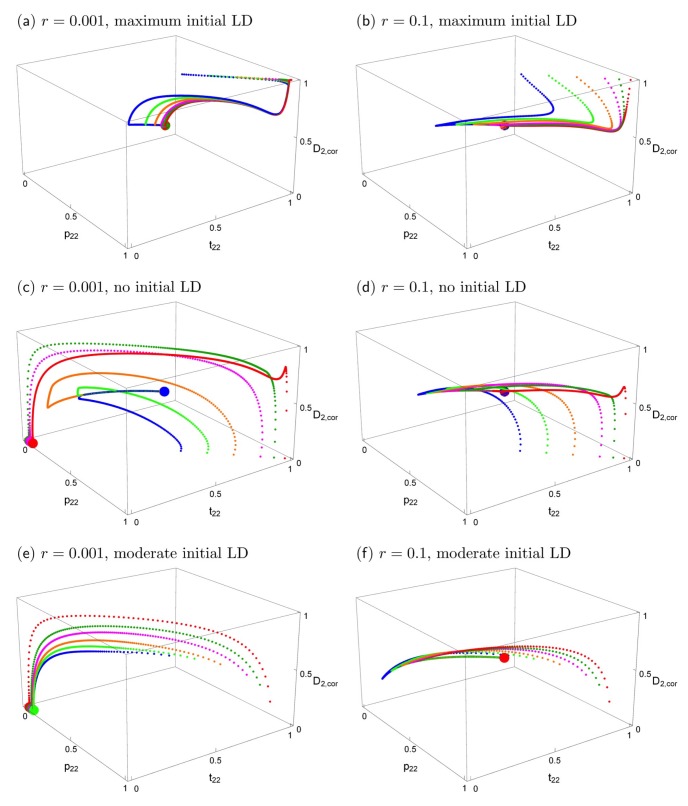
The effects of initial conditions and recombination rates on evolutionary trajectories when the trait is expressed only in males. This figure is analogous to Figure 3 with the sole difference that here the trait is expressed only in males. The fully polymorphic equilibrium is the same in all panels. It is symmetric and given by *p*_2,2_ = 1 − *p*_2,1_ = 0.526, *t*_2,2_ = 1 − *t*_2,1_ = 0.549, *D*_2_ = *D*_1_ = 0.1316. Convergence to the polymorphic equilibrium is typically very slow. If *r* = 0.001 (left column), convergence occurs after about 500,000–1 million generations; if *r* = 0.1 (right column), it occurs after about 10,000–50,000 generations. Loss of the trait and preference alleles occurs on a much shorter time scale, on the order of 100–2000 generations. (**a**,**b**) maximum initial linkage disequilibrium (D_2,cor_ = 1); (**c**,**d**) and no initial linkage disequilibrium (D_2,cor_ = 0); (**e**,**f**), the initial values of the preference allele and the linkage disequilibrium are taken from the curve of stable equilibria that exists in allopatry (see main text).

Similar to the case with selection in both sexes, when there is selection only in males, even very low recombination causes the equilibrium values to differ sharply from those that occur with phenotype matching. More specifically, with selection only in males, the polymorphic equilibrium is uniquely determined when *r* > 0, and it is the same for every *r* > 0 (Supplementary Materials File S4, Section 2.2; Figure 4 and Figure 5; see also [9]). However, the equilibrium that is reached can differ with the recombination rate and the initial frequencies, in that sometimes loss of trait variation occurs. In particular, we see that with very tight linkage trait variation is sometimes lost with intermediate to high preference strengths (bottom row of Figure 4, where *r* = 0.001; Figure 5c,e; the pattern in Figure 4 persists at 1 million generations post-contact). The pattern of trait loss for very tightly linked loci mimics that which is expected from phenotype matching in this range, and apparently occurs because the populations are following the phenotype matching pattern for a long enough period of time to enter this basin of attraction. Looking at patterns of divergence over time, it can again be seen that with low recombination rates, populations can arrive at a pattern of inflated divergence and remain there for some time, especially at intermediate preference strengths (Figure 4).

We again examined a broader range of starting conditions by focusing in more detail on the case paralleling Figure 3, of fixation in population 1 with a polymorphism in population 2 (again with *α* = 10). Comparison of Figure 5 with Figure 3 shows that the short-term behavior of evolutionary trajectories is quite similar, but the long-term behavior differs strongly in most cases. If the trait is expressed only in males, the unique stable polymorphic equilibrium typically exhibits weak differentiation and weak local adaptation, i.e., the frequencies of the locally advantageous trait allele and its preference allele are only slightly above 0.5 (see also bottom row of Figure 4 if *α* > 1). In addition, convergence to the polymorphic equilibrium follows complex trajectories and is typically extremely slow. A typical trajectory is shown in Figure S1e, in which the different phases are made clearly visible. However, depending on initial conditions and the recombination rate, loss of the trait and preference alleles can also occur. This occurs usually on a much shorter time scale (see also Figure S1f).

If populations were strongly divergent and in linkage equilibrium before contact, or if they were at the allopatric equilibrium, again there are only two outcomes. We observe either convergence to one and the same polymorphic equilibrium, which maintains weak differentiation, or the loss of a trait allele (Figure 4, Figure 5 and Figure S5). In these cases, there is no initial increase of differentiation (in contrast to the cases when the initial populations had maximum linkage disequilibrium), but the long-term evolution is again complex in many cases (see Figure 5c–f and Figure S1e,f) with phases of decreasing and increasing differentiation.

### 3.3. Asymmetric Migration, Selection and Preference Strength

An asymmetry in the migration rate has the tendency to increase the loss of variation at the trait and preference loci. The case of unidirectional migration (a continent-island model) was analyzed in depth by [11], for both the phenotype matching model and the case of preferences and traits on separate chromosomes. They found that under phenotype matching, if the trait T_1_ is fixed on the continent (our population 1), a polymorphic equilibrium will often exist on the island (our population 2), determined by a balance between viability and sexual selection increasing the frequency of T_2_ and migration lowering it. When viability selection is weak, this polymorphic equilibrium representing trait divergence exists only at intermediate preference strengths (see also Figure S6a or Figure S7a, far left column). In a preference/trait model, however, Servedio and Bürger [11] found that maintenance of an island trait is possible only when preferences are sufficiently weak (see also Figure S6a or Figure S7a, far right column), unless the preference allele P_2_ is also present on the continent.

When examining the case of secondary contact of populations that have almost completely diverged in allopatry (starting with complete linkage disequilibrium) and assessing the role of tight physical linkage between the preference and trait, we find that with unidirectional migration, as with symmetrical migration, the equilibrium trait frequencies on the island resemble those with preferences and traits are on separate chromosomes (both when the trait is expressed only in males and when it is expressed in both sexes (Figure S6a or Figure S7a)). Inflated frequencies of the trait T_2_ on the island can remain present with tight linkage for long periods of time, but particularly when preferences are very strong, and the trait is expressed in both sexes (Figures S6a and S8). Strongly asymmetric migration rates, instead of unidirectional rates, exhibit similar findings, with the exception that when the trait is expressed in both sexes, trait and preference divergence can be maintained at very high preference strengths at equilibrium (Figure S6b vs. Figure S7b). Exploration of broader starting conditions with moderately strong preferences demonstrates that, as in the case with symmetric migration, high initial linkage disequilibrium is necessary for a temporary inflation of trait divergence to be observed (Figure S9).

Asymmetry in the strength of preference of P_1_ versus P_2_ females can also have a strong effect on preference and trait frequencies, and indeed on the ability of divergence to be maintained across populations. The patterns observed, however, are consistent with the ways in which we understand the mechanisms of phenotype matching and separate preferences and trait to work. We examined *α*_2_ > *α*_1_ when preferences and traits started at highly diverged frequencies (both starting in linkage equilibrium and with complete linkage disequilibrium), and examined the cases of selection in both sexes (Figure S10) and of selection on males alone (Figure S11). Convergence to equilibrium is slowed considerably when preference strengths are asymmetric, so while we examined the pattern present at one million generations post-contact, this should be understood to be only partway to an equilibrium state.

We find that even when the asymmetry in preference strengths is quite low, such that P_2_ females have a slightly stronger preference for T_2_ males than P_1_ females do for T_1_ males (e.g., between 1% and 5% stronger with selection in both sexes and <1% stronger with selection only in males), the mating trait T_2_ can fix across both populations in regions of parameter space and time in which evolution mimics a phenotype matching mechanism with strong sexual selection. This occurs with low recombination and intermediate preference strengths; under these conditions, a higher *α*_2_ will favor T_2_ globally across the two-population system, due to the fact that sexual selection is positive frequency-dependent when the system behaves as under phenotype matching.

On the other hand, the system is expected to behave as under separate preferences and traits when either the recombination rate is high or enough generations have passed under low recombination (see above). Under these conditions, we find that a higher preference of P_2_ females for T_2_ males tends to paradoxically lead to the loss of T_2_ if preferences are strong enough, but only if selection on T_2_ occurs in both sexes. A detailed examination of the changes in genotype frequencies through the life cycle shows that when selection occurs in both sexes, this loss of T_2_ can be explained by a complex interaction of viability selection in each population with frequency-dependent sexual selection, explained in full in Supplementary Materials File S5. This explanation hinges in part on the fact that when preferences are more homogenized than traits, which is expected in models with separate preference and trait loci [9], sexual selection tends to favor the rarer trait allele (though in this case of asymmetric preferences this is tempered by the differences in the preference strengths, see Supplementary Materials File S5). When selection on the trait only occurs in males, we find that strong but slightly-asymmetric preferences instead lead to the evolution of trait frequencies to a point that is close to that which is found with symmetric preferences (recall that when selection occurs only in males there is a unique equilibrium point instead of the line of equilibria that is found when selection is in both sexes; see above).

As with the case of asymmetric preference strengths, asymmetric selection coefficients between the populations can also lead to some unexpected results. We examined the case where the selection coefficient in population 2 was twice as large as that in population 1 (Figure S12), again looking at the patterns after 1 million generations. When selection occurs in both sexes, the increase in frequency in the locally adapted trait in females leads, within the viability selection stage of the life cycle, to an increase in the “local” preference due to linkage disequilibrium between the P and T loci (see [9]). The patterns in this case are relatively straightforward; we see an increase in population 2 of T_2_ (and P_2_) over the frequencies of T_1_ (and P_1_) in population 1 (Figure S12a). When selection occurs only in males, however, we see an unexpected dip in the frequencies of both preferences (P_1_ and P_2_) in population 2 when preferences are weak to moderate (Figure S12b).

## 4. Discussion

The evolutionary consequences of physical linkage between loci involved in reproductive isolation have sparked interest for over a decade. Studies have concentrated on the linkage between various types of components of reproductive isolation, including among loci involved in ecological adaptation and between the components of premating and postzygotic isolation (e.g., [24,25,26,27]). Here we study the effects of linkage between loci for female preferences and male traits, motivated by two observations: first, co-localization of such loci has been found in systems of interest to speciation researchers (e.g., [17,19,21]), and second, theoretical studies show that pleiotropy (phenotype matching) is expected to have drastically different evolutionary effects than preferences and traits on separate chromosomes (e.g., sympatric speciation: [7,8,12,14]; two-island model: [15] vs. [9]). We thus ask, will preferences and traits that are tightly physically linked mimic the behavior of ones that are controlled by a single, pleiotropic locus?

For secondary contact between geographically isolated populations, we find that the equilibrium reached with tight linkage between a preference and a trait does not mimic pleiotropy. Instead, this equilibrium is often much closer to that which is reached when preferences and traits are on separate chromosomes. Specifically, when traits are expressed in both sexes, we find a curve of equilibrium values that is independent of the (nonzero) recombination rate. The specific point to which preferences, traits, and the disequilibrium between them evolve depends on the recombination rate and on the starting frequencies. Importantly, it is often close to that reached under free recombination, especially if we start with populations that have diverged almost completely in preferences and traits before secondary contact. Additionally, often the locally advantageous trait allele and its matching preference in one of the subpopulations have frequencies below 50%, so that divergence will appear to be very limited. In the case in which the trait is expressed only in males, there instead exists a unique polymorphic equilibrium that is independent of the (non-zero) recombination rate and that typically exhibits weak differentiation; thus, this equilibrium is again far from the pleiotropic one.

Thus, for both cases, even when linkage is so tight as to be empirically indistinguishable from pleiotropy by mapping techniques, over the long run, loci do not act as if they are pleiotropic. However, if there is also initially strong linkage disequilibrium, tight physical linkage between preference and trait loci will cause populations to initially evolve towards the equilibrium predicted by pleiotropy (that expected under phenotype matching). The population will remain in the vicinity of this equilibrium for longer with lower recombination, evolving away only after ~1/*r* generations and taking ~10–1000 times as many generations or more to reach equilibrium. This behavior may lead researchers studying populations in recent contact to arrive at misleading conclusions regarding both the future of the populations and the effects of sexual selection on the speciation process. When tight linkage temporarily mimics phenotype matching in our model, for example, it leads to divergent sexual selection at intermediate preference strengths, causing trait divergence to sometimes be inflated far above migration-selection balance, or even allowing trait differentiation at all in cases where divergent ecological selection is otherwise too weak to support it. Trait and preference divergence may thus be inflated for many generations; this time span, ranging up to 10,000 generations for very tight linkage, encompasses times since secondary contact that are likely to be of interest for speciation biologists studying a very wide variety of taxa. At the same preference strengths, however, separate preference and trait loci will instead result in sexual selection causing populations to converge in their traits, compared to ecological selection alone. Sexual selection may thus promote long periods of divergence in studied populations that have tight linkage, yet in the absence of other evolutionary factors coming into play, convergence, rather than speciation, would be expected as an eventual outcome.

Given that the arrival of populations to the often-convergent equilibrium pattern of preferences and traits on separate chromosomes may take a very long time with tight linkage, we might expect additional factors not included in the model to affect the eventual evolutionary outcome. Mutations affecting preference strength, for example, might allow the evolution of choosiness. In our primary scenario of symmetric migration, the evolution of choosiness under phenotype matching leads to the preference strength that results in the largest degree of divergence between populations in trait frequencies [15]. However, a parallel model with separate preferences and traits has found that choosiness evolves to be progressively weaker [11]. Because the evolution of choosiness occurs very slowly in both of these models, we expect convergence to the pattern of separate preferences and traits would still occur, with the system evolving slowly towards random mating (even if there may be an initial tendency for choosiness to evolve to an intermediate level). Additionally, the prolonged separation of the populations allowed by tight linkage may allow the build-up of genes involved in post-zygotic isolation in natural populations, although this can be very restricted when there is ongoing gene flow [28,29,30]. The presence of postzygotic isolation would be expected to select for stronger choosiness through the process of reinforcement [31,32].

Whether the trait is expressed in both sexes or in males only, an imbalance of starting conditions in the two populations combined with low or moderate initial linkage disequilibrium frequently leads to loss of variation in the trait, even when all parameters (preference strength, selection coefficient, migration) are otherwise symmetric. This loss of variation is particularly likely with strong preferences and tightly linked loci, as well as if one preference allele is initially sufficiently common across both populations (e.g., fixed in one subpopulation, and at moderate frequency in the other). The question of speciation then becomes moot.

The loss of variation in the trait (and the preference) becomes even more likely when parameter values are asymmetric between the two populations. Loss of one of the traits is the most likely outcome under asymmetric migration, especially when the difference is extreme (e.g., a continent-island model). This is expected, as stronger selection is needed to overcome the swamping effect of migration and maintain trait polymorphism (e.g., [33]). Interesting instances of trait loss, with complex causes, also occur with asymmetric preference strengths and strengths of viability selection in each population. In these cases, the recombination rate between preference and trait loci has a larger effect on the equilibrium reached—both whether trait loss will occur and, for the case of selection in both sexes, what point on the line of equilibria will be reached. Asymmetries, however, do not alter our main conclusion that tight linkage does not allow separate preference and trait loci to mimic a single pleiotropic locus. They also do not, in any of the cases examined, cause more divergence in preferences and traits across populations than when parameters are symmetrical (though this may potentially occur when there are simultaneous asymmetries in different directions between different evolutionary forces).

Both a wide variety of parameter values and starting conditions matching those in our models could potentially be found in nature. It is reasonable to think, however, that populations might be in equilibrium in allopatry before secondary contact occurs. We addressed this situation by including analyses that started at equilibrium for a single population under sexual selection. These conditions were originally analyzed (for selection in males alone; selection in both sexes is similar) by [23], who found a line of polymorphic equilibria when there are no costs to preference (when preference is costly this line is eliminated, [34]). We examined starting populations with preference and trait frequencies chosen from a number of points on this line. However, many of these points have relatively low preference frequencies (below 0.5), even when trait frequencies are high. While strictly matching the equilibrium conditions of this population genetic model, these conditions will doubtless seem strange to many biologists, who often imagine allopatric populations starting with divergent preferences and traits. If such divergence were to occur because of drift or another historical contingency, we would instead have starting conditions very close to our initial analyses: high divergence, perhaps with high linkage disequilibrium. Another cause of allopatric preference and trait divergence could be divergent selection on preferences; this should result in the preference that is favored by selection becoming fixed in each population, and the trait following, contingent on the strength and direction of sexual and viability selection [34]. While we do not include divergent direct selection on preferences in our model, we expect that it would greatly increase the capability of populations to maintain preference and trait divergence after contact (see, e.g., [6,8,35,36,37,38,39]).

Our findings have implications for the power of chromosomal inversions to affect divergence between species when they capture loci affecting both mating preferences and mating traits. Rates of recombination within chromosomal inversions appear to vary widely. Stevison et al. [40], for example, found a double crossover rate of 10^−4^ within a ~12.5 Mb inversion in *Drosophila pseudoobscura* and *Drosophila persimilis*, while [41] found rates of 0.004 and 0.13 for two different pericentric inversions in human chromosomes. Another estimate within *Drosophila melanogaster* found the recombination rate within inversions to be 25% of the rate for a comparable region outside of an inversion [42]. For all but the largest of these rates, it is very likely that inversions containing preferences and traits will lead to divergence patterns mimicking those of phenotype matching for very many generations, provided that preferences and traits have high linkage disequilibrium at the time of contact. However, our results also predict that, in the long run, inversions do not maintain divergence. This result is similar to that found in a model of inversions by Feder and Nosil [27], who noted that very small amounts of recombination (rates of 10^−8^) between loci involved in Dobzhansky-Muller incompatibilities resulted in very different long-term outcomes for differentiation than did no recombination.

We model preferences and traits by single loci, but they are likely to be polygenic in natural systems. The effects of sexual selection on trait divergence documented in our preference/trait model are likely to also hold for “absolute” preferences (e.g., with unimodal preference functions for specific trait values) when traits and preferences are polygenic. This is because when absolute preferences are similar (i.e., relatively “homogenized”) between populations, they will select for traits that are correspondingly close together in their values [10]; this is the analog of homogenized preference frequencies causing homogenization of trait frequencies in the discrete-locus case [9]. Lande [10], however, found that sexual selection tends to promote, rather than inhibit trait divergence under two other forms of preference functions for continuous traits. When traits and preference are controlled by many loci the issue of pleiotropy becomes complex. It is not likely that every single locus between preferences and traits would contain pleiotropic alleles unless there is a mechanistic constraint underlying this relationship. Self-reference phenotype matching [43], for example, would cause the trait phenotype to directly affect the preference phenotype; alleles at every locus affecting the trait would by definition also affect the preference. Under self-reference, previous models predict a pattern of divergence similar to that in Figure 1a when the number of loci is relatively low and viability selection is divergent, but not necessarily for very large numbers of loci or stabilizing selection on traits [15,44,45].

We conclude by focusing more closely on the implications of our results for the role of sexual selection in speciation. Our findings imply that the conclusions of [9] for the discrete-locus case, that sexual selection plays a causal role in inhibiting divergence in many cases, holds at long time scales for tight linkage as well. The exception found by [9], when selection on traits occurs in both sexes and preferences are very strong, is shown to be more singular given the results from the much broader set of cases considered in the current work. We find that many different starting conditions as well as departures from symmetry in migration rates (see also [11]), preference strengths, and selection coefficients will lead to loss of one of the traits across the entire system, and observe that frequency-dependent sexual selection often contributes to this loss of trait polymorphism. Finally, when traits and preferences remain polymorphic but are at similar frequencies across both systems, as when traits are expressed only in males and preferences are strong, the equilibrium can be interpreted as the evolution of two isolated groups; this is the case because associations between preferences and traits will be strong even if neither associated pair is characteristic of a particular population. However, because preference strength itself will weaken evolutionarily [9], such isolated groups will not be expected to be stable. In general, we thus continue to conclude that sexual selection will maintain, or promote divergence only rarely with the type of preference considered here.

## Supplementary Materials

The following are available online at http://www.mdpi.com/2073-4425/9/4/217/s1. Figure S1: Detailed temporal evolution for selected initial conditions, Figure S2: Preference and trait divergence 100, 1000, 10,000 and 1 million generations after secondary contact when the trait is expressed in both sexes, Figure S3: The effects of initial conditions and recombination rates on evolutionary trajectories with symmetric initial conditions, Figure S4: The effects of initial conditions and recombination rates on evolutionary trajectories when the trait is expressed in both sexes and migration is symmetric and very small, Figure S5: Preference and trait divergence at 100, 1000, 10,000, and 1 million generations after secondary contact when the trait is expressed only in males, Figure S6: Preference and trait divergence at 100, 1000, 10,000, and 1 million generations after secondary contact when the trait is expressed in both sexes, Figure S7: Preference and trait divergence at 100, 1000, 10,000, and 1 million generations after secondary contact when the trait is expressed only in males, Figure S8: The effects of initial conditions and recombination rates on evolutionary trajectories when the trait is expressed in both sexes and there is continent-to-island migration, Figure S9: The effects of initial conditions and asymmetric migration on evolutionary trajectories when the trait is expressed in both sexes, Figure S10: Preference and trait divergence at 1 million generations after secondary contact when the trait is expressed in both sexes and preference strengths are asymmetric, Figure S11: Preference and trait divergence at 1 million generations after secondary contact when the trait is expressed in only in males and preference strengths are asymmetric, Figure S12: Preference and trait divergence at 1 million generations after secondary contact when viability selection strengths are asymmetric, Supplementary Materials File S1: Simulations starting with arbitrary amounts of divergence, Supplementary Materials File S2: Simulations starting from allopatric equilibrium, Supplementary Materials File S3: The preference/trait model with natural selection on both sexes, recombination between the preference and trait loci, and migration between two demes, Supplementary Materials File S4: The preference/trait model with natural selection only on males, recombination between the reference and trait loci, and migration between two demes, Supplementary Materials File S5: Asymmetric preferences.
