# Supplementary material for: The Effects on Parapatric Divergence of Linkage between Preference and Trait Loci versus Pleiotropy
Source: Genes (Basel). 2018 Apr 17;9(4):217. doi: 10.3390/genes9040217 (PMC5924559; doi:10.3390/genes9040217)
Supplement: Supplementary file 1 [file FigureS10.pdf › Servedio and Burger Supplementary Information proofs/FigureS10.pdf]

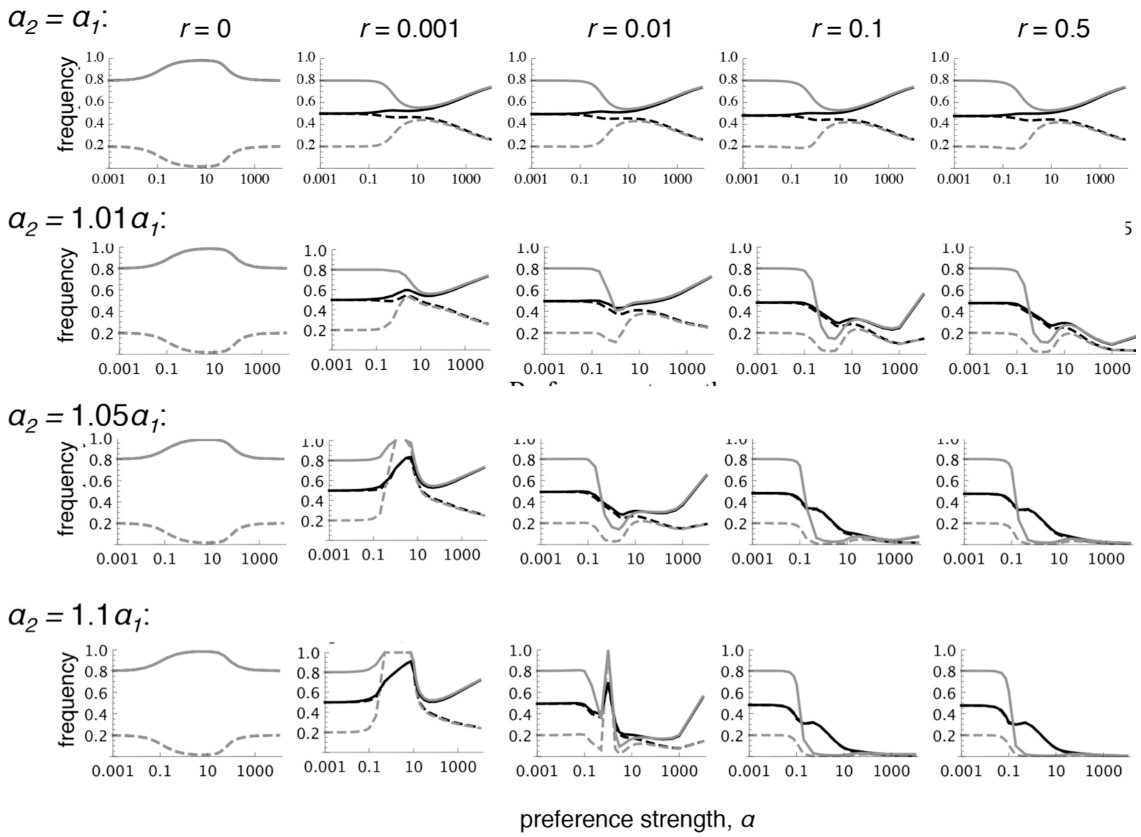

Figure S10. Preference and trait divergence at 1 million generations after secondary contact when the trait is expressed in both sexes and preference strengths are asymmetric (as marked). The parameter values and legend follow Figure 1.
