# Supplementary material for: The Effects on Parapatric Divergence of Linkage between Preference and Trait Loci versus Pleiotropy
Source: Genes (Basel). 2018 Apr 17;9(4):217. doi: 10.3390/genes9040217 (PMC5924559; doi:10.3390/genes9040217)
Supplement: Supplementary file 1 [file FigureS5.pdf › Servedio and Burger Supplementary Information proofs/FigureS5.pdf]

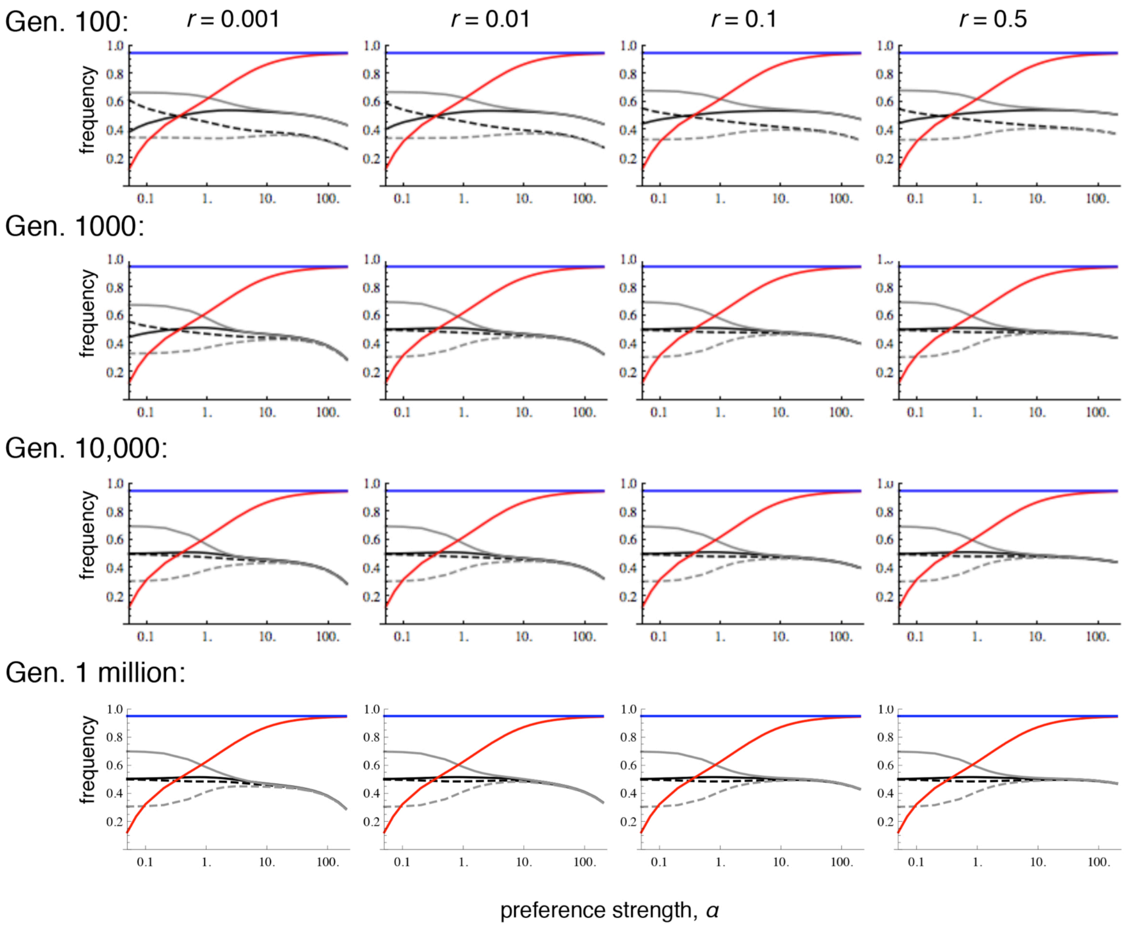

Figure S5. Preference and trait divergence at 100, 1000, 10,000, and 1 million generations after secondary contact when the trait is expressed only in males. The parameter values and legend follow Figure S2. The Red lines are now calculated assuming the trait T_2 (where again t_2 = 0.95) is expressed only in males.
