# Supplementary material for: The Effects on Parapatric Divergence of Linkage between Preference and Trait Loci versus Pleiotropy
Source: Genes (Basel). 2018 Apr 17;9(4):217. doi: 10.3390/genes9040217 (PMC5924559; doi:10.3390/genes9040217)
Supplement: Supplementary file 1 [file genes-09-00217-s001.zip › Servedio and Burger Supplementary Information proofs/FigureS9.pdf]

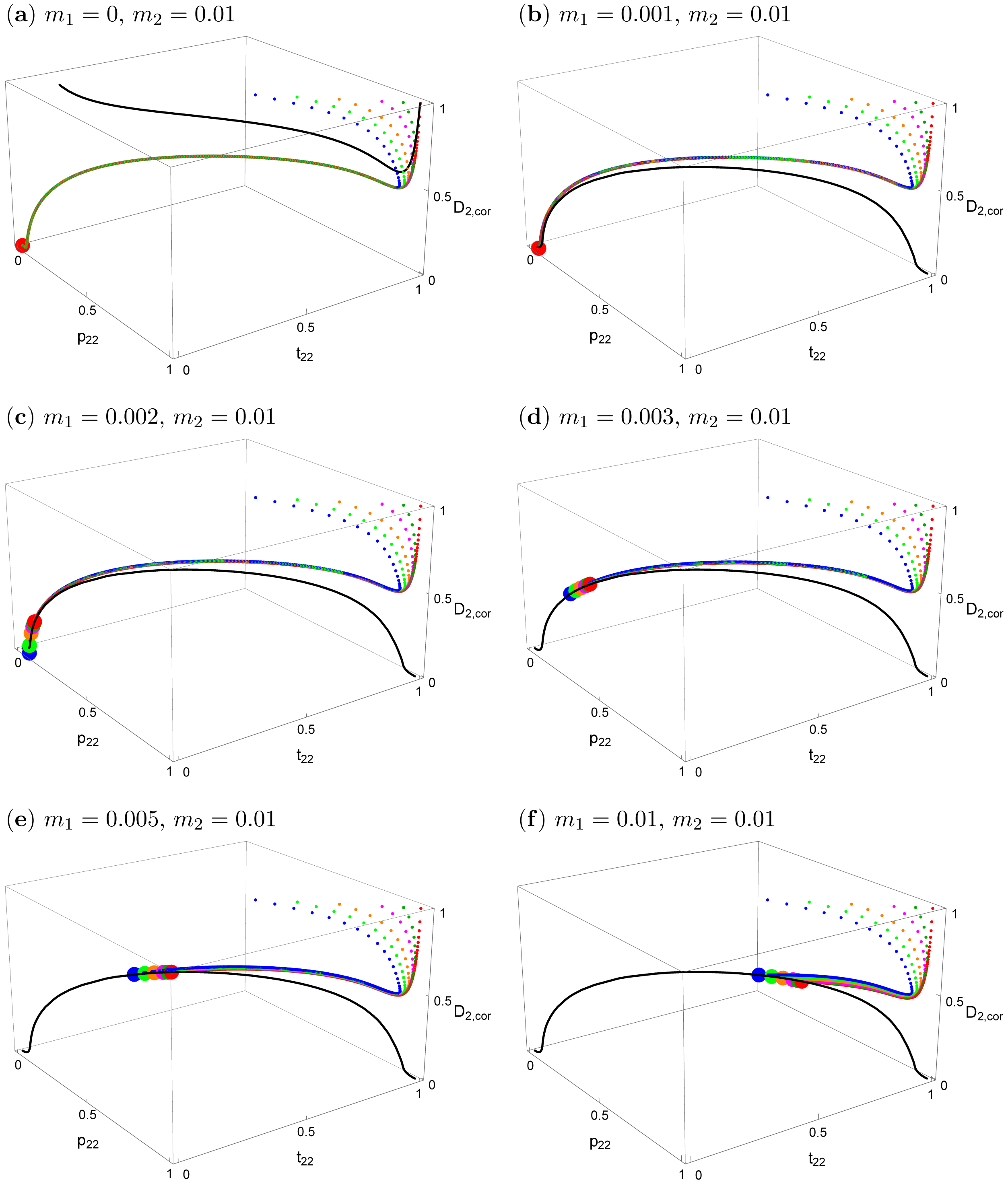

Figure S9. The effects of initial conditions and asymmetric migration on evolutionary trajectories when the trait is expressed in both sexes. The black curve is the curve of all stable equilibria. Small dots in different colors show evolutionary trajectories in population 2. The big colored dots show the equilibrium that is reached. In all panels, population 1 is initially fixed for P_1 and T_1, and the initial allele frequencies in population 2 are $p_{2,2} = t_{2,2} = 0.99$ (red), 0.95 (dark green), 0.90 (magenta), 0.80 (orange), 0.70 (light green), 0.60 (blue). In population 2, there is maximum initial linkage disequilibrium, i.e., $D_{2,\mathrm{cor}} = 1$. Other parameters are $s_1 = s_2 = 0.2$, $\alpha_1 = \alpha_2 = 10$, and $r = 0.1$.
