# Supplementary material for: The Effects on Parapatric Divergence of Linkage between Preference and Trait Loci versus Pleiotropy
Source: Genes (Basel). 2018 Apr 17;9(4):217. doi: 10.3390/genes9040217 (PMC5924559; doi:10.3390/genes9040217)
Supplement: Supplementary file 1 [file genes-09-00217-s001.zip › Servedio and Burger Supplementary Information proofs/FigureS12.pdf]

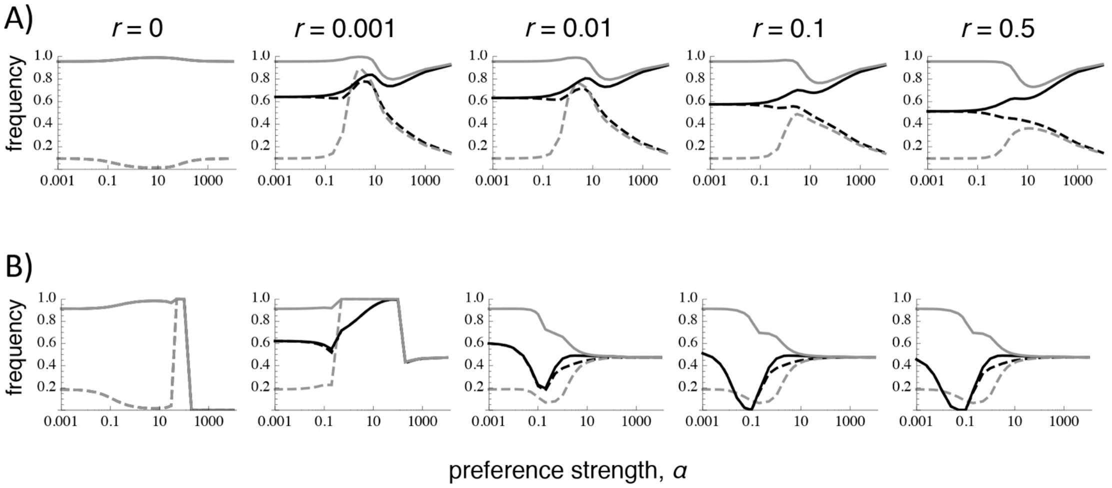

Figure S12. Preference and trait divergence at 1 million generations after secondary contact when viability selection strengths are asymmetric, such that s_1 = 0.1 and s_2 = 0.2. Other parameter values and legend follow Figure 1. a) viability selection occurs in both sexes, and b) viability selection occurs only in males.
