# Supplementary material for: The Effects on Parapatric Divergence of Linkage between Preference and Trait Loci versus Pleiotropy
Source: Genes (Basel). 2018 Apr 17;9(4):217. doi: 10.3390/genes9040217 (PMC5924559; doi:10.3390/genes9040217)
Supplement: Supplementary file 1 [file genes-09-00217-s001.zip › Servedio and Burger Supplementary Information proofs/Supplementary Materials File S1.pdf]

Simulations starting with arbitrary amounts of divergence

From Servedio and Bürger, The Effects on Parapatric Divergence of Linkage between Preference and Trait Loci versus Pleiotropy, *Genes*

Double click on the arrows on the right-hand side to open each sub-section

Simulation program with T expressed in both sexes

This program can be set for any starting condition, though the example below starts with p2, t2 in pop 2 at 0.95 and p1, t1 in pop 1 at 1 (full LD). The example below is run at 1 million generations.

```
PTmigprog := (
  c1 =  65; (* number of items in alphaparams *)
  k1c = 1; (* number of items in p2arams *)
  k2c = 1; (* number of items in t2params *)
  eqp2pop1 = Table[0, {count, c1}];
  eqt2pop1 = Table[0, {count, c1}];
  eqdiseqpop1 = Table[0, {count, c1}];
  eqp2pop2 = Table[0, {count, c1}];
  eqt2pop2 = Table[0, {count, c1}];
  eqdiseqpop2 = Table[0, {count, c1}];
  eqp2popdiff = Table[0, {count, c1}];
  eqt2popdiff = Table[0, {count, c1}];
  (*plot1=Table[0,{count,c1},{k2,k2c},{k1,k1c}];
  plot2=Table[0,{count,c1},{k2,k2c},{k1,k1c}];*)
  Do[αparams = {0.001, 0.0025, 0.005, 0.0075, 0.01, 0.025, 0.05, 0.06, 0.07, 0.08, 0.09,
     0.1, 0.11, 0.12, 0.13, 0.14, 0.15, 0.175, 0.2, 0.25, 0.3, 0.35, 0.4, 0.5, 0.6,
     0.7, 0.8, 0.9, 1.0, 1.5, 2.0, 3.0, 5.0, 6.0, 7.0, 8.0, 9.0, 10.0 , 11.0, 12.0,
     13.0, 14.0, 15.0, 16.0, 17.0, 18.0, 19.0, 20.0, 22.5, 25.0, 30.0, 40.0, 50.0, 75.0,
     100.0, 150.0, 200.0, 300.0, 400.0, 500.0, 700.0, 1000.0, 2000.0, 5000.0, 10000.0};
   α2 = αparams[[count]];
   α1 = αparams[[count]];
   gen = 1;

   (*x1[1] = (1-p12) (1-t12) +diseq1;
   x1[2] = (1-p12)t12- diseq1;
   x1[3] = p12 (1-t12) - diseq1;
   x1[4] = p12 t12  + diseq1;
```

```
x2[1] = (1-p22) (1-t22) +diseq2;
x2[2] = (1-p22)t22- diseq2;
x2[3] = p22 (1-t22) - diseq2;
x2[4] = p22 t22  + diseq2;*)

(*override allele freqs for full LD*)
x1[1] = 1.0;
x1[2] = 0;
x1[3] = 0;
x1[4] = 0;
x2[1] = 0.05;
x2[2] = 0;
x2[3] = 0;
x2[4] = 0.95;

Do[ x1t1[cc] = 0, {cc, 1, 4}];
Do[ x2t1[cc] = 0, {cc, 1, 4}];

F1 = Table[0, {i, 4}, {j, 4}];
F2 = Table[0, {i, 4}, {j, 4}];

Coef = Table[1, {i, 4}, {j, 4}];
Do[Coef[[i, 1]] = (1 + α1), {i, 1, 2}];
Do[Coef[[i, 3]] = (1 + α1), {i, 1, 2}];
Do[Coef[[i, 2]] = (1 + α2), {i, 3, 4}];
Do[Coef[[i, 4]] = (1 + α2), {i, 3, 4}];

While[(gen < 1 000 000),


 If[gen > 1,
  Do[x1[cc] = x1t1[cc], {cc, 1, 4}];
  Do[x2[cc] = x2t1[cc], {cc, 1, 4}]]; (* recursions *)


 x1m[1] = (1 - m1) x1[1] + m1 x2[1];
 x1m[2] = (1 - m1) x1[2] + m1 x2[2];
 x1m[3] = (1 - m1) x1[3] + m1 x2[3];
 x1m[4] = (1 - m1) x1[4] + m1 x2[4];

 x2m[1] = (1 - m2) x2[1] + m2 x1[1];
 x2m[2] = (1 - m2) x2[2] + m2 x1[2];
 x2m[3] = (1 - m2) x2[3] + m2 x1[3];
 x2m[4] = (1 - m2) x2[4] + m2 x1[4];

 w1barm = x1m[1] (1 + s) + x1m[2] + x1m[3] (1 + s) + x1m[4];
```

```
xnsm1[1] = x1m[1] (1 + s) / w1barm;
xnsm1[2] = x1m[2] / w1barm;
xnsm1[3] = x1m[3] (1 + s) / w1barm;
xnsm1[4] = x1m[4] / w1barm;

Do[xnsf1[i] = xnsm1[i], {i, 4}];

w2barm =  x2m[1] + (1 + s) x2m[2] + x2m[3] + (1 + s) x2m[4];
xnsm2[1] = x2m[1] / w2barm;
xnsm2[2] = (1 + s) x2m[2] / w2barm;
xnsm2[3] = x2m[3] / w2barm;
xnsm2[4] = (1 + s) x2m[4] / w2barm;

Do[xnsf2[i] = xnsm2[i], {i, 4}];

Mate1 = Table[xnsf1[i] xnsm1[j], {i, 4}, {j, 4}];
G1 = Mate1 Coef;

Do[z1[i] = Sum[xnsm1[j] Coef[[i, j]], {j, 4}], {i, 4}];

Do[F1[[i, j]] = G1[[i, j]] / z1[i], {i, 4}, {j, 4}];

Mate2 = Table[xnsf2[i] xnsm2[j], {i, 4}, {j, 4}];
G2 = Mate2 Coef;

Do[z2[i] = Sum[xnsm2[j] Coef[[i, j]], {j, 4}], {i, 4}];

Do[F2[[i, j]] = G2[[i, j]] / z2[i], {i, 4}, {j, 4}];

x1t1[1] = Simplify[F1[[1, 1]] + (1 / 2) F1[[1, 2]] + (1 / 2) F1[[1, 3]] +
    (1 / 2) (1 - r) F1[[1, 4]] + (1 / 2) F1[[2, 1]] + (1 / 2) r F1[[2, 3]] +
    (1 / 2) F1[[3, 1]] + (1 / 2) r F1[[3, 2]] + (1 / 2) (1 - r) F1[[4, 1]]];
x1t1[2] = Simplify[(1 / 2) F1[[1, 2]] + (1 / 2) r F1[[1, 4]] + (1 / 2) F1[[2, 1]] +
    F1[[2, 2]] + (1 / 2) (1 - r) F1[[2, 3]] + (1 / 2) F1[[2, 4]] +
    (1 / 2) (1 - r) F1[[3, 2]] + (1 / 2) r F1[[4, 1]] + (1 / 2) F1[[4, 2]]];
x1t1[3] = Simplify[(1 / 2) F1[[1, 3]] + (1 / 2) r F1[[1, 4]] + (1 / 2) (1 - r) F1[[2, 3]] +
    (1 / 2) F1[[3, 1]] + (1 / 2) (1 - r) F1[[3, 2]] + F1[[3, 3]] +
    (1 / 2) F1[[3, 4]] + (1 / 2) r F1[[4, 1]] + (1 / 2) F1[[4, 3]]];
x1t1[4] = Simplify[(1 / 2) (1 - r) F1[[1, 4]] + (1 / 2) r F1[[2, 3]] +
    (1 / 2) F1[[2, 4]] + (1 / 2) r F1[[3, 2]] + (1 / 2) F1[[3, 4]] +
    (1 / 2) (1 - r) F1[[4, 1]] + (1 / 2) F1[[4, 2]] + (1 / 2) F1[[4, 3]] + F1[[4, 4]]];

x2t1[1] = Simplify[F2[[1, 1]] + (1 / 2) F2[[1, 2]] + (1 / 2) F2[[1, 3]] +
    (1 / 2) (1 - r) F2[[1, 4]] + (1 / 2) F2[[2, 1]] + (1 / 2) r F2[[2, 3]] +
    (1 / 2) F2[[3, 1]] + (1 / 2) r F2[[3, 2]] + (1 / 2) (1 - r) F2[[4, 1]]];
x2t1[2] = Simplify[(1 / 2) F2[[1, 2]] + (1 / 2) r F2[[1, 4]] + (1 / 2) F2[[2, 1]] +
```

```
        F2[[2, 2]] + (1 / 2) (1 - r) F2[[2, 3]] + (1 / 2) F2[[2, 4]] +
        (1 / 2) (1 - r) F2[[3, 2]] + (1 / 2) r F2[[4, 1]] + (1 / 2) F2[[4, 2]]]];
    x2t1[3] = Simplify[(1 / 2) F2[[1, 3]] + (1 / 2) r F2[[1, 4]] + (1 / 2) (1 - r) F2[[2, 3]] +
        (1 / 2) F2[[3, 1]] + (1 / 2) (1 - r) F2[[3, 2]] + F2[[3, 3]] +
        (1 / 2) F2[[3, 4]] + (1 / 2) r F2[[4, 1]] + (1 / 2) F2[[4, 3]]]];
    x2t1[4] = Simplify[(1 / 2) (1 - r) F2[[1, 4]] + (1 / 2) r F2[[2, 3]] +
        (1 / 2) F2[[2, 4]] + (1 / 2) r F2[[3, 2]] + (1 / 2) F2[[3, 4]] +
        (1 / 2) (1 - r) F2[[4, 1]] + (1 / 2) F2[[4, 2]] + (1 / 2) F2[[4, 3]] + F2[[4, 4]]]];

    gen++;]; (* loop *)

   p1t1 = Simplify[x1t1[3] + x1t1[4]];
   t1t1 = Simplify[x1t1[2] + x1t1[4]];
   diseq1t1 = x1t1[1] x1t1[4] - x1t1[2] x1t1[3];
   p2t1 = Simplify[x2t1[3] + x2t1[4]];
   t2t1 = Simplify[x2t1[2] + x2t1[4]];
   diseq2t1 = x2t1[1] x2t1[4] - x2t1[2] x2t1[3];

   Print["run ", count, ",  α = ", α1, ",   gen = ", gen];

   eqp2pop1[[count]] = {αparams[[count]], p1t1};
   eqt2pop1[[count]] = {αparams[[count]], t1t1};
   eqdiseqpop1[[count]] = {αparams[[count]], diseq1t1};
   eqp2pop2[[count]] = {αparams[[count]], p2t1};
   eqt2pop2[[count]] = {αparams[[count]], t2t1};
   eqdiseqpop2[[count]] = {αparams[[count]], diseq2t1};
   eqp2popdiff[[count]] = {αparams[[count]], p2t1 - p1t1};
   eqt2popdiff[[count]] = {αparams[[count]], t2t1 - t1t1},
   {count, c1}]
 )
```

Example of code needed to run this:

```
pC = 0;
s = 0.038;
r = 0;
m1 = 0;
m2 = 0.01;
PTmigprog
```

Simulation program with T expressed only in males

This program can be set for any starting condition, though the example below starts with p2, t2 in pop 2 at 0.95 and p1, t1 in pop 1 at 1 (full LD). The example below is run at 1 million generations.

```
PTmigprog := (
  c1 =  65; (* number of items in alphaparams *)
  k1c = 1; (* number of items in p2arams *)
  k2c = 1; (* number of items in t2params *)
  eqp2pop1 = Table[0, {count, c1}];
  eqt2pop1 = Table[0, {count, c1}];
  eqdiseqpop1 = Table[0, {count, c1}];
  eqp2pop2 = Table[0, {count, c1}];
  eqt2pop2 = Table[0, {count, c1}];
  eqdiseqpop2 = Table[0, {count, c1}];
  eqp2popdiff = Table[0, {count, c1}];
  eqt2popdiff = Table[0, {count, c1}];
  (*plot1=Table[0,{count,c1},{k2,k2c},{k1,k1c}];
  plot2=Table[0,{count,c1},{k2,k2c},{k1,k1c}];*)
  Do[αparams = {0.001, 0.0025, 0.005, 0.0075, 0.01, 0.025, 0.05, 0.06, 0.07, 0.08, 0.09,
     0.1, 0.11, 0.12, 0.13, 0.14, 0.15, 0.175, 0.2, 0.25, 0.3, 0.35, 0.4, 0.5, 0.6,
     0.7, 0.8, 0.9, 1.0, 1.5, 2.0, 3.0, 5.0, 6.0, 7.0, 8.0, 9.0, 10.0 , 11.0, 12.0,
     13.0, 14.0, 15.0, 16.0, 17.0, 18.0, 19.0, 20.0, 22.5, 25.0, 30.0, 40.0, 50.0, 75.0,
     100.0, 150.0, 200.0, 300.0, 400.0, 500.0, 700.0, 1000.0, 2000.0, 5000.0, 10000.0};
   α2 = αparams[[count]];
   α1 = αparams[[count]];
   gen = 1;

   (*To start in LE
     x1[1] = (1-p12) (1-t12) +diseq1;
   x1[2] = (1-p12)t12- diseq1;
   x1[3] = p12 (1-t12) - diseq1;
   x1[4] = p12 t12  + diseq1;
   x2[1] = (1-p22) (1-t22) +diseq2;
   x2[2] = (1-p22)t22- diseq2;
   x2[3] = p22 (1-t22) - diseq2;
   x2[4] = p22 t22  + diseq2;*)

   (*override allele freqs for full LD*)
   x1[1] = 1.0;
   x1[2] = 0;
   x1[3] = 0;
   x1[4] = 0;
   x2[1] = 0.05;
   x2[2] = 0;
   x2[3] = 0;
   x2[4] = 0.95;

   Do[ x1t1[cc] = 0, {cc, 1, 4}];
   Do[ x2t1[cc] = 0, {cc, 1, 4}];
```

```
F1 = Table[0, {i, 4}, {j, 4}];
F2 = Table[0, {i, 4}, {j, 4}];

Coef = Table[1, {i, 4}, {j, 4}];
Do[Coef[[i, 1]] = (1 + α1), {i, 1, 2}];
Do[Coef[[i, 3]] = (1 + α1), {i, 1, 2}];
Do[Coef[[i, 2]] = (1 + α2), {i, 3, 4}];
Do[Coef[[i, 4]] = (1 + α2), {i, 3, 4}];

While[(gen < 1 000 000),


 If[gen > 1,
  Do[x1[cc] = x1t1[cc], {cc, 1, 4}];
  Do[x2[cc] = x2t1[cc], {cc, 1, 4}]]; (* recursions *)



 x1m[1] = (1 - m1) x1[1] + m1 x2[1];
 x1m[2] = (1 - m1) x1[2] + m1 x2[2];
 x1m[3] = (1 - m1) x1[3] + m1 x2[3];
 x1m[4] = (1 - m1) x1[4] + m1 x2[4];

 x2m[1] = (1 - m2) x2[1] + m2 x1[1];
 x2m[2] = (1 - m2) x2[2] + m2 x1[2];
 x2m[3] = (1 - m2) x2[3] + m2 x1[3];
 x2m[4] = (1 - m2) x2[4] + m2 x1[4];

 w1barm = x1m[1] (1 + s) + x1m[2] + x1m[3] (1 + s) + x1m[4];
 xnsm1[1] = x1m[1] (1 + s) / w1barm;
 xnsm1[2] = x1m[2] / w1barm;
 xnsm1[3] = x1m[3] (1 + s) / w1barm;
 xnsm1[4] = x1m[4] / w1barm;

 Do[xnsf1[i] = x1m[i], {i, 4}]; (* Trait not expressed in females *)

 w2barm =  x2m[1] + (1 + s) x2m[2] + x2m[3] + (1 + s) x2m[4];
 xnsm2[1] = x2m[1] / w2barm;
 xnsm2[2] = (1 + s) x2m[2] / w2barm;
 xnsm2[3] = x2m[3] / w2barm;
 xnsm2[4] = (1 + s) x2m[4] / w2barm;

 Do[xnsf2[i] = x2m[i], {i, 4}]; (* Trait not expressed in females *)

 Mate1 = Table[xnsf1[i] xnsm1[j], {i, 4}, {j, 4}];
 G1 = Mate1 Coef;
```

```
Do[z1[i] = Sum[xnsm1[j] Coef[[i, j]], {j, 4}], {i, 4}];

Do[F1[[i, j]] = G1[[i, j]] / z1[i], {i, 4}, {j, 4}];

Mate2 = Table[xnsf2[i] xnsm2[j], {i, 4}, {j, 4}];
G2 = Mate2 Coef;

Do[z2[i] = Sum[xnsm2[j] Coef[[i, j]], {j, 4}], {i, 4}];

Do[F2[[i, j]] = G2[[i, j]] / z2[i], {i, 4}, {j, 4}];

x1t1[1] = Simplify[F1[[1, 1]] + (1 / 2) F1[[1, 2]] + (1 / 2) F1[[1, 3]] +
    (1 / 2) (1 - r) F1[[1, 4]] + (1 / 2) F1[[2, 1]] + (1 / 2) r F1[[2, 3]] +
    (1 / 2) F1[[3, 1]] + (1 / 2) r F1[[3, 2]] + (1 / 2) (1 - r) F1[[4, 1]]];
x1t1[2] = Simplify[(1 / 2) F1[[1, 2]] + (1 / 2) r F1[[1, 4]] + (1 / 2) F1[[2, 1]] +
    F1[[2, 2]] + (1 / 2) (1 - r) F1[[2, 3]] + (1 / 2) F1[[2, 4]] +
    (1 / 2) (1 - r) F1[[3, 2]] + (1 / 2) r F1[[4, 1]] + (1 / 2) F1[[4, 2]]];
x1t1[3] = Simplify[(1 / 2) F1[[1, 3]] + (1 / 2) r F1[[1, 4]] + (1 / 2) (1 - r) F1[[2, 3]] +
    (1 / 2) F1[[3, 1]] + (1 / 2) (1 - r) F1[[3, 2]] + F1[[3, 3]] +
    (1 / 2) F1[[3, 4]] + (1 / 2) r F1[[4, 1]] + (1 / 2) F1[[4, 3]]];
x1t1[4] = Simplify[(1 / 2) (1 - r) F1[[1, 4]] + (1 / 2) r F1[[2, 3]] +
    (1 / 2) F1[[2, 4]] + (1 / 2) r F1[[3, 2]] + (1 / 2) F1[[3, 4]] +
    (1 / 2) (1 - r) F1[[4, 1]] + (1 / 2) F1[[4, 2]] + (1 / 2) F1[[4, 3]] + F1[[4, 4]]];

x2t1[1] = Simplify[F2[[1, 1]] + (1 / 2) F2[[1, 2]] + (1 / 2) F2[[1, 3]] +
    (1 / 2) (1 - r) F2[[1, 4]] + (1 / 2) F2[[2, 1]] + (1 / 2) r F2[[2, 3]] +
    (1 / 2) F2[[3, 1]] + (1 / 2) r F2[[3, 2]] + (1 / 2) (1 - r) F2[[4, 1]]];
x2t1[2] = Simplify[(1 / 2) F2[[1, 2]] + (1 / 2) r F2[[1, 4]] + (1 / 2) F2[[2, 1]] +
    F2[[2, 2]] + (1 / 2) (1 - r) F2[[2, 3]] + (1 / 2) F2[[2, 4]] +
    (1 / 2) (1 - r) F2[[3, 2]] + (1 / 2) r F2[[4, 1]] + (1 / 2) F2[[4, 2]]];
x2t1[3] = Simplify[(1 / 2) F2[[1, 3]] + (1 / 2) r F2[[1, 4]] + (1 / 2) (1 - r) F2[[2, 3]] +
    (1 / 2) F2[[3, 1]] + (1 / 2) (1 - r) F2[[3, 2]] + F2[[3, 3]] +
    (1 / 2) F2[[3, 4]] + (1 / 2) r F2[[4, 1]] + (1 / 2) F2[[4, 3]]];
x2t1[4] = Simplify[(1 / 2) (1 - r) F2[[1, 4]] + (1 / 2) r F2[[2, 3]] +
    (1 / 2) F2[[2, 4]] + (1 / 2) r F2[[3, 2]] + (1 / 2) F2[[3, 4]] +
    (1 / 2) (1 - r) F2[[4, 1]] + (1 / 2) F2[[4, 2]] + (1 / 2) F2[[4, 3]] + F2[[4, 4]]];

gen++;]; (* loop *)

p1t1 = Simplify[x1t1[3] + x1t1[4]];
t1t1 = Simplify[x1t1[2] + x1t1[4]];
diseq1t1 = x1t1[1] x1t1[4] - x1t1[2] x1t1[3];
p2t1 = Simplify[x2t1[3] + x2t1[4]];
t2t1 = Simplify[x2t1[2] + x2t1[4]];
diseq2t1 = x2t1[1] x2t1[4] - x2t1[2] x2t1[3];

Print["run ", count, ",  α = ", α1, ",   gen = ", gen];
```

```
    eqp2pop1[[count]] = {αparams[[count]], p1t1};
    eqt2pop1[[count]] = {αparams[[count]], t1t1};
    eqdiseqpop1[[count]] = {αparams[[count]], diseq1t1};
    eqp2pop2[[count]] = {αparams[[count]], p2t1};
    eqt2pop2[[count]] = {αparams[[count]], t2t1};
    eqdiseqpop2[[count]] = {αparams[[count]], diseq2t1};
    eqp2popdiff[[count]] = {αparams[[count]], p2t1 - p1t1};
    eqt2popdiff[[count]] = {αparams[[count]], t2t1 - t1t1},
    {count, c1}]
  )
```

Example of code needed to run this:

```
pC = 0;
s = 0.038;
r = 0;
m1 = 0;
m2 = 0.01;
PTmigprog
```
