# Supplementary material for: The Effects on Parapatric Divergence of Linkage between Preference and Trait Loci versus Pleiotropy
Source: Genes (Basel). 2018 Apr 17;9(4):217. doi: 10.3390/genes9040217 (PMC5924559; doi:10.3390/genes9040217)
Supplement: Supplementary file 1 [file genes-09-00217-s001.zip › Servedio and Burger Supplementary Information proofs/Supplementary Materials File S3.pdf]

Supplementary Material File S3:

The preference/trait model with natural selection on both sexes, recombination between the preference and trait loci, and migration between two demes

by

Maria Servedio and Reinhard Bürger

■ 1. Model and derivation of recursion relations

P2 is a preference allele for T2, with strength $\alpha 2$,
P1 is a preference allele for T1, with strength $\alpha 1$.
Migration into deme 1 occurs at rate $m1$, i.e., a fraction $m1$ of individuals in deme 1 is replaced by individuals from deme 2; migration into deme 2 occurs at rate $m2$.
T1 (T2) is favored by viability selection in both sexes in deme 1 (2) with selection coefficient $s1$ ($s2$).
The recombination rate between the P and T locus is $r \geq 0$.

There are the four genotypes P1T1, P1T2, P2T1, and P2T2. Their frequencies are denoted by
x1[1] = frequency of P1T1 in deme 1
x1[2] = frequency of P1T2 in deme 1
x1[3] = frequency of P2T1 in deme 1
x1[4] = frequency of P2T2 in deme 1
x2[1] = frequency of P1T1 in deme 2
x2[2] = frequency of P1T2 in deme 2
x2[3] = frequency of P2T1 in deme 2
x2[4] = frequency of P2T2 in deme 2

We use mainly allele frequencies and linkage disequilibria to describe the dynamics:

p12 = x1[3]+x1[4] = frequency of preference allele P2 in deme 1 (Note: the ordering of subscripts differs from that in the manuscript)
t12 = x1[2]+x1[4] = frequency of trait allele T2 in deme 1 (Note: the ordering of subscripts differs from that in the manuscript)
dd1 = x1[1]x1[4]-x1[2]x1[3] = linkage disequilibrium between P and T in deme 1
p22, t22, dd2 are the analogous quantities in deme 2

The phenotype-matching model is obtained as a special case if *r* = 0 and p12 = t12 and p22 = t22. As a consequence, linkage disequilibrium is maximal, i.e., dd1 = t12(1-t12) and dd2 = t22(1-t22).

1.1 Modeling the stages of the life cycle

1.1.1 Migration

Gamete frequencies after migration

```
x1m[1] = (1 - m1) x1[1] + m1 x2[1];
x1m[2] = (1 - m1) x1[2] + m1 x2[2];
x1m[3] = (1 - m1) x1[3] + m1 x2[3];
x1m[4] = (1 - m1) x1[4] + m1 x2[4];
```

```
x2m[1] = (1 - m2) x2[1] + m2 x1[1];
x2m[2] = (1 - m2) x2[2] + m2 x1[2];
x2m[3] = (1 - m2) x2[3] + m2 x1[3];
x2m[4] = (1 - m2) x2[4] + m2 x1[4];
```

1.1.2 Viability selection

Gamete frequencies after viability selection

pop 1

```
w1bar = 1 + s1 (x1m[1] + x1m[3]);
```

```
x1v[1] = x1m[1] (1 + s1) / w1bar;
x1v[2] = x1m[2] / w1bar;
x1v[3] = x1m[3] (1 + s1) / w1bar;
x1v[4] = x1m[4] / w1bar;
```

pop 2

```
w2bar = 1 + s2 (x2m[2] + x2m[4]);
```

```
x2v[1] = x2m[1] / w2bar;
x2v[2] = (1 + s2) x2m[2] / w2bar;
x2v[3] = x2m[3] / w2bar;
x2v[4] = (1 + s2) x2m[4] / w2bar;
```

1.1.3 Sexual selection

P1 prefers T1 (by a factor of 1+α1), and P2 prefers T2 (by a factor of 1+α2)

pop 1

```
Mate1 = Table[x1v[i] x1v[j], {i, 4}, {j, 4}];
Coef = Table[1, {i, 4}, {j, 4}];
Do[Coef[[i, 1]] = (1 + α1), {i, 1, 2}];
Do[Coef[[i, 3]] = (1 + α1), {i, 1, 2}];
Do[Coef[[i, 2]] = (1 + α2), {i, 3, 4}];
Do[Coef[[i, 4]] = (1 + α2), {i, 3, 4}];
G1 = Mate1 Coef;
```

```
Simplify[Coef] // TableForm
```

1 + α1	1	1 + α1	1
1 + α1	1	1 + α1	1
1	1 + α2	1	1 + α2
1	1 + α2	1	1 + α2

```
Do[z1[i] = Sum[x1v[j] Coef[[i, j]], {j, 4}], {i, 4}]
```

```
F1 = Table[0, {i, 4}, {j, 4}];
Do[F1[[i, j]] = G1[[i, j]] / z1[i], {i, 4}, {j, 4}];
```

```
TableForm[Simplify[F1]]
```

```
− ─────────────────────────────────────────────────────────────────────────────────────────────────────────
  (-1+s1 ((-1+m1) x1[1]+(-1+m1) x1[3]-m1 (x2[1]+x2[3]))) (-(-1+m1) (1+s1) (1+α1) x1[1]+x1[2]-m1 x1[2]+x1[3]-m1 x1[3]+s1 x1[3
                                                                                                                        (:
− ─────────────────────────────────────────────────────────────────────────────────────────────────────────
  (-1+s1 ((-1+m1) x1[1]+(-1+m1) x1[3]-m1 (x2[1]+x2[3]))) (-(-1+m1) (1+s1) (1+α1) x1[1]+x1[2]-m1 x1[2]+x1[3]-m1 x1[3]+s1 x1[3
                                                                                                      (1+s1)^2 ((-1+m1) x1[1]-m1
− ─────────────────────────────────────────────────────────────────────────────────────────────────────────
  (-1+s1 ((-1+m1) x1[1]+(-1+m1) x1[3]-m1 (x2[1]+x2[3]))) (-(-1+m1) (1+s1) x1[1]-(-1+m1) (1+α2) x1[2]+x1[3]-m1 x1[3]+s1 x1[3]
                                                                                                      (1+s1) ((-1+m1) x1[1]-m1 :
− ─────────────────────────────────────────────────────────────────────────────────────────────────────────
  (-1+s1 ((-1+m1) x1[1]+(-1+m1) x1[3]-m1 (x2[1]+x2[3]))) (-(-1+m1) (1+s1) x1[1]-(-1+m1) (1+α2) x1[2]+x1[3]-m1 x1[3]+s1 x1[3]
```

pop 2

```
Mate2 = Table[x2v[i] x2v[j], {i, 4}, {j, 4}];
G2 = Mate2 Coef;
```

```
Do[z2[i] = Sum[x2v[j] Coef[[i, j]], {j, 4}], {i, 4}]
```

```
F2 = Table[0, {i, 4}, {j, 4}];
Do[F2[[i, j]] = G2[[i, j]] / z2[i], {i, 4}, {j, 4}];
```

```
TableForm[F2]
```

```
                (1+α1) xnsf2[1] xnsm2[1]                                   xnsf2[1] xnsm2[2]                                 (1+α1
───────────────────────────────────────────────────────    ───────────────────────────────────────────────────────    ────────────
(1+α1) xnsm2[1]+xnsm2[2]+(1+α1) xnsm2[3]+xnsm2[4]          (1+α1) xnsm2[1]+xnsm2[2]+(1+α1) xnsm2[3]+xnsm2[4]          (1+α1) xnsm2[1]+xn
                (1+α1) xnsf2[2] xnsm2[1]                                   xnsf2[2] xnsm2[2]                                 (1+α1
───────────────────────────────────────────────────────    ───────────────────────────────────────────────────────    ────────────
(1+α1) xnsm2[1]+xnsm2[2]+(1+α1) xnsm2[3]+xnsm2[4]          (1+α1) xnsm2[1]+xnsm2[2]+(1+α1) xnsm2[3]+xnsm2[4]          (1+α1) xnsm2[1]+xn
                   xnsf2[3] xnsm2[1]                                 (1+α2) xnsf2[3] xnsm2[2]                                  xn
───────────────────────────────────────────────────────    ───────────────────────────────────────────────────────    ────────────
xnsm2[1]+(1+α2) xnsm2[2]+xnsm2[3]+(1+α2) xnsm2[4]          xnsm2[1]+(1+α2) xnsm2[2]+xnsm2[3]+(1+α2) xnsm2[4]          xnsm2[1]+(1+α2) xn
                   xnsf2[4] xnsm2[1]                                 (1+α2) xnsf2[4] xnsm2[2]                                  xn
───────────────────────────────────────────────────────    ───────────────────────────────────────────────────────    ────────────
xnsm2[1]+(1+α2) xnsm2[2]+xnsm2[3]+(1+α2) xnsm2[4]          xnsm2[1]+(1+α2) xnsm2[2]+xnsm2[3]+(1+α2) xnsm2[4]          xnsm2[1]+(1+α2) xn
```

1.1.4 Recombination

pop 1

```
x1t1[1] = Simplify[F1[[1, 1]] + (1/2) F1[[1, 2]] + (1/2) F1[[1, 3]] +
    (1/2) (1 - r) F1[[1, 4]] + (1/2) F1[[2, 1]] + (1/2) r F1[[2, 3]] +
    (1/2) F1[[3, 1]] + (1/2) r F1[[3, 2]] + (1/2) (1 - r) F1[[4, 1]]];

x1t1[2] = Simplify[(1/2) F1[[1,2]] + (1/2) r F1[[1,4]] +
    (1/2) F1[[2,1]] + F1[[2,2]] + (1/2)(1-r)F1[[2,3]] +
    (1/2) F1[[2,4]] + (1/2) (1-r) F1[[3,2]] +
    (1/2) r F1[[4,1]] + (1/2) F1[[4,2]]];

x1t1[3] = Simplify[(1/2) F1[[1,3]] + (1/2) r F1[[1,4]] +
    (1/2) (1-r) F1[[2,3]] + (1/2) F1[[3,1]] +
    (1/2) (1-r) F1[[3,2]] + F1[[3,3]] + (1/2) F1[[3,4]] +
    (1/2) r F1[[4,1]] + (1/2) F1[[4,3]]];

x1t1[4] = Simplify[(1/2) (1-r) F1[[1,4]] + (1/2) r F1[[2,3]] +
    (1/2) F1[[2,4]] + (1/2) r F1[[3,2]] + (1/2) F1[[3,4]] +
    (1/2) (1-r) F1[[4,1]] + (1/2) F1[[4,2]] + (1/2) F1[[4,3]] + F1[[4,4]]];
```

pop 2

```
x2t1[1] = Simplify[F2[[1, 1]] + (1/2) F2[[1, 2]] + (1/2) F2[[1, 3]] +
    (1/2) (1 - r) F2[[1, 4]] + (1/2) F2[[2, 1]] + (1/2) r F2[[2, 3]] +
    (1/2) F2[[3, 1]] + (1/2) r F2[[3, 2]] + (1/2) (1 - r) F2[[4, 1]]];

x2t1[2] = Simplify[(1/2) F2[[1,2]] + (1/2) r F2[[1,4]] +
    (1/2) F2[[2,1]] + F2[[2,2]] + (1/2)(1-r)F2[[2,3]] +
    (1/2) F2[[2,4]] + (1/2) (1-r) F2[[3,2]] +
    (1/2) r F2[[4,1]] + (1/2) F2[[4,2]]];

x2t1[3] = Simplify[(1/2) F2[[1,3]] + (1/2) r F2[[1,4]] +
    (1/2) (1-r) F2[[2,3]] + (1/2) F2[[3,1]] +
    (1/2) (1-r) F2[[3,2]] + F2[[3,3]] + (1/2) F2[[3,4]] +
    (1/2) r F2[[4,1]] + (1/2) F2[[4,3]]];

x2t1[4] = Simplify[(1/2) (1-r) F2[[1,4]] + (1/2) r F2[[2,3]] +
    (1/2) F2[[2,4]] + (1/2) r F2[[3,2]] + (1/2) F2[[3,4]] +
    (1/2) (1-r) F2[[4,1]] + (1/2) F2[[4,2]] + (1/2) F2[[4,3]] + F2[[4,4]]];
```

1.2 Recursion relation for allele frequencies and LD

The notation here is that the 1st subscript represents the population and the second represents the allele.
So all is done with respect to allele 2 in both populations.

1.2.1 Derivation of the recursion relations

```
x1[1] = (1 - p12) (1 - t12) + dd1;
x1[2] = (1 - p12) t12 - dd1;
x1[3] = p12 (1 - t12) - dd1;
x1[4] = p12 t12 + dd1;
x2[1] = (1 - p22) (1 - t22) + dd2;
x2[2] = (1 - p22) t22 - dd2;
x2[3] = p22 (1 - t22) - dd2;
x2[4] = p22 t22 + dd2;

p1t1 = Simplify[x1t1[3] + x1t1[4]]

t1t1 = Simplify[x1t1[2] + x1t1[4]]
```

```
dd1t1 = x1t1[1] x1t1[4] - x1t1[2] x1t1[3]
```

```
p2t1 = Simplify[x2t1[3] + x2t1[4]]
```

```
t2t1 = Simplify[x2t1[2] + x2t1[4]]
```

```
dd2t1 = x2t1[1] x2t1[4] - x2t1[2] x2t1[3]
```

1.2.2 The main recursion relation, itgen[s1, s2, α1, α2, m1, m2, r][{p12, t12, dd1, p22, t22, dd2}]

For given parameters {s1, s2, α1, α2, m1, m2, r}, itgen computes {p12 (t + 1), t12 (t + 1), dd1 (t + 1), p22 (t + 1), t22 (t + 1), dd2 (t + 1)} from {p12 (t), t12 (t), dd1 (t), p22 (t), t22 (t), dd2 (t)} .
Because computation of p1t1 etc (see above) is time consuming, we define itgen[s1, s2, α1, α2, m1, m2, r][{p12, t12, dd1, p22, t22, dd2}] explicitly by the right-hand side of {p1t1, t1t1, dd1t1, p2t1, t2t1, dd2t1}.

```
itgen[s1_, s2_, α1_, α2_, m1_, m2_, r_][{p12_, t12_, dd1_, p22_, t22_, dd2_}] :=
  {(- (-1 + m1)^2 m1 p12^2 (1 + s1) (-1 + s1 (-1 + t12)) (t12 - t22) (α1 + α2 + α1 α2) -
      dd1 (α1 + 2 s1^3 (-1 + t12)^2 (1 + α1) + t12 α1 α2 +
          s1 (2 + dd1 α2 + 2 t12 α2 + α1 (4 + dd1 - 3 t12 + dd1 α2 + 3 t12 α2 - 2 t12^2 α2)) +
          s1^2 (4 + dd1 α2 - 2 t12^2 (α1 (-1 + α2) + α2) +
              α1 (5 + dd1 + dd1 α2) + t12 (-4 - 7 α1 + 2 α2 + 2 α1 α2))) -
      m1^3 (t12 - t22) (-2 (dd1 - dd2) s1 (t12 - t22) (s1 + α1 + s1 α1) (s1 - α2) +
          p22^2 (1 + s1) (-1 + s1 (-1 + t22)) (α1 + α2 + α1 α2) +
          p22 (2 s1^3 (t12 - t22) (-1 + t22) (1 + α1) + (t12 - t22) α1 α2 +
              s1 (-dd1 (α1 + α2 + α1 α2) + dd2 (α1 + α2 + α1 α2) - (t12 - t22)
                    (α1 - 2 α2 - 3 α1 α2 + 2 t22 α1 α2)) + s1^2 (t22 (2 + α1 (3 - 2 α2) - 2 α2) + 2
                  t22^2 (α1 (-1 + α2) + α2) - (dd1 - dd2) (α1 + α2 + α1 α2) + t12
                    (-2 + 2 α2 - 2 t22 α2 + α1 (-3 + 2 t22 + 2 α2 - 2 t22 α2))))) +
      m1 (-dd2 (-α1 + 2 s1^2 (-1 + t12) (1 + α1) + s1 (-2 + (-3 + 2 t12) α1))
            (-1 + s1 (-1 + t12) - t12 α2) + 2 dd1^2 s1 (1 + s1) (α1 + α2 + α1 α2) +
          dd1 (2 s1^3 (-1 + t12) (-1 + 3 t12 - 2 t22) (1 + α1) + α1 (1 + 2 t12 α2 - t22 α2) +
              s1^2 (4 + 5 α1 - 2 dd2 α1 - 2 dd2 α2 - 2 dd2 α1 α2 - 6 t12^2
                    (α1 (-1 + α2) + α2) + t22 (4 + 7 α1 - 2 α2 - 2 α1 α2) + 2 t12
                    (α1 (-7 + 2 t22 (-1 + α2) + 2 α2) + 2 (-2 + α2 + t22 α2))) +
              s1 (2 - 2 dd2 α2 + 4 t12 α2 - 2 t22 α2 + α1 (4 + 3 t22 - 6 t12^2 α2 -
                      3 t22 α2 - 2 dd2 (1 + α2) + t12 (-6 + 6 α2 + 4 t22 α2)))) +
          p22 (2 - 2 s1^3 (-1 + t12)^2 (-1 + t22) (1 + α1) + dd1 α2 + 2 t12 α2 +
              α1 (dd1 (1 + α2) - (-2 + t12 + t22) (1 + t12 α2)) + s1^2 (6 + 6 α1 + dd1 α1 + dd1
                    α2 + dd1 α1 α2 + t12^2 (2 + α1 (3 + 2 t22 (-1 + α2) - 2 α2) + 2 (-1 + t22) α2) +
                  t12 (-8 + t22 (4 + α1 (7 - 2 α2) - 2 α2) + 2 α2 + dd1 α2 + α1 (-9 + dd1 +
                          2 α2 + dd1 α2)) - t22 (4 + 2 dd1 α2 + α1 (5 + 2 dd1 (1 + α2)))) +
              s1 (t12^2 (α1 - 2 α2 - 3 α1 α2 + 2 t22 α1 α2) + t12 (-4 + (4 + dd1 - 2 t22) α2 +
                      α1 (-6 + dd1 + 3 t22 + 4 α2 + dd1 α2 - 3 t22 α2)) - 2 (-3 - dd1 α2 -
                      α1 (3 + dd1 + dd1 α2) + t22 (1 + dd1 α2 + α1 (2 + dd1 + dd1 α2)))))) +
      m1^2 (-dd1^2 s1 (1 + s1) (α1 + α2 + α1 α2) + p22^2 (1 + s1) (-1 + s1 (-1 + t22))
            (t12 - t22) (α1 + α2 + α1 α2) -
          dd2 (-4 s1^3 (-1 + t12) (t12 - t22) (1 + α1) + (-t12 + t22) α1 α2 +
```

```
s1 (dd2 (α1 + α2 + α1 α2) + (t12 - t22) (-2 α2 + α1 (3 - 3 α2 + 4 t12 α2))) + s1^2
  (4 t12^2 (α1 (-1 + α2) + α2) + dd2 (α1 + α2 + α1 α2) + t22 (-4 - 7 α1 + 2 α2 + 2 α1
      α2) + t12 (α1 (7 + 4 t22 - 2 α2 - 4 t22 α2) - 2 (-2 + α2 + 2 t22 α2)))) +
dd1 (-2 s1^3 (3 t12^2 + t22 (2 + t22) - 2 t12 (1 + 2 t22)) (1 + α1) +
  (-t12 + t22) α1 α2 + s1 (2 dd2 (α1 + α2 + α1 α2) + (t12 - t22)
     (-2 α2 + α1 (3 + (-3 + 6 t12 - 2 t22) α2))) +
  s1^2 (6 t12^2 (α1 (-1 + α2) + α2) + 2 t22^2 (α1 (-1 + α2) + α2) + 2 dd2
     (α1 + α2 + α1 α2) + t22 (-4 - 7 α1 + 2 α2 + 2 α1 α2) + t12
     (α1 (7 + 8 t22 - 2 α2 - 8 t22 α2) - 2 (-2 + α2 + 4 t22 α2)))) +
p22 (-dd1 α1 + dd2 α1 + t12 α1 - t22 α1 + 4 s1^3 (-1 + t12) (t12 - t22)
  (-1 + t22) (1 + α1) - dd1 α2 + dd2 α2 - 2 t12 α2 + 2 t22 α2 - dd1 α1 α2 +
  dd2 α1 α2 - 2 t12 α1 α2 + 2 t12^2 α1 α2 + 2 t22 α1 α2 - t12 t22 α1 α2 -
  t22^2 α1 α2 + s1^2 (t22^2 (4 + α1 (7 - 2 α2) - 2 α2) - (dd1 - dd2) (α1 + α2 + α1 α2) +
    t12^2 (-4 - 4 (-1 + t22) α2 + α1 (-6 - 4 t22 (-1 + α2) + 4 α2)) + t12
     (8 + (-2 - 2 dd1 + dd2 - 2 t22 + 4 t22^2) α2 + α1 (9 + dd2 - t22 - 4 t22^2 - 2 α2 +
        dd2 α2 - 2 t22 α2 + 4 t22^2 α2 - 2 dd1 (1 + α2))) + t22 (-8 + 2 α2 +
      3 dd1 α2 - 2 dd2 α2 + α1 (-9 + 2 α2 + 3 dd1 (1 + α2) - 2 dd2 (1 + α2)))) +
  s1 (t22^2 (-3 α1 (-1 + α2) - 2 α2) - 2 (dd1 - dd2) (α1 + α2 + α1 α2) - 2
     t12^2 (α1 - 2 α2 - 3 α1 α2 + 2 t22 α1 α2) + t12
     (4 + (-4 - 2 dd1 + dd2 - 2 t22) α2 + α1 (6 + dd2 - t22 - 4 α2 + dd2 α2 -
        3 t22 α2 + 4 t22^2 α2 - 2 dd1 (1 + α2))) + t22 (-4 + (4 + 3 dd1 - 2 dd2)
      α2 + α1 (3 dd1 (1 + α2) - 2 (3 + dd2 - 2 α2 + dd2 α2)))))) +
(-1 + m1) p12 (-2 - 2 α1 - dd1 α1 + dd1 m1 α1 - dd2 m1 α1 + 2 t12 α1 -
  m1 t12 α1 + m1 p22 t12 α1 - 2 m1^2 p22 t12 α1 +
  m1 t22 α1 - m1 p22 t22 α1 + 2 m1^2 p22 t22 α1 +
  2 s1^3 (-1 + t12) (-1 + t12 - m1 t12 + m1 t22)^2 (1 + α1) -
  dd1 α2 + dd1 m1 α2 - dd2 m1 α2 - 2 t12 α2 + 2 m1 t12 α2 +
  m1 p22 t12 α2 - 2 m1^2 p22 t12 α2 - 2 m1 t22 α2 - m1 p22 t22 α2 +
  2 m1^2 p22 t22 α2 - dd1 α1 α2 + dd1 m1 α1 α2 - dd2 m1 α1 α2 -
  2 t12 α1 α2 + 2 m1 t12 α1 α2 + m1 p22 t12 α1 α2 -
  2 m1^2 p22 t12 α1 α2 + 2 t12^2 α1 α2 - 3 m1 t12^2 α1 α2 + m1^2 t12^2 α1 α2 -
  2 m1 t22 α1 α2 - m1 p22 t22 α1 α2 + 2 m1^2 p22 t22 α1 α2 +
  3 m1 t12 t22 α1 α2 - 2 m1^2 t12 t22 α1 α2 + m1^2 t22^2 α1 α2 +
  s1 (-2 (-1 + m1)^2 t12^3 α1 α2 - 2 (3 + dd1 α2 + α1 (3 + dd1 + dd1 α2)) - m1^2 t22
     (-4 p22 α1 + t22 α1 + p22 t22 α1 - 4 p22 α2 - 2 t22 α2 + p22 t22 α2 - 4 p22 α1 α2 -
      3 t22 α1 α2 + p22 t22 α1 α2 - dd1 (α1 + α2 + α1 α2) + dd2 (α1 + α2 + α1 α2)) -
    m1 (-2 (dd1 - dd2) (α1 + α2 + α1 α2) + t22 (-4 + (4 + dd1 + 2 p22) α2 + α1
          (-6 + dd1 + 2 p22 + 4 α2 + dd1 α2 + 2 p22 α2))) + (-1 + m1) t12^2 ((-4 + m1
          (2 + p22)) α2 + α1 (4 - 6 α2 + m1 (-1 + p22 + 3 α2 + p22 α2 + 4 t22 α2))) +
    t12 (6 + 10 α1 + dd1 α1 - 4 α2 + dd1 α2 - 4 α1 α2 + dd1 α1 α2 - m1^2
       (4 p22 α1 - 2 t22 α1 + 4 p22 α2 + 4 t22 α2 + 4 p22 α1 α2 + 6 t22 α1 α2 +
        2 t22^2 α1 α2 + dd1 (α1 + α2 + α1 α2) - dd2 (α1 + α2 + α1 α2)) + m1
       (-4 + 4 α2 + dd2 α2 + 2 p22 α2 + 6 t22 α2 + p22 t22 α2 + α1
          (-6 - 5 t22 + 4 α2 + 9 t22 α2 + dd2 (1 + α2) + p22 (2 + t22) (1 + α2))))) -
  s1^2 (6 + 6 α1 + dd1 α1 + dd1 α2 + dd1 α1 α2 + 2 (-1 + m1)^2 t12^3 (α1 (-1 + α2) + α2) +
    m1^2 t22 (-(dd1 - dd2 + 2 p22) (α1 + α2 + α1 α2) + t22 (2 + (-2 + p22) α2 +
          α1 (3 + p22 - 2 α2 + p22 α2))) + m1 (-(dd1 - dd2) (α1 + α2 + α1 α2) + t22
       (-8 + (2 + dd1 + p22) α2 + α1 (-9 + dd1 + p22 + 2 α2 + dd1 α2 + p22 α2))) -
    (-1 + m1) t12^2 (6 + 10 α1 - 4 α2 - 4 α1 α2 + m1 (-2 + (2 + p22 + 4 t22) α2 +
          α1 (-3 + p22 - 4 t22 + 2 α2 + p22 α2 + 4 t22 α2))) + t12 (-12 - 14 α1 - dd1
```

$$
\begin{aligned}
&\alpha1 + 2\,\alpha2 - dd1\,\alpha2 + 2\,\alpha1\,\alpha2 - dd1\,\alpha1\,\alpha2 + m1^2\,(2\,t22^2\,(\alpha1\,(-1 + \alpha2) + \alpha2) + \\
&(dd1 - dd2 + 2\,p22)\,(\alpha1 + \alpha2 + \alpha1\,\alpha2) + t22\,(-4 - 6\,\alpha1 + 4\,\alpha2 + 4\,\alpha1\,\alpha2)) - \\
&m1\,(-8 + 2\,\alpha2 + dd2\,\alpha2 + p22\,\alpha2 + \alpha1\,(-9 + dd2 + p22 + 2\,\alpha2 + dd2\,\alpha2 + p22\,\alpha2) + \\
&t22\,(-8 + (6 + p22)\,\alpha2 + \alpha1\,(-13 + p22 + 6\,\alpha2 + p22\,\alpha2))))))) / \\
&(2\,(1 + s1\,(1 + (-1 + m1)\,t12 - m1\,t22))\,(1 + (1 + (-1 + m1)\,t12 - m1\,t22)\,\alpha1 + \\
&s1\,(1 + (-1 + m1)\,t12 - m1\,t22)\,(1 + \alpha1)) \\
&(1 + s1\,(1 + (-1 + m1)\,t12 - m1\,t22) + m1\,t22\,\alpha2 + t12\,(\alpha2 - m1\,\alpha2))), \\
&-((((-1 + m1)\,t12 - m1\,t22)\,(2 + p12\,\alpha2 - m1\,p12\,\alpha2 + m1\,p22\,\alpha2 + 2\,t12\,\alpha2 - \\
&2\,m1\,t12\,\alpha2 - p12\,t12\,\alpha2 + 2\,m1\,p12\,t12\,\alpha2 - m1^2\,p12\,t12\,\alpha2 - \\
&m1\,p22\,t12\,\alpha2 + m1^2\,p22\,t12\,\alpha2 + 2\,m1\,t22\,\alpha2 - m1\,p12\,t22\,\alpha2 + \\
&m1^2\,p12\,t22\,\alpha2 - m1^2\,p22\,t22\,\alpha2 - (1 + (-1 + m1)\,t12 - m1\,t22)\,\alpha1 \\
&(-1 - t12\,\alpha2 + (-1 + m1)\,p12\,(1 + \alpha2) - m1\,(p22 + p22\,\alpha2 - t12\,\alpha2 + t22\,\alpha2)) + \\
&s1\,(1 + (-1 + m1)\,t12 - m1\,t22)\,(4 - dd1\,\alpha2 + dd1\,m1\,\alpha2 - dd2\,m1\,\alpha2 + 2\,p12\,\alpha2 - \\
&2\,m1\,p12\,\alpha2 + 2\,m1\,p22\,\alpha2 + 2\,t12\,\alpha2 - 2\,m1\,t12\,\alpha2 - p12\,t12\,\alpha2 + m1\,p12\,t12\,\alpha2 + \\
&2\,m1\,t22\,\alpha2 - m1\,p22\,t22\,\alpha2 + \alpha1\,(2 + 2\,p12 - 2\,m1\,p12 + 2\,m1\,p22 - t12 + m1\,t12 - \\
&p12\,t12 + m1\,p12\,t12 - m1\,t22 - m1\,p22\,t22 + 2\,p12\,\alpha2 - 2\,m1\,p12\,\alpha2 + \\
&2\,m1\,p22\,\alpha2 + t12\,\alpha2 - m1\,t12\,\alpha2 - p12\,t12\,\alpha2 + m1\,p12\,t12\,\alpha2 + \\
&m1\,t22\,\alpha2 - m1\,p22\,t22\,\alpha2 + dd1\,(-1 + m1)\,(1 + \alpha2) - dd2\,m1\,(1 + \alpha2))) + \\
&s1^2\,(1 + (-1 + m1)\,t12 - m1\,t22)\,(2 + \alpha1 - dd1\,\alpha1 + p12\,\alpha1 - dd1\,\alpha2 + p12\,\alpha2 - \\
&dd1\,\alpha1\,\alpha2 + p12\,\alpha1\,\alpha2 + (-1 + m1)\,t12\,(2 + p12\,\alpha2 + \alpha1\,(1 + p12 + p12\,\alpha2)) - \\
&m1\,(-(dd1 - dd2 - p12 + p22)\,(\alpha1 + \alpha2 + \alpha1\,\alpha2) + \\
&t22\,(2 + p22\,\alpha2 + \alpha1\,(1 + p22 + p22\,\alpha2)))))) / \\
&(2\,(1 + s1\,(1 + (-1 + m1)\,t12 - m1\,t22))\,(1 + (1 + (-1 + m1)\,t12 - m1\,t22)\,\alpha1 + \\
&s1\,(1 + (-1 + m1)\,t12 - m1\,t22)\,(1 + \alpha1)) \\
&(1 + s1\,(1 + (-1 + m1)\,t12 - m1\,t22) + m1\,t22\,\alpha2 + t12\,(\alpha2 - m1\,\alpha2)))), \\
&-(((1 + s1)\,(-1 + s1\,((-1 + m1)\,(dd1 + (1 - p12)\,(1 - t12)) + (-1 + m1) \\
&(-dd1 + p12\,(1 - t12)) - m1\,((1 - p22)\,(1 - t22) + p22\,(1 - t22))))^2 \\
&(-((r\,((-1 + m1)\,(dd1 + (1 - p12)\,(1 - t12)) - m1\,(dd2 + (1 - p22)\,(1 - t22))) \\
&((-1 + m1)\,(dd1 + p12\,t12) - m1\,(dd2 + p22\,t22))) / (-dd1 - m1\,(-dd1 + p12 \\
&(1 - t12)) + s1\,(-dd1 + p12\,(1 - t12)) - m1\,s1\,(-dd1 + p12\,(1 - t12)) + \\
&p12\,(1 - t12) + (1 - p12)\,t12 + p12\,t12 - m1\,(-dd1 + (1 - p12)\,t12) - \\
&m1\,(dd1 + p12\,t12) + m1\,(dd2 + (1 - p22)\,(1 - t22)) + \\
&m1\,s1\,(dd2 + (1 - p22)\,(1 - t22)) + m1\,(-dd2 + p22\,(1 - t22)) + m1\,s1 \\
&(-dd2 + p22\,(1 - t22)) + m1\,(-dd2 + (1 - p22)\,t22) + m1\,(dd2 + p22\,t22) + \\
&(-dd1 + p12\,(1 - t12))\,\alpha1 - m1\,(-dd1 + p12\,(1 - t12))\,\alpha1 + \\
&s1\,(-dd1 + p12\,(1 - t12))\,\alpha1 - m1\,s1\,(-dd1 + p12\,(1 - t12))\,\alpha1 + \\
&m1\,(dd2 + (1 - p22)\,(1 - t22))\,\alpha1 + m1\,s1\,(dd2 + (1 - p22)\,(1 - t22))\,\alpha1 + \\
&m1\,(-dd2 + p22\,(1 - t22))\,\alpha1 + m1\,s1\,(-dd2 + p22\,(1 - t22))\,\alpha1 - \\
&(-1 + m1)\,(1 + s1)\,(dd1 + (1 - p12)\,(1 - t12))\,(1 + \alpha1))) - \\
&((1 + s1)\,((-1 + m1)\,(dd1 + (1 - p12)\,(1 - t12)) - m1\,(dd2 + (1 - p22)\,(1 - t22))) \\
&((-1 + m1)\,(-dd1 + p12\,(1 - t12)) - m1\,(-dd2 + p22\,(1 - t22)))\,(1 + \alpha1)) / \\
&(-dd1 - m1\,(-dd1 + p12\,(1 - t12)) + s1\,(-dd1 + p12\,(1 - t12)) - \\
&m1\,s1\,(-dd1 + p12\,(1 - t12)) + p12\,(1 - t12) + (1 - p12)\,t12 + p12\,t12 - m1 \\
&(-dd1 + (1 - p12)\,t12) - m1\,(dd1 + p12\,t12) + m1\,(dd2 + (1 - p22)\,(1 - t22)) + \\
&m1\,s1\,(dd2 + (1 - p22)\,(1 - t22)) + m1\,(-dd2 + p22\,(1 - t22)) + \\
&m1\,s1\,(-dd2 + p22\,(1 - t22)) + m1\,(-dd2 + (1 - p22)\,t22) + m1\,(dd2 + p22\,t22) + \\
&(-dd1 + p12\,(1 - t12))\,\alpha1 - m1\,(-dd1 + p12\,(1 - t12))\,\alpha1 + \\
&s1\,(-dd1 + p12\,(1 - t12))\,\alpha1 - m1\,s1\,(-dd1 + p12\,(1 - t12))\,\alpha1 + \\
&m1\,(dd2 + (1 - p22)\,(1 - t22))\,\alpha1 + m1\,s1\,(dd2 + (1 - p22)\,(1 - t22))\,\alpha1 + \\
&m1\,(-dd2 + p22\,(1 - t22))\,\alpha1 + m1\,s1\,(-dd2 + p22\,(1 - t22))\,\alpha1 -
\end{aligned}
$$

$(-1+m1)\ (1+s1)\ (dd1+(1-p12)\ (1-t12))\ (1+\alpha1))+$
$((-1+r)\ ((-1+m1)\ (-dd1+p12\ (1-t12))-m1\ (-dd2+p22\ (1-t22))))$
$((-1+m1)\ (-dd1+(1-p12)\ t12)-m1\ (-dd2+(1-p22)\ t22))\ (1+\alpha1))\ /$
$(-dd1-m1\ (-dd1+p12\ (1-t12))+s1\ (-dd1+p12\ (1-t12))-$
$m1\ s1\ (-dd1+p12\ (1-t12))+p12\ (1-t12)+(1-p12)\ t12+p12\ t12-m1$
$(-dd1+(1-p12)\ t12)-m1\ (dd1+p12\ t12)+m1\ (dd2+(1-p22)\ (1-t22))+$
$m1\ s1\ (dd2+(1-p22)\ (1-t22))+m1\ (-dd2+p22\ (1-t22))+$
$m1\ s1\ (-dd2+p22\ (1-t22))+m1\ (-dd2+(1-p22)\ t22)+m1\ (dd2+p22\ t22)+$
$(-dd1+p12\ (1-t12))\ \alpha1-m1\ (-dd1+p12\ (1-t12))\ \alpha1+$
$s1\ (-dd1+p12\ (1-t12))\ \alpha1-m1\ s1\ (-dd1+p12\ (1-t12))\ \alpha1+$
$m1\ (dd2+(1-p22)\ (1-t22))\ \alpha1+m1\ s1\ (dd2+(1-p22)\ (1-t22))\ \alpha1+$
$m1\ (-dd2+p22\ (1-t22))\ \alpha1+m1\ s1\ (-dd2+p22\ (1-t22))\ \alpha1-$
$(-1+m1)\ (1+s1)\ (dd1+(1-p12)\ (1-t12))\ (1+\alpha1))-$
$((1+s1)\ ((-1+m1)\ (dd1+(1-p12)\ (1-t12))-m1\ (dd2+(1-p22)\ (1-t22)))$
$((-1+m1)\ (-dd1+p12\ (1-t12))-m1\ (-dd2+p22\ (1-t22))))\ /$
$(-(-1+m1)\ (1+s1)\ (dd1+(1-p12)\ (1-t12))-m1\ (-dd1+p12\ (1-t12))+$
$s1\ (-dd1+p12\ (1-t12))-m1\ s1\ (-dd1+p12\ (1-t12))+p12\ (1-t12)+$
$p12\ t12-m1\ (dd1+p12\ t12)+m1\ (dd2+(1-p22)\ (1-t22))+$
$m1\ s1\ (dd2+(1-p22)\ (1-t22))+m1\ (-dd2+p22\ (1-t22))+$
$m1\ s1\ (-dd2+p22\ (1-t22))+m1\ (-dd2+(1-p22)\ t22)+m1\ (dd2+p22\ t22)+$
$(dd1+p12\ t12)\ \alpha2-m1\ (dd1+p12\ t12)\ \alpha2+m1\ (-dd2+(1-p22)\ t22)\ \alpha2+$
$m1\ (dd2+p22\ t22)\ \alpha2-(-1+m1)\ (-dd1+(1-p12)\ t12)\ (1+\alpha2))-$
$(2\ (1+s1)\ ((-1+m1)\ (-dd1+p12\ (1-t12))-m1\ (-dd2+p22\ (1-t22)))^2)\ /$
$(-(-1+m1)\ (1+s1)\ (dd1+(1-p12)\ (1-t12))-m1\ (-dd1+p12\ (1-t12))+$
$s1\ (-dd1+p12\ (1-t12))-m1\ s1\ (-dd1+p12\ (1-t12))+p12\ (1-t12)+$
$p12\ t12-m1\ (dd1+p12\ t12)+m1\ (dd2+(1-p22)\ (1-t22))+$
$m1\ s1\ (dd2+(1-p22)\ (1-t22))+m1\ (-dd2+p22\ (1-t22))+$
$m1\ s1\ (-dd2+p22\ (1-t22))+m1\ (-dd2+(1-p22)\ t22)+m1\ (dd2+p22\ t22)+$
$(dd1+p12\ t12)\ \alpha2-m1\ (dd1+p12\ t12)\ \alpha2+m1\ (-dd2+(1-p22)\ t22)\ \alpha2+$
$m1\ (dd2+p22\ t22)\ \alpha2-(-1+m1)\ (-dd1+(1-p12)\ t12)\ (1+\alpha2))-$
$(r\ ((-1+m1)\ (dd1+(1-p12)\ (1-t12))-m1\ (dd2+(1-p22)\ (1-t22)))$
$((-1+m1)\ (dd1+p12\ t12)-m1\ (dd2+p22\ t22)))\ /$
$(-(-1+m1)\ (1+s1)\ (dd1+(1-p12)\ (1-t12))-m1\ (-dd1+p12\ (1-t12))+$
$s1\ (-dd1+p12\ (1-t12))-m1\ s1\ (-dd1+p12\ (1-t12))+p12\ (1-t12)+$
$p12\ t12-m1\ (dd1+p12\ t12)+m1\ (dd2+(1-p22)\ (1-t22))+$
$m1\ s1\ (dd2+(1-p22)\ (1-t22))+m1\ (-dd2+p22\ (1-t22))+$
$m1\ s1\ (-dd2+p22\ (1-t22))+m1\ (-dd2+(1-p22)\ t22)+m1\ (dd2+p22\ t22)+$
$(dd1+p12\ t12)\ \alpha2-m1\ (dd1+p12\ t12)\ \alpha2+m1\ (-dd2+(1-p22)\ t22)\ \alpha2+$
$m1\ (dd2+p22\ t22)\ \alpha2-(-1+m1)\ (-dd1+(1-p12)\ t12)\ (1+\alpha2))-$
$(((-1+m1)\ (-dd1+p12\ (1-t12))-m1\ (-dd2+p22\ (1-t22)))$
$((-1+m1)\ (dd1+p12\ t12)-m1\ (dd2+p22\ t22)))\ /$
$(-(-1+m1)\ (1+s1)\ (dd1+(1-p12)\ (1-t12))-m1\ (-dd1+p12\ (1-t12))+$
$s1\ (-dd1+p12\ (1-t12))-m1\ s1\ (-dd1+p12\ (1-t12))+p12\ (1-t12)+$
$p12\ t12-m1\ (dd1+p12\ t12)+m1\ (dd2+(1-p22)\ (1-t22))+$
$m1\ s1\ (dd2+(1-p22)\ (1-t22))+m1\ (-dd2+p22\ (1-t22))+$
$m1\ s1\ (-dd2+p22\ (1-t22))+m1\ (-dd2+(1-p22)\ t22)+m1\ (dd2+p22\ t22)+$
$(dd1+p12\ t12)\ \alpha2-m1\ (dd1+p12\ t12)\ \alpha2+m1\ (-dd2+(1-p22)\ t22)\ \alpha2+$
$m1\ (dd2+p22\ t22)\ \alpha2-(-1+m1)\ (-dd1+(1-p12)\ t12)\ (1+\alpha2))+$
$((-1+r)\ ((-1+m1)\ (-dd1+p12\ (1-t12))-m1\ (-dd2+p22\ (1-t22)))$
$((-1+m1)\ (-dd1+(1-p12)\ t12)-m1\ (-dd2+(1-p22)\ t22))\ (1+\alpha2))\ /$

$$
\begin{aligned}
&(-(-1+m1)\,(1+s1)\,(dd1+(1-p12)\,(1-t12))-m1\,(-dd1+p12\,(1-t12))+\\
&\quad s1\,(-dd1+p12\,(1-t12))-m1\,s1\,(-dd1+p12\,(1-t12))+p12\,(1-t12)+\\
&\quad p12\,t12-m1\,(dd1+p12\,t12)+m1\,(dd2+(1-p22)\,(1-t22))+\\
&\quad m1\,s1\,(dd2+(1-p22)\,(1-t22))+m1\,(-dd2+p22\,(1-t22))+\\
&\quad m1\,s1\,(-dd2+p22\,(1-t22))+m1\,(-dd2+(1-p22)\,t22)+m1\,(dd2+p22\,t22)+\\
&\quad (dd1+p12\,t12)\,\alpha2-m1\,(dd1+p12\,t12)\,\alpha2+m1\,(-dd2+(1-p22)\,t22)\,\alpha2+\\
&\quad m1\,(dd2+p22\,t22)\,\alpha2-(-1+m1)\,(-dd1+(1-p12)\,t12)\,(1+\alpha2))-\\
&(((-1+m1)\,(-dd1+p12\,(1-t12))-m1\,(-dd2+p22\,(1-t22)))\\
&\quad ((-1+m1)\,(dd1+p12\,t12)-m1\,(dd2+p22\,t22))\,(1+\alpha2))\,/\\
&(-(-1+m1)\,(1+s1)\,(dd1+(1-p12)\,(1-t12))-m1\,(-dd1+p12\,(1-t12))+\\
&\quad s1\,(-dd1+p12\,(1-t12))-m1\,s1\,(-dd1+p12\,(1-t12))+p12\,(1-t12)+\\
&\quad p12\,t12-m1\,(dd1+p12\,t12)+m1\,(dd2+(1-p22)\,(1-t22))+\\
&\quad m1\,s1\,(dd2+(1-p22)\,(1-t22))+m1\,(-dd2+p22\,(1-t22))+\\
&\quad m1\,s1\,(-dd2+p22\,(1-t22))+m1\,(-dd2+(1-p22)\,t22)+m1\,(dd2+p22\,t22)+\\
&\quad (dd1+p12\,t12)\,\alpha2-m1\,(dd1+p12\,t12)\,\alpha2+m1\,(-dd2+(1-p22)\,t22)\,\alpha2+\\
&\quad m1\,(dd2+p22\,t22)\,\alpha2-(-1+m1)\,(-dd1+(1-p12)\,t12)\,(1+\alpha2)))\\
&(-(((1+s1)\,((-1+m1)\,(dd1+(1-p12)\,(1-t12))-m1\,(dd2+\\
&\qquad (1-p22)\,(1-t22))))\,((-1+m1)\,(-dd1+(1-p12)\,t12)-\\
&\qquad m1\,(-dd2+(1-p22)\,t22)))\,/\,(-dd1-m1\,(-dd1+p12\,(1-t12))+\\
&\qquad s1\,(-dd1+p12\,(1-t12))-m1\,s1\,(-dd1+p12\,(1-t12))+p12\,(1-t12)+\\
&\qquad (1-p12)\,t12+p12\,t12-m1\,(-dd1+(1-p12)\,t12)-m1\,(dd1+p12\,t12)+\\
&\qquad m1\,(dd2+(1-p22)\,(1-t22))+m1\,s1\,(dd2+(1-p22)\,(1-t22))+\\
&\qquad m1\,(-dd2+p22\,(1-t22))+m1\,s1\,(-dd2+p22\,(1-t22))+\\
&\qquad m1\,(-dd2+(1-p22)\,t22)+m1\,(dd2+p22\,t22)+\\
&\qquad (-dd1+p12\,(1-t12))\,\alpha1-m1\,(-dd1+p12\,(1-t12))\,\alpha1+\\
&\qquad s1\,(-dd1+p12\,(1-t12))\,\alpha1-m1\,s1\,(-dd1+p12\,(1-t12))\,\alpha1+\\
&\qquad m1\,(dd2+(1-p22)\,(1-t22))\,\alpha1+m1\,s1\,(dd2+(1-p22)\,(1-t22))\,\alpha1+\\
&\qquad m1\,(-dd2+p22\,(1-t22))\,\alpha1+m1\,s1\,(-dd2+p22\,(1-t22))\,\alpha1-\\
&\qquad (-1+m1)\,(1+s1)\,(dd1+(1-p12)\,(1-t12))\,(1+\alpha1)))-\\
&\quad (2\,((-1+m1)\,(-dd1+(1-p12)\,t12)-m1\,(-dd2+(1-p22)\,t22))^{2})\,/\\
&\quad (-dd1-m1\,(-dd1+p12\,(1-t12))+s1\,(-dd1+p12\,(1-t12))-\\
&\qquad m1\,s1\,(-dd1+p12\,(1-t12))+p12\,(1-t12)+(1-p12)\,t12+p12\,t12-m1\\
&\qquad\; (-dd1+(1-p12)\,t12)-m1\,(dd1+p12\,t12)+m1\,(dd2+(1-p22)\,(1-t22))+\\
&\qquad m1\,s1\,(dd2+(1-p22)\,(1-t22))+m1\,(-dd2+p22\,(1-t22))+\\
&\qquad m1\,s1\,(-dd2+p22\,(1-t22))+m1\,(-dd2+(1-p22)\,t22)+m1\,(dd2+p22\,t22)+\\
&\qquad (-dd1+p12\,(1-t12))\,\alpha1-m1\,(-dd1+p12\,(1-t12))\,\alpha1+\\
&\qquad s1\,(-dd1+p12\,(1-t12))\,\alpha1-m1\,s1\,(-dd1+p12\,(1-t12))\,\alpha1+\\
&\qquad m1\,(dd2+(1-p22)\,(1-t22))\,\alpha1+m1\,s1\,(dd2+(1-p22)\,(1-t22))\,\alpha1+\\
&\qquad m1\,(-dd2+p22\,(1-t22))\,\alpha1+m1\,s1\,(-dd2+p22\,(1-t22))\,\alpha1-\\
&\qquad (-1+m1)\,(1+s1)\,(dd1+(1-p12)\,(1-t12))\,(1+\alpha1))-\\
&\quad (r\,(1+s1)\,((-1+m1)\,(dd1+(1-p12)\,(1-t12))-m1\,(dd2+(1-p22)\\
&\qquad (1-t22)))\,((-1+m1)\,(dd1+p12\,t12)-m1\,(dd2+p22\,t22)))\,/\\
&\quad (-dd1-m1\,(-dd1+p12\,(1-t12))+s1\,(-dd1+p12\,(1-t12))-\\
&\qquad m1\,s1\,(-dd1+p12\,(1-t12))+p12\,(1-t12)+(1-p12)\,t12+p12\,t12-m1\\
&\qquad\; (-dd1+(1-p12)\,t12)-m1\,(dd1+p12\,t12)+m1\,(dd2+(1-p22)\,(1-t22))+\\
&\qquad m1\,s1\,(dd2+(1-p22)\,(1-t22))+m1\,(-dd2+p22\,(1-t22))+\\
&\qquad m1\,s1\,(-dd2+p22\,(1-t22))+m1\,(-dd2+(1-p22)\,t22)+m1\,(dd2+p22\,t22)+\\
&\qquad (-dd1+p12\,(1-t12))\,\alpha1-m1\,(-dd1+p12\,(1-t12))\,\alpha1+\\
&\qquad s1\,(-dd1+p12\,(1-t12))\,\alpha1-m1\,s1\,(-dd1+p12\,(1-t12))\,\alpha1+
\end{aligned}
$$

```
        m1 (dd2 + (1 - p22) (1 - t22)) α1 + m1 s1 (dd2 + (1 - p22) (1 - t22)) α1 +
        m1 (-dd2 + p22 (1 - t22)) α1 + m1 s1 (-dd2 + p22 (1 - t22)) α1 -
        (-1 + m1) (1 + s1) (dd1 + (1 - p12) (1 - t12)) (1 + α1)) -
 (((-1 + m1) (-dd1 + (1 - p12) t12) - m1 (-dd2 + (1 - p22) t22))
    ((-1 + m1) (dd1 + p12 t12) - m1 (dd2 + p22 t22))) /
  (-dd1 - m1 (-dd1 + p12 (1 - t12)) + s1 (-dd1 + p12 (1 - t12)) -
     m1 s1 (-dd1 + p12 (1 - t12)) + p12 (1 - t12) + (1 - p12) t12 + p12 t12 - m1
       (-dd1 + (1 - p12) t12) - m1 (dd1 + p12 t12) + m1 (dd2 + (1 - p22) (1 - t22)) +
     m1 s1 (dd2 + (1 - p22) (1 - t22)) + m1 (-dd2 + p22 (1 - t22)) +
     m1 s1 (-dd2 + p22 (1 - t22)) + m1 (-dd2 + (1 - p22) t22) + m1 (dd2 + p22 t22) +
     (-dd1 + p12 (1 - t12)) α1 - m1 (-dd1 + p12 (1 - t12)) α1 +
     s1 (-dd1 + p12 (1 - t12)) α1 - m1 s1 (-dd1 + p12 (1 - t12)) α1 +
     m1 (dd2 + (1 - p22) (1 - t22)) α1 + m1 s1 (dd2 + (1 - p22) (1 - t22)) α1 +
     m1 (-dd2 + p22 (1 - t22)) α1 + m1 s1 (-dd2 + p22 (1 - t22)) α1 -
     (-1 + m1) (1 + s1) (dd1 + (1 - p12) (1 - t12)) (1 + α1)) -
 ((1 + s1) ((-1 + m1) (dd1 + (1 - p12) (1 - t12)) - m1 (dd2 + (1 - p22) (1 - t22)))
    ((-1 + m1) (-dd1 + (1 - p12) t12) - m1 (-dd2 + (1 - p22) t22)) (1 + α1)) /
  (-dd1 - m1 (-dd1 + p12 (1 - t12)) + s1 (-dd1 + p12 (1 - t12)) -
     m1 s1 (-dd1 + p12 (1 - t12)) + p12 (1 - t12) + (1 - p12) t12 + p12 t12 - m1
       (-dd1 + (1 - p12) t12) - m1 (dd1 + p12 t12) + m1 (dd2 + (1 - p22) (1 - t22)) +
     m1 s1 (dd2 + (1 - p22) (1 - t22)) + m1 (-dd2 + p22 (1 - t22)) +
     m1 s1 (-dd2 + p22 (1 - t22)) + m1 (-dd2 + (1 - p22) t22) + m1 (dd2 + p22 t22) +
     (-dd1 + p12 (1 - t12)) α1 - m1 (-dd1 + p12 (1 - t12)) α1 +
     s1 (-dd1 + p12 (1 - t12)) α1 - m1 s1 (-dd1 + p12 (1 - t12)) α1 +
     m1 (dd2 + (1 - p22) (1 - t22)) α1 + m1 s1 (dd2 + (1 - p22) (1 - t22)) α1 +
     m1 (-dd2 + p22 (1 - t22)) α1 + m1 s1 (-dd2 + p22 (1 - t22)) α1 -
     (-1 + m1) (1 + s1) (dd1 + (1 - p12) (1 - t12)) (1 + α1)) +
 ((-1 + r) (1 + s1) ((-1 + m1) (-dd1 + p12 (1 - t12)) - m1 (-dd2 + p22 (1 - t22)))
    ((-1 + m1) (-dd1 + (1 - p12) t12) - m1 (-dd2 + (1 - p22) t22)) (1 + α1)) /
  (-dd1 - m1 (-dd1 + p12 (1 - t12)) + s1 (-dd1 + p12 (1 - t12)) -
     m1 s1 (-dd1 + p12 (1 - t12)) + p12 (1 - t12) + (1 - p12) t12 + p12 t12 - m1
       (-dd1 + (1 - p12) t12) - m1 (dd1 + p12 t12) + m1 (dd2 + (1 - p22) (1 - t22)) +
     m1 s1 (dd2 + (1 - p22) (1 - t22)) + m1 (-dd2 + p22 (1 - t22)) +
     m1 s1 (-dd2 + p22 (1 - t22)) + m1 (-dd2 + (1 - p22) t22) + m1 (dd2 + p22 t22) +
     (-dd1 + p12 (1 - t12)) α1 - m1 (-dd1 + p12 (1 - t12)) α1 +
     s1 (-dd1 + p12 (1 - t12)) α1 - m1 s1 (-dd1 + p12 (1 - t12)) α1 +
     m1 (dd2 + (1 - p22) (1 - t22)) α1 + m1 s1 (dd2 + (1 - p22) (1 - t22)) α1 +
     m1 (-dd2 + p22 (1 - t22)) α1 + m1 s1 (-dd2 + p22 (1 - t22)) α1 -
     (-1 + m1) (1 + s1) (dd1 + (1 - p12) (1 - t12)) (1 + α1)) -
 (r (1 + s1) ((-1 + m1) (dd1 + (1 - p12) (1 - t12)) - m1 (dd2 + (1 - p22)
          (1 - t22))) ((-1 + m1) (dd1 + p12 t12) - m1 (dd2 + p22 t22))) /
  (- (-1 + m1) (1 + s1) (dd1 + (1 - p12) (1 - t12)) - m1 (-dd1 + p12 (1 - t12)) +
     s1 (-dd1 + p12 (1 - t12)) - m1 s1 (-dd1 + p12 (1 - t12)) + p12 (1 - t12) +
     p12 t12 - m1 (dd1 + p12 t12) + m1 (dd2 + (1 - p22) (1 - t22)) +
     m1 s1 (dd2 + (1 - p22) (1 - t22)) + m1 (-dd2 + p22 (1 - t22)) +
     m1 s1 (-dd2 + p22 (1 - t22)) + m1 (-dd2 + (1 - p22) t22) + m1 (dd2 + p22 t22) +
     (dd1 + p12 t12) α2 - m1 (dd1 + p12 t12) α2 + m1 (-dd2 + (1 - p22) t22) α2 +
     m1 (dd2 + p22 t22) α2 - (-1 + m1) (-dd1 + (1 - p12) t12) (1 + α2)) +
 ((-1 + r) (1 + s1) ((-1 + m1) (-dd1 + p12 (1 - t12)) - m1 (-dd2 + p22 (1 - t22)))
    ((-1 + m1) (-dd1 + (1 - p12) t12) - m1 (-dd2 + (1 - p22) t22)) (1 + α2)) /
```

```
(-(-1 + m1) (1 + s1) (dd1 + (1 - p12) (1 - t12)) - m1 (-dd1 + p12 (1 - t12)) +
  s1 (-dd1 + p12 (1 - t12)) - m1 s1 (-dd1 + p12 (1 - t12)) + p12 (1 - t12) +
  p12 t12 - m1 (dd1 + p12 t12) + m1 (dd2 + (1 - p22) (1 - t22)) +
  m1 s1 (dd2 + (1 - p22) (1 - t22)) + m1 (-dd2 + p22 (1 - t22)) +
  m1 s1 (-dd2 + p22 (1 - t22)) + m1 (-dd2 + (1 - p22) t22) + m1 (dd2 + p22 t22) +
  (dd1 + p12 t12) α2 - m1 (dd1 + p12 t12) α2 + m1 (-dd2 + (1 - p22) t22) α2 +
  m1 (dd2 + p22 t22) α2 - (-1 + m1) (-dd1 + (1 - p12) t12) (1 + α2)) -
(((-1 + m1) (-dd1 + (1 - p12) t12) - m1 (-dd2 + (1 - p22) t22))
  ((-1 + m1) (dd1 + p12 t12) - m1 (dd2 + p22 t22)) (1 + α2)) /
 (-(-1 + m1) (1 + s1) (dd1 + (1 - p12) (1 - t12)) - m1 (-dd1 + p12 (1 - t12)) +
  s1 (-dd1 + p12 (1 - t12)) - m1 s1 (-dd1 + p12 (1 - t12)) + p12 (1 - t12) +
  p12 t12 - m1 (dd1 + p12 t12) + m1 (dd2 + (1 - p22) (1 - t22)) +
  m1 s1 (dd2 + (1 - p22) (1 - t22)) + m1 (-dd2 + p22 (1 - t22)) +
  m1 s1 (-dd2 + p22 (1 - t22)) + m1 (-dd2 + (1 - p22) t22) + m1 (dd2 + p22 t22) +
  (dd1 + p12 t12) α2 - m1 (dd1 + p12 t12) α2 + m1 (-dd2 + (1 - p22) t22) α2 +
  m1 (dd2 + p22 t22) α2 - (-1 + m1) (-dd1 + (1 - p12) t12) (1 + α2)))) /
(4 (1 + s1 (-m1 (dd1 + (1 - p12) (1 - t12)) - m1 (-dd1 + p12 (1 - t12)) +
      (1 - p12) (1 - t12) + p12 (1 - t12) +
      m1 (dd2 + (1 - p22) (1 - t22)) + m1 (-dd2 + p22 (1 - t22))))^4)) +
((1 + s1) (-1 + s1 ((-1 + m1) (dd1 + (1 - p12) (1 - t12)) + (-1 + m1) (-dd1 +
       p12 (1 - t12)) - m1 ((1 - p22) (1 - t22) + p22 (1 - t22))))^2
 (-((((-1 + m1) (dd1 + (1 - p12) (1 - t12)) - m1 (dd2 + (1 - p22) (1 - t22)))
     ((-1 + m1) (-dd1 + (1 - p12) t12) - m1 (-dd2 + (1 - p22) t22))) /
    (-dd1 - m1 (-dd1 + p12 (1 - t12)) + s1 (-dd1 + p12 (1 - t12)) -
     m1 s1 (-dd1 + p12 (1 - t12)) + p12 (1 - t12) + (1 - p12) t12 + p12 t12 - m1
       (-dd1 + (1 - p12) t12) - m1 (dd1 + p12 t12) + m1 (dd2 + (1 - p22) (1 - t22)) +
     m1 s1 (dd2 + (1 - p22) (1 - t22)) + m1 (-dd2 + p22 (1 - t22)) +
     m1 s1 (-dd2 + p22 (1 - t22)) + m1 (-dd2 + (1 - p22) t22) + m1 (dd2 + p22 t22) +
     (-dd1 + p12 (1 - t12)) α1 - m1 (-dd1 + p12 (1 - t12)) α1 +
     s1 (-dd1 + p12 (1 - t12)) α1 - m1 s1 (-dd1 + p12 (1 - t12)) α1 +
     m1 (dd2 + (1 - p22) (1 - t22)) α1 + m1 s1 (dd2 + (1 - p22) (1 - t22)) α1 +
     m1 (-dd2 + p22 (1 - t22)) α1 + m1 s1 (-dd2 + p22 (1 - t22)) α1 -
     (-1 + m1) (1 + s1) (dd1 + (1 - p12) (1 - t12)) (1 + α1))) +
   ((-1 + r) ((-1 + m1) (dd1 + (1 - p12) (1 - t12)) - m1 (dd2 + (1 - p22) (1 - t22)))
     ((-1 + m1) (dd1 + p12 t12) - m1 (dd2 + p22 t22))) /
   (-dd1 - m1 (-dd1 + p12 (1 - t12)) + s1 (-dd1 + p12 (1 - t12)) -
    m1 s1 (-dd1 + p12 (1 - t12)) + p12 (1 - t12) + (1 - p12) t12 +
    p12 t12 - m1 (-dd1 + (1 - p12) t12) - m1 (dd1 + p12 t12) +
    m1 (dd2 + (1 - p22) (1 - t22)) + m1 s1 (dd2 + (1 - p22) (1 - t22)) +
    m1 (-dd2 + p22 (1 - t22)) + m1 s1 (-dd2 + p22 (1 - t22)) +
    m1 (-dd2 + (1 - p22) t22) + m1 (dd2 + p22 t22) +
    (-dd1 + p12 (1 - t12)) α1 - m1 (-dd1 + p12 (1 - t12)) α1 +
    s1 (-dd1 + p12 (1 - t12)) α1 - m1 s1 (-dd1 + p12 (1 - t12)) α1 +
    m1 (dd2 + (1 - p22) (1 - t22)) α1 + m1 s1 (dd2 + (1 - p22) (1 - t22)) α1 +
    m1 (-dd2 + p22 (1 - t22)) α1 + m1 s1 (-dd2 + p22 (1 - t22)) α1 -
    (-1 + m1) (1 + s1) (dd1 + (1 - p12) (1 - t12)) (1 + α1)) -
   (2 (1 + s1) ((-1 + m1) (dd1 + (1 - p12) (1 - t12)) - m1 (dd2 + (1 - p22) (1 - t22)))^2
     (1 + α1)) / (-dd1 - m1 (-dd1 + p12 (1 - t12)) + s1 (-dd1 + p12 (1 - t12)) -
```

```
      m1 s1 (-dd1 + p12 (1 - t12)) + p12 (1 - t12) + (1 - p12) t12 +
      p12 t12 - m1 (-dd1 + (1 - p12) t12) - m1 (dd1 + p12 t12) +
      m1 (dd2 + (1 - p22) (1 - t22)) + m1 s1 (dd2 + (1 - p22) (1 - t22)) +
      m1 (-dd2 + p22 (1 - t22)) + m1 s1 (-dd2 + p22 (1 - t22)) +
      m1 (-dd2 + (1 - p22) t22) + m1 (dd2 + p22 t22) +
      (-dd1 + p12 (1 - t12)) α1 - m1 (-dd1 + p12 (1 - t12)) α1 +
      s1 (-dd1 + p12 (1 - t12)) α1 - m1 s1 (-dd1 + p12 (1 - t12)) α1 +
      m1 (dd2 + (1 - p22) (1 - t22)) α1 + m1 s1 (dd2 + (1 - p22) (1 - t22)) α1 +
      m1 (-dd2 + p22 (1 - t22)) α1 + m1 s1 (-dd2 + p22 (1 - t22)) α1 -
      (-1 + m1) (1 + s1) (dd1 + (1 - p12) (1 - t12)) (1 + α1)) -
  ((1 + s1) ((-1 + m1) (dd1 + (1 - p12) (1 - t12)) - m1 (dd2 + (1 - p22) (1 - t22)))
      ((-1 + m1) (-dd1 + p12 (1 - t12)) - m1 (-dd2 + p22 (1 - t22))) (1 + α1)) /
   (-dd1 - m1 (-dd1 + p12 (1 - t12)) + s1 (-dd1 + p12 (1 - t12)) -
      m1 s1 (-dd1 + p12 (1 - t12)) + p12 (1 - t12) + (1 - p12) t12 +
      p12 t12 - m1 (-dd1 + (1 - p12) t12) - m1 (dd1 + p12 t12) +
      m1 (dd2 + (1 - p22) (1 - t22)) + m1 s1 (dd2 + (1 - p22) (1 - t22)) +
      m1 (-dd2 + p22 (1 - t22)) + m1 s1 (-dd2 + p22 (1 - t22)) +
      m1 (-dd2 + (1 - p22) t22) + m1 (dd2 + p22 t22) +
      (-dd1 + p12 (1 - t12)) α1 - m1 (-dd1 + p12 (1 - t12)) α1 +
      s1 (-dd1 + p12 (1 - t12)) α1 - m1 s1 (-dd1 + p12 (1 - t12)) α1 +
      m1 (dd2 + (1 - p22) (1 - t22)) α1 + m1 s1 (dd2 + (1 - p22) (1 - t22)) α1 +
      m1 (-dd2 + p22 (1 - t22)) α1 + m1 s1 (-dd2 + p22 (1 - t22)) α1 -
      (-1 + m1) (1 + s1) (dd1 + (1 - p12) (1 - t12)) (1 + α1)) -
  (((-1 + m1) (dd1 + (1 - p12) (1 - t12)) - m1 (dd2 + (1 - p22) (1 - t22)))
      ((-1 + m1) (-dd1 + (1 - p12) t12) - m1 (-dd2 + (1 - p22) t22)) (1 + α1)) /
   (-dd1 - m1 (-dd1 + p12 (1 - t12)) + s1 (-dd1 + p12 (1 - t12)) -
      m1 s1 (-dd1 + p12 (1 - t12)) + p12 (1 - t12) + (1 - p12) t12 +
      p12 t12 - m1 (-dd1 + (1 - p12) t12) - m1 (dd1 + p12 t12) +
      m1 (dd2 + (1 - p22) (1 - t22)) + m1 s1 (dd2 + (1 - p22) (1 - t22)) +
      m1 (-dd2 + p22 (1 - t22)) + m1 s1 (-dd2 + p22 (1 - t22)) +
      m1 (-dd2 + (1 - p22) t22) + m1 (dd2 + p22 t22) +
      (-dd1 + p12 (1 - t12)) α1 - m1 (-dd1 + p12 (1 - t12)) α1 +
      s1 (-dd1 + p12 (1 - t12)) α1 - m1 s1 (-dd1 + p12 (1 - t12)) α1 +
      m1 (dd2 + (1 - p22) (1 - t22)) α1 + m1 s1 (dd2 + (1 - p22) (1 - t22)) α1 +
      m1 (-dd2 + p22 (1 - t22)) α1 + m1 s1 (-dd2 + p22 (1 - t22)) α1 -
      (-1 + m1) (1 + s1) (dd1 + (1 - p12) (1 - t12)) (1 + α1)) -
  (r ((-1 + m1) (-dd1 + p12 (1 - t12)) - m1 (-dd2 + p22 (1 - t22)))
      ((-1 + m1) (-dd1 + (1 - p12) t12) - m1 (-dd2 + (1 - p22) t22)) (1 + α1)) /
   (-dd1 - m1 (-dd1 + p12 (1 - t12)) + s1 (-dd1 + p12 (1 - t12)) -
      m1 s1 (-dd1 + p12 (1 - t12)) + p12 (1 - t12) + (1 - p12) t12 +
      p12 t12 - m1 (-dd1 + (1 - p12) t12) - m1 (dd1 + p12 t12) +
      m1 (dd2 + (1 - p22) (1 - t22)) + m1 s1 (dd2 + (1 - p22) (1 - t22)) +
      m1 (-dd2 + p22 (1 - t22)) + m1 s1 (-dd2 + p22 (1 - t22)) +
      m1 (-dd2 + (1 - p22) t22) + m1 (dd2 + p22 t22) +
      (-dd1 + p12 (1 - t12)) α1 - m1 (-dd1 + p12 (1 - t12)) α1 +
      s1 (-dd1 + p12 (1 - t12)) α1 - m1 s1 (-dd1 + p12 (1 - t12)) α1 +
      m1 (dd2 + (1 - p22) (1 - t22)) α1 + m1 s1 (dd2 + (1 - p22) (1 - t22)) α1 +
      m1 (-dd2 + p22 (1 - t22)) α1 + m1 s1 (-dd2 + p22 (1 - t22)) α1 -
      (-1 + m1) (1 + s1) (dd1 + (1 - p12) (1 - t12)) (1 + α1)) -
  ((1 + s1) ((-1 + m1) (dd1 + (1 - p12) (1 - t12)) - m1 (dd2 + (1 - p22) (1 - t22)))
```

```
        ((-1 + m1) (-dd1 + p12 (1 - t12)) - m1 (-dd2 + p22 (1 - t22)))) /
      (-(-1 + m1) (1 + s1) (dd1 + (1 - p12) (1 - t12)) - m1 (-dd1 + p12 (1 - t12)) +
        s1 (-dd1 + p12 (1 - t12)) - m1 s1 (-dd1 + p12 (1 - t12)) + p12 (1 - t12) +
        p12 t12 - m1 (dd1 + p12 t12) + m1 (dd2 + (1 - p22) (1 - t22)) +
        m1 s1 (dd2 + (1 - p22) (1 - t22)) + m1 (-dd2 + p22 (1 - t22)) +
        m1 s1 (-dd2 + p22 (1 - t22)) + m1 (-dd2 + (1 - p22) t22) + m1 (dd2 + p22 t22) +
        (dd1 + p12 t12) α2 - m1 (dd1 + p12 t12) α2 + m1 (-dd2 + (1 - p22) t22) α2 +
        m1 (dd2 + p22 t22) α2 - (-1 + m1) (-dd1 + (1 - p12) t12) (1 + α2)) +
    ((-1 + r) ((-1 + m1) (dd1 + (1 - p12) (1 - t12)) - m1 (dd2 + (1 - p22) (1 - t22)))
        ((-1 + m1) (dd1 + p12 t12) - m1 (dd2 + p22 t22))) /
      (-(-1 + m1) (1 + s1) (dd1 + (1 - p12) (1 - t12)) - m1 (-dd1 + p12 (1 - t12)) +
        s1 (-dd1 + p12 (1 - t12)) - m1 s1 (-dd1 + p12 (1 - t12)) + p12 (1 - t12) +
        p12 t12 - m1 (dd1 + p12 t12) + m1 (dd2 + (1 - p22) (1 - t22)) +
        m1 s1 (dd2 + (1 - p22) (1 - t22)) + m1 (-dd2 + p22 (1 - t22)) +
        m1 s1 (-dd2 + p22 (1 - t22)) + m1 (-dd2 + (1 - p22) t22) + m1 (dd2 + p22 t22) +
        (dd1 + p12 t12) α2 - m1 (dd1 + p12 t12) α2 + m1 (-dd2 + (1 - p22) t22) α2 +
        m1 (dd2 + p22 t22) α2 - (-1 + m1) (-dd1 + (1 - p12) t12) (1 + α2)) -
    (r ((-1 + m1) (-dd1 + p12 (1 - t12)) - m1 (-dd2 + p22 (1 - t22)))
        ((-1 + m1) (-dd1 + (1 - p12) t12) - m1 (-dd2 + (1 - p22) t22)) (1 + α2)) /
      (-(-1 + m1) (1 + s1) (dd1 + (1 - p12) (1 - t12)) - m1 (-dd1 + p12 (1 - t12)) +
        s1 (-dd1 + p12 (1 - t12)) - m1 s1 (-dd1 + p12 (1 - t12)) + p12 (1 - t12) +
        p12 t12 - m1 (dd1 + p12 t12) + m1 (dd2 + (1 - p22) (1 - t22)) +
        m1 s1 (dd2 + (1 - p22) (1 - t22)) + m1 (-dd2 + p22 (1 - t22)) +
        m1 s1 (-dd2 + p22 (1 - t22)) + m1 (-dd2 + (1 - p22) t22) + m1 (dd2 + p22 t22) +
        (dd1 + p12 t12) α2 - m1 (dd1 + p12 t12) α2 + m1 (-dd2 + (1 - p22) t22) α2 +
        m1 (dd2 + p22 t22) α2 - (-1 + m1) (-dd1 + (1 - p12) t12) (1 + α2)))
  (((-1 + r) (1 + s1) ((-1 + m1) (dd1 + (1 - p12) (1 - t12)) - m1 (dd2 +
              (1 - p22) (1 - t22))) ((-1 + m1) (dd1 + p12 t12) - m1 (dd2 + p22 t22))) /
      (-dd1 - m1 (-dd1 + p12 (1 - t12)) + s1 (-dd1 + p12 (1 - t12)) -
        m1 s1 (-dd1 + p12 (1 - t12)) + p12 (1 - t12) + (1 - p12) t12 + p12 t12 -
        m1 (-dd1 + (1 - p12) t12) - m1 (dd1 + p12 t12) + m1 (dd2 + (1 - p22) (1 - t22)) +
        m1 s1 (dd2 + (1 - p22) (1 - t22)) + m1 (-dd2 + p22 (1 - t22)) +
        m1 s1 (-dd2 + p22 (1 - t22)) + m1 (-dd2 + (1 - p22) t22) + m1 (dd2 + p22 t22) +
        (-dd1 + p12 (1 - t12)) α1 - m1 (-dd1 + p12 (1 - t12)) α1 +
        s1 (-dd1 + p12 (1 - t12)) α1 - m1 s1 (-dd1 + p12 (1 - t12)) α1 +
        m1 (dd2 + (1 - p22) (1 - t22)) α1 + m1 s1 (dd2 + (1 - p22) (1 - t22)) α1 +
        m1 (-dd2 + p22 (1 - t22)) α1 + m1 s1 (-dd2 + p22 (1 - t22)) α1 -
        (-1 + m1) (1 + s1) (dd1 + (1 - p12) (1 - t12)) (1 + α1)) -
    (((-1 + m1) (-dd1 + (1 - p12) t12) - m1 (-dd2 + (1 - p22) t22))
        ((-1 + m1) (dd1 + p12 t12) - m1 (dd2 + p22 t22))) /
      (-dd1 - m1 (-dd1 + p12 (1 - t12)) + s1 (-dd1 + p12 (1 - t12)) -
        m1 s1 (-dd1 + p12 (1 - t12)) + p12 (1 - t12) + (1 - p12) t12 + p12 t12 -
        m1 (-dd1 + (1 - p12) t12) - m1 (dd1 + p12 t12) + m1 (dd2 + (1 - p22) (1 - t22)) +
        m1 s1 (dd2 + (1 - p22) (1 - t22)) + m1 (-dd2 + p22 (1 - t22)) +
        m1 s1 (-dd2 + p22 (1 - t22)) + m1 (-dd2 + (1 - p22) t22) + m1 (dd2 + p22 t22) +
        (-dd1 + p12 (1 - t12)) α1 - m1 (-dd1 + p12 (1 - t12)) α1 +
        s1 (-dd1 + p12 (1 - t12)) α1 - m1 s1 (-dd1 + p12 (1 - t12)) α1 +
        m1 (dd2 + (1 - p22) (1 - t22)) α1 + m1 s1 (dd2 + (1 - p22) (1 - t22)) α1 +
        m1 (-dd2 + p22 (1 - t22)) α1 + m1 s1 (-dd2 + p22 (1 - t22)) α1 -
        (-1 + m1) (1 + s1) (dd1 + (1 - p12) (1 - t12)) (1 + α1)) -
```

```
(r (1 + s1) ((-1 + m1) (-dd1 + p12 (1 - t12)) - m1 (-dd2 + p22 (1 - t22)))
    ((-1 + m1) (-dd1 + (1 - p12) t12) - m1 (-dd2 + (1 - p22) t22)) (1 + α1)) /
  (-dd1 - m1 (-dd1 + p12 (1 - t12)) + s1 (-dd1 + p12 (1 - t12)) -
    m1 s1 (-dd1 + p12 (1 - t12)) + p12 (1 - t12) + (1 - p12) t12 + p12 t12 -
    m1 (-dd1 + (1 - p12) t12) - m1 (dd1 + p12 t12) + m1 (dd2 + (1 - p22) (1 - t22)) +
    m1 s1 (dd2 + (1 - p22) (1 - t22)) + m1 (-dd2 + p22 (1 - t22)) +
    m1 s1 (-dd2 + p22 (1 - t22)) + m1 (-dd2 + (1 - p22) t22) + m1 (dd2 + p22 t22) +
    (-dd1 + p12 (1 - t12)) α1 - m1 (-dd1 + p12 (1 - t12)) α1 +
    s1 (-dd1 + p12 (1 - t12)) α1 - m1 s1 (-dd1 + p12 (1 - t12)) α1 +
    m1 (dd2 + (1 - p22) (1 - t22)) α1 + m1 s1 (dd2 + (1 - p22) (1 - t22)) α1 +
    m1 (-dd2 + p22 (1 - t22)) α1 + m1 s1 (-dd2 + p22 (1 - t22)) α1 -
    (-1 + m1) (1 + s1) (dd1 + (1 - p12) (1 - t12)) (1 + α1)) +
((-1 + r) (1 + s1) ((-1 + m1) (dd1 + (1 - p12) (1 - t12)) - m1 (dd2 +
          (1 - p22) (1 - t22))) ((-1 + m1) (dd1 + p12 t12) - m1 (dd2 + p22 t22))) /
  (-(-1 + m1) (1 + s1) (dd1 + (1 - p12) (1 - t12)) - m1 (-dd1 + p12 (1 - t12)) +
    s1 (-dd1 + p12 (1 - t12)) - m1 s1 (-dd1 + p12 (1 - t12)) + p12 (1 - t12) +
    p12 t12 - m1 (dd1 + p12 t12) + m1 (dd2 + (1 - p22) (1 - t22)) +
    m1 s1 (dd2 + (1 - p22) (1 - t22)) + m1 (-dd2 + p22 (1 - t22)) +
    m1 s1 (-dd2 + p22 (1 - t22)) + m1 (-dd2 + (1 - p22) t22) + m1 (dd2 + p22 t22) +
    (dd1 + p12 t12) α2 - m1 (dd1 + p12 t12) α2 + m1 (-dd2 + (1 - p22) t22) α2 +
    m1 (dd2 + p22 t22) α2 - (-1 + m1) (-dd1 + (1 - p12) t12) (1 + α2)) -
((1 + s1) ((-1 + m1) (-dd1 + p12 (1 - t12)) - m1 (-dd2 + p22 (1 - t22)))
    ((-1 + m1) (dd1 + p12 t12) - m1 (dd2 + p22 t22))) /
  (-(-1 + m1) (1 + s1) (dd1 + (1 - p12) (1 - t12)) - m1 (-dd1 + p12 (1 - t12)) +
    s1 (-dd1 + p12 (1 - t12)) - m1 s1 (-dd1 + p12 (1 - t12)) + p12 (1 - t12) +
    p12 t12 - m1 (dd1 + p12 t12) + m1 (dd2 + (1 - p22) (1 - t22)) +
    m1 s1 (dd2 + (1 - p22) (1 - t22)) + m1 (-dd2 + p22 (1 - t22)) +
    m1 s1 (-dd2 + p22 (1 - t22)) + m1 (-dd2 + (1 - p22) t22) + m1 (dd2 + p22 t22) +
    (dd1 + p12 t12) α2 - m1 (dd1 + p12 t12) α2 + m1 (-dd2 + (1 - p22) t22) α2 +
    m1 (dd2 + p22 t22) α2 - (-1 + m1) (-dd1 + (1 - p12) t12) (1 + α2)) -
(r (1 + s1) ((-1 + m1) (-dd1 + p12 (1 - t12)) - m1 (-dd2 + p22 (1 - t22)))
    ((-1 + m1) (-dd1 + (1 - p12) t12) - m1 (-dd2 + (1 - p22) t22)) (1 + α2)) /
  (-(-1 + m1) (1 + s1) (dd1 + (1 - p12) (1 - t12)) - m1 (-dd1 + p12 (1 - t12)) +
    s1 (-dd1 + p12 (1 - t12)) - m1 s1 (-dd1 + p12 (1 - t12)) + p12 (1 - t12) +
    p12 t12 - m1 (dd1 + p12 t12) + m1 (dd2 + (1 - p22) (1 - t22)) +
    m1 s1 (dd2 + (1 - p22) (1 - t22)) + m1 (-dd2 + p22 (1 - t22)) +
    m1 s1 (-dd2 + p22 (1 - t22)) + m1 (-dd2 + (1 - p22) t22) + m1 (dd2 + p22 t22) +
    (dd1 + p12 t12) α2 - m1 (dd1 + p12 t12) α2 + m1 (-dd2 + (1 - p22) t22) α2 +
    m1 (dd2 + p22 t22) α2 - (-1 + m1) (-dd1 + (1 - p12) t12) (1 + α2)) -
((1 + s1) ((-1 + m1) (-dd1 + p12 (1 - t12)) - m1 (-dd2 + p22 (1 - t22)))
    ((-1 + m1) (dd1 + p12 t12) - m1 (dd2 + p22 t22)) (1 + α2)) /
  (-(-1 + m1) (1 + s1) (dd1 + (1 - p12) (1 - t12)) - m1 (-dd1 + p12 (1 - t12)) +
    s1 (-dd1 + p12 (1 - t12)) - m1 s1 (-dd1 + p12 (1 - t12)) + p12 (1 - t12) +
    p12 t12 - m1 (dd1 + p12 t12) + m1 (dd2 + (1 - p22) (1 - t22)) +
    m1 s1 (dd2 + (1 - p22) (1 - t22)) + m1 (-dd2 + p22 (1 - t22)) +
    m1 s1 (-dd2 + p22 (1 - t22)) + m1 (-dd2 + (1 - p22) t22) + m1 (dd2 + p22 t22) +
    (dd1 + p12 t12) α2 - m1 (dd1 + p12 t12) α2 + m1 (-dd2 + (1 - p22) t22) α2 +
    m1 (dd2 + p22 t22) α2 - (-1 + m1) (-dd1 + (1 - p12) t12) (1 + α2)) -
((((-1 + m1) (-dd1 + (1 - p12) t12) - m1 (-dd2 + (1 - p22) t22))
    ((-1 + m1) (dd1 + p12 t12) - m1 (dd2 + p22 t22)) (1 + α2)) /
```

$$(-(-1+m1)\,(1+s1)\,(dd1+(1-p12)\,(1-t12))-m1\,(-dd1+p12\,(1-t12))+$$
$$s1\,(-dd1+p12\,(1-t12))-m1\,s1\,(-dd1+p12\,(1-t12))+p12\,(1-t12)+$$
$$p12\,t12-m1\,(dd1+p12\,t12)+m1\,(dd2+(1-p22)\,(1-t22))+$$
$$m1\,s1\,(dd2+(1-p22)\,(1-t22))+m1\,(-dd2+p22\,(1-t22))+$$
$$m1\,s1\,(-dd2+p22\,(1-t22))+m1\,(-dd2+(1-p22)\,t22)+m1\,(dd2+p22\,t22)+$$
$$(dd1+p12\,t12)\,\alpha2-m1\,(dd1+p12\,t12)\,\alpha2+m1\,(-dd2+(1-p22)\,t22)\,\alpha2+$$
$$m1\,(dd2+p22\,t22)\,\alpha2-(-1+m1)\,(-dd1+(1-p12)\,t12)\,(1+\alpha2))-$$
$$\left(2\,((-1+m1)\,(dd1+p12\,t12)-m1\,(dd2+p22\,t22))^2\,(1+\alpha2)\right)\Big/$$
$$(-(-1+m1)\,(1+s1)\,(dd1+(1-p12)\,(1-t12))-m1\,(-dd1+p12\,(1-t12))+$$
$$s1\,(-dd1+p12\,(1-t12))-m1\,s1\,(-dd1+p12\,(1-t12))+p12\,(1-t12)+$$
$$p12\,t12-m1\,(dd1+p12\,t12)+m1\,(dd2+(1-p22)\,(1-t22))+$$
$$m1\,s1\,(dd2+(1-p22)\,(1-t22))+m1\,(-dd2+p22\,(1-t22))+$$
$$m1\,s1\,(-dd2+p22\,(1-t22))+m1\,(-dd2+(1-p22)\,t22)+m1\,(dd2+p22\,t22)+$$
$$(dd1+p12\,t12)\,\alpha2-m1\,(dd1+p12\,t12)\,\alpha2+m1\,(-dd2+(1-p22)\,t22)\,\alpha2+$$
$$m1\,(dd2+p22\,t22)\,\alpha2-(-1+m1)\,(-dd1+(1-p12)\,t12)\,(1+\alpha2))))\Big/$$
$$\Big(4\,(1+s1\,(-m1\,(dd1+(1-p12)\,(1-t12))-m1\,(-dd1+p12\,(1-t12))+$$
$$(1-p12)\,(1-t12)+p12\,(1-t12)+$$
$$m1\,(dd2+(1-p22)\,(1-t22))+m1\,(-dd2+p22\,(1-t22))))^4\Big),$$
$$(p22\,(1+s2\,t22)\,(2+dd2\,\alpha2+2\,t22\,\alpha2+2\,s2^2\,t22^2\,(1+\alpha2)+$$
$$\alpha1\,(dd2\,(1+\alpha2)-2\,(-1+t22)\,(1+t22\,\alpha2))+$$
$$s2\,(-2\,t22^2\,(\alpha1-\alpha2+\alpha1\,\alpha2)+dd2\,(\alpha1+\alpha2+\alpha1\,\alpha2)+2\,t22\,(2+\alpha1+\alpha2+\alpha1\,\alpha2)))+$$
$$dd2\,(2\,s2^3\,t22^2\,(1+\alpha2)-\alpha1\,(1+t22\,\alpha2)+$$
$$s2\,(2+dd2\,\alpha2+2\,t22\,\alpha2+\alpha1\,(1+dd2-3\,t22+dd2\,\alpha2-2\,t22^2\,\alpha2))+$$
$$s2^2\,(-2\,t22^2\,(\alpha1-\alpha2+\alpha1\,\alpha2)+dd2\,(\alpha1+\alpha2+\alpha1\,\alpha2)+t22\,(4+\alpha1+2\,\alpha2+\alpha1\,\alpha2)))-$$
$$m2^3\,(t12-t22)\,(-2\,(dd1-dd2)\,s2\,(t12-t22)\,(s2-\alpha1)\,(s2+\alpha2+s2\,\alpha2)+$$
$$p12^2\,(1+s2)\,(1+s2\,t12)\,(\alpha1+\alpha2+\alpha1\,\alpha2)+$$
$$p22^2\,(1+s2)\,(1+s2\,t22)\,(\alpha1+\alpha2+\alpha1\,\alpha2)-$$
$$p12\,(2\,p22\,\alpha1+2\,p22\,\alpha2+2\,p22\,\alpha1\,\alpha2-t12\,\alpha1\,\alpha2+t22\,\alpha1\,\alpha2+$$
$$2\,s2^3\,t12\,(t12-t22)\,(1+\alpha2)+s2\,(2\,p22\,\alpha1-t12\,\alpha1+p22\,t12\,\alpha1+t22$$
$$\alpha1+p22\,t22\,\alpha1+2\,p22\,\alpha2+2\,t12\,\alpha2+p22\,t12\,\alpha2-2\,t22\,\alpha2+p22\,t22$$
$$\alpha2+2\,p22\,\alpha1\,\alpha2+p22\,t12\,\alpha1\,\alpha2-2\,t12^2\,\alpha1\,\alpha2+p22\,t22\,\alpha1\,\alpha2+2$$
$$t12\,t22\,\alpha1\,\alpha2-dd1\,(\alpha1+\alpha2+\alpha1\,\alpha2)+dd2\,(\alpha1+\alpha2+\alpha1\,\alpha2))+$$
$$s2^2\,(-2\,t12^2\,(\alpha1-\alpha2+\alpha1\,\alpha2)-(dd1-dd2)\,(\alpha1+\alpha2+\alpha1\,\alpha2)+t22$$
$$(-2+(-2+p22)\,\alpha2+(-1+p22)\,\alpha1\,(1+\alpha2))+t12$$
$$(2+(2+p22-2\,t22)\,\alpha2+(1+p22+2\,t22)\,\alpha1\,(1+\alpha2))))+$$
$$p22\,((-t12+t22)\,\alpha1\,\alpha2+2\,s2^3\,(t12-t22)\,t22\,(1+\alpha2)+s2\,(-dd1\,(\alpha1+\alpha2+$$
$$\alpha1\,\alpha2)+dd2\,(\alpha1+\alpha2+\alpha1\,\alpha2)-(t12-t22)\,(\alpha1-2\,\alpha2+2\,t22\,\alpha1\,\alpha2))+$$
$$s2^2\,(-t22\,(2+\alpha1)\,(1+\alpha2)+2\,t22^2\,(\alpha1-\alpha2+\alpha1\,\alpha2)-(dd1-dd2)\,(\alpha1+$$
$$\alpha2+\alpha1\,\alpha2)+t12\,(-(-1+2\,t22)\,\alpha1\,(1+\alpha2)+2\,(1+\alpha2+t22\,\alpha2))))))+$$
$$m2\,(2\,dd1\,s2-2\,dd2\,s2+4\,dd2\,s2^2\,t12+4\,dd1\,s2^2\,t22-8\,dd2\,s2^2\,t22+$$
$$4\,dd2\,s2^3\,t12\,t22+2\,dd1\,s2^3\,t22^2-$$
$$6\,dd2\,s2^3\,t22^2-dd1\,\alpha1+dd2\,\alpha1+$$
$$dd1\,s2\,\alpha1-dd2\,s2\,\alpha1+2\,dd1\,dd2\,s2\,\alpha1-$$
$$2\,dd2^2\,s2\,\alpha1+2\,dd1\,dd2\,s2^2\,\alpha1-$$
$$2\,dd2^2\,s2^2\,\alpha1-3\,dd2\,s2\,t12\,\alpha1+$$
$$dd2\,s2^2\,t12\,\alpha1-3\,dd1\,s2\,t22\,\alpha1+$$
$$6\,dd2\,s2\,t22\,\alpha1+dd1\,s2^2\,t22\,\alpha1-$$
$$2\,dd2\,s2^2\,t22\,\alpha1-4\,dd2\,s2^2\,t12\,t22\,\alpha1-$$
$$2\,dd1\,s2^2\,t22^2\,\alpha1+6\,dd2\,s2^2\,t22^2\,\alpha1+$$
$$2\,dd1\,dd2\,s2\,\alpha2-2\,dd2^2\,s2\,\alpha2+$$

$2\, dd1\, dd2\, s2^{2}\, \alpha2 - 2\, dd2^{2}\, s2^{2}\, \alpha2 +$
$2\, dd2\, s2\, t12\, \alpha2 + 2\, dd2\, s2^{2}\, t12\, \alpha2 +$
$2\, dd1\, s2\, t22\, \alpha2 - 4\, dd2\, s2\, t22\, \alpha2 +$
$2\, dd1\, s2^{2}\, t22\, \alpha2 - 4\, dd2\, s2^{2}\, t22\, \alpha2 +$
$4\, dd2\, s2^{2}\, t12\, t22\, \alpha2 + 4\, dd2\, s2^{3}\, t12\, t22\, \alpha2 +$
$2\, dd1\, s2^{2}\, t22^{2}\, \alpha2 - 6\, dd2\, s2^{2}\, t22^{2}\, \alpha2 +$
$2\, dd1\, s2^{3}\, t22^{2}\, \alpha2 - 6\, dd2\, s2^{3}\, t22^{2}\, \alpha2 +$
$2\, dd1\, dd2\, s2\, \alpha1\, \alpha2 - 2\, dd2^{2}\, s2\, \alpha1\, \alpha2 +$
$2\, dd1\, dd2\, s2^{2}\, \alpha1\, \alpha2 - 2\, dd2^{2}\, s2^{2}\, \alpha1\, \alpha2 -$
$dd2\, t12\, \alpha1\, \alpha2 + dd2\, s2^{2}\, t12\, \alpha1\, \alpha2 -$
$dd1\, t22\, \alpha1\, \alpha2 + 2\, dd2\, t22\, \alpha1\, \alpha2 +$
$dd1\, s2^{2}\, t22\, \alpha1\, \alpha2 - 2\, dd2\, s2^{2}\, t22\, \alpha1\, \alpha2 -$
$4\, dd2\, s2\, t12\, t22\, \alpha1\, \alpha2 - 4\, dd2\, s2^{2}\, t12\, t22\, \alpha1\, \alpha2 -$
$2\, dd1\, s2\, t22^{2}\, \alpha1\, \alpha2 + 6\, dd2\, s2\, t22^{2}\, \alpha1\, \alpha2 -$
$2\, dd1\, s2^{2}\, t22^{2}\, \alpha1\, \alpha2 + 6\, dd2\, s2^{2}\, t22^{2}\, \alpha1\, \alpha2 -$
$p22^{2}\, (1 + s2)\, (t12 - t22)\, (1 + s2\, t22)\, (\alpha1 + \alpha2 + \alpha1\, \alpha2) +$
$p22\, (-2 - 2\, \alpha1 + dd1\, \alpha1 - 2\, dd2\, \alpha1 - t12\, \alpha1 + 3\, t22\, \alpha1 + dd1\, \alpha2 - 2\, dd2\, \alpha2 +$
$2\, t12\, \alpha2 - 4\, t22\, \alpha2 + dd1\, \alpha1\, \alpha2 - 2\, dd2\, \alpha1\, \alpha2 + 2\, t12\, \alpha1\, \alpha2 - 4\, t22\, \alpha1\, \alpha2 -$
$3\, t12\, t22\, \alpha1\, \alpha2 + 5\, t22^{2}\, \alpha1\, \alpha2 + 2\, s2^{3}\, (2\, t12 - 3\, t22)\, t22^{2}\, (1 + \alpha2) -$
$s2\, (-6\, t22^{3}\, \alpha1\, \alpha2 + t22^{2}\, (-9\, \alpha1 + 10\, \alpha2) - (dd1 - 2\, dd2)$
$(\alpha1 + \alpha2 + \alpha1\, \alpha2) + t12\, (-4 + (-2 + dd2 - 6\, t22)\, \alpha2 +$
$\alpha1\, (-3 + dd2 + 5\, t22 - 2\, \alpha2 + dd2\, \alpha2 + 4\, t22^{2}\, \alpha2)) + t22$
$(10 + (4 - dd1 + dd2)\, \alpha2 + \alpha1\, (7 + dd2 + 4\, \alpha2 + dd2\, \alpha2 - dd1\, (1 + \alpha2)))) -$
$s2^{2}\, (t22\, (-6\, t22^{2}\, (\alpha1 - \alpha2 + \alpha1\, \alpha2) - (dd1 - dd2)\, (\alpha1 + \alpha2 + \alpha1\, \alpha2) +$
$t22\, (14 + 5\, \alpha1 + 10\, \alpha2 + 5\, \alpha1\, \alpha2)) + t12\, (4\, t22^{2}\, (\alpha1 - \alpha2 + \alpha1\, \alpha2) +$
$dd2\, (\alpha1 + \alpha2 + \alpha1\, \alpha2) - t22\, (8 + 6\, \alpha2 + 3\, \alpha1\, (1 + \alpha2)))))) +$
$p12\, (2 + 2\, \alpha1 + dd2\, \alpha1 - t12\, \alpha1 + p22\, t12\, \alpha1 - t22\, \alpha1 - p22\, t22\, \alpha1 + dd2\, \alpha2 +$
$p22\, t12\, \alpha2 + 2\, t22\, \alpha2 - p22\, t22\, \alpha2 + dd2\, \alpha1\, \alpha2 + p22\, t12\, \alpha1\, \alpha2 + 2\, t22\, \alpha1\, \alpha2 -$
$p22\, t22\, \alpha1\, \alpha2 - t12\, t22\, \alpha1\, \alpha2 - t22^{2}\, \alpha1\, \alpha2 + 2\, s2^{3}\, t12\, t22^{2}\, (1 + \alpha2) +$
$s2\, (dd2\, (\alpha1 + \alpha2 + \alpha1\, \alpha2) - t22^{2}\, ((-2 + p22)\, \alpha2 + \alpha1\, (1 + p22 + p22\, \alpha2)) - t22$
$(-4 + (-2 + dd2 + p22)\, \alpha2 + \alpha1\, (-3 + dd2 + p22 - 2\, \alpha2 + dd2\, \alpha2 + p22\, \alpha2)) +$
$t12\, (2 + 2\, dd2\, \alpha2 + p22\, \alpha2 + 2\, t22\, \alpha2 + p22\, t22\, \alpha2 +$
$\alpha1\, (1 - 3\, t22 - 2\, t22^{2}\, \alpha2 + 2\, dd2\, (1 + \alpha2) + p22\, (1 + t22)\, (1 + \alpha2)))) +$
$s2^{2}\, (-t22\, (dd2\, (\alpha1 + \alpha2 + \alpha1\, \alpha2) + t22\, (-2 + (-2 + p22)\, \alpha2 + (-1 + p22)$
$\alpha1\, (1 + \alpha2))) + t12\, (-2\, t22^{2}\, (\alpha1 - \alpha2 + \alpha1\, \alpha2) + 2\, dd2$
$(\alpha1 + \alpha2 + \alpha1\, \alpha2) + t22\, (4 + (2 + p22)\, \alpha2 + (1 + p22)\, \alpha1\, (1 + \alpha2)))))) +$
$m2^{2}\, (4\, dd1\, s2^{2}\, t12 - 4\, dd2\, s2^{2}\, t12 + 2\, dd2\, s2^{3}\, t12^{2} - 4\, dd1\, s2^{2}\, t22 +$
$4\, dd2\, s2^{2}\, t22 + 4\, dd1\, s2^{3}\, t12\, t22 -$
$8\, dd2\, s2^{3}\, t12\, t22 - 4\, dd1\, s2^{3}\, t22^{2} +$
$6\, dd2\, s2^{3}\, t22^{2} + dd1^{2}\, s2\, \alpha1 - 2\, dd1\, dd2\, s2\, \alpha1 +$
$dd2^{2}\, s2\, \alpha1 + dd1^{2}\, s2^{2}\, \alpha1 - 2\, dd1\, dd2\, s2^{2}\, \alpha1 +$
$dd2^{2}\, s2^{2}\, \alpha1 - 3\, dd1\, s2\, t12\, \alpha1 + 3\, dd2\, s2\, t12\, \alpha1 +$
$dd1\, s2^{2}\, t12\, \alpha1 - dd2\, s2^{2}\, t12\, \alpha1 -$
$2\, dd2\, s2^{2}\, t12^{2}\, \alpha1 + 3\, dd1\, s2\, t22\, \alpha1 -$
$3\, dd2\, s2\, t22\, \alpha1 - dd1\, s2^{2}\, t22\, \alpha1 +$
$dd2\, s2^{2}\, t22\, \alpha1 - 4\, dd1\, s2^{2}\, t12\, t22\, \alpha1 +$
$8\, dd2\, s2^{2}\, t12\, t22\, \alpha1 + 4\, dd1\, s2^{2}\, t22^{2}\, \alpha1 -$
$6\, dd2\, s2^{2}\, t22^{2}\, \alpha1 + dd1^{2}\, s2\, \alpha2 -$
$2\, dd1\, dd2\, s2\, \alpha2 + dd2^{2}\, s2\, \alpha2 + dd1^{2}\, s2^{2}\, \alpha2 -$
$2\, dd1\, dd2\, s2^{2}\, \alpha2 + dd2^{2}\, s2^{2}\, \alpha2 + 2\, dd1\, s2\, t12\, \alpha2 -$
$2\, dd2\, s2\, t12\, \alpha2 + 2\, dd1\, s2^{2}\, t12\, \alpha2 -$
$2\, dd2\, s2^{2}\, t12\, \alpha2 + 2\, dd2\, s2^{2}\, t12^{2}\, \alpha2 +$
$2\, dd2\, s2^{3}\, t12^{2}\, \alpha2 - 2\, dd1\, s2\, t22\, \alpha2 +$

```
2 dd2 s2 t22 α2 - 2 dd1 s2^2 t22 α2 +
2 dd2 s2^2 t22 α2 + 4 dd1 s2^2 t12 t22 α2 -
8 dd2 s2^2 t12 t22 α2 + 4 dd1 s2^3 t12 t22 α2 -
8 dd2 s2^3 t12 t22 α2 - 4 dd1 s2^2 t22^2 α2 +
6 dd2 s2^2 t22^2 α2 - 4 dd1 s2^3 t22^2 α2 +
6 dd2 s2^3 t22^2 α2 + dd1^2 s2 α1 α2 -
2 dd1 dd2 s2 α1 α2 + dd2^2 s2 α1 α2 +
dd1^2 s2^2 α1 α2 - 2 dd1 dd2 s2^2 α1 α2 +
dd2^2 s2^2 α1 α2 - dd1 t12 α1 α2 + dd2 t12 α1 α2 +
dd1 s2^2 t12 α1 α2 - dd2 s2^2 t12 α1 α2 -
2 dd2 s2 t12^2 α1 α2 - 2 dd2 s2^2 t12^2 α1 α2 +
dd1 t22 α1 α2 - dd2 t22 α1 α2 -
dd1 s2^2 t22 α1 α2 + dd2 s2^2 t22 α1 α2 -
4 dd1 s2 t12 t22 α1 α2 + 8 dd2 s2 t12 t22 α1 α2 -
4 dd1 s2^2 t12 t22 α1 α2 + 8 dd2 s2^2 t12 t22 α1 α2 +
4 dd1 s2 t22^2 α1 α2 - 6 dd2 s2 t22^2 α1 α2 +
4 dd1 s2^2 t22^2 α1 α2 - 6 dd2 s2^2 t22^2 α1 α2 +
p12^2 (1 + s2) (1 + s2 t12) (t12 - t22) (α1 + α2 + α1 α2) +
2 p22^2 (1 + s2) (t12 - t22) (1 + s2 t22) (α1 + α2 + α1 α2) +
p22 (- dd1 α1 + dd2 α1 + t12 α1 - t22 α1 - dd1 α2 + dd2 α2 - 2 t12 α2 +
   2 t22 α2 - dd1 α1 α2 + dd2 α1 α2 - 2 t12 α1 α2 - t12^2 α1 α2 + 2 t22 α1 α2 +
   5 t12 t22 α1 α2 - 4 t22^2 α1 α2 + 2 s2^3 t22 (t12^2 - 4 t12 t22 + 3 t22^2) (1 + α2) -
   s2 (t22^2 (6 α1 - 8 α2) + 6 t22^3 α1 α2 + (dd1 - dd2) (α1 + α2 + α1 α2) +
      t12^2 (α1 - 2 α2 + 2 t22 α1 α2) + t22 (-4 + (-2 + dd2) α2 +
         α1 (-3 + dd2 - 2 α2 + dd2 α2)) + t12 (4 + (2 + dd1 - 2 dd2 + 10 t22) α2 +
         α1 (3 - 7 t22 + 2 α2 - 8 t22^2 α2 + dd1 (1 + α2) - 2 dd2 (1 + α2)))) -
   s2^2 (t12^2 ((-1 + 2 t22) α1 (1 + α2) - 2 (1 + α2 + t22 α2)) + t12
        (-8 t22^2 (α1 - α2 + α1 α2) + (dd1 - 2 dd2) (α1 + α2 + α1 α2) +
         t22 (12 + 5 α1 + 10 α2 + 5 α1 α2)) + t22 (6 t22^2 (α1 - α2 + α1 α2) +
         dd2 (α1 + α2 + α1 α2) - 2 t22 (5 + 4 α2 + 2 α1 (1 + α2))))) +
p12 (dd1 α1 - dd2 α1 - t12 α1 - 3 p22 t12 α1 + t22 α1 + 3 p22 t22 α1 +
   dd1 α2 - dd2 α2 + 2 t12 α2 - 3 p22 t12 α2 - 2 t22 α2 + 3 p22 t22 α2 +
   dd1 α1 α2 - dd2 α1 α2 + 2 t12 α1 α2 - 3 p22 t12 α1 α2 - t12^2 α1 α2 -
   2 t22 α1 α2 + 3 p22 t22 α1 α2 - t12 t22 α1 α2 + 2 t22^2 α1 α2 +
   4 s2^3 t12 (t12 - t22) t22 (1 + α2) - s2 (- (dd1 - dd2) (α1 + α2 + α1 α2) - 2
        t22^2 ((-2 + p22) α2 + α1 (1 + p22 + p22 α2)) + t12^2 ((-2 + p22) α2 +
         α1 (3 + p22 + p22 α2 + 4 t22 α2)) + t22 (4 + (2 + dd1 - 2 dd2 - 3 p22) α2 +
         α1 (3 - 3 p22 + 2 α2 - 3 p22 α2 + dd1 (1 + α2) - 2 dd2 (1 + α2))) +
      t12 (-4 + (-2 - 2 dd1 + 3 dd2 + 3 p22 - 2 t22 + p22 t22) α2 +
         α1 (-3 + 3 p22 - t22 + p22 t22 - 2 α2 + 3 p22 α2 + p22 t22 α2 -
            4 t22^2 α2 - 2 dd1 (1 + α2) + 3 dd2 (1 + α2)))) -
   s2^2 (t12^2 (-4 + (-2 + p22 - 4 t22) α2 + (-1 + p22 + 4 t22) α1 (1 + α2)) + t22
        ((dd1 - 2 dd2) (α1 + α2 + α1 α2) - 2 t22 (-2 + (-2 + p22) α2 + (-1 + p22) α1
              (1 + α2))) + t12 (-2 dd1 (α1 + α2 + α1 α2) + 3 dd2 (α1 + α2 + α1 α2) +
         t22 ((-2 + p22 + 4 t22) α2 + (-1 + p22 - 4 t22) α1 (1 + α2)))))) /
(2 (1 + m2 s2 (t12 - t22) + s2 t22) (1 + s2 t22 + m2 (t12 - t22) (s2 - α1) +
    α1 - t22 α1)
  (1 + t22 α2 + s2 t22 (1 + α2) + m2 (t12 - t22) (s2 + α2 + s2 α2))),
((1 + s2) (m2 (t12 - t22) + t22)
   (2 + α1 + p22 α1 -
     t22 α1 - p22 t22 α1 +
     p22 α2 + 2 t22 α2 -
```

```
p22 t22 α2 +
p22 α1 α2 + t22 α1 α2 -
p22 t22 α1 α2 -
t22^2 α1 α2 +
2 s2^2 t22^2 (1 + α2) +
s2 (dd2 (α1 + α2 + α1 α2) + t22 (4 + (2 - dd2 + p22) α2 - (-1 + dd2 - p22) α1 (1 + α2)) -
    t22^2 ((-2 + p22) α2 + (1 + p22) α1 (1 + α2))) +
m2^2 (t12 - t22) (p22 α1 + p22 α2 + p22 α1 α2 - t12 α1 α2 + t22 α1 α2 +
    2 s2^2 (t12 - t22) (1 + α2) - p12 (α1 + α2 + α1 α2) +
    s2 (-t12 α1 - p12 t12 α1 + t22 α1 + p22 t22 α1 + 2 t12 α2 - p12 t12 α2 -
        2 t22 α2 + p22 t22 α2 - t12 α1 α2 - p12 t12 α1 α2 + t22 α1 α2 +
        p22 t22 α1 α2 - dd1 (α1 + α2 + α1 α2) + dd2 (α1 + α2 + α1 α2))) +
m2 (p12 α1 - p22 α1 - t12 α1 - p22 t12 α1 + t22 α1 - p12 t22 α1 + 2 p22 t22 α1 +
    p12 α2 - p22 α2 + 2 t12 α2 - p22 t12 α2 - 2 t22 α2 -
    p12 t22 α2 + 2 p22 t22 α2 + p12 α1 α2 - p22 α1 α2 + t12 α1 α2 -
    p22 t12 α1 α2 - t22 α1 α2 - p12 t22 α1 α2 + 2 p22 t22 α1 α2 -
    2 t12 t22 α1 α2 + 2 t22^2 α1 α2 + 4 s2^2 (t12 - t22) t22 (1 + α2) - s2
     (-(dd1 - dd2) (α1 + α2 + α1 α2) - 2 t22^2 ((-2 + p22) α2 + (1 + p22) α1 (1 + α2)) +
        t22 (4 + (2 + dd1 - 2 dd2 + p22) α2 + (1 + dd1 - 2 dd2 + p22) α1 (1 + α2)) +
        t12 (-4 + (-2 + dd2 + p12 (-1 + t22) - 4 t22 + p22 t22) α2 +
            (-1 + dd2 + p12 (-1 + t22) + 2 t22 + p22 t22) α1 (1 + α2)))))) /
  (2 (1 + m2 s2 (t12 - t22) + s2 t22) (1 + s2 t22 + m2 (t12 - t22) (s2 - α1) +
       α1 - t22 α1)
     (1 + t22 α2 + s2 t22 (1 + α2) + m2 (t12 - t22) (s2 + α2 + s2 α2))),
 -(((1 + s2) (1 + m2 s2 ((1 - p12) t12 + p12 t12 - (1 - p22) t22 - p22 t22) +
            s2 ((1 - p22) t22 + p22 t22))^2
      ((r (1 + s2) (dd2 + m2 (dd1 - dd2 + (1 - p12) (1 - t12) - (1 - p22) (1 - t22)) +
                 (1 - p22) (1 - t22)) (dd2 + p22 t22 + m2 (dd1 - dd2 + p12 t12 - p22 t22))) /
           (-dd2 + p22 (1 - t22) + (1 - p22) t22 + p22 t22 + s2 (-dd2 + (1 - p22) t22) +
              s2 (dd2 + p22 t22) + (-dd2 + p22 (1 - t22)) α1 + (dd2 + (1 - p22) (1 - t22))
               (1 + α1) + m2 (-dd1 + p12 (1 - t12) + (1 - p12) t12 + p12 t12 +
                  s2 (-dd1 + (1 - p12) t12) + s2 (dd1 + p12 t12) - (1 - p22) (1 - t22) -
                  p22 (1 - t22) - (1 - p22) t22 - p22 t22 - s2 (-dd2 + (1 - p22) t22) - s2
                   (dd2 + p22 t22) + (-dd1 + p12 (1 - t12)) α1 - (dd2 + (1 - p22) (1 - t22))
                   α1 - (-dd2 + p22 (1 - t22)) α1 + (dd1 + (1 - p12) (1 - t12)) (1 + α1))) +
         ((dd2 + m2 (dd1 - dd2 + (1 - p12) (1 - t12) - (1 - p22) (1 - t22)) + (1 - p22)
                (1 - t22)) (-dd2 + m2 (-dd1 + dd2 + p12 (1 - t12) - p22 (1 - t22)) +
               p22 (1 - t22)) (1 + α1)) / (-dd2 + p22 (1 - t22) + (1 - p22) t22 +
              p22 t22 + s2 (-dd2 + (1 - p22) t22) + s2 (dd2 + p22 t22) +
              (-dd2 + p22 (1 - t22)) α1 + (dd2 + (1 - p22) (1 - t22)) (1 + α1) +
              m2 (-dd1 + p12 (1 - t12) + (1 - p12) t12 + p12 t12 + s2 (-dd1 + (1 - p12) t12) +
                  s2 (dd1 + p12 t12) - (1 - p22) (1 - t22) - p22 (1 - t22) - (1 - p22) t22 -
                  p22 t22 - s2 (-dd2 + (1 - p22) t22) - s2 (dd2 + p22 t22) +
                  (-dd1 + p12 (1 - t12)) α1 - (dd2 + (1 - p22) (1 - t22)) α1 -
                  (-dd2 + p22 (1 - t22)) α1 + (dd1 + (1 - p12) (1 - t12)) (1 + α1))) -
         ((-1 + r) (1 + s2) (-dd2 + m2 (-dd1 + dd2 + p12 (1 - t12) - p22 (1 - t22)) +
               p22 (1 - t22)) (-dd2 + (1 - p22) t22 +
               m2 (-dd1 + dd2 + (1 - p12) t12 - (1 - p22) t22)) (1 + α1)) /
           (-dd2 + p22 (1 - t22) + (1 - p22) t22 + p22 t22 + s2 (-dd2 + (1 - p22) t22) +
              s2 (dd2 + p22 t22) + (-dd2 + p22 (1 - t22)) α1 + (dd2 + (1 - p22) (1 - t22))
```

```
(1 + α1) + m2 (-dd1 + p12 (1 - t12) + (1 - p12) t12 + p12 t12 +
    s2 (-dd1 + (1 - p12) t12) + s2 (dd1 + p12 t12) - (1 - p22) (1 - t22) -
    p22 (1 - t22) - (1 - p22) t22 - p22 t22 - s2 (-dd2 + (1 - p22) t22) - s2
      (dd2 + p22 t22) + (-dd1 + p12 (1 - t12)) α1 - (dd2 + (1 - p22) (1 - t22))
      α1 - (-dd2 + p22 (1 - t22)) α1 + (dd1 + (1 - p12) (1 - t12)) (1 + α1))) +
((dd2 + m2 (dd1 - dd2 + (1 - p12) (1 - t12) - (1 - p22) (1 - t22)) + (1 - p22)
      (1 - t22)) (-dd2 + m2 (-dd1 + dd2 + p12 (1 - t12) - p22 (1 - t22)) +
    p22 (1 - t22))) / ((1 - p22) (1 - t22) + p22 (1 - t22) +
   (1 - p22) t22 + p22 t22 + s2 (-dd2 + (1 - p22) t22) + s2 (dd2 + p22 t22) +
   (-dd2 + (1 - p22) t22) α2 + s2 (-dd2 + (1 - p22) t22) α2 + (dd2 + p22 t22) α2 +
   s2 (dd2 + p22 t22) α2 + m2 (dd1 + (1 - p12) (1 - t12) + p12 (1 - t12) +
      p12 t12 + s2 (dd1 + p12 t12) - (1 - p22) (1 - t22) - p22 (1 - t22) -
      (1 - p22) t22 - p22 t22 - s2 (-dd2 + (1 - p22) t22) - s2 (dd2 + p22 t22) +
      (dd1 + p12 t12) α2 + s2 (dd1 + p12 t12) α2 - (-dd2 + (1 - p22) t22) α2 -
      s2 (-dd2 + (1 - p22) t22) α2 - (dd2 + p22 t22) α2 -
      s2 (dd2 + p22 t22) α2 + (1 + s2) (-dd1 + (1 - p12) t12) (1 + α2))) +
(2 (-dd2 + m2 (-dd1 + dd2 + p12 (1 - t12) - p22 (1 - t22)) + p22 (1 - t22))^2) /
 ((1 - p22) (1 - t22) + p22 (1 - t22) + (1 - p22) t22 + p22 t22 +
   s2 (-dd2 + (1 - p22) t22) + s2 (dd2 + p22 t22) + (-dd2 + (1 - p22) t22) α2 +
   s2 (-dd2 + (1 - p22) t22) α2 + (dd2 + p22 t22) α2 + s2 (dd2 + p22 t22) α2 +
   m2 (dd1 + (1 - p12) (1 - t12) + p12 (1 - t12) + p12 t12 + s2 (dd1 + p12 t12) -
      (1 - p22) (1 - t22) - p22 (1 - t22) - (1 - p22) t22 - p22 t22 -
      s2 (-dd2 + (1 - p22) t22) - s2 (dd2 + p22 t22) + (dd1 + p12 t12) α2 +
      s2 (dd1 + p12 t12) α2 - (-dd2 + (1 - p22) t22) α2 -
      s2 (-dd2 + (1 - p22) t22) α2 - (dd2 + p22 t22) α2 -
      s2 (dd2 + p22 t22) α2 + (1 + s2) (-dd1 + (1 - p12) t12) (1 + α2))) +
(r (1 + s2) (dd2 + m2 (dd1 - dd2 + (1 - p12) (1 - t12) - (1 - p22) (1 - t22)) +
     (1 - p22) (1 - t22)) (dd2 + p22 t22 + m2 (dd1 - dd2 + p12 t12 - p22 t22))) /
 ((1 - p22) (1 - t22) + p22 (1 - t22) + (1 - p22) t22 + p22 t22 +
   s2 (-dd2 + (1 - p22) t22) + s2 (dd2 + p22 t22) + (-dd2 + (1 - p22) t22) α2 +
   s2 (-dd2 + (1 - p22) t22) α2 + (dd2 + p22 t22) α2 + s2 (dd2 + p22 t22) α2 +
   m2 (dd1 + (1 - p12) (1 - t12) + p12 (1 - t12) + p12 t12 + s2 (dd1 + p12 t12) -
      (1 - p22) (1 - t22) - p22 (1 - t22) - (1 - p22) t22 - p22 t22 -
      s2 (-dd2 + (1 - p22) t22) - s2 (dd2 + p22 t22) + (dd1 + p12 t12) α2 +
      s2 (dd1 + p12 t12) α2 - (-dd2 + (1 - p22) t22) α2 -
      s2 (-dd2 + (1 - p22) t22) α2 - (dd2 + p22 t22) α2 -
      s2 (dd2 + p22 t22) α2 + (1 + s2) (-dd1 + (1 - p12) t12) (1 + α2))) +
((1 + s2) (-dd2 + m2 (-dd1 + dd2 + p12 (1 - t12) - p22 (1 - t22)) +
     p22 (1 - t22)) (dd2 + p22 t22 + m2 (dd1 - dd2 + p12 t12 - p22 t22))) /
 ((1 - p22) (1 - t22) + p22 (1 - t22) + (1 - p22) t22 + p22 t22 +
   s2 (-dd2 + (1 - p22) t22) + s2 (dd2 + p22 t22) + (-dd2 + (1 - p22) t22) α2 +
   s2 (-dd2 + (1 - p22) t22) α2 + (dd2 + p22 t22) α2 + s2 (dd2 + p22 t22) α2 +
   m2 (dd1 + (1 - p12) (1 - t12) + p12 (1 - t12) + p12 t12 + s2 (dd1 + p12 t12) -
      (1 - p22) (1 - t22) - p22 (1 - t22) - (1 - p22) t22 - p22 t22 -
      s2 (-dd2 + (1 - p22) t22) - s2 (dd2 + p22 t22) + (dd1 + p12 t12) α2 +
      s2 (dd1 + p12 t12) α2 - (-dd2 + (1 - p22) t22) α2 -
      s2 (-dd2 + (1 - p22) t22) α2 - (dd2 + p22 t22) α2 -
      s2 (dd2 + p22 t22) α2 + (1 + s2) (-dd1 + (1 - p12) t12) (1 + α2))) -
((-1 + r) (1 + s2) (-dd2 + m2 (-dd1 + dd2 + p12 (1 - t12) - p22 (1 - t22)) +
     p22 (1 - t22)) (-dd2 + (1 - p22) t22 +
```

```
m2 (-dd1 + dd2 + (1 - p12) t12 - (1 - p22) t22)) (1 + α2)) /
((1 - p22) (1 - t22) + p22 (1 - t22) + (1 - p22) t22 + p22 t22 +
s2 (-dd2 + (1 - p22) t22) + s2 (dd2 + p22 t22) + (-dd2 + (1 - p22) t22) α2 +
s2 (-dd2 + (1 - p22) t22) α2 + (dd2 + p22 t22) α2 + s2 (dd2 + p22 t22) α2 +
m2 (dd1 + (1 - p12) (1 - t12) + p12 (1 - t12) + p12 t12 + s2 (dd1 + p12 t12) -
(1 - p22) (1 - t22) - p22 (1 - t22) - (1 - p22) t22 - p22 t22 -
s2 (-dd2 + (1 - p22) t22) - s2 (dd2 + p22 t22) + (dd1 + p12 t12) α2 +
s2 (dd1 + p12 t12) α2 - (-dd2 + (1 - p22) t22) α2 -
s2 (-dd2 + (1 - p22) t22) α2 - (dd2 + p22 t22) α2 -
s2 (dd2 + p22 t22) α2 + (1 + s2) (-dd1 + (1 - p12) t12) (1 + α2))) +
((1 + s2) (-dd2 + m2 (-dd1 + dd2 + p12 (1 - t12) - p22 (1 - t22)) + p22 (1 - t22))
(dd2 + p22 t22 + m2 (dd1 - dd2 + p12 t12 - p22 t22)) (1 + α2)) /
((1 - p22) (1 - t22) + p22 (1 - t22) + (1 - p22) t22 + p22 t22 +
s2 (-dd2 + (1 - p22) t22) + s2 (dd2 + p22 t22) + (-dd2 + (1 - p22) t22) α2 +
s2 (-dd2 + (1 - p22) t22) α2 + (dd2 + p22 t22) α2 + s2 (dd2 + p22 t22) α2 +
m2 (dd1 + (1 - p12) (1 - t12) + p12 (1 - t12) + p12 t12 + s2 (dd1 + p12 t12) -
(1 - p22) (1 - t22) - p22 (1 - t22) - (1 - p22) t22 - p22 t22 -
s2 (-dd2 + (1 - p22) t22) - s2 (dd2 + p22 t22) + (dd1 + p12 t12) α2 +
s2 (dd1 + p12 t12) α2 - (-dd2 + (1 - p22) t22) α2 -
s2 (-dd2 + (1 - p22) t22) α2 - (dd2 + p22 t22) α2 -
s2 (dd2 + p22 t22) α2 + (1 + s2) (-dd1 + (1 - p12) t12) (1 + α2))))
(((dd2 + m2 (dd1 - dd2 + (1 - p12) (1 - t12) - (1 - p22) (1 - t22)) +
(1 - p22) (1 - t22)) (-dd2 + (1 - p22) t22 +
m2 (-dd1 + dd2 + (1 - p12) t12 - (1 - p22) t22))) / (-dd2 + p22 (1 - t22) +
(1 - p22) t22 + p22 t22 + s2 (-dd2 + (1 - p22) t22) + s2 (dd2 + p22 t22) +
(-dd2 + p22 (1 - t22)) α1 + (dd2 + (1 - p22) (1 - t22)) (1 + α1) +
m2 (-dd1 + p12 (1 - t12) + (1 - p12) t12 + p12 t12 + s2 (-dd1 + (1 - p12) t12) +
s2 (dd1 + p12 t12) - (1 - p22) (1 - t22) - p22 (1 - t22) - (1 - p22) t22 -
p22 t22 - s2 (-dd2 + (1 - p22) t22) - s2 (dd2 + p22 t22) +
(-dd1 + p12 (1 - t12)) α1 - (dd2 + (1 - p22) (1 - t22)) α1 -
(-dd2 + p22 (1 - t22)) α1 + (dd1 + (1 - p12) (1 - t12)) (1 + α1))) +
(2 (1 + s2) (-dd2 + (1 - p22) t22 + m2 (-dd1 + dd2 + (1 - p12) t12 -
(1 - p22) t22))^2) / (-dd2 + p22 (1 - t22) + (1 - p22) t22 +
p22 t22 + s2 (-dd2 + (1 - p22) t22) + s2 (dd2 + p22 t22) +
(-dd2 + p22 (1 - t22)) α1 + (dd2 + (1 - p22) (1 - t22)) (1 + α1) +
m2 (-dd1 + p12 (1 - t12) + (1 - p12) t12 + p12 t12 + s2 (-dd1 + (1 - p12) t12) +
s2 (dd1 + p12 t12) - (1 - p22) (1 - t22) - p22 (1 - t22) - (1 - p22) t22 -
p22 t22 - s2 (-dd2 + (1 - p22) t22) - s2 (dd2 + p22 t22) +
(-dd1 + p12 (1 - t12)) α1 - (dd2 + (1 - p22) (1 - t22)) α1 -
(-dd2 + p22 (1 - t22)) α1 + (dd1 + (1 - p12) (1 - t12)) (1 + α1))) +
(r (dd2 + m2 (dd1 - dd2 + (1 - p12) (1 - t12) - (1 - p22) (1 - t22)) +
(1 - p22) (1 - t22)) (dd2 + p22 t22 + m2 (dd1 - dd2 + p12 t12 - p22 t22))) /
(-dd2 + p22 (1 - t22) + (1 - p22) t22 + p22 t22 + s2 (-dd2 + (1 - p22) t22) +
s2 (dd2 + p22 t22) + (-dd2 + p22 (1 - t22)) α1 + (dd2 + (1 - p22) (1 - t22))
(1 + α1) + m2 (-dd1 + p12 (1 - t12) + (1 - p12) t12 + p12 t12 +
s2 (-dd1 + (1 - p12) t12) + s2 (dd1 + p12 t12) - (1 - p22) (1 - t22) -
p22 (1 - t22) - (1 - p22) t22 - p22 t22 - s2 (-dd2 + (1 - p22) t22) - s2
(dd2 + p22 t22) + (-dd1 + p12 (1 - t12)) α1 - (dd2 + (1 - p22) (1 - t22))
α1 - (-dd2 + p22 (1 - t22)) α1 + (dd1 + (1 - p12) (1 - t12)) (1 + α1))) +
```

```
((1 + s2) (-dd2 + (1 - p22) t22 + m2 (-dd1 + dd2 + (1 - p12) t12 - (1 - p22) t22))
    (dd2 + p22 t22 + m2 (dd1 - dd2 + p12 t12 - p22 t22))) / (-dd2 + p22 (1 - t22) +
    (1 - p22) t22 + p22 t22 + s2 (-dd2 + (1 - p22) t22) + s2 (dd2 + p22 t22) +
    (-dd2 + p22 (1 - t22)) α1 + (dd2 + (1 - p22) (1 - t22)) (1 + α1) +
    m2 (-dd1 + p12 (1 - t12) + (1 - p12) t12 + p12 t12 + s2 (-dd1 + (1 - p12) t12) +
       s2 (dd1 + p12 t12) - (1 - p22) (1 - t22) - p22 (1 - t22) - (1 - p22) t22 -
       p22 t22 - s2 (-dd2 + (1 - p22) t22) - s2 (dd2 + p22 t22) +
       (-dd1 + p12 (1 - t12)) α1 - (dd2 + (1 - p22) (1 - t22)) α1 -
       (-dd2 + p22 (1 - t22)) α1 + (dd1 + (1 - p12) (1 - t12)) (1 + α1))) +
((dd2 + m2 (dd1 - dd2 + (1 - p12) (1 - t12) - (1 - p22) (1 - t22)) +
      (1 - p22) (1 - t22)) (-dd2 + (1 - p22) t22 +
      m2 (-dd1 + dd2 + (1 - p12) t12 - (1 - p22) t22)) (1 + α1)) /
  (-dd2 + p22 (1 - t22) + (1 - p22) t22 + p22 t22 + s2 (-dd2 + (1 - p22) t22) +
    s2 (dd2 + p22 t22) + (-dd2 + p22 (1 - t22)) α1 + (dd2 + (1 - p22) (1 - t22))
     (1 + α1) + m2 (-dd1 + p12 (1 - t12) + (1 - p12) t12 + p12 t12 +
       s2 (-dd1 + (1 - p12) t12) + s2 (dd1 + p12 t12) - (1 - p22) (1 - t22) -
       p22 (1 - t22) - (1 - p22) t22 - p22 t22 - s2 (-dd2 + (1 - p22) t22) - s2
        (dd2 + p22 t22) + (-dd1 + p12 (1 - t12)) α1 - (dd2 + (1 - p22) (1 - t22))
        α1 - (-dd2 + p22 (1 - t22)) α1 + (dd1 + (1 - p12) (1 - t12)) (1 + α1))) -
((-1 + r) (-dd2 + m2 (-dd1 + dd2 + p12 (1 - t12) - p22 (1 - t22)) + p22 (1 - t22))
    (-dd2 + (1 - p22) t22 + m2 (-dd1 + dd2 + (1 - p12) t12 - (1 - p22) t22))
    (1 + α1)) / (-dd2 + p22 (1 - t22) + (1 - p22) t22 +
    p22 t22 + s2 (-dd2 + (1 - p22) t22) + s2 (dd2 + p22 t22) +
    (-dd2 + p22 (1 - t22)) α1 + (dd2 + (1 - p22) (1 - t22)) (1 + α1) +
    m2 (-dd1 + p12 (1 - t12) + (1 - p12) t12 + p12 t12 + s2 (-dd1 + (1 - p12) t12) +
       s2 (dd1 + p12 t12) - (1 - p22) (1 - t22) - p22 (1 - t22) - (1 - p22) t22 -
       p22 t22 - s2 (-dd2 + (1 - p22) t22) - s2 (dd2 + p22 t22) +
       (-dd1 + p12 (1 - t12)) α1 - (dd2 + (1 - p22) (1 - t22)) α1 -
       (-dd2 + p22 (1 - t22)) α1 + (dd1 + (1 - p12) (1 - t12)) (1 + α1))) +
(r (dd2 + m2 (dd1 - dd2 + (1 - p12) (1 - t12) - (1 - p22) (1 - t22)) +
      (1 - p22) (1 - t22)) (dd2 + p22 t22 + m2 (dd1 - dd2 + p12 t12 - p22 t22))) /
  ((1 - p22) (1 - t22) + p22 (1 - t22) + (1 - p22) t22 + p22 t22 +
    s2 (-dd2 + (1 - p22) t22) + s2 (dd2 + p22 t22) + (-dd2 + (1 - p22) t22) α2 +
    s2 (-dd2 + (1 - p22) t22) α2 + (dd2 + p22 t22) α2 + s2 (dd2 + p22 t22) α2 +
    m2 (dd1 + (1 - p12) (1 - t12) + p12 (1 - t12) + p12 t12 + s2 (dd1 + p12 t12) -
       (1 - p22) (1 - t22) - p22 (1 - t22) - (1 - p22) t22 - p22 t22 -
       s2 (-dd2 + (1 - p22) t22) - s2 (dd2 + p22 t22) + (dd1 + p12 t12) α2 +
       s2 (dd1 + p12 t12) α2 - (-dd2 + (1 - p22) t22) α2 -
       s2 (-dd2 + (1 - p22) t22) α2 - (dd2 + p22 t22) α2 -
       s2 (dd2 + p22 t22) α2 + (1 + s2) (-dd1 + (1 - p12) t12) (1 + α2))) -
((-1 + r) (-dd2 + m2 (-dd1 + dd2 + p12 (1 - t12) - p22 (1 - t22)) + p22 (1 - t22))
    (-dd2 + (1 - p22) t22 + m2 (-dd1 + dd2 + (1 - p12) t12 - (1 - p22) t22))
    (1 + α2)) / ((1 - p22) (1 - t22) + p22 (1 - t22) + (1 - p22) t22 +
    p22 t22 + s2 (-dd2 + (1 - p22) t22) + s2 (dd2 + p22 t22) +
    (-dd2 + (1 - p22) t22) α2 + s2 (-dd2 + (1 - p22) t22) α2 + (dd2 + p22 t22) α2 +
    s2 (dd2 + p22 t22) α2 + m2 (dd1 + (1 - p12) (1 - t12) + p12 (1 - t12) +
       p12 t12 + s2 (dd1 + p12 t12) - (1 - p22) (1 - t22) - p22 (1 - t22) -
       (1 - p22) t22 - p22 t22 - s2 (-dd2 + (1 - p22) t22) - s2 (dd2 + p22 t22) +
       (dd1 + p12 t12) α2 + s2 (dd1 + p12 t12) α2 - (-dd2 + (1 - p22) t22) α2 -
       s2 (-dd2 + (1 - p22) t22) α2 - (dd2 + p22 t22) α2 -
```

```
            s2 (dd2 + p22 t22) α2 + (1 + s2) (-dd1 + (1 - p12) t12) (1 + α2))) +
      ((1 + s2) (-dd2 + (1 - p22) t22 + m2 (-dd1 + dd2 + (1 - p12) t12 - (1 - p22) t22))
         (dd2 + p22 t22 + m2 (dd1 - dd2 + p12 t12 - p22 t22)) (1 + α2)) /
       ((1 - p22) (1 - t22) + p22 (1 - t22) + (1 - p22) t22 + p22 t22 +
          s2 (-dd2 + (1 - p22) t22) + s2 (dd2 + p22 t22) + (-dd2 + (1 - p22) t22) α2 +
          s2 (-dd2 + (1 - p22) t22) α2 + (dd2 + p22 t22) α2 + s2 (dd2 + p22 t22) α2 +
          m2 (dd1 + (1 - p12) (1 - t12) + p12 (1 - t12) + p12 t12 + s2 (dd1 + p12 t12) -
             (1 - p22) (1 - t22) - p22 (1 - t22) - (1 - p22) t22 - p22 t22 -
             s2 (-dd2 + (1 - p22) t22) - s2 (dd2 + p22 t22) + (dd1 + p12 t12) α2 +
             s2 (dd1 + p12 t12) α2 - (-dd2 + (1 - p22) t22) α2 -
             s2 (-dd2 + (1 - p22) t22) α2 - (dd2 + p22 t22) α2 -
             s2 (dd2 + p22 t22) α2 + (1 + s2) (-dd1 + (1 - p12) t12) (1 + α2))))) /
  (4 (1 + s2 ((1 - p22) t22 + p22 t22 + m2 ((1 - p12) t12 + p12 t12 -
             (1 - p22) t22 - p22 t22)))^4)) +
 ((1 + s2) (1 + m2 s2 ((1 - p12) t12 + p12 t12 - (1 - p22) t22 - p22 t22) +
       s2 ((1 - p22) t22 + p22 t22))^2
    ((((1 + s2) (dd2 + m2 (dd1 - dd2 + (1 - p12) (1 - t12) - (1 - p22) (1 - t22)) +
             (1 - p22) (1 - t22))
          (-dd2 + (1 - p22) t22 + m2 (-dd1 + dd2 + (1 - p12) t12 - (1 - p22) t22))) /
       (-dd2 + p22 (1 - t22) + (1 - p22) t22 + p22 t22 + s2 (-dd2 + (1 - p22) t22) +
          s2 (dd2 + p22 t22) + (-dd2 + p22 (1 - t22)) α1 +
          (dd2 + (1 - p22) (1 - t22)) (1 + α1) +
          m2 (-dd1 + p12 (1 - t12) + (1 - p12) t12 + p12 t12 + s2 (-dd1 + (1 - p12) t12) +
             s2 (dd1 + p12 t12) - (1 - p22) (1 - t22) - p22 (1 - t22) - (1 - p22)
              t22 - p22 t22 - s2 (-dd2 + (1 - p22) t22) - s2 (dd2 + p22 t22) +
             (-dd1 + p12 (1 - t12)) α1 - (dd2 + (1 - p22) (1 - t22)) α1 -
             (-dd2 + p22 (1 - t22)) α1 + (dd1 + (1 - p12) (1 - t12)) (1 + α1))) -
      ((-1 + r) (1 + s2) (dd2 + m2 (dd1 - dd2 + (1 - p12) (1 - t12) - (1 - p22) (1 - t22)) +
             (1 - p22) (1 - t22)) (dd2 + p22 t22 + m2 (dd1 - dd2 + p12 t12 - p22 t22))) /
       (-dd2 + p22 (1 - t22) + (1 - p22) t22 + p22 t22 + s2 (-dd2 + (1 - p22) t22) +
          s2 (dd2 + p22 t22) + (-dd2 + p22 (1 - t22)) α1 +
          (dd2 + (1 - p22) (1 - t22)) (1 + α1) +
          m2 (-dd1 + p12 (1 - t12) + (1 - p12) t12 + p12 t12 + s2 (-dd1 + (1 - p12) t12) +
             s2 (dd1 + p12 t12) - (1 - p22) (1 - t22) - p22 (1 - t22) - (1 - p22)
              t22 - p22 t22 - s2 (-dd2 + (1 - p22) t22) - s2 (dd2 + p22 t22) +
             (-dd1 + p12 (1 - t12)) α1 - (dd2 + (1 - p22) (1 - t22)) α1 -
             (-dd2 + p22 (1 - t22)) α1 + (dd1 + (1 - p12) (1 - t12)) (1 + α1))) +
      (2 (dd2 + m2 (dd1 - dd2 + (1 - p12) (1 - t12) - (1 - p22) (1 - t22)) +
             (1 - p22) (1 - t22))^2 (1 + α1)) /
       (-dd2 + p22 (1 - t22) + (1 - p22) t22 + p22 t22 + s2 (-dd2 + (1 - p22) t22) +
          s2 (dd2 + p22 t22) + (-dd2 + p22 (1 - t22)) α1 +
          (dd2 + (1 - p22) (1 - t22)) (1 + α1) +
          m2 (-dd1 + p12 (1 - t12) + (1 - p12) t12 + p12 t12 + s2 (-dd1 + (1 - p12) t12) +
             s2 (dd1 + p12 t12) - (1 - p22) (1 - t22) - p22 (1 - t22) - (1 - p22)
              t22 - p22 t22 - s2 (-dd2 + (1 - p22) t22) - s2 (dd2 + p22 t22) +
             (-dd1 + p12 (1 - t12)) α1 - (dd2 + (1 - p22) (1 - t22)) α1 -
             (-dd2 + p22 (1 - t22)) α1 + (dd1 + (1 - p12) (1 - t12)) (1 + α1))) +
      ((dd2 + m2 (dd1 - dd2 + (1 - p12) (1 - t12) - (1 - p22) (1 - t22)) +
```

```
        (1 - p22) (1 - t22))
      (-dd2 + m2 (-dd1 + dd2 + p12 (1 - t12) - p22 (1 - t22)) + p22 (1 - t22))
      (1 + α1)) / (-dd2 + p22 (1 - t22) + (1 - p22) t22 + p22 t22 +
      s2 (-dd2 + (1 - p22) t22) + s2 (dd2 + p22 t22) +
      (-dd2 + p22 (1 - t22)) α1 + (dd2 + (1 - p22) (1 - t22)) (1 + α1) +
      m2 (-dd1 + p12 (1 - t12) + (1 - p12) t12 + p12 t12 + s2 (-dd1 + (1 - p12) t12) +
          s2 (dd1 + p12 t12) - (1 - p22) (1 - t22) - p22 (1 - t22) - (1 - p22)
           t22 - p22 t22 - s2 (-dd2 + (1 - p22) t22) - s2 (dd2 + p22 t22) +
          (-dd1 + p12 (1 - t12)) α1 - (dd2 + (1 - p22) (1 - t22)) α1 -
          (-dd2 + p22 (1 - t22)) α1 + (dd1 + (1 - p12) (1 - t12)) (1 + α1))) +
  ((1 + s2) (dd2 + m2 (dd1 - dd2 + (1 - p12) (1 - t12) - (1 - p22) (1 - t22)) +
        (1 - p22) (1 - t22)) (-dd2 + (1 - p22) t22 +
        m2 (-dd1 + dd2 + (1 - p12) t12 - (1 - p22) t22)) (1 + α1)) /
   (-dd2 + p22 (1 - t22) + (1 - p22) t22 + p22 t22 + s2 (-dd2 + (1 - p22) t22) +
      s2 (dd2 + p22 t22) + (-dd2 + p22 (1 - t22)) α1 +
      (dd2 + (1 - p22) (1 - t22)) (1 + α1) +
      m2 (-dd1 + p12 (1 - t12) + (1 - p12) t12 + p12 t12 + s2 (-dd1 + (1 - p12) t12) +
          s2 (dd1 + p12 t12) - (1 - p22) (1 - t22) - p22 (1 - t22) - (1 - p22)
           t22 - p22 t22 - s2 (-dd2 + (1 - p22) t22) - s2 (dd2 + p22 t22) +
          (-dd1 + p12 (1 - t12)) α1 - (dd2 + (1 - p22) (1 - t22)) α1 -
          (-dd2 + p22 (1 - t22)) α1 + (dd1 + (1 - p12) (1 - t12)) (1 + α1))) +
  (r (1 + s2) (-dd2 + m2 (-dd1 + dd2 + p12 (1 - t12) - p22 (1 - t22)) + p22 (1 - t22))
      (-dd2 + (1 - p22) t22 + m2 (-dd1 + dd2 + (1 - p12) t12 - (1 - p22) t22))
      (1 + α1)) / (-dd2 + p22 (1 - t22) + (1 - p22) t22 +
      p22 t22 + s2 (-dd2 + (1 - p22) t22) + s2 (dd2 + p22 t22) +
      (-dd2 + p22 (1 - t22)) α1 + (dd2 + (1 - p22) (1 - t22)) (1 + α1) +
      m2 (-dd1 + p12 (1 - t12) + (1 - p12) t12 + p12 t12 + s2 (-dd1 + (1 - p12) t12) +
          s2 (dd1 + p12 t12) - (1 - p22) (1 - t22) - p22 (1 - t22) - (1 - p22)
           t22 - p22 t22 - s2 (-dd2 + (1 - p22) t22) - s2 (dd2 + p22 t22) +
          (-dd1 + p12 (1 - t12)) α1 - (dd2 + (1 - p22) (1 - t22)) α1 -
          (-dd2 + p22 (1 - t22)) α1 + (dd1 + (1 - p12) (1 - t12)) (1 + α1))) +
  ((dd2 + m2 (dd1 - dd2 + (1 - p12) (1 - t12) - (1 - p22) (1 - t22)) +
        (1 - p22) (1 - t22))
      (-dd2 + m2 (-dd1 + dd2 + p12 (1 - t12) - p22 (1 - t22)) + p22 (1 - t22))) /
   ((1 - p22) (1 - t22) + p22 (1 - t22) + (1 - p22) t22 + p22 t22 +
      s2 (-dd2 + (1 - p22) t22) + s2 (dd2 + p22 t22) + (-dd2 + (1 - p22) t22) α2 +
      s2 (-dd2 + (1 - p22) t22) α2 + (dd2 + p22 t22) α2 + s2 (dd2 + p22 t22) α2 +
      m2 (dd1 + (1 - p12) (1 - t12) + p12 (1 - t12) + p12 t12 + s2
            (dd1 + p12 t12) - (1 - p22) (1 - t22) - p22 (1 - t22) - (1 - p22)
           t22 - p22 t22 - s2 (-dd2 + (1 - p22) t22) - s2 (dd2 + p22 t22) +
          (dd1 + p12 t12) α2 + s2 (dd1 + p12 t12) α2 - (-dd2 + (1 - p22) t22)
           α2 - s2 (-dd2 + (1 - p22) t22) α2 - (dd2 + p22 t22) α2 - s2
            (dd2 + p22 t22) α2 + (1 + s2) (-dd1 + (1 - p12) t12) (1 + α2))) -
  ((-1 + r) (1 + s2) (dd2 + m2 (dd1 - dd2 + (1 - p12) (1 - t12) - (1 - p22) (1 - t22)) +
        (1 - p22) (1 - t22)) (dd2 + p22 t22 + m2 (dd1 - dd2 + p12 t12 - p22 t22))) /
   ((1 - p22) (1 - t22) + p22 (1 - t22) + (1 - p22) t22 + p22 t22 +
      s2 (-dd2 + (1 - p22) t22) + s2 (dd2 + p22 t22) + (-dd2 + (1 - p22) t22) α2 +
      s2 (-dd2 + (1 - p22) t22) α2 + (dd2 + p22 t22) α2 + s2 (dd2 + p22 t22) α2 +
      m2 (dd1 + (1 - p12) (1 - t12) + p12 (1 - t12) + p12 t12 + s2
            (dd1 + p12 t12) - (1 - p22) (1 - t22) - p22 (1 - t22) - (1 - p22)
```

```
t22 - p22 t22 - s2 (-dd2 + (1 - p22) t22) - s2 (dd2 + p22 t22) +
(dd1 + p12 t12) α2 + s2 (dd1 + p12 t12) α2 - (-dd2 + (1 - p22) t22)
α2 - s2 (-dd2 + (1 - p22) t22) α2 - (dd2 + p22 t22) α2 - s2
(dd2 + p22 t22) α2 + (1 + s2) (-dd1 + (1 - p12) t12) (1 + α2))) +
(r (1 + s2) (-dd2 + m2 (-dd1 + dd2 + p12 (1 - t12) - p22 (1 - t22)) + p22 (1 - t22))
(-dd2 + (1 - p22) t22 + m2 (-dd1 + dd2 + (1 - p12) t12 - (1 - p22) t22))
(1 + α2)) / ((1 - p22) (1 - t22) + p22 (1 - t22) + (1 - p22) t22 + p22 t22 +
s2 (-dd2 + (1 - p22) t22) + s2 (dd2 + p22 t22) + (-dd2 + (1 - p22) t22) α2 +
s2 (-dd2 + (1 - p22) t22) α2 + (dd2 + p22 t22) α2 + s2 (dd2 + p22 t22) α2 +
m2 (dd1 + (1 - p12) (1 - t12) + p12 (1 - t12) + p12 t12 + s2
(dd1 + p12 t12) - (1 - p22) (1 - t22) - p22 (1 - t22) - (1 - p22)
t22 - p22 t22 - s2 (-dd2 + (1 - p22) t22) - s2 (dd2 + p22 t22) +
(dd1 + p12 t12) α2 + s2 (dd1 + p12 t12) α2 - (-dd2 + (1 - p22) t22)
α2 - s2 (-dd2 + (1 - p22) t22) α2 - (dd2 + p22 t22) α2 - s2
(dd2 + p22 t22) α2 + (1 + s2) (-dd1 + (1 - p12) t12) (1 + α2))))
(-(((-1 + r) (dd2 + m2 (dd1 - dd2 + (1 - p12) (1 - t12) - (1 - p22) (1 - t22)) +
(1 - p22) (1 - t22)) (dd2 + p22 t22 + m2 (dd1 - dd2 + p12 t12 - p22 t22))) /
(-dd2 + p22 (1 - t22) + (1 - p22) t22 + p22 t22 + s2 (-dd2 + (1 - p22) t22) +
s2 (dd2 + p22 t22) + (-dd2 + p22 (1 - t22)) α1 + (dd2 + (1 - p22) (1 - t22))
(1 + α1) + m2 (-dd1 + p12 (1 - t12) + (1 - p12) t12 + p12 t12 +
s2 (-dd1 + (1 - p12) t12) + s2 (dd1 + p12 t12) - (1 - p22) (1 - t22) -
p22 (1 - t22) - (1 - p22) t22 - p22 t22 - s2 (-dd2 + (1 - p22) t22) - s2
(dd2 + p22 t22) + (-dd1 + p12 (1 - t12)) α1 - (dd2 + (1 - p22) (1 - t22))
α1 - (-dd2 + p22 (1 - t22)) α1 + (dd1 + (1 - p12) (1 - t12)) (1 + α1)))) +
((1 + s2) (-dd2 + (1 - p22) t22 + m2 (-dd1 + dd2 + (1 - p12) t12 - (1 - p22) t22))
(dd2 + p22 t22 + m2 (dd1 - dd2 + p12 t12 - p22 t22))) /
(-dd2 + p22 (1 - t22) + (1 - p22) t22 + p22 t22 + s2 (-dd2 + (1 - p22) t22) +
s2 (dd2 + p22 t22) + (-dd2 + p22 (1 - t22)) α1 +
(dd2 + (1 - p22) (1 - t22)) (1 + α1) +
m2 (-dd1 + p12 (1 - t12) + (1 - p12) t12 + p12 t12 + s2 (-dd1 + (1 - p12) t12) +
s2 (dd1 + p12 t12) - (1 - p22) (1 - t22) - p22 (1 - t22) - (1 - p22)
t22 - p22 t22 - s2 (-dd2 + (1 - p22) t22) - s2 (dd2 + p22 t22) +
(-dd1 + p12 (1 - t12)) α1 - (dd2 + (1 - p22) (1 - t22)) α1 -
(-dd2 + p22 (1 - t22)) α1 + (dd1 + (1 - p12) (1 - t12)) (1 + α1))) +
(r (-dd2 + m2 (-dd1 + dd2 + p12 (1 - t12) - p22 (1 - t22)) + p22 (1 - t22))
(-dd2 + (1 - p22) t22 + m2 (-dd1 + dd2 + (1 - p12) t12 - (1 - p22) t22))
(1 + α1)) / (-dd2 + p22 (1 - t22) + (1 - p22) t22 +
p22 t22 + s2 (-dd2 + (1 - p22) t22) + s2 (dd2 + p22 t22) +
(-dd2 + p22 (1 - t22)) α1 + (dd2 + (1 - p22) (1 - t22)) (1 + α1) +
m2 (-dd1 + p12 (1 - t12) + (1 - p12) t12 + p12 t12 + s2 (-dd1 + (1 - p12) t12) +
s2 (dd1 + p12 t12) - (1 - p22) (1 - t22) - p22 (1 - t22) - (1 - p22)
t22 - p22 t22 - s2 (-dd2 + (1 - p22) t22) - s2 (dd2 + p22 t22) +
(-dd1 + p12 (1 - t12)) α1 - (dd2 + (1 - p22) (1 - t22)) α1 -
(-dd2 + p22 (1 - t22)) α1 + (dd1 + (1 - p12) (1 - t12)) (1 + α1))) -
((-1 + r) (dd2 + m2 (dd1 - dd2 + (1 - p12) (1 - t12) - (1 - p22) (1 - t22)) +
(1 - p22) (1 - t22)) (dd2 + p22 t22 + m2 (dd1 - dd2 + p12 t12 - p22 t22))) /
((1 - p22) (1 - t22) + p22 (1 - t22) + (1 - p22) t22 + p22 t22 +
s2 (-dd2 + (1 - p22) t22) + s2 (dd2 + p22 t22) + (-dd2 + (1 - p22) t22) α2 +
s2 (-dd2 + (1 - p22) t22) α2 + (dd2 + p22 t22) α2 + s2 (dd2 + p22 t22) α2 +
m2 (dd1 + (1 - p12) (1 - t12) + p12 (1 - t12) + p12 t12 + s2
```

```
(dd1 + p12 t12) - (1 - p22) (1 - t22) - p22 (1 - t22) - (1 - p22)
t22 - p22 t22 - s2 (-dd2 + (1 - p22) t22) - s2 (dd2 + p22 t22) +
(dd1 + p12 t12) α2 + s2 (dd1 + p12 t12) α2 - (-dd2 + (1 - p22) t22)
α2 - s2 (-dd2 + (1 - p22) t22) α2 - (dd2 + p22 t22) α2 - s2
(dd2 + p22 t22) α2 + (1 + s2) (-dd1 + (1 - p12) t12) (1 + α2))) +
((-dd2 + m2 (-dd1 + dd2 + p12 (1 - t12) - p22 (1 - t22)) + p22 (1 - t22))
(dd2 + p22 t22 + m2 (dd1 - dd2 + p12 t12 - p22 t22))) /
((1 - p22) (1 - t22) + p22 (1 - t22) + (1 - p22) t22 + p22 t22 +
s2 (-dd2 + (1 - p22) t22) + s2 (dd2 + p22 t22) + (-dd2 + (1 - p22) t22) α2 +
s2 (-dd2 + (1 - p22) t22) α2 + (dd2 + p22 t22) α2 + s2 (dd2 + p22 t22) α2 +
m2 (dd1 + (1 - p12) (1 - t12) + p12 (1 - t12) + p12 t12 + s2
(dd1 + p12 t12) - (1 - p22) (1 - t22) - p22 (1 - t22) - (1 - p22)
t22 - p22 t22 - s2 (-dd2 + (1 - p22) t22) - s2 (dd2 + p22 t22) +
(dd1 + p12 t12) α2 + s2 (dd1 + p12 t12) α2 - (-dd2 + (1 - p22) t22)
α2 - s2 (-dd2 + (1 - p22) t22) α2 - (dd2 + p22 t22) α2 - s2
(dd2 + p22 t22) α2 + (1 + s2) (-dd1 + (1 - p12) t12) (1 + α2))) +
(r (-dd2 + m2 (-dd1 + dd2 + p12 (1 - t12) - p22 (1 - t22)) + p22 (1 - t22))
(-dd2 + (1 - p22) t22 + m2 (-dd1 + dd2 + (1 - p12) t12 - (1 - p22) t22))
(1 + α2)) / ((1 - p22) (1 - t22) + p22 (1 - t22) + (1 - p22) t22 + p22 t22 +
s2 (-dd2 + (1 - p22) t22) + s2 (dd2 + p22 t22) + (-dd2 + (1 - p22) t22) α2 +
s2 (-dd2 + (1 - p22) t22) α2 + (dd2 + p22 t22) α2 + s2 (dd2 + p22 t22) α2 +
m2 (dd1 + (1 - p12) (1 - t12) + p12 (1 - t12) + p12 t12 + s2
(dd1 + p12 t12) - (1 - p22) (1 - t22) - p22 (1 - t22) - (1 - p22)
t22 - p22 t22 - s2 (-dd2 + (1 - p22) t22) - s2 (dd2 + p22 t22) +
(dd1 + p12 t12) α2 + s2 (dd1 + p12 t12) α2 - (-dd2 + (1 - p22) t22)
α2 - s2 (-dd2 + (1 - p22) t22) α2 - (dd2 + p22 t22) α2 - s2
(dd2 + p22 t22) α2 + (1 + s2) (-dd1 + (1 - p12) t12) (1 + α2))) +
((-dd2 + m2 (-dd1 + dd2 + p12 (1 - t12) - p22 (1 - t22)) + p22 (1 - t22))
(dd2 + p22 t22 + m2 (dd1 - dd2 + p12 t12 - p22 t22)) (1 + α2)) /
((1 - p22) (1 - t22) + p22 (1 - t22) + (1 - p22) t22 + p22 t22 +
s2 (-dd2 + (1 - p22) t22) + s2 (dd2 + p22 t22) + (-dd2 + (1 - p22) t22) α2 +
s2 (-dd2 + (1 - p22) t22) α2 + (dd2 + p22 t22) α2 + s2 (dd2 + p22 t22) α2 +
m2 (dd1 + (1 - p12) (1 - t12) + p12 (1 - t12) + p12 t12 + s2
(dd1 + p12 t12) - (1 - p22) (1 - t22) - p22 (1 - t22) - (1 - p22)
t22 - p22 t22 - s2 (-dd2 + (1 - p22) t22) - s2 (dd2 + p22 t22) +
(dd1 + p12 t12) α2 + s2 (dd1 + p12 t12) α2 - (-dd2 + (1 - p22) t22)
α2 - s2 (-dd2 + (1 - p22) t22) α2 - (dd2 + p22 t22) α2 - s2
(dd2 + p22 t22) α2 + (1 + s2) (-dd1 + (1 - p12) t12) (1 + α2))) +
((1 + s2) (-dd2 + (1 - p22) t22 + m2 (-dd1 + dd2 + (1 - p12) t12 - (1 - p22) t22))
(dd2 + p22 t22 + m2 (dd1 - dd2 + p12 t12 - p22 t22)) (1 + α2)) /
((1 - p22) (1 - t22) + p22 (1 - t22) + (1 - p22) t22 + p22 t22 +
s2 (-dd2 + (1 - p22) t22) + s2 (dd2 + p22 t22) + (-dd2 + (1 - p22) t22) α2 +
s2 (-dd2 + (1 - p22) t22) α2 + (dd2 + p22 t22) α2 + s2 (dd2 + p22 t22) α2 +
m2 (dd1 + (1 - p12) (1 - t12) + p12 (1 - t12) + p12 t12 + s2
(dd1 + p12 t12) - (1 - p22) (1 - t22) - p22 (1 - t22) - (1 - p22)
t22 - p22 t22 - s2 (-dd2 + (1 - p22) t22) - s2 (dd2 + p22 t22) +
(dd1 + p12 t12) α2 + s2 (dd1 + p12 t12) α2 - (-dd2 + (1 - p22) t22)
α2 - s2 (-dd2 + (1 - p22) t22) α2 - (dd2 + p22 t22) α2 - s2
(dd2 + p22 t22) α2 + (1 + s2) (-dd1 + (1 - p12) t12) (1 + α2))) +
(2 (1 + s2) (dd2 + p22 t22 + m2 (dd1 - dd2 + p12 t12 - p22 t22))^2 (1 + α2)) /
```

```
((1 - p22) (1 - t22) + p22 (1 - t22) + (1 - p22) t22 + p22 t22 +
  s2 (-dd2 + (1 - p22) t22) + s2 (dd2 + p22 t22) + (-dd2 + (1 - p22) t22) α2 +
  s2 (-dd2 + (1 - p22) t22) α2 + (dd2 + p22 t22) α2 + s2 (dd2 + p22 t22) α2 +
  m2 (dd1 + (1 - p12) (1 - t12) + p12 (1 - t12) + p12 t12 + s2
      (dd1 + p12 t12) - (1 - p22) (1 - t22) - p22 (1 - t22) - (1 - p22)
      t22 - p22 t22 - s2 (-dd2 + (1 - p22) t22) - s2 (dd2 + p22 t22) +
     (dd1 + p12 t12) α2 + s2 (dd1 + p12 t12) α2 - (-dd2 + (1 - p22) t22)
      α2 - s2 (-dd2 + (1 - p22) t22) α2 - (dd2 + p22 t22) α2 - s2
      (dd2 + p22 t22) α2 + (1 + s2) (-dd1 + (1 - p12) t12) (1 + α2)))) /
(4 (1 + s2 ((1 - p22) t22 + p22 t22 + m2 ((1 - p12) t12 + p12 t12 -
      (1 - p22) t22 - p22 t22)))^4)};
```

```
itgen[.1, .2, 3, 3.5, .1, .1, .5][{.8, .5, .05, .6, .9, .04}]
```

{0.790576, 0.602846, 0.0474188, 0.621487, 0.865297, 0.0358771}

1.2.3 Computing the equilibrium from itgen using FindRoot

```
Fritgen[s1_, s2_, α1_, α2_, m1_, m2_, r_][
  {fp12_, ft12_, fdd1_, fp22_, ft22_, fdd2_}] :=
 FindRoot[itgen[s1, s2, α1, α2, m1, m2, r][{p12, t12, dd1, p22, t22, dd2}] ==
   {p12, t12, dd1, p22, t22, dd2},
  {{p12, fp12}, {t12, ft12}, {dd1, fdd1}, {p22, fp22}, {t22, ft22}, {dd2, fdd2}}]
```

```
testdif[n_][x_, y_] := If[Max[Abs[x - y]] < 10.^(-n), False, True]
```

Because there exists a curve of equilibria, the above procedure is of limited use and sometimes has convergence problems

2. Analysis

Under the assumption of symmetry of the two populations, this models was analyzed in Servedio and Bürger (2014)

2.1 Equilibria with fixation of one allele

It is easy to show that if dd1 = dd2=0 and p12 = p22, then only t12 = t22 = 0 and t12 = t22 = 1 are possible equilibria

2.1.1 Determination of equilibria

Assume t12 = t22 = 0 (loss of trait allele T2)

```
Simplify[itgen[s1, s2, α1, α2, m1, m2, r][{p12, 0, 0, p22, 0, 0}] - {p12, 0, 0, p22, 0, 0}]
```

{m1 (-p12 + p22), 0, 0, m2 (p12 - p22), 0, 0}

If m1 > 0 or m2 > 0, then all equilibria on this edge satisfy p12 = p22 (and dd1=dd2=0).

Assume t12 = t22 = 1

```
Simplify[itgen[s1, s2, α1, α2, m1, m2, r][{p12, 1, 0, p22, 1, 0}] - {p12, 1, 0, p22, 1, 0}]
```

```
{m1 (-p12 + p22), 0, 0, m2 (p12 - p22), 0, 0}
```

If m1 > 0 or m2 > 0, then all equilibria on this edge satisfy p12 = p22 (and dd1=dd2=0).

Assume p12 = p22 = 0

```
Factor[itgen[s1, s2, α1, α2, m1, m2, r][{0, t12, 0, 0, t22, 0}] - {0, t12, 0, 0, t22, 0}]
```

```
{0, -((2 m1 t12 + 2 s1 t12 + 2 m1 s1 t12 + 2 s1^2 t12 - 2 s1 t12^2 + 2 m1^2 s1 t12^2 - 4 s1^2 t12^2 +
      4 m1 s1^2 t12^2 + 2 s1^2 t12^3 - 4 m1 s1^2 t12^3 + 2 m1^2 s1^2 t12^3 - 2 m1 t22 - 2 m1 s1 t22 -
      4 m1^2 s1 t12 t22 - 4 m1 s1^2 t12 t22 + 4 m1 s1^2 t12^2 t22 - 4 m1^2 s1^2 t12^2 t22 +
      2 m1^2 s1 t22^2 + 2 m1^2 s1^2 t12 t22^2 + t12 α1 + m1 t12 α1 + 3 s1 t12 α1 + m1 s1 t12 α1 +
      2 s1^2 t12 α1 - t12^2 α1 + m1^2 t12^2 α1 - 5 s1 t12^2 α1 + 4 m1 s1 t12^2 α1 + m1^2 s1 t12^2 α1 -
      4 s1^2 t12^2 α1 + 4 m1 s1^2 t12^2 α1 + 2 s1 t12^3 α1 - 4 m1 s1 t12^3 α1 + 2 m1^2 s1 t12^3 α1 +
      2 s1^2 t12^3 α1 - 4 m1 s1^2 t12^3 α1 + 2 m1^2 s1^2 t12^3 α1 - m1 t22 α1 - m1 s1 t22 α1 -
      2 m1^2 t12 t22 α1 - 4 m1 s1 t12 t22 α1 - 2 m1^2 s1 t12 t22 α1 - 4 m1 s1^2 t12 t22 α1 +
      4 m1 s1 t12^2 t22 α1 - 4 m1^2 s1 t12^2 t22 α1 + 4 m1 s1^2 t12^2 t22 α1 - 4 m1^2 s1^2 t12^2 t22 α1 +
      m1^2 t22^2 α1 + m1^2 s1 t22^2 α1 + 2 m1^2 s1 t12 t22^2 α1 + 2 m1^2 s1^2 t12 t22^2 α1) /
    (2 (1 + s1 - s1 t12 + m1 s1 t12 - m1 s1 t22) (1 + s1 - s1 t12 + m1 s1 t12 - m1 s1 t22 +
        α1 + s1 α1 - t12 α1 + m1 t12 α1 - s1 t12 α1 + m1 s1 t12 α1 - m1 t22 α1 - m1 s1 t22 α1))),
 0, 0, -((-2 m2 t12 - 2 m2 s2 t12 - 2 m2^2 s2 t12^2 - 2 m2^2 s2^2 t12^2 + 2 m2 t22 - 2 s2 t22 +
      2 m2 s2 t22 + 4 m2^2 s2 t12 t22 - 4 m2 s2^2 t12 t22 + 4 m2^2 s2^2 t12 t22 + 2 m2^2 s2^2 t12^2 t22 +
      2 s2 t22^2 - 2 m2^2 s2 t22^2 - 2 s2^2 t22^2 + 4 m2 s2^2 t22^2 - 2 m2^2 s2^2 t22^2 + 4 m2 s2^2 t12 t22^2 -
      4 m2^2 s2^2 t12 t22^2 + 2 s2^2 t22^3 - 4 m2 s2^2 t22^3 + 2 m2^2 s2^2 t22^3 - m2 t12 α1 - m2 s2 t12 α1 +
      m2^2 t12^2 α1 + m2^2 s2 t12^2 α1 + t22 α1 + m2 t22 α1 - s2 t22 α1 + m2 s2 t22 α1 - 2 m2^2 t12 t22 α1 +
      4 m2 s2 t12 t22 α1 - 2 m2^2 s2 t12 t22 α1 - 2 m2^2 s2 t12^2 t22 α1 - t22^2 α1 + m2^2 t22^2 α1 +
      3 s2 t22^2 α1 - 4 m2 s2 t22^2 α1 + m2^2 s2 t22^2 α1 - 4 m2 s2 t12 t22^2 α1 + 4 m2^2 s2 t12 t22^2 α1 -
      2 s2 t22^3 α1 + 4 m2 s2 t22^3 α1 - 2 m2^2 s2 t22^3 α1) / (2 (-1 - m2 s2 t12 - s2 t22 + m2 s2 t22)
      (-1 - m2 s2 t12 - s2 t22 + m2 s2 t22 - α1 + m2 t12 α1 + t22 α1 - m2 t22 α1))), 0}
```

```
Simplify[Numerator[Factor[
   itgen[s1, s2, α1, α2, m1, m2, r][{0, t12, 0, 0, t22, 0}] - {0, t12, 0, 0, t22, 0}]]]
```

```
{0, -m1^2 (t12 - t22)^2 (α1 + 2 s1^2 t12 (1 + α1) + s1 (2 + α1 + 2 t12 α1)) -
  (-1 + t12) t12 (-α1 + 2 s1^2 (-1 + t12) (1 + α1) + s1 (-2 + (-3 + 2 t12) α1)) +
  m1 (t12 - t22) (-2 - α1 + 4 s1^2 (-1 + t12) t12 (1 + α1) + s1 (-2 + (-1 - 4 t12 + 4 t12^2) α1)),
 0, 0, -m2 (t12 - t22) (-2 + 4 s2^2 (-1 + t22) t22 - α1 - s2 (2 + (1 - 2 t22)^2 α1)) -
  m2^2 (t12 - t22)^2 (2 s2^2 (-1 + t22) + α1 + s2 (-2 + α1 - 2 t22 α1)) -
  (-1 + t22) t22 (2 s2^2 t22 - α1 + s2 (2 + α1 - 2 t22 α1)), 0}
```

This is independent of $\alpha 2$ (and of r, of course)!

The above may or may not have solutions. If $\alpha 1$ is small, then it usually has (close to migration-selection balance). For large $\alpha 1$, there is typically no solution.

```
NSolve[{-m1^2 (t12 - t22)^2 (α1 + 2 s1^2 t12 (1 + α1) + s1 (2 + α1 + 2 t12 α1)) -
    (-1 + t12) t12 (-α1 + 2 s1^2 (-1 + t12) (1 + α1) + s1 (-2 + (-3 + 2 t12) α1)) +
    m1 (t12 - t22) (-2 - α1 + 4 s1^2 (-1 + t12) t12 (1 + α1) + s1 (-2 + (-1 - 4 t12 + 4 t12^2) α1)),
   -m2 (t12 - t22) (-2 + 4 s2^2 (-1 + t22) t22 - α1 - s2 (2 + (1 - 2 t22)^2 α1)) -
    m2^2 (t12 - t22)^2 (2 s2^2 (-1 + t22) + α1 + s2 (-2 + α1 - 2 t22 α1)) -
    (-1 + t22) t22 (2 s2^2 t22 - α1 + s2 (2 + α1 - 2 t22 α1))} /.
  {s1 → 0.2, s2 → 0.2, m1 → 0.01, m2 → 0.01, α1 → 0.4}, {t12, t22}]
```

```
{{t12 → 4.29625, t22 → -0.67648}, {t12 → 4.27168 + 0.0098123 i, t22 → 1.30515 - 0.790794 i},
 {t12 → 4.27168 - 0.0098123 i, t22 → 1.30515 + 0.790794 i},
 {t12 → 1.04237, t22 → -0.293469}, {t12 → 0.0413652, t22 → 1.6079}, {t12 → 1., t22 → 1.},
 {t12 → 0.0101669, t22 → 0.411472}, {t12 → 0.989981, t22 → 1.29379}, {t12 → 0., t22 → 0.}}
```

```
NSolve[{-m1^2 (t12 - t22)^2 (α1 + 2 s1^2 t12 (1 + α1) + s1 (2 + α1 + 2 t12 α1)) -
    (-1 + t12) t12 (-α1 + 2 s1^2 (-1 + t12) (1 + α1) + s1 (-2 + (-3 + 2 t12) α1)) +
    m1 (t12 - t22) (-2 - α1 + 4 s1^2 (-1 + t12) t12 (1 + α1) + s1 (-2 + (-1 - 4 t12 + 4 t12^2) α1)),
   -m2 (t12 - t22) (-2 + 4 s2^2 (-1 + t22) t22 - α1 - s2 (2 + (1 - 2 t22)^2 α1)) -
    m2^2 (t12 - t22)^2 (2 s2^2 (-1 + t22) + α1 + s2 (-2 + α1 - 2 t22 α1)) -
    (-1 + t22) t22 (2 s2^2 t22 - α1 + s2 (2 + α1 - 2 t22 α1))} /.
  {s1 → 0.2, s2 → 0.2, m1 → 0.01, m2 → 0.01, α1 → 0.5}, {t12, t22}]
```

```
{{t12 → 4.18932, t22 → -0.872673},
 {t12 → 4.16661 + 0.00833685 i, t22 → 0.894128 - 0.648105 i},
 {t12 → 4.16661 - 0.00833685 i, t22 → 0.894128 + 0.648105 i},
 {t12 → 1.04553, t22 → -0.523105}, {t12 → 0.0277905, t22 → 1.20646},
 {t12 → 1.01521, t22 → 0.502458}, {t12 → 1., t22 → 1.},
 {t12 → -0.00456512, t22 → -0.206632}, {t12 → 0., t22 → 0.}}
```

```
NSolve[{-m1^2 (t12 - t22)^2 (α1 + 2 s1^2 t12 (1 + α1) + s1 (2 + α1 + 2 t12 α1)) -
    (-1 + t12) t12 (-α1 + 2 s1^2 (-1 + t12) (1 + α1) + s1 (-2 + (-3 + 2 t12) α1)) +
    m1 (t12 - t22) (-2 - α1 + 4 s1^2 (-1 + t12) t12 (1 + α1) + s1 (-2 + (-1 - 4 t12 + 4 t12^2) α1)),
   -m2 (t12 - t22) (-2 + 4 s2^2 (-1 + t22) t22 - α1 - s2 (2 + (1 - 2 t22)^2 α1)) -
    m2^2 (t12 - t22)^2 (2 s2^2 (-1 + t22) + α1 + s2 (-2 + α1 - 2 t22 α1)) -
    (-1 + t22) t22 (2 s2^2 t22 - α1 + s2 (2 + α1 - 2 t22 α1))} /.
  {s1 → 0.2, s2 → 0.2, m1 → 0.01, m2 → 0.01, α1 → 100}, {t12, t22}]
```

```
NSolve[{-m1^2 (t12 - t22)^2 (α1 + 2 s1^2 t12 (1 + α1) + s1 (2 + α1 + 2 t12 α1)) -
    (-1 + t12) t12 (-α1 + 2 s1^2 (-1 + t12) (1 + α1) + s1 (-2 + (-3 + 2 t12) α1)) +
    m1 (t12 - t22) (-2 - α1 + 4 s1^2 (-1 + t12) t12 (1 + α1) + s1 (-2 + (-1 - 4 t12 + 4 t12^2) α1)),
   -m2 (t12 - t22) (-2 + 4 s2^2 (-1 + t22) t22 - α1 - s2 (2 + (1 - 2 t22)^2 α1)) -
    m2^2 (t12 - t22)^2 (2 s2^2 (-1 + t22) + α1 + s2 (-2 + α1 - 2 t22 α1)) -
    (-1 + t22) t22 (2 s2^2 t22 - α1 + s2 (2 + α1 - 2 t22 α1))} /.
  {s1 → 0.2, s2 → 0.2, m1 → 0.01, m2 → 0.01, α1 → 10000}, {t12, t22}]
```

```
{{t12 → 3.59725, t22 → -2.13778}, {t12 → 3.55978, t22 → 0.0504928},
 {t12 → 3.54386, t22 → 0.974298}, {t12 → 1.03107, t22 → -2.07591},
 {t12 → -0.0144, t22 → -2.05009}, {t12 → 1.00995, t22 → 0.0147497},
 {t12 → 0.00719977, t22 → 1.01003}, {t12 → 1., t22 → 1.}, {t12 → 0., t22 → 0.}}
```

Assume p12 = p22 = 1

Analogous to the case p12 = p22 = 0

2.1.2 Jacobians and stability of equilibria, where one trait allele is fixed

Jacobian at edge t12 = t22 = 0

```
Jacobdt20 =
  Simplify[D[itgen[s1, s2, α1, α2, m1, m2, r][{p12, t12, dd1, p22, t22, dd2}], {{p12,
      t12, dd1, p22, t22, dd2}}] /. {t12 → 0, dd1 → 0, t22 → 0, dd2 → 0, p12 → p22}];
```

An equilibrium is stable if the modulus of eigenvalues of its Jacobian is < 1

```
TableForm[Jacobdt20]
```

$1-m1$	0	$\frac{(-1+m1)\,(2\,s1\,(1+\alpha1)-p22\,\alpha2-\alpha1\,(-1+p22+p22\,\alpha2))}{2\,(1+s1)\,(1+\alpha1)}$	m
0	$-\frac{(-1+m1)\,(2+p22\,\alpha2+\alpha1\,(1+p22+p22\,\alpha2))}{2\,(1+s1)\,(1+\alpha1)}$	0	0
0	$\frac{(-1+m1)\,(-1+p22)\,p22\,r\,(\alpha1+\alpha2+\alpha1\,\alpha2)}{2\,(1+s1)\,(1+\alpha1)}$	$\frac{(-1+m1)\,(-1+r)\,(2+p22\,\alpha2+\alpha1\,(1+p22+p22\,\alpha2))}{2\,(1+s1)\,(1+\alpha1)}$	0
$m2$	0	$\frac{m2\,(p22\,\alpha2+\alpha1\,(-1+p22+p22\,\alpha2)+s2\,(2+p22\,\alpha2+\alpha1\,(1+p22+p22\,\alpha2)))}{2\,(1+\alpha1)}$	1
0	$\frac{m2\,(1+s2)\,(2+p22\,\alpha2+\alpha1\,(1+p22+p22\,\alpha2))}{2\,(1+\alpha1)}$	0	0
0	$-\frac{m2\,(-1+p22)\,p22\,r\,(1+s2)\,(\alpha1+\alpha2+\alpha1\,\alpha2)}{2\,(1+\alpha1)}$	$-\frac{m2\,(-1+r)\,(1+s2)\,(2+p22\,\alpha2+\alpha1\,(1+p22+p22\,\alpha2))}{2\,(1+\alpha1)}$	0

Characteristic polynomial at this equilibrium:

```
cpolgenT20 = Simplify[Det[Jacobdt20 - x IdentityMatrix[6]]]
```

$$\frac{1}{16\,(1+s1)^2\,(1+\alpha1)^4}(-1+x)\,(-1+m1+m2+x)\,\Big(-m1\,m2\,(1+s2)\,(2+p22\,\alpha2+\alpha1\,(1+p22+p22\,\alpha2))^2+(-2-\alpha1-p22\,\alpha1+2\,(1+s1)\,x\,(1+\alpha1)-p22\,\alpha2-p22\,\alpha1\,\alpha2+m1\,(2+p22\,\alpha2+\alpha1\,(1+p22+p22\,\alpha2)))\,(-2+2\,x-\alpha1-p22\,\alpha1+2\,x\,\alpha1-p22\,\alpha2-p22\,\alpha1\,\alpha2-s2\,(2+\alpha1+p22\,\alpha1+p22\,\alpha2+p22\,\alpha1\,\alpha2)+m2\,(1+s2)\,(2+p22\,\alpha2+\alpha1\,(1+p22+p22\,\alpha2)))\Big)\,\Big(-m1\,m2\,(-1+r)^2\,(1+s2)\,(2+p22\,\alpha2+\alpha1\,(1+p22+p22\,\alpha2))^2+(2-2\,x-2\,s1\,x+\alpha1+p22\,\alpha1-2\,x\,\alpha1-2\,s1\,x\,\alpha1+p22\,\alpha2+p22\,\alpha1\,\alpha2-r\,(2+\alpha1+p22\,\alpha1+p22\,\alpha2+p22\,\alpha1\,\alpha2)+m1\,(-1+r)\,(2+p22\,\alpha2+\alpha1\,(1+p22+p22\,\alpha2)))\,(2+2\,s2-2\,x+\alpha1+p22\,\alpha1+s2\,\alpha1+p22\,s2\,\alpha1-2\,x\,\alpha1+p22\,\alpha2+p22\,s2\,\alpha2+p22\,\alpha1\,\alpha2+p22\,s2\,\alpha1\,\alpha2+m2\,(-1+r)\,(1+s2)\,(2+p22\,\alpha2+\alpha1\,(1+p22+p22\,\alpha2))-r\,(1+s2)\,(2+p22\,\alpha2+\alpha1\,(1+p22+p22\,\alpha2)))\Big)$$

There are four factors; x = 1 and x = 1 - m1 - m2 are always eigenvalues.
In addition, there is one factor without r, and one factor with r. Both factors are quadratic in x and given below:

```
cpolgenT20noR[x_, p22_] :=
  (-m1 m2 (1 + s2) (2 + p22 α2 + α1 (1 + p22 + p22 α2))^2 + (-2 - α1 - p22 α1 +
       2 (1 + s1) x (1 + α1) - p22 α2 - p22 α1 α2 + m1 (2 + p22 α2 + α1 (1 + p22 + p22 α2))) (-2 +
       2 x - α1 - p22 α1 + 2 x α1 - p22 α2 - p22 α1 α2 - s2 (2 + α1 + p22 α1 + p22 α2 + p22 α1 α2) +
       m2 (1 + s2) (2 + p22 α2 + α1 (1 + p22 + p22 α2))))
```

```
Simplify[Series[cpolgenT20noR[x, p22], {x, 0, 3}]]
```

$-(-1+m1+m2)(1+s2)(2+p22\,\alpha2+\alpha1(1+p22+p22\,\alpha2))^2+$
$2(-2+m1-s1-s2-s1\,s2+m2(1+s1)(1+s2))(1+\alpha1)(2+p22\,\alpha2+\alpha1(1+p22+p22\,\alpha2))\,x+$
$4(1+s1)(1+\alpha1)^2x^2+O[x]^4$

```
cpolgenT20R[x_, p22_] := (-m1 m2 (-1 + r)^2 (1 + s2) (2 + p22 α2 + α1 (1 + p22 + p22 α2))^2 +
   (2 - 2 x - 2 s1 x + α1 + p22 α1 - 2 x α1 - 2 s1 x α1 + p22 α2 + p22 α1 α2 -
      r (2 + α1 + p22 α1 + p22 α2 + p22 α1 α2) + m1 (-1 + r) (2 + p22 α2 + α1 (1 + p22 + p22 α2)))
    (2 + 2 s2 - 2 x + α1 + p22 α1 + s2 α1 + p22 s2 α1 - 2 x α1 + p22 α2 + p22 s2 α2 +
      p22 α1 α2 + p22 s2 α1 α2 + m2 (-1 + r) (1 + s2) (2 + p22 α2 + α1 (1 + p22 + p22 α2)) -
      r (1 + s2) (2 + p22 α2 + α1 (1 + p22 + p22 α2))))
```

```
Simplify[Series[cpolgenT20R[x, p22], {x, 0, 3}]]
```

$-(-1+m1+m2)(-1+r)^2(1+s2)(2+p22\,\alpha2+\alpha1(1+p22+p22\,\alpha2))^2-$
$2((-1+r)(-2+m1-s1-s2-s1\,s2+m2(1+s1)(1+s2))(1+\alpha1)$
$(2+p22\,\alpha2+\alpha1(1+p22+p22\,\alpha2)))\,x+4(1+s1)(1+\alpha1)^2x^2+O[x]^4$

```
Simplify[cpolgenT20R[x (1 - r), p22] - (1 - r)^2 cpolgenT20noR[x, p22]]
```

0

Therefore, the two polynomials cpolgenT20noR and cpolgenT20R (in x) have the same leading term, and it is positive.

Therefore, cpolgenT20noR[x,p22] = 0 if and only if cpolgenT20R[(1-r)x,p22] = 0. Hence, x is an eigenvalue resulting from cpolgenT20noR[x,p22] = 0 if and only if (1-r)x is an eigenvalue resulting from cpolgenT20R[x,p22] = 0.

As a consequence, an equilibrium p22 (=p12) at this edge (t12=t22=0, dd1=dd2=0) is unstable if and only if there exists and eigenvalue x>1 with cpolgenT20noR[x,p22] = 0.
Thus, a change of stability at this edge occurs at a point p22 if cpolgenT20noR[1,p22] = 0.

The curve of equilibria that connects the edges t12=t22=0 and t12=t22=1 has its endpoint at the value p22, at which the stability at this edge changes, i.e., where cpolgenT20noR[1,p22] = 0.
This is easily computed numerically.

Now, we show: The value of both polynomials at x = 0 is positive (and proportional); their derivatives at x=0 have the same sign, and they are negative!

```
Factor[D[cpolgenT20noR[x, p22], {x, 1}] /. x → 0]
```

$2(-2+m1+m2-s1+m2\,s1-s2+m2\,s2-s1\,s2+m2\,s1\,s2)$
$(1+\alpha1)(2+\alpha1+p22\,\alpha1+p22\,\alpha2+p22\,\alpha1\,\alpha2)$

```
Simplify[Reduce[(-2 + m1 + m2 - s1 + m2 s1 - s2 + m2 s2 - s1 s2 + m2 s1 s2) < 0 &&
   -(1 - m2) < m1/s1 - m2/s2 < 1 - m1 && s1 > 0 && s2 > 0 && 1 > m1 > 0 && 1 > m2 > 0],
 Assumptions → {s1 > 0, s2 > 0, 1 > m1 > 0, 1 > m2 > 0}]
```

$$\left(s2 < m2 + m2\,s2\ \&\&\ \frac{m1\,s2}{m2 + s2 - m1\,s2} < s1 < \frac{m1\,s2}{m2 - s2 + m2\,s2}\right)\ ||\ \left(s2 \geq m2 + m2\,s2\ \&\&\ m2\,s1 > \left(m1 - s1 + m1\,s1\right)\,s2\right)$$

```
Simplify[Reduce[(-2 + m1 + m2 - s1 + m2 s1 - s2 + m2 s2 - s1 s2 + m2 s1 s2) > 0 &&
   -(1 - m2) < m1/s1 - m2/s2 < 1 - m1 && s1 > 0 && s2 > 0 && 1 > m1 > 0 && 1 > m2 > 0],
 Assumptions → {s1 > 0, s2 > 0, 1 > m1 > 0, 1 > m2 > 0}]
```

False

```
FindInstance[(-2 + m1 + m2 - s1 + m2 s1 - s2 + m2 s2 - s1 s2 + m2 s1 s2) < 0 &&
  -(1 - m2) < m1/s1 - m2/s2 < 1 - m1 && s1 > 0 && s2 > 0 && 1 > m1 > 0 && 1 > m2 > 0, {s1, s2, m1, m2}]
```

$$\left\{\left\{s1 \to \frac{5}{16},\ s2 \to \frac{1}{2},\ m1 \to \frac{1}{4},\ m2 \to \frac{1}{2}\right\}\right\}$$

Stability of equilibria at the edge t12 = t22 = 0

By the above, the equilibrium p22 at this edge is unstable if cpolgenT20noR[1, p22] < 0.

```
Simplify[Series[cpolgenT20noR[1, p22], {p22, 0, 3}]]
```

$$\left(-m1\,m2\,(1 + s2)\,(2 + \alpha1)^2 + (\alpha1 + 2\,s1\,(1 + \alpha1) + m1\,(2 + \alpha1))\,(\alpha1 - s2\,(2 + \alpha1) + m2\,(1 + s2)\,(2 + \alpha1))\right) - 2\,\left((m1 + s1 - s2 + s1\,s2 + \alpha1 + s1\,\alpha1 + s1\,s2\,\alpha1 + m1\,s2\,(2 + \alpha1) - m2\,(1 + s2)\,(-1 + s1 + s1\,\alpha1))\,(\alpha1 + \alpha2 + \alpha1\,\alpha2)\right)\,p22 - (-1 + m1 + m2)\,(1 + s2)\,(\alpha1 + \alpha2 + \alpha1\,\alpha2)^2\,p22^2 + O[p22]^4$$

Stability of p22 = 0:

```
FullSimplify[cpolgenT20noR[1, 0]]
```

$$m2\,(1 + s2)\,(2 + \alpha1)\,(\alpha1 + 2\,s1\,(1 + \alpha1)) - (\alpha1 + 2\,s1\,(1 + \alpha1) + m1\,(2 + \alpha1))\,(-\alpha1 + s2\,(2 + \alpha1))$$

This is positive if $\alpha1 > 2\,s2/(1 - s2)$. Then

```
Simplify[D[cpolgenT20noR[x, 0], x] /. x → 1]
```

$$2\,(1 + \alpha1)\,(\alpha1 + 2\,s1\,(1 + \alpha1) + m1\,(2 + \alpha1) + (1 + s1)\,(\alpha1 - s2\,(2 + \alpha1) + m2\,(1 + s2)\,(2 + \alpha1)))$$

is positive. Therefore, p22 = 0 is stable because there are no zeros > 1.

A similar analysis yields stability conditions of p22 = 1.

Complete symmetry of parameters and determination of the endpoints of the curve of equilibria for r>0

By the above, it is sufficient to study cpolgenT20noR[x, p22]

```
Simplify[Series[
  cpolgenT20noR[x, p22] /. {m2 → m, m1 → m, s1 → s, s2 → s, α1 → α, α2 → α}, {x, 0, 2}]]
```

$-(-1+2m)(1+s)(2+\alpha)^2(1+p22\,\alpha)^2+2(-1+m)(2+2s+s^2)(1+p22\,\alpha)(2+3\alpha+\alpha^2)x+4(1+s)(1+\alpha)^2x^2+O[x]^3$

```
cpolgenT20noRsym[p22_, x_, s_, α_, m_] := -(-1 + 2 m) (1 + s) (2 + α)^2 (1 + p22 α)^2 +
  2 (-1 + m) (2 + 2 s + s^2) (1 + p22 α) (2 + 3 α + α^2) x + 4 (1 + s) (1 + α)^2 x^2
```

Compute p22, where the eigenvalue is 1:

```
p22ev1noR = Simplify[Solve[cpolgenT20noRsym[p22, 1, s, α, m] == 0, p22],
  Assumptions → {0 < r < 1/2, α > 0, s > 0, m > s}]
```

$\{\{p22\to-\left(\left(2m+2ms+s^2-\sqrt{\left(s^2(2+s)^2-2ms^2(2+s)^2+m^2(2+2s+s^2)^2\right)}+\alpha+s\alpha+s^2\alpha-\sqrt{\left(s^2(2+s)^2-2ms^2(2+s)^2+m^2(2+2s+s^2)^2\right)}\alpha-ms^2(1+\alpha)\right)\Big/\left((-1+2m)(1+s)\alpha(2+\alpha)\right)\right)\},$
$\{p22\to-\left(\left(2m+2ms+s^2+\sqrt{\left(s^2(2+s)^2-2ms^2(2+s)^2+m^2(2+2s+s^2)^2\right)}+\alpha+s\alpha+s^2\alpha+\sqrt{\left(s^2(2+s)^2-2ms^2(2+s)^2+m^2(2+2s+s^2)^2\right)}\alpha-ms^2(1+\alpha)\right)\Big/\left((-1+2m)(1+s)\alpha(2+\alpha)\right)\right)\}\}$

```
N[{p22ev1noR /. {s → 0.2, α → 1, m → 0.01},
  p22ev1noR /. {s → 0.2, α → 10, m → 0.01}, p22ev1noR /. {s → 0.2, α → 100, m → 0.01}}]
```

```
{{{p22 → 0.122074}, {p22 → 0.616701}},
 {{p22 → 0.0542852}, {p22 → 0.122296}}, {{p22 → 0.00666611}, {p22 → 0.0140128}}}
```

Stability of p22 = 0:

```
FullSimplify[cpolgenT20noRsym[0, 1, s, α, m]]
```

$2(-1+m)s^2(1+\alpha)(2+\alpha)+\alpha(\alpha+2m(2+\alpha))+s\alpha(\alpha+2m(2+\alpha))$

This is always positive for m sufficiently close to 1

```
FullSimplify[cpolgenT20noRsym[0, 1, s, α, 0]]
```

$-(\alpha+2s(1+\alpha))(-\alpha+s(2+\alpha))$

This is positive if $\alpha > \frac{2s}{1-s}$ and negative otherwise !

```
FullSimplify[cpolgenT20noRsym[0, 1, s, α, m] /. α -> 2 s/(1 - s) (1 + z)]
```

$\frac{1}{(-1+s)^2}4s\left(-(-1+s)sz(2+s+z+2sz)+m(1+sz)\left(2+3s+s^2+2(1+s+s^2)z\right)\right)$

This is positive whenever $\alpha > \frac{2s}{1-s}$ (and $s < 1$)

```
FullSimplify[D[cpolgenT20noRsym[0, x, s, α, m], x] /. x → 1]
```

$2(1+\alpha)\left(4(1+s)(1+\alpha)+(-1+m)(2+s(2+s))(2+\alpha)\right)$

```
FullSimplify[D[cpolgenT20noRsym[0, x, s, α, m], x] /. x → 1 /. α -> (2 s)/(1 - s) (1 + z)]
```

$$\frac{1}{(-1+s)^2} 4\ (1+s+2\,s\,z)\ (m\ (2+s\ (2+s))\ (1+s\,z)+s\ (s-(-2+s)\ s\,z+2\ (1+z)))$$

This is positive whenever s < 1.

Therefore, p22 = 0 is stable if $\alpha > \frac{2\,s}{1-s}$

In a similar way it can be shown that p22 = 1 unstable, and stability changes exactly once between p22 = 0 and p22 = 1

The following computes the eigenvalue that determines stability of p22 :

```
EVsgenT20noRsym[p22_, s_, α_, m_] := x /. NSolve[cpolgenT20noRsym[p22, x, s, α, m] == 0, x]
```

```
EVsgenT20noRsym[0.1, 0.2, 10^0, 0.01]
```

```
{0.680398, 0.980327}
```

Plot region in which the maximum eigenvalue is less than 1 in modulus (implying stability of the edge equilibrium t2 = 0, p22, D = 0). This region increases with increases m:

```
RegionPlot[{Abs[Max[EVsgenT20noRsym[p22, 0.2, 10^a, 0.01]]] < 1,
  Abs[Max[EVsgenT20noRsym[p22, 0.2, 10^a, 0.05]]] < 1},
 {a, -2, 3}, {p22, 0, 0.2}, FrameLabel → {"Log10(α)", "p22"}]
```

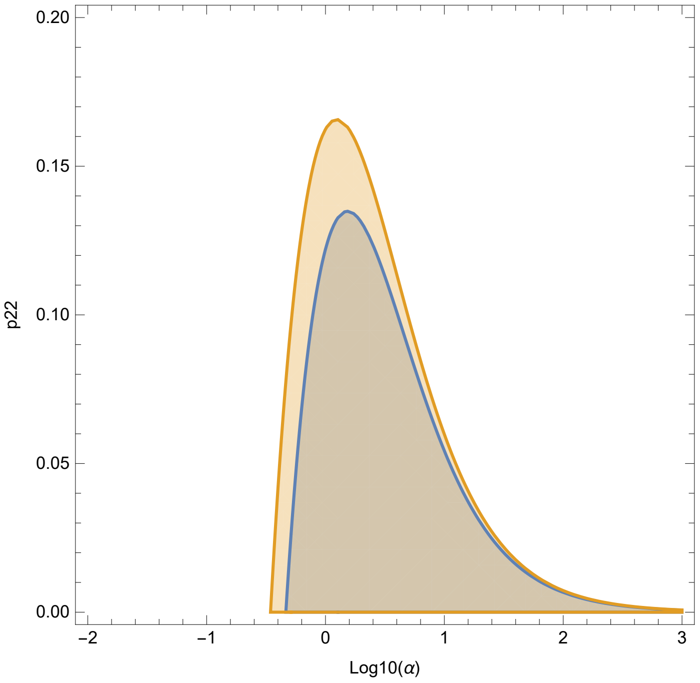


```
NSolve[cpolgenT20noRsym[p22, 1, 0.2, 1, 0.01] == 0, p22]
```

```
{{p22 → 0.122074}, {p22 → 0.616701}}
```

```
NSolve[cpolgenT20noRsym[p22, 1, 0.2, 10, 0.01] == 0, p22]
```

```
{{p22 → 0.0542852}, {p22 → 0.122296}}
```

```
NSolve[cpolgenT20noRsym[p22, 1, 0.2, 100, 0.01] == 0, p22]
```

{{p22 → 0.00666611}, {p22 → 0.0140128}}

Therefore, if {s -> 0.2, α -> 1, m -> 0.01, r > 0}, then the equilibria with p22 < 0.122074 are stable.
Therefore, if {s -> 0.2, α -> 10, m -> 0.01, r > 0}, then the equilibria with p22 < 0.0542852 are stable.
Therefore, if {s -> 0.2, α -> 100, m -> 0.01, r > 0}, then the equilibria with p22 < 0.00666611 are stable.

Jacobian at edge t12 = t22 = 1 and stability of edge equilibria

This analysis is analogous to that at t12 = t22 = 0

```
Jacobdt21 =
  Simplify[D[itgen[s1, s2, α1, α2, m1, m2, r][{p12, t12, dd1, p22, t22, dd2}], {{p12,
      t12, dd1, p22, t22, dd2}}] /. {t12 → 1, dd1 → 0, t22 → 1, dd2 → 0, p12 → p22}];
```

An equilibrium is stable if the modulus of eigenvalues of its Jacobian is < 1

```
TableForm[Jacobdt21]
```

1 − m1	0	$-\frac{(-1+m1)\,(p22\,\alpha2+(-1+p22)\,\alpha1\,(1+\alpha2)+s1\,(-2+(-2+p22)\,\alpha2+\ldots}{2\,(1+\alpha2)}$ [illegible]
0	$\frac{(-1+m1)\,(1+s1)\,(-2+(-2+p22)\,\alpha2+(-1+p22)\,\alpha1\,(1+\alpha2))}{2\,(1+\alpha2)}$	0
0	$-\frac{(-1+m1)\,(-1+p22)\,p22\,r\,(1+s1)\,(\alpha1+\alpha2+\alpha1\,\alpha2)}{2\,(1+\alpha2)}$	$-\frac{(-1+m1)\,(-1+r)\,(1+s1)\,(-2+(-2+p22)\,\alpha2+(-1+p22)\,\alpha1\,(1+\ldots}{2\,(1+\alpha2)}$ [illegible]
m2	0	$\frac{m2\,(p22\,\alpha2+2\,s2\,(1+\alpha2)+(-1+p22)\,\alpha1\,(1+\alpha2))}{2\,(1+s2)\,(1+\alpha2)}$
0	$-\frac{m2\,(-2+(-2+p22)\,\alpha2+(-1+p22)\,\alpha1\,(1+\alpha2))}{2\,(1+s2)\,(1+\alpha2)}$	0
0	$\frac{m2\,(-1+p22)\,p22\,r\,(\alpha1+\alpha2+\alpha1\,\alpha2)}{2\,(1+s2)\,(1+\alpha2)}$	$\frac{m2\,(-1+r)\,(-2+(-2+p22)\,\alpha2+(-1+p22)\,\alpha1\,(1+\alpha2))}{2\,(1+s2)\,(1+\alpha2)}$

```
cpolgenT21 = Simplify[Det[Jacobdt21 - x IdentityMatrix[6]]]
```

$$\begin{aligned}
&\frac{1}{16\,(1+s2)^2\,(1+\alpha2)^4}\\
&(-1+x)\,(-1+m1+m2+x)\,\Big(-m1\,m2\,(1+s1)\,\big(-2+(-2+p22)\,\alpha2+(-1+p22)\,\alpha1\,(1+\alpha2)\big)^2+\\
&\quad\big(2+\alpha1-p22\,\alpha1+2\,\alpha2-p22\,\alpha2+\alpha1\,\alpha2-p22\,\alpha1\,\alpha2-2\,(1+s2)\,x\,(1+\alpha2)+\\
&\qquad m2\,\big(-2+(-2+p22)\,\alpha2+(-1+p22)\,\alpha1\,(1+\alpha2)\big)\big)\,\big(2-2\,x+\alpha1-p22\,\alpha1+2\,\alpha2-\\
&\qquad p22\,\alpha2-2\,x\,\alpha2+\alpha1\,\alpha2-p22\,\alpha1\,\alpha2+s1\,\big(2-(-2+p22)\,\alpha2-(-1+p22)\,\alpha1\,(1+\alpha2)\big)+\\
&\qquad m1\,(1+s1)\,\big(-2+(-2+p22)\,\alpha2+(-1+p22)\,\alpha1\,(1+\alpha2)\big)\big)\Big)\\
&\Big(-m1\,m2\,(-1+r)^2\,(1+s1)\,\big(-2+(-2+p22)\,\alpha2+(-1+p22)\,\alpha1\,(1+\alpha2)\big)^2+\\
&\quad\big(-2+2\,x+2\,s2\,x-\alpha1+p22\,\alpha1-2\,\alpha2+p22\,\alpha2+2\,x\,\alpha2+2\,s2\,x\,\alpha2-\\
&\qquad \alpha1\,\alpha2+p22\,\alpha1\,\alpha2+r\,\big(2-(-2+p22)\,\alpha2-(-1+p22)\,\alpha1\,(1+\alpha2)\big)+\\
&\qquad m2\,(-1+r)\,\big(-2+(-2+p22)\,\alpha2+(-1+p22)\,\alpha1\,(1+\alpha2)\big)\big)\\
&\quad\big(-2-2\,s1+2\,x-\alpha1+p22\,\alpha1-s1\,\alpha1+p22\,s1\,\alpha1-2\,\alpha2+p22\,\alpha2-2\,s1\,\alpha2+\\
&\qquad p22\,s1\,\alpha2+2\,x\,\alpha2-\alpha1\,\alpha2+p22\,\alpha1\,\alpha2-s1\,\alpha1\,\alpha2+p22\,s1\,\alpha1\,\alpha2+\\
&\qquad m1\,(-1+r)\,(1+s1)\,\big(-2+(-2+p22)\,\alpha2+(-1+p22)\,\alpha1\,(1+\alpha2)\big)-\\
&\qquad r\,(1+s1)\,\big(-2+(-2+p22)\,\alpha2+(-1+p22)\,\alpha1\,(1+\alpha2)\big)\big)\Big)
\end{aligned}$$

There are four factors; x = 1 and x = 1 - m1 - m2 are always eigenvalues;
in addition, there is one factor without r, and one factor with r (each of them quadratic)

```
cpolgenT21noR[x_, p22_] := (-m1 m2 (1 + s1) (-2 + (-2 + p22) α2 + (-1 + p22) α1 (1 + α2))^2 +
   (2 + α1 - p22 α1 + 2 α2 - p22 α2 + α1 α2 - p22 α1 α2 - 2 (1 + s2) x (1 + α2) +
      m2 (-2 + (-2 + p22) α2 + (-1 + p22) α1 (1 + α2))) (2 - 2 x + α1 - p22 α1 + 2 α2 -
      p22 α2 - 2 x α2 + α1 α2 - p22 α1 α2 + s1 (2 - (-2 + p22) α2 - (-1 + p22) α1 (1 + α2)) +
      m1 (1 + s1) (-2 + (-2 + p22) α2 + (-1 + p22) α1 (1 + α2))))
```

```
Simplify[Series[cpolgenT21noR[x, p22], {x, 0, 3}]]
```

$$-(-1+m1+m2)(1+s1)(-2+(-2+p22)\alpha 2+(-1+p22)\alpha 1(1+\alpha 2))^2 - 2((-2+m2-s1-s2-s1\,s2+m1(1+s1)(1+s2))(1+\alpha 2)(-2+(-2+p22)\alpha 2+(-1+p22)\alpha 1(1+\alpha 2)))x+4(1+s2)(1+\alpha 2)^2x^2+O[x]^4$$

```
cpolgenT21R[x_, p22_] :=
 (-m1 m2 (-1 + r)^2 (1 + s1) (-2 + (-2 + p22) α2 + (-1 + p22) α1 (1 + α2))^2 +
   (-2 + 2 x + 2 s2 x - α1 + p22 α1 - 2 α2 + p22 α2 + 2 x α2 + 2 s2 x α2 -
      α1 α2 + p22 α1 α2 + r (2 - (-2 + p22) α2 - (-1 + p22) α1 (1 + α2)) +
      m2 (-1 + r) (-2 + (-2 + p22) α2 + (-1 + p22) α1 (1 + α2)))
    (-2 - 2 s1 + 2 x - α1 + p22 α1 - s1 α1 + p22 s1 α1 - 2 α2 + p22 α2 - 2 s1 α2 +
      p22 s1 α2 + 2 x α2 - α1 α2 + p22 α1 α2 - s1 α1 α2 + p22 s1 α1 α2 +
      m1 (-1 + r) (1 + s1) (-2 + (-2 + p22) α2 + (-1 + p22) α1 (1 + α2)) -
      r (1 + s1) (-2 + (-2 + p22) α2 + (-1 + p22) α1 (1 + α2))))
```

```
Simplify[cpolgenT21R[x (1 - r), p22] - (1 - r)^2 cpolgenT21noR[x, p22]]
```

0

We have (1-r)^2 cpolgenT21noR[x, p22] = cpolgenT20R[x, p22] and an analysis as above shows that stability at this edge is changes at the value p22 at which cpolgenT21noR[1, p22]=0.

We have cpolgenT21noR[x, p22] = a0 + a1 x + a2 x^2, where a0 > 0, a1 < 0, a2 > 0.
We have cpolgenT21R[x, p22] = (1 - r)^2 a0 + (1 - r) a1 x + a2 x^2

Complete symmetry of parameters and determination of the endpoints of the curve of equilibria for r>0

```
Simplify[Series[
  cpolgenT21noR[x, p22] //. {s2 → s1, s1 → s, α2 → α1, α1 → α, m2 → m1, m1 → m}, {x, 0, 3}]]
```

$$-(-1+2m)(1+s)(2+\alpha)^2(-1+(-1+p22)\alpha)^2 - 2((-1+m)(2+2s+s^2)(-1+(-1+p22)\alpha)(2+3\alpha+\alpha^2))x+4(1+s)(1+\alpha)^2x^2+O[x]^4$$

```
cpolgenT21noRsym[p22_, x_, s_, α_, m_] := -(-1 + 2 m) (1 + s) (2 + α)^2 (-1 + (-1 + p22) α)^2 -
   2 ((-1 + m) (2 + 2 s + s^2) (-1 + (-1 + p22) α) (2 + 3 α + α^2)) x + 4 (1 + s) (1 + α)^2 x^2
```

An analogous analysis yields the conditions of stability at the edge t12 = t22 =1. In particular, we obtain:

Therefore, if {s -> 0.2, α -> 10, m -> 0.01, r > 0}, then the equilibria with p22 > 0.945715 are

stable.
If {s -> 0.2, α -> 1, m -> 0.01, r > 0}, then the equilibria with p22 > 0.877926 are stable.

2.2 Equilibria under random mating (α1 = α2 = 0)

```
condeq = FullSimplify[itgen[s1, s2, 0, 0, m1, m2, r][{p12, t12, d1, p22, t22, d2}] -
    {p12, t12, d1, p22, t22, d2}]
```

$$\left\{\left(-d1\,s1+m1\left(p22+d1\,s1-d2\,s1+p22\,s1+p12\left(-1+s1\left(-1+t22\right)\right)-p22\,s1\,t22\right)\right)\Big/\left(1+s1\left(1+\left(-1+m1\right)t12-m1\,t22\right)\right),\ \frac{s1\left(-1+t12\right)t12-m1\left(1+s1\,t12\right)\left(t12-t22\right)}{1+s1\left(1+\left(-1+m1\right)t12-m1\,t22\right)},\right.$$

$$-\frac{1}{\left(1+s1\left(1+\left(-1+m1\right)t12-m1\,t22\right)\right)^2}\left(m1\left(-1+r\right)\left(1+s1\right)\left(d2-\left(-1+m1\right)\left(p12-p22\right)\left(t12-t22\right)\right)+d1\left(r+s1+s1\left(r+s1\left(-1+t12\right)^2-2\,t12\right)+m1\left(1+s1-r\left(1+s1\right)-2\,s1\left(-1+s1\left(-1+t12\right)\right)\right)\left(t12-t22\right)\right)+m1^2\,s1^2\left(t12-t22\right)^2\right)\right),$$

$$\left(d2\,s2+m2\left(p12+\left(d1-d2\right)s2+p12\,s2\,t12-p22\left(1+s2\,t12\right)\right)\right)\Big/\left(1+m2\,s2\left(t12-t22\right)+s2\,t22\right),$$

$$1+\frac{1}{s2}-t22-\frac{1+s2}{s2\left(1+m2\,s2\left(t12-t22\right)+s2\,t22\right)},$$

$$\frac{1}{\left(1+m2\,s2\left(t12-t22\right)+s2\,t22\right)^2}\left(-d1\,m2\left(-1+r\right)\left(1+s2\right)+\left(-1+m2\right)m2\left(p12-p22\right)\left(-1+r\right)\left(1+s2\right)\left(t12-t22\right)-d2\left(r-s2+m2^2\,s2^2\left(t12-t22\right)^2+m2\left(1+s2-r\left(1+s2\right)+2\,s2\left(t12-t22\right)\left(1+s2\,t22\right)\right)+s2\left(r+t22\left(2+s2\,t22\right)\right)\right)\right)\left.\right\}$$

```
Simplify[Solve[{s1 (-1 + t12) t12 - m1 (1 + s1 t12) (t12 - t22),
    1 + 1/s2 - t22 - (1 + s2)/(s2 (1 + m2 s2 (t12 - t22) + s2 t22))} == 0,
  {t12, t22}], Assumptions → {s1 > 0, s2 > 0, m1 > 0, m2 > 0}]
```

```
{{t12 → 0, t22 → 0}, {t12 → 1, t22 → 1}, {t12 →
   -1/(2 (-1 + m1) (-1 + m1 + m2) s1 s2) (-m1 m2 s1 - 2 m1 s2 + 2 m1^2 s2 + m1 m2 s2 - s1 s2 + m1 s1 s2 + m2
        s1 s2 - m1 m2 s1 s2 + √(-4 (-1 + m1) m1 (-1 + m1 + m2) s2 ((m1 + s1) s2 - m2 s1 (1 + s2)) +
          (2 m1^2 s2 + (-1 + m2) s1 s2 + m1 ((-2 + s1) s2 - m2 (s1 - s2 + s1 s2)))^2)),
  t22 → (1/(4 (-1 + m1) m1 (-1 + m2) (-1 + m1 + m2) s1 s2^2))
    (-m1 m2 s1 - 2 m1 s2 + 2 m1^2 s2 + m1 m2 s2 - s1 s2 + m1 s1 s2 + m2 s1 s2 - m1 m2 s1 s2 +
      √(-4 (-1 + m1) m1 (-1 + m1 + m2) s2 ((m1 + s1) s2 - m2 s1 (1 + s2)) +
          (2 m1^2 s2 + (-1 + m2) s1 s2 + m1 ((-2 + s1) s2 - m2 (s1 - s2 + s1 s2)))^2))
    (m1 m2 s1 - m1 m2 s2 + s1 s2 - m1 s1 s2 - m2 s1 s2 + m1 m2 s1 s2 +
      √(-4 (-1 + m1) m1 (-1 + m1 + m2) s2 ((m1 + s1) s2 - m2 s1 (1 + s2)) +
          (2 m1^2 s2 + (-1 + m2) s1 s2 + m1 ((-2 + s1) s2 - m2 (s1 - s2 + s1 s2)))^2))},
 {t12 → 1/(2 (-1 + m1) (-1 + m1 + m2) s1 s2) (m1 m2 s1 + 2 m1 s2 - 2 m1^2 s2 - m1 m2 s2 + s1 s2 - m1 s1 s2 -
      m2 s1 s2 + m1 m2 s1 s2 + √(-4 (-1 + m1) m1 (-1 + m1 + m2) s2 ((m1 + s1) s2 - m2 s1 (1 + s2)) +
         (2 m1^2 s2 + (-1 + m2) s1 s2 + m1 ((-2 + s1) s2 - m2 (s1 - s2 + s1 s2)))^2)),
  t22 → (1/(4 (-1 + m1) m1 (-1 + m2) (-1 + m1 + m2) s1 s2^2))
    (-m1 m2 s1 + m1 m2 s2 - s1 s2 + m1 s1 s2 + m2 s1 s2 - m1 m2 s1 s2 +
      √(-4 (-1 + m1) m1 (-1 + m1 + m2) s2 ((m1 + s1) s2 - m2 s1 (1 + s2)) +
          (2 m1^2 s2 + (-1 + m2) s1 s2 + m1 ((-2 + s1) s2 - m2 (s1 - s2 + s1 s2)))^2))
    (m1 m2 s1 + 2 m1 s2 - 2 m1^2 s2 - m1 m2 s2 + s1 s2 - m1 s1 s2 - m2 s1 s2 + m1 m2 s1 s2 +
      √(-4 (-1 + m1) m1 (-1 + m1 + m2) s2 ((m1 + s1) s2 - m2 s1 (1 + s2)) +
          (2 m1^2 s2 + (-1 + m2) s1 s2 + m1 ((-2 + s1) s2 - m2 (s1 - s2 + s1 s2)))^2))}}
```

Comparison with the polymorphism from 1-locus model shows that the migration-selection equilibrium is

```
migselsol = {t12 → - 1/(2 (-1 + m1) (-1 + m1 + m2) s1 s2)
      (-m1 m2 s1 - 2 m1 s2 + 2 m1^2 s2 + m1 m2 s2 - s1 s2 + m1 s1 s2 + m2 s1 s2 - m1 m2 s1 s2 +
        √(-4 (-1 + m1) m1 (-1 + m1 + m2) s2 ((m1 + s1) s2 - m2 s1 (1 + s2)) +
           (2 m1^2 s2 + (-1 + m2) s1 s2 + m1 ((-2 + s1) s2 - m2 (s1 - s2 + s1 s2)))^2)),
    t22 → (1 / (4 (-1 + m1) m1 (-1 + m2) (-1 + m1 + m2) s1 s2^2))
      (-m1 m2 s1 - 2 m1 s2 + 2 m1^2 s2 + m1 m2 s2 - s1 s2 + m1 s1 s2 + m2 s1 s2 - m1 m2 s1 s2 +
        √(-4 (-1 + m1) m1 (-1 + m1 + m2) s2 ((m1 + s1) s2 - m2 s1 (1 + s2)) +
           (2 m1^2 s2 + (-1 + m2) s1 s2 + m1 ((-2 + s1) s2 - m2 (s1 - s2 + s1 s2)))^2))
      (m1 m2 s1 - m1 m2 s2 + s1 s2 - m1 s1 s2 - m2 s1 s2 + m1 m2 s1 s2 +
        √(-4 (-1 + m1) m1 (-1 + m1 + m2) s2 ((m1 + s1) s2 - m2 s1 (1 + s2)) +
           (2 m1^2 s2 + (-1 + m2) s1 s2 + m1 ((-2 + s1) s2 - m2 (s1 - s2 + s1 s2)))^2))};
```

```
Simplify[{condeq[[2]], condeq[[5]]} /. migselsol,
 Assumptions → {s1 > 0, s2 > 0, m1 > 0, m2 > 0}]
```

```
{0, 0}
```

Determine other coordinates:

```
Simplify[Numerator[Factor[{condeq[[1]], condeq[[4]]} /. migselsol]],
 Assumptions → {s1 > 0, s2 > 0, m1 > 0, m2 > 0}]
```

```
{2 d1 (-1 + m2)^2 s1 s2 + m1^2 ((2 (d1 - d2) s1 - p12 (2 + s1) + p22 (2 + s1)) s2 +
      m2 (2 (-d1 + d2) s1 s2 + p12 (s1 (-1 + s2) + 3 s2) + p22 (s1 - 3 s2 - s1 s2))) +
   m1 (2 m2 p12 s1 - 2 m2^2 p12 s1 - 2 m2 p22 s1 + 2 m2^2 p22 s1 + 2 p12 s2 - 4 m2 p12 s2 + 2 m2^2 p12 s2 -
      2 p22 s2 + 4 m2 p22 s2 - 2 m2^2 p22 s2 - 4 d1 s1 s2 + 2 d2 s1 s2 + 6 d1 m2 s1 s2 - 4 d2 m2 s1 s2 -
      2 d1 m2^2 s1 s2 + 2 d2 m2^2 s1 s2 + p12 s1 s2 - m2 p12 s1 s2 - p22 s1 s2 + m2 p22 s1 s2 -
      p12 √((-1 + m2)^2 s1^2 s2^2 - 2 m1 (-1 + m2) s1 s2 (-s1 s2 + m2 (2 + s1 + s2 + s1 s2)) +
          m1^2 (s1^2 s2^2 + m2^2 (s1 + s2 + s1 s2)^2 - 2 m2 s1 s2 (2 + s1 + s2 + s1 s2))) +
      p22 √((-1 + m2)^2 s1^2 s2^2 - 2 m1 (-1 + m2) s1 s2 (-s1 s2 + m2 (2 + s1 + s2 + s1 s2)) +
          m1^2 (s1^2 s2^2 + m2^2 (s1 + s2 + s1 s2)^2 - 2 m2 s1 s2 (2 + s1 + s2 + s1 s2)))),
 -2 d2 (-1 + m1)^2 s1 s2 + m2^2 (-2 (d1 - d2) (-1 + m1) s1 s2 +
      p12 (m1 s2 + s1 (2 + s2 - m1 (3 + s2))) + p22 (-m1 s2 + s1 (-2 - s2 + m1 (3 + s2)))) +
   m2 (-2 p12 s1 + 4 m1 p12 s1 - 2 m1^2 p12 s1 + 2 p22 s1 - 4 m1 p22 s1 + 2 m1^2 p22 s1 - 2 m1 p12 s2 +
      2 m1^2 p12 s2 + 2 m1 p22 s2 - 2 m1^2 p22 s2 - 2 d1 s1 s2 + 4 d2 s1 s2 + 4 d1 m1 s1 s2 - 6 d2 m1 s1 s2 -
      2 d1 m1^2 s1 s2 + 2 d2 m1^2 s1 s2 - p12 s1 s2 + m1 p12 s1 s2 + p22 s1 s2 - m1 p22 s1 s2 +
      p12 √((-1 + m2)^2 s1^2 s2^2 - 2 m1 (-1 + m2) s1 s2 (-s1 s2 + m2 (2 + s1 + s2 + s1 s2)) +
          m1^2 (s1^2 s2^2 + m2^2 (s1 + s2 + s1 s2)^2 - 2 m2 s1 s2 (2 + s1 + s2 + s1 s2))) -
      p22 √((-1 + m2)^2 s1^2 s2^2 - 2 m1 (-1 + m2) s1 s2 (-s1 s2 + m2 (2 + s1 + s2 + s1 s2)) +
          m1^2 (s1^2 s2^2 + m2^2 (s1 + s2 + s1 s2)^2 - 2 m2 s1 s2 (2 + s1 + s2 + s1 s2))))}
```

```
Simplify[Solve[% == 0, {d1, d2}]]
```

```
{{d1 → 1/(2 (-1 + m1) (-1 + m1 + m2) s1 s2) m1 (p12 - p22)
    (-m1 m2 s1 - 2 s2 + 2 m1 s2 + 2 m2 s2 - m1 m2 s2 - s1 s2 + m1 s1 s2 + m2 s1 s2 - m1 m2 s1 s2 +
      √(((-1 + m2)^2 s1^2 s2^2 - 2 m1 (-1 + m2) s1 s2 (-s1 s2 + m2 (2 + s1 + s2 + s1 s2)) +
         m1^2 (s1^2 s2^2 + m2^2 (s1 + s2 + s1 s2)^2 - 2 m2 s1 s2 (2 + s1 + s2 + s1 s2))))),
  d2 → 1/(2 (-1 + m2) (-1 + m1 + m2) s1 s2) m2 (p12 - p22) (-2 s1 + 2 m1 s1 + 2 m2 s1 -
      m1 m2 s1 - m1 m2 s2 - s1 s2 + m1 s1 s2 + m2 s1 s2 - m1 m2 s1 s2 +
      √(((-1 + m2)^2 s1^2 s2^2 - 2 m1 (-1 + m2) s1 s2 (-s1 s2 + m2 (2 + s1 + s2 + s1 s2)) +
         m1^2 (s1^2 s2^2 + m2^2 (s1 + s2 + s1 s2)^2 - 2 m2 s1 s2 (2 + s1 + s2 + s1 s2)))))}}
```

```
d1d2alpha0 = {d1 → 1/(2 (-1 + m1) (-1 + m1 + m2) s1 s2) m1 (p12 - p22)
    (-m1 m2 s1 - 2 s2 + 2 m1 s2 + 2 m2 s2 - m1 m2 s2 - s1 s2 + m1 s1 s2 + m2 s1 s2 - m1 m2 s1 s2 +
      √(((-1 + m2)^2 s1^2 s2^2 - 2 m1 (-1 + m2) s1 s2 (-s1 s2 + m2 (2 + s1 + s2 + s1 s2)) +
         m1^2 (s1^2 s2^2 + m2^2 (s1 + s2 + s1 s2)^2 - 2 m2 s1 s2 (2 + s1 + s2 + s1 s2))))),
   d2 → 1/(2 (-1 + m2) (-1 + m1 + m2) s1 s2) m2 (p12 - p22) (-2 s1 + 2 m1 s1 + 2 m2 s1 -
      m1 m2 s1 - m1 m2 s2 - s1 s2 + m1 s1 s2 + m2 s1 s2 - m1 m2 s1 s2 +
      √(((-1 + m2)^2 s1^2 s2^2 - 2 m1 (-1 + m2) s1 s2 (-s1 s2 + m2 (2 + s1 + s2 + s1 s2)) +
         m1^2 (s1^2 s2^2 + m2^2 (s1 + s2 + s1 s2)^2 - 2 m2 s1 s2 (2 + s1 + s2 + s1 s2)))))};
```

We have p12 = p22 if and only if d1 = d2 = 0!!!

```
Simplify[condeq /. migselsol /. d1d2alpha0]
```

```
{0, 0, -((2 m1 (-1 + m2) (p12 - p22) r (1 + s1) s2) /
    (s1 (m1 m2 s1 + 2 s2 - 2 m1 s2 - 2 m2 s2 + m1 m2 s2 + s1 s2 - m1 s1 s2 - m2 s1 s2 +
       m1 m2 s1 s2 + √(-4 (-1 + m1) m1 (-1 + m1 + m2) s2 ((m1 + s1) s2 - m2 s1 (1 + s2)) +
          (2 m1^2 s2 + (-1 + m2) s1 s2 + m1 ((-2 + s1) s2 - m2 (s1 - s2 + s1 s2)))^2)))),
 0, 0, -((2 (-1 + m1) m2 (p12 - p22) r s1 (1 + s2)) /
    (s2 (2 s1 - 2 m1 s1 - 2 m2 s1 + m1 m2 s1 + m1 m2 s2 + s1 s2 - m1 s1 s2 - m2 s1 s2 +
       m1 m2 s1 s2 + √(-4 (-1 + m1) m1 (-1 + m1 + m2) s2 ((m1 + s1) s2 - m2 s1 (1 + s2)) +
          (2 m1^2 s2 + (-1 + m2) s1 s2 + m1 ((-2 + s1) s2 - m2 (s1 - s2 + s1 s2)))^2))))}
```

```
Simplify[Solve[% == 0, {p12, p22}]]
```

Solve: Equations may not give solutions for all "solve" variables.

```
{{p22 → p12}}
```

Therefore: If $\alpha 1 = \alpha 2 = 0$, the equilibria are given by
p12 = p22, d1 = d2 = 0, and {t12, t22} are the allele frequencies at migration-selection balance (migselsol).
Therefore, as expected, there is a line of equilibria.

The condition for existence of the polymorphic migration – selection equilibrium is $-(1-m2) < \frac{m1}{s1} - \frac{m2}{s2} < 1 - m1$

2.3 Derivation of manifold/curve of equilibria for r=0/r>0 (assuming symmetric parameters)

2.3.1 Equilibrium conditions and their structure

Solving deltagensym = 0 yields the potential equilibria (conditional on admissibility)

```
deltagensym = Simplify[itgen[s, s, α, α, m, m, r][{p12, t12, dd1, p22, t22, dd2}] -
   {p12, t12, dd1, p22, t22, dd2}]
```

```
deltagensym =
  {-p12 + (-(-1 + m)^2 m p12^2 (1 + s) (-1 + s (-1 + t12)) (t12 - t22) α (2 + α) - dd1 (α + t12 α^2 +
          2 s^3 (-1 + t12)^2 (1 + α) + s (2 + (4 + 2 dd1 - t12) α + (dd1 + (3 - 2 t12) t12) α^2) +
          s^2 (4 + (5 + 2 dd1) α + dd1 α^2 - 2 t12^2 α^2 + t12 (-4 - 5 α + 2 α^2))) -
       m^3 (t12 - t22) (p22^2 (1 + s) (-1 + s (-1 + t22)) α (2 + α) - 2 (dd1 - dd2) s (t12 - t22)
          (s - α) (s + α + s α) + p22 ((t12 - t22) α^2 + 2 s^3 (t12 - t22) (-1 + t22) (1 + α) -
             s α (dd1 (2 + α) - dd2 (2 + α) + (t12 - t22) (-1 - 3 α + 2 t22 α)) + s^2 (2 t22^2 α^2 -
                (dd1 - dd2) α (2 + α) + t22 (2 + α - 2 α^2) - t12 (2 + α + 2 (-1 + t22) α^2)))) +
       m^2 (-dd1^2 s (1 + s) α (2 + α) + p22^2 (1 + s) (-1 + s (-1 + t22)) (t12 - t22)
          α (2 + α) - dd2 ((-t12 + t22) α^2 - 4 s^3 (-1 + t12) (t12 - t22) (1 + α) +
             s (dd2 α (2 + α) + (t12 - t22) α (1 + (-3 + 4 t12) α)) + s^2 (4 t12^2 α^2 +
                dd2 α (2 + α) + t22 (-4 - 5 α + 2 α^2) + t12 (4 + 5 α - 2 (1 + 2 t22) α^2))) +
          dd1 ((-t12 + t22) α^2 - 2 s^3 (3 t12^2 + t22 (2 + t22) - 2 t12 (1 + 2 t22)) (1 + α) +
             s (2 dd2 α (2 + α) + (t12 - t22) α (1 + (-3 + 6 t12 - 2 t22) α)) +
             s^2 (6 t12^2 α^2 + 2 t22^2 α^2 + 2 dd2 α (2 + α) +
                t22 (-4 - 5 α + 2 α^2) + t12 (4 + 5 α - 2 (1 + 4 t22) α^2))) +
          p22 (-2 dd1 α + 2 dd2 α - t12 α + t22 α - dd1 α^2 + dd2 α^2 - 2 t12 α^2 + 2 t12^2 α^2 +
             2 t22 α^2 - t12 t22 α^2 - t22^2 α^2 + 4 s^3 (-1 + t12) (t12 - t22) (-1 + t22) (1 + α) +
             s (-2 (dd1 - dd2) α (2 + α) + 2 t12^2 α (1 + (3 - 2 t22) α) + t22^2 (α - 3 α^2) +
                t22 (-4 + (-2 + 6 dd1 - 4 dd2) α + (4 + 3 dd1 - 2 dd2) α^2) + t12
                 (4 + (2 - 4 dd1 + 2 dd2 - 3 t22) α + (-4 - 2 dd1 + dd2 - 3 t22 + 4 t22^2) α^2)) +
             s^2 (-(dd1 - dd2) α (2 + α) + t22^2 (4 + 5 α - 2 α^2) + t22 (-8 + (-7 + 6 dd1 - 4 dd2)
                     α + (2 + 3 dd1 - 2 dd2) α^2) - 2 t12^2 (2 + α + 2 (-1 + t22) α^2) + t12 (8 +
                    (7 - 4 dd1 + 2 dd2 - 3 t22) α + (-2 - 2 dd1 + dd2 - 2 t22 + 4 t22^2) α^2)))) +
       m (2 dd1^2 s (1 + s) α (2 + α) - dd2 (-1 + s (-1 + t12) - t12 α)
          (-α + 2 s^2 (-1 + t12) (1 + α) + s (-2 + (-3 + 2 t12) α)) +
          dd1 (2 s^3 (-1 + t12) (-1 + 3 t12 - 2 t22) (1 + α) + α (1 + 2 t12 α - t22 α) +
             s (2 + (4 - 4 dd2 - 2 t12 + t22) α + (-2 dd2 + 6 t12 - 6 t12^2 - 3 t22 + 4 t12 t22)
                 α^2) + s^2 (4 + 5 α - 4 dd2 α - 2 dd2 α^2 - 6 t12^2 α^2 +
                t22 (4 + 5 α - 2 α^2) + 2 t12 (-4 - 5 α + 2 (1 + t22) α^2))) +
          p22 (2 + dd1 α + 2 t12 α - 2 s^3 (-1 + t12)^2 (-1 + t22) (1 + α) +
             α (dd1 (1 + α) - (-2 + t12 + t22) (1 + t12 α)) + s^2 (6 + 6 α + 2 dd1 α + dd1 α^2 -
                t22 (4 + (5 + 4 dd1) α + 2 dd1 α^2) + t12^2 (2 + α + 2 (-1 + t22) α^2) + t12
```

```
              (-8 + (-7 + 2 dd1) α + (2 + dd1) α^2 + t22 (4 + 5 α - 2 α^2))) + s (t12^2 α (-1 +
                (-3 + 2 t22) α) + t12 (-4 + (-2 + 2 dd1 + t22) α + (4 + dd1 - 3 t22) α^2) -
              2 (-3 - dd1 α - α (3 + dd1 + dd1 α) + t22 (1 + 2 (1 + dd1) α + dd1 α^2))))) +
      (-1 + m) p12 (-2 - 2 α - 2 dd1 α + 2 dd1 m α - 2 dd2 m α + m t12 α + 2 m p22 t12 α -
         4 m^2 p22 t12 α - m t22 α - 2 m p22 t22 α + 4 m^2 p22 t22 α - dd1 α^2 +
         dd1 m α^2 - dd2 m α^2 - 2 t12 α^2 + 2 m t12 α^2 + m p22 t12 α^2 -
         2 m^2 p22 t12 α^2 + 2 t12^2 α^2 - 3 m t12^2 α^2 + m^2 t12^2 α^2 - 2 m t22 α^2 -
         m p22 t22 α^2 + 2 m^2 p22 t22 α^2 + 3 m t12 t22 α^2 - 2 m^2 t12 t22 α^2 +
         m^2 t22^2 α^2 + 2 s^3 (-1 + t12) (-1 + t12 - m t12 + m t22)^2 (1 + α) -
         s^2 (6 + 6 α + 2 dd1 α + dd1 α^2 + 2 (-1 + m)^2 t12^3 α^2 + m^2 t22 ((-dd1 + dd2 - 2 p22) α
                (2 + α) + t22 (2 + α + 2 p22 α + (-2 + p22) α^2)) + m ((-dd1 + dd2) α (2 + α) +
              t22 (-8 + (-7 + 2 dd1 + 2 p22) α + (2 + dd1 + p22) α^2)) -
            (-1 + m) t12^2 (6 + 6 α - 4 α^2 + m (-2 + (-1 + 2 p22) α + (2 + p22 + 4 t22) α^2)) +
            t12 (-12 - 12 α - 2 dd1 α + 2 α^2 - dd1 α^2 + m^2 (2 t22^2 α^2 + (dd1 - dd2 + 2 p22)
                    α (2 + α) + t22 (-4 - 2 α + 4 α^2)) - m (-8 + (-7 + 2 dd2 + 2 p22) α +
                   (2 + dd2 + p22) α^2 + t22 (-8 + (-7 + 2 p22) α + (6 + p22) α^2)))) +
         s (-2 (-1 + m)^2 t12^3 α^2 + m^2 t22 α (8 p22 + t22 - 2 p22 t22 + 4 p22 α +
              3 t22 α - p22 t22 α + dd1 (2 + α) - dd2 (2 + α)) -
            2 (3 + dd1 α + α (3 + dd1 + dd1 α)) - m (-2 (dd1 - dd2) α (2 + α) +
              t22 (-4 + 2 (-1 + dd1 + 2 p22) α + (4 + dd1 + 2 p22) α^2)) +
            (-1 + m) t12^2 α (-6 α + m (1 + 3 α + 4 t22 α + p22 (2 + α))) +
            t12 (6 + 6 α + 2 dd1 α - 4 α^2 + dd1 α^2 + m (-4 + (-2 + 2 dd2 + t22 + 2 p22 (2 + t22))
                   α + (4 + dd2 + 9 t22 + p22 (2 + t22)) α^2) - m^2 α (dd1 (2 + α) -
                  dd2 (2 + α) + 2 (2 p22 (2 + α) + t22 (1 + (3 + t22) α))))))) /
   (2 (1 + s (1 + (-1 + m) t12 - m t22)) (1 + (1 + (-1 + m) t12 - m t22) α +
        s (1 + (-1 + m) t12 - m t22) (1 + α))
      (1 + s (1 + (-1 + m) t12 - m t22) + m t22 α + t12 (α - m α))),
 -t12 - (((-1 + m) t12 - m t22) (2 + p12 α - m p12 α + m p22 α + 2 t12 α -
        2 m t12 α - p12 t12 α + 2 m p12 t12 α - m^2 p12 t12 α -
        m p22 t12 α + m^2 p22 t12 α + 2 m t22 α - m p12 t22 α +
        m^2 p12 t22 α - m^2 p22 t22 α + s (1 + (-1 + m) t12 - m t22)
          (4 + (2 + 2 dd1 (-1 + m) - 2 dd2 m + 4 p12 - 4 m p12 + 4 m p22 + t12 - m
                t12 - 2 p12 t12 + 2 m p12 t12 + m t22 - 2 m p22 t22) α +
             (dd1 (-1 + m) - dd2 m + 2 p12 - 2 m p12 + 2 m p22 + t12 - m t12 - p12
                t12 + m p12 t12 + m t22 - m p22 t22) α^2) - (1 + (-1 + m) t12 - m t22)
          α (-1 - t12 α + (-1 + m) p12 (1 + α) - m (p22 + p22 α - t12 α + t22 α)) +
        s^2 (1 + (-1 + m) t12 - m t22) (2 + α) (1 - dd1 α + p12 α + (-1 + m) t12 (1 + p12 α) -
           m (t22 + (-dd1 + dd2 + p12 - p22) α + p22 t22 α)))) /
   (2 (1 + s (1 + (-1 + m) t12 - m t22)) (1 + (1 + (-1 + m) t12 - m t22) α +
        s (1 + (-1 + m) t12 - m t22) (1 + α))
      (1 + s (1 + (-1 + m) t12 - m t22) + m t22 α + t12 (α - m α))),
```

$$\frac{1}{4}\left(-4\,dd1-\frac{1}{\left(1+s\left(1+(-1+m)\,t12-m\,t22\right)\right)^4}\,(1+s)\right.$$

```
      (-1 + s (-1 + t12 - m t12 + m t22))^2
      (-(((dd1 (-1 + m) - dd2 m - p12 t12 + m p12 t12 - m p22 t22) (dd1 (-1 + m) -
                dd2 m + p12 - m p12 + m p22 - p12 t12 + m p12 t12 - m p22 t22) (1 + α)) /
            (-1 + s (-1 + t12 - m t12 + m t22) + (-1 + m) t12 α - m t22 α)) -
         ((-1 + r) (dd1 (-1 + m) - dd2 m + p12 - m p12 + m p22 - p12 t12 + m p12 t12 - m p22 t22)
```

```
(dd1 (-1 + m) - dd2 m + t12 - m t12 - p12 t12 + m p12 t12 + m t22 - m p22 t22)
   (1 + α)) / (-1 + s (-1 + t12 - m t12 + m t22) + (-1 + m) t12 α - m t22 α) -
 (r (dd1 (-1 + m) - dd2 m - p12 t12 + m p12 t12 - m p22 t22) (-1 + dd1 (-1 + m) - dd2 m +
      p12 - m p12 + m p22 + t12 - m t12 - p12 t12 + m p12 t12 + m t22 - m p22 t22)) /
  (1 + (1 + (-1 + m) t12 - m t22) α + s (1 + (-1 + m) t12 - m t22) (1 + α)) +
 ((-1 + r) (dd1 (-1 + m) - dd2 m + p12 - m p12 + m p22 - p12 t12 + m p12 t12 - m p22 t22)
    (dd1 (-1 + m) - dd2 m + t12 - m t12 - p12 t12 + m p12 t12 + m t22 - m p22 t22) (1 +
      α)) / (1 + (1 + (-1 + m) t12 - m t22) α + s (1 + (-1 + m) t12 - m t22) (1 + α)) +
 ((1 + s) (dd1 (-1 + m) - dd2 m + p12 - m p12 + m p22 - p12 t12 + m p12 t12 - m p22 t22)
    (-1 + dd1 (-1 + m) - dd2 m + p12 - m p12 + m p22 + t12 -
      m t12 - p12 t12 + m p12 t12 + m t22 - m p22 t22) (1 + α)) /
  (1 + (1 + (-1 + m) t12 - m t22) α + s (1 + (-1 + m) t12 - m t22) (1 + α)) +
 ((dd1 (-1 + m) - dd2 m - p12 t12 + m p12 t12 - m p22 t22)
    (dd1 (-1 + m) - dd2 m + p12 - m p12 + m p22 - p12 t12 + m p12 t12 - m p22 t22)) /
  (1 + s (1 + (-1 + m) t12 - m t22) + m t22 α + t12 (α - m α)) -
 (r (dd1 (-1 + m) - dd2 m - p12 t12 + m p12 t12 - m p22 t22) (-1 + dd1 (-1 + m) - dd2 m +
      p12 - m p12 + m p22 + t12 - m t12 - p12 t12 + m p12 t12 + m t22 - m p22 t22)) /
  (1 + s (1 + (-1 + m) t12 - m t22) + m t22 α + t12 (α - m α)) +
 ((1 + s) (dd1 (-1 + m) - dd2 m + p12 - m p12 + m p22 - p12 t12 + m p12 t12 - m p22 t22)
    (-1 + dd1 (-1 + m) - dd2 m + p12 - m p12 + m p22 + t12 -
      m t12 - p12 t12 + m p12 t12 + m t22 - m p22 t22)) /
  (1 + s (1 + (-1 + m) t12 - m t22) + m t22 α + t12 (α - m α)) - (2 (1 + s)
    (dd1 - dd1 m + dd2 m - p12 + m p12 - m p22 + p12 t12 - m p12 t12 + m p22 t22)^2) /
  (1 + s (1 + (-1 + m) t12 - m t22) + m t22 α + t12 (α - m α)))
(- (((dd1 (-1 + m) - dd2 m - p12 t12 + m p12 t12 - m p22 t22) (dd1 (-1 + m) -
        dd2 m + t12 - m t12 - p12 t12 + m p12 t12 + m t22 - m p22 t22) (1 + α)) /
    (-1 + s (-1 + t12 - m t12 + m t22) + (-1 + m) t12 α - m t22 α)) +
 ((dd1 (-1 + m) - dd2 m - p12 t12 + m p12 t12 - m p22 t22)
    (dd1 (-1 + m) - dd2 m + t12 - m t12 - p12 t12 + m p12 t12 + m t22 - m p22 t22)) /
  (1 + (1 + (-1 + m) t12 - m t22) α + s (1 + (-1 + m) t12 - m t22) (1 + α)) -
 (r (1 + s) (dd1 (-1 + m) - dd2 m - p12 t12 + m p12 t12 - m p22 t22)
    (-1 + dd1 (-1 + m) - dd2 m + p12 - m p12 + m p22 + t12 -
      m t12 - p12 t12 + m p12 t12 + m t22 - m p22 t22)) /
  (1 + (1 + (-1 + m) t12 - m t22) α + s (1 + (-1 + m) t12 - m t22) (1 + α)) +
 ((1 + s) (dd1 (-1 + m) - dd2 m + t12 - m t12 - p12 t12 + m p12 t12 + m t22 - m p22 t22)
    (-1 + dd1 (-1 + m) - dd2 m + p12 - m p12 + m p22 + t12 -
      m t12 - p12 t12 + m p12 t12 + m t22 - m p22 t22)) /
  (1 + (1 + (-1 + m) t12 - m t22) α + s (1 + (-1 + m) t12 - m t22) (1 + α)) -
 (2 (dd1 - dd1 m + dd2 m - t12 + m t12 + p12 t12 - m p12 t12 - m t22 + m p22 t22)^2) /
  (1 + (1 + (-1 + m) t12 - m t22) α + s (1 + (-1 + m) t12 - m t22) (1 + α)) +
 ((-1 + r) (1 + s) (dd1 (-1 + m) - dd2 m + p12 - m p12 + m p22 - p12 t12 +
      m p12 t12 - m p22 t22) (dd1 (-1 + m) - dd2 m + t12 -
      m t12 - p12 t12 + m p12 t12 + m t22 - m p22 t22) (1 + α)) /
  (1 + (1 + (-1 + m) t12 - m t22) α + s (1 + (-1 + m) t12 - m t22) (1 + α)) +
 ((1 + s) (dd1 (-1 + m) - dd2 m + t12 - m t12 - p12 t12 + m p12 t12 + m t22 - m p22 t22)
    (-1 + dd1 (-1 + m) - dd2 m + p12 - m p12 + m p22 + t12 -
      m t12 - p12 t12 + m p12 t12 + m t22 - m p22 t22) (1 + α)) /
  (1 + (1 + (-1 + m) t12 - m t22) α + s (1 + (-1 + m) t12 - m t22) (1 + α)) -
 (r (1 + s) (dd1 (-1 + m) - dd2 m - p12 t12 + m p12 t12 - m p22 t22)
```

```
      (-1 + dd1 (-1 + m) - dd2 m + p12 - m p12 + m p22 + t12 -
         m t12 - p12 t12 + m p12 t12 + m t22 - m p22 t22)) /
    (1 + s (1 + (-1 + m) t12 - m t22) + m t22 α + t12 (α - m α)) + ((-1 + r) (1 + s)
      (dd1 (-1 + m) - dd2 m + p12 - m p12 + m p22 - p12 t12 + m p12 t12 - m p22 t22)
      (dd1 (-1 + m) - dd2 m + t12 - m t12 - p12 t12 + m p12 t12 + m t22 - m p22 t22)
      (1 + α)) / (1 + s (1 + (-1 + m) t12 - m t22) + m t22 α + t12 (α - m α))) +
          1
 ----------------------------------- (1 + s) (-1 + s (-1 + t12 - m t12 + m t22))^2
 (1 + s (1 + (-1 + m) t12 - m t22))^4
  ((r (dd1 (-1 + m) - dd2 m + p12 - m p12 + m p22 - p12 t12 + m p12 t12 - m p22 t22)
      (dd1 (-1 + m) - dd2 m + t12 - m t12 - p12 t12 + m p12 t12 + m t22 - m p22 t22)
      (1 + α)) / (-1 + s (-1 + t12 - m t12 + m t22) + (-1 + m) t12 α - m t22 α) +
   ((-1 + r) (dd1 (-1 + m) - dd2 m - p12 t12 + m p12 t12 - m p22 t22)
      (-1 + dd1 (-1 + m) - dd2 m + p12 - m p12 + m p22 + t12 -
         m t12 - p12 t12 + m p12 t12 + m t22 - m p22 t22)) /
    (1 + (1 + (-1 + m) t12 - m t22) α + s (1 + (-1 + m) t12 - m t22) (1 + α)) +
   ((dd1 (-1 + m) - dd2 m + t12 - m t12 - p12 t12 + m p12 t12 + m t22 - m p22 t22)
      (-1 + dd1 (-1 + m) - dd2 m + p12 - m p12 + m p22 + t12 -
         m t12 - p12 t12 + m p12 t12 + m t22 - m p22 t22)) /
    (1 + (1 + (-1 + m) t12 - m t22) α + s (1 + (-1 + m) t12 - m t22) (1 + α)) -
   (r (dd1 (-1 + m) - dd2 m + p12 - m p12 + m p22 - p12 t12 + m p12 t12 - m p22 t22)
      (dd1 (-1 + m) - dd2 m + t12 - m t12 - p12 t12 + m p12 t12 + m t22 - m p22 t22) (1 +
         α)) / (1 + (1 + (-1 + m) t12 - m t22) α + s (1 + (-1 + m) t12 - m t22) (1 + α)) +
   ((1 + s) (dd1 (-1 + m) - dd2 m + p12 - m p12 + m p22 - p12 t12 + m p12 t12 - m p22 t22)
      (-1 + dd1 (-1 + m) - dd2 m + p12 - m p12 + m p22 + t12 -
         m t12 - p12 t12 + m p12 t12 + m t22 - m p22 t22) (1 + α)) /
    (1 + (1 + (-1 + m) t12 - m t22) α + s (1 + (-1 + m) t12 - m t22) (1 + α)) +
   ((dd1 (-1 + m) - dd2 m + t12 - m t12 - p12 t12 + m p12 t12 + m t22 - m p22 t22)
      (-1 + dd1 (-1 + m) - dd2 m + p12 - m p12 + m p22 + t12 -
         m t12 - p12 t12 + m p12 t12 + m t22 - m p22 t22) (1 + α)) /
    (1 + (1 + (-1 + m) t12 - m t22) α + s (1 + (-1 + m) t12 - m t22) (1 + α)) -
   (2 (1 + s) (-1 + dd1 (-1 + m) - dd2 m + p12 - m p12 + m p22 + t12 -
          m t12 - p12 t12 + m p12 t12 + m t22 - m p22 t22)^2 (1 + α)) /
    (1 + (1 + (-1 + m) t12 - m t22) α + s (1 + (-1 + m) t12 - m t22) (1 + α)) +
   ((-1 + r) (dd1 (-1 + m) - dd2 m - p12 t12 + m p12 t12 - m p22 t22)
      (-1 + dd1 (-1 + m) - dd2 m + p12 - m p12 + m p22 + t12 -
         m t12 - p12 t12 + m p12 t12 + m t22 - m p22 t22)) /
    (1 + s (1 + (-1 + m) t12 - m t22) + m t22 α + t12 (α - m α)) +
   ((1 + s) (dd1 (-1 + m) - dd2 m + p12 - m p12 + m p22 - p12 t12 + m p12 t12 - m p22 t22)
      (-1 + dd1 (-1 + m) - dd2 m + p12 - m p12 + m p22 + t12 -
         m t12 - p12 t12 + m p12 t12 + m t22 - m p22 t22)) /
    (1 + s (1 + (-1 + m) t12 - m t22) + m t22 α + t12 (α - m α)))
  (- (((dd1 (-1 + m) - dd2 m - p12 t12 + m p12 t12 - m p22 t22) (dd1 (-1 + m) -
            dd2 m + t12 - m t12 - p12 t12 + m p12 t12 + m t22 - m p22 t22) (1 + α)) /
      (-1 + s (-1 + t12 - m t12 + m t22) + (-1 + m) t12 α - m t22 α)) +
   (2 (dd1 - dd1 m + dd2 m + p12 t12 - m p12 t12 + m p22 t22)^2 (1 + α)) /
    (-1 + s (-1 + t12 - m t12 + m t22) + (-1 + m) t12 α - m t22 α) +
   ((dd1 (-1 + m) - dd2 m - p12 t12 + m p12 t12 - m p22 t22)
      (dd1 (-1 + m) - dd2 m + t12 - m t12 - p12 t12 + m p12 t12 + m t22 - m p22 t22)) /
```

$$
\begin{aligned}
&(1 + (1 + (-1 + m)\, t12 - m\, t22)\, \alpha + s\, (1 + (-1 + m)\, t12 - m\, t22)\, (1 + \alpha)) + \\
&((-1 + r)\, (1 + s)\, (dd1\, (-1 + m) - dd2\, m - p12\, t12 + m\, p12\, t12 - m\, p22\, t22) \\
&\quad (-1 + dd1\, (-1 + m) - dd2\, m + p12 - m\, p12 + m\, p22 + t12 - \\
&\qquad m\, t12 - p12\, t12 + m\, p12\, t12 + m\, t22 - m\, p22\, t22)) / \\
&(1 + (1 + (-1 + m)\, t12 - m\, t22)\, \alpha + s\, (1 + (-1 + m)\, t12 - m\, t22)\, (1 + \alpha)) - \\
&(r\, (1 + s)\, (dd1\, (-1 + m) - dd2\, m + p12 - m\, p12 + m\, p22 - p12\, t12 + m\, p12\, t12 - m\, p22\, t22) \\
&\quad (dd1\, (-1 + m) - dd2\, m + t12 - m\, t12 - p12\, t12 + m\, p12\, t12 + m\, t22 - m\, p22\, t22)\, (1 + \\
&\qquad \alpha)) / (1 + (1 + (-1 + m)\, t12 - m\, t22)\, \alpha + s\, (1 + (-1 + m)\, t12 - m\, t22)\, (1 + \alpha)) + \\
&((1 + s)\, (dd1\, (-1 + m) - dd2\, m - p12\, t12 + m\, p12\, t12 - m\, p22\, t22) \\
&\quad (dd1\, (-1 + m) - dd2\, m + p12 - m\, p12 + m\, p22 - p12\, t12 + m\, p12\, t12 - m\, p22\, t22)) / \\
&(1 + s\, (1 + (-1 + m)\, t12 - m\, t22) + m\, t22\, \alpha + t12\, (\alpha - m\, \alpha)) + \\
&((-1 + r)\, (1 + s)\, (dd1\, (-1 + m) - dd2\, m - p12\, t12 + m\, p12\, t12 - m\, p22\, t22) \\
&\quad (-1 + dd1\, (-1 + m) - dd2\, m + p12 - m\, p12 + m\, p22 + t12 - \\
&\qquad m\, t12 - p12\, t12 + m\, p12\, t12 + m\, t22 - m\, p22\, t22)) / \\
&(1 + s\, (1 + (-1 + m)\, t12 - m\, t22) + m\, t22\, \alpha + t12\, (\alpha - m\, \alpha)) + \\
&((1 + s)\, (dd1\, (-1 + m) - dd2\, m - p12\, t12 + m\, p12\, t12 - m\, p22\, t22) \\
&\quad (dd1\, (-1 + m) - dd2\, m + p12 - m\, p12 + m\, p22 - p12\, t12 + m\, p12\, t12 - m\, p22\, t22) \\
&\quad (1 + \alpha)) / (1 + s\, (1 + (-1 + m)\, t12 - m\, t22) + m\, t22\, \alpha + t12\, (\alpha - m\, \alpha)) - \\
&(r\, (1 + s)\, (dd1\, (-1 + m) - dd2\, m + p12 - m\, p12 + m\, p22 - p12\, t12 + m\, p12\, t12 - m\, p22\, t22) \\
&\quad (dd1\, (-1 + m) - dd2\, m + t12 - m\, t12 - p12\, t12 + m\, p12\, t12 + m\, t22 - m\, p22\, t22) \\
&\quad (1 + \alpha)) / (1 + s\, (1 + (-1 + m)\, t12 - m\, t22) + m\, t22\, \alpha + t12\, (\alpha - m\, \alpha)))\Big), \\
&-p22 + (p22\, (1 + s\, t22)\, (2 + 2\, (1 + dd2)\, \alpha + (dd2 - 2\, (-1 + t22)\, t22)\, \alpha^2 + \\
&\quad 2\, s^2\, t22^2\, (1 + \alpha) + s\, (-2\, t22^2\, \alpha^2 + dd2\, \alpha\, (2 + \alpha) + 2\, t22\, (2 + 2\, \alpha + \alpha^2))) + \\
&\quad dd2\, (2\, s^3\, t22^2\, (1 + \alpha) - \alpha\, (1 + t22\, \alpha) + s\, (2 + (1 + 2\, dd2 - t22)\, \alpha + (dd2 - 2\, t22^2)\, \alpha^2) + \\
&\qquad s^2\, (-2\, t22^2\, \alpha^2 + dd2\, \alpha\, (2 + \alpha) + t22\, (4 + 3\, \alpha + \alpha^2))) - \\
&\quad m^3\, (t12 - t22)\, (p12^2\, (1 + s)\, (1 + s\, t12)\, \alpha\, (2 + \alpha) + p22^2\, (1 + s)\, (1 + s\, t22)\, \alpha\, (2 + \alpha) - \\
&\qquad 2\, (dd1 - dd2)\, s\, (t12 - t22)\, (s - \alpha)\, (s + \alpha + s\, \alpha) + \\
&\qquad p22\, ((-t12 + t22)\, \alpha^2 + 2\, s^3\, (t12 - t22)\, t22\, (1 + \alpha) - \\
&\qquad\quad s\, \alpha\, (dd1\, (2 + \alpha) - dd2\, (2 + \alpha) + (t12 - t22)\, (-1 + 2\, t22\, \alpha)) + s^2\, (2\, t22^2\, \alpha^2 - \\
&\qquad\qquad (dd1 - dd2)\, \alpha\, (2 + \alpha) - t22\, (1 + \alpha)\, (2 + \alpha) + t12\, (2 + 3\, \alpha + (1 - 2\, t22)\, \alpha^2))) - \\
&\qquad p12\, (2\, s^3\, t12\, (t12 - t22)\, (1 + \alpha) + s\, \alpha\, (4\, p22 + t12 + 2\, p22\, t12 - t22 + \\
&\qquad\qquad 2\, p22\, t22 + 2\, p22\, \alpha + p22\, t12\, \alpha - 2\, t12^2\, \alpha + p22\, t22\, \alpha + 2\, t12\, t22\, \alpha - \\
&\qquad\qquad dd1\, (2 + \alpha) + dd2\, (2 + \alpha)) + \alpha\, ((-t12 + t22)\, \alpha + 2\, p22\, (2 + \alpha)) + \\
&\qquad\quad s^2\, (-2\, t12^2\, \alpha^2 + t12\, (2 + (3 + 2\, p22)\, \alpha + (1 + p22 + 2\, t22)\, \alpha^2) + \\
&\qquad\qquad (2 + \alpha)\, ((-dd1 + dd2)\, \alpha + t22\, (-1 + (-1 + p22)\, \alpha)))))) + \\
&\quad m\, (2\, dd1\, s - 2\, dd2\, s + 4\, dd2\, s^2\, t12 + 4\, dd1\, s^2\, t22 - 8\, dd2\, s^2\, t22 + 4\, dd2\, s^3\, t12\, t22 + \\
&\qquad 2\, dd1\, s^3\, t22^2 - 6\, dd2\, s^3\, t22^2 - dd1\, \alpha + dd2\, \alpha + dd1\, s\, \alpha - dd2\, s\, \alpha + \\
&\qquad 4\, dd1\, dd2\, s\, \alpha - 4\, dd2^2\, s\, \alpha + 4\, dd1\, dd2\, s^2\, \alpha - 4\, dd2^2\, s^2\, \alpha - dd2\, s\, t12\, \alpha + \\
&\qquad 3\, dd2\, s^2\, t12\, \alpha - dd1\, s\, t22\, \alpha + 2\, dd2\, s\, t22\, \alpha + 3\, dd1\, s^2\, t22\, \alpha - 6\, dd2\, s^2\, t22\, \alpha + \\
&\qquad 4\, dd2\, s^3\, t12\, t22\, \alpha + 2\, dd1\, s^3\, t22^2\, \alpha - 6\, dd2\, s^3\, t22^2\, \alpha + 2\, dd1\, dd2\, s\, \alpha^2 - \\
&\qquad 2\, dd2^2\, s\, \alpha^2 + 2\, dd1\, dd2\, s^2\, \alpha^2 - 2\, dd2^2\, s^2\, \alpha^2 - dd2\, t12\, \alpha^2 + dd2\, s^2\, t12\, \alpha^2 - \\
&\qquad dd1\, t22\, \alpha^2 + 2\, dd2\, t22\, \alpha^2 + dd1\, s^2\, t22\, \alpha^2 - 2\, dd2\, s^2\, t22\, \alpha^2 - 4\, dd2\, s\, t12\, t22\, \alpha^2 - \\
&\qquad 4\, dd2\, s^2\, t12\, t22\, \alpha^2 - 2\, dd1\, s\, t22^2\, \alpha^2 + 6\, dd2\, s\, t22^2\, \alpha^2 - 2\, dd1\, s^2\, t22^2\, \alpha^2 + \\
&\qquad 6\, dd2\, s^2\, t22^2\, \alpha^2 - p22^2\, (1 + s)\, (t12 - t22)\, (1 + s\, t22)\, \alpha\, (2 + \alpha) + \\
&\qquad p22\, (-2 - 2\, \alpha + 2\, dd1\, \alpha - 4\, dd2\, \alpha + t12\, \alpha - t22\, \alpha + dd1\, \alpha^2 - 2\, dd2\, \alpha^2 + 2\, t12\, \alpha^2 - \\
&\qquad\quad 4\, t22\, \alpha^2 - 3\, t12\, t22\, \alpha^2 + 5\, t22^2\, \alpha^2 + 2\, s^3\, (2\, t12 - 3\, t22)\, t22^2\, (1 + \alpha) - \\
&\qquad\quad s\, (t22^2\, \alpha - 6\, t22^3\, \alpha^2 - (dd1 - 2\, dd2)\, \alpha\, (2 + \alpha) + \\
&\qquad\qquad t22\, (10 + (11 - 2\, dd1 + 2\, dd2)\, \alpha + (4 - dd1 + dd2)\, \alpha^2) + \\
&\qquad\qquad t12\, (-4 + (-5 + 2\, dd2 - t22)\, \alpha + (-2 + dd2 + 4\, t22^2)\, \alpha^2)) -
\end{aligned}
$$

```
            s^2 (t12 (4 t22^2 α^2 + dd2 α (2 + α) - t22 (8 + 9 α + 3 α^2)) +
                t22 (-6 t22^2 α^2 - (dd1 - dd2) α (2 + α) + t22 (14 + 15 α + 5 α^2))))) +
        p12 (2 + 2 α + 2 dd2 α - t12 α + 2 p22 t12 α + t22 α - 2 p22 t22 α + dd2 α^2 +
            p22 t12 α^2 + 2 t22 α^2 - p22 t22 α^2 - t12 t22 α^2 - t22^2 α^2 + 2 s^3 t12 t22^2 (1 + α) +
            s (dd2 α (2 + α) - t22 (-4 + (-5 + 2 dd2 + 2 p22) α + (-2 + dd2 + p22) α^2) +
                t12 (2 + (1 + 4 dd2 - t22 + 2 p22 (1 + t22)) α +
                    (2 dd2 + p22 + p22 t22 - 2 t22^2) α^2) - t22^2 α (-1 + p22 (2 + α))) +
            s^2 (-t22 (2 + α) (dd2 α + t22 (-1 + (-1 + p22) α)) + t12 (-2 t22^2 α^2 +
                    2 dd2 α (2 + α) + t22 (4 + (2 + p22) α + (1 + p22) α (1 + α)))))) +
    m^2 (4 dd1 s^2 t12 - 4 dd2 s^2 t12 + 2 dd2 s^3 t12^2 - 4 dd1 s^2 t22 + 4 dd2 s^2 t22 +
        4 dd1 s^3 t12 t22 - 8 dd2 s^3 t12 t22 - 4 dd1 s^3 t22^2 + 6 dd2 s^3 t22^2 +
        2 dd1^2 s α - 4 dd1 dd2 s α + 2 dd2^2 s α + 2 dd1^2 s^2 α - 4 dd1 dd2 s^2 α +
        2 dd2^2 s^2 α - dd1 s t12 α + dd2 s t12 α + 3 dd1 s^2 t12 α - 3 dd2 s^2 t12 α +
        2 dd2 s^3 t12^2 α + dd1 s t22 α - dd2 s t22 α - 3 dd1 s^2 t22 α +
        3 dd2 s^2 t22 α + 4 dd1 s^3 t12 t22 α - 8 dd2 s^3 t12 t22 α -
        4 dd1 s^3 t22^2 α + 6 dd2 s^3 t22^2 α + dd1^2 s α^2 - 2 dd1 dd2 s α^2 +
        dd2^2 s α^2 + dd1^2 s^2 α^2 - 2 dd1 dd2 s^2 α^2 + dd2^2 s^2 α^2 - dd1 t12 α^2 +
        dd2 t12 α^2 + dd1 s^2 t12 α^2 - dd2 s^2 t12 α^2 - 2 dd2 s t12^2 α^2 -
        2 dd2 s^2 t12^2 α^2 + dd1 t22 α^2 - dd2 t22 α^2 - dd1 s^2 t22 α^2 + dd2 s^2 t22 α^2 -
        4 dd1 s t12 t22 α^2 + 8 dd2 s t12 t22 α^2 - 4 dd1 s^2 t12 t22 α^2 +
        8 dd2 s^2 t12 t22 α^2 + 4 dd1 s t22^2 α^2 - 6 dd2 s t22^2 α^2 + 4 dd1 s^2 t22^2 α^2 -
        6 dd2 s^2 t22^2 α^2 + p12^2 (1 + s) (1 + s t12) (t12 - t22) α (2 + α) +
        2 p22^2 (1 + s) (t12 - t22) (1 + s t22) α (2 + α) +
        p22 (2 s^3 t22 (t12^2 - 4 t12 t22 + 3 t22^2) (1 + α) -
            α (dd1 (2 + α) - dd2 (2 + α) + (t12 - t22) (1 + (2 + t12 - 4 t22) α)) -
            s (-2 t22^2 α + 6 t22^3 α^2 + (dd1 - dd2) α (2 + α) + t12^2 α (-1 + 2 t22 α) + t22
                    (-4 + (-5 + 2 dd2) α + (-2 + dd2) α^2) + t12 (4 + (5 + 2 dd1 - 4 dd2 + 3 t22)
                        α + (2 + dd1 - 2 dd2 - 8 t22^2) α^2)) + s^2 (t12^2 (2 + 3 α + (1 - 2 t22) α^2) +
                t22 (-6 t22^2 α^2 - dd2 α (2 + α) + 2 t22 (5 + 6 α + 2 α^2)) +
                t12 (8 t22^2 α^2 - (dd1 - 2 dd2) α (2 + α) - t22 (12 + 15 α + 5 α^2)))) +
        p12 (2 dd1 α - 2 dd2 α + t12 α - 6 p22 t12 α - t22 α + 6 p22 t22 α + dd1 α^2 -
            dd2 α^2 + 2 t12 α^2 - 3 p22 t12 α^2 - t12^2 α^2 - 2 t22 α^2 + 3 p22 t22 α^2 - t12 t22 α^2 +
            2 t22^2 α^2 + 4 s^3 t12 (t12 - t22) t22 (1 + α) - s (-(dd1 - dd2) α (2 + α) +
                t22 (4 + (5 + 2 dd1 - 4 dd2 - 6 p22) α + (2 + dd1 - 2 dd2 - 3 p22) α^2) +
                t12 (-4 + (-5 - 4 dd1 + 6 dd2 + 6 p22 - 3 t22 + 2 p22 t22) α +
                    (-2 - 2 dd1 + 3 dd2 + 3 p22 + p22 t22 - 4 t22^2) α^2) -
                2 t22^2 α (-1 + p22 (2 + α)) + t12^2 α (1 + 4 t22 α + p22 (2 + α))) -
            s^2 (t12^2 (-4 + (-3 + 2 p22) α + (-1 + p22 + 4 t22) α^2) +
                t22 (2 + α) ((dd1 - 2 dd2) α + t22 (2 - 2 (-1 + p22) α)) - t12 α
                    (2 dd1 (2 + α) - 3 dd2 (2 + α) + t22 (3 + α + 4 t22 α - p22 (2 + α))))))) /
  (2 (1 + m s (t12 - t22) + s t22) (1 + s t22 + m (t12 - t22) (s - α) + α - t22 α)
    (1 + t22 α + s t22 (1 + α) + m (t12 - t22) (s + α + s α))),
-t22 + ((1 + s) (m (t12 - t22) + t22) (2 + α + 2 p22 α + t22 α - 2 p22 t22 α +
        p22 α^2 + t22 α^2 - p22 t22 α^2 - t22^2 α^2 + 2 s^2 t22^2 (1 + α) +
        s (dd2 α (2 + α) + t22 (4 + (3 - 2 dd2 + 2 p22) α + (1 - dd2 + p22) α^2) -
            t22^2 α (-1 + α + p22 (2 + α))) + m^2 (t12 - t22) (2 s^2 (t12 - t22) (1 + α) +
            s α (t12 - 2 p12 t12 - t22 + 2 p22 t22 - t12 α - p12 t12 α + t22 α + p22 t22 α - dd1
                (2 + α) + dd2 (2 + α)) + α ((-t12 + t22) α - p12 (2 + α) + p22 (2 + α))) +
        m (4 s^2 (t12 - t22) t22 (1 + α) - α (p22 (1 + t12 - 2 t22) (2 + α) + p12
                (-1 + t22) (2 + α) + (t12 - t22) (-1 - α + 2 t22 α)) -
            s (-(dd1 - dd2) α (2 + α) + t22 (4 + (3 + 2 dd1 - 4 dd2 + 2 p22) α +
```

$$
\begin{aligned}
&(1+dd1-2\,dd2+p22)\,\alpha^2)+t12\,(-4+(-3+2\,dd2+2\,p12\,(-1+t22)-\\
&\quad 2\,t22+2\,p22\,t22)\,\alpha+(-1+dd2+p12\,(-1+t22)+2\,t22+\\
&\quad p22\,t22)\,\alpha^2)-2\,t22^2\,\alpha\,(-1+\alpha+p22\,(2+\alpha))))))\,/\\
&(2\,(1+m\,s\,(t12-t22)+s\,t22)\,(1+s\,t22+m\,(t12-t22)\,(s-\alpha)+\alpha-t22\,\alpha)\\
&\quad(1+t22\,\alpha+s\,t22\,(1+\alpha)+m\,(t12-t22)\,(s+\alpha+s\,\alpha))),\\
&\frac{1}{4}\left(-4\,dd2-\frac{1}{(1+m\,s\,(t12-t22)+s\,t22)^2}\,(1+s)\right.\\
&\quad((2\,(1+s)\,(dd2+dd1\,m-dd2\,m-m\,t12+m\,p12\,t12-t22+m\,t22+p22\,t22-\\
&\quad\quad m\,p22\,t22)^2)\,/\,(1+s\,t22+m\,(t12-t22)\,(s-\alpha)+\alpha-t22\,\alpha)-\\
&\quad((1+s)\,(dd2\,(-1+m)-dd1\,m-m\,p12\,t12-p22\,t22+m\,p22\,t22)\\
&\quad\quad(dd2\,(-1+m)-dd1\,m+m\,t12-m\,p12\,t12+t22-m\,t22-p22\,t22+m\,p22\,t22))\,/\\
&\quad\quad(1+s\,t22+m\,(t12-t22)\,(s-\alpha)+\alpha-t22\,\alpha)+\\
&\quad(r\,(dd2\,(-1+m)-dd1\,m-m\,p12\,t12-p22\,t22+m\,p22\,t22)\\
&\quad\quad(-1+dd2\,(-1+m)-dd1\,m+m\,p12+p22-m\,p22+m\,t12-m\,p12\,t12+t22-m\,t22-\\
&\quad\quad p22\,t22+m\,p22\,t22))\,/\,(1+s\,t22+m\,(t12-t22)\,(s-\alpha)+\alpha-t22\,\alpha)-\\
&\quad((dd2\,(-1+m)-dd1\,m+m\,t12-m\,p12\,t12+t22-m\,t22-p22\,t22+m\,p22\,t22)\\
&\quad\quad(-1+dd2\,(-1+m)-dd1\,m+m\,p12+p22-m\,p22+m\,t12-m\,p12\,t12+t22-m\,t22-\\
&\quad\quad p22\,t22+m\,p22\,t22))\,/\,(1+s\,t22+m\,(t12-t22)\,(s-\alpha)+\alpha-t22\,\alpha)-\\
&\quad((-1+r)\,(dd2\,(-1+m)-dd1\,m+m\,p12+p22-m\,p22-m\,p12\,t12-p22\,t22+m\,p22\,t22)\\
&\quad\quad(dd2\,(-1+m)-dd1\,m+m\,t12-m\,p12\,t12+t22-m\,t22-p22\,t22+m\,p22\,t22)\\
&\quad\quad(1+\alpha))\,/\,(1+s\,t22+m\,(t12-t22)\,(s-\alpha)+\alpha-t22\,\alpha)-\\
&\quad(1\,/\,(1+s\,t22+m\,(t12-t22)\,(s-\alpha)+\alpha-t22\,\alpha))\\
&\quad\quad(dd2\,(-1+m)-dd1\,m+m\,t12-m\,p12\,t12+t22-m\,t22-p22\,t22+m\,p22\,t22)\\
&\quad\quad(-1+dd2\,(-1+m)-dd1\,m+m\,p12+p22-m\,p22+m\,t12-\\
&\quad\quad m\,p12\,t12+t22-m\,t22-p22\,t22+m\,p22\,t22)\,(1+\alpha)+\\
&\quad(r\,(dd2\,(-1+m)-dd1\,m-m\,p12\,t12-p22\,t22+m\,p22\,t22)\,(-1+dd2\,(-1+m)-dd1\,m+\\
&\quad\quad m\,p12+p22-m\,p22+m\,t12-m\,p12\,t12+t22-m\,t22-p22\,t22+m\,p22\,t22))\,/\\
&\quad\quad(1+t22\,\alpha+s\,t22\,(1+\alpha)+m\,(t12-t22)\,(s+\alpha+s\,\alpha))-\\
&\quad((1+s)\,(dd2\,(-1+m)-dd1\,m-m\,p12\,t12-p22\,t22+m\,p22\,t22)\\
&\quad\quad(dd2\,(-1+m)-dd1\,m+m\,t12-m\,p12\,t12+t22-m\,t22-p22\,t22+m\,p22\,t22)\\
&\quad\quad(1+\alpha))\,/\,(1+t22\,\alpha+s\,t22\,(1+\alpha)+m\,(t12-t22)\,(s+\alpha+s\,\alpha))-\\
&\quad((-1+r)\,(dd2\,(-1+m)-dd1\,m+m\,p12+p22-m\,p22-m\,p12\,t12-p22\,t22+m\,p22\,t22)\\
&\quad\quad(dd2\,(-1+m)-dd1\,m+m\,t12-m\,p12\,t12+t22-m\,t22-p22\,t22+m\,p22\,t22)\\
&\quad\quad(1+\alpha))\,/\,(1+t22\,\alpha+s\,t22\,(1+\alpha)+m\,(t12-t22)\,(s+\alpha+s\,\alpha)))\\
&\quad((r\,(1+s)\,(dd2\,(-1+m)-dd1\,m-m\,p12\,t12-p22\,t22+m\,p22\,t22)\\
&\quad\quad(-1+dd2\,(-1+m)-dd1\,m+m\,p12+p22-m\,p22+m\,t12-\\
&\quad\quad m\,p12\,t12+t22-m\,t22-p22\,t22+m\,p22\,t22))\,/\\
&\quad\quad(1+s\,t22+m\,(t12-t22)\,(s-\alpha)+\alpha-t22\,\alpha)-((-1+r)\,(1+s)\\
&\quad\quad(dd2\,(-1+m)-dd1\,m+m\,p12+p22-m\,p22-m\,p12\,t12-p22\,t22+m\,p22\,t22)\\
&\quad\quad(dd2\,(-1+m)-dd1\,m+m\,t12-m\,p12\,t12+t22-m\,t22-p22\,t22+m\,p22\,t22)\\
&\quad\quad(1+\alpha))\,/\,(1+s\,t22+m\,(t12-t22)\,(s-\alpha)+\alpha-t22\,\alpha)-\\
&\quad(1\,/\,(1+s\,t22+m\,(t12-t22)\,(s-\alpha)+\alpha-t22\,\alpha))\\
&\quad\quad(dd2\,(-1+m)-dd1\,m+m\,p12+p22-m\,p22-m\,p12\,t12-p22\,t22+m\,p22\,t22)\\
&\quad\quad(-1+dd2\,(-1+m)-dd1\,m+m\,p12+p22-m\,p22+m\,t12-\\
&\quad\quad m\,p12\,t12+t22-m\,t22-p22\,t22+m\,p22\,t22)\,(1+\alpha)+\\
&\quad(2\,(dd2+dd1\,m-dd2\,m-m\,p12-p22+m\,p22+m\,p12\,t12+p22\,t22-m\,p22\,t22)^2)\,/\\
&\quad\quad(1+t22\,\alpha+s\,t22\,(1+\alpha)+m\,(t12-t22)\,(s+\alpha+s\,\alpha))-\\
&\quad((1+s)\,(dd2\,(-1+m)-dd1\,m-m\,p12\,t12-p22\,t22+m\,p22\,t22)
\end{aligned}
$$

$$
\begin{aligned}
&(dd2\ (-1+m) - dd1\ m + m\ p12 + p22 - m\ p22 - m\ p12\ t12 - p22\ t22 + m\ p22\ t22))\ /\\
&(1 + t22\ \alpha + s\ t22\ (1+\alpha) + m\ (t12 - t22)\ (s + \alpha + s\ \alpha)) +\\
&(r\ (1+s)\ (dd2\ (-1+m) - dd1\ m - m\ p12\ t12 - p22\ t22 + m\ p22\ t22)\\
&(-1 + dd2\ (-1+m) - dd1\ m + m\ p12 + p22 - m\ p22 + m\ t12 - m\ p12\ t12 + t22 - m\ t22 -\\
&p22\ t22 + m\ p22\ t22))\ /\ (1 + t22\ \alpha + s\ t22\ (1+\alpha) + m\ (t12 - t22)\ (s + \alpha + s\ \alpha)) -\\
&((dd2\ (-1+m) - dd1\ m + m\ p12 + p22 - m\ p22 - m\ p12\ t12 - p22\ t22 + m\ p22\ t22)\\
&(-1 + dd2\ (-1+m) - dd1\ m + m\ p12 + p22 - m\ p22 + m\ t12 - m\ p12\ t12 + t22 - m\ t22 -\\
&p22\ t22 + m\ p22\ t22))\ /\ (1 + t22\ \alpha + s\ t22\ (1+\alpha) + m\ (t12 - t22)\ (s + \alpha + s\ \alpha)) -\\
&((1+s)\ (dd2\ (-1+m) - dd1\ m - m\ p12\ t12 - p22\ t22 + m\ p22\ t22)\ (dd2\ (-1+m) -\\
&dd1\ m + m\ p12 + p22 - m\ p22 - m\ p12\ t12 - p22\ t22 + m\ p22\ t22)\ (1+\alpha))\ /\\
&(1 + t22\ \alpha + s\ t22\ (1+\alpha) + m\ (t12 - t22)\ (s + \alpha + s\ \alpha)) - ((-1 + r)\ (1+s)\\
&(dd2\ (-1+m) - dd1\ m + m\ p12 + p22 - m\ p22 - m\ p12\ t12 - p22\ t22 + m\ p22\ t22)\\
&(dd2\ (-1+m) - dd1\ m + m\ t12 - m\ p12\ t12 + t22 - m\ t22 - p22\ t22 + m\ p22\ t22)\\
&(1+\alpha))\ /\ (1 + t22\ \alpha + s\ t22\ (1+\alpha) + m\ (t12 - t22)\ (s + \alpha + s\ \alpha))) +\\
&\frac{1}{(1 + m\ s\ (t12 - t22) + s\ t22)^2}\ (1+s)\ (-(((1+s)\ (dd2\ (-1+m) - dd1\ m - m\ p12\ t12 -\\
&p22\ t22 + m\ p22\ t22)\ (dd2\ (-1+m) - dd1\ m + m\ t12 - m\ p12\ t12 + t22 - m\ t22 -\\
&p22\ t22 + m\ p22\ t22))\ /\ (1 + s\ t22 + m\ (t12 - t22)\ (s - \alpha) + \alpha - t22\ \alpha)) -\\
&((-1 + r)\ (dd2\ (-1+m) - dd1\ m - m\ p12\ t12 - p22\ t22 + m\ p22\ t22)\\
&(-1 + dd2\ (-1+m) - dd1\ m + m\ p12 + p22 - m\ p22 + m\ t12 - m\ p12\ t12 + t22 - m\ t22 -\\
&p22\ t22 + m\ p22\ t22))\ /\ (1 + s\ t22 + m\ (t12 - t22)\ (s - \alpha) + \alpha - t22\ \alpha) +\\
&(r\ (dd2\ (-1+m) - dd1\ m + m\ p12 + p22 - m\ p22 - m\ p12\ t12 - p22\ t22 + m\ p22\ t22)\\
&(dd2\ (-1+m) - dd1\ m + m\ t12 - m\ p12\ t12 + t22 - m\ t22 - p22\ t22 + m\ p22\ t22)\\
&(1+\alpha))\ /\ (1 + s\ t22 + m\ (t12 - t22)\ (s - \alpha) + \alpha - t22\ \alpha) -\\
&((dd2\ (-1+m) - dd1\ m - m\ p12\ t12 - p22\ t22 + m\ p22\ t22)\\
&(dd2\ (-1+m) - dd1\ m + m\ p12 + p22 - m\ p22 - m\ p12\ t12 - p22\ t22 + m\ p22\ t22))\ /\\
&(1 + t22\ \alpha + s\ t22\ (1+\alpha) + m\ (t12 - t22)\ (s + \alpha + s\ \alpha)) -\\
&((-1 + r)\ (dd2\ (-1+m) - dd1\ m - m\ p12\ t12 - p22\ t22 + m\ p22\ t22)\\
&(-1 + dd2\ (-1+m) - dd1\ m + m\ p12 + p22 - m\ p22 + m\ t12 - m\ p12\ t12 + t22 - m\ t22 -\\
&p22\ t22 + m\ p22\ t22))\ /\ (1 + t22\ \alpha + s\ t22\ (1+\alpha) + m\ (t12 - t22)\ (s + \alpha + s\ \alpha)) +\\
&(2\ (1+s)\ (dd2 + dd1\ m - dd2\ m + m\ p12\ t12 + p22\ t22 - m\ p22\ t22)^2\ (1+\alpha))\ /\\
&(1 + t22\ \alpha + s\ t22\ (1+\alpha) + m\ (t12 - t22)\ (s + \alpha + s\ \alpha)) -\\
&((dd2\ (-1+m) - dd1\ m - m\ p12\ t12 - p22\ t22 + m\ p22\ t22)\\
&(dd2\ (-1+m) - dd1\ m + m\ p12 + p22 - m\ p22 - m\ p12\ t12 - p22\ t22 + m\ p22\ t22)\\
&(1+\alpha))\ /\ (1 + t22\ \alpha + s\ t22\ (1+\alpha) + m\ (t12 - t22)\ (s + \alpha + s\ \alpha)) -\\
&((1+s)\ (dd2\ (-1+m) - dd1\ m - m\ p12\ t12 - p22\ t22 + m\ p22\ t22)\\
&(dd2\ (-1+m) - dd1\ m + m\ t12 - m\ p12\ t12 + t22 - m\ t22 - p22\ t22 + m\ p22\ t22)\\
&(1+\alpha))\ /\ (1 + t22\ \alpha + s\ t22\ (1+\alpha) + m\ (t12 - t22)\ (s + \alpha + s\ \alpha)) +\\
&(r\ (dd2\ (-1+m) - dd1\ m + m\ p12 + p22 - m\ p22 - m\ p12\ t12 - p22\ t22 + m\ p22\ t22)\\
&(dd2\ (-1+m) - dd1\ m + m\ t12 - m\ p12\ t12 + t22 - m\ t22 - p22\ t22 + m\ p22\ t22)\\
&(1+\alpha))\ /\ (1 + t22\ \alpha + s\ t22\ (1+\alpha) + m\ (t12 - t22)\ (s + \alpha + s\ \alpha)))\\
&(-(((-1 + r)\ (1+s)\ (dd2\ (-1+m) - dd1\ m - m\ p12\ t12 - p22\ t22 + m\ p22\ t22)\\
&(-1 + dd2\ (-1+m) - dd1\ m + m\ p12 + p22 - m\ p22 + m\ t12 - m\ p12\ t12 + t22 - m\ t22 -\\
&p22\ t22 + m\ p22\ t22))\ /\ (1 + s\ t22 + m\ (t12 - t22)\ (s - \alpha) + \alpha - t22\ \alpha)) -\\
&(1\ /\ (1 + s\ t22 + m\ (t12 - t22)\ (s - \alpha) + \alpha - t22\ \alpha))\ (1+s)\\
&(dd2\ (-1+m) - dd1\ m + m\ t12 - m\ p12\ t12 + t22 - m\ t22 - p22\ t22 + m\ p22\ t22)\\
&(-1 + dd2\ (-1+m) - dd1\ m + m\ p12 + p22 - m\ p22 +\\
&m\ t12 - m\ p12\ t12 + t22 - m\ t22 - p22\ t22 + m\ p22\ t22) +\\
&(r\ (1+s)\ (dd2\ (-1+m) - dd1\ m + m\ p12 + p22 - m\ p22 - m\ p12\ t12 - p22\ t22 + m\ p22\ t22)
\end{aligned}
$$

```
        (dd2 (-1 + m) - dd1 m + m t12 - m p12 t12 + t22 - m t22 - p22 t22 + m p22 t22)
        (1 + α)) / (1 + s t22 + m (t12 - t22) (s - α) + α - t22 α) -
    (1 / (1 + s t22 + m (t12 - t22) (s - α) + α - t22 α))
     (dd2 (-1 + m) - dd1 m + m p12 + p22 - m p22 - m p12 t12 - p22 t22 + m p22 t22)
      (-1 + dd2 (-1 + m) - dd1 m + m p12 + p22 - m p22 + m t12 -
        m p12 t12 + t22 - m t22 - p22 t22 + m p22 t22) (1 + α) -
    (1 / (1 + s t22 + m (t12 - t22) (s - α) + α - t22 α)) (1 + s)
      (dd2 (-1 + m) - dd1 m + m t12 - m p12 t12 + t22 - m t22 - p22 t22 + m p22 t22)
      (-1 + dd2 (-1 + m) - dd1 m + m p12 + p22 - m p22 + m t12 -
        m p12 t12 + t22 - m t22 - p22 t22 + m p22 t22) (1 + α) +
    (2 (-1 + dd2 (-1 + m) - dd1 m + m p12 + p22 - m p22 + m t12 - m p12 t12 +
          t22 - m t22 - p22 t22 + m p22 t22)^2
        (1 + α)) / (1 + s t22 + m (t12 - t22) (s - α) + α - t22 α) -
    ((-1 + r) (1 + s) (dd2 (-1 + m) - dd1 m - m p12 t12 - p22 t22 + m p22 t22)
        (-1 + dd2 (-1 + m) - dd1 m + m p12 + p22 - m p22 + m t12 - m p12 t12 + t22 - m t22 -
          p22 t22 + m p22 t22)) / (1 + t22 α + s t22 (1 + α) + m (t12 - t22) (s + α + s α)) -
    ((dd2 (-1 + m) - dd1 m + m p12 + p22 - m p22 - m p12 t12 - p22 t22 + m p22 t22)
        (-1 + dd2 (-1 + m) - dd1 m + m p12 + p22 - m p22 + m t12 - m p12 t12 + t22 - m t22 -
          p22 t22 + m p22 t22)) / (1 + t22 α + s t22 (1 + α) + m (t12 - t22) (s + α + s α)) +
    (r (1 + s) (dd2 (-1 + m) - dd1 m + m p12 + p22 - m p22 - m p12 t12 - p22 t22 + m p22 t22)
        (dd2 (-1 + m) - dd1 m + m t12 - m p12 t12 + t22 - m t22 - p22 t22 + m p22 t22)
        (1 + α)) / (1 + t22 α + s t22 (1 + α) + m (t12 - t22) (s + α + s α)))};
```

```
Coefficient[deltagensym[[1]], r]
```

0

```
Coefficient[deltagensym[[2]], r]
```

0

```
Exponent[deltagensym[[3]], r]
```

1

```
Coefficient[deltagensym[[3]], r]
```

The following gives the structure of the (numerator of the) components of deltagensym

```
Exponent[Numerator[Factor[deltagensym[[1]]]], {p12, t12, dd1, p22, t22, dd2}]
```

{2, 2, 2, 2, 3, 2}

```
Exponent[Numerator[Factor[deltagensym[[2]]]], {p12, t12, dd1, p22, t22, dd2}]
```

{1, 4, 1, 1, 3, 1}

```
Exponent[Numerator[Factor[deltagensym[[3]]]], {p12, t12, dd1, p22, t22, dd2}]
```

{3, 6, 3, 3, 6, 3}

```
Exponent[Numerator[Factor[deltagensym[[4]]]], {p12, t12, dd1, p22, t22, dd2}]
```

{2, 3, 2, 2, 2, 2}

```
Exponent[Numerator[Factor[deltagensym[[5]]]], {p12, t12, dd1, p22, t22, dd2}]
{1, 3, 1, 1, 4, 1}

Exponent[Numerator[Factor[deltagensym[[6]]]], {p12, t12, dd1, p22, t22, dd2}]
{3, 6, 3, 3, 6, 3}
```

Therefore, deltagensym[[2]] and deltagensym[[5]] can be used to compute p12 and p22 (at equilibrium)

```
Exponent[deltagensym[[3]], r]

Exponent[deltagensym[[6]], r]
1
```

There is symmetry between deme 1 and deme 2, as it should be :

```
Simplify[(deltagensym[[1]] /. {p12 → 1 - p11, t12 → 1 - t11} /.
    {p11 → x2, p22 → x1, t11 → y2, t22 → y1, dd1 → z2, dd2 → z1}) +
  (deltagensym[[4]] /. {p12 → 1 - p11, t12 → 1 - t11} /.
    {p11 → x1, p22 → x2, t11 → y1, t22 → y2, dd1 → z1, dd2 → z2})]

Simplify[(deltagensym[[2]] /. {p12 → 1 - p11, t12 → 1 - t11} /.
    {p11 → x2, p22 → x1, t11 → y2, t22 → y1, dd1 → z2, dd2 → z1}) +
  (deltagensym[[5]] /. {p12 → 1 - p11, t12 → 1 - t11} /.
    {p11 → x1, p22 → x2, t11 → y1, t22 → y2, dd1 → z1, dd2 → z2})]

Simplify[(deltagensym[[3]] /. {p12 → 1 - p11, t12 → 1 - t11} /.
    {p11 → x2, p22 → x1, t11 → y2, t22 → y1, dd1 → z2, dd2 → z1}) -
  (deltagensym[[6]] /. {p12 → 1 - p11, t12 → 1 - t11} /.
    {p11 → x1, p22 → x2, t11 → y1, t22 → y2, dd1 → z1, dd2 → z2})]
0

Simplify[(deltagensym[[3]] /. {p12 → 1 - p11, t12 → 1 - t11} /.
    {p11 → x1, p22 → x2, t11 → y1, t22 → y2, dd1 → z1, dd2 → z2}) -
  (deltagensym[[6]] /. {p12 → 1 - p11, t12 → 1 - t11} /.
    {p11 → x2, p22 → x1, t11 → y2, t22 → y1, dd1 → z2, dd2 → z1})]
0
```

2.3.2 Computing the equilibrium values of (p12,p22) and (dd1,dd2) as functions of (t12,t22) for arbitrary r

The structure of deltagensym (see above) suggests that we can obtain (p12, dd1, p22, dd2) as functions of (t12, t22) by solving {deltagensym[[2]], deltagensym[[5]]} = 0 and ,subsequently, {deltagensym[[1]], deltagensym[[4]]} = 0

```
Simplify[Solve[{deltagensym[[2]], deltagensym[[5]]} == 0, {p12, p22}]]
```

The following is (p12, p22) as a function of (t12, t22, dd1, dd2); it is independent of r:

```
p12p22gentdd =
  {p12 → - (((-1 + t12) t12 (-1 + t22) t22 (1 + s t22) (2 s^3 (-1 + t12)^2 (1 + α) + α (1 + t12 α) +
          s (2 + (4 + 2 dd1 - t12) α + (dd1 + (3 - 2 t12) t12) α^2) +
```

$$s^2 (4 + 5 \alpha + 2\, dd1\, \alpha + dd1\, \alpha^2 - 2\, t12^2 \alpha^2 + t12 (-4 - 5 \alpha + 2 \alpha^2))) +$$
$$m^6 s (t12 - t22)^4 (2 s^3 (t12^2 t22 + (-1 + t22)^2 t22 + t12 (-1 + 2\, t22 - 2\, t22^2)) (1 + \alpha) -$$
$$2 \alpha (-dd1 (2 + \alpha) + dd2 (2 + \alpha) + (t12 - t22) (1 + (-1 + t12 + 2\, t22) \alpha)) +$$
$$s \alpha (3\, dd1 (2 + \alpha) - 3\, dd2 (2 + \alpha) - (t12 - t22)$$
$$(3 + (-3 + 7\, t22 - 2\, t22^2 + 2\, t12 (1 + t22)) \alpha)) +$$
$$s^2 (-2\, t22^3 \alpha^2 + (dd1 - dd2) \alpha (2 + \alpha) + t22 (4 + 5 \alpha - \alpha^2) + 3\, t22^2 (-2 - 2 \alpha + \alpha^2) +$$
$$t12^2 (2 + 2 \alpha - 2\, t22\, \alpha^2) + t12 (-4 - 5 \alpha + \alpha^2 + 4\, t22^2 \alpha^2 + t22 (4 + 4 \alpha - 3 \alpha^2)))) -$$
$$m^5 (t12 - t22)^4 (2 s^4 (4\, t12^2 t22 + (-1 + t22)^2 (1 + 3\, t22) + t12 (-2 + 2\, t22 - 5\, t22^2))$$
$$(1 + \alpha) + 2 \alpha (1 + t22\, \alpha) - 2 s \alpha (-2 + 3\, t12 - 3\, t22 - 3\, t12\, \alpha + 4\, t12^2$$
$$\alpha + t22\, \alpha + t12\, t22\, \alpha - 5\, t22^2 \alpha - 3\, dd1 (2 + \alpha) + 3\, dd2 (2 + \alpha)) -$$
$$s^2 (-8 - 10 \alpha - 16\, dd1\, \alpha + 18\, dd2\, \alpha - 8\, dd1\, \alpha^2 + 9\, dd2\, \alpha^2 - 19\, t22^2 \alpha^2 + 6\, t22^3$$
$$\alpha^2 + 8\, t12^2 (1 + t22) \alpha^2 + t22 (14 - 4 (-1 + dd1) \alpha + (7 - 2\, dd1) \alpha^2) + 2$$
$$t12 (1 + (5 + t22) \alpha + (-4 + 3\, t22 - 5\, t22^2) \alpha^2)) +$$
$$s^3 (8 + 8 \alpha + 4\, dd1\, \alpha - 6\, dd2\, \alpha + 2\, dd1\, \alpha^2 - 3\, dd2\, \alpha^2 - 6\, t22^3 \alpha^2 + 3\, t22^2$$
$$(-4 - 4 \alpha + 3 \alpha^2) + t12^2 (8 + 8 \alpha - 8\, t22\, \alpha^2) + t22 (-4 - 3 \alpha^2 + 2\, dd1\, \alpha (2 + \alpha)) + 2$$
$$t12 (-6 - 7 \alpha + \alpha^2 + 5\, t22^2 \alpha^2 + t22 (2 + \alpha - 2 \alpha^2)))) + m^4 (t12 - t22)^2$$
$$(2 s^4 (6\, t12^4 t22 + 3 (-1 + t22)^2 t22^2 (1 + t22) - t12^3 (1 + 3\, t22 + 18\, t22^2) -$$
$$t12\, t22 (4 + t22 - 7\, t22^2 + 12\, t22^3) + t12^2 (1 + 5\, t22 - t22^2 + 21\, t22^3)) (1 + \alpha) +$$
$$(t12 - t22)^2 \alpha (5 + t12\, \alpha + 4\, t22\, \alpha) + s (-12\, t12^4 \alpha^2 + 8\, t22^4 \alpha^2 - 2$$
$$(dd1 - dd2) \alpha (2 + \alpha) + t22^3 \alpha (7 + 6 \alpha) + t12^3 \alpha (-7 + (7 + 31\, t22) \alpha) +$$
$$t22^2 (-14 + 2 (-1 + 7\, dd1 - 6\, dd2) \alpha + (-6 + 7\, dd1 - 6\, dd2) \alpha^2) + 2$$
$$t22 (4 + (3 + 2\, dd1 - 2\, dd2) \alpha + (1 + dd1 - dd2) \alpha^2) + t12^2$$
$$(2 + (10 + 14\, dd1 - 12\, dd2 + 25\, t22) \alpha +$$
$$(4 + 7\, dd1 - 6\, dd2 - 24\, t22 - 6\, t22^2) \alpha^2) - t12 (t22^2 (25 - 11 \alpha) \alpha +$$
$$21\, t22^3 \alpha^2 + 2\, t22 (-6 + 2 (2 + 9\, dd1 - 8\, dd2) \alpha + (-1 + 9\, dd1 - 8\, dd2) \alpha^2) +$$
$$2 (4 + (3 - 2\, dd1 + 2\, dd2) \alpha + (1 - dd1 + dd2) \alpha^2))) +$$
$$s^3 (-6\, t22^5 \alpha^2 - (dd1 - dd2) \alpha (2 + \alpha) + t22^4 (-6 - 6 \alpha + 7 \alpha^2) + t22^3$$
$$(-22 + 2 (-8 + 5\, dd1) \alpha + (2 + 5\, dd1) \alpha^2) + t22^2$$
$$(12 + (13 + 2\, dd1 - 6\, dd2) \alpha + (-4 + dd1 - 3\, dd2) \alpha^2) + t22$$
$$(4 + (3 + 2\, dd1 - 2\, dd2) \alpha + (1 + dd1 - dd2) \alpha^2) - 12\, t12^4 (-1 - \alpha + t22\, \alpha^2) +$$
$$t12^3 (-14 - 15 \alpha + \alpha^2 + 36\, t22^2 \alpha^2 + t22 (-36 - 41 \alpha + \alpha^2)) + t12^2$$
$$(12 + (11 + 2\, dd1 - 6\, dd2) \alpha + (1 + dd1 - 3\, dd2) \alpha^2 - 42\, t22^3 \alpha^2 +$$
$$t22^2 (14 + 24 \alpha + 11 \alpha^2) + t22 (30 + 10 (4 + dd1) \alpha + (-8 + 5\, dd1) \alpha^2)) +$$
$$t12 (-4 + (-3 + 2\, dd1 - 2\, dd2) \alpha + (-1 + dd1 - dd2) \alpha^2 + 24\, t22^4 \alpha^2 +$$
$$t22^3 (16 + 11 \alpha - 19 \alpha^2) + t22^2 (6 - (9 + 20\, dd1) \alpha + (5 - 10\, dd1) \alpha^2) +$$
$$t22 (-24 - 8 (3 + dd1 - 2\, dd2) \alpha + (3 - 4\, dd1 + 8\, dd2) \alpha^2))) +$$
$$s^2 (15\, t22^4 \alpha^2 - 6\, t22^5 \alpha^2 - 12\, t12^4 (1 + t22) \alpha^2 - 3 (dd1 - dd2) \alpha$$
$$(2 + \alpha) + t22^3 (-26 + (-13 + 10\, dd1) \alpha + (4 + 5\, dd1) \alpha^2) + t22^2$$
$$(-4 + 2 (2 + 8\, dd1 - 9\, dd2) \alpha + (-10 + 8\, dd1 - 9\, dd2) \alpha^2) + 3$$
$$t22 (4 + (3 + 2\, dd1 - 2\, dd2) \alpha + (1 + dd1 - dd2) \alpha^2) + t12^3$$
$$(-8 - (16 + 5\, t22) \alpha + (7 + 32\, t22 + 36\, t22^2) \alpha^2) + t12^2$$
$$(20 + 2 (11 + 8\, dd1 - 9\, dd2) \alpha + (5 + 8\, dd1 - 9\, dd2) \alpha^2 - 42\, t22^3 \alpha^2 +$$
$$5\, t22^2 \alpha (2 + \alpha) + t22 (-10 + 5 (5 + 2\, dd1) \alpha + (-34 + 5\, dd1) \alpha^2)) + t12$$
$$(24\, t22^4 \alpha^2 - 5\, t22^3 \alpha (1 + 8 \alpha) + t22^2 (44 + (4 - 20\, dd1) \alpha + (23 - 10\, dd1) \alpha^2) +$$
$$3 (-4 + (-3 + 2\, dd1 - 2\, dd2) \alpha + (-1 + dd1 - dd2) \alpha^2) +$$
$$t22 (-16 + (-26 - 44\, dd1 + 48\, dd2) \alpha + (5 - 22\, dd1 + 24\, dd2) \alpha^2)))) -$$
$$m^3 (t12 - t22)^2 (2 s^4 t22 (4\, t12^4 + (-1 + t22)^2 t22 (3 + t22) - 3\, t12^3$$
$$(1 + 5\, t22) + t12^2 t22 (11 + 13\, t22) + t12 (-1 - 3\, t22 + t22^2 - 7\, t22^3)) (1 + \alpha) +$$

$$2\,(2+\alpha+t12^3\,\alpha^2+t22^3\,\alpha^2+t22^2\,\alpha\,(2+\alpha)+t12^2\,\alpha\,(2-t22\,\alpha)-t22$$
$$(3+2\,\alpha+\alpha^2)+t12\,(-1+t22\,(-6+\alpha)\,\alpha-3\,t22^2\,\alpha^2))\,+$$
$$s^2\,(t22^4\,\alpha^2-2\,t22^5\,\alpha^2-8\,t12^4\,(1+t22)\,\alpha^2-(2+\alpha)\,(-2+5\,dd1\,\alpha-6\,dd2\,\alpha)\,+$$
$$t22^3\,(-16+8\,(-1+dd1)\,\alpha+(15+4\,dd1)\,\alpha^2)+t22$$
$$(16+(11+6\,dd1-12\,dd2)\,\alpha+(4+3\,dd1-6\,dd2)\,\alpha^2)+t22^2$$
$$(-22+(-11+12\,dd1-6\,dd2)\,\alpha+(-16+6\,dd1-3\,dd2)\,\alpha^2)+2$$
$$t12^3\,(-5-(7+2\,t22)\,\alpha+t22\,(16+15\,t22)\,\alpha^2)+t12^2$$
$$(14+(13+8\,dd1-6\,dd2)\,\alpha+(11+4\,dd1-3\,dd2)\,\alpha^2-26\,t22^3\,\alpha^2\,-$$
$$3\,t22^2\,\alpha\,(-4+7\,\alpha)+2\,t22\,(7+(19+4\,dd1)\,\alpha+2\,(-6+dd1)\,\alpha^2))+t12$$
$$(-14+(-7+10\,dd1-12\,dd2)\,\alpha+(-5+5\,dd1-6\,dd2)\,\alpha^2+14\,t22^4\,\alpha^2\,-$$
$$2\,t22^3\,\alpha\,(2+11\,\alpha)+t22^2\,(32-4\,(1+6\,dd1)\,\alpha-3\,(-7+4\,dd1)\,\alpha^2)\,-$$
$$2\,t22\,(11+(15+16\,dd1-18\,dd2)\,\alpha+(-1+8\,dd1-9\,dd2)\,\alpha^2)))\,-$$
$$2\,s\,(4\,t12^4\,\alpha^2-t22^4\,\alpha^2-2\,(dd1-dd2)\,t22\,\alpha\,(2+\alpha)-2\,t22^3\,\alpha\,(1+2\,\alpha)\,+$$
$$2\,(2+\alpha)\,(-1+dd1\,\alpha-dd2\,\alpha)-t12^3\,\alpha\,(-2+\alpha+16\,t22\,\alpha)+t22^2$$
$$(10+(4-4\,dd1+2\,dd2)\,\alpha+(3-2\,dd1+dd2)\,\alpha^2)+t12^2\,(-2\,-$$
$$2\,(2+2\,dd1-dd2+5\,t22)\,\alpha+(-5-2\,dd1+dd2+14\,t22+7\,t22^2)\,\alpha^2)+t12$$
$$(-5\,t22^2\,(-2+\alpha)\,\alpha+6\,t22^3\,\alpha^2+2\,t22\,(-4+(2+8\,dd1-6\,dd2)\,\alpha+(-1+4\,dd1\,-$$
$$3\,dd2)\,\alpha^2)+2\,(4+(2-2\,dd1+2\,dd2)\,\alpha+(1-dd1+dd2)\,\alpha^2)))\,-$$
$$s^3\,(t22^4\,\alpha^2+2\,t22^5\,\alpha^2+(dd1-2\,dd2)\,\alpha\,(2+\alpha)+t22^3\,(16+(12-8\,dd1)\,\alpha\,-$$
$$(9+4\,dd1)\,\alpha^2)+t22^2\,(2+(-1-4\,dd1+2\,dd2)\,\alpha+(8-2\,dd1+dd2)\,\alpha^2)\,+$$
$$t22\,(-10+(-7+2\,dd1+4\,dd2)\,\alpha+(-2+dd1+2\,dd2)\,\alpha^2)+8\,t12^4$$
$$(-1-\alpha+t22\,\alpha^2)+t12^3\,(6+40\,t22+6\,\alpha+44\,t22\,\alpha-30\,t22^2\,\alpha^2)+t12^2$$
$$(2+(3+2\,dd2)\,\alpha+(-1+dd2)\,\alpha^2+26\,t22^3\,\alpha^2+t22^2\,(-32-44\,\alpha+7\,\alpha^2)\,-$$
$$2\,t22\,(21+(23+4\,dd1)\,\alpha+(1+2\,dd1)\,\alpha^2))+t12$$
$$(-14\,t22^4\,\alpha^2+2\,t22^3\,\alpha\,(2+5\,\alpha)+t22\,(26+(22-12\,dd2)\,\alpha-6\,dd2\,\alpha^2)+t22^2$$
$$\alpha\,(16-5\,\alpha+12\,dd1\,(2+\alpha))+\alpha\,(-1+\alpha-dd1\,(2+\alpha)+2\,dd2\,(2+\alpha)))))\,+$$
$$m^2\,(2\,s\,t12^6\,(1+s\,t22)\,(-\alpha^2-s\,\alpha^2+s^2\,(1+\alpha))-t12^5\,(1+s\,t22)$$
$$(-\alpha^2+2\,s^3\,(-1+10\,t22)\,(1+\alpha)+s\,\alpha$$
$$(1+(3-20\,t22)\,\alpha)+s^2\,(4+5\,\alpha+(4-20\,t22)\,\alpha^2))\,+$$
$$t12^4\,(1+s\,t22)\,(2\,s^3\,(-6+11\,t22+21\,t22^2)\,(1+\alpha)+\alpha\,(1+\alpha-6\,t22\,\alpha)\,+$$
$$s\,(2+(1+2\,dd1+10\,t22)\,\alpha+(11+dd1-12\,t22-42\,t22^2)\,\alpha^2)+s^2$$
$$(-2+2\,(-2+dd1)\,\alpha+(10+dd1)\,\alpha^2-42\,t22^2\,\alpha^2+t22\,(28+38\,\alpha-6\,\alpha^2)))\,+$$
$$t22^2\,(4+\alpha+2\,s^4\,(-1+t22)^2\,t22^2\,(1+\alpha)+t22^2\,\alpha\,(1+2\,\alpha)-t22\,(6+3\,\alpha+2\,\alpha^2)\,+$$
$$s^2\,(-2\,t22^4\,\alpha^2-3\,(2+\alpha)\,(-1+dd1\,\alpha-dd2\,\alpha)+t22^3\,(-4+2\,(-1+dd1)\,\alpha\,+$$
$$(7+dd1)\,\alpha^2)+t22^2\,(-14+(-7+8\,dd1)\,\alpha+(-6+4\,dd1)\,\alpha^2)\,+$$
$$t22\,(6+(3-6\,dd2)\,\alpha+(1-3\,dd2)\,\alpha^2))+s\,(10+(4-6\,dd1+4\,dd2)\,\alpha\,+$$
$$(-3\,dd1+2\,dd2)\,\alpha^2+t22^3\,\alpha\,(1+2\,\alpha)+t22^2\,(2+\alpha)\,(-5+dd1\,\alpha)\,+$$
$$t22\,(-6+(-3+6\,dd1-4\,dd2)\,\alpha+(-2+3\,dd1-2\,dd2)\,\alpha^2))+s^3$$
$$(-2\,t22^4\,\alpha^2+dd2\,\alpha\,(2+\alpha)+t22^3\,(-2+(-1+2\,dd1)\,\alpha+(5+dd1)\,\alpha^2)\,+$$
$$t22^2\,(-8+(-5+6\,dd1)\,\alpha+(-4+3\,dd1)\,\alpha^2)\,+$$
$$t22\,(8+(5-6\,dd1-2\,dd2)\,\alpha-(-1+3\,dd1+dd2)\,\alpha^2)))-t12^3$$
$$(2-\alpha+10\,t22\,\alpha+\alpha^2-t22\,\alpha^2-6\,t22^2\,\alpha^2+2\,s^4\,t22\,(-5-3\,t22+24\,t22^2+18\,t22^3)$$
$$(1+\alpha)+s^2\,(-6-6\,(2+dd1-dd2)\,\alpha+3\,t22^3\,(6-17\,\alpha)\,\alpha+(4-3\,dd1+3\,dd2)$$
$$\alpha^2-36\,t22^4\,\alpha^2+2\,t22^2\,(19+2\,(19+5\,dd1)\,\alpha+(-14+5\,dd1)\,\alpha^2)\,+$$
$$t22\,(28+(23+14\,dd1-12\,dd2)\,\alpha+(29+7\,dd1-6\,dd2)\,\alpha^2))+s$$
$$(6+(-3-6\,dd1+4\,dd2)\,\alpha+15\,t22^2\,(2-3\,\alpha)\,\alpha+(5-3\,dd1+2\,dd2)\,\alpha^2\,-$$
$$32\,t22^3\,\alpha^2+2\,t22\,(2+2\,(4+5\,dd1-2\,dd2)\,\alpha+(11+5\,dd1-2\,dd2)\,\alpha^2))+s^3$$
$$(-10+2\,(-5+dd2)\,\alpha+dd2\,\alpha^2-36\,t22^4\,\alpha^2+t22^3\,(72+90\,\alpha-19\,\alpha^2)\,+$$
$$t22^2\,(70+(78+20\,dd1)\,\alpha+(11+10\,dd1)\,\alpha^2)\,-$$

$$t22\ (12 + (19 + 6\ dd1 + 4\ dd2)\ \alpha + (-6 + 3\ dd1 + 2\ dd2)\ \alpha^{2}))) +$$
$$t12^{2}\ (4 - 2\ t22 + \alpha - 5\ t22\ \alpha + 18\ t22^{2}\ \alpha - 3\ t22^{2}\ \alpha^{2} + 2\ t22^{3}\ \alpha^{2} + 2\ s^{4}$$
$$t22\ (-1 - 5\ t22 + 5\ t22^{2} + 14\ t22^{3} + 8\ t22^{4})\ (1 + \alpha) + s^{3}$$
$$(-2 + 2\ (-1 + dd2)\ \alpha + dd2\ \alpha^{2} - 16\ t22^{5}\ \alpha^{2} + t22^{4}\ (28 + 38\ \alpha - 9\ \alpha^{2}) +$$
$$2\ t22^{3}\ (25 + 18\ (2 + dd1)\ \alpha + (2 + 9\ dd1)\ \alpha^{2}) +$$
$$t22^{2}\ (16 + (7 - 4\ dd1 - 10\ dd2)\ \alpha + (3 - 2\ dd1 - 5\ dd2)\ \alpha^{2}) -$$
$$2\ t22\ (5 + (7 + 4\ dd1 - 2\ dd2)\ \alpha + (-1 + 2\ dd1 - dd2)\ \alpha^{2})) + s$$
$$(6 + 4\ dd2\ \alpha + t22^{3}\ (30 - 29\ \alpha)\ \alpha + 2\ dd2\ \alpha^{2} - 3\ dd1\ \alpha\ (2 + \alpha) +$$
$$t22^{2}\ (-16 + 20\ (1 + 2\ dd1 - dd2)\ \alpha + (1 + 20\ dd1 - 10\ dd2)\ \alpha^{2}) +$$
$$t22\ (18 + (1 - 10\ dd1 + 8\ dd2)\ \alpha + (10 - 5\ dd1 + 4\ dd2)\ \alpha^{2})) + s^{2}\ (t22^{4}$$
$$(10 - 9\ \alpha)\ \alpha - 16\ t22^{5}\ \alpha^{2} + t22^{3}\ (-2 + (50 + 36\ dd1)\ \alpha + 9\ (-3 + 2\ dd1)\ \alpha^{2}) +$$
$$t22^{2}\ (36 + (45 + 36\ dd1 - 30\ dd2)\ \alpha + (7 + 18\ dd1 - 15\ dd2)\ \alpha^{2}) -$$
$$3\ t22\ (-4 + (2 + 6\ dd1 - 4\ dd2)\ \alpha + (3\ dd1 - 2\ (2 + dd2))\ \alpha^{2}) -$$
$$3\ \alpha\ (1 + dd1\ (2 + \alpha) - dd2\ (2 + \alpha)))) -$$
$$t12\ t22\ (8 - 10\ t22 + 2\ \alpha - 7\ t22\ \alpha + 10\ t22^{2}\ \alpha - 3\ t22\ \alpha^{2} + t22^{2}\ \alpha^{2} + 3\ t22^{3}$$
$$\alpha^{2} + 2\ s^{4}\ t22\ (-1 + t22 + 3\ t22^{3} + 2\ t22^{4})\ (1 + \alpha) + s^{2}\ (6 + 18\ dd2\ \alpha + 9\ dd2\ \alpha^{2} -$$
$$4\ t22^{5}\ \alpha^{2} + t22^{4}\ \alpha\ (1 + \alpha) - 9\ dd1\ \alpha\ (2 + \alpha) + t22^{3}\ (-18 + (6 + 20\ dd1)\ \alpha +$$
$$(9 + 10\ dd1)\ \alpha^{2}) + t22\ (24 + (9 - 12\ dd2)\ \alpha + (9 - 6\ dd2)\ \alpha^{2}) +$$
$$t22^{2}\ (-8 + (11 + 26\ dd1 - 12\ dd2)\ \alpha + (13\ dd1 - 6\ (3 + dd2))\ \alpha^{2})) +$$
$$s^{3}\ (-2 - 2\ (1 + dd1 - 3\ dd2)\ \alpha - (dd1 - 3\ dd2)\ \alpha^{2} - 4\ t22^{5}\ \alpha^{2} + t22^{4}$$
$$(4 + 5\ \alpha - 2\ \alpha^{2}) + t22\ (8 + (1 - 10\ dd1 - 4\ dd2)\ \alpha + (3 - 5\ dd1 - 2\ dd2)\ \alpha^{2}) +$$
$$t22^{2}\ (8 + (9 + 6\ dd1 - 4\ dd2)\ \alpha + (-7 + 3\ dd1 - 2\ dd2)\ \alpha^{2}) +$$
$$t22^{3}\ \alpha\ (13 + 8\ \alpha + 10\ dd1\ (2 + \alpha))) + s\ (3\ t22^{4}\ \alpha^{2} + t22^{3}\ \alpha\ (11 + 4\ \alpha) +$$
$$t22\ (6 + (1 + 10\ dd1 - 8\ dd2)\ \alpha + (3 + 5\ dd1 - 4\ dd2)\ \alpha^{2}) +$$
$$2\ (8 + (2 - 8\ dd1 + 6\ dd2)\ \alpha + (-4\ dd1 + 3\ dd2)\ \alpha^{2}) +$$
$$2\ t22^{2}\ (-14 - 5\ \alpha^{2} + 5\ dd1\ \alpha\ (2 + \alpha) - 2\ dd2\ \alpha\ (2 + \alpha))))) +$$
$$m\ (2\ t12\ t22 - 2\ t22^{2} - 2\ t12\ t22^{2} + 2\ t22^{3} + t12^{2}\ \alpha - t12^{3}\ \alpha - 2\ t12\ t22\ \alpha +$$
$$3\ t12^{2}\ t22\ \alpha + 2\ t12^{3}\ t22\ \alpha - t22^{2}\ \alpha + t12\ t22^{2}\ \alpha -$$
$$6\ t12^{2}\ t22^{2}\ \alpha + t22^{3}\ \alpha + 2\ t12\ t22^{3}\ \alpha + t12^{3}\ \alpha^{2} - t12^{4}\ \alpha^{2} -$$
$$2\ t12^{2}\ t22\ \alpha^{2} + t12^{3}\ t22\ \alpha^{2} + 2\ t12^{4}\ t22\ \alpha^{2} - t12\ t22^{2}\ \alpha^{2} +$$
$$3\ t12^{2}\ t22^{2}\ \alpha^{2} - 4\ t12^{3}\ t22^{2}\ \alpha^{2} + t12\ t22^{3}\ \alpha^{2} + 2\ s^{4}\ t12\ t22$$
$$(t12^{4}\ (-1 + 2\ t22) - t12^{3}\ (-3 + t22 + 6\ t22^{2}) + t22\ (-2 + t22 + t22^{2} + t22^{3}) + 3\ t12^{2}$$
$$(-1 - 2\ t22 + 4\ t22^{2} + t22^{3}) - t12\ (-1 - 7\ t22 + 7\ t22^{2} + 4\ t22^{3} + t22^{4}))\ (1 + \alpha) +$$
$$s^{3}\ (-(-1 + t22)\ t22^{3}\ (2 + \alpha)\ (-1 + dd1\ \alpha) - 2\ t12^{5}\ (-1 + 2\ t22)\ (-1 - \alpha + t22\ \alpha^{2}) +$$
$$t12^{4}\ (12\ t22^{3}\ \alpha^{2} + 6\ (1 + \alpha) + t22\ (2 + 3\ \alpha - 4\ \alpha^{2}) - 2\ t22^{2}\ (10 + 11\ \alpha + \alpha^{2})) + t12^{3}$$
$$(-6\ t22^{4}\ \alpha^{2} - 6\ (1 + \alpha) + t22^{3}\ (24 + 30\ \alpha - 14\ \alpha^{2}) - t22\ (20 + 2\ (11 + dd1)\ \alpha +$$
$$(-2 + dd1)\ \alpha^{2}) + t22^{2}\ (26 + (25 + 4\ dd1)\ \alpha + 2\ (6 + dd1)\ \alpha^{2})) + t12^{2}$$
$$(2\ t22^{5}\ \alpha^{2} + 2\ (1 + \alpha) + t22^{4}\ (-8 - 10\ \alpha + 3\ \alpha^{2}) - t22^{3}\ (28 + (37 + 12\ dd1)\ \alpha +$$
$$(-4 + 6\ dd1)\ \alpha^{2}) + t22\ (18 + (19 + 2\ dd1 - 2\ dd2)\ \alpha + (dd1 - dd2)\ \alpha^{2}) +$$
$$t22^{2}\ (-4 + (3 + 6\ dd1 + 2\ dd2)\ \alpha + (-7 + 3\ dd1 + dd2)\ \alpha^{2})) + t12$$
$$t22\ (-4 + 2\ (-2 + dd2)\ \alpha + dd2\ \alpha^{2} - 2\ t22^{4}\ \alpha^{2} + t22^{3}\ (2 + \alpha)$$
$$(3 + (3 + 2\ dd1)\ \alpha) + t22^{2}\ (6 + (8 + 6\ dd1)\ \alpha + (-2 + 3\ dd1)\ \alpha^{2}) -$$
$$t22\ (2 + 2\ (3 + 4\ dd1 + dd2)\ \alpha + (-1 + 4\ dd1 + dd2)\ \alpha^{2}))) +$$
$$s\ (2\ t12^{5}\ (1 - 2\ t22)\ \alpha^{2} + (-1 + t22)\ t22^{2}\ (2 + \alpha)\ (2 + t22 - dd1\ \alpha) + t12^{4}\ \alpha$$
$$(1 - 5\ \alpha + 14\ t22^{2}\ \alpha - t22\ (2 + \alpha)) + t12^{3}\ (-2 - (5 + 2\ dd1)\ \alpha + t22^{2}\ (8 - 17\ \alpha)\ \alpha -$$
$$(-3 + dd1)\ \alpha^{2} - 10\ t22^{3}\ \alpha^{2} + 2\ t22\ (2 + 2\ (1 + dd1)\ \alpha + (7 + dd1)\ \alpha^{2})) + t12$$
$$t22\ (2 + (-6 - 8\ dd1 + 4\ dd2)\ \alpha + (-4\ dd1 + 2\ dd2)\ \alpha^{2} + t22^{3}\ \alpha\ (2 + \alpha) +$$
$$2\ t22^{2}\ (-2 + 2\ (1 + dd1)\ \alpha + (-1 + dd1)\ \alpha^{2}) + t22\ (4 + (5 + 6\ dd1 - 4\ dd2)\ \alpha +$$
$$(1 + 3\ dd1 - 2\ dd2)\ \alpha^{2})) + t12^{2}\ (2 + 2\ (2 + dd1)\ \alpha + dd1\ \alpha^{2} + t22^{3}\ \alpha$$
$$(-8 + 11\ \alpha) + t22\ (-4 + (7 + 6\ dd1 - 4\ dd2)\ \alpha + (-8 + 3\ dd1 - 2\ dd2)\ \alpha^{2}) +$$

$$t22^2\ (-2 - 2\ (7 + 6\ dd1 - 2\ dd2)\ \alpha + (1 - 6\ dd1 + 2\ dd2)\ \alpha^2)))\ -$$
$$s^2\ (2\ t12^5\ (-1 + t22 + 2\ t22^2)\ \alpha^2 + (-1 + t22)\ t22^2\ (2 + \alpha)\ (-1 + dd1\ \alpha +$$
$$t22\ (-2 + dd1\ \alpha)) + t12^4\ (-4 - 5\ \alpha + 2\ t22^2\ (1 - 6\ \alpha)\ \alpha + 4\ \alpha^2 - 12\ t22^3\ \alpha^2 +$$
$$t22\ (8 + 9\ \alpha + 7\ \alpha^2)) + t12^2\ (-4 - (5 + 2\ dd1)\ \alpha + t22^4\ (2 - 3\ \alpha)\ \alpha -$$
$$dd1\ \alpha^2 - 2\ t22^5\ \alpha^2 + t22^3\ (8 + (25 + 12\ dd1)\ \alpha + 3\ (-5 + 2\ dd1)\ \alpha^2) +$$
$$t22^2\ (24 + (23 + 6\ dd1 - 6\ dd2)\ \alpha + 3\ (3 + dd1 - dd2)\ \alpha^2) +$$
$$t22\ (-12 + (-21 - 8\ dd1 + 6\ dd2)\ \alpha + (6 - 4\ dd1 + 3\ dd2)\ \alpha^2)) + t12^3$$
$$(8 + 2\ (5 + dd1)\ \alpha + (-2 + dd1)\ \alpha^2 + 6\ t22^4\ \alpha^2 + 6\ t22^3\ \alpha\ (-1 + 4\ \alpha) + t22^2$$
$$(-22 - (29 + 4\ dd1)\ \alpha + (1 - 2\ dd1)\ \alpha^2) - t22\ \alpha\ (-4 + 15\ \alpha + dd1\ (2 + \alpha))) +$$
$$t12\ t22\ (4 + 8\ \alpha + 8\ dd1\ \alpha - 6\ dd2\ \alpha + 4\ dd1\ \alpha^2 - 3\ dd2\ \alpha^2 + 2\ t22^4\ \alpha^2 -$$
$$t22^2\ (6 + 2\ (7 + 5\ dd1)\ \alpha + 5\ (-1 + dd1)\ \alpha^2) - t22^3\ (-2 + (3 + 4\ dd1)\ \alpha +$$
$$2\ (2 + dd1)\ \alpha^2) + t22\ (dd1\ \alpha\ (2 + \alpha) + 3\ dd2\ \alpha\ (2 + \alpha) - 3\ (2 + \alpha^2)))))) \Big/$$
$$((1 + s)\ (m^2\ s\ (2 + s)\ (t12 - t22) + (-1 + s\ (-1 + t12))\ (1 + s\ t22)\ -$$
$$2\ m\ (-1 + s\ (-1 + t12))\ (1 + s\ t22))$$
$$(-2\ m^3\ (t12 - t22)^4 + m^4\ (t12 - t22)^4 + (-1 + t12)\ t12\ (-1 + t22)\ t22 +$$
$$m^2\ (t12 - t22)^2\ (-1 + t12 + t12^2 + t22 - 4\ t12\ t22 + t22^2) +$$
$$m\ (t12 - t22)^2\ (1 - t22 + t12\ (-1 + 2\ t22)))\ \alpha\ (2 + \alpha))),$$
$$p22 \to ((-1 + t22)\ t22\ (2\ s^3\ t22^2\ (1 + \alpha) - \alpha\ (1 + t22\ \alpha) +$$
$$s\ (2 + (1 + 2\ dd2 - t22)\ \alpha + (dd2 - 2\ t22^2)\ \alpha^2) +$$
$$s^2\ (-2\ t22^2\ \alpha^2 + dd2\ \alpha\ (2 + \alpha) + t22\ (4 + 3\ \alpha + \alpha^2))) +$$
$$m^3\ (t12 - t22)^2\ ((t12 - t22)\ \alpha^2 + 2\ s^3\ (t12 - t22)\ (-1 + t22)\ (1 + \alpha) +$$
$$s\ \alpha\ (dd1\ (2 + \alpha) - dd2\ (2 + \alpha) - (t12 - t22)\ (1 + 2\ (-1 + t22)\ \alpha)) +$$
$$s^2\ (2\ t22^2\ \alpha^2 + (dd1 - dd2)\ \alpha\ (2 + \alpha) +$$
$$t22\ (2 + 3\ \alpha - \alpha^2) + t12\ (-2 - 3\ \alpha + (1 - 2\ t22)\ \alpha^2))) +$$
$$m^2\ (t12 - t22)\ (6\ s^3\ (t12 - t22)\ (-1 + t22)\ t22\ (1 + \alpha) +$$
$$(t12 - t22)\ \alpha\ (-1 + (-1 + t22)\ \alpha) +$$
$$s^2\ (t22^2\ (3 - 5\ \alpha)\ \alpha + 6\ t22^3\ \alpha^2 - (dd1 - dd2)\ \alpha\ (2 + \alpha) +$$
$$t22\ (4 + (3 + 4\ dd1 - 6\ dd2)\ \alpha + (1 + 2\ dd1 - 3\ dd2)\ \alpha^2) +$$
$$t12\ (-4 + (-3 + 2\ dd2 - 3\ t22)\ \alpha + (-1 + dd2 + 5\ t22 - 6\ t22^2)\ \alpha^2)) +$$
$$s\ (3\ t22^2\ (1 - 2\ \alpha)\ \alpha + 6\ t22^3\ \alpha^2 - (dd1 - dd2)\ \alpha\ (2 + \alpha) +$$
$$t22\ (4 + (4 + 4\ dd1 - 6\ dd2)\ \alpha + (2 + 2\ dd1 - 3\ dd2)\ \alpha^2) +$$
$$t12\ (-4 + (-4 + 2\ dd2 - 3\ t22)\ \alpha + (-2 + dd2 + 6\ t22 - 6\ t22^2)\ \alpha^2))) +$$
$$(m\ (1 + s\ t12)\ (-(-1 + m)\ (-1 + m\ (t12 - t22) + t22)\ (m\ (t12 - t22) + t22)$$
$$(1 + s\ t22)\ (2\ ((-1 + m)\ t12 - m\ t22) + 4\ s\ ((-1 + m)\ t12 - m\ t22)$$
$$(1 + (-1 + m)\ t12 - m\ t22) + 2\ t12\ ((-1 + m)\ t12 - m\ t22)\ \alpha - 2\ m\ t12$$
$$((-1 + m)\ t12 - m\ t22)\ \alpha + ((-1 + m)\ t12 - m\ t22)\ (1 + (-1 + m)\ t12 - m\ t22)$$
$$\alpha + 2\ s\ ((-1 + m)\ t12 - m\ t22)\ (1 + (-1 + m)\ t12 - m\ t22)\ \alpha + 2$$
$$dd1\ (-1 + m)\ s\ ((-1 + m)\ t12 - m\ t22)\ (1 + (-1 + m)\ t12 - m\ t22)$$
$$\alpha - 2\ dd2\ m\ s\ ((-1 + m)\ t12 - m\ t22)\ (1 + (-1 + m)\ t12 - m\ t22)$$
$$\alpha + s\ t12\ ((-1 + m)\ t12 - m\ t22)\ (1 + (-1 + m)\ t12 - m\ t22)\ \alpha - m\ s$$
$$t12\ ((-1 + m)\ t12 - m\ t22)\ (1 + (-1 + m)\ t12 - m\ t22)\ \alpha - 2\ m\ t22$$
$$(t12 - m\ t12 + m\ t22)\ \alpha + m\ s\ t22\ (-1 + t12 - m\ t12 + m\ t22)\ (t12 - m\ t12 + m\ t22)$$
$$\alpha + dd1\ (-1 + m)\ s\ ((-1 + m)\ t12 - m\ t22)\ (1 + (-1 + m)\ t12 - m\ t22)$$
$$\alpha^2 - dd2\ m\ s\ ((-1 + m)\ t12 - m\ t22)\ (1 + (-1 + m)\ t12 - m\ t22)$$
$$\alpha^2 + t12\ ((-1 + m)\ t12 - m\ t22)\ (1 + (-1 + m)\ t12 - m\ t22)\ \alpha^2 - m$$
$$t12\ ((-1 + m)\ t12 - m\ t22)\ (1 + (-1 + m)\ t12 - m\ t22)\ \alpha^2 + s$$
$$t12\ ((-1 + m)\ t12 - m\ t22)\ (1 + (-1 + m)\ t12 - m\ t22)\ \alpha^2 - m$$
$$s\ t12\ ((-1 + m)\ t12 - m\ t22)\ (1 + (-1 + m)\ t12 - m\ t22)\ \alpha^2 + m$$

$$
\begin{aligned}
&t22\,(-1+t12-m\,t12+m\,t22)\,(t12-m\,t12+m\,t22)\,\alpha^2+m\,s\,t22\\
&(-1+t12-m\,t12+m\,t22)\,(t12-m\,t12+m\,t22)\,\alpha^2+s^2\,((-1+m)\,t12-m\,t22)\\
&(1+(-1+m)\,t12-m\,t22)\,(2+\alpha)+(-1+m)\,s^2\,t12\,((-1+m)\,t12-m\,t22)\\
&(1+(-1+m)\,t12-m\,t22)\,(2+\alpha)-m\,s^2\,t22\,(-1+t12-m\,t12+m\,t22)\\
&(t12-m\,t12+m\,t22)\,(2+\alpha)-dd1\,s^2\,((-1+m)\,t12-m\,t22)\\
&(1+(-1+m)\,t12-m\,t22)\,\alpha\,(2+\alpha)+dd1\,m\,s^2\,((-1+m)\,t12-m\,t22)\\
&(1+(-1+m)\,t12-m\,t22)\,\alpha\,(2+\alpha)-dd2\,m\,s^2\,((-1+m)\,t12-m\,t22)\\
&(1+(-1+m)\,t12-m\,t22)\,\alpha\,(2+\alpha)+2\,t12\,(1+s\,(1+(-1+m)\,t12-m\,t22))\\
&(1+(1+(-1+m)\,t12-m\,t22)\,\alpha+s\,(1+(-1+m)\,t12-m\,t22)\,(1+\alpha))\\
&(1+s\,(1+(-1+m)\,t12-m\,t22)+m\,t22\,\alpha+t12\,(\alpha-m\,\alpha)))+\\
&m\,(-1+s\,(-1+t22))\,((-1+m)\,t12-m\,t22)\,(-1+t12-m\,t12+m\,t22)\\
&(2\,(1+s)\,(m\,(t12-t22)+t22)+4\,m\,s\,(1+s)\,t12\,(m\,(t12-t22)+t22)+4\,s\\
&(1+s)\,t22\,(m\,(t12-t22)+t22)-4\,m\,s\,(1+s)\,t22\,(m\,(t12-t22)+t22)+\\
&(1+s)\,(m\,(t12-t22)+t22)\,\alpha+3\,m\,s\,(1+s)\,t12\,(m\,(t12-t22)+t22)\\
&\alpha-2\,dd2\,m\,s\,(1+s)\,t12\,(m\,(t12-t22)+t22)\,\alpha+m^2\,s\,(1+s)\,t12\,(t12-t22)\\
&(m\,(t12-t22)+t22)\,\alpha+(1+s)\,t22\,(m\,(t12-t22)+t22)\,\alpha+3\,s\,(1+s)\\
&t22\,(m\,(t12-t22)+t22)\,\alpha-2\,dd2\,s\,(1+s)\,t22\,(m\,(t12-t22)+t22)\\
&\alpha-3\,m\,s\,(1+s)\,t22\,(m\,(t12-t22)+t22)\,\alpha-2\,dd1\,m\,s\,(1+s)\,t22\\
&(m\,(t12-t22)+t22)\,\alpha+4\,dd2\,m\,s\,(1+s)\,t22\,(m\,(t12-t22)+t22)\,\alpha+2\\
&m\,s\,(1+s)\,t12\,t22\,(m\,(t12-t22)+t22)\,\alpha-m^2\,s\,(1+s)\,(t12-t22)\,t22\\
&(m\,(t12-t22)+t22)\,\alpha+s\,(1+s)\,t22^2\,(m\,(t12-t22)+t22)\,\alpha-2\,m\,s\\
&(1+s)\,t22^2\,(m\,(t12-t22)+t22)\,\alpha+m\,s\,(1+s)\,t12\,(m\,(t12-t22)+t22)\\
&\alpha^2-dd2\,m\,s\,(1+s)\,t12\,(m\,(t12-t22)+t22)\,\alpha^2-m^2\,s\,(1+s)\,t12\\
&(t12-t22)\,(m\,(t12-t22)+t22)\,\alpha^2-m^2\,(1+s)\,(t12-t22)^2\\
&(m\,(t12-t22)+t22)\,\alpha^2+(1+s)\,t22\,(m\,(t12-t22)+t22)\,\alpha^2+s\,(1+s)\\
&t22\,(m\,(t12-t22)+t22)\,\alpha^2-dd2\,s\,(1+s)\,t22\,(m\,(t12-t22)+t22)\\
&\alpha^2-m\,s\,(1+s)\,t22\,(m\,(t12-t22)+t22)\,\alpha^2-dd1\,m\,s\,(1+s)\,t22\\
&(m\,(t12-t22)+t22)\,\alpha^2+2\,dd2\,m\,s\,(1+s)\,t22\,(m\,(t12-t22)+t22)\,\alpha^2-2\\
&m\,s\,(1+s)\,t12\,t22\,(m\,(t12-t22)+t22)\,\alpha^2+m^2\,s\,(1+s)\,(t12-t22)\,t22\\
&(m\,(t12-t22)+t22)\,\alpha^2-(1+s)\,t22^2\,(m\,(t12-t22)+t22)\,\alpha^2-s\,(1+s)\\
&t22^2\,(m\,(t12-t22)+t22)\,\alpha^2+2\,m\,s\,(1+s)\,t22^2\,(m\,(t12-t22)+t22)\\
&\alpha^2+2\,m^2\,s^2\,(1+s)\,(t12-t22)^2\,(m\,(t12-t22)+t22)\,(1+\alpha)+4\,m\,s^2\\
&(1+s)\,(t12-t22)\,t22\,(m\,(t12-t22)+t22)\,(1+\alpha)+2\,s^2\,(1+s)\,t22^2\\
&(m\,(t12-t22)+t22)\,(1+\alpha)+dd2\,s\,(1+s)\,(m\,(t12-t22)+t22)\,\alpha\\
&(2+\alpha)+(dd1-dd2)\,m\,s\,(1+s)\,(m\,(t12-t22)+t22)\,\alpha\,(2+\alpha)-dd1\\
&m^2\,s\,(1+s)\,(t12-t22)\,(m\,(t12-t22)+t22)\,\alpha\,(2+\alpha)+dd2\,m^2\\
&s\,(1+s)\,(t12-t22)\,(m\,(t12-t22)+t22)\,\alpha\,(2+\alpha)+m\,(1+s)\\
&(t12-t22)\,(m\,(t12-t22)+t22)\,\alpha\,(1+\alpha-2\,t22\,\alpha)-2\,t22\\
&(1+m\,s\,(t12-t22)+s\,t22)\,(1+s\,t22+m\,(t12-t22)\,(s-\alpha)+\alpha-t22\,\alpha)\\
&(1+t22\,\alpha+s\,t22\,(1+\alpha)+m\,(t12-t22)\,(s+\alpha+s\,\alpha)))))\,/\\
&(((-1+m)^2\,t12^2+m\,t22\,(-1+m\,t22)-(-1+m)\,t12\,(-1+2\,m\,t22))\\
&(m^2\,s\,(2+s)\,(t12-t22)+(-1+s\,(-1+t12))\,(1+s\,t22)-\\
&2\,m\,(-1+s\,(-1+t12))\,(1+s\,t22)))+\\
&m\,(t22\,(2+\alpha+2\,t22\,\alpha+t22^2\,\alpha^2-6\,s^3\,(-1+t22)\,t22^2\,(1+\alpha)+\\
&s\,(3\,t22^2\,(1-2\,\alpha)\,\alpha+6\,t22^3\,\alpha^2-(2+\alpha)\,(-1+dd1\,\alpha-2\,dd2\,\alpha)+\\
&t22\,(2+2\,(1+dd1-3\,dd2)\,\alpha+(2+dd1-3\,dd2)\,\alpha^2))+\\
&s^2\,(6\,t22^3\,\alpha^2-(dd1-2\,dd2)\,\alpha\,(2+\alpha)-t22^2\,(6+3\,\alpha+7\,\alpha^2)+\\
&t22\,(8+2\,(3+dd1-3\,dd2)\,\alpha+(2+dd1-3\,dd2)\,\alpha^2)))+\\
&t12\,(-2-\alpha-2\,t22\,\alpha-t22^2\,\alpha^2+6\,s^3\,(-1+t22)\,t22^2\,(1+\alpha)-
\end{aligned}
$$

```
s^2 (6 t22^3 α^2 + dd2 α (2 + α) - t22^2 (6 + 3 α + 7 α^2) +
      t22 (8 + (6 - 4 dd2) α - 2 (-1 + dd2) α^2)) - s (3 t22^2 (1 - 2 α) α +
      6 t22^3 α^2 + (2 + α) (1 + dd2 α) + 2 t22 (1 + α - 2 dd2 α + α^2 - dd2 α^2))))) /
  ((-1 + m) (1 + s) (1 + s t22) (m^2 (t12 - t22)^2 + (-1 + t22) t22 +
      m (t12 - t22) (-1 + 2 t22)) α (2 +
      α))};
```

```
del1 = Factor[deltagensym[[1]] /. p12p22gentdd];
```

```
Exponent[Numerator[del1], {t12, t22, dd1, dd2}]
```

```
{9, 9, 1, 1}
```

```
del4 = Factor[deltagensym[[4]] /. p12p22gentdd];
```

```
Exponent[Numerator[del4], {t12, t22, dd1, dd2}]
```

```
{9, 9, 1, 1}
```

Therefore, we can indeed solve {deltagensym[[1]], deltagensym[[4]]} = 0 for (dd1, dd2):

```
Simplify[{dd1, dd2} /. Solve[{del1, del4} == 0, {dd1, dd2}]]
```

The following is (dd1, dd2) as a function of (t12, t22); this is independent of r:

```
dd12gent12 =
  {dd1 → - ((((-1 + t12) t12 (m^6 s (t12 - t22)^6 (-2 α^2 + s (-3 - 2 t12 + 2 t22) α^2 + 2 s^3 (1 + t12 -
                t22) (1 + α) + s^2 (4 + 4 α + (-1 - 2 t12 + 2 t22) α^2)) - m^5 (t12 - t22)^5
            (2 α^2 + 2 s (2 - t12 + t22) α^2 + 2 s^4 (-2 + t12 + 3 t12^2 - t22 - 6 t12 t22 + 3 t22^2)
              (1 + α) - 3 s^2 (4 + 4 α + (-1 + t12 + 2 t12^2 - t22 - 4 t12 t22 + 2 t22^2) α^2) +
             s^3 (-12 - 12 α + α^2 - 6 t12^2 α^2 - 6 t22^2 α^2 + t22 (-4 - 4 α + α^2) +
                t12 (4 + 4 α + (-1 + 12 t22) α^2))) +
           m^4 (t12 - t22)^4 (3 (t12 - t22) α^2 + 2 s^4 (1 + 3 t12^3 + 5 t22 - 12 t12^2 t22 -
                3 t22^2 - 3 t22^3 + 2 t12 (-2 + t22 + 6 t22^2)) (1 + α) +
             s (12 + 12 α + (2 + 4 t12^2 + t12 (3 - 2 t22) - 9 t22 + 4 t22^2) α^2) -
             3 s^2 (-6 - 6 α - α^2 + 2 t12^3 α^2 - 3 t22^2 α^2 - 2 t22^3 α^2 - t12^2 (1 + 8 t22) α^2 +
                t22 (-6 - 6 α + 4 α^2) + t12 (6 + 6 α + (-1 + t22 + 8 t22^2) α^2)) +
             s^3 (10 + 10 α + α^2 - 6 t12^3 α^2 + 6 t22^3 α^2 + t22 (20 + 20 α - 6 α^2) +
                t22^2 (-6 - 6 α + 5 α^2) + t12^2 (-6 - 6 α + (-1 + 24 t22) α^2) -
                t12 (16 + 16 α - 3 α^2 + 24 t22^2 α^2 + t22 (-8 - 8 α + α^2)))) -
           (-1 + t12) t12 (-1 + t22) t22 ((t12 - t22) α^2 + 2 s^4 (-1 + t12) (-1 + t12 - t22)
              t22 (1 + α) + s (4 + 4 α - (-3 t12 + 2 t12^2 + t22 - 2 t12 t22 + 2 t22^2) α^2) +
             s^2 (-4 t22^2 α^2 - 2 t12^2 (1 + t22) α^2 + 6 (1 + α) + t22 (6 + 6 α + α^2) +
                t12 (-6 - 6 α + (2 + 3 t22 + 2 t22^2) α^2)) +
             s^3 (2 (1 + α) + t22^2 (2 + 2 α - 2 α^2) + t22 (8 + 8 α + α^2) + t12^2
                  (2 + 2 α - 2 t22 α^2) + t12 (2 t22^2 α^2 - 4 (1 + α) + t22 (-8 - 8 α + α^2)))) -
           m^3 (t12 - t22)^3 (2 s^4 (t12^4 + t12^3 (1 - 10 t22) + t12^2 (-5 + 5 t22 + 18 t22^2) +
                t12 (3 + 8 t22 - 11 t22^2 - 10 t22^3) + t22 (-3 - 3 t22 + 5 t22^2 + t22^3))
              (1 + α) - 2 (2 + 2 α + (1 - t22 + t12 (-1 + 2 t22)) α^2) +
             2 s (3 t12^3 α^2 + t22^2 α^2 - 3 t22^3 α^2 - t12^2 (1 + 7 t22) α^2 + 2 t22 (-4 - 4 α + α^2) -
                2 (2 + 2 α + α^2) + t12 (8 + 8 α + (2 - 4 t22 + 7 t22^2) α^2)) -
             s^2 (2 t12^4 α^2 + 15 t22^3 α^2 + 2 t22^4 α^2 - t12^3 (3 + 20 t22) α^2 +
                t22^2 (6 + 6 α - 9 α^2) - 3 t22 (-8 - 8 α + α^2) +
                3 (2 + 2 α + α^2) + 3 t12^2 (2 + 2 α + (-1 + 5 t22 + 12 t22^2) α^2) -
```

$$t12\ (27\ t22^2\ \alpha^2 + 20\ t22^3\ \alpha^2 + 3\ (8 + 8\ \alpha + \alpha^2) - 6\ t22\ (-2 - 2\ \alpha + 3\ \alpha^2))) -$$
$$s^3\ (2 + 2\ \alpha + \alpha^2 + 2\ t12^4\ \alpha^2 + 2\ t22^4\ \alpha^2 + t22^2\ (2 + 2\ \alpha - 7\ \alpha^2) + t22\ (20 + 20\ \alpha -$$
$$\alpha^2) + t22^3\ (-8 - 8\ \alpha + 9\ \alpha^2) + t12^3\ (8 + 8\ \alpha + (3 - 20\ t22)\ \alpha^2) + t12^2$$
$$(36\ t22^2\ \alpha^2 + t22\ (-32 - 32\ \alpha + \alpha^2) - 5\ (-2 - 2\ \alpha + \alpha^2)) - t12\ (20 + 20\ \alpha +$$
$$\alpha^2 + 20\ t22^3\ \alpha^2 - 2\ t22\ (-6 - 6\ \alpha + 7\ \alpha^2) + t22^2\ (-32 - 32\ \alpha + 13\ \alpha^2)))) -$$
$$m\ (t12 - t22)\ (2\ s\ t12^4\ (-1 - 2\ (-1 + s)\ t22 + 3\ s\ t22^2)\ (-\alpha^2 - s\ \alpha^2 + s^2\ (1 + \alpha)) +$$
$$(1 + s)\ (-1 + t22)\ t22\ (-2 - 2\ \alpha - t22\ \alpha^2 + 2\ s^3\ t22^2\ (1 + \alpha) +$$
$$s^2\ t22\ (2 + 2\ \alpha + (1 - 2\ t22)\ \alpha^2) - 2\ s\ t22\ (1 + \alpha + t22\ \alpha^2)) +$$
$$t12^3\ ((-1 + 2\ t22)\ \alpha^2 - 6\ s^4\ t22\ (-2 + t22 + 2\ t22^2)\ (1 + \alpha) +$$
$$s\ (2 + 2\ \alpha + (-5 + 2\ t22 + 10\ t22^2)\ \alpha^2) +$$
$$s^3\ (-3 + 4\ t22)\ (3\ t22^2\ \alpha^2 - 2\ (1 + \alpha) + 2\ t22\ (-3 - 3\ \alpha + \alpha^2)) +$$
$$s^2\ (6 + 6\ \alpha - 4\ \alpha^2 + 9\ t22^2\ \alpha^2 + 12\ t22^3\ \alpha^2 - 6\ t22\ (1 + \alpha + \alpha^2))) +$$
$$t12\ (2 + 2\ \alpha + t22^2\ (-1 + 2\ t22)\ \alpha^2 - 2\ s^4\ t22\ (-2 - 3\ t22 + 2\ t22^2 + 4\ t22^3)$$
$$(1 + \alpha) + s\ (6\ t22^3\ \alpha^2 + 4\ t22^4\ \alpha^2 + 6\ (1 + \alpha) - t22^2\ (2 + 2\ \alpha + 7\ \alpha^2)) +$$
$$s^3\ (2\ (1 + \alpha) + t22^2\ (2 + 2\ \alpha - 5\ \alpha^2) + 2\ t22\ (9 + 9\ \alpha + \alpha^2) - 2\ t22^3$$
$$(11 + 11\ \alpha + 2\ \alpha^2) + t22^4\ (-4 - 4\ \alpha + 8\ \alpha^2)) + s^2\ (12\ t22^4\ \alpha^2 + 6\ (1 + \alpha) -$$
$$6\ t22^3\ (1 + \alpha) + 2\ t22\ (9 + 9\ \alpha + \alpha^2) - t22^2\ (18 + 18\ \alpha + 11\ \alpha^2))) +$$
$$t12^2\ (-2 - 2\ \alpha - (-1 + t22 + 2\ t22^2)\ \alpha^2 + 6\ s^4\ t22\ (-2 - t22 + 3\ t22^2 + t22^3)$$
$$(1 + \alpha) + s\ (-8 - 8\ \alpha + 3\ \alpha^2 - 4\ t22^2\ \alpha^2 - 10\ t22^3\ \alpha^2 + t22\ (2 + 2\ \alpha + 3\ \alpha^2)) +$$
$$s^2\ (-21\ t22^3\ \alpha^2 - 6\ t22^4\ \alpha^2 + 2\ (-6 - 6\ \alpha + \alpha^2) + 4\ t22\ (-3 - 3\ \alpha + \alpha^2) +$$
$$t22^2\ (24 + 24\ \alpha + 11\ \alpha^2)) + s^3\ (-6\ t22^4\ \alpha^2 - 6\ (1 + \alpha) - 32\ t22\ (1 + \alpha) +$$
$$t22^3\ (24 + 24\ \alpha - 11\ \alpha^2) + t22^2\ (24 + 24\ \alpha + 13\ \alpha^2)))) -$$
$$m^2\ (t12 - t22)^2\ (2\ s\ t12^4\ (1 + s\ (-1 + 3\ t22))\ (-\alpha^2 - s\ \alpha^2 + s^2\ (1 + \alpha)) +$$
$$t22\ (-4 - 4\ \alpha + (-1 + t22 - t22^2)\ \alpha^2 + 2\ s^4\ t22\ (-3 + t22 + 2\ t22^2)\ (1 + \alpha) -$$
$$s^2\ (6 + 6\ \alpha + 4\ \alpha^2 + 4\ t22^2\ \alpha^2 + 6\ t22^3\ \alpha^2 + t22\ (6 + 6\ \alpha - 11\ \alpha^2)) -$$
$$s\ (4 + 4\ \alpha + 3\ \alpha^2 + 5\ t22^2\ \alpha^2 + 2\ t22^3\ \alpha^2 + t22\ (8 + 8\ \alpha - 7\ \alpha^2)) + s^3\ (8\ t22^2\ (1 +$$
$$\alpha) + t22^3\ (2 + 2\ \alpha - 4\ \alpha^2) + 5\ t22\ (-2 - 2\ \alpha + \alpha^2) - 2\ (2 + 2\ \alpha + \alpha^2))) +$$
$$t12^2\ (-(1 + t22)\ \alpha^2 + 6\ s^4\ (-1 - 3\ t22 + 4\ t22^2 + 4\ t22^3)\ (1 + \alpha) -$$
$$s\ (8 + 8\ \alpha + (-3 + 5\ t22 + 18\ t22^2)\ \alpha^2) +$$
$$s^3\ (14\ t22\ \alpha^2 - 24\ t22^3\ \alpha^2 - 22\ (1 + \alpha) + t22^2\ (48 + 48\ \alpha - 9\ \alpha^2)) +$$
$$s^2\ (-27\ t22^2\ \alpha^2 - 24\ t22^3\ \alpha^2 + 2\ (-9 - 9\ \alpha + 2\ \alpha^2) + 2\ t22\ (6 + 6\ \alpha + 5\ \alpha^2))) +$$
$$t12^3\ (\alpha^2 + s\ (-1 + 14\ t22)\ \alpha^2 + s^2\ (-5 + 9\ t22 + 24\ t22^2)\ \alpha^2 - 6\ s^4\ (-1 + 4\ t22^2)$$
$$(1 + \alpha) + s^3\ (8 + 8\ \alpha - 3\ \alpha^2 + 24\ t22^2\ \alpha^2 - t22\ (24 + 24\ \alpha + 5\ \alpha^2))) +$$
$$t12\ (4 + 4\ \alpha + (1 + t22^2)\ \alpha^2 - 2\ s^4\ (-1 - 6\ t22 - 3\ t22^2 + 12\ t22^3 + 3\ t22^4)$$
$$(1 + \alpha) + s\ (12 + 12\ \alpha + \alpha^2 - t22^2\ \alpha^2 + 14\ t22^3\ \alpha^2 + t22\ (8 + 8\ \alpha - 2\ \alpha^2)) + s^3$$
$$(12 + 12\ \alpha + \alpha^2 + 6\ t22^4\ \alpha^2 + t22\ (28 + 28\ \alpha - \alpha^2) - t22^2\ (24 + 24\ \alpha + 17\ \alpha^2) +$$
$$t22^3\ (-24 - 24\ \alpha + 19\ \alpha^2)) + s^2\ (18 + 18\ \alpha + \alpha^2 + 33\ t22^3\ \alpha^2 +$$
$$6\ t22^4\ \alpha^2 - 3\ t22\ (-4 - 4\ \alpha + \alpha^2) - t22^2\ (12 + 12\ \alpha + 19\ \alpha^2)))))) \Big/$$
$$((1 + s)\ (-1 + m\ (t12 - t22) + t22)\ (m\ (t12 - t22) + t22)\ ((-1 + m)\ t12 - m\ t22)$$
$$(1 + (-1 + m)\ t12 - m\ t22)$$
$$((-1 + m)\ m\ s\ (2 + s)\ t12^2 +$$
$$t12\ (-1 - 2\ s\ t22 - s^2\ t22 - 2\ m^2\ s\ (2 + s)\ t22 + m\ (2 + 2\ s + s^2 + 4\ s\ t22 + 2\ s^2\ t22)) +$$
$$t22\ ((1 + s)^2 + m^2\ s\ (2 + s)\ t22 - m\ (2 + 2\ s\ (1 + t22) + s^2\ (1 + t22))))\ \alpha\ (2 + \alpha))),$$
$$dd2 \to ((-1 + t22)\ t22\ (-m^6\ s\ (t12 - t22)^6\ (-2\ \alpha^2 + s\ (-3 - 2\ t12 + 2\ t22)\ \alpha^2 +$$
$$2\ s^3\ (1 + t12 - t22)\ (1 + \alpha) + s^2\ (4 + 4\ \alpha + (-1 - 2\ t12 + 2\ t22)\ \alpha^2)) +$$
$$m^5\ (t12 - t22)^5\ (2\ \alpha^2 + 2\ s\ (2 - t12 + t22)\ \alpha^2 +$$
$$2\ s^4\ (-2 + t12 + 3\ t12^2 - t22 - 6\ t12\ t22 + 3\ t22^2)\ (1 + \alpha) -$$
$$3\ s^2\ (4 + 4\ \alpha + (-1 + t12 + 2\ t12^2 - t22 - 4\ t12\ t22 + 2\ t22^2)\ \alpha^2) +$$
$$s^3\ (-12 - 12\ \alpha + \alpha^2 - 6\ t12^2\ \alpha^2 - 6\ t22^2\ \alpha^2 + t22$$

$$(-4 - 4\alpha + \alpha^2) + t12\,(4 + 4\alpha + (-1 + 12\,t22)\,\alpha^2))) -$$
$$m^4\,(t12 - t22)^4\,(3\,(t12 - t22)\,\alpha^2 + 2\,s^4\,(1 + 3\,t12^3 + 5\,t22 - 12\,t12^2$$
$$t22 - 3\,t22^2 - 3\,t22^3 + 2\,t12\,(-2 + t22 + 6\,t22^2))\,(1 + \alpha) +$$
$$s\,(12 + 12\alpha + (2 + 4\,t12^2 + t12\,(3 - 2\,t22) - 9\,t22 + 4\,t22^2)\,\alpha^2) -$$
$$3\,s^2\,(-6 - 6\alpha - \alpha^2 + 2\,t12^3\,\alpha^2 - 3\,t22^2\,\alpha^2 - 2\,t22^3\,\alpha^2 - t12^2\,(1 + 8\,t22)$$
$$\alpha^2 + t22\,(-6 - 6\alpha + 4\alpha^2) + t12\,(6 + 6\alpha + (-1 + t22 + 8\,t22^2)\,\alpha^2)) +$$
$$s^3\,(10 + 10\alpha + \alpha^2 - 6\,t12^3\,\alpha^2 + 6\,t22^3\,\alpha^2 + t22\,(20 + 20\alpha - 6\alpha^2) + t22^2$$
$$(-6 - 6\alpha + 5\alpha^2) + t12^2\,(-6 - 6\alpha + (-1 + 24\,t22)\,\alpha^2) - t12$$
$$(16 + 16\alpha - 3\alpha^2 + 24\,t22^2\,\alpha^2 + t22\,(-8 - 8\alpha + \alpha^2))))) + (-1 + t12)\,t12$$
$$(-1 + t22)\,t22\,((t12 - t22)\,\alpha^2 + 2\,s^4\,(-1 + t12)\,(-1 + t12 - t22)\,t22\,(1 + \alpha) +$$
$$s\,(4 + 4\alpha - (-3\,t12 + 2\,t12^2 + t22 - 2\,t12\,t22 + 2\,t22^2)\,\alpha^2) +$$
$$s^2\,(-4\,t22^2\,\alpha^2 - 2\,t12^2\,(1 + t22)\,\alpha^2 + 6\,(1 + \alpha) + t22$$
$$(6 + 6\alpha + \alpha^2) + t12\,(-6 - 6\alpha + (2 + 3\,t22 + 2\,t22^2)\,\alpha^2)) +$$
$$s^3\,(2\,(1 + \alpha) + t22^2\,(2 + 2\alpha - 2\alpha^2) + t22\,(8 + 8\alpha + \alpha^2) + t12^2$$
$$(2 + 2\alpha - 2\,t22\,\alpha^2) + t12\,(2\,t22^2\,\alpha^2 - 4\,(1 + \alpha) + t22\,(-8 - 8\alpha + \alpha^2))))) +$$
$$m^3\,(t12 - t22)^3\,(2\,s^4\,(t12^4 + t12^3\,(1 - 10\,t22) + t12^2\,(-5 + 5\,t22 + 18\,t22^2) +$$
$$t12\,(3 + 8\,t22 - 11\,t22^2 - 10\,t22^3) + t22\,(-3 - 3\,t22 + 5\,t22^2 + t22^3))$$
$$(1 + \alpha) - 2\,(2 + 2\alpha + (1 - t22 + t12\,(-1 + 2\,t22))\,\alpha^2) +$$
$$2\,s\,(3\,t12^3\,\alpha^2 + t22^2\,\alpha^2 - 3\,t22^3\,\alpha^2 - t12^2\,(1 + 7\,t22)\,\alpha^2 + 2\,t22$$
$$(-4 - 4\alpha + \alpha^2) - 2\,(2 + 2\alpha + \alpha^2) + t12\,(8 + 8\alpha + (2 - 4\,t22 + 7\,t22^2)\,\alpha^2)) -$$
$$s^2\,(2\,t12^4\,\alpha^2 + 15\,t22^3\,\alpha^2 + 2\,t22^4\,\alpha^2 - t12^3\,(3 + 20\,t22)\,\alpha^2 + t22^2$$
$$(6 + 6\alpha - 9\alpha^2) - 3\,t22\,(-8 - 8\alpha + \alpha^2) + 3\,(2 + 2\alpha + \alpha^2) + 3$$
$$t12^2\,(2 + 2\alpha + (-1 + 5\,t22 + 12\,t22^2)\,\alpha^2) - t12$$
$$(27\,t22^2\,\alpha^2 + 20\,t22^3\,\alpha^2 + 3\,(8 + 8\alpha + \alpha^2) - 6\,t22\,(-2 - 2\alpha + 3\alpha^2))) -$$
$$s^3\,(2 + 2\alpha + \alpha^2 + 2\,t12^4\,\alpha^2 + 2\,t22^4\,\alpha^2 + t22^2\,(2 + 2\alpha - 7\alpha^2) + t22$$
$$(20 + 20\alpha - \alpha^2) + t22^3\,(-8 - 8\alpha + 9\alpha^2) + t12^3\,(8 + 8\alpha + (3 - 20\,t22)\,\alpha^2) +$$
$$t12^2\,(36\,t22^2\,\alpha^2 + t22\,(-32 - 32\alpha + \alpha^2) - 5\,(-2 - 2\alpha + \alpha^2)) - t12\,(20 + 20\alpha +$$
$$\alpha^2 + 20\,t22^3\,\alpha^2 - 2\,t22\,(-6 - 6\alpha + 7\alpha^2) + t22^2\,(-32 - 32\alpha + 13\alpha^2))))) +$$
$$m\,(t12 - t22)\,(2\,s\,t12^4\,(-1 - 2\,(-1 + s)\,t22 + 3\,s\,t22^2)\,(-\alpha^2 - s\,\alpha^2 + s^2\,(1 + \alpha)) +$$
$$(1 + s)\,(-1 + t22)\,t22\,(-2 - 2\alpha - t22\,\alpha^2 + 2\,s^3\,t22^2\,(1 + \alpha) + s^2$$
$$t22\,(2 + 2\alpha + (1 - 2\,t22)\,\alpha^2) - 2\,s\,t22\,(1 + \alpha + t22\,\alpha^2)) +$$
$$t12^3\,((-1 + 2\,t22)\,\alpha^2 - 6\,s^4\,t22\,(-2 + t22 + 2\,t22^2)\,(1 + \alpha) + s$$
$$(2 + 2\alpha + (-5 + 2\,t22 + 10\,t22^2)\,\alpha^2) + s^3\,(-3 + 4\,t22)$$
$$(3\,t22^2\,\alpha^2 - 2\,(1 + \alpha) + 2\,t22\,(-3 - 3\alpha + \alpha^2)) + s^2$$
$$(6 + 6\alpha - 4\alpha^2 + 9\,t22^2\,\alpha^2 + 12\,t22^3\,\alpha^2 - 6\,t22\,(1 + \alpha + \alpha^2))) +$$
$$t12\,(2 + 2\alpha + t22^2\,(-1 + 2\,t22)\,\alpha^2 - 2\,s^4\,t22\,(-2 - 3\,t22 + 2\,t22^2 + 4\,t22^3)$$
$$(1 + \alpha) + s\,(6\,t22^3\,\alpha^2 + 4\,t22^4\,\alpha^2 + 6\,(1 + \alpha) - t22^2\,(2 + 2\alpha + 7\alpha^2)) + s^3$$
$$(2\,(1 + \alpha) + t22^2\,(2 + 2\alpha - 5\alpha^2) + 2\,t22\,(9 + 9\alpha + \alpha^2) - 2\,t22^3$$
$$(11 + 11\alpha + 2\alpha^2) + t22^4\,(-4 - 4\alpha + 8\alpha^2)) + s^2\,(12\,t22^4\,\alpha^2 + 6\,(1 + \alpha) -$$
$$6\,t22^3\,(1 + \alpha) + 2\,t22\,(9 + 9\alpha + \alpha^2) - t22^2\,(18 + 18\alpha + 11\alpha^2))) +$$
$$t12^2\,(-2 - 2\alpha - (-1 + t22 + 2\,t22^2)\,\alpha^2 + 6\,s^4\,t22\,(-2 - t22 + 3\,t22^2 + t22^3)$$
$$(1 + \alpha) + s\,(-8 - 8\alpha + 3\alpha^2 - 4\,t22^2\,\alpha^2 - 10\,t22^3\,\alpha^2 + t22\,(2 + 2\alpha + 3\alpha^2)) + s^2$$
$$(-21\,t22^3\,\alpha^2 - 6\,t22^4\,\alpha^2 + 2\,(-6 - 6\alpha + \alpha^2) + 4\,t22\,(-3 - 3\alpha + \alpha^2) +$$
$$t22^2\,(24 + 24\alpha + 11\alpha^2)) + s^3\,(-6\,t22^4\,\alpha^2 - 6\,(1 + \alpha) - 32\,t22\,(1 + \alpha) +$$
$$t22^3\,(24 + 24\alpha - 11\alpha^2) + t22^2\,(24 + 24\alpha + 13\alpha^2))))) +$$
$$m^2\,(t12 - t22)^2\,(2\,s\,t12^4\,(1 + s\,(-1 + 3\,t22))\,(-\alpha^2 - s\,\alpha^2 + s^2\,(1 + \alpha)) +$$
$$t22\,(-4 - 4\alpha + (-1 + t22 - t22^2)\,\alpha^2 + 2\,s^4\,t22\,(-3 + t22 + 2\,t22^2)\,(1 + \alpha) - s^2$$
$$(6 + 6\alpha + 4\alpha^2 + 4\,t22^2\,\alpha^2 + 6\,t22^3\,\alpha^2 + t22\,(6 + 6\alpha - 11\alpha^2)) - s$$
$$(4 + 4\alpha + 3\alpha^2 + 5\,t22^2\,\alpha^2 + 2\,t22^3\,\alpha^2 + t22\,(8 + 8\alpha - 7\alpha^2)) + s^3\,(8\,t22^2$$
$$(1 + \alpha) + t22^3\,(2 + 2\alpha - 4\alpha^2) + 5\,t22\,(-2 - 2\alpha + \alpha^2) - 2\,(2 + 2\alpha + \alpha^2))) +$$

```
t12^2 (-(1 + t22) α^2 + 6 s^4 (-1 - 3 t22 + 4 t22^2 + 4 t22^3) (1 + α) - s
      (8 + 8 α + (-3 + 5 t22 + 18 t22^2) α^2) + s^3
      (14 t22 α^2 - 24 t22^3 α^2 - 22 (1 + α) + t22^2 (48 + 48 α - 9 α^2)) + s^2
      (-27 t22^2 α^2 - 24 t22^3 α^2 + 2 (-9 - 9 α + 2 α^2) + 2 t22 (6 + 6 α + 5 α^2))) +
t12^3 (α^2 + s (-1 + 14 t22) α^2 + s^2 (-5 + 9 t22 + 24 t22^2) α^2 - 6 s^4 (-1 + 4 t22^2)
      (1 + α) + s^3 (8 + 8 α - 3 α^2 + 24 t22^2 α^2 - t22 (24 + 24 α + 5 α^2))) +
t12 (4 + 4 α + (1 + t22^2) α^2 - 2 s^4 (-1 - 6 t22 - 3 t22^2 + 12 t22^3 + 3 t22^4)
      (1 + α) + s (12 + 12 α + α^2 - t22^2 α^2 + 14 t22^3 α^2 + t22 (8 + 8 α - 2 α^2)) + s^3
      (12 + 12 α + α^2 + 6 t22^4 α^2 + t22 (28 + 28 α - α^2) - t22^2 (24 + 24 α + 17 α^2) +
        t22^3 (-24 - 24 α + 19 α^2)) + s^2 (18 + 18 α + α^2 + 33 t22^3 α^2 +
        6 t22^4 α^2 - 3 t22 (-4 - 4 α + α^2) - t22^2 (12 + 12 α + 19 α^2)))))) /
((1 + s) (-1 + m (t12 - t22) + t22) (m (t12 - t22) + t22)
  ((-1 + m) t12 - m t22)
  (1 + (-1 + m) t12 - m t22)
  ((-1 + m) m s (2 + s) t12^2 +
    t12 (-1 - 2 s t22 - s^2 t22 - 2 m^2 s (2 + s) t22 + m (2 + 2 s + s^2 + 4 s t22 + 2 s^2 t22)) +
    t22 ((1 + s)^2 + m^2 s (2 + s) t22 - m (2 + 2 s (1 + t22) + s^2 (1 + t22)))) α (2 +
    α))};
```

```
Exponent[Numerator[dd2] /. dd12gent12, {p12, t12, dd1, p22, t22, d22}]
```

```
{0, 7, 0, 0, 9, 0}
```

Now we can substitute dd12gent12 into p12p22gentdd to obtain (p12, p22) as functions of (t12, t22):

```
Simplify[p12p22gentdd /. dd12gent12]
```

```
p12p22gent12 =
  {p12 → -((-m^6 s (t12 - t22)^6 (2 s^3 t12 (t12 - t22) (1 + α) + 2 α (1 + t22 α) + s α
          (3 - 2 t12^2 α + 2 t22 α + t12 (α + 2 t22 α)) + s^2 (α - 2 t12^2 α^2 - 2 t22
            (1 + α) + t12 (2 + 2 α + (1 + 2 t22) α^2))) + (-1 + t12) t12 (-1 + t22) t22
       (2 s^4 (-1 + t12) t22 (1 + t12^2 - t12 (2 + t22)) (1 + α) + (t12 - t22) α (1 + t12 α) -
         s (1 + t12 α) (2 t12^2 α + t22 (2 + 5 α) - t12 (2 + (3 + 4 t22) α)) - s^2 (-1 + t12)
          (2 t12^2 (1 + t22) α^2 - 3 t22 (2 + 3 α) + t12 (6 + 6 α - t22 (5 + 2 t22) α^2)) -
         s^3 (-1 + t12) (-t22 (6 + 7 α) + 2 t12^2 (-1 - α + t22 α^2) + t12
            (-2 t22^2 α^2 + 2 (1 + α) + t22 (10 + 10 α - α^2)))) + m^5 (t12 - t22)^5
       (2 s^4 t12 (3 t12^2 - 2 t12 (1 + 3 t22) + t22 (2 + 3 t22)) (1 + α) - 2 α (1 + t22 α) -
         2 s α (2 - 2 t12^2 α + 3 t22^2 α + t22 (3 + α) + t12 (-3 + α - t22 α)) -
         3 s^2 (t22 (-2 + α) + α + 2 t12^3 α^2 + 2 t22^2 α^2 - t12^2
            (3 + 4 t22) α^2 + t12 (2 - α + (1 + t22 + 2 t22^2) α^2)) -
         s^3 (6 t12^3 α^2 + t12^2 (2 + 2 α - (5 + 12 t22) α^2) - (1 + 3 t22)
            (-α + 2 t22 (1 + α)) + t12 (2 - α + α^2 + 6 t22^2 α^2 + t22 (4 + 4 α + 5 α^2)))) -
      m^4 (t12 - t22)^4 (2 s^4 t12 (1 + 3 t12^3 - 2 t22 - 6 t22^2 - 3 t22^3 - 3 t12^2
            (1 + 4 t22) + t12 (-1 + 11 t22 + 12 t22^2)) (1 + α) -
        (t12 - t22) α (5 + t12 α + 4 t22 α) + s (10 t12^3 α^2 + 6 t22^3 α^2 + 6 t22^2
            α (1 + α) + 2 (2 + α) + t12^2 α (6 - 7 (1 + 2 t22) α) + t22
            (-10 + 3 α - 2 α^2) + t12 (2 - (5 + 18 t22) α + (7 - 8 t22) t22 α^2)) -
        s^2 (6 t12^4 α^2 - 6 t22^3 α^2 - 3 t12^3 (5 + 8 t22) α^2 - 3 (2 + α) + t22^2
            (12 + 3 α - 2 α^2) + 2 t22 (6 + α^2) + t12^2 (6 - 3 α + (5 + 33 t22 + 24 t22^2) α^2) +
          t12 (-3 t22^2 α^2 - 6 t22^3 α^2 + α (3 + α) - 3 t22 (6 - 3 α + 4 α^2))) +
```

$$s^3\,(2+2\,t22\,(-1+\alpha)+\alpha-6\,t12^4\,\alpha^2-6\,t22^3\,(1+\alpha)-3\,t22^2\,(2+\alpha)+t12^3\,(-12-12\,\alpha+(5+24\,t22)\,\alpha^2)+t12^2\,(2+5\,\alpha+\alpha^2-24\,t22^2\,\alpha^2+t22\,(20+20\,\alpha-19\,\alpha^2))+t12\,(2+\alpha-\alpha^2+6\,t22^3\,\alpha^2+t22\,(-8-17\,\alpha+2\,\alpha^2)+t22^2\,(6+6\,\alpha+11\,\alpha^2))))+$$

$$m\,(t12-t22)\,(2\,t12\,t22-2\,t22^2-2\,t12\,t22^2+2\,t22^3+t12^2\,\alpha-t12^3\,\alpha-2\,t12\,t22\,\alpha+3\,t12^2\,t22\,\alpha+2\,t12^3\,t22\,\alpha-t22^2\,\alpha+t12\,t22^2\,\alpha-6\,t12^2\,t22^2\,\alpha+t22^3\,\alpha+2\,t12\,t22^3\,\alpha+t12^3\,\alpha^2-t12^4\,\alpha^2-2\,t12^2\,t22\,\alpha^2+t12^3\,t22\,\alpha^2+2\,t12^4\,t22\,\alpha^2-t12\,t22^2\,\alpha^2+3\,t12^2\,t22^2\,\alpha^2-4\,t12^3\,t22^2\,\alpha^2+t12\,t22^3\,\alpha^2+2\,s^4\,(-1+t12)\,t12\,t22\,(2-2\,t22^2-t22^3+t12^3\,(-2+3\,t22)-3\,t12^2\,(-2+t22+2\,t22^2)+3\,t12\,(-2+2\,t22^2+t22^3))\,(1+\alpha)-s^3\,(-1+t12)\,((-1+t22)\,t22^2\,(2+\alpha)+2\,t12^4\,(1+\alpha+3\,t22^2\,\alpha^2-2\,t22\,(1+\alpha+\alpha^2))+t12\,t22\,(t22\,(2+\alpha)^2-2\,(6+7\,\alpha)-2\,t22^3\,(1+\alpha+\alpha^2)+t22^2\,(16+19\,\alpha+\alpha^2))+t12^3\,(-12\,t22^3\,\alpha^2-4\,(1+\alpha)+6\,t22\,(-3-3\,\alpha+\alpha^2)+t22^2\,(30+30\,\alpha+\alpha^2))+t12^2\,(6\,t22^4\,\alpha^2+2\,(1+\alpha)+t22\,(34+36\,\alpha-2\,\alpha^2)-3\,t22^2\,(8+9\,\alpha+2\,\alpha^2)+t22^3\,(-24-24\,\alpha+5\,\alpha^2)))-s^2\,(-1+t12)\,(2\,t12^4\,(-1+3\,t22^2)\,\alpha^2+3\,(-1+t22)\,t22^2\,(2+\alpha)+t12^2\,(15\,t22^3\,\alpha^2+6\,t22^4\,\alpha^2+6\,(1+\alpha)+t22\,(18+24\,\alpha-10\,\alpha^2)-t22^2\,(30+39\,\alpha+4\,\alpha^2))+t12\,t22\,(t22^2\,(9-5\,\alpha)\,\alpha-6\,(2+3\,\alpha)+t22\,(18+18\,\alpha+5\,\alpha^2))+t12^3\,(-15\,t22^2\,\alpha^2-12\,t22^3\,\alpha^2+2\,(-3-3\,\alpha+\alpha^2)+2\,t22\,(3+3\,\alpha+7\,\alpha^2)))+s\,(2\,t12^5\,(1-2\,t22)\,\alpha^2+3\,(-1+t22)\,t22^2\,(2+\alpha)+t12^4\,(2+2\,\alpha+(-5-2\,t22+16\,t22^2)\,\alpha^2)+t12^2\,(4+5\,\alpha-2\,t22^4\,\alpha^2+2\,t22^3\,\alpha\,(-3+8\,\alpha)+t22\,(-4+7\,\alpha-10\,\alpha^2)+t22^2\,(-2-14\,\alpha+\alpha^2))+t12\,t22\,(2-6\,\alpha+2\,t22^3\,\alpha^2+t22^2\,(-4+6\,\alpha-5\,\alpha^2)+t22\,(4+5\,\alpha+3\,\alpha^2))+t12^3\,(-6-7\,\alpha+2\,t22^2\,(3-11\,\alpha)\,\alpha+3\,\alpha^2-10\,t22^3\,\alpha^2+t22\,(4+2\,\alpha+17\,\alpha^2)))))+$$

$$m^3\,(t12-t22)^3\,(2\,s^4\,t12\,(-2+t12^4-10\,t12^3\,t22+5\,t22^2+6\,t22^3+t22^4+t12^2\,(-5+14\,t22+18\,t22^2)-2\,t12\,(-3+2\,t22+10\,t22^2+5\,t22^3))\,(1+\alpha)-2\,(2+\alpha+t12^3\,\alpha^2+t22^3\,\alpha^2+t22^2\,\alpha\,(2+\alpha)+t12^2\,\alpha\,(2-t22\,\alpha)-t22\,(3+2\,\alpha+\alpha^2)+t12\,(-1+t22\,(-6+\alpha)\,\alpha-3\,t22^2\,\alpha^2))+2\,s\,(4\,t12^4\,\alpha^2-t22^4\,\alpha^2-2\,(2+\alpha)-t22^3\,\alpha\,(1+3\,\alpha)+t12^3\,\alpha\,(1-(2+15\,t22)\,\alpha)+t22^2\,(7+\alpha^2)+t22\,(3+3\,\alpha+\alpha^2)+t12^2\,(1-3\,\alpha^2+7\,t22^2\,\alpha^2+t22\,\alpha\,(-9+13\,\alpha))+t12\,(5+\alpha+t22^2\,(9-4\,\alpha)\,\alpha+\alpha^2+5\,t22^3\,\alpha^2-2\,t22\,(4-2\,\alpha+\alpha^2)))-s^3\,(2+t22^2\,(-4+\alpha)+\alpha+2\,t12^5\,\alpha^2-2\,t22^4\,(1+\alpha)+t22\,(2+\alpha)-t22^3\,(6+5\,\alpha)+t12^4\,(10+10\,\alpha+(1-20\,t22)\,\alpha^2)+t12^2\,(-22-23\,\alpha+5\,\alpha^2-20\,t22^3\,\alpha^2+t22^2\,(32+32\,\alpha-31\,\alpha^2)+t22\,(46+55\,\alpha+2\,\alpha^2))+t12\,(6+9\,\alpha-\alpha^2+2\,t22^4\,\alpha^2+t22^2\,(-20-29\,\alpha+3\,\alpha^2)-t22\,(10+16\,\alpha+3\,\alpha^2)+t22^3\,(8+8\,\alpha+11\,\alpha^2))+t12^3\,(4+3\,\alpha-7\,\alpha^2+36\,t22^2\,\alpha^2+t22\,(-48-48\,\alpha+19\,\alpha^2)))+s^2\,(-2\,t12^5\,\alpha^2-2\,t22^4\,\alpha^2+t12^4\,(7+20\,t22)\,\alpha^2-3\,(2+\alpha)-3\,t22\,(2+\alpha)+t12^3\,\alpha\,(3+(5-49\,t22-36\,t22^2)\,\alpha)+t22^3\,(6+3\,\alpha-4\,\alpha^2)+t22^2\,(24+9\,\alpha+4\,\alpha^2)+t12\,(-t22^3\,\alpha^2-2\,t22^4\,\alpha^2+t22^2\,(-24+3\,\alpha-17\,\alpha^2)+3\,(2-\alpha+\alpha^2)+t22\,(6+24\,\alpha+\alpha^2))+t12^2\,(6+9\,\alpha-11\,\alpha^2+45\,t22^2\,\alpha^2+20\,t22^3\,\alpha^2+t22\,(-6-33\,\alpha+22\,\alpha^2))))+$$

$$m^2\,(t12-t22)^2\,(2\,s\,t12^5\,(1+s\,(-1+3\,t22))\,(-\alpha^2-s\,\alpha^2+s^2\,(1+\alpha))+t12^4\,(\alpha^2+s\,(-3+20\,t22)\,\alpha^2+3\,s^2\,(-3+7\,t22+8\,t22^2)\,\alpha^2-2\,s^4\,(-4+3\,t22+12\,t22^2)\,(1+\alpha)+s^3\,(24\,t22^2\,\alpha^2+t22\,(-30-30\,\alpha+\alpha^2)-5\,(-2-2\,\alpha+\alpha^2)))+t22\,(-4-\alpha-t22^2\,\alpha\,(1+2\,\alpha)+t22\,(6+3\,\alpha+2\,\alpha^2)+s^3\,(2\,t22^2+2\,t22^3\,(1+\alpha)-2\,(2+\alpha)+t22\,(2+\alpha))+s^2\,(-2\,t22^3\,\alpha^2-6\,(2+\alpha)+3\,t22\,(2+\alpha)+2\,t22^2\,(6+3\,\alpha+\alpha^2))+s\,(-t22^2\,(-4+\alpha)-2\,t22^3\,\alpha^2-7\,(2+\alpha)+t22\,(16+11\,\alpha+2\,\alpha^2)))+$$

$t12\,(4 + \alpha - t22^2\,(-9 + \alpha)\,\alpha + 3\,t22^3\,\alpha^2 + 2\,s^4\,(-1 - 3\,t22 + 3\,t22^2 + 4\,t22^3 + 2\,t22^4)$

$(1 + \alpha) - t22\,(2 + \alpha)^2 - s^3\,(6 + 7\,\alpha + t22\,(6 + 11\,\alpha - 2\,\alpha^2) +$

$4\,t22^4\,(1 + \alpha + \alpha^2) - t22^2\,(26 + 35\,\alpha + 3\,\alpha^2) + t22^3\,(-8 - 11\,\alpha + 4\,\alpha^2)) + s$

$(2 - 3\,\alpha - t22^3\,(-6 + \alpha)\,\alpha + 4\,t22^4\,\alpha^2 + t22^2\,(-20 + 7\,\alpha - 9\,\alpha^2) +$

$t22\,(16 + 6\,\alpha + 5\,\alpha^2)) - s^2\,(6 + 9\,\alpha + t22^2\,\alpha\,(-27 + 5\,\alpha) +$

$t22^3\,(12 + 3\,\alpha + 8\,\alpha^2) - t22\,(24 + 9\,\alpha + 8\,\alpha^2))) +$

$t12^3\,(6\,s^4\,(-2 - 2\,t22 + 7\,t22^2 + 4\,t22^3)\,(1 + \alpha) + \alpha\,(1 + \alpha - 5\,t22\,\alpha) - s$

$(4 + (5 - 6\,t22)\,\alpha + (-11 + 17\,t22 + 30\,t22^2)\,\alpha^2) + s^2\,(-18 - 21\,\alpha + 14\,\alpha^2 -$

$57\,t22^2\,\alpha^2 - 24\,t22^3\,\alpha^2 + t22\,(24 + 33\,\alpha + 2\,\alpha^2)) + s^3\,(-32 - 33\,\alpha +$

$4\,\alpha^2 - 24\,t22^3\,\alpha^2 + t22^2\,(60 + 60\,\alpha - 27\,\alpha^2) + t22\,(24 + 27\,\alpha + 14\,\alpha^2))) -$

$t12^2\,(2 + (-1 + 9\,t22)\,\alpha - (-1 + 2\,t22 + t22^2)\,\alpha^2 + 2\,s^4$

$(-4 - 9\,t22 + 12\,t22^2 + 15\,t22^3 + 3\,t22^4)\,(1 + \alpha) + s$

$(-4 - 11\,\alpha + t22^2\,(18 - 35\,\alpha)\,\alpha + 5\,\alpha^2 - 2\,t22^3\,\alpha^2 + t22\,(4 + 13\,\alpha + 10\,\alpha^2)) + s^3$

$(-26 - 28\,\alpha + \alpha^2 - 6\,t22^4\,\alpha^2 + t22^3\,(12 + 12\,\alpha - 25\,\alpha^2) + t22^2\,(84 + 93\,\alpha - \alpha^2) +$

$t22\,(-16 - 16\,\alpha + 11\,\alpha^2)) + s^2\,(-24 - 30\,\alpha + 5\,\alpha^2 - 27\,t22^3\,\alpha^2 -$

$6\,t22^4\,\alpha^2 + t22^2\,(12 + 39\,\alpha - 35\,\alpha^2) + t22\,(30 + 30\,\alpha + 23\,\alpha^2))))) \Big/$

$((1 + s)\,(-1 + m\,(t12 - t22) + t22)\,(m\,(t12 - t22) + t22)\,((-1 + m)\,t12 - m\,t22)$

$(1 + (-1 + m)\,t12 - m\,t22)$

$((-1 + m)\,m\,s\,(2 + s)\,t12^2 +$

$t12\,(-1 - 2\,s\,t22 - s^2\,t22 - 2\,m^2\,s\,(2 + s)\,t22 + m\,(2 + 2\,s + s^2 + 4\,s\,t22 + 2\,s^2\,t22)) +$

$t22\,((1 + s)^2 + m^2\,s\,(2 + s)\,t22 - m\,(2 + 2\,s\,(1 + t22) + s^2\,(1 + t22))))\,\alpha\,(2 + \alpha))),$

$p22 \to -((-m^6\,s\,(t12 - t22)^6\,(2\,s^3\,(t12 - t22)\,(-1 + t22)\,(1 + \alpha) + 2\,\alpha\,(1 + t12\,\alpha) +$

$s\,\alpha\,(3 - 2\,t12\,(-2 + t22)\,\alpha - t22\,\alpha + 2\,t22^2\,\alpha) +$

$s^2\,(\alpha + 2\,t22^2\,\alpha^2 + t22\,(2 + 2\,\alpha - \alpha^2) - 2\,t12\,(1 + \alpha + (-1 + t22)\,\alpha^2))) +$

$m^5\,(t12 - t22)^5\,(2\,s^4\,(-1 + t22)\,(3\,t12^2 - 2\,t12\,(1 + 3\,t22) + t22\,(2 + 3\,t22))$

$(1 + \alpha) - 2\,\alpha\,(1 + t12\,\alpha) +$

$2\,s\,\alpha\,(-2 + t22\,(-3 + \alpha) + 3\,t12^2\,\alpha - 2\,t22^2\,\alpha - t12\,(-3 + (3 + t22)\,\alpha)) -$

$3\,s^2\,(\alpha + 2\,t12^2\,(-2 + t22)\,\alpha^2 + t22^2\,\alpha^2 + 2\,t22^3\,\alpha^2 + t22\,(2 + 5\,\alpha - \alpha^2) + t12$

$(-2 - 5\,\alpha + (2 + 3\,t22 - 4\,t22^2)\,\alpha^2)) + s^3\,(-\alpha - 6\,t22^3\,\alpha^2 + t22$

$(-10 - 13\,\alpha + \alpha^2) + t22^2\,(2 + 2\,\alpha + \alpha^2) - 6\,t12^2\,(1 + \alpha + (-1 + t22)\,\alpha^2) +$

$t12\,(10 + 13\,\alpha - 2\,\alpha^2 + 12\,t22^2\,\alpha^2 + t22\,(4 + 4\,\alpha - 7\,\alpha^2)))) +$

$(-1 + t12)\,t12\,(-1 + t22)\,t22\,(-2\,s^4\,t22\,(1 - t12^2\,(-1 + t22) - t22 -$

$t22^2 + t12\,(-2 + 2\,t22 + t22^2))\,(1 + \alpha) + (t12 - t22)\,\alpha\,(1 + t22\,\alpha) -$

$s\,(1 + t22\,\alpha)\,(t12\,(2 + \alpha - 4\,t22\,\alpha) + t22\,(-2 + \alpha + 2\,t22\,\alpha)) +$

$s^2\,t22\,(-3\,\alpha - 2\,t12^2\,(-1 + t22)\,\alpha^2 - 4\,t22^2\,\alpha^2 + t22$

$(6 + 6\,\alpha + \alpha^2) + t12\,(-6 - 3\,\alpha + (-4 + 5\,t22 + 2\,t22^2)\,\alpha^2)) +$

$s^3\,t22\,(-4 - 5\,\alpha - 2\,t12^2\,(-1 + t22)\,\alpha^2 + t22^2\,(2 + 2\,\alpha - 2\,\alpha^2) + t22$

$(8 + 8\,\alpha + \alpha^2) + t12\,(4 + 5\,\alpha - 2\,\alpha^2 + 2\,t22^2\,\alpha^2 + t22\,(-10 - 10\,\alpha + \alpha^2)))) -$

$m^4\,(t12 - t22)^4\,(-2\,s^4\,(-1 + t22)\,(-3\,t12^3 + 3\,t12^2\,(1 + 4\,t22) - t12$

$t22\,(11 + 12\,t22) + t22\,(1 + 6\,t22 + 3\,t22^2))\,(1 + \alpha) -$

$(t12 - t22)\,\alpha\,(5 + 4\,t12\,\alpha + t22\,\alpha) + s\,(-3\,t22^2\,(-2 + \alpha)\,\alpha + 6\,t12^3\,\alpha^2 +$

$10\,t22^3\,\alpha^2 + 2\,(2 + \alpha) + t22\,(2 + 15\,\alpha) - 2\,t12^2\,\alpha\,(-3 + (5 + 4\,t22)\,\alpha) -$

$t12\,(10 + (17 + 18\,t22)\,\alpha + (2 - 19\,t22 + 14\,t22^2)\,\alpha^2)) +$

$s^3\,(2 + \alpha + 6\,t22^4\,\alpha^2 + t22^2\,(14 + 17\,\alpha - 6\,\alpha^2) + t22\,(8 + 12\,\alpha + \alpha^2) + t22^3$

$(-12 - 12\,\alpha + 5\,\alpha^2) - 6\,t12^3\,(1 + \alpha + (-1 + t22)\,\alpha^2) + t12^2$

$(18 + 21\,\alpha - 2\,\alpha^2 + 24\,t22^2\,\alpha^2 + t22\,(6 + 6\,\alpha - 19\,\alpha^2)) - t12$

$(8 + 9\,\alpha + 2\,\alpha^2 + 24\,t22^3\,\alpha^2 - 5\,t22^2\,(2 + \alpha)^2 + t22\,(44 + 53\,\alpha - 11\,\alpha^2))) +$

$$
\begin{aligned}
& s^2 \left(-6\,t12^3 \left(-2+t22\right) \alpha^2 + 15\,t22^3 \alpha^2 + 6\,t22^4 \alpha^2 + 3 \left(2+\alpha\right) + t22^2 \left(6+15\,\alpha - 10\,\alpha^2\right) + t22 \left(6+18\,\alpha+\alpha^2\right) + t12^2 \left(12+21\,\alpha + \left(-8-27\,t22+24\,t22^2\right) \alpha^2\right) - \right.\\
& \left.\quad t12 \left(18+21\,\alpha+4\,\alpha^2+9\,t22^2 \alpha^2+24\,t22^3 \alpha^2-9\,t22 \left(-2-5\,\alpha+3\,\alpha^2\right)\right)\right)\Big) +\\
& m^3 \left(t12-t22\right)^3 \left(2\,s^4 \left(-1+t22\right) \left(t12^4-10\,t12^3\,t22+t22^2 \left(3+6\,t22+t22^2\right) + t12^2 \left(-3+14\,t22+18\,t22^2\right) - 2\,t12 \left(-1+2\,t22+10\,t22^2+5\,t22^3\right)\right) \left(1+\alpha\right) - \right.\\
& \quad 2 \left(2-t22+\alpha+2\,t22^2\,\alpha+t12^3\,\alpha^2+t22^3\,\alpha^2+t12^2\,\alpha \left(2+\alpha-3\,t22\,\alpha\right) - t12 \left(3+\left(2+6\,t22\right)\alpha+\left(1-t22+t22^2\right)\alpha^2\right)\right) +\\
& \quad 2\,s \left(t12^4\,\alpha^2-4\,t22^4\,\alpha^2-2 \left(2+\alpha\right)-t22^3\,\alpha \left(1+2\,\alpha\right)-t12^3\,\alpha \left(-1+\alpha+5\,t22\,\alpha\right)-t22 \left(1+\alpha+\alpha^2\right)+t22^2 \left(-1-8\,\alpha+3\,\alpha^2\right)-t12^2 \left(7+\left(8+9\,t22\right)\alpha+\left(5-16\,t22+7\,t22^2\right)\alpha^2\right)+t12 \left(9+5\,\alpha-9\,t22^2 \left(-1+\alpha\right)\alpha+3\,\alpha^2+15\,t22^3\,\alpha^2+t22 \left(8+20\,\alpha-2\,\alpha^2\right)\right)\right) +\\
& \quad s^2 \left(-2\,t12^4 \left(-2+t22\right)\alpha^2-17\,t22^4\,\alpha^2-2\,t22^5\,\alpha^2-3 \left(2+\alpha\right)+t22^3\,\alpha \left(-3+5\,\alpha\right)+t22^2\,\alpha \left(-15+7\,\alpha\right)-3\,t22 \left(4+3\,\alpha+\alpha^2\right)+t12^3 \left(6+9\,\alpha+\left(2-31\,t22+20\,t22^2\right)\alpha^2\right)-t12^2 \left(18+15\,\alpha+14\,\alpha^2-3\,t22^2\,\alpha^2+36\,t22^3\,\alpha^2+t22 \left(24+51\,\alpha-43\,\alpha^2\right)\right)+t12 \left(41\,t22^3\,\alpha^2+20\,t22^4\,\alpha^2+t22^2 \left(-6+21\,\alpha-44\,\alpha^2\right)+t22 \left(54+72\,\alpha+\alpha^2\right)+3 \left(4+\alpha+2\,\alpha^2\right)\right)\right) +\\
& \quad s^3 \left(-2-\alpha-2\,t22^5\,\alpha^2+t22^4 \left(10+10\,\alpha-9\,\alpha^2\right)+t22^2 \left(-16-21\,\alpha+\alpha^2\right)-t22 \left(4+3\,\alpha+\alpha^2\right)+t22^3 \left(4+3\,\alpha+7\,\alpha^2\right)-2\,t12^4 \left(1+\alpha+\left(-1+t22\right)\alpha^2\right)+t12^3 \left(6+7\,\alpha+2\,\alpha^2+20\,t22^2\,\alpha^2+t22 \left(8+8\,\alpha-21\,\alpha^2\right)\right)+t12 \left(-4-7\,\alpha+2\,\alpha^2+20\,t22^4\,\alpha^2+t22^2 \left(34+43\,\alpha-24\,\alpha^2\right)+t22 \left(50+56\,\alpha+3\,\alpha^2\right)+t22^3 \left(-48-48\,\alpha+11\,\alpha^2\right)\right)+t12^2 \left(2+3\,\alpha-6\,\alpha^2-36\,t22^3\,\alpha^2+t22 \left(-68-77\,\alpha+17\,\alpha^2\right)+t22^2 \left(32+32\,\alpha+17\,\alpha^2\right)\right)\right)\Big) + m^2 \left(t12-t22\right)^2\\
& \Big(2\,s\,t12^4 \left(1+s-2\,t22-4\,s\,t22+3\,s\,t22^2\right) \left(-\alpha^2-s\,\alpha^2+s^2 \left(1+\alpha\right)\right) +\\
& \quad t12^2 \left(-6-3 \left(1+3\,t22\right)\alpha+\left(-2+t22-t22^2\right)\alpha^2+6\,s^4 \left(1-8\,t22^2+3\,t22^3+4\,t22^4\right)\left(1+\alpha\right)-s^2 \left(33\,t22^3\,\alpha^2+24\,t22^4\,\alpha^2+t22^2 \left(-12+15\,\alpha-64\,\alpha^2\right)+6 \left(-1-2\,\alpha+\alpha^2\right)+4\,t22 \left(15+15\,\alpha+4\,\alpha^2\right)\right)-s^3 \left(24\,t22^4\,\alpha^2+t22^2 \left(60+69\,\alpha-32\,\alpha^2\right)+3\,t22^3 \left(-20-20\,\alpha+\alpha^2\right)+2 \left(-7-8\,\alpha+\alpha^2\right)+2\,t22 \left(17+17\,\alpha+5\,\alpha^2\right)\right)-s \left(8+\alpha+t22^2 \left(18-31\,\alpha\right)\alpha+6\,\alpha^2+30\,t22^3\,\alpha^2+t22 \left(20+29\,\alpha+5\,\alpha^2\right)\right)\right) +\\
& \quad t22 \left(-4-\alpha-t22^3\,\alpha^2+2\,s^4\,t22^2 \left(-3+t22+2\,t22^2\right)\left(1+\alpha\right)-t22^2\,\alpha \left(1+\alpha\right)+t22 \left(2-\alpha+\alpha^2\right)-s \left(7\,t22^3\,\alpha^2+2\,t22^4\,\alpha^2+7 \left(2+\alpha\right)+t22^2 \left(4+9\,\alpha-7\,\alpha^2\right)+t22 \left(-12-7\,\alpha+\alpha^2\right)\right)-s^3 \left(-10\,t22^3 \left(1+\alpha\right)+2 \left(2+\alpha\right)+t22^2 \left(8+10\,\alpha-5\,\alpha^2\right)+t22 \left(2+3\,\alpha+2\,\alpha^2\right)+t22^4 \left(-2-2\,\alpha+4\,\alpha^2\right)\right)-s^2 \left(6\,t22^3\,\alpha^2+6\,t22^4\,\alpha^2+6 \left(2+\alpha\right)+t22\,\alpha \left(3+4\,\alpha\right)-t22^2 \left(6+13\,\alpha^2\right)\right)\right) +\\
& \quad t12^3 \left(-6\,s^4 \left(1-2\,t22-3\,t22^2+4\,t22^3\right)\left(1+\alpha\right)+\alpha \left(1+\left(2-3\,t22\right)\alpha\right)+s \left(4+\left(3+6\,t22\right)\alpha+\left(8-13\,t22+2\,t22^2\right)\alpha^2\right)+s^2 \left(-21\,t22^2\,\alpha^2+24\,t22^3\,\alpha^2+\alpha \left(-3+10\,\alpha\right)+t22 \left(12+21\,\alpha-13\,\alpha^2\right)\right)+s^3 \left(-10-11\,\alpha+4\,\alpha^2+24\,t22^3\,\alpha^2+t22 \left(32+35\,\alpha-3\,\alpha^2\right)-t22^2 \left(12+12\,\alpha+23\,\alpha^2\right)\right)\right) +\\
& \quad t12 \Big(4+\alpha+t22^2 \left(9-2\,\alpha\right)\alpha+5\,t22^3\,\alpha^2-2\,s^4 \left(1+2\,t22-12\,t22^2-6\,t22^3+12\,t22^4+3\,t22^5\right)\left(1+\alpha\right)+t22 \left(2+\alpha\right)^2+s \left(2-3\,\alpha+20\,t22^4\,\alpha^2+3\,t22^3\,\alpha \left(2+\alpha\right)+t22^2 \left(-4+23\,\alpha-18\,\alpha^2\right)+t22 \left(32+22\,\alpha+9\,\alpha^2\right)\right)+s^3 \left(-6-7\,\alpha+6\,t22^5\,\alpha^2+t22^2 \left(64+73\,\alpha-3\,\alpha^2\right)+5\,t22 \left(2+\alpha+\alpha^2\right)-3\,t22^3 \left(8+7\,\alpha+7\,\alpha^2\right)+t22^4 \left(-30-30\,\alpha+19\,\alpha^2\right)\right)+s^2 \left(-6-9\,\alpha+39\,t22^4\,\alpha^2+6\,t22^5\,\alpha^2+t22^2 \left(18+45\,\alpha-19\,\alpha^2\right)+t22 \left(48+33\,\alpha+13\,\alpha^2\right)-t22^3 \left(24+15\,\alpha+23\,\alpha^2\right)\right)\Big)\Big) + m \left(t12-t22\right)\\
& \Big(2\,s\,t12^4 \left(-1+t22\right) t22 \left(-1+s \left(-2+3\,t22\right)\right) \left(-\alpha^2-s\,\alpha^2+s^2 \left(1+\alpha\right)\right) +\\
& \quad \left(1+s\right) \left(-1+t22\right) t22^2 \left(2\,s^3\,t22^2 \left(1+\alpha\right)-\alpha \left(1+t22\,\alpha\right)-2\,s \left(-2+t22-\alpha+t22\,\alpha+t22^2\,\alpha^2\right)+s^2 \left(2+\alpha-2\,t22^2\,\alpha^2+t22 \left(2+2\,\alpha+\alpha^2\right)\right)\right) +
\end{aligned}
$$

```
        t12 t22 (2 + (-2 + 3 t22 + 2 t22^2) α + t22 (-2 + t22 + 2 t22^2) α^2 - 4 s^4
            (1 + t22)^2 (1 - 3 t22 + 2 t22^2) (1 + α) + s (6 - 2 α + 10 t22^3 α^2 +
              4 t22^4 α^2 + t22^2 (-4 + 6 α - 13 α^2) + t22 (4 + 5 α + 2 α^2)) + s^3
            (-2 (5 + 6 α) + t22^2 (16 + 20 α - 7 α^2) - 2 t22^3 (13 + 13 α + 2 α^2) + t22
                (26 + 25 α + 4 α^2) + t22^4 (-4 - 4 α + 8 α^2)) + s^2 (12 t22^4 α^2 - 6 (1 + 2 α) +
              t22^3 (-6 - 6 α + 4 α^2) - 3 t22^2 (8 + 4 α + 7 α^2) + t22 (42 + 39 α + 8 α^2))) +
        t12^3 (-12 s^4 t22 (1 - 2 t22 + t22^3) (1 + α) + (2 + α) (1 + t22 α) + s^2
            t22 (6 + t22 (9 - 28 α) α + 16 α^2 + 3 t22^2 α^2 + 12 t22^3 α^2) + s^3 t22 (12 t22^3 α^2 +
              t22 (40 + 43 α - 12 α^2) + 2 (-7 - 8 α + 4 α^2) - t22^2 (24 + 24 α + 7 α^2)) + s
            (2 + α + 2 t22^2 (3 - 8 α) α + 10 t22^3 α^2 + t22 (4 + 2 α + 9 α^2))) +
        t12^2 (-2 - α + 3 t22^2 (-2 + α) α - 4 t22^3 α^2 + 6 s^4 t22 (2 - 3 t22 - 3 t22^2 + 3 t22^3 +
              t22^4) (1 + α) + t22 (-2 + α - α^2) + s^3 t22 (22 + 26 α - 4 α^2 - 6 t22^4 α^2 +
              t22^3 (30 + 30 α - 11 α^2) - t22 (62 + 65 α + 2 α^2) + 3 t22^2 (2 + α + 7 α^2)) - s
            (2 + α - 6 t22^3 (-1 + α) α + 16 t22^4 α^2 + t22^2 (-2 + 10 α - 11 α^2) +
              t22 (12 + α + 7 α^2)) + s^2 t22 (2 (6 - 5 α) α - 27 t22^3 α^2 -
              6 t22^4 α^2 + t22 (-42 - 51 α + 6 α^2) + t22^2 (30 + 21 α + 31 α^2))))) /
    ((1 + s) (-1 + m (t12 - t22) + t22) (m (t12 - t22) + t22) ((-1 + m) t12 - m t22)
      (1 + (-1 + m) t12 - m t22)
      ((-1 + m) m s (2 + s) t12^2 +
        t12 (-1 - 2 s t22 - s^2 t22 - 2 m^2 s (2 + s) t22 + m (2 + 2 s + s^2 + 4 s t22 + 2 s^2 t22)) +
        t22 ((1 + s)^2 + m^2 s (2 + s) t22 - m (2 + 2 s (1 + t22) + s^2 (1 + t22)))) α (2 +
        α)))};
```

We know from 2.3.1 that deltagensym[[3]] is linear in r. The following gives the constant term :

```
factdelta3r0 = Factor[Coefficient[deltagensym[[3]], r, 0]]
```

```
-((4 dd1 m - 4 dd2 m + 4 dd1 s + 20 dd1 m s - 20 dd2 m s + 20 dd1 s^2 + 40 dd1 m s^2 -
     40 dd2 m s^2 + <<37075>> + 48 dd1 m^5 s^3 t12 t22^5 α^4 - 48 dd1 m^6 s^3 t12 t22^5 α^4 +
     24 dd1 m^5 s^4 t12 t22^5 α^4 - 24 dd1 m^6 s^4 t12 t22^5 α^4 + 4 dd1 m^6 s^2 t22^6 α^4 +
     8 dd1 m^6 s^3 t22^6 α^4 + 4 dd1 m^6 s^4 t22^6 α^4) / (4 (1 + s - s t12 + m s t12 - m s t22)^2
     (1 + s - s t12 + m s t12 - m s t22 + t12 α - m t12 α + m t22 α)^2 (1 + s - s t12 + m s t12 -
        m s t22 + α + s α - t12 α + m t12 α - s t12 α + m s t12 α - m t22 α - m s t22 α)^2))

large output | show less | show more | show all | set size limit...
```

```
Denominator[factdelta3r0]
```

```
4 (1 + s - s t12 + m s t12 - m s t22)^2 (1 + s - s t12 + m s t12 - m s t22 + t12 α - m t12 α + m t22 α)^2
  (1 + s - s t12 + m s t12 - m s t22 + α + s α - t12 α + m t12 α - s t12 α + m s t12 α - m t22 α - m s t22 α)^2
```

```
Numerator[factdelta3r0]
```

$-4\,dd1\,m + 4\,dd2\,m - 4\,dd1\,s - 20\,dd1\,m\,s + 20\,dd2\,m\,s - 20\,dd1\,s^2 - 40\,dd1\,m\,s^2 + 40\,dd2\,m\,s^2 - 40\,dd1\,s^3 - 40\,dd1\,m\,s^3 + 40\,dd2\,m\,s^3 - 40\,dd1\,s^4 - 20\,dd1\,m\,s^4 + 20\,dd2\,m\,s^4 - 20\,dd1\,s^5 - 4\,dd1\,m\,s^5 + \cdots 37286 \cdots + 2\,m^6\,p22^2\,s^4\,t22^5\,\alpha^4 - m^5\,p12\,p22^2\,s^4\,t22^5\,\alpha^4 + m^6\,p12\,p22^2\,s^4\,t22^5\,\alpha^4 + m^5\,p22^3\,s^4\,t22^5\,\alpha^4 - m^6\,p22^3\,s^4\,t22^5\,\alpha^4 - 24\,dd1\,m^5\,s^2\,t12\,t22^5\,\alpha^4 + 24\,dd1\,m^6\,s^2\,t12\,t22^5\,\alpha^4 - 48\,dd1\,m^5\,s^3\,t12\,t22^5\,\alpha^4 + 48\,dd1\,m^6\,s^3\,t12\,t22^5\,\alpha^4 - 24\,dd1\,m^5\,s^4\,t12\,t22^5\,\alpha^4 + 24\,dd1\,m^6\,s^4\,t12\,t22^5\,\alpha^4 - 4\,dd1\,m^6\,s^2\,t22^6\,\alpha^4 - 8\,dd1\,m^6\,s^3\,t22^6\,\alpha^4 - 4\,dd1\,m^6\,s^4\,t22^6\,\alpha^4$

large output | show less | show more | show all | set size limit...

Therefore, the constant term in deltagensym[[3]] is given by

```
Factor[factdelta3r0 /. p12p22gent12 /. dd12gent12]
```

0

This gives indeed 0, but takes > 12 CPU hours. An analogous fact holds for the constant term in deltagensym[[6]].

In addition, we find :

```
Factor[
 Numerator[Factor[Coefficient[deltagensym[[3]], r, 1] /. p12p22gent12 /. dd12gent12]] -
  Numerator[Factor[Coefficient[deltagensym[[6]], r, 1] /. p12p22gent12 /. dd12gent12]]]
```

0

Therefore, Coefficient[deltagensym[[3]], r, 1] = 0 is satisfied if and only if Coefficient[deltagensym[[6]], r, 1] = 0. Therefore, the conditions are dependent and the equilibria for r>0 are determined by solving Coefficient[deltagensym[[3]], r, 1] = 0. This implies that t22 is obtained as function of t12. Hence, there is a curve of equilibria!

Summary.

If r=0, then p12p22gent12 and dd12gent12 give the manifold of equilibria, i.e., for every (t12,t22) there is a potential equilibrium (not necessarily admissible).
For given (t12, t22), p12p22gent12 gives the values of p12 and p22, and dd12gent12 gives dd1and dd2.

Because the constant terms in deltagensym[[3]] and deltagensym[[6]] vanish, this also shows that if r>0, then the set of equilibria does not depend on r!
If r>0, it is sufficient to equate the coefficient of r in deltagensym[[3]] to zero to obtain the equilibria!
Because this coefficient is a polynomial of high degree in t12 and t22, an explicit analytical solution is impossible.
However, there exists a curve of equilibria (in each deme), and it is independent of r!

This curve was computed numerically by taking a large number of initial conditions, iterating until equilibrium was reached, and then using curve fitting through these points to display the curve (see Section 3.1.1 for an example).
The endpoints of this curve were computed numerically as outlined in Section 2.1.2 above.

One numerical check:

```
Simplify[deltagensym[[3]] /. p12p22gent12 /. dd12gent12 /. {t12 → 0.3, t22 → 0.64} /.
  {s → 0.2, α → 10, m → 0.01}]
```

$-4.16334\times10^{-17}+0.0205532\,r$

2.4 One isolated population: Derivation of curve of equilibria

This is analogous to Kirkpatrick' s (1982) model, who assumed that the trait is expressed only in males. It is also a psecial case of the continent-island model with m=0 (Servedio and Bürger 2015).

2.4.1 Basics and import definitions

It is sufficient to focus on p2 = p22, t2 = t22, and d2 = dd2, and set m1 = m2 = 0 in itgen. In addition, s1 becomes irrelevant.

```
Simplify[
 (itgen[s1, s, α1, α2, 0, 0, r][{p12, t12, d1, p2, t2, d2}] - {p12, t12, d1, p2, t2, d2})[[
  4 ;; 6]]]
```

```
delitgenNoMig =
  {(d2 (p2 α2 + 2 s^3 t2^2 (1 + α2) + α1 (-1 + p2 + p2 α2 - t2 α2) + s (2 + d2 α2 + p2 α2 + 2 t2 α2 +
            p2 t2 α2 + α1 (1 - 3 t2 - 2 t2^2 α2 + d2 (1 + α2) + p2 (1 + t2) (1 + α2))) +
        s^2 (-2 t2^2 (α1 - α2 + α1 α2) + d2 (α1 + α2 + α1 α2) +
            t2 (4 + (2 + p2) α2 + (1 + p2) α1 (1 + α2))))) /
    (2 (1 + s t2) (1 + s t2 + α1 - t2 α1) (1 + t2 α2 + s t2 (1 + α2))),
  1/2 t2 (-2 + ((1 + s) (2 + p2 α2 + 2 t2 α2 - p2 t2 α2 + 2 s^2 t2^2 (1 + α2) - (-1 + t2) α1
            (1 + p2 + p2 α2 + t2 α2) + s (d2 (α1 + α2 + α1 α2) + t2 (4 + (2 - d2 + p2) α2 -
                  (-1 + d2 - p2) α1 (1 + α2)) - t2^2 ((-2 + p2) α2 + (1 + p2) α1 (1 + α2))))) /
      ((1 + s t2) (1 + s t2 + α1 - t2 α1) (1 + t2 α2 + s t2 (1 + α2)))),
  (-4 d2 (1 + s t2)^2 (1 + s t2 + α1 - t2 α1)^2 (1 + t2 α2 + s t2 (1 + α2))^2 +
     (1 + s) (d2^2 (r + s - s t2 + r s t2) (α1 + α2 + α1 α2) +
        p2 t2 (2 + p2 α2 + r α2 - p2 r α2 + 2 t2 α2 - p2 t2 α2 - r t2 α2 + p2 r t2 α2 +
            2 s^2 t2^2 (1 + α2) - (-1 + t2) α1 (1 + r + r α2 + t2 α2 - p2 (-1 + r) (1 + α2)) +
            s t2 (4 + (2 + r + p2 (-1 + r) (-1 + t2) + 2 t2 - r t2) α2 + (-1 + p2 (-1 + r) - r)
                (-1 + t2) α1 (1 + α2))) + d2 (2 + α1 + p2 α1 - t2 α1 - p2 t2 α1 + p2 α2 +
            2 t2 α2 - p2 t2 α2 + p2 α1 α2 + t2 α1 α2 - p2 t2 α1 α2 - t2^2 α1 α2 + 2 s^2 t2^2 (1 + α2) +
            s t2 (4 + 2 (1 + p2 + t2 - p2 t2) α2 - (1 + 2 p2) (-1 + t2) α1 (1 + α2)) +
            r (-2 - p2 α2 - 2 t2 α2 + 2 p2 t2 α2 + α1 (-1 - t2 α2 + p2 (-1 + 2 t2) (1 + α2)) + s
                t2 (-2 - α2 - t2 (α1 + α2 + α1 α2) + p2 (-1 + 2 t2) (α1 + α2 + α1 α2)))))
      (d2^2 (1 + s) (r - s t2 + r s t2) (α1 + α2 + α1 α2) + (-1 + p2) (-1 + t2)
         (2 + 2 α1 - t2 α1 - p2 t2 α1 + p2 r t2 α1 + 2 t2 α2 - p2 t2 α2 + p2 r t2 α2 +
            2 t2 α1 α2 - p2 t2 α1 α2 + p2 r t2 α1 α2 - t2^2 α1 α2 + s^2 t2^2 (2 + (2 + p2 (-1 + r))
                α2 + (1 + p2 (-1 + r)) α1 (1 + α2)) + s t2 (4 + (2 + p2 (-1 + r))
                (1 + t2) α2 + α1 (3 - t2 + 2 α2 + p2 (-1 + r) (1 + t2) (1 + α2)))) +
        d2 (2 + 2 α1 - t2 α1 - p2 t2 α1 + 2 t2 α2 - p2 t2 α2 + 2 t2 α1 α2 - p2 t2 α1 α2 -
```

```
t2^2 α1 α2 + s t2 (4 + α2 + 3 t2 α2 - 2 p2 t2 α2 + α1
      (2 + α2 + t2 α2 - 2 p2 t2 (1 + α2))) + s^2 t2 ((-1 + p2) (α1 + α2 + α1 α2) +
    t2 (2 + 3 α2 - 2 p2 α2 - 2 (-1 + p2) α1 (1 + α2))) + r (1 + s)
   (-2 - p2 α2 - 2 t2 α2 + 2 p2 t2 α2 + α1 (-1 - t2 α2 + p2 (-1 + 2 t2) (1 + α2)) +
    s t2 (-2 - α2 - t2 (α1 + α2 + α1 α2) + p2 (-1 + 2 t2) (α1 + α2 + α1 α2))))) -
(1 + s) (d2^2 (r + s - s t2 + r s t2) (α1 + α2 + α1 α2) + (-1 + p2) t2
   (2 + p2 α2 - p2 r α2 + 2 t2 α2 - p2 t2 α2 + p2 r t2 α2 + 2 s^2 t2^2 (1 + α2) -
    (-1 + t2) α1 (1 + t2 α2 - p2 (-1 + r) (1 + α2)) + s t2 (4 + p2 (-1 + r) (-1 + t2)
      α2 + 2 (1 + t2) α2 + (-1 + p2 (-1 + r)) (-1 + t2) α1 (1 + α2))) +
  d2 (2 + α1 + p2 α1 - t2 α1 - p2 t2 α1 + p2 α2 + 2 t2 α2 - p2 t2 α2 + p2 α1 α2 +
    t2 α1 α2 - p2 t2 α1 α2 - t2^2 α1 α2 + 2 s^2 t2^2 (1 + α2) +
    s t2 (4 + α2 + 3 t2 α2 - 2 p2 (-1 + t2) (α1 + α2 + α1 α2)) +
    r (-2 - p2 α2 - 2 t2 α2 + 2 p2 t2 α2 + α1 (-1 - t2 α2 + p2 (-1 + 2 t2) (1 + α2)) + s
      t2 (-2 - α2 - t2 (α1 + α2 + α1 α2) + p2 (-1 + 2 t2) (α1 + α2 + α1 α2)))))
 (d2^2 (1 + s) (r - s t2 + r s t2) (α1 + α2 + α1 α2) + p2 (-1 + t2)
   (2 + 2 α1 - t2 α1 - p2 t2 α1 - r t2 α1 + p2 r t2 α1 + 2 t2 α2 - p2 t2 α2 - r t2 α2 +
    p2 r t2 α2 + 2 t2 α1 α2 - p2 t2 α1 α2 - r t2 α1 α2 + p2 r t2 α1 α2 - t2^2 α1 α2 +
    s^2 t2^2 (2 + (2 + p2 (-1 + r) - r) α2 + (-1 + p2) (-1 + r) α1 (1 + α2)) +
    s t2 (4 + (2 + p2 (-1 + r) - r) (1 + t2) α2 + α1
      (3 - t2 + 2 α2 + p2 (-1 + r) (1 + t2) (1 + α2) - r (1 + t2) (1 + α2)))) +
  d2 (2 + 2 α1 - t2 α1 - p2 t2 α1 + 2 t2 α2 - p2 t2 α2 + 2 t2 α1 α2 - p2 t2 α1 α2 -
    t2^2 α1 α2 + s t2 (4 + 2 (1 + t2 - p2 t2) α2 - α1 (-3 + t2 - 2 α2 + 2 p2 t2 (1 + α2))) +
    s^2 t2 (p2 (α1 + α2 + α1 α2) + t2 (2 + 2 α2 - 2 p2 α2 - (-1 + 2 p2) α1 (1 + α2))) +
    r (1 + s) (-2 - p2 α2 - 2 t2 α2 + 2 p2 t2 α2 + α1
      (-1 - t2 α2 + p2 (-1 + 2 t2) (1 + α2)) + s t2
      (-2 - α2 - t2 (α1 + α2 + α1 α2) + p2 (-1 + 2 t2) (α1 + α2 + α1 α2)))))) /
(4 (1 + s t2)^2 (1 + s t2 + α1 - t2 α1)^2 (1 + t2 α2 + s t2 (1 + α2))^2)};
```

As in 2.1.1, the edges t2 = 0 and t2 = 1 consist of equilibria.

2.4.2 Stability of edge equilibria

Jacobian at t2 = 0:

```
Simplify[D[delitgenNoMig, {{p2, t2, d2}}] //. {t2 → 0, d2 → 0}]
```

$$\Big\{\Big\{0,\ 0,\ \frac{1}{2\,(1+\alpha1)}\big(p2\,\alpha2+\alpha1\,(-1+p2+p2\,\alpha2)+s\,(2+p2\,\alpha2+\alpha1\,(1+p2+p2\,\alpha2))\big)\Big\},$$

$$\Big\{0,\ -1+\frac{(1+s)\,(2+p2\,\alpha2+\alpha1\,(1+p2+p2\,\alpha2))}{2\,(1+\alpha1)},\ 0\Big\},$$

$$\Big\{0,\ -\frac{(-1+p2)\,p2\,r\,(1+s)\,(\alpha1+\alpha2+\alpha1\,\alpha2)}{2\,(1+\alpha1)},\ \frac{1}{2\,(1+\alpha1)}\big(-\alpha1+p2\,\alpha1+p2\,\alpha2+p2\,\alpha1\,\alpha2+ s\,(2+\alpha1+p2\,\alpha1+p2\,\alpha2+p2\,\alpha1\,\alpha2)-r\,(1+s)\,(2+p2\,\alpha2+\alpha1\,(1+p2+p2\,\alpha2))\big)\Big\}\Big\}$$

Eigenvalues:

```
eigenvt20 = Simplify[Eigenvalues[%]]
```

$$\left\{0, \frac{1}{2\,(1+\alpha 1)}\left(p2\,\alpha 2+\alpha 1\,(-1+p2+p2\,\alpha 2)+s\,(2+p2\,\alpha 2+\alpha 1\,(1+p2+p2\,\alpha 2))\right),\right.$$
$$\left.\frac{1}{2\,(1+\alpha 1)}\left(-\alpha 1+p2\,\alpha 1+p2\,\alpha 2+p2\,\alpha 1\,\alpha 2+s\,(2+\alpha 1+p2\,\alpha 1+p2\,\alpha 2+p2\,\alpha 1\,\alpha 2)-r\,(1+s)\,(2+p2\,\alpha 2+\alpha 1\,(1+p2+p2\,\alpha 2))\right)\right\}$$

```
Simplify[eigenvt20[[3]] - eigenvt20[[2]]]
```

$$-\frac{r\,(1+s)\,(2+p2\,\alpha 2+\alpha 1\,(1+p2+p2\,\alpha 2))}{2\,(1+\alpha 1)}$$

This is always negative! Thus, the second ev is always larger than the third! (This could be also inferred from the analysis of the characteristic polynomials cpolgenT20noR and cpolgenT20R in Section 2.1.2)
Hence, stability is determined by the second eigenvalue.

```
Simplify[Series[Numerator[eigenvt20[[2]]], {p2, 0, 2}]] // Normal
```

$$-\alpha 1+s\,(2+\alpha 1)+p2\,(1+s)\,(\alpha 1+\alpha 2+\alpha 1\,\alpha 2)$$

This is strictly monotone increasing in p2. Therefore, stability of equilibria on this edge changes at:

```
Simplify[Solve[-α1 + s (2 + α1) + p2 (1 + s) (α1 + α2 + α1 α2) == 0, p2]]
```

$$\left\{\left\{p2\to\frac{\alpha 1-s\,(2+\alpha 1)}{(1+s)\,(\alpha 1+\alpha 2+\alpha 1\,\alpha 2)}\right\}\right\}$$

Analogously, one obtains the value of p2 at t2 = 1, at which stability of equilibria changes (i.e., for smaller p2, equilibria are stable, for larger they are unstable).

Important points on the edges t2 = 0, t2 = 1 :

```
p2t0ast = (α1 - s (2 + α1)) / ((1 + s) (α1 + α2 + α1 α2));
p2t1ast = ((α1 - 2 s) (1 + α2)) / (α1 + α2 + α1 α2);
```

We observe that :

$0 < p2t0ast < 1$ if and only if $\frac{\alpha 1}{2+\alpha 1} > s$;

$0 < p2t1ast < 1$ if and only if $\alpha 1 > 2\,s$.

Simple considerations show the following

Edge t2 = 0:

(a) If $\frac{\alpha 1}{2+\alpha 1} < s$, then p2t0ast ≤ 0 and all equilibria are unstable.

The condition $\frac{\alpha 1}{2+\alpha 1} < s$ is equivalent to $s \geq 1$ or $\left(s < 1 \text{ and } \alpha 1 < \frac{2\,s}{1-s}\right)$.

(b) If $\frac{\alpha 1}{2+\alpha 1} > s$, then 0 < p2t0ast < 1 and equilibria with p2 < p2t0ast are stable, and equilibria with p2 > p2t0ast are unstable.

The condition $\frac{\alpha 1}{2+\alpha 1} > s$ is equivalent to $s < 1$ and $\alpha 1 > \frac{2 s}{1-s}$.

Edge t2 = 1:

(a) If $\frac{\alpha 1}{2} \leq s$, then p2t1ast ≤ 0 and all equilibria are stable.

(b) If $\frac{\alpha 1}{2} > s$, then 0 < p2t1ast < 1 and equilibria p2 < p2t1ast are unstable and equilibria p2 > p2t1ast are stable.

If $\frac{\alpha 1}{2+\alpha 1} < s < \frac{\alpha 1}{2}$, the equilibrium p2=0 and t2 = $\frac{\alpha 1 - s\,(2+\alpha 1)}{2\,s\,(s-\alpha 1)}$ exists. It is the endpoint of a curve and stable.

2.4.3 Determining the curve of polymorphic equilibria

The following shows that the equilibrium conditions for p2 and t2 are proportional (hence, there will be a curve of polymorphic equilibria):

```
Simplify[delitgenNoMig[[1]] / delitgenNoMig[[2]]]
```

$$\frac{d2}{t2 - t2^2}$$

```
Numerator[Factor[delitgenNoMig[[2]]]]
```

Compute d2 as a function of p2 and t2 delitgenNoMig[[2]] :

```
FullSimplify[Solve[delitgenNoMig[[2]] == 0, d2]]
```

Substitute this into delitgenNoMig[[3]] to compute p2

```
Simplify[Numerator[Factor[
   delitgenNoMig[[3]] /. d2 → - (((1 + s t2) ((-1 + p2) α1 + s (2 + 2 s t2 + (1 + p2 - 2 t2) α1)) +
            (1 + s) (-t2 α1 + p2 (1 + s t2) (1 + α1) + s t2 (2 + 2 s t2 + α1 - 2 t2 α1)) α2) / (s
            (1 + s) (α1 + α2 + α1 α2)))]]]
```

The above is quadratic in p2 (and r is just a factor). Solve for p2 :

```
Simplify[Solve[% == 0, p2], Assumptions → {s > 0, t2 ≥ 0, α1 > 0, α2 > 0, r > 0}]
```

A straightforward analysis shows that only the second solution is admissible.

Therefore, we obtain the following curve of equilibria as a function of t2 (and the other parameters):

```
p2curve[t2_, s_, α1_, α2_] := - 1/((1 + s)^2 (1 + s t2) (α1 + α2 + α1 α2))
  (3 s + 2 s^2 + 8 s^2 t2 + 5 s^3 t2 + 7 s^3 t2^2 + 4 s^4 t2^2 + 2 s^4 t2^3 + s^5 t2^3 - α1 + s α1 + s^2 α1 -
    4 s t2 α1 + s^2 t2 α1 + 2 s^3 t2 α1 - 5 s^2 t2^2 α1 - s^3 t2^2 α1 + s^4 t2^2 α1 - 2 s^3 t2^3 α1 -
    s^4 t2^3 α1 + 3 s t2 α2 + 5 s^2 t2 α2 + 2 s^3 t2 α2 + 5 s^2 t2^2 α2 + 8 s^3 t2^2 α2 + 3 s^4 t2^2 α2 +
    2 s^3 t2^3 α2 + 3 s^4 t2^3 α2 + s^5 t2^3 α2 - t2 α1 α2 + 2 s^2 t2 α1 α2 + s^3 t2 α1 α2 - 3 s t2^2 α1 α2 -
    3 s^2 t2^2 α1 α2 + s^3 t2^2 α1 α2 + s^4 t2^2 α1 α2 - 2 s^2 t2^3 α1 α2 - 3 s^3 t2^3 α1 α2 -
    s^4 t2^3 α1 α2 - s √((1 + s t2) (1 + s t2 + α1 - t2 α1) (1 + t2 α2 + s t2 (1 + α2))
       (1 + α1 - t2 α1 + t2 α2 + s^5 t2^3 (1 + α2) + s t2 (5 - 2 (-1 + t2) α1 + (3 + 2 t2) α2) +
         s^4 t2^2 (5 + (4 + t2) α2 - (-1 + t2) α1 (1 + α2)) +
         s^3 t2 (-t2^2 α1 α2 + (2 + α1) (2 + α2) + t2 (6 - 2 α1 + 8 α2)) +
         s^2 t2 (6 + 4 α2 + α1 (2 + α2) + t2 (4 + 6 α2 - α1 (2 + α2))))));
d2curve[t2_, s_, α1_, α2_] := 1/((1 + s)^2 (α1 + α2 + α1 α2))
  (1 + 4 s t2 + s^2 t2 + 5 s^2 t2^2 + 2 s^3 t2^2 + 2 s^3 t2^3 + s^4 t2^3 + α1 - t2 α1 + 3 s t2 α1 + s^2 t2 α1 -
    3 s t2^2 α1 + s^2 t2^2 α1 + s^3 t2^2 α1 - 2 s^2 t2^3 α1 - s^3 t2^3 α1 + t2 α2 + s t2 α2 + 3 s t2^2 α2 +
    4 s^2 t2^2 α2 + s^3 t2^2 α2 + 2 s^2 t2^3 α2 + 3 s^3 t2^3 α2 + s^4 t2^3 α2 + t2 α1 α2 + s t2 α1 α2 -
    t2^2 α1 α2 + s t2^2 α1 α2 + 3 s^2 t2^2 α1 α2 + s^3 t2^2 α1 α2 - 2 s t2^3 α1 α2 - 3 s^2 t2^3 α1 α2 -
    s^3 t2^3 α1 α2 - √((1 + s t2) (1 + s t2 + α1 - t2 α1) (1 + t2 α2 + s t2 (1 + α2))
      (1 + α1 - t2 α1 + t2 α2 + s^5 t2^3 (1 + α2) + s t2 (5 - 2 (-1 + t2) α1 + (3 + 2 t2) α2) +
        s^4 t2^2 (5 + (4 + t2) α2 - (-1 + t2) α1 (1 + α2)) +
        s^3 t2 (-t2^2 α1 α2 + (2 + α1) (2 + α2) + t2 (6 - 2 α1 + 8 α2)) +
        s^2 t2 (6 + 4 α2 + α1 (2 + α2) + t2 (4 + 6 α2 - α1 (2 + α2))))));
```

It indeed connects (p2t0ast, 0, 0) with (p2t1ast, 1, 0).

2.4.4 Graphs

```
ParametricPlot3D[{t2, p2curve[t2, 0.2, 10, 10], d2curve[t2, 0.2, 10, 10]}, {t2, 0, 1},
 PlotRange → {{0, 1}, {0, 1}, {0, 0.15}}, BoxRatios → 1, AxesLabel → {t2, p2, D}]
```

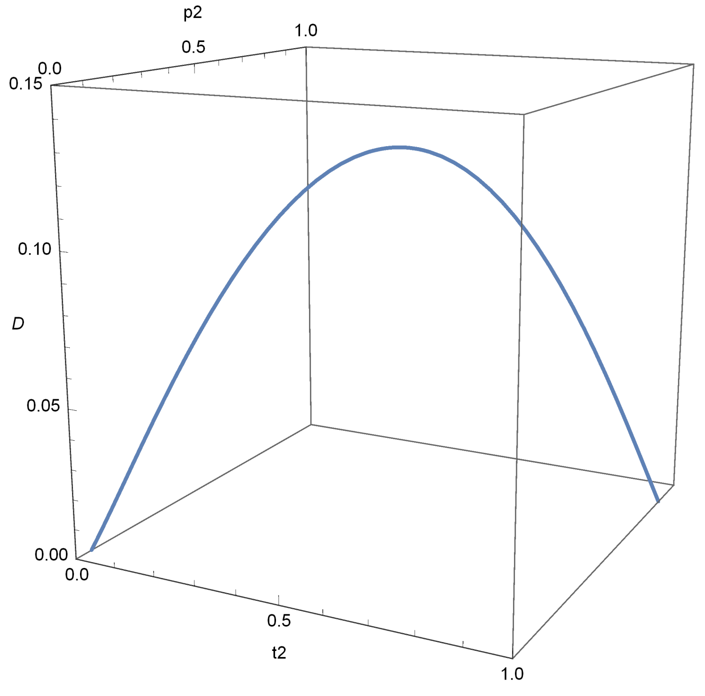

2.4.5. Properties of the curve (summary)

If $s < \frac{\alpha 1}{2}$, the curve exists and connects equilibria on the boundaryas follows :

1. If $\frac{\alpha 1}{2 + \alpha 1} < s \left(\text{or, equivalently, } \alpha 1 > \frac{2\,s}{1 - s} \text{ and } s < 1\right)$,

then it connects (p2, t2, d2) = (p2t0ast, 0, 0) with (p2t1ast, 1, 0).

2. If $\frac{\alpha 1}{2 + \alpha 1} < s < \frac{\alpha 1}{2}$,

the it connects (p2, t2, d2) = $\left(0, \frac{\alpha 1 - s\,(2 + \alpha 1)}{2\,s\,(s - \alpha 1)}, 0\right)$ with (p2t1ast, 1, 0).

Note that case 2 does not exist if selection acts only on males.

If $s > \frac{\alpha 1}{2}$, then the edge t2 = 1 is stable, i.e.,

trajectories from the interior converge to some point on this edge (or value p2).

It can also be shown that the two nonzero eigenvalues at the curve equilibria are negative. Hence, the curve is attracting.

3. Numerics

3.1 3D Graphs

3.1.1 Computing the curve of equilibria for r>0

Here is a list of points at the equilibrium curve (in deme 2) generated by forward iteration from many initial conditions and adding the two endpoints of the curve (see Section 2.1.2). The parameters are s1=s2=0.2, $\alpha 1=\alpha 2=10$, m1=m2=0.01.

```
curvetestcor = {{0, 0, 0}, {0.0543`, 0, 0},
   {0.05660184549540463`, 0.003797945690028683 6`, 0.08956592091594318`},
   {0.06293855717352347`, 0.01416124988810209`, 0.16830729752874554`},
   {0.06697210466039187`, 0.020735137757946442`, 0.20034661333909676`},
   {0.07187476326385878`, 0.028698339084111516`, 0.2312458954044843`},
   {0.07544512372411044`, 0.034477312711836634`, 0.2501032537113619`},
   {0.07717254954432423`, 0.0372669013298359 7`, 0.25839209179040895`},
   {0.08083017489183085`, 0.043159256993953836`, 0.27447405890270077`},
   {0.08633379352554969`, 0.05198773295467537`, 0.2956236194244254`},
   {0.08761521824010333`, 0.054036622403928584`, 0.300111656227197`},
   {0.08973001650999651`, 0.05741239751113015`, 0.3072066941250179`},
   {0.09814867464065638`, 0.07078034924694529`, 0.3321888747519035`},
   {0.10864419728816559`, 0.08728515952065873`, 0.3577159585557959`},
   {0.10870477365339459`, 0.08737989463453665`, 0.3578488302527233`},
   {0.11127287951672861`, 0.09139058129873132`, 0.3633491761294169`},
```

```
{0.11812426799323449`, 0.10203735791085268`, 0.37686452761090644`},
{0.11901022085398592`, 0.10340843611575098`, 0.37850044234757785`},
{0.1209895230416802`, 0.1064668780489906`, 0.38207056269203943`},
{0.12917967091488097`, 0.11905387276980248`, 0.3957093973091313`},
{0.13817711838388322`, 0.132755096551799 5`, 0.4088825748528965`},
{0.14470515205365492`, 0.1426138843371581`, 0.41744699919639994`},
{0.15706596239702128`, 0.16109541730424295`, 0.4317699225248873`},
{0.1896641867757147`, 0.20870873113438498`, 0.4607171431040528`},
{0.20759819201782306`, 0.23424062261727313`, 0.4727135883051294`},
{0.2134783491466003`, 0.242514355197026 3`, 0.4761834705190131`},
{0.21779411537695048`, 0.24855696602625846`, 0.4786008647940324`},
{0.23519344478351012`, 0.27266725572017686`, 0.4873474570893123`},
{0.24511873060981698`, 0.28624545557885095`, 0.4916974110617419`},
{0.2922048377543788`, 0.3490310315493098 7`, 0.5073368199586237`},
{0.3149159753823795`, 0.3784126410529403 7`, 0.512515196855238 8`},
{0.3174900231636321`, 0.3817077853226969`, 0.5130211497579203`},
{0.33129893831153834`, 0.39926742753133965`, 0.5154762025192541`},
{0.3359364468607152`, 0.405120769682420 8`, 0.5162065531815315`},
{0.34534059458250055`, 0.41692429037741024`, 0.5175494289931301`},
{0.3667408164820048`, 0.4434631850094184`, 0.519954015721805`},
{0.3784466413933181`, 0.45779694161704004`, 0.5209094747718551`},
{0.38287673965708674`, 0.4631887979796312`, 0.5212078563674708`},
{0.4076699312356923`, 0.49304216287247343`, 0.5222645091159591`},
{0.40888996243457`, 0.4944973931147247`, 0.52229039235307`},
{0.40915658900192814`, 0.49481525118937486`, 0.5222957297700026`},
{0.4339569087470273`, 0.524120730712402 3`, 0.5223001359510501`},
{0.4446090855901148`, 0.5365539498479851`, 0.5220086853028184`},
{0.4699251377135113`, 0.5657472827711086`, 0.5206226004516094`},
{0.47848775667480375`, 0.5755115234140892`, 0.5199338701986029`},
{0.47851970199604676`, 0.57554785025021`, 0.5199310922645882`},
{0.4792329740687634`, 0.5763587571519981`, 0.5198686627458622`},
{0.48643317738660835`, 0.5845237330803713`, 0.5191950770289251`},
{0.4949159898914635`, 0.5940951213831318`, 0.5182997888217864`},
{0.5159926309422291`, 0.6176568952074447`, 0.515592548504511`},
{0.5249854564755814`, 0.627617154798091 3`, 0.5142237933026124`},
{0.5371679561106322`, 0.6410239976729145`, 0.512160036073494`},
{0.5404446474354232`, 0.6446133260559913`, 0.5115630154593267`},
{0.5413684583424265`, 0.6456240160053848`, 0.5113914369495139`},
{0.5420332714962874`, 0.6463510079923273`, 0.5112670711865185`},
{0.5439349817158053`, 0.6484290003160936`, 0.5109071912816989`},
{0.57178403642519 4`, 0.6785959990488386`, 0.5049139310684305`},
{0.5742766392404987`, 0.6812724667109248`, 0.5043091051332322`},
{0.576837201336487`, 0.6840179458670907`, 0.5036756170201854`},
{0.5894437181868252`, 0.6974769183145952`, 0.5003725862976591`},
{0.5946632739946408`, 0.7030215332474032`, 0.49891281837638485`},
{0.5979969238483621`, 0.7065543430834157`, 0.4979513536919065`},
{0.60491162289776`, 0.7138613077245021`, 0.4958829286847526`},
{0.6074027448992102`, 0.7164869042863247`, 0.4951127345087458`},
{0.6295483657040672`, 0.73967095981419 2`, 0.48765154270459543`},
{0.6382138932160628`, 0.7486670930088165`, 0.4844128844618195`},
{0.6415082123927798`, 0.7520760713559929`, 0.4831314693576045`},
{0.6433063614338183`, 0.7539342551416135`, 0.4824200421399131`},
{0.6474924239835468`, 0.7582531226112944`, 0.48073042924068976`},
{0.6517098416131714`, 0.7625945304106434`, 0.47897986266862896`},
{0.656967634858931`, 0.7679316349155 29`, 0.4767275694047644`},
```

```
{0.6635058538773997`, 0.7746853362417472`, 0.47381473882984537`},
{0.673442257398772`, 0.7848109264476019`, 0.4691382168575423`},
{0.6864834319878824`, 0.7980186716833192`, 0.46251015574925536`},
{0.6874402827624676`, 0.7989840923306586`, 0.46200061403011555`},
{0.6882878535959853`, 0.7998388369551203`, 0.46154651291756243`},
{0.6890406903600791`, 0.800597716812932`, 0.4611409840507769`},
{0.6922989018062435`, 0.8038785126162025`, 0.4593619090714377`},
{0.6961184813158338`, 0.807717173664323 5`, 0.4572256728998312`},
{0.6969205193530782`, 0.8085222030191773`, 0.4567700152578396`},
{0.698793776837788`, 0.8104010720140574`, 0.4556960186491584`},
{0.7203909710548612`, 0.8319225862253015`, 0.44225752147076`},
{0.7317880631593816`, 0.8431734688989039`, 0.4342985217686242`},
{0.7334223430227556`, 0.8447805921595001`, 0.43310278562944093`},
{0.7382808652967848`, 0.8495490260243432`, 0.4294626610617528`},
{0.7384769545256583`, 0.849741182846746 8`, 0.4293130062291318`},
{0.7416093874705 2`, 0.8528076438223302`, 0.42689273729456756`},
{0.7619357973570297`, 0.8725558572268646`, 0.40971888713657334`},
{0.7695145612414444`, 0.8798480733153262`, 0.4025889141564653`},
{0.7723627821992717`, 0.8825778492241741`, 0.39979540339410563`},
{0.7759494703646802`, 0.886006663591593`, 0.39618424353839315`},
{0.7768751778771739`, 0.8868900082206609`, 0.39523483717156965`},
{0.7810743847121827`, 0.8908884646848251`, 0.3908354763542134`},
{0.7984358528426314`, 0.9072564679952063`, 0.3708803298969047`},
{0.8015210591546166`, 0.9101343693730436`, 0.36700490240665773`},
{0.8108252528857616`, 0.9187478521743578`, 0.3546357642396313`},
{0.811331533473403`, 0.919213503425836 7`, 0.35393164651384595`},
{0.8143056601135491`, 0.9219420875253119`, 0.34972690940189294`},
{0.8165385396788698`, 0.923982611809436`, 0.3464913131256027`},
{0.8263023333386098`, 0.9328156197867329`, 0.33149425270135274`},
{0.8418273198029743`, 0.9464871298307743`, 0.3044335414653078`},
{0.844379779046111`, 0.9486796476216544`, 0.2995595442428612`},
{0.8482777314932839`, 0.951991435568306`, 0.2918637209630855`},
{0.8519604417927931`, 0.9550757621266964`, 0.28430192523648307`},
{0.8532439822946108`, 0.9561396687169569`, 0.2815979587194742`},
{0.8614468864597258`, 0.9627848979299587`, 0.26344929769419717`},
{0.8761036718543275`, 0.9738113661703788`, 0.2271314418759672`},
{0.8769685155710457`, 0.9744195610745443`, 0.2248323831818406`},
{0.877275490470237`, 0.9746341306033633`, 0.2240122861274224`},
{0.8855047036089843`, 0.9801096353904881`, 0.20127326166811996`},
{0.8909469842157165`, 0.9834051663463637`, 0.18551729997724517`},
{0.9031038766252986`, 0.9896823612150077`, 0.14889336604266454`},
{0.9076458532821965`, 0.9916160065182898`, 0.13494426712407512`},
{0.9114143286570854`, 0.9930481602067847`, 0.12335228482418856`},
{0.9280481225312094`, 0.9976275374149793`, 0.07263886559671419`},
{0.934476877938098`, 0.9987362821293031`, 0.052907308944191794`},
{0.945715`, 1, 0}, {1.`, 1.`, 0}};
```

```
f10cor = BSplineFunction[curvetestcor];
plotcurveequilcor10 = ParametricPlot3D[f10cor[x], {x, 0, 1},
  PlotRange → {{0, 1}, {0, 1}, {0, 0.6}}, BoxRatios → {1, 1, 0.6}, PlotStyle → Black]
```

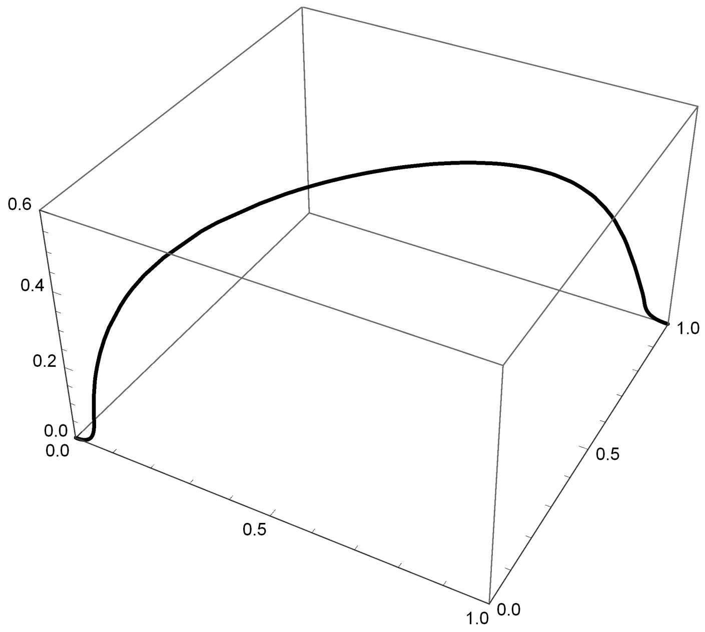

3.1.2 Generating Figure 3b (see main text for details)

```
datsymfix1[p20_, t20_, dd0_, s_, α_, m_, r_, tend_] :=
 datsymfix1[p20, t20, dd0, s, α, m, r, tend] = NestList[itgen[s, s, α, α, m, m, r],
   {0, 0, 0, p2, t2, dd} /. {p2 → p20, t2 → t20, dd → dd0}, tend]
```

```
initphm = {{0.99, 0.99, 0.99 * 0.01}, {0.95, 0.95, 0.05 * 0.95}, {0.9, 0.9, 0.1 * 0.9},
   {0.8, 0.8, 0.2 * 0.8}, {0.7, 0.7, 0.3 * 0.7}, {0.6, 0.6, 0.4 * 0.6}};
```

```
tabdatphmfix1[ss_, αα_, mm_, r_, tend_][nvals_] :=
 Table[{n, Transpose[Table[datsymfix1[initphm[[i, 1]], initphm[[i, 2]],
        initphm[[i, 3]], ss, αα, mm, r, tend][[n]] // Chop, {i, 1, 6}]]}, {n, nvals}]
```

Data for specific generations ({1, 10, 50, 100, 250, 500, 1000}) :

```
Round[10^4 * tabdatphmfix1[0.2, 10, 0.01, 0.1, 2000][
    {1, 10, 50, 100, 250, 500, 1000, 2000}]] / 10^4. // TableForm
```

	0.	0.	0.	0.	0.	0.
	0.	0.	0.	0.	0.	0.
1.	0.	0.	0.	0.	0.	0.
	0.99	0.95	0.9	0.8	0.7	0.6
	0.99	0.95	0.9	0.8	0.7	0.6
	0.0099	0.0475	0.09	0.16	0.21	0.24
	0.0157	0.0156	0.0155	0.015	0.0143	0.0132
	0.0113	0.0112	0.0111	0.0108	0.0103	0.0095
10.	0.0095	0.0094	0.0093	0.009	0.0085	0.0078
	0.9834	0.9797	0.9746	0.961	0.938	0.8983
	0.9885	0.9876	0.9857	0.9777	0.9589	0.922
	0.0096	0.0103	0.0117	0.0177	0.0319	0.0596
	0.0541	0.0538	0.0534	0.0525	0.0515	0.0502
	0.0191	0.019	0.0189	0.0186	0.0182	0.0178
50.	0.0149	0.0148	0.0146	0.0143	0.0139	0.0133
	0.9448	0.9403	0.9346	0.9222	0.9073	0.8877
	0.9806	0.9794	0.9778	0.9738	0.9682	0.9594
	0.0152	0.016	0.0172	0.0199	0.0237	0.0293
	0.117	0.1158	0.1142	0.111	0.1075	0.1035
	0.0425	0.0418	0.0409	0.039	0.0369	0.0344
100.	0.0289	0.0284	0.0277	0.0262	0.0245	0.0225
	0.8809	0.8737	0.8642	0.8427	0.8158	0.7804
	0.9564	0.9525	0.9471	0.9334	0.9139	0.8854
	0.0296	0.0319	0.0351	0.0428	0.0531	0.0669
	0.3275	0.3197	0.3101	0.2913	0.2718	0.251
	0.2164	0.2085	0.1989	0.1801	0.161	0.1408
250.	0.0938	0.0911	0.0878	0.081	0.0737	0.0655
	0.6678	0.6573	0.6443	0.6174	0.588	0.5546
	0.7789	0.7683	0.755	0.7272	0.6963	0.6605
	0.0953	0.0984	0.1021	0.1092	0.1159	0.1221
	0.3694	0.3601	0.3489	0.3267	0.3039	0.2795
	0.2592	0.2496	0.238	0.2153	0.1923	0.168
500.	0.1031	0.1006	0.0973	0.0905	0.0829	0.0742
	0.6258	0.6163	0.6045	0.58	0.5533	0.5226
	0.7358	0.7259	0.7134	0.6875	0.6586	0.625
	0.1044	0.1068	0.1097	0.1151	0.12	0.1245
	0.3702	0.3609	0.3496	0.3273	0.3045	0.28
	0.26	0.2504	0.2387	0.216	0.1929	0.1685
1000.	0.1033	0.1007	0.0975	0.0907	0.0831	0.0744
	0.6251	0.6156	0.6038	0.5794	0.5526	0.522
	0.735	0.7251	0.7127	0.6867	0.6579	0.6243
	0.1045	0.107	0.1098	0.1152	0.1201	0.1245
	0.3702	0.3609	0.3496	0.3273	0.3045	0.28
	0.26	0.2504	0.2387	0.216	0.1929	0.1685
2000.	0.1033	0.1007	0.0975	0.0907	0.0831	0.0744
	0.6251	0.6156	0.6038	0.5794	0.5526	0.522
	0.735	0.7251	0.7127	0.6867	0.6579	0.6243
	0.1045	0.107	0.1098	0.1152	0.1201	0.1245

This figure was produced as follows:

```
plotcor2br01endp =
 ListPointPlot3D[Table[ddat = datsymfix1[initphm[[i, 1]], initphm[[i, 2]],
      initphm[[i, 3]], 0.2, 10, 0.01, 0.1, 2000][[1000, 4 ;; 6]];
   {{ddat[[1]], ddat[[2]], ddat[[3]] / (√(ddat[[1]] (1 - ddat[[1]]) ddat[[2]] (1 -
            ddat[[2]])))}}, {i, 1, 6}], PlotStyle → {Directive[Red, PointSize[0.035]],
    Directive[Darker[Green], PointSize[0.035]], Directive[Magenta, PointSize[0.035]],
    Directive[Orange, PointSize[0.035]], Directive[Green, PointSize[0.035]],
    Directive[Blue, PointSize[0.035]]}, AxesLabel → {p2, t2, D}];
plotcor2br01all = ListPointPlot3D[Table[Table[ddat = datsymfix1[initphm[[i, 1]],
       initphm[[i, 2]], initphm[[i, 3]], 0.2, 10, 0.01, 0.1, 2000][[n, 4 ;; 6]];
    {ddat[[1]], ddat[[2]], ddat[[3]] / (√(ddat[[1]] (1 - ddat[[1]])
          ddat[[2]] (1 - ddat[[2]])))}, {n, 1, 1000}], {i, 1, 6}],
  PlotStyle → {Red, Darker[Green], Magenta, Orange, Green, Blue}];
plotcor2br01 = Show[plotcor2br01endp, plotcor2br01all, plotcurveequilcor10,
  ImageSize → 450, PlotRange → {{0, 1}, {0, 1}, {-0.005, 1.005}},
  AxesLabel → {Text[Style[p22, Black, 15]],
    Text[Style[t22, Black, 15]], Text[Style[D2,cor, Black, 15]]},
  LabelStyle → Directive[Black, FontFamily → "Helvetica", 12], BoxRatios → {1, 1, 0.6},
  ViewPoint → {3.4, -2.4, 1.6}, AxesEdge → {{0, 0}, {1, 0}, {1, 1}},
  Ticks → {{0, 0.5, 1}, {{0, "     0"}, 0.5, 1}, {0, 0.5, 1}}]
```

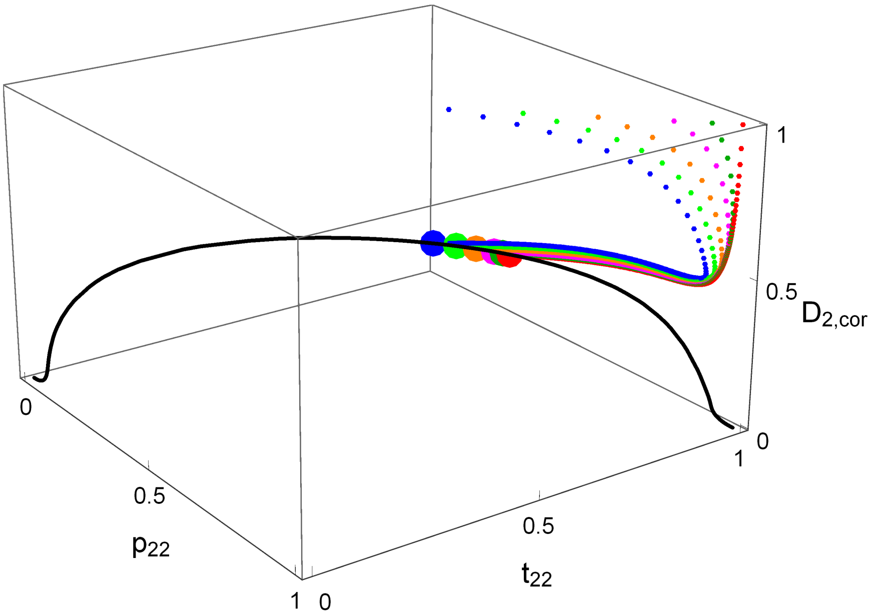

3.1.3 Generating Figures 3c, 3d (see main text for details)

```
initvalD0 = {{0.99, 0.99, 0}, {0.95, 0.95, 0},
   {0.9, 0.9, 0}, {0.8, 0.8, 0}, {0.7, 0.7, 0}, {0.6, 0.6, 0}};
```

```
tabdatfixD0[ss_, αα_, mm_, r_, tend_][nvals_] := Table[
  {n, Transpose[Table[datsymfix1[initvalD0[[i, 1]], initvalD0[[i, 2]], initvalD0[[i,
         3]], ss, αα, mm, r, tend][[n, 4 ;; 6]] // Chop, {i, 1, 6}]]}, {n, nvals}]
```

Data for specific generations ({1,10,50,100,500,1000,5000,10000,20000,50000}) :

Figure 3c

```
Round[10^4 * tabdatfixD0[0.2, 10, 0.01, 0.001, 50000][
    {1, 10, 50, 100, 500, 1000, 5000, 10000, 20000, 50000}]] / 10^4. // TableForm
```

	0.99	0.95	0.9	0.8	0.7	0.6
1.	0.99	0.95	0.9	0.8	0.7	0.6
	0.	0.	0.	0.	0.	0.
	0.9775	0.9278	0.8635	0.7491	0.6489	0.5536
10.	0.9873	0.9749	0.9452	0.8547	0.7528	0.6475
	0.0123	0.0216	0.0345	0.0438	0.039	0.0308
	0.9769	0.9241	0.8255	0.591	0.4797	0.4019
50.	0.9871	0.9732	0.9173	0.7061	0.5818	0.4856
	0.0125	0.0247	0.0676	0.1474	0.1291	0.098
	0.9764	0.9232	0.8213	0.5138	0.379	0.3113
100.	0.987	0.9729	0.9138	0.623	0.4655	0.3758
	0.0126	0.0249	0.0702	0.177	0.1498	0.1103
	0.9721	0.9155	0.7929	0.4247	0.2928	0.2502
500.	0.9862	0.97	0.8914	0.5217	0.3554	0.2943
	0.0133	0.0273	0.0849	0.1844	0.1391	0.1032
	0.9665	0.9049	0.7483	0.3526	0.2704	0.2473
1000.	0.9851	0.9655	0.8534	0.4351	0.3251	0.2902
	0.0142	0.0308	0.1073	0.1733	0.1279	0.1011
	0.9086	0.6953	0.28	0.1162	0.2063	0.2379
5000.	0.9674	0.8046	0.3401	0.1004	0.2333	0.2765
	0.028	0.1259	0.1419	0.0406	0.0848	0.0939
	0.7237	0.3679	0.1612	0.1071	0.1975	0.2362
10000.	0.831	0.4479	0.168	0.085	0.22	0.2741
	0.1025	0.1443	0.0629	0.0307	0.0773	0.0925
	0.5545	0.3089	0.1548	0.1068	0.1966	0.236
20000.	0.66	0.3708	0.1577	0.0844	0.2186	0.2738
	0.1212	0.1145	0.0566	0.0304	0.0765	0.0923
	0.5506	0.308	0.1548	0.1068	0.1966	0.236
50000.	0.6557	0.3695	0.1577	0.0844	0.2186	0.2738
	0.1204	0.1139	0.0566	0.0304	0.0765	0.0923

Computing divergence between subpopulations (p22 - p12, t22 - t12):

```
divergefixD0[ss_, αα_, mm_, r_, tend_][nvals_] :=
 Table[{n, Transpose[Table[{datsymfix1[initvalD0[[i, 1]], initvalD0[[i, 2]],
           initvalD0[[i, 3]], ss, αα, mm, r, tend][[n]][[4]] -
        datsymfix1[initvalD0[[i, 1]], initvalD0[[i, 2]], initvalD0[[i, 3]],
           ss, αα, mm, r, tend][[n]][[1]], datsymfix1[initvalD0[[i, 1]],
           initvalD0[[i, 2]], initvalD0[[i, 3]], ss, αα, mm, r, tend][[n]][[5]] -
        datsymfix1[initvalD0[[i, 1]], initvalD0[[i, 2]], initvalD0[[i, 3]],
           ss, αα, mm, r, tend][[n]][[2]]} // Chop, {i, 1, 6}]]}, {n, nvals}]
```

```
Round[10^4 * divergefixD0[0.2, 10, 0.01, 0.001, 50000][
    {1, 10, 50, 100, 500, 1000, 5000, 10000, 20000, 50000}]] / 10^4. // TableForm
```

1.	0.99<br>0.99	0.95<br>0.95	0.9<br>0.9	0.8<br>0.8	0.7<br>0.7	0.6<br>0.6
10.	0.9666<br>0.9767	0.9163<br>0.9643	0.8507<br>0.9348	0.7324<br>0.8449	0.6291<br>0.7439	0.5319<br>0.6397
50.	0.9656<br>0.9764	0.9122<br>0.9625	0.8113<br>0.9069	0.5591<br>0.6957	0.425<br>0.5697	0.3314<br>0.4725
100.	0.9646<br>0.9762	0.9108<br>0.9621	0.8066<br>0.9034	0.4765<br>0.6131	0.3068<br>0.4522	0.2134<br>0.3579
500.	0.9565<br>0.9748	0.8995<br>0.9587	0.7745<br>0.8807	0.3807<br>0.5125	0.2088<br>0.3422	0.1357<br>0.2727
1000.	0.946<br>0.9728	0.8842<br>0.9535	0.7249<br>0.8424	0.3025<br>0.4265	0.1826<br>0.3119	0.1318<br>0.2682
5000.	0.8408<br>0.944	0.6315<br>0.7858	0.21<br>0.3304	0.0439<br>0.0971	0.1041<br>0.2193	0.119<br>0.2535
10000.	0.552<br>0.7539	0.2426<br>0.4146	0.0722<br>0.1601	0.0324<br>0.082	0.0926<br>0.2056	0.1166<br>0.2508
20000.	0.2553<br>0.4723	0.1592<br>0.3243	0.0639<br>0.1499	0.032<br>0.0814	0.0914<br>0.2042	0.1164<br>0.2505
50000.	0.2478<br>0.4645	0.1578<br>0.3228	0.0638<br>0.1499	0.032<br>0.0814	0.0914<br>0.2042	0.1163<br>0.2505

```
plotcor2cr0001endp =
 ListPointPlot3D[Table[ddat = datsymfix1[initvalD0[[i, 1]], initvalD0[[i, 2]],
      initvalD0[[i, 3]], 0.2, 10, 0.01, 0.001, 50000][[50000, 4 ;; 6]];
    {{ddat[[1]], ddat[[2]], ddat[[3]] / (√ (ddat[[1]] (1 - ddat[[1]]) ddat[[2]] (1 -
               ddat[[2]])))}}, {i, 1, 6}], PlotStyle → {Directive[Red, PointSize[0.035]],
    Directive[Darker[Green], PointSize[0.035]], Directive[Magenta, PointSize[0.035]],
    Directive[Orange, PointSize[0.035]], Directive[Green, PointSize[0.035]],
    Directive[Blue, PointSize[0.035]]}, AxesLabel → {p2, t2, D}];
plotcor2cr0001all = ListPointPlot3D[Table[Table[ddat = datsymfix1[initvalD0[[i, 1]],
       initvalD0[[i, 2]], initvalD0[[i, 3]], 0.2, 10, 0.01, 0.001, 50000][[n, 4 ;; 6]];
     {ddat[[1]], ddat[[2]], ddat[[3]] /
       (√ (ddat[[1]] (1 - ddat[[1]]) ddat[[2]] (1 - ddat[[2]])))},
     {n, Flatten[{Join[Table[j, {j, 1, 1000}], Table[1000 + 10 j, {j, 1, 4000}]]}]}],
    {i, 1, 6}], PlotStyle → {Red, Darker[Green], Magenta, Orange, Green, Blue}];
plotcor2cr0001 = Show[plotcor2cr0001all, plotcor2cr0001endp, plotcurveequilcor10,
  ImageSize → 450, PlotRange → {{0, 1}, {0, 1}, {-0.005, 1.005}},
  AxesLabel → {Text[Style[p22, Black, 15]],
    Text[Style[t22, Black, 15]], Text[Style[D2,cor, Black, 15]]},
  LabelStyle → Directive[Black, FontFamily → "Helvetica", 12], BoxRatios → {1, 1, 0.6},
  ViewPoint → {3.4, -2.4, 1.6}, AxesEdge → {{0, 0}, {1, 0}, {1, 1}},
  Ticks → {{0, 0.5, 1}, {{0, "     0"}, 0.5, 1}, {0, 0.5, 1}}]
```

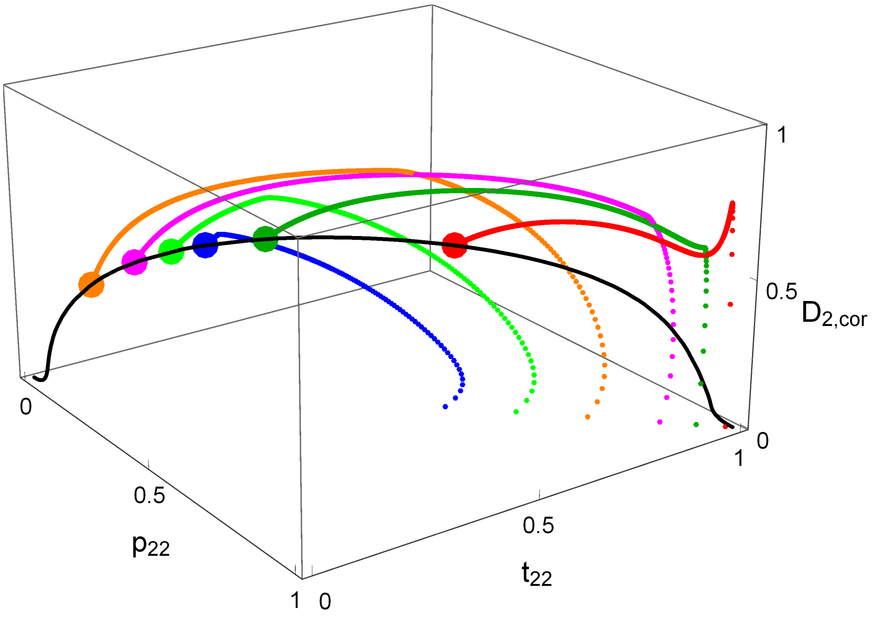

Figure 3d

```
Round[10^4 * tabdatfixD0[0.2, 10, 0.01, 0.1, 5000][{1, 10, 50, 100, 500, 1000, 5000}]] /
  10^4. // TableForm
```

	0.99	0.95	0.9	0.8	0.7	0.6
1.	0.99	0.95	0.9	0.8	0.7	0.6
	0.	0.	0.	0.	0.	0.
	0.9726	0.9255	0.867	0.7586	0.6591	0.5631
10.	0.9867	0.9745	0.9469	0.8622	0.7645	0.6616
	0.0111	0.0192	0.0325	0.0561	0.0702	0.0767
	0.9305	0.859	0.7527	0.6053	0.5134	0.4346
50.	0.9765	0.9437	0.8624	0.7196	0.6209	0.5311
	0.018	0.0389	0.08	0.1244	0.1368	0.1371
	0.8573	0.7337	0.6043	0.4762	0.4043	0.3425
100.	0.9428	0.844	0.7167	0.5774	0.4935	0.4178
	0.0375	0.0845	0.1223	0.1378	0.1353	0.1264
	0.5969	0.4917	0.4089	0.3347	0.2936	0.2539
500.	0.7054	0.5905	0.4945	0.4036	0.3508	0.2981
	0.1115	0.1275	0.1284	0.1195	0.1103	0.0986
	0.5962	0.4912	0.4085	0.3345	0.2934	0.2537
1000.	0.7046	0.5899	0.494	0.4033	0.3506	0.2979
	0.1116	0.1275	0.1284	0.1194	0.1103	0.0985
	0.5962	0.4912	0.4085	0.3345	0.2934	0.2537
5000.	0.7046	0.5899	0.494	0.4033	0.3506	0.2979
	0.1116	0.1275	0.1284	0.1194	0.1103	0.0985

Divergence:

```
Round[10^4 * divergefixD0[0.2, 10, 0.01, 0.1, 5000][{1, 10, 50, 100, 500, 1000, 5000}]] /
  10^4. // TableForm
```

1.	0.99	0.95	0.9	0.8	0.7	0.6
	0.99	0.95	0.9	0.8	0.7	0.6
10.	0.9568	0.9095	0.8501	0.7394	0.6383	0.5416
	0.9755	0.9634	0.936	0.8521	0.7554	0.6538
50.	0.8771	0.8076	0.7018	0.5498	0.4535	0.3731
	0.9576	0.926	0.8461	0.7049	0.6071	0.5188
100.	0.7436	0.6312	0.5079	0.3812	0.3099	0.2516
	0.9023	0.8111	0.6896	0.5546	0.4733	0.4009
500.	0.2551	0.235	0.2059	0.1719	0.1501	0.1273
	0.4746	0.4449	0.4004	0.3461	0.3097	0.27
1000.	0.2537	0.234	0.2053	0.1715	0.1498	0.1271
	0.4732	0.4439	0.3997	0.3456	0.3093	0.2698
5000.	0.2537	0.234	0.2053	0.1715	0.1498	0.1271
	0.4732	0.4439	0.3997	0.3456	0.3093	0.2698

Note that the divergence here, both for p and t, is higher than for the smaller recombination rate in Figure 3c!

```
plotcor2dr01endp =
 ListPointPlot3D[Table[ddat = datsymfix1[initvalD0[[i, 1]], initvalD0[[i, 2]],
      initvalD0[[i, 3]], 0.2, 10, 0.01, 0.1, 5000][[5000, 4 ;; 6]];
    {{ddat[[1]], ddat[[2]], ddat[[3]] / (√ (ddat[[1]] (1 - ddat[[1]]) ddat[[2]] (1 -
              ddat[[2]])))}}, {i, 1, 6}], PlotStyle → {Directive[Red, PointSize[0.035]],
     Directive[Darker[Green], PointSize[0.035]], Directive[Magenta, PointSize[0.035]],
     Directive[Orange, PointSize[0.035]], Directive[Green, PointSize[0.035]],
     Directive[Blue, PointSize[0.035]]}, AxesLabel → {p2, t2, D}];
plotcor2dr01all = ListPointPlot3D[Table[Table[ddat = datsymfix1[initvalD0[[i, 1]],
       initvalD0[[i, 2]], initvalD0[[i, 3]], 0.2, 10, 0.01, 0.1, 5000][[n, 4 ;; 6]];
     {ddat[[1]], ddat[[2]], ddat[[3]] /
       (√ (ddat[[1]] (1 - ddat[[1]]) ddat[[2]] (1 - ddat[[2]])))},
     {n, Flatten[{Join[Table[j, {j, 1, 1000}], Table[1000 + 10 j, {j, 1, 400}]]}]}],
    {i, 1, 6}], PlotStyle → {Red, Darker[Green], Magenta, Orange, Green, Blue}];
plotcor2dr01 = Show[plotcor2dr01all, plotcor2dr01endp, plotcurveequilcor10,
  ImageSize → 450, PlotRange → {{0, 1}, {0, 1}, {-0.005, 1.005}},
  AxesLabel → {Text[Style[p22, Black, 15]],
    Text[Style[t22, Black, 15]], Text[Style[D2,cor, Black, 15]]},
  LabelStyle → Directive[Black, FontFamily → "Helvetica", 12], BoxRatios → {1, 1, 0.6},
  ViewPoint → {3.4, -2.4, 1.6}, AxesEdge → {{0, 0}, {1, 0}, {1, 1}},
  Ticks → {{0, 0.5, 1}, {{0, "     0"}, 0.5, 1}, {0, 0.5, 1}}]
```

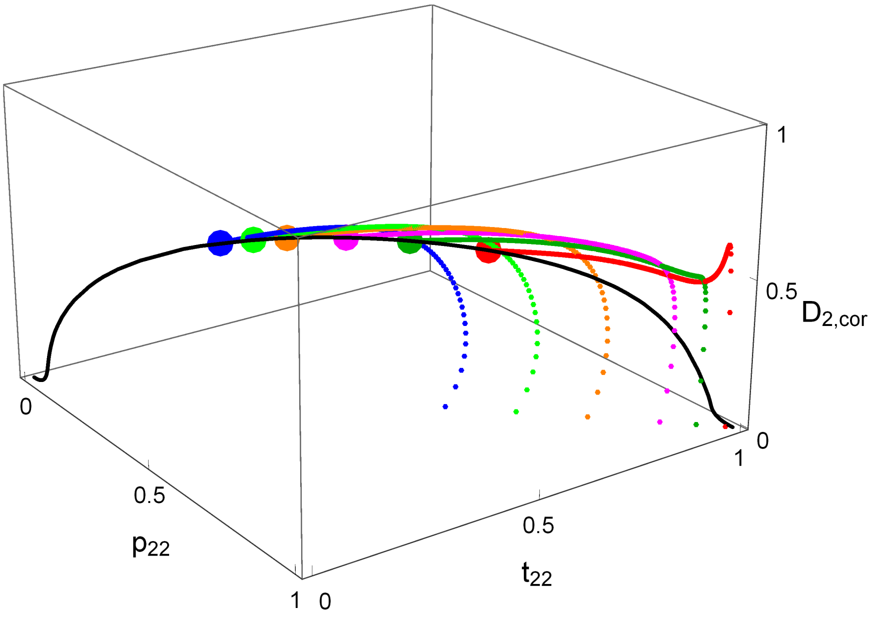

Other 3D figures were produced analogously

3.2 Generate initial data with nearly symmetric initial conditions

```
datsymsymnear[p20_, t20_, dd0_, s_, α_, m_, r_, tend_] :=
 datsymsymnear[p20, t20, dd0, s, α, m, r, tend] =
  NestList[itgen[s, s, α, α, m, m, r], {(1 - p2) RandomReal[{0.95, 1.05}],
     (1 - t2) RandomReal[{0.95, 1.05}], 0.98 * Min[dd * RandomReal[{0.95, 1.05}],
       Min[(1 - p2) RandomReal[{0.95, 1.05}] * (1 - (1 - t2) RandomReal[{0.95, 1.05}]),
        (1 - (1 - p2) RandomReal[{0.95, 1.05}]) * (1 - t2) RandomReal[{0.95, 1.05}]]],
     p2, t2, dd} /. {p2 → p20, t2 → t20, dd → dd0}, tend]
```

```
tabdatsymnear[ss_, αα_, mm_, r_, tend_][nvals_] :=
 Table[{n, Transpose[Table[datsymsymnear[initvals[[i, 1]], initvals[[i, 2]],
        initvals[[i, 3]], ss, αα, mm, r, tend][[n]] // Chop, {i, 1, 8}]]}, {n, nvals}]
```

The following provides an example for the fact that slight deviations from symmetric initial conditions (in this case by up to 5%) lead to equilibria that are also close to the symmetric equilibrium.

```
SeedRandom[5]
```

```
tabdatsymnear[0.2, 10, 0.01, 0.01, 10000][{1, 10, 100, 1000, 5000, 10000}] // TableForm
```

1	0.047504	0.104707	0.308284	0.298176	0.0502728	0.0957219	0.285646	0.2940
	0.0478251	0.0972991	0.0502547	0.289269	0.0511082	0.0960357	0.0495603	0.3107
	0	0	0	0	0.0382716	0.0681139	0.0291374	0.1971
	0.95	0.9	0.7	0.7	0.95	0.9	0.7	0.7
	0.95	0.9	0.95	0.7	0.95	0.9	0.95	0.7
	0	0	0	0	0.04	0.07	0.03	0.2
10	0.0690184	0.139359	0.354977	0.327689	0.0238609	0.0382095	0.3946	0.0395
	0.0237022	0.0556002	0.239333	0.225387	0.0133083	0.0173711	0.255147	0.0305
	0.0192383	0.0300719	0.0362455	0.0210997	0.012598	0.0157453	0.105109	0.0274
	0.927858	0.866856	0.654343	0.670703	0.978517	0.959254	0.573493	0.9459
	0.975257	0.947898	0.770308	0.772628	0.987152	0.981808	0.716413	0.9618
	0.0199225	0.0286655	0.035643	0.0210803	0.0121895	0.0164315	0.114012	0.0345
100	0.0852246	0.195301	0.426903	0.419742	0.0330694	0.0472057	0.374527	0.033
	0.0296503	0.0972022	0.321911	0.314236	0.0147077	0.0178431	0.264674	0.014
	0.0248472	0.0652225	0.0918839	0.0896788	0.0137572	0.0161617	0.0999837	0.013
	0.910631	0.817269	0.583205	0.578504	0.969535	0.950209	0.5885	0.957
	0.968628	0.913016	0.689071	0.683857	0.985836	0.981488	0.696225	0.983
	0.0262359	0.0590644	0.0903139	0.0899407	0.0132568	0.0167547	0.108813	0.015
1000	0.317089	0.372389	0.379522	0.373623	0.226052	0.256262	0.355183	0.204557
	0.206161	0.262297	0.269806	0.263644	0.12265	0.148691	0.244507	0.103139
	0.0977929	0.106811	0.105158	0.103616	0.0769609	0.0844003	0.098942	0.0643932
	0.662475	0.648281	0.630555	0.624618	0.791993	0.725626	0.608514	0.728574
	0.773332	0.759058	0.740709	0.734521	0.893078	0.834256	0.717651	0.836449
	0.104753	0.100949	0.102515	0.104077	0.0683369	0.092177	0.108531	0.095076
5000	0.362008	0.383018	0.377593	0.371665	0.385235	0.362092	0.354615	0.330943
	0.251563	0.27345	0.267775	0.261592	0.275774	0.25165	0.24392	0.219651
	0.101032	0.106611	0.105224	0.103665	0.107168	0.101056	0.0989397	0.091804
	0.616773	0.637788	0.632473	0.626577	0.639939	0.616859	0.609094	0.583438
	0.726331	0.748226	0.742712	0.736577	0.750453	0.726421	0.718267	0.691077
	0.106664	0.101089	0.102551	0.104132	0.100487	0.106642	0.108562	0.114345
10 000	0.362008	0.383018	0.377593	0.371665	0.385235	0.362092	0.354615	0.330943
	0.251563	0.27345	0.267775	0.261592	0.275775	0.25165	0.24392	0.219651
	0.101032	0.106611	0.105224	0.103665	0.107168	0.101056	0.0989397	0.091804
	0.616773	0.637788	0.632473	0.626577	0.639939	0.616859	0.609094	0.583438
	0.726331	0.748226	0.742712	0.736577	0.750453	0.726421	0.718267	0.691077
	0.106664	0.101089	0.102551	0.104132	0.100487	0.106642	0.108562	0.114345
