# Supplementary material for: The Effects on Parapatric Divergence of Linkage between Preference and Trait Loci versus Pleiotropy
Source: Genes (Basel). 2018 Apr 17;9(4):217. doi: 10.3390/genes9040217 (PMC5924559; doi:10.3390/genes9040217)
Supplement: Supplementary file 1 [file genes-09-00217-s001.zip › Servedio and Burger Supplementary Information proofs/Supplementary Materials File S2.pdf]

Simulations starting from allopatric equilibrium

From Servedio and Bürger, The Effects on Parapatric Divergence of Linkage between Preference and Trait Loci versus Pleiotropy, *Genes*

Double click on the arrows on the right-hand side to open each sub-section

Simulation program with T expressed in both sexes

```
PTmigprog := (

  c1 = 41; (* number of items in alphaparams *)
  k1c = 1; (* number of items in p2arams *)
  k2c = 1; (* number of items in t2params *)
  eqp2pop1 = Table[0, {count, c1}];
  eqt2pop1 = Table[0, {count, c1}];
  eqdiseqpop1 = Table[0, {count, c1}];
  eqp2pop2 = Table[0, {count, c1}];
  eqt2pop2 = Table[0, {count, c1}];
  eqdiseqpop2 = Table[0, {count, c1}];
  eqp2popdiff = Table[0, {count, c1}];
  eqt2popdiff = Table[0, {count, c1}];
  (*plot1=Table[0,{count,c1},{k2,k2c},{k1,k1c}];
  plot2=Table[0,{count,c1},{k2,k2c},{k1,k1c}];*)
  Do[αparams = {0.05, 0.07, 0.1, 0.2, 0.5, 0.6, 0.7, 0.8, 0.9, 1.0, 1.5, 2.0, 3.0, 5.0,
     6.0, 7.0, 8.0, 9.0, 10.0 , 11.0, 12.0, 13.0, 14.0, 15.0, 16.0, 17.0, 18.0, 19.0,
     20.0, 22.5, 25.0, 30.0, 35.0, 40.0, 35.0, 50.0, 60.0, 75.0, 100.0, 150.0, 200.0};
   α2 = αparams[[count]];
   α1 = αparams[[count]];
   gen = 1;
   t22 = 0.95;

   p2curve[t2_, s_, α1_, α2_] :=
```

$$-\frac{1}{(1+s)^2\,(1+s\,t2)\,(\alpha1+\alpha2+\alpha1\,\alpha2)}\Big(3\,s+2\,s^2+8\,s^2\,t2+5\,s^3\,t2+7\,s^3\,t2^2+4\,s^4\,t2^2+2\,s^4\,t2^3+ s^5\,t2^3-\alpha1+s\,\alpha1+s^2\,\alpha1-4\,s\,t2\,\alpha1+s^2\,t2\,\alpha1+2\,s^3\,t2\,\alpha1-5\,s^2\,t2^2\,\alpha1-s^3\,t2^2\,\alpha1+ s^4\,t2^2\,\alpha1-2\,s^3\,t2^3\,\alpha1-s^4\,t2^3\,\alpha1+3\,s\,t2\,\alpha2+5\,s^2\,t2\,\alpha2+2\,s^3\,t2\,\alpha2+5\,s^2\,t2^2\,\alpha2+ 8\,s^3\,t2^2\,\alpha2+3\,s^4\,t2^2\,\alpha2+2\,s^3\,t2^3\,\alpha2+3\,s^4\,t2^3\,\alpha2+s^5\,t2^3\,\alpha2-t2\,\alpha1\,\alpha2+2\,s^2\,t2\,\alpha1\,\alpha2+ s^3\,t2\,\alpha1\,\alpha2-3\,s\,t2^2\,\alpha1\,\alpha2-3\,s^2\,t2^2\,\alpha1\,\alpha2+s^3\,t2^2\,\alpha1\,\alpha2+s^4\,t2^2\,\alpha1\,\alpha2-2\,s^2\,t2^3\,\alpha1\,\alpha2-$$

```
3 s^3 t2^3 α1 α2 - s^4 t2^3 α1 α2 - s √((1 + s t2) (1 + s t2 + α1 - t2 α1) (1 + t2 α2 + s t2 (1 + α2))
    (1 + α1 - t2 α1 + t2 α2 + s^5 t2^3 (1 + α2) + s t2 (5 - 2 (-1 + t2) α1 + (3 + 2 t2) α2) +
      s^4 t2^2 (5 + (4 + t2) α2 - (-1 + t2) α1 (1 + α2)) + s^3 t2 (-t2^2 α1 α2 + (2 + α1) (2 + α2) +
        t2 (6 - 2 α1 + 8 α2)) + s^2 t2 (6 + 4 α2 + α1 (2 + α2) + t2 (4 + 6 α2 - α1 (2 + α2)))));

discurve[t2_, s_, α1_, α2_] :=
  1 / ((1 + s)^2 (α1 + α2 + α1 α2)) (1 + 4 s t2 + s^2 t2 + 5 s^2 t2^2 + 2 s^3 t2^2 + 2 s^3 t2^3 + s^4 t2^3 + α1 -
    t2 α1 + 3 s t2 α1 + s^2 t2 α1 - 3 s t2^2 α1 + s^2 t2^2 α1 + s^3 t2^2 α1 - 2 s^2 t2^3 α1 - s^3 t2^3 α1 +
    t2 α2 + s t2 α2 + 3 s t2^2 α2 + 4 s^2 t2^2 α2 + s^3 t2^2 α2 + 2 s^2 t2^3 α2 + 3 s^3 t2^3 α2 + s^4 t2^3 α2 +
    t2 α1 α2 + s t2 α1 α2 - t2^2 α1 α2 + s t2^2 α1 α2 + 3 s^2 t2^2 α1 α2 + s^3 t2^2 α1 α2 - 2 s t2^3 α1 α2 -
    3 s^2 t2^3 α1 α2 - s^3 t2^3 α1 α2 - √((1 + s t2) (1 + s t2 + α1 - t2 α1) (1 + t2 α2 + s t2 (1 + α2))
      (1 + α1 - t2 α1 + t2 α2 + s^5 t2^3 (1 + α2) + s t2 (5 - 2 (-1 + t2) α1 + (3 + 2 t2) α2) +
        s^4 t2^2 (5 + (4 + t2) α2 - (-1 + t2) α1 (1 + α2)) + s^3 t2 (-t2^2 α1 α2 + (2 + α1) (2 + α2) + t2
          (6 - 2 α1 + 8 α2)) + s^2 t2 (6 + 4 α2 + α1 (2 + α2) + t2 (4 + 6 α2 - α1 (2 + α2)))));

p22 = p2curve[t22, s, α1, α2];
diseq2 = discurve[t22, s, α1, α2];
t12 = 0.07;
p12 = 1 - p2curve[1 - t12, s, α1, α2];
diseq1 = discurve[1 - t12, s, α1, α2];


x1[1] = (1 - p12) (1 - t12) + diseq1;
x1[2] = (1 - p12) t12 - diseq1;
x1[3] = p12 (1 - t12) - diseq1;
x1[4] = p12 t12 + diseq1;
x2[1] = (1 - p22) (1 - t22) + diseq2;
x2[2] = (1 - p22) t22 - diseq2;
x2[3] = p22 (1 - t22) - diseq2;
x2[4] = p22 t22 + diseq2;

(*override allele freqs for full LD*)
(*x1[1] = 1.0;
x1[2] = 0;
x1[3] = 0;
x1[4] = 0;
x2[1] = 0.05;
x2[2] = 0;
x2[3] = 0;
x2[4] = 0.95;*)


Do[ x1t1[cc] = 0, {cc, 1, 4}];
Do[ x2t1[cc] = 0, {cc, 1, 4}];
```

```
F1 = Table[0, {i, 4}, {j, 4}];
F2 = Table[0, {i, 4}, {j, 4}];

Coef = Table[1, {i, 4}, {j, 4}];
Do[Coef[[i, 1]] = (1 + α1), {i, 1, 2}];
Do[Coef[[i, 3]] = (1 + α1), {i, 1, 2}];
Do[Coef[[i, 2]] = (1 + α2), {i, 3, 4}];
Do[Coef[[i, 4]] = (1 + α2), {i, 3, 4}];

While[(gen < numgen),

 If[gen > 1,
  Do[x1[cc] = x1t1[cc], {cc, 1, 4}];
  Do[x2[cc] = x2t1[cc], {cc, 1, 4}]]; (* recursions *)


 x1m[1] = (1 - m1) x1[1] + m1 x2[1];
 x1m[2] = (1 - m1) x1[2] + m1 x2[2];
 x1m[3] = (1 - m1) x1[3] + m1 x2[3];
 x1m[4] = (1 - m1) x1[4] + m1 x2[4];

 x2m[1] = (1 - m2) x2[1] + m2 x1[1];
 x2m[2] = (1 - m2) x2[2] + m2 x1[2];
 x2m[3] = (1 - m2) x2[3] + m2 x1[3];
 x2m[4] = (1 - m2) x2[4] + m2 x1[4];

 w1barm = x1m[1] (1 + s) + x1m[2] + x1m[3] (1 + s) + x1m[4];
 xnsm1[1] = x1m[1] (1 + s) / w1barm;
 xnsm1[2] = x1m[2] / w1barm;
 xnsm1[3] = x1m[3] (1 + s) / w1barm;
 xnsm1[4] = x1m[4] / w1barm;

 Do[xnsf1[i] = xnsm1[i], {i, 4}];

 w2barm =  x2m[1] + (1 + s) x2m[2] + x2m[3] + (1 + s) x2m[4];
 xnsm2[1] = x2m[1] / w2barm;
 xnsm2[2] = (1 + s) x2m[2] / w2barm;
 xnsm2[3] = x2m[3] / w2barm;
 xnsm2[4] = (1 + s) x2m[4] / w2barm;

 Do[xnsf2[i] = xnsm2[i], {i, 4}];

 Mate1 = Table[xnsf1[i] xnsm1[j], {i, 4}, {j, 4}];
 G1 = Mate1 Coef;

 Do[z1[i] = Sum[xnsm1[j] Coef[[i, j]], {j, 4}], {i, 4}];
```

```
 Do[F1[[i, j]] = G1[[i, j]] / z1[i], {i, 4}, {j, 4}];

 Mate2 = Table[xnsf2[i] xnsm2[j], {i, 4}, {j, 4}];
 G2 = Mate2 Coef;

 Do[z2[i] = Sum[xnsm2[j] Coef[[i, j]], {j, 4}], {i, 4}];

 Do[F2[[i, j]] = G2[[i, j]] / z2[i], {i, 4}, {j, 4}];

 x1t1[1] = Simplify[F1[[1, 1]] + (1 / 2) F1[[1, 2]] + (1 / 2) F1[[1, 3]] +
    (1 / 2) (1 - r) F1[[1, 4]] + (1 / 2) F1[[2, 1]] + (1 / 2) r F1[[2, 3]] +
    (1 / 2) F1[[3, 1]] + (1 / 2) r F1[[3, 2]] + (1 / 2) (1 - r) F1[[4, 1]]];
 x1t1[2] = Simplify[(1 / 2) F1[[1, 2]] + (1 / 2) r F1[[1, 4]] + (1 / 2) F1[[2, 1]] +
    F1[[2, 2]] + (1 / 2) (1 - r) F1[[2, 3]] + (1 / 2) F1[[2, 4]] +
    (1 / 2) (1 - r) F1[[3, 2]] + (1 / 2) r F1[[4, 1]] + (1 / 2) F1[[4, 2]]];
 x1t1[3] = Simplify[(1 / 2) F1[[1, 3]] + (1 / 2) r F1[[1, 4]] + (1 / 2) (1 - r) F1[[2, 3]] +
    (1 / 2) F1[[3, 1]] + (1 / 2) (1 - r) F1[[3, 2]] + F1[[3, 3]] +
    (1 / 2) F1[[3, 4]] + (1 / 2) r F1[[4, 1]] + (1 / 2) F1[[4, 3]]];
 x1t1[4] = Simplify[(1 / 2) (1 - r) F1[[1, 4]] + (1 / 2) r F1[[2, 3]] +
    (1 / 2) F1[[2, 4]] + (1 / 2) r F1[[3, 2]] + (1 / 2) F1[[3, 4]] +
    (1 / 2) (1 - r) F1[[4, 1]] + (1 / 2) F1[[4, 2]] + (1 / 2) F1[[4, 3]] + F1[[4, 4]]];

 x2t1[1] = Simplify[F2[[1, 1]] + (1 / 2) F2[[1, 2]] + (1 / 2) F2[[1, 3]] +
    (1 / 2) (1 - r) F2[[1, 4]] + (1 / 2) F2[[2, 1]] + (1 / 2) r F2[[2, 3]] +
    (1 / 2) F2[[3, 1]] + (1 / 2) r F2[[3, 2]] + (1 / 2) (1 - r) F2[[4, 1]]];
 x2t1[2] = Simplify[(1 / 2) F2[[1, 2]] + (1 / 2) r F2[[1, 4]] + (1 / 2) F2[[2, 1]] +
    F2[[2, 2]] + (1 / 2) (1 - r) F2[[2, 3]] + (1 / 2) F2[[2, 4]] +
    (1 / 2) (1 - r) F2[[3, 2]] + (1 / 2) r F2[[4, 1]] + (1 / 2) F2[[4, 2]]];
 x2t1[3] = Simplify[(1 / 2) F2[[1, 3]] + (1 / 2) r F2[[1, 4]] + (1 / 2) (1 - r) F2[[2, 3]] +
    (1 / 2) F2[[3, 1]] + (1 / 2) (1 - r) F2[[3, 2]] + F2[[3, 3]] +
    (1 / 2) F2[[3, 4]] + (1 / 2) r F2[[4, 1]] + (1 / 2) F2[[4, 3]]];
 x2t1[4] = Simplify[(1 / 2) (1 - r) F2[[1, 4]] + (1 / 2) r F2[[2, 3]] +
    (1 / 2) F2[[2, 4]] + (1 / 2) r F2[[3, 2]] + (1 / 2) F2[[3, 4]] +
    (1 / 2) (1 - r) F2[[4, 1]] + (1 / 2) F2[[4, 2]] + (1 / 2) F2[[4, 3]] + F2[[4, 4]]];

 gen++;]; (* loop *)

p1t1 = Simplify[x1t1[3] + x1t1[4]];
t1t1 = Simplify[x1t1[2] + x1t1[4]];
diseq1t1 = x1t1[1] x1t1[4] - x1t1[2] x1t1[3];
p2t1 = Simplify[x2t1[3] + x2t1[4]];
t2t1 = Simplify[x2t1[2] + x2t1[4]];
diseq2t1 = x2t1[1] x2t1[4] - x2t1[2] x2t1[3];

Print["run ", count, ",  α = ", α1, ",   gen = ", gen];
```

```
    (*If finding starting p2 and diseq *)
    (*eqp2pop1[[count]] = {αparams[[count]],p12};
    eqt2pop1[[count]] ={αparams[[count]], t12};
    eqdiseqpop1[[count]] = {αparams[[count]],diseq1};
    eqp2pop2[[count]]={αparams[[count]],p22};
    eqt2pop2[[count]] = {αparams[[count]],t22};
    eqdiseqpop2[[count]] = {αparams[[count]],diseq2};
    eqp2popdiff[[count]] = {αparams[[count]],p22-p12};
    eqt2popdiff[[count]] = {αparams[[count]],t22-t12}*)

    eqp2pop1[[count]] = {αparams[[count]], p1t1};
    eqt2pop1[[count]] = {αparams[[count]], t1t1};
    eqdiseqpop1[[count]] = {αparams[[count]], diseq1t1};
    eqp2pop2[[count]] = {αparams[[count]], p2t1};
    eqt2pop2[[count]] = {αparams[[count]], t2t1};
    eqdiseqpop2[[count]] = {αparams[[count]], diseq2t1};
    eqp2popdiff[[count]] = {αparams[[count]], p2t1 - p1t1};
    eqt2popdiff[[count]] = {αparams[[count]], t2t1 - t1t1},
    {count, c1}]
  )
```

Example of code needed to run this:

```
pC = 0;
s = 0.038;
r = 0;
m1 = 0;
m2 = 0.01;
PTmigprog
```

Simulation program with T expressed only in males

```
PTmigprog := (

  c1 = 41; (* number of items in alphaparams *)
  k1c = 1; (* number of items in p2arams *)
  k2c = 1; (* number of items in t2params *)
  eqp2pop1 = Table[0, {count, c1}];
  eqt2pop1 = Table[0, {count, c1}];
  eqdiseqpop1 = Table[0, {count, c1}];
  eqp2pop2 = Table[0, {count, c1}];
  eqt2pop2 = Table[0, {count, c1}];
  eqdiseqpop2 = Table[0, {count, c1}];
```

```
eqp2popdiff = Table[0, {count, c1}];
eqt2popdiff = Table[0, {count, c1}];
(*plot1=Table[0,{count,c1},{k2,k2c},{k1,k1c}];
plot2=Table[0,{count,c1},{k2,k2c},{k1,k1c}];*)
Do[αparams = {0.05, 0.07, 0.1, 0.2, 0.5, 0.6, 0.7, 0.8, 0.9, 1.0, 1.5, 2.0, 3.0, 5.0,
   6.0, 7.0, 8.0, 9.0, 10.0 , 11.0, 12.0, 13.0, 14.0, 15.0, 16.0, 17.0, 18.0, 19.0,
   20.0, 22.5, 25.0, 30.0, 35.0, 40.0, 35.0, 50.0, 60.0, 75.0, 100.0, 150.0, 200.0};
 α2 = αparams[[count]];
 α1 = αparams[[count]];
 gen = 1;
 t22 = 0.95;
```

$$\text{p2curve[t2\_, s\_, α1\_, α2\_] := } -\frac{(s-\alpha1)\,(1+t22\,\alpha2+s\,t22\,(1+\alpha2))}{(1+s)\,(\alpha1+\alpha2+\alpha1\,\alpha2)};$$

discurve[t2_, s_, α1_, α2_] :=

$$\frac{1}{(1+s)^2\,(\alpha1+\alpha2+\alpha1\,\alpha2)^2}\Big(\alpha1+s\,\alpha1+2\,s\,t2\,\alpha1+2\,s^2\,t2\,\alpha1+s^2\,t2^2\,\alpha1+s^3\,t2^2\,\alpha1+\alpha1^2+ s\,\alpha1^2-t2\,\alpha1^2+s^2\,t2\,\alpha1^2-s\,t2^2\,\alpha1^2-s^2\,t2^2\,\alpha1^2+\alpha2+s\,\alpha2+2\,s\,t2\,\alpha2+2\,s^2\,t2\,\alpha2+ s^2\,t2^2\,\alpha2+s^3\,t2^2\,\alpha2+2\,\alpha1\,\alpha2+2\,s\,\alpha1\,\alpha2+4\,s\,t2\,\alpha1\,\alpha2+4\,s^2\,t2\,\alpha1\,\alpha2+2\,s^2\,t2^2\,\alpha1\,\alpha2+ 2\,s^3\,t2^2\,\alpha1\,\alpha2+\alpha1^2\,\alpha2+s\,\alpha1^2\,\alpha2+2\,s\,t2\,\alpha1^2\,\alpha2+2\,s^2\,t2\,\alpha1^2\,\alpha2-t2^2\,\alpha1^2\,\alpha2- 3\,s\,t2^2\,\alpha1^2\,\alpha2-2\,s^2\,t2^2\,\alpha1^2\,\alpha2+t2\,\alpha2^2+2\,s\,t2\,\alpha2^2+s^2\,t2\,\alpha2^2+s\,t2^2\,\alpha2^2+2\,s^2\,t2^2\,\alpha2^2+ s^3\,t2^2\,\alpha2^2+2\,t2\,\alpha1\,\alpha2^2+4\,s\,t2\,\alpha1\,\alpha2^2+2\,s^2\,t2\,\alpha1\,\alpha2^2-t2^2\,\alpha1\,\alpha2^2-s\,t2^2\,\alpha1\,\alpha2^2+ s^2\,t2^2\,\alpha1\,\alpha2^2+s^3\,t2^2\,\alpha1\,\alpha2^2+t2\,\alpha1^2\,\alpha2^2+2\,s\,t2\,\alpha1^2\,\alpha2^2+s^2\,t2\,\alpha1^2\,\alpha2^2-t2^2\,\alpha1^2\,\alpha2^2- 2\,s\,t2^2\,\alpha1^2\,\alpha2^2-s^2\,t2^2\,\alpha1^2\,\alpha2^2-\sqrt{\Big((1+s)^2\,(1+s\,t2+\alpha1-t2\,\alpha1)\,(\alpha1+\alpha2+\alpha1\,\alpha2)^2\,(1+t2\,\alpha2+s\,t2\,(1+\alpha2))\,\big(1+\alpha1-t2\,\alpha1+t2\,\alpha2+2\,s\,t2\,(1+\alpha2)+s^2\,t2\,(1+\alpha2)\big)\Big)}\Big);$$

```
 p22 = p2curve[t22, s, α1, α2];
 diseq2 = discurve[t22, s, α1, α2];
 t12 = 0.07;
 p12 = 1 - p2curve[1 - t12, s, α1, α2];
 diseq1 = discurve[1 - t12, s, α1, α2];


 x1[1] = (1 - p12) (1 - t12) + diseq1;
 x1[2] = (1 - p12) t12 - diseq1;
 x1[3] = p12 (1 - t12) - diseq1;
 x1[4] = p12 t12 + diseq1;
 x2[1] = (1 - p22) (1 - t22) + diseq2;
 x2[2] = (1 - p22) t22 - diseq2;
 x2[3] = p22 (1 - t22) - diseq2;
 x2[4] = p22 t22 + diseq2;

 (*override allele freqs for full LD*)
 (*x1[1] = 1.0;
 x1[2] = 0;
 x1[3] = 0;
```

```
x1[4] = 0;
x2[1] = 0.05;
x2[2] = 0;
x2[3] = 0;
x2[4] = 0.95;*)


Do[ x1t1[cc] = 0, {cc, 1, 4}];
Do[ x2t1[cc] = 0, {cc, 1, 4}];

F1 = Table[0, {i, 4}, {j, 4}];
F2 = Table[0, {i, 4}, {j, 4}];

Coef = Table[1, {i, 4}, {j, 4}];
Do[Coef[[i, 1]] = (1 + α1), {i, 1, 2}];
Do[Coef[[i, 3]] = (1 + α1), {i, 1, 2}];
Do[Coef[[i, 2]] = (1 + α2), {i, 3, 4}];
Do[Coef[[i, 4]] = (1 + α2), {i, 3, 4}];

While[(gen < numgen),

 If[gen > 1,
  Do[x1[cc] = x1t1[cc], {cc, 1, 4}];
  Do[x2[cc] = x2t1[cc], {cc, 1, 4}]]; (* recursions *)


 x1m[1] = (1 - m1) x1[1] + m1 x2[1];
 x1m[2] = (1 - m1) x1[2] + m1 x2[2];
 x1m[3] = (1 - m1) x1[3] + m1 x2[3];
 x1m[4] = (1 - m1) x1[4] + m1 x2[4];

 x2m[1] = (1 - m2) x2[1] + m2 x1[1];
 x2m[2] = (1 - m2) x2[2] + m2 x1[2];
 x2m[3] = (1 - m2) x2[3] + m2 x1[3];
 x2m[4] = (1 - m2) x2[4] + m2 x1[4];

 w1barm = x1m[1] (1 + s) + x1m[2] + x1m[3] (1 + s) + x1m[4];
 xnsm1[1] = x1m[1] (1 + s) / w1barm;
 xnsm1[2] = x1m[2] / w1barm;
 xnsm1[3] = x1m[3] (1 + s) / w1barm;
 xnsm1[4] = x1m[4] / w1barm;

 Do[xnsf1[i] = x1m[i], {i, 4}]; (* Trait not expressed in females *)

 w2barm =  x2m[1] + (1 + s) x2m[2] + x2m[3] + (1 + s) x2m[4];
 xnsm2[1] = x2m[1] / w2barm;
```

```
xnsm2[2] = (1 + s) x2m[2] / w2barm;
xnsm2[3] = x2m[3] / w2barm;
xnsm2[4] = (1 + s) x2m[4] / w2barm;

Do[xnsf2[i] = x2m[i], {i, 4}]; (* Trait not expressed in females *)

Mate1 = Table[xnsf1[i] xnsm1[j], {i, 4}, {j, 4}];
G1 = Mate1 Coef;

Do[z1[i] = Sum[xnsm1[j] Coef[[i, j]], {j, 4}], {i, 4}];

Do[F1[[i, j]] = G1[[i, j]] / z1[i], {i, 4}, {j, 4}];

Mate2 = Table[xnsf2[i] xnsm2[j], {i, 4}, {j, 4}];
G2 = Mate2 Coef;

Do[z2[i] = Sum[xnsm2[j] Coef[[i, j]], {j, 4}], {i, 4}];

Do[F2[[i, j]] = G2[[i, j]] / z2[i], {i, 4}, {j, 4}];

x1t1[1] = Simplify[F1[[1, 1]] + (1 / 2) F1[[1, 2]] + (1 / 2) F1[[1, 3]] +
    (1 / 2) (1 - r) F1[[1, 4]] + (1 / 2) F1[[2, 1]] + (1 / 2) r F1[[2, 3]] +
    (1 / 2) F1[[3, 1]] + (1 / 2) r F1[[3, 2]] + (1 / 2) (1 - r) F1[[4, 1]]];
x1t1[2] = Simplify[(1 / 2) F1[[1, 2]] + (1 / 2) r F1[[1, 4]] + (1 / 2) F1[[2, 1]] +
    F1[[2, 2]] + (1 / 2) (1 - r) F1[[2, 3]] + (1 / 2) F1[[2, 4]] +
    (1 / 2) (1 - r) F1[[3, 2]] + (1 / 2) r F1[[4, 1]] + (1 / 2) F1[[4, 2]]];
x1t1[3] = Simplify[(1 / 2) F1[[1, 3]] + (1 / 2) r F1[[1, 4]] + (1 / 2) (1 - r) F1[[2, 3]] +
    (1 / 2) F1[[3, 1]] + (1 / 2) (1 - r) F1[[3, 2]] + F1[[3, 3]] +
    (1 / 2) F1[[3, 4]] + (1 / 2) r F1[[4, 1]] + (1 / 2) F1[[4, 3]]];
x1t1[4] = Simplify[(1 / 2) (1 - r) F1[[1, 4]] + (1 / 2) r F1[[2, 3]] +
    (1 / 2) F1[[2, 4]] + (1 / 2) r F1[[3, 2]] + (1 / 2) F1[[3, 4]] +
    (1 / 2) (1 - r) F1[[4, 1]] + (1 / 2) F1[[4, 2]] + (1 / 2) F1[[4, 3]] + F1[[4, 4]]];

x2t1[1] = Simplify[F2[[1, 1]] + (1 / 2) F2[[1, 2]] + (1 / 2) F2[[1, 3]] +
    (1 / 2) (1 - r) F2[[1, 4]] + (1 / 2) F2[[2, 1]] + (1 / 2) r F2[[2, 3]] +
    (1 / 2) F2[[3, 1]] + (1 / 2) r F2[[3, 2]] + (1 / 2) (1 - r) F2[[4, 1]]];
x2t1[2] = Simplify[(1 / 2) F2[[1, 2]] + (1 / 2) r F2[[1, 4]] + (1 / 2) F2[[2, 1]] +
    F2[[2, 2]] + (1 / 2) (1 - r) F2[[2, 3]] + (1 / 2) F2[[2, 4]] +
    (1 / 2) (1 - r) F2[[3, 2]] + (1 / 2) r F2[[4, 1]] + (1 / 2) F2[[4, 2]]];
x2t1[3] = Simplify[(1 / 2) F2[[1, 3]] + (1 / 2) r F2[[1, 4]] + (1 / 2) (1 - r) F2[[2, 3]] +
    (1 / 2) F2[[3, 1]] + (1 / 2) (1 - r) F2[[3, 2]] + F2[[3, 3]] +
    (1 / 2) F2[[3, 4]] + (1 / 2) r F2[[4, 1]] + (1 / 2) F2[[4, 3]]];
x2t1[4] = Simplify[(1 / 2) (1 - r) F2[[1, 4]] + (1 / 2) r F2[[2, 3]] +
    (1 / 2) F2[[2, 4]] + (1 / 2) r F2[[3, 2]] + (1 / 2) F2[[3, 4]] +
    (1 / 2) (1 - r) F2[[4, 1]] + (1 / 2) F2[[4, 2]] + (1 / 2) F2[[4, 3]] + F2[[4, 4]]];
```

```
 gen++;]; (* loop *)

   p1t1 = Simplify[x1t1[3] + x1t1[4]];
   t1t1 = Simplify[x1t1[2] + x1t1[4]];
   diseq1t1 = x1t1[1] x1t1[4] - x1t1[2] x1t1[3];
   p2t1 = Simplify[x2t1[3] + x2t1[4]];
   t2t1 = Simplify[x2t1[2] + x2t1[4]];
   diseq2t1 = x2t1[1] x2t1[4] - x2t1[2] x2t1[3];

   Print["run ", count, ",  α = ", α1, ",   gen = ", gen];

   (*If finding starting p2 an diseq *)
   (*eqp2pop1[[count]] = {αparams[[count]],p12};
   eqt2pop1[[count]] ={αparams[[count]], t12};
   eqdiseqpop1[[count]] = {αparams[[count]],diseq1};
   eqp2pop2[[count]]={αparams[[count]],p22};
   eqt2pop2[[count]] = {αparams[[count]],t22};
   eqdiseqpop2[[count]] = {αparams[[count]],diseq2};
   eqp2popdiff[[count]] = {αparams[[count]],p22-p12};
   eqt2popdiff[[count]] = {αparams[[count]],t22-t12}*)

   eqp2pop1[[count]] = {αparams[[count]], p1t1};
   eqt2pop1[[count]] = {αparams[[count]], t1t1};
   eqdiseqpop1[[count]] = {αparams[[count]], diseq1t1};
   eqp2pop2[[count]] = {αparams[[count]], p2t1};
   eqt2pop2[[count]] = {αparams[[count]], t2t1};
   eqdiseqpop2[[count]] = {αparams[[count]], diseq2t1};
   eqp2popdiff[[count]] = {αparams[[count]], p2t1 - p1t1};
   eqt2popdiff[[count]] = {αparams[[count]], t2t1 - t1t1},
   {count, c1}]
 )
```

Example of code needed to run this:

```
pC = 0;
s = 0.038;
r = 0;
m1 = 0;
m2 = 0.01;
PTmigprog
```
