# Supplementary material for: The Effects on Parapatric Divergence of Linkage between Preference and Trait Loci versus Pleiotropy
Source: Genes (Basel). 2018 Apr 17;9(4):217. doi: 10.3390/genes9040217 (PMC5924559; doi:10.3390/genes9040217)
Supplement: Supplementary file 1 [file genes-09-00217-s001.zip › Servedio and Burger Supplementary Information proofs/Supplementary Materials File S5.pdf]

THE EFFECTS ON PARAPATRIC DIVERGENCE OF LINKAGE BETWEEN PREFERENCE AND TRAIT LOCI VERSUS PLEIOTROPY

Maria R. Servedio and Reinhard Bürger

Supplementary Materials File S5 – Asymmetric preferences

In order to understand the counterintuitive observation of the loss of allele T_2 with strong preferences when $\alpha_2 > \alpha_1$, we tracked genotype frequencies through the life cycle for selected generations when $\alpha_2 > 1.1\ \alpha_1$ and $\alpha_1 = 500$. We examined both cases in which the loci within each population started in linkage equilibrium and cases which started with high linkage disequilibrium, with selection occurring in both sexes. We discovered the loss of T_2 to be due to two primary factors. First, changes in allele frequency at the trait locus due to viability selection are unequal in the two populations even when the selection coefficients are symmetrical. Specifically, local adaptation results in larger changes in allele frequency in the population with more genetic variation; this can cause viability selection to lead to a global (across both populations) reduction in frequency of the globally more common allele. Second, the homogenization of preference frequencies across the populations can cause sexual selection to favor rare trait alleles in each population (see Servedio and Bürger 2014). We observed these two factors to lead to the loss of T_2 through the following sequence of steps:

1) When $\alpha_2 > \alpha_1$, t_2 initially increases globally, due to the stronger global preference for T_2 than for T_1 (p_2 also increases, due to the quickly-established LD between the P and T loci). Therefore, right before viability selection in the life cycle, t_2 (and p_2) are higher in population 2 than t_1 (and p_1) are in population 1. Given the same selection coefficient for local adaptation, s, in both populations, this lower genetic variation in population 2 than population 1 leads to less of an increase of t_2 (and p_2) in population 2 than of t_1 (and p_1) in population 1. Consequently, summed across both populations, t_2 (and p_2) decrease in the viability selection step of the life cycle in these early generations.

2) In contrast, the frequency of t_2 (and p_2) increases globally in the sexual selection step of the life cycle in the generations shortly after secondary contact. This occurs because the global frequencies of the preferences, p_1 and p_2, are similar to one another given the starting conditions that we used, and $\alpha_2 > \alpha_1$. More specifically, sexual selection causes t_2 to decrease in population 2 and increase in population 1(because the preferences quickly become more homogenized between populations than the trait frequencies, see Servedio and Bürger, 2014), but the increase of t_2 in population 1 is bigger than its decrease in population 2 because of the high α_2. In early generations after contact the increase of t_2 (and p_2) from sexual selection overwhelms the decrease in t_2 (and p_2) from viability selection, and t_2 (and p_2) globally increase overall.

3) Over the course of generations, the global overall increase of t_2 (and p_2) across the life cycle becomes smaller and smaller. At some point the viability selection decrease in t_2 (and p_2) overwhelms the sexual selection increase in these frequencies and t_2 (and p_2) start to decrease across the life cycle. It was observed that the global decrease in p_2 occurs at least a generation or so before the global decrease in t_2 occurs. The frequency p_2 was also observed to have a greater global decrease at the viability selection step of the life cycle than does t_2. This may initially seem surprising, since viability selection acts directly on the T locus and frequencies at the P locus change only due to indirect selection. The greater global decrease in p_2 than t_2 at the viability selection step occurs, however, because LD between the P and T loci is higher in population 1 (where there is higher variation) than in population 2. The decrease in t_2 (and p_2) in population 1 due to viability selection, which drives the global decrease in t_2 and p_2, thus leads to a disproportionally larger decrease in p_2 in the population in which the change (decrease) in t_2 is already greater.

4) In later generations, the effects of viability and sexual selection on t_2 (and p_2) are reversed from what they are in early generations after contact. Once the global values of t_2 and p_2 decrease sufficiently, there becomes more genetic variation in population 2 than in population 1 (due to the overall lower frequency of t_2 and p_2 than *of* t_1 and p_1 – the opposite of the pattern in the early generations after contact). Viability selection thus becomes more effective at increasing t_2 (and p_2) in population 2 than at increasing t_1 (and p_1) in population 1; the frequencies t_2 and p_2 thus increase globally at the viability selection stage of the life cycle. However, when p_1 is sufficiently more frequent than p_2 across both populations this frequency difference more than compensates for the fact that $\alpha_2 > \alpha_1$, and sexual selection leads to a global increase in t_1 (and p_1). This eventually can lead, as observed, to the global loss of the T_2 allele.
