# Supplementary material for: The Effects on Parapatric Divergence of Linkage between Preference and Trait Loci versus Pleiotropy
Source: Genes (Basel). 2018 Apr 17;9(4):217. doi: 10.3390/genes9040217 (PMC5924559; doi:10.3390/genes9040217)

Supplementary Material File S4:

The preference/trait model with natural selection only on males, recombination between the preference and trait loci, and migration between two demes

by

Maria Servedio and Reinhard Bürger

1. Model and derivation of recursion relations

P2 is a preference allele for T2, with strength $\alpha 2$,
P1 is a preference allele for T1, with strength $\alpha 1$.
Migration into deme 1 occurs at rate $m1$, i.e., a fraction $m1$ of individuals in deme 1 is replaced by individuals from deme 2; migration into deme 2 occurs at rate $m2$.
T1 (T2) is favored by viability selection in males in deme 1 (2) with selection coefficient $s1$ ($s2$).
The recombination rate between the P and T locus is $r \geq 0$.

Four genotypes, with frequencies (on the island) denoted by
x1[1] = frequency of P1T1 in deme 1
x1[2] = frequency of P1T2 in deme 1
x1[3] = frequency of P2T1 in deme 1
x1[4] = frequency of P2T2 in deme 1
x2[1] = frequency of P1T1 in deme 2
x2[2] = frequency of P1T2 in deme 2
x2[3] = frequency of P2T1 in deme 2
x2[4] = frequency of P2T2 in deme 2

We use mainly allele frequencies and linkage disequilibria to describe the dynamics:

p12 = x1[3]+x1[4] = frequency of preference allele P2 in deme 1 (Note: the ordering of subscripts differs from that in the manuscript)
t12 = x1[2]+x1[4] = frequency of trait allele T2 in deme 1 (Note: the ordering of subscripts differs from that in the manuscript)
dd1 = x1[1]x1[4]-x1[2]x1[3] = linkage disequilibrium between P and T in deme 1
p22, t22, dd2 are the analogous quantities in deme 2

The phenotype-matching model is obtained as a special case if *r* = 0 and p12 = t12 and p22 = t22. As a consequence, linkage disequilibrium is maximal, i.e., dd1 = t12(1-t12) and dd2 = t22(1-t22).

1.1 Modeling the stages of the life cycle

1.1.1 Migration

Gamete frequencies after migration

```
x1m[1] = (1 - m1) x1[1] + m1 x2[1];
x1m[2] = (1 - m1) x1[2] + m1 x2[2];
x1m[3] = (1 - m1) x1[3] + m1 x2[3];
x1m[4] = (1 - m1) x1[4] + m1 x2[4];
```

```
x2m[1] = (1 - m2) x2[1] + m2 x1[1];
x2m[2] = (1 - m2) x2[2] + m2 x1[2];
x2m[3] = (1 - m2) x2[3] + m2 x1[3];
x2m[4] = (1 - m2) x2[4] + m2 x1[4];
```

1.1.2 Viability selection in males

Gamete frequencies after viability selection

males in pop 1:

```
w1barm = 1 + s1 (x1m[1] + x1m[3]);
```

```
xnsm1[1] = x1m[1] (1 + s1) / w1barm;
xnsm1[2] = x1m[2] / w1barm;
xnsm1[3] = x1m[3] (1 + s1) / w1barm;
xnsm1[4] = x1m[4] / w1barm;
```

```
Clear[x1m, w1barm, x2m, w2barm]
```

```
Simplify[Sum[xnsm1[i], {i, 4}]]
```

$$\frac{1}{\text{w1barm}} \left(\text{x1m}[1] + \text{s x1m}[1] + \text{x1m}[2] + \text{x1m}[3] + \text{s x1m}[3] + \text{x1m}[4]\right)$$

```
Simplify[Sum[xnsm1[i], {i, 4}] /.
  {x1[1] → 1 - x1[2] - x1[3] - x1[4], x2[1] → 1 - x2[2] - x2[3] - x2[4]}]
```

1

no viability selection in females:

```
Do[xnsf1[i] = x1m[i], {i, 4}];
```

males in pop 2:

```
w2barm = 1 + s2 (x2m[2] + x2m[4]);
```

```
xnsm2[1] = x2m[1] / w2barm;
xnsm2[2] = (1 + s2) x2m[2] / w2barm;
xnsm2[3] = x2m[3] / w2barm;
xnsm2[4] = (1 + s2) x2m[4] / w2barm;
```

no viability selection in females:

```
Do[xnsf2[i] = x2m[i], {i, 4}];
```

1.1.3 Sexual selection

P1 prefers T1 (by a factor of 1+α1), and P2 prefers T2 (by a factor of 1+α2)

pop 1

```
Mate1 = Table[xnsf1[i] xnsm1[j], {i, 4}, {j, 4}];
Coef = Table[1, {i, 4}, {j, 4}];
Do[Coef[[i, 1]] = (1 + α1), {i, 1, 2}];
Do[Coef[[i, 3]] = (1 + α1), {i, 1, 2}];
Do[Coef[[i, 2]] = (1 + α2), {i, 3, 4}];
Do[Coef[[i, 4]] = (1 + α2), {i, 3, 4}];
G1 = Mate1 Coef;
```

```
Simplify[Coef] // TableForm
```

1 + α1	1	1 + α1	1
1 + α1	1	1 + α1	1
1	1 + α2	1	1 + α2
1	1 + α2	1	1 + α2

```
Do[z1[i] = Sum[xnsm1[j] Coef[[i, j]], {j, 4}], {i, 4}]
```

```
F1 = Table[0, {i, 4}, {j, 4}];
Do[F1[[i, j]] = G1[[i, j]] / z1[i], {i, 4}, {j, 4}];
```

```
TableForm[Simplify[F1]]
```

```
- ———————————————————————————————————————————————————————————————————————————————————————————
  (-1+s1 ((-1+m1) x1[1]+(-1+m1) x1[3]-m1 (x2[1]+x2[3]))) (-(-1+m1) (1+s1) (1+α1) x1[1]+x1[2]-m1 x1[2]+x1[3]-m1 x1[3]+s1 x1[3
                                                                                                                            (:
- ———————————————————————————————————————————————————————————————————————————————————————————
  (-1+s1 ((-1+m1) x1[1]+(-1+m1) x1[3]-m1 (x2[1]+x2[3]))) (-(-1+m1) (1+s1) (1+α1) x1[1]+x1[2]-m1 x1[2]+x1[3]-m1 x1[3]+s1 x1[3
                                                                                                  (1+s1)^2 ((-1+m1) x1[1]-m1
- ———————————————————————————————————————————————————————————————————————————————————————————
  (-1+s1 ((-1+m1) x1[1]+(-1+m1) x1[3]-m1 (x2[1]+x2[3]))) (-(-1+m1) (1+s1) x1[1]-(-1+m1) (1+α2) x1[2]+x1[3]-m1 x1[3]+s1 x1[3]
                                                                                                  (1+s1) ((-1+m1) x1[1]-m1 :
- ———————————————————————————————————————————————————————————————————————————————————————————
  (-1+s1 ((-1+m1) x1[1]+(-1+m1) x1[3]-m1 (x2[1]+x2[3]))) (-(-1+m1) (1+s1) x1[1]-(-1+m1) (1+α2) x1[2]+x1[3]-m1 x1[3]+s1 x1[3]
```

pop 2

```
Mate2 = Table[xnsf2[i] xnsm2[j], {i, 4}, {j, 4}];
G2 = Mate2 Coef;
```

```
Do[z2[i] = Sum[xnsm2[j] Coef[[i, j]], {j, 4}], {i, 4}]
```

```
F2 = Table[0, {i, 4}, {j, 4}];
Do[F2[[i, j]] = G2[[i, j]] / z2[i], {i, 4}, {j, 4}];
```

```
TableForm[F2]
```

$\frac{(1+\alpha1)\ \text{xnsf2[1] xnsm2[1]}}{(1+\alpha1)\ \text{xnsm2[1]}+\text{xnsm2[2]}+(1+\alpha1)\ \text{xnsm2[3]}+\text{xnsm2[4]}}$	$\frac{\text{xnsf2[1] xnsm2[2]}}{(1+\alpha1)\ \text{xnsm2[1]}+\text{xnsm2[2]}+(1+\alpha1)\ \text{xnsm2[3]}+\text{xnsm2[4]}}$	$\frac{(1+\alpha1}{(1+\alpha1)\ \text{xnsm2[1]+xn}}$
$\frac{(1+\alpha1)\ \text{xnsf2[2] xnsm2[1]}}{(1+\alpha1)\ \text{xnsm2[1]}+\text{xnsm2[2]}+(1+\alpha1)\ \text{xnsm2[3]}+\text{xnsm2[4]}}$	$\frac{\text{xnsf2[2] xnsm2[2]}}{(1+\alpha1)\ \text{xnsm2[1]}+\text{xnsm2[2]}+(1+\alpha1)\ \text{xnsm2[3]}+\text{xnsm2[4]}}$	$\frac{(1+\alpha1}{(1+\alpha1)\ \text{xnsm2[1]+xn}}$
$\frac{\text{xnsf2[3] xnsm2[1]}}{\text{xnsm2[1]}+(1+\alpha2)\ \text{xnsm2[2]}+\text{xnsm2[3]}+(1+\alpha2)\ \text{xnsm2[4]}}$	$\frac{(1+\alpha2)\ \text{xnsf2[3] xnsm2[2]}}{\text{xnsm2[1]}+(1+\alpha2)\ \text{xnsm2[2]}+\text{xnsm2[3]}+(1+\alpha2)\ \text{xnsm2[4]}}$	$\frac{\text{xr}}{\text{xnsm2[1]}+(1+\alpha2)\ \text{xn}}$
$\frac{\text{xnsf2[4] xnsm2[1]}}{\text{xnsm2[1]}+(1+\alpha2)\ \text{xnsm2[2]}+\text{xnsm2[3]}+(1+\alpha2)\ \text{xnsm2[4]}}$	$\frac{(1+\alpha2)\ \text{xnsf2[4] xnsm2[2]}}{\text{xnsm2[1]}+(1+\alpha2)\ \text{xnsm2[2]}+\text{xnsm2[3]}+(1+\alpha2)\ \text{xnsm2[4]}}$	$\frac{\text{xr}}{\text{xnsm2[1]}+(1+\alpha2)\ \text{xn}}$

1.1.4 Recombination

pop 1

```
x1t1[1] = Simplify[F1[[1, 1]] + (1/2) F1[[1, 2]] + (1/2) F1[[1, 3]] +
    (1/2) (1 - r) F1[[1, 4]] + (1/2) F1[[2, 1]] + (1/2) r F1[[2, 3]] +
    (1/2) F1[[3, 1]] + (1/2) r F1[[3, 2]] + (1/2) (1 - r) F1[[4, 1]]];

x1t1[2] = Simplify[(1/2) F1[[1,2]] + (1/2) r F1[[1,4]] +
    (1/2) F1[[2,1]] + F1[[2,2]] + (1/2)(1-r)F1[[2,3]] +
    (1/2) F1[[2,4]] + (1/2) (1-r) F1[[3,2]] +
    (1/2) r F1[[4,1]] + (1/2) F1[[4,2]]];

x1t1[3] = Simplify[(1/2) F1[[1,3]] + (1/2) r F1[[1,4]] +
    (1/2) (1-r) F1[[2,3]] + (1/2) F1[[3,1]] +
    (1/2) (1-r) F1[[3,2]] + F1[[3,3]] + (1/2) F1[[3,4]] +
    (1/2) r F1[[4,1]] + (1/2) F1[[4,3]]];

x1t1[4] = Simplify[(1/2) (1-r) F1[[1,4]] + (1/2) r F1[[2,3]] +
    (1/2) F1[[2,4]] + (1/2) r F1[[3,2]] + (1/2) F1[[3,4]] +
    (1/2) (1-r) F1[[4,1]] + (1/2) F1[[4,2]] + (1/2) F1[[4,3]] + F1[[4,4]]];
```

pop 2

```
x2t1[1] = Simplify[F2[[1, 1]] + (1/2) F2[[1, 2]] + (1/2) F2[[1, 3]] +
    (1/2) (1 - r) F2[[1, 4]] + (1/2) F2[[2, 1]] + (1/2) r F2[[2, 3]] +
    (1/2) F2[[3, 1]] + (1/2) r F2[[3, 2]] + (1/2) (1 - r) F2[[4, 1]]];

x2t1[2] = Simplify[(1/2) F2[[1,2]] + (1/2) r F2[[1,4]] +
    (1/2) F2[[2,1]] + F2[[2,2]] + (1/2)(1-r)F2[[2,3]] +
    (1/2) F2[[2,4]] + (1/2) (1-r) F2[[3,2]] +
    (1/2) r F2[[4,1]] + (1/2) F2[[4,2]]];

x2t1[3] = Simplify[(1/2) F2[[1,3]] + (1/2) r F2[[1,4]] +
    (1/2) (1-r) F2[[2,3]] + (1/2) F2[[3,1]] +
    (1/2) (1-r) F2[[3,2]] + F2[[3,3]] + (1/2) F2[[3,4]] +
    (1/2) r F2[[4,1]] + (1/2) F2[[4,3]]];

x2t1[4] = Simplify[(1/2) (1-r) F2[[1,4]] + (1/2) r F2[[2,3]] +
    (1/2) F2[[2,4]] + (1/2) r F2[[3,2]] + (1/2) F2[[3,4]] +
    (1/2) (1-r) F2[[4,1]] + (1/2) F2[[4,2]] + (1/2) F2[[4,3]] + F2[[4,4]]];
```

1.2 Recursions for allele frequencies and LD

The notation here is that the 1st subscript represents the population and the second represents the allele.

So all is done with respect to allele 2 in both populations.

1.2.1 Derivation of the recursion relations

```
x1[1] = (1 - p12) (1 - t12) + dd1;
x1[2] = (1 - p12) t12 - dd1;
x1[3] = p12 (1 - t12) - dd1;
x1[4] = p12 t12 + dd1;
x2[1] = (1 - p22) (1 - t22) + dd2;
x2[2] = (1 - p22) t22 - dd2;
x2[3] = p22 (1 - t22) - dd2;
x2[4] = p22 t22 + dd2;
```

```
p1t1 = Simplify[x1t1[3] + x1t1[4]]
```

```
t1t1 = Simplify[x1t1[2] + x1t1[4]]
```

```
dd1t1 = x1t1[1] x1t1[4] - x1t1[2] x1t1[3]
```

```
p2t1 = Simplify[x2t1[3] + x2t1[4]]
```

```
t2t1 = Simplify[x2t1[2] + x2t1[4]]
```

```
dd2t1 = x2t1[1] x2t1[4] - x2t1[2] x2t1[3]
```

```
{p1t1, t1t1, dd1t1, p2t1, t2t1, dd2t1}
```

1.2.2 The main recursion relation, itgenmale[s1, s2, α1, α2, m1, m2, r][{p12, t12, dd1, p22, t22, dd2}]

For given parameters {s1, s2, α1, α2, m1, m2, r}, itgenmale computes {p12 (t + 1), t12 (t + 1), dd1 (t + 1), p22 (t + 1), t22 (t + 1), dd2 (t + 1)} from {p12 (t), t12 (t), dd1 (t), p22 (t), t22 (t), dd2 (t)} . Because computation of p1t1 etc (see above) is time consuming, we define itgenmale[s1, s2, α1, α2, m1, m2, r][{p12, t12, dd1, p22, t22, dd2}] explicitly by the right-hand side of {p1t1, t1t1, dd1t1, p2t1, t2t1, dd2t1}.

```
itgenmale[s1_, s2_, α1_, α2_, m1_, m2_, r_][{p12_, t12_, dd1_, p22_, t22_, dd2_}] :=
  {(dd1 (s1 + α1 + s1 α1) (-1 + s1 (-1 + t12) - t12 α2) +
      (-1 + m1)^2 m1 p12^2 (1 + s1) (t12 - t22) (α1 + α2 + α1 α2) + m1^3 p22 (t12 - t22)
       (s1^2 (t12 - t22) (1 + α1) + (-t12 + t22) α1 α2 + p22 (α1 + α2 + α1 α2) +
         s1 (-(t12 - t22) (α1 (-1 + α2) + α2) + p22 (α1 + α2 + α1 α2))) +
     m1 (-(s1 + α1 + s1 α1) (dd2 (1 + s1 - s1 t12 + t12 α2) + dd1 (-1 + s1 (-1 + 2 t12 - t22) -
              2 t12 α2 + t22 α2)) + p22 (2 + s1^2 (-1 + t12) (-2 + t12 + t22) (1 + α1) +
           dd1 α2 + 2 t12 α2 + α1 (dd1 (1 + α2) - (-2 + t12 + t22) (1 + t12 α2)) +
           s1 (4 - t22 + 4 α1 + dd1 α1 - 2 t22 α1 + dd1 α2 + dd1 α1 α2 - t12^2 (α1 (-1 + α2) +
                α2) - t12 (3 + α1 (4 + t22 (-1 + α2) - 2 α2) + (-2 + t22) α2)))) +
     m1^2 ((dd1 - dd2) (t12 - t22) (s1 + α1 + s1 α1) (s1 - α2) - p22^2 (1 + s1)
         (t12 - t22) (α1 + α2 + α1 α2) +
        p22 (-dd1 α1 + dd2 α1 + t12 α1 - t22 α1 - s1^2 (2 t12^2 - (-3 + t22) t22 - t12 (3 + t22))
            (1 + α1) - dd1 α2 + dd2 α2 - 2 t12 α2 + 2 t22 α2 - dd1 α1 α2 + dd2 α1 α2 -
           2 t12 α1 α2 + 2 t12^2 α1 α2 + 2 t22 α1 α2 - t12 t22 α1 α2 - t22^2 α1 α2 +
           s1 (2 t12^2 (α1 (-1 + α2) + α2) - t22^2 (α1 (-1 + α2) + α2) + t22
```

$$
\begin{array}{l}
(-3+2\,\alpha 1\,(-2+\alpha 2)+2\,\alpha 2)-t12\,(-3+\alpha 1\,(2\,(-2+\alpha 2)+t22\,(-1+\alpha 2))+\\
(2+t22)\,\alpha 2)-(dd1-dd2)\,(\alpha 1+\alpha 2+\alpha 1\,\alpha 2))))-\\
(-1+m1)\,p12\,(2+2\,\alpha 1+dd1\,\alpha 1-dd1\,m1\,\alpha 1+dd2\,m1\,\alpha 1-2\,t12\,\alpha 1+m1\,t12\,\alpha 1-\\
m1\,p22\,t12\,\alpha 1+2\,m1^2\,p22\,t12\,\alpha 1-m1\,t22\,\alpha 1+m1\,p22\,t22\,\alpha 1-\\
2\,m1^2\,p22\,t22\,\alpha 1+s1^2\,(2+(2-3\,m1+m1^2)\,t12^2-3\,m1\,t22+m1^2\,t22^2+\\
t12\,(-4-2\,m1^2\,t22+3\,m1\,(1+t22)))\,(1+\alpha 1)+dd1\,\alpha 2-dd1\,m1\,\alpha 2+\\
dd2\,m1\,\alpha 2+2\,t12\,\alpha 2-2\,m1\,t12\,\alpha 2-m1\,p22\,t12\,\alpha 2+2\,m1^2\,p22\,t12\,\alpha 2+\\
2\,m1\,t22\,\alpha 2+m1\,p22\,t22\,\alpha 2-2\,m1^2\,p22\,t22\,\alpha 2+dd1\,\alpha 1\,\alpha 2-dd1\,m1\,\alpha 1\,\alpha 2+\\
dd2\,m1\,\alpha 1\,\alpha 2+2\,t12\,\alpha 1\,\alpha 2-2\,m1\,t12\,\alpha 1\,\alpha 2-m1\,p22\,t12\,\alpha 1\,\alpha 2+2\,m1^2\,p22\,t12\,\alpha 1\,\alpha 2-\\
2\,t12^2\,\alpha 1\,\alpha 2+3\,m1\,t12^2\,\alpha 1\,\alpha 2-m1^2\,t12^2\,\alpha 1\,\alpha 2+2\,m1\,t22\,\alpha 1\,\alpha 2+m1\,p22\,t22\,\alpha 1\,\alpha 2-\\
2\,m1^2\,p22\,t22\,\alpha 1\,\alpha 2-3\,m1\,t12\,t22\,\alpha 1\,\alpha 2+2\,m1^2\,t12\,t22\,\alpha 1\,\alpha 2-m1^2\,t22^2\,\alpha 1\,\alpha 2+\\
s1\,(4+4\,\alpha 1+dd1\,\alpha 1+dd1\,\alpha 2+dd1\,\alpha 1\,\alpha 2-(2-3\,m1+m1^2)\,t12^2\,(\alpha 1\,(-1+\alpha 2)+\alpha 2)-\\
m1^2\,t22\,(t22\,(\alpha 1\,(-1+\alpha 2)+\alpha 2)+2\,p22\,(\alpha 1+\alpha 2+\alpha 1\,\alpha 2))+m1\,(-(dd1-dd2)\\
(\alpha 1+\alpha 2+\alpha 1\,\alpha 2)+t22\,(-3+(2+p22)\,\alpha 2+\alpha 1\,(-4+p22+2\,\alpha 2+p22\,\alpha 2)))+\\
t12\,(2\,(-2+\alpha 1\,(-3+\alpha 2)+\alpha 2)-m1\,(-3+(2+p22+3\,t22)\,\alpha 2+\\
\alpha 1\,(-4+p22-3\,t22+2\,\alpha 2+p22\,\alpha 2+3\,t22\,\alpha 2))+2\\
m1^2\,(t22\,(\alpha 1\,(-1+\alpha 2)+\alpha 2)+p22\,(\alpha 1+\alpha 2+\alpha 1\,\alpha 2)))))))\,/\\
(2\,(1+(1+(-1+m1)\,t12-m1\,t22)\,\alpha 1+s1\,(1+(-1+m1)\,t12-m1\,t22)\,(1+\alpha 1))\\
(1+s1\,(1+(-1+m1)\,t12-m1\,t22)+m1\,t22\,\alpha 2+t12\,(\alpha 2-m1\,\alpha 2))),\\
-((((-1+m1)\,t12-m1\,t22)\,(2+s1^2\,(1+(-1+m1)\,t12-m1\,t22)^2\,(1+\alpha 1)+\\
p12\,\alpha 2-m1\,p12\,\alpha 2+m1\,p22\,\alpha 2+2\,t12\,\alpha 2-2\,m1\,t12\,\alpha 2-p12\,t12\,\alpha 2+\\
2\,m1\,p12\,t12\,\alpha 2-m1^2\,p12\,t12\,\alpha 2-m1\,p22\,t12\,\alpha 2+m1^2\,p22\,t12\,\alpha 2+2\,m1\,t22\,\alpha 2-\\
m1\,p12\,t22\,\alpha 2+m1^2\,p12\,t22\,\alpha 2-m1^2\,p22\,t22\,\alpha 2-(1+(-1+m1)\,t12-m1\,t22)\,\alpha 1\\
(-1-t12\,\alpha 2+(-1+m1)\,p12\,(1+\alpha 2)-m1\,(p22+p22\,\alpha 2-t12\,\alpha 2+t22\,\alpha 2))-\\
s1\,(1+(-1+m1)\,t12-m1\,t22)\,(-3-p12\,\alpha 2+m1\,p12\,\alpha 2-m1\,p22\,\alpha 2-t12\,\alpha 2+\\
m1\,t12\,\alpha 2-m1\,t22\,\alpha 2+\alpha 1\,(-2+t12-t12\,\alpha 2+(-1+m1)\,p12\,(1+\alpha 2)-\\
m1\,(p22+t12-t22+p22\,\alpha 2-t12\,\alpha 2+t22\,\alpha 2))))))\,/\\
(2\,(1+(1+(-1+m1)\,t12-m1\,t22)\,\alpha 1+s1\,(1+(-1+m1)\,t12-m1\,t22)\,(1+\alpha 1))\\
(1+s1\,(1+(-1+m1)\,t12-m1\,t22)+m1\,t22\,\alpha 2+t12\,(\alpha 2-m1\,\alpha 2)))),\\
-\frac{1}{4}\,(-(((dd1\,(-1+m1)-dd2\,m1-p12\,t12+m1\,p12\,t12-m1\,p22\,t22)\,(dd1\,(-1+m1)-\\
dd2\,m1+t12-m1\,t12-p12\,t12+m1\,p12\,t12+m1\,t22-m1\,p22\,t22))\,/\\
(1+(1+(-1+m1)\,t12-m1\,t22)\,\alpha 1+s1\,(1+(-1+m1)\,t12-m1\,t22)\,(1+\alpha 1)))+\\
(r\,(dd1\,(-1+m1)-dd2\,m1-p12\,t12+m1\,p12\,t12-m1\,p22\,t22)\\
(-1+dd1\,(-1+m1)-dd2\,m1+p12-m1\,p12+m1\,p22+t12-\\
m1\,t12-p12\,t12+m1\,p12\,t12+m1\,t22-m1\,p22\,t22))\,/\\
(1+(1+(-1+m1)\,t12-m1\,t22)\,\alpha 1+s1\,(1+(-1+m1)\,t12-m1\,t22)\,(1+\alpha 1))-\\
((dd1\,(-1+m1)-dd2\,m1+t12-m1\,t12-p12\,t12+m1\,p12\,t12+m1\,t22-m1\,p22\,t22)\\
(-1+dd1\,(-1+m1)-dd2\,m1+p12-m1\,p12+m1\,p22+t12-\\
m1\,t12-p12\,t12+m1\,p12\,t12+m1\,t22-m1\,p22\,t22))\,/\\
(1+(1+(-1+m1)\,t12-m1\,t22)\,\alpha 1+s1\,(1+(-1+m1)\,t12-m1\,t22)\,(1+\alpha 1))+\\
(2\,(dd1-dd1\,m1+dd2\,m1-t12+m1\,t12+p12\,t12-m1\,p12\,t12-m1\,t22+m1\,p22\,t22)^2)\,/\\
(1+(1+(-1+m1)\,t12-m1\,t22)\,\alpha 1+s1\,(1+(-1+m1)\,t12-m1\,t22)\,(1+\alpha 1))-\\
((-1+r)\,(1+s1)\,(dd1\,(-1+m1)-dd2\,m1+p12-m1\,p12+m1\,p22-\\
p12\,t12+m1\,p12\,t12-m1\,p22\,t22)\,(dd1\,(-1+m1)-dd2\,m1+t12-\\
m1\,t12-p12\,t12+m1\,p12\,t12+m1\,t22-m1\,p22\,t22)\,(1+\alpha 1))\,/\\
(1+(1+(-1+m1)\,t12-m1\,t22)\,\alpha 1+s1\,(1+(-1+m1)\,t12-m1\,t22)\,(1+\alpha 1))-\\
((1+s1)\,(dd1\,(-1+m1)-dd2\,m1+t12-m1\,t12-p12\,t12+m1\,p12\,t12+\\
m1\,t22-m1\,p22\,t22)\,(-1+dd1\,(-1+m1)-dd2\,m1+p12-m1\,p12+m1\,p22+
\end{array}
$$

```
        t12 - m1 t12 - p12 t12 + m1 p12 t12 + m1 t22 - m1 p22 t22) (1 + α1)) /
    (1 + (1 + (-1 + m1) t12 - m1 t22) α1 + s1 (1 + (-1 + m1) t12 - m1 t22) (1 + α1)) +
   ((dd1 (-1 + m1) - dd2 m1 - p12 t12 + m1 p12 t12 - m1 p22 t22) (dd1 (-1 + m1) -
        dd2 m1 + t12 - m1 t12 - p12 t12 + m1 p12 t12 + m1 t22 - m1 p22 t22) (1 + α2)) /
    (-1 + s1 (-1 + t12 - m1 t12 + m1 t22) + (-1 + m1) t12 α2 - m1 t22 α2) +
   ((-1 + r) (dd1 (-1 + m1) - dd2 m1 + p12 - m1 p12 + m1 p22 - p12 t12 +
        m1 p12 t12 - m1 p22 t22) (dd1 (-1 + m1) - dd2 m1 + t12 -
        m1 t12 - p12 t12 + m1 p12 t12 + m1 t22 - m1 p22 t22) (1 + α2)) /
    (-1 + s1 (-1 + t12 - m1 t12 + m1 t22) + (-1 + m1) t12 α2 - m1 t22 α2) +
   (r (1 + s1) (dd1 (-1 + m1) - dd2 m1 - p12 t12 + m1 p12 t12 - m1 p22 t22)
      (-1 + dd1 (-1 + m1) - dd2 m1 + p12 - m1 p12 + m1 p22 + t12 -
        m1 t12 - p12 t12 + m1 p12 t12 + m1 t22 - m1 p22 t22)) /
    (1 + s1 (1 + (-1 + m1) t12 - m1 t22) + m1 t22 α2 + t12 (α2 - m1 α2)))
  ((r (dd1 (-1 + m1) - dd2 m1 - p12 t12 + m1 p12 t12 - m1 p22 t22)
      (-1 + dd1 (-1 + m1) - dd2 m1 + p12 - m1 p12 + m1 p22 + t12 -
        m1 t12 - p12 t12 + m1 p12 t12 + m1 t22 - m1 p22 t22)) /
    (1 + (1 + (-1 + m1) t12 - m1 t22) α1 + s1 (1 + (-1 + m1) t12 - m1 t22) (1 + α1)) -
   ((-1 + r) (1 + s1) (dd1 (-1 + m1) - dd2 m1 + p12 - m1 p12 + m1 p22 -
        p12 t12 + m1 p12 t12 - m1 p22 t22) (dd1 (-1 + m1) - dd2 m1 + t12 -
        m1 t12 - p12 t12 + m1 p12 t12 + m1 t22 - m1 p22 t22) (1 + α1)) /
    (1 + (1 + (-1 + m1) t12 - m1 t22) α1 + s1 (1 + (-1 + m1) t12 - m1 t22) (1 + α1)) -
   ((1 + s1) (dd1 (-1 + m1) - dd2 m1 + p12 - m1 p12 + m1 p22 - p12 t12 + m1 p12 t12 -
        m1 p22 t22) (-1 + dd1 (-1 + m1) - dd2 m1 + p12 - m1 p12 + m1 p22 +
        t12 - m1 t12 - p12 t12 + m1 p12 t12 + m1 t22 - m1 p22 t22) (1 + α1)) /
    (1 + (1 + (-1 + m1) t12 - m1 t22) α1 + s1 (1 + (-1 + m1) t12 - m1 t22) (1 + α1)) +
   ((dd1 (-1 + m1) - dd2 m1 - p12 t12 + m1 p12 t12 - m1 p22 t22) (dd1 (-1 + m1) -
        dd2 m1 + p12 - m1 p12 + m1 p22 - p12 t12 + m1 p12 t12 - m1 p22 t22) (1 + α2)) /
    (-1 + s1 (-1 + t12 - m1 t12 + m1 t22) + (-1 + m1) t12 α2 - m1 t22 α2) +
   ((-1 + r) (dd1 (-1 + m1) - dd2 m1 + p12 - m1 p12 + m1 p22 - p12 t12 +
        m1 p12 t12 - m1 p22 t22) (dd1 (-1 + m1) - dd2 m1 + t12 -
        m1 t12 - p12 t12 + m1 p12 t12 + m1 t22 - m1 p22 t22) (1 + α2)) /
    (-1 + s1 (-1 + t12 - m1 t12 + m1 t22) + (-1 + m1) t12 α2 - m1 t22 α2) -
   ((1 + s1) (dd1 (-1 + m1) - dd2 m1 - p12 t12 + m1 p12 t12 - m1 p22 t22)
      (dd1 (-1 + m1) - dd2 m1 + p12 - m1 p12 + m1 p22 - p12 t12 + m1 p12 t12 - m1 p22 t22)) /
    (1 + s1 (1 + (-1 + m1) t12 - m1 t22) + m1 t22 α2 + t12 (α2 - m1 α2)) +
   (r (1 + s1) (dd1 (-1 + m1) - dd2 m1 - p12 t12 + m1 p12 t12 - m1 p22 t22)
      (-1 + dd1 (-1 + m1) - dd2 m1 + p12 - m1 p12 + m1 p22 + t12 -
        m1 t12 - p12 t12 + m1 p12 t12 + m1 t22 - m1 p22 t22)) /
    (1 + s1 (1 + (-1 + m1) t12 - m1 t22) + m1 t22 α2 + t12 (α2 - m1 α2)) -
   ((1 + s1) (dd1 (-1 + m1) - dd2 m1 + p12 - m1 p12 + m1 p22 - p12 t12 +
        m1 p12 t12 - m1 p22 t22) (-1 + dd1 (-1 + m1) - dd2 m1 + p12 - m1 p12 +
        m1 p22 + t12 - m1 t12 - p12 t12 + m1 p12 t12 + m1 t22 - m1 p22 t22)) /
    (1 + s1 (1 + (-1 + m1) t12 - m1 t22) + m1 t22 α2 + t12 (α2 - m1 α2)) + (2 (1 + s1)
      (dd1 - dd1 m1 + dd2 m1 - p12 + m1 p12 - m1 p22 + p12 t12 - m1 p12 t12 + m1 p22 t22)^2) /
    (1 + s1 (1 + (-1 + m1) t12 - m1 t22) + m1 t22 α2 + t12 (α2 - m1 α2))) +
 1/4 (- (((-1 + r) (dd1 (-1 + m1) - dd2 m1 - p12 t12 + m1 p12 t12 - m1 p22 t22)
         (-1 + dd1 (-1 + m1) - dd2 m1 + p12 - m1 p12 + m1 p22 + t12 -
           m1 t12 - p12 t12 + m1 p12 t12 + m1 t22 - m1 p22 t22)) /
```

```
      (1 + (1 + (-1 + m1) t12 - m1 t22) α1 + s1 (1 + (-1 + m1) t12 - m1 t22) (1 + α1))) -
    ((dd1 (-1 + m1) - dd2 m1 + t12 - m1 t12 - p12 t12 + m1 p12 t12 + m1 t22 - m1 p22 t22)
      (-1 + dd1 (-1 + m1) - dd2 m1 + p12 - m1 p12 + m1 p22 + t12 -
        m1 t12 - p12 t12 + m1 p12 t12 + m1 t22 - m1 p22 t22)) /
     (1 + (1 + (-1 + m1) t12 - m1 t22) α1 + s1 (1 + (-1 + m1) t12 - m1 t22) (1 + α1)) +
    (r (1 + s1) (dd1 (-1 + m1) - dd2 m1 + p12 - m1 p12 + m1 p22 - p12 t12 +
        m1 p12 t12 - m1 p22 t22) (dd1 (-1 + m1) - dd2 m1 + t12 -
        m1 t12 - p12 t12 + m1 p12 t12 + m1 t22 - m1 p22 t22) (1 + α1)) /
     (1 + (1 + (-1 + m1) t12 - m1 t22) α1 + s1 (1 + (-1 + m1) t12 - m1 t22) (1 + α1)) -
    ((1 + s1) (dd1 (-1 + m1) - dd2 m1 + p12 - m1 p12 + m1 p22 - p12 t12 + m1 p12 t12 -
        m1 p22 t22) (-1 + dd1 (-1 + m1) - dd2 m1 + p12 - m1 p12 + m1 p22 +
        t12 - m1 t12 - p12 t12 + m1 p12 t12 + m1 t22 - m1 p22 t22) (1 + α1)) /
     (1 + (1 + (-1 + m1) t12 - m1 t22) α1 + s1 (1 + (-1 + m1) t12 - m1 t22) (1 + α1)) -
    ((1 + s1) (dd1 (-1 + m1) - dd2 m1 + t12 - m1 t12 - p12 t12 + m1 p12 t12 +
        m1 t22 - m1 p22 t22) (-1 + dd1 (-1 + m1) - dd2 m1 + p12 - m1 p12 + m1 p22 +
        t12 - m1 t12 - p12 t12 + m1 p12 t12 + m1 t22 - m1 p22 t22) (1 + α1)) /
     (1 + (1 + (-1 + m1) t12 - m1 t22) α1 + s1 (1 + (-1 + m1) t12 - m1 t22) (1 + α1)) +
    (2 (1 + s1) (-1 + dd1 (-1 + m1) - dd2 m1 + p12 - m1 p12 + m1 p22 + t12 -
          m1 t12 - p12 t12 + m1 p12 t12 + m1 t22 - m1 p22 t22)^2 (1 + α1)) /
     (1 + (1 + (-1 + m1) t12 - m1 t22) α1 + s1 (1 + (-1 + m1) t12 - m1 t22) (1 + α1)) -
    ((-1 + r) (1 + s1) (dd1 (-1 + m1) - dd2 m1 - p12 t12 + m1 p12 t12 - m1 p22 t22)
      (-1 + dd1 (-1 + m1) - dd2 m1 + p12 - m1 p12 + m1 p22 + t12 -
        m1 t12 - p12 t12 + m1 p12 t12 + m1 t22 - m1 p22 t22)) /
     (1 + s1 (1 + (-1 + m1) t12 - m1 t22) + m1 t22 α2 + t12 (α2 - m1 α2)) -
    ((1 + s1) (dd1 (-1 + m1) - dd2 m1 + p12 - m1 p12 + m1 p22 - p12 t12 +
        m1 p12 t12 - m1 p22 t22) (-1 + dd1 (-1 + m1) - dd2 m1 + p12 - m1 p12 +
        m1 p22 + t12 - m1 t12 - p12 t12 + m1 p12 t12 + m1 t22 - m1 p22 t22)) /
     (1 + s1 (1 + (-1 + m1) t12 - m1 t22) + m1 t22 α2 + t12 (α2 - m1 α2)) +
    (r (dd1 (-1 + m1) - dd2 m1 + p12 - m1 p12 + m1 p22 - p12 t12 + m1 p12 t12 - m1 p22 t22)
      (dd1 (-1 + m1) - dd2 m1 + t12 - m1 t12 - p12 t12 + m1 p12 t12 + m1 t22 - m1 p22 t22)
      (1 + α2)) / (1 + s1 (1 + (-1 + m1) t12 - m1 t22) + m1 t22 α2 + t12 (α2 - m1 α2)))
  (- (((dd1 (-1 + m1) - dd2 m1 - p12 t12 + m1 p12 t12 - m1 p22 t22) (dd1 (-1 + m1) -
          dd2 m1 + t12 - m1 t12 - p12 t12 + m1 p12 t12 + m1 t22 - m1 p22 t22)) /
      (1 + (1 + (-1 + m1) t12 - m1 t22) α1 + s1 (1 + (-1 + m1) t12 - m1 t22) (1 + α1))) -
    ((-1 + r) (dd1 (-1 + m1) - dd2 m1 - p12 t12 + m1 p12 t12 - m1 p22 t22)
      (-1 + dd1 (-1 + m1) - dd2 m1 + p12 - m1 p12 + m1 p22 + t12 -
        m1 t12 - p12 t12 + m1 p12 t12 + m1 t22 - m1 p22 t22)) /
     (1 + (1 + (-1 + m1) t12 - m1 t22) α1 + s1 (1 + (-1 + m1) t12 - m1 t22) (1 + α1)) +
    (r (1 + s1) (dd1 (-1 + m1) - dd2 m1 + p12 - m1 p12 + m1 p22 - p12 t12 +
        m1 p12 t12 - m1 p22 t22) (dd1 (-1 + m1) - dd2 m1 + t12 -
        m1 t12 - p12 t12 + m1 p12 t12 + m1 t22 - m1 p22 t22) (1 + α1)) /
     (1 + (1 + (-1 + m1) t12 - m1 t22) α1 + s1 (1 + (-1 + m1) t12 - m1 t22) (1 + α1)) +
    ((dd1 (-1 + m1) - dd2 m1 - p12 t12 + m1 p12 t12 - m1 p22 t22) (dd1 (-1 + m1) -
        dd2 m1 + p12 - m1 p12 + m1 p22 - p12 t12 + m1 p12 t12 - m1 p22 t22) (1 + α2)) /
     (-1 + s1 (-1 + t12 - m1 t12 + m1 t22) + (-1 + m1) t12 α2 - m1 t22 α2) +
    ((dd1 (-1 + m1) - dd2 m1 - p12 t12 + m1 p12 t12 - m1 p22 t22) (dd1 (-1 + m1) -
        dd2 m1 + t12 - m1 t12 - p12 t12 + m1 p12 t12 + m1 t22 - m1 p22 t22) (1 + α2)) /
     (-1 + s1 (-1 + t12 - m1 t12 + m1 t22) + (-1 + m1) t12 α2 - m1 t22 α2) -
    ((1 + s1) (dd1 (-1 + m1) - dd2 m1 - p12 t12 + m1 p12 t12 - m1 p22 t22)
```

$$
\begin{aligned}
&(dd1\ (-1 + m1) - dd2\ m1 + p12 - m1\ p12 + m1\ p22 - p12\ t12 + m1\ p12\ t12 - m1\ p22\ t22))\ / \\
&(1 + s1\ (1 + (-1 + m1)\ t12 - m1\ t22) + m1\ t22\ \alpha2 + t12\ (\alpha2 - m1\ \alpha2)) - \\
&((-1 + r)\ (1 + s1)\ (dd1\ (-1 + m1) - dd2\ m1 - p12\ t12 + m1\ p12\ t12 - m1\ p22\ t22) \\
&(-1 + dd1\ (-1 + m1) - dd2\ m1 + p12 - m1\ p12 + m1\ p22 + t12 - \\
&m1\ t12 - p12\ t12 + m1\ p12\ t12 + m1\ t22 - m1\ p22\ t22))\ / \\
&(1 + s1\ (1 + (-1 + m1)\ t12 - m1\ t22) + m1\ t22\ \alpha2 + t12\ (\alpha2 - m1\ \alpha2)) + \\
&(r\ (dd1\ (-1 + m1) - dd2\ m1 + p12 - m1\ p12 + m1\ p22 - p12\ t12 + m1\ p12\ t12 - m1\ p22\ t22) \\
&(dd1\ (-1 + m1) - dd2\ m1 + t12 - m1\ t12 - p12\ t12 + m1\ p12\ t12 + m1\ t22 - m1\ p22\ t22) \\
&(1 + \alpha2))\ /\ (1 + s1\ (1 + (-1 + m1)\ t12 - m1\ t22) + m1\ t22\ \alpha2 + t12\ (\alpha2 - m1\ \alpha2)) + \\
&(2\ (dd1 - dd1\ m1 + dd2\ m1 + p12\ t12 - m1\ p12\ t12 + m1\ p22\ t22)^2\ (1 + \alpha2))\ / \\
&(1 + s1\ (1 + (-1 + m1)\ t12 - m1\ t22) + m1\ t22\ \alpha2 + t12\ (\alpha2 - m1\ \alpha2))), \\
&(dd2\ (s2 - \alpha1)\ (1 + t22\ \alpha2 + s2\ t22\ (1 + \alpha2)) - m2^3\ (p12 - p22)\ (t12 - t22) \\
&(p12\ \alpha1 - p22\ \alpha1 + p12\ \alpha2 - p22\ \alpha2 + p12\ \alpha1\ \alpha2 - p22\ \alpha1\ \alpha2 + \\
&t12\ \alpha1\ \alpha2 - t22\ \alpha1\ \alpha2 - s2^2\ (t12 - t22)\ (1 + \alpha2) + \\
&s2\ ((t12 - t22)\ (\alpha1 - \alpha2 + \alpha1\ \alpha2) + p12\ (\alpha1 + \alpha2 + \alpha1\ \alpha2) - p22\ (\alpha1 + \alpha2 + \alpha1\ \alpha2))) + \\
&p22\ (2 + dd2\ \alpha2 + 2\ t22\ \alpha2 + 2\ s2^2\ t22^2\ (1 + \alpha2) + \\
&\alpha1\ (dd2\ (1 + \alpha2) - 2\ (-1 + t22)\ (1 + t22\ \alpha2)) + \\
&s2\ (-2\ t22^2\ (\alpha1 - \alpha2 + \alpha1\ \alpha2) + dd2\ (\alpha1 + \alpha2 + \alpha1\ \alpha2) + 2\ t22\ (2 + \alpha1 + \alpha2 + \alpha1\ \alpha2))) + \\
&m2^2\ ((dd1 - dd2)\ (t12 - t22)\ (s2 - \alpha1)\ (s2 + \alpha2 + s2\ \alpha2) + p12^2\ (1 + s2) \\
&(t12 - t22)\ (\alpha1 + \alpha2 + \alpha1\ \alpha2) + 2\ p22^2\ (1 + s2)\ (t12 - t22)\ (\alpha1 + \alpha2 + \alpha1\ \alpha2) + \\
&p12\ (dd1\ \alpha1 - dd2\ \alpha1 - t12\ \alpha1 - 3\ p22\ t12\ \alpha1 + t22\ \alpha1 + 3\ p22\ t22\ \alpha1 + dd1\ \alpha2 - \\
&dd2\ \alpha2 + 2\ t12\ \alpha2 - 3\ p22\ t12\ \alpha2 - 2\ t22\ \alpha2 + 3\ p22\ t22\ \alpha2 + dd1\ \alpha1\ \alpha2 - dd2\ \alpha1\ \alpha2 + \\
&2\ t12\ \alpha1\ \alpha2 - 3\ p22\ t12\ \alpha1\ \alpha2 - t12^2\ \alpha1\ \alpha2 - 2\ t22\ \alpha1\ \alpha2 + 3\ p22\ t22\ \alpha1\ \alpha2 - \\
&t12\ t22\ \alpha1\ \alpha2 + 2\ t22^2\ \alpha1\ \alpha2 + s2^2\ (t12^2 + t12\ t22 - 2\ t22^2)\ (1 + \alpha2) - \\
&s2\ (t12^2\ (\alpha1 - \alpha2 + \alpha1\ \alpha2) - 2\ t22^2\ (\alpha1 - \alpha2 + \alpha1\ \alpha2) - (dd1 - dd2) \\
&(\alpha1 + \alpha2 + \alpha1\ \alpha2) + t22\ (3 + (2 - 3\ p22)\ \alpha2 - (-2 + 3\ p22)\ \alpha1\ (1 + \alpha2)) + t12 \\
&(-3 + (-2 + 3\ p22 - t22)\ \alpha2 + (-2 + 3\ p22 + t22)\ \alpha1\ (1 + \alpha2)))) + \\
&p22\ (-dd1\ \alpha1 + dd2\ \alpha1 + t12\ \alpha1 - t22\ \alpha1 - dd1\ \alpha2 + dd2\ \alpha2 - 2\ t12\ \alpha2 + \\
&2\ t22\ \alpha2 - dd1\ \alpha1\ \alpha2 + dd2\ \alpha1\ \alpha2 - 2\ t12\ \alpha1\ \alpha2 - t12^2\ \alpha1\ \alpha2 + 2\ t22\ \alpha1\ \alpha2 + \\
&5\ t12\ t22\ \alpha1\ \alpha2 - 4\ t22^2\ \alpha1\ \alpha2 + s2^2\ (t12^2 - 5\ t12\ t22 + 4\ t22^2)\ (1 + \alpha2) - \\
&s2\ (t12^2\ (\alpha1 - \alpha2 + \alpha1\ \alpha2) + 4\ t22^2\ (\alpha1 - \alpha2 + \alpha1\ \alpha2) + (dd1 - dd2) \\
&(\alpha1 + \alpha2 + \alpha1\ \alpha2) - t22\ (3 + 2\ \alpha2 + 2\ \alpha1\ (1 + \alpha2)) + t12 \\
&(3 + 2\ \alpha2 + 5\ t22\ \alpha2 - (-2 + 5\ t22)\ \alpha1\ (1 + \alpha2))))) + \\
&m2\ (-p22^2\ (1 + s2)\ (t12 - t22)\ (\alpha1 + \alpha2 + \alpha1\ \alpha2) + (s2 - \alpha1)\ (dd2\ (-1 + t12\ \alpha2 - \\
&2\ t22\ \alpha2 + s2\ (t12 - 2\ t22)\ (1 + \alpha2)) + dd1\ (1 + t22\ \alpha2 + s2\ t22\ (1 + \alpha2))) + \\
&p22\ (-2 - 2\ \alpha1 + dd1\ \alpha1 - 2\ dd2\ \alpha1 - t12\ \alpha1 + 3\ t22\ \alpha1 + dd1\ \alpha2 - 2\ dd2\ \alpha2 + \\
&2\ t12\ \alpha2 - 4\ t22\ \alpha2 + dd1\ \alpha1\ \alpha2 - 2\ dd2\ \alpha1\ \alpha2 + 2\ t12\ \alpha1\ \alpha2 - 4\ t22\ \alpha1\ \alpha2 - \\
&3\ t12\ t22\ \alpha1\ \alpha2 + 5\ t22^2\ \alpha1\ \alpha2 + s2^2\ (3\ t12 - 5\ t22)\ t22\ (1 + \alpha2) + \\
&s2\ (5\ t22^2\ (\alpha1 - \alpha2 + \alpha1\ \alpha2) + (dd1 - 2\ dd2)\ (\alpha1 + \alpha2 + \alpha1\ \alpha2) - t22\ (7 + 4\ \alpha2 + \\
&4\ \alpha1\ (1 + \alpha2)) + t12\ (3 + 2\ \alpha2 + 3\ t22\ \alpha2 - (-2 + 3\ t22)\ \alpha1\ (1 + \alpha2)))) + \\
&p12\ (2 + 2\ \alpha1 + dd2\ \alpha1 - t12\ \alpha1 + p22\ t12\ \alpha1 - t22\ \alpha1 - p22\ t22\ \alpha1 + dd2\ \alpha2 + \\
&p22\ t12\ \alpha2 + 2\ t22\ \alpha2 - p22\ t22\ \alpha2 + dd2\ \alpha1\ \alpha2 + p22\ t12\ \alpha1\ \alpha2 + \\
&2\ t22\ \alpha1\ \alpha2 - p22\ t22\ \alpha1\ \alpha2 - t12\ t22\ \alpha1\ \alpha2 - t22^2\ \alpha1\ \alpha2 + \\
&s2^2\ t22\ (t12 + t22)\ (1 + \alpha2) + s2\ (-t22^2\ (\alpha1 - \alpha2 + \alpha1\ \alpha2) + dd2 \\
&(\alpha1 + \alpha2 + \alpha1\ \alpha2) - t22\ (-3 + (-2 + p22)\ \alpha2 + (-2 + p22)\ \alpha1\ (1 + \alpha2)) + \\
&t12\ (1 - t22\ (\alpha1 - \alpha2 + \alpha1\ \alpha2) + p22\ (\alpha1 + \alpha2 + \alpha1\ \alpha2))))))\ / \\
&(2\ (1 + s2\ t22 + m2\ (t12 - t22)\ (s2 - \alpha1) + \alpha1 - t22\ \alpha1)\ (1 + t22\ \alpha2 + \\
&s2\ t22\ (1 + \alpha2) + m2\ (t12 - t22)\ (s2 + \alpha2 + s2\ \alpha2))), \\
&((m2\ (t12 - t22) + t22)\ (2 + m2\ p12\ \alpha2 + p22\ \alpha2 - m2\ p22\ \alpha2 + 2\ m2\ t12\ \alpha2 - \\
&m2^2\ p12\ t12\ \alpha2 - m2\ p22\ t12\ \alpha2 + m2^2\ p22\ t12\ \alpha2 +
\end{aligned}
$$

$$
\begin{aligned}
&2\,t22\,\alpha2 - 2\,m2\,t22\,\alpha2 - m2\,p12\,t22\,\alpha2 + m2^2\,p12\,t22\,\alpha2 - \\
&p22\,t22\,\alpha2 + 2\,m2\,p22\,t22\,\alpha2 - m2^2\,p22\,t22\,\alpha2 + \\
&s2^2\left(m2^2\,(t12 - t22)^2 + t22\,(1 + t22) + m2\,(t12 - t22)\,(1 + 2\,t22)\right)(1 + \alpha2) - \\
&(-1 + m2\,(t12 - t22) + t22)\,\alpha1 \\
&\quad(1 + p22 + p22\,\alpha2 + t22\,\alpha2 + m2\,((t12 - t22)\,\alpha2 + p12\,(1 + \alpha2) - p22\,(1 + \alpha2))) - \\
&s2\,(-1 - 3\,t22 - p22\,\alpha1 - t22\,\alpha1 + p22\,t22\,\alpha1 + t22^2\,\alpha1 - p22\,\alpha2 - 3\,t22\,\alpha2 + p22\,t22\,\alpha2 - \\
&\quad t22^2\,\alpha2 - p22\,\alpha1\,\alpha2 - t22\,\alpha1\,\alpha2 + p22\,t22\,\alpha1\,\alpha2 + t22^2\,\alpha1\,\alpha2 + m2^2\,(t12 - t22) \\
&\qquad((t12 - t22)\,(\alpha1 - \alpha2 + \alpha1\,\alpha2) + p12\,(\alpha1 + \alpha2 + \alpha1\,\alpha2) - p22\,(\alpha1 + \alpha2 + \alpha1\,\alpha2)) + \\
&\quad m2\,(-2\,t22^2\,(\alpha1 - \alpha2 + \alpha1\,\alpha2) - (p12 - p22)\,(\alpha1 + \alpha2 + \alpha1\,\alpha2) + \\
&\qquad t22\,(3 + (3 + p12 - 2\,p22)\,\alpha2 + (1 + p12 - 2\,p22)\,\alpha1\,(1 + \alpha2)) + \\
&\qquad t12\,(-3 + (-3 + p22 - 2\,t22)\,\alpha2 + (-1 + p22 + 2\,t22)\,\alpha1\,(1 + \alpha2)))))\Big) \Big/ \\
&(2\,(1 + s2\,t22 + m2\,(t12 - t22)\,(s2 - \alpha1) + \alpha1 - t22\,\alpha1)\,(1 + t22\,\alpha2 + \\
&\quad s2\,t22\,(1 + \alpha2) + m2\,(t12 - t22)\,(s2 + \alpha2 + s2\,\alpha2))), \\
&-\frac{1}{4}\,((r\,(1 + s2)\,(dd2 + dd1\,m2 - dd2\,m2 + m2\,p12\,t12 + p22\,t22 - m2\,p22\,t22) \\
&\quad(1 + dd2 + dd1\,m2 - dd2\,m2 - m2\,p12 - p22 + m2\,p22 - m2\,t12 + \\
&\qquad m2\,p12\,t12 - t22 + m2\,t22 + p22\,t22 - m2\,p22\,t22)) / \\
&\quad(1 + s2\,t22 + m2\,(t12 - t22)\,(s2 - \alpha1) + \alpha1 - t22\,\alpha1) - ((-1 + r) \\
&\qquad(dd2\,(-1 + m2) - dd1\,m2 + m2\,p12 + p22 - m2\,p22 - m2\,p12\,t12 - p22\,t22 + m2\,p22\,t22) \\
&\qquad(dd2\,(-1 + m2) - dd1\,m2 + m2\,t12 - m2\,p12\,t12 + t22 - m2\,t22 - p22\,t22 + m2\,p22\,t22) \\
&\qquad(1 + \alpha1)) / (1 + s2\,t22 + m2\,(t12 - t22)\,(s2 - \alpha1) + \alpha1 - t22\,\alpha1) - \\
&\quad((dd2\,(-1 + m2) - dd1\,m2 + m2\,p12 + p22 - m2\,p22 - m2\,p12\,t12 - p22\,t22 + m2\,p22\,t22) \\
&\qquad(-1 + dd2\,(-1 + m2) - dd1\,m2 + m2\,p12 + p22 - m2\,p22 + m2\,t12 - \\
&\qquad m2\,p12\,t12 + t22 - m2\,t22 - p22\,t22 + m2\,p22\,t22)\,(1 + \alpha1)) / \\
&\quad(1 + s2\,t22 + m2\,(t12 - t22)\,(s2 - \alpha1) + \alpha1 - t22\,\alpha1) + \\
&\quad\left(2\,(dd2 + dd1\,m2 - dd2\,m2 - m2\,p12 - p22 + m2\,p22 + m2\,p12\,t12 + p22\,t22 - m2\,p22\,t22)^2\right) \Big/ \\
&\quad(1 + t22\,\alpha2 + s2\,t22\,(1 + \alpha2) + m2\,(t12 - t22)\,(s2 + \alpha2 + s2\,\alpha2)) - \\
&\quad((dd2\,(-1 + m2) - dd1\,m2 - m2\,p12\,t12 - p22\,t22 + m2\,p22\,t22) \\
&\qquad(dd2\,(-1 + m2) - dd1\,m2 + m2\,p12 + p22 - m2\,p22 - m2\,p12\,t12 - p22\,t22 + m2\,p22\,t22)) / \\
&\quad(1 + t22\,\alpha2 + s2\,t22\,(1 + \alpha2) + m2\,(t12 - t22)\,(s2 + \alpha2 + s2\,\alpha2)) + \\
&\quad(r\,(dd2\,(-1 + m2) - dd1\,m2 - m2\,p12\,t12 - p22\,t22 + m2\,p22\,t22) \\
&\qquad(-1 + dd2\,(-1 + m2) - dd1\,m2 + m2\,p12 + p22 - m2\,p22 + \\
&\qquad m2\,t12 - m2\,p12\,t12 + t22 - m2\,t22 - p22\,t22 + m2\,p22\,t22)) / \\
&\quad(1 + t22\,\alpha2 + s2\,t22\,(1 + \alpha2) + m2\,(t12 - t22)\,(s2 + \alpha2 + s2\,\alpha2)) - \\
&\quad((dd2\,(-1 + m2) - dd1\,m2 + m2\,p12 + p22 - m2\,p22 - m2\,p12\,t12 - p22\,t22 + m2\,p22\,t22) \\
&\qquad(-1 + dd2\,(-1 + m2) - dd1\,m2 + m2\,p12 + p22 - m2\,p22 + \\
&\qquad m2\,t12 - m2\,p12\,t12 + t22 - m2\,t22 - p22\,t22 + m2\,p22\,t22)) / \\
&\quad(1 + t22\,\alpha2 + s2\,t22\,(1 + \alpha2) + m2\,(t12 - t22)\,(s2 + \alpha2 + s2\,\alpha2)) - \\
&\quad((1 + s2)\,(dd2 + dd1\,m2 - dd2\,m2 + m2\,p12\,t12 + p22\,t22 - m2\,p22\,t22) \\
&\qquad(dd2 + dd1\,m2 - dd2\,m2 - m2\,p12 - p22 + m2\,p22 + m2\,p12\,t12 + p22\,t22 - m2\,p22\,t22) \\
&\qquad(1 + \alpha2)) / (1 + t22\,\alpha2 + s2\,t22\,(1 + \alpha2) + m2\,(t12 - t22)\,(s2 + \alpha2 + s2\,\alpha2)) - \\
&\quad((-1 + r)\,(1 + s2)\,(dd2 + dd1\,m2 - dd2\,m2 - m2\,p12 - p22 + m2\,p22 + m2\,p12\,t12 + \\
&\qquad p22\,t22 - m2\,p22\,t22)\,(dd2 + dd1\,m2 - dd2\,m2 - m2\,t12 + \\
&\qquad m2\,p12\,t12 - t22 + m2\,t22 + p22\,t22 - m2\,p22\,t22)\,(1 + \alpha2)) / \\
&\quad(1 + t22\,\alpha2 + s2\,t22\,(1 + \alpha2) + m2\,(t12 - t22)\,(s2 + \alpha2 + s2\,\alpha2))) \\
&\Big(-(((1 + s2)\,(dd2 + dd1\,m2 - dd2\,m2 + m2\,p12\,t12 + p22\,t22 - m2\,p22\,t22)\,(dd2 + \\
&\qquad dd1\,m2 - dd2\,m2 - m2\,t12 + m2\,p12\,t12 - t22 + m2\,t22 + p22\,t22 - m2\,p22\,t22)) / \\
&\qquad(1 + s2\,t22 + m2\,(t12 - t22)\,(s2 - \alpha1) + \alpha1 - t22\,\alpha1)) + \big(2\,(1 + s2)
\end{aligned}
$$

$$(dd2 + dd1\, m2 - dd2\, m2 - m2\, t12 + m2\, p12\, t12 - t22 + m2\, t22 + p22\, t22 - m2\, p22\, t22)^{2}) /$$
$$(1 + s2\, t22 + m2\, (t12 - t22)\, (s2 - \alpha 1) + \alpha 1 - t22\, \alpha 1) +$$
$$(r\, (1 + s2)\, (dd2 + dd1\, m2 - dd2\, m2 + m2\, p12\, t12 + p22\, t22 - m2\, p22\, t22)$$
$$(1 + dd2 + dd1\, m2 - dd2\, m2 - m2\, p12 - p22 + m2\, p22 - m2\, t12 + m2\, p12\, t12 - t22 + m2\, t22 +$$
$$p22\, t22 - m2\, p22\, t22)) / (1 + s2\, t22 + m2\, (t12 - t22)\, (s2 - \alpha 1) + \alpha 1 - t22\, \alpha 1) -$$
$$((1 + s2)\, (dd2\, (-1 + m2) - dd1\, m2 + m2\, t12 - m2\, p12\, t12 + t22 - m2\, t22 -$$
$$p22\, t22 + m2\, p22\, t22)\, (-1 + dd2\, (-1 + m2) - dd1\, m2 + m2\, p12 + p22 -$$
$$m2\, p22 + m2\, t12 - m2\, p12\, t12 + t22 - m2\, t22 - p22\, t22 + m2\, p22\, t22)) /$$
$$(1 + s2\, t22 + m2\, (t12 - t22)\, (s2 - \alpha 1) + \alpha 1 - t22\, \alpha 1) - ((-1 + r)$$
$$(dd2\, (-1 + m2) - dd1\, m2 + m2\, p12 + p22 - m2\, p22 - m2\, p12\, t12 - p22\, t22 + m2\, p22\, t22)$$
$$(dd2\, (-1 + m2) - dd1\, m2 + m2\, t12 - m2\, p12\, t12 + t22 - m2\, t22 - p22\, t22 + m2\, p22\, t22)$$
$$(1 + \alpha 1)) / (1 + s2\, t22 + m2\, (t12 - t22)\, (s2 - \alpha 1) + \alpha 1 - t22\, \alpha 1) -$$
$$((dd2\, (-1 + m2) - dd1\, m2 + m2\, t12 - m2\, p12\, t12 + t22 - m2\, t22 - p22\, t22 + m2\, p22\, t22)$$
$$(-1 + dd2\, (-1 + m2) - dd1\, m2 + m2\, p12 + p22 - m2\, p22 + m2\, t12 -$$
$$m2\, p12\, t12 + t22 - m2\, t22 - p22\, t22 + m2\, p22\, t22)\, (1 + \alpha 1)) /$$
$$(1 + s2\, t22 + m2\, (t12 - t22)\, (s2 - \alpha 1) + \alpha 1 - t22\, \alpha 1) +$$
$$(r\, (dd2\, (-1 + m2) - dd1\, m2 - m2\, p12\, t12 - p22\, t22 + m2\, p22\, t22)$$
$$(-1 + dd2\, (-1 + m2) - dd1\, m2 + m2\, p12 + p22 - m2\, p22 +$$
$$m2\, t12 - m2\, p12\, t12 + t22 - m2\, t22 - p22\, t22 + m2\, p22\, t22)) /$$
$$(1 + t22\, \alpha 2 + s2\, t22\, (1 + \alpha 2) + m2\, (t12 - t22)\, (s2 + \alpha 2 + s2\, \alpha 2)) -$$
$$((1 + s2)\, (dd2 + dd1\, m2 - dd2\, m2 + m2\, p12\, t12 + p22\, t22 - m2\, p22\, t22)$$
$$(dd2 + dd1\, m2 - dd2\, m2 - m2\, t12 + m2\, p12\, t12 - t22 + m2\, t22 + p22\, t22 - m2\, p22\, t22)$$
$$(1 + \alpha 2)) / (1 + t22\, \alpha 2 + s2\, t22\, (1 + \alpha 2) + m2\, (t12 - t22)\, (s2 + \alpha 2 + s2\, \alpha 2)) -$$
$$((-1 + r)\, (1 + s2)\, (dd2 + dd1\, m2 - dd2\, m2 - m2\, p12 - p22 + m2\, p22 + m2\, p12\, t12 +$$
$$p22\, t22 - m2\, p22\, t22)\, (dd2 + dd1\, m2 - dd2\, m2 - m2\, t12 +$$
$$m2\, p12\, t12 - t22 + m2\, t22 + p22\, t22 - m2\, p22\, t22)\, (1 + \alpha 2)) /$$
$$(1 + t22\, \alpha 2 + s2\, t22\, (1 + \alpha 2) + m2\, (t12 - t22)\, (s2 + \alpha 2 + s2\, \alpha 2))) +$$
$$\frac{1}{4}\, (-(((-1 + r)\, (1 + s2)\, (dd2 + dd1\, m2 - dd2\, m2 + m2\, p12\, t12 + p22\, t22 - m2\, p22\, t22)$$
$$(1 + dd2 + dd1\, m2 - dd2\, m2 - m2\, p12 - p22 + m2\, p22 - m2\, t12 +$$
$$m2\, p12\, t12 - t22 + m2\, t22 + p22\, t22 - m2\, p22\, t22)) /$$
$$(1 + s2\, t22 + m2\, (t12 - t22)\, (s2 - \alpha 1) + \alpha 1 - t22\, \alpha 1)) -$$
$$((1 + s2)\, (dd2\, (-1 + m2) - dd1\, m2 + m2\, t12 - m2\, p12\, t12 + t22 - m2\, t22 -$$
$$p22\, t22 + m2\, p22\, t22)\, (-1 + dd2\, (-1 + m2) - dd1\, m2 + m2\, p12 + p22 -$$
$$m2\, p22 + m2\, t12 - m2\, p12\, t12 + t22 - m2\, t22 - p22\, t22 + m2\, p22\, t22)) /$$
$$(1 + s2\, t22 + m2\, (t12 - t22)\, (s2 - \alpha 1) + \alpha 1 - t22\, \alpha 1) +$$
$$(r\, (dd2\, (-1 + m2) - dd1\, m2 + m2\, p12 + p22 - m2\, p22 - m2\, p12\, t12 - p22\, t22 + m2\, p22\, t22)$$
$$(dd2\, (-1 + m2) - dd1\, m2 + m2\, t12 - m2\, p12\, t12 + t22 - m2\, t22 - p22\, t22 + m2\, p22\, t22)$$
$$(1 + \alpha 1)) / (1 + s2\, t22 + m2\, (t12 - t22)\, (s2 - \alpha 1) + \alpha 1 - t22\, \alpha 1) -$$
$$((dd2\, (-1 + m2) - dd1\, m2 + m2\, p12 + p22 - m2\, p22 - m2\, p12\, t12 - p22\, t22 + m2\, p22\, t22)$$
$$(-1 + dd2\, (-1 + m2) - dd1\, m2 + m2\, p12 + p22 - m2\, p22 + m2\, t12 -$$
$$m2\, p12\, t12 + t22 - m2\, t22 - p22\, t22 + m2\, p22\, t22)\, (1 + \alpha 1)) /$$
$$(1 + s2\, t22 + m2\, (t12 - t22)\, (s2 - \alpha 1) + \alpha 1 - t22\, \alpha 1) -$$
$$((dd2\, (-1 + m2) - dd1\, m2 + m2\, t12 - m2\, p12\, t12 + t22 - m2\, t22 - p22\, t22 + m2\, p22\, t22)$$
$$(-1 + dd2\, (-1 + m2) - dd1\, m2 + m2\, p12 + p22 - m2\, p22 + m2\, t12 -$$
$$m2\, p12\, t12 + t22 - m2\, t22 - p22\, t22 + m2\, p22\, t22)\, (1 + \alpha 1)) /$$
$$(1 + s2\, t22 + m2\, (t12 - t22)\, (s2 - \alpha 1) + \alpha 1 - t22\, \alpha 1) +$$
$$(2\, (-1 + dd2\, (-1 + m2) - dd1\, m2 + m2\, p12 + p22 - m2\, p22 + m2\, t12 -$$
$$m2\, p12\, t12 + t22 - m2\, t22 - p22\, t22 + m2\, p22\, t22)^{2}\, (1 + \alpha 1)) /$$

```
        (1 + s2 t22 + m2 (t12 - t22) (s2 - α1) + α1 - t22 α1) -
      ((-1 + r) (dd2 (-1 + m2) - dd1 m2 - m2 p12 t12 - p22 t22 + m2 p22 t22)
          (-1 + dd2 (-1 + m2) - dd1 m2 + m2 p12 + p22 - m2 p22 +
            m2 t12 - m2 p12 t12 + t22 - m2 t22 - p22 t22 + m2 p22 t22)) /
        (1 + t22 α2 + s2 t22 (1 + α2) + m2 (t12 - t22) (s2 + α2 + s2 α2)) -
      ((dd2 (-1 + m2) - dd1 m2 + m2 p12 + p22 - m2 p22 - m2 p12 t12 - p22 t22 + m2 p22 t22)
          (-1 + dd2 (-1 + m2) - dd1 m2 + m2 p12 + p22 - m2 p22 +
            m2 t12 - m2 p12 t12 + t22 - m2 t22 - p22 t22 + m2 p22 t22)) /
        (1 + t22 α2 + s2 t22 (1 + α2) + m2 (t12 - t22) (s2 + α2 + s2 α2)) +
      (r (1 + s2) (dd2 + dd1 m2 - dd2 m2 - m2 p12 - p22 + m2 p22 + m2 p12 t12 +
            p22 t22 - m2 p22 t22) (dd2 + dd1 m2 - dd2 m2 - m2 t12 +
            m2 p12 t12 - t22 + m2 t22 + p22 t22 - m2 p22 t22) (1 + α2)) /
        (1 + t22 α2 + s2 t22 (1 + α2) + m2 (t12 - t22) (s2 + α2 + s2 α2)))
    (- (((1 + s2) (dd2 + dd1 m2 - dd2 m2 + m2 p12 t12 + p22 t22 - m2 p22 t22) (dd2 +
              dd1 m2 - dd2 m2 - m2 t12 + m2 p12 t12 - t22 + m2 t22 + p22 t22 - m2 p22 t22)) /
          (1 + s2 t22 + m2 (t12 - t22) (s2 - α1) + α1 - t22 α1)) -
      ((-1 + r) (1 + s2) (dd2 + dd1 m2 - dd2 m2 + m2 p12 t12 + p22 t22 - m2 p22 t22)
          (1 + dd2 + dd1 m2 - dd2 m2 - m2 p12 - p22 + m2 p22 - m2 t12 + m2 p12 t12 - t22 + m2 t22 +
            p22 t22 - m2 p22 t22)) / (1 + s2 t22 + m2 (t12 - t22) (s2 - α1) + α1 - t22 α1) +
      (r (dd2 (-1 + m2) - dd1 m2 + m2 p12 + p22 - m2 p22 - m2 p12 t12 - p22 t22 + m2 p22 t22)
          (dd2 (-1 + m2) - dd1 m2 + m2 t12 - m2 p12 t12 + t22 - m2 t22 - p22 t22 + m2 p22 t22)
          (1 + α1)) / (1 + s2 t22 + m2 (t12 - t22) (s2 - α1) + α1 - t22 α1) -
      ((dd2 (-1 + m2) - dd1 m2 - m2 p12 t12 - p22 t22 + m2 p22 t22)
          (dd2 (-1 + m2) - dd1 m2 + m2 p12 + p22 - m2 p22 - m2 p12 t12 - p22 t22 + m2 p22 t22)) /
        (1 + t22 α2 + s2 t22 (1 + α2) + m2 (t12 - t22) (s2 + α2 + s2 α2)) -
      ((-1 + r) (dd2 (-1 + m2) - dd1 m2 - m2 p12 t12 - p22 t22 + m2 p22 t22)
          (-1 + dd2 (-1 + m2) - dd1 m2 + m2 p12 + p22 - m2 p22 +
            m2 t12 - m2 p12 t12 + t22 - m2 t22 - p22 t22 + m2 p22 t22)) /
        (1 + t22 α2 + s2 t22 (1 + α2) + m2 (t12 - t22) (s2 + α2 + s2 α2)) +
      (2 (1 + s2) (dd2 + dd1 m2 - dd2 m2 + m2 p12 t12 + p22 t22 - m2 p22 t22)^2 (1 + α2)) /
        (1 + t22 α2 + s2 t22 (1 + α2) + m2 (t12 - t22) (s2 + α2 + s2 α2)) -
      ((1 + s2) (dd2 + dd1 m2 - dd2 m2 + m2 p12 t12 + p22 t22 - m2 p22 t22)
          (dd2 + dd1 m2 - dd2 m2 - m2 p12 - p22 + m2 p22 + m2 p12 t12 + p22 t22 - m2 p22 t22)
          (1 + α2)) / (1 + t22 α2 + s2 t22 (1 + α2) + m2 (t12 - t22) (s2 + α2 + s2 α2)) -
      ((1 + s2) (dd2 + dd1 m2 - dd2 m2 + m2 p12 t12 + p22 t22 - m2 p22 t22)
          (dd2 + dd1 m2 - dd2 m2 - m2 t12 + m2 p12 t12 - t22 + m2 t22 + p22 t22 - m2 p22 t22)
          (1 + α2)) / (1 + t22 α2 + s2 t22 (1 + α2) + m2 (t12 - t22) (s2 + α2 + s2 α2)) +
      (r (1 + s2) (dd2 + dd1 m2 - dd2 m2 - m2 p12 - p22 + m2 p22 + m2 p12 t12 +
            p22 t22 - m2 p22 t22) (dd2 + dd1 m2 - dd2 m2 - m2 t12 +
            m2 p12 t12 - t22 + m2 t22 + p22 t22 - m2 p22 t22) (1 + α2)) /
        (1 + t22 α2 + s2 t22 (1 + α2) + m2 (t12 - t22) (s2 + α2 + s2 α2)))};
```

```
itgenmale[.1, .2, 3, 3.5, .1, .1, .5][{.8, .5, .05, .6, .9, .04}]
```

```
{0.792781, 0.615953, 0.0469744, 0.618342, 0.854094, 0.037247}
```

2. Analysis

2.1 Equilibria with fixation of one allele

2.1.1 Determination of equilibria

Assume dd1 = dd2 = 0 and p12 = p22

We show that the only possible equilibria are t12 = t22 = 0 and t12 = t22 = 1 (Determining all equilibria with dd1 = dd2 = 0 seems impossible)

```
Factor[(itgenmale[s1, s2, α1, α2, m1, m2, r][{p12, t12, dd1, p22, t22, dd2}] /.
    {dd1 → 0, p22 → p12, dd2 → 0}) - {p12, t12, 0, p12, t22, 0}]
```

$$
\{0, \\
-((2\,m1\,t12 + s1\,t12 + 3\,m1\,s1\,t12 + s1^2\,t12 + m1\,s1^2\,t12 - s1\,t12^2 - 2\,m1\,s1\,t12^2 + 3\,m1^2\,s1\,t12^2 - 2\,s1^2\,t12^2 + 2\,m1^2\,s1^2\,t12^2 + s1^2\,t12^3 - m1\,s1^2\,t12^3 - m1^2\,s1^2\,t12^3 + m1^3\,s1^2\,t12^3 - 2\,m1\,t22 - \\
3\,m1\,s1\,t22 - m1\,s1^2\,t22 + 2\,m1\,s1\,t12\,t22 - 6\,m1^2\,s1\,t12\,t22 - 4\,m1^2\,s1^2\,t12\,t22 + \\
m1\,s1^2\,t12^2\,t22 + 2\,m1^2\,s1^2\,t12^2\,t22 - 3\,m1^3\,s1^2\,t12^2\,t22 + 3\,m1^2\,s1\,t22^2 + 2\,m1^2\,s1^2\,t22^2 - \\
m1^2\,s1^2\,t12\,t22^2 + 3\,m1^3\,s1^2\,t12\,t22^2 - m1^3\,s1^2\,t22^3 + t12\,\alpha1 + m1\,t12\,\alpha1 - p12\,t12\,\alpha1 + \\
m1\,p12\,t12\,\alpha1 + 2\,s1\,t12\,\alpha1 + 2\,m1\,s1\,t12\,\alpha1 - p12\,s1\,t12\,\alpha1 + m1\,p12\,s1\,t12\,\alpha1 + \\
s1^2\,t12\,\alpha1 + m1\,s1^2\,t12\,\alpha1 - t12^2\,\alpha1 + m1^2\,t12^2\,\alpha1 + p12\,t12^2\,\alpha1 - 2\,m1\,p12\,t12^2\,\alpha1 + \\
m1^2\,p12\,t12^2\,\alpha1 - 3\,s1\,t12^2\,\alpha1 + 3\,m1^2\,s1\,t12^2\,\alpha1 + p12\,s1\,t12^2\,\alpha1 - 2\,m1\,p12\,s1\,t12^2\,\alpha1 + \\
m1^2\,p12\,s1\,t12^2\,\alpha1 - 2\,s1^2\,t12^2\,\alpha1 + 2\,m1^2\,s1^2\,t12^2\,\alpha1 + s1\,t12^3\,\alpha1 - m1\,s1\,t12^3\,\alpha1 - \\
m1^2\,s1\,t12^3\,\alpha1 + m1^3\,s1\,t12^3\,\alpha1 + s1^2\,t12^3\,\alpha1 - m1\,s1^2\,t12^3\,\alpha1 - m1^2\,s1^2\,t12^3\,\alpha1 + \\
m1^3\,s1^2\,t12^3\,\alpha1 - m1\,t22\,\alpha1 - m1\,p12\,t22\,\alpha1 - 2\,m1\,s1\,t22\,\alpha1 - m1\,p12\,s1\,t22\,\alpha1 - \\
m1\,s1^2\,t22\,\alpha1 - 2\,m1^2\,t12\,t22\,\alpha1 + 2\,m1\,p12\,t12\,t22\,\alpha1 - 2\,m1^2\,p12\,t12\,t22\,\alpha1 - \\
6\,m1^2\,s1\,t12\,t22\,\alpha1 + 2\,m1\,p12\,s1\,t12\,t22\,\alpha1 - 2\,m1^2\,p12\,s1\,t12\,t22\,\alpha1 - 4\,m1^2\,s1^2\,t12\,t22\,\alpha1 + \\
m1\,s1\,t12^2\,t22\,\alpha1 + 2\,m1^2\,s1\,t12^2\,t22\,\alpha1 - 3\,m1^3\,s1\,t12^2\,t22\,\alpha1 + m1\,s1^2\,t12^2\,t22\,\alpha1 + \\
2\,m1^2\,s1^2\,t12^2\,t22\,\alpha1 - 3\,m1^3\,s1^2\,t12^2\,t22\,\alpha1 + m1^2\,t22^2\,\alpha1 + m1^2\,p12\,t22^2\,\alpha1 + \\
3\,m1^2\,s1\,t22^2\,\alpha1 + m1^2\,p12\,s1\,t22^2\,\alpha1 + 2\,m1^2\,s1^2\,t22^2\,\alpha1 - m1^2\,s1\,t12\,t22^2\,\alpha1 + \\
3\,m1^3\,s1\,t12\,t22^2\,\alpha1 - m1^2\,s1^2\,t12\,t22^2\,\alpha1 + 3\,m1^3\,s1^2\,t12\,t22^2\,\alpha1 - m1^3\,s1\,t22^3\,\alpha1 - \\
m1^3\,s1^2\,t22^3\,\alpha1 - p12\,t12\,\alpha2 + m1\,p12\,t12\,\alpha2 - p12\,s1\,t12\,\alpha2 + m1\,p12\,s1\,t12\,\alpha2 + \\
2\,m1\,t12^2\,\alpha2 - 2\,m1^2\,t12^2\,\alpha2 + p12\,t12^2\,\alpha2 - 2\,m1\,p12\,t12^2\,\alpha2 + m1^2\,p12\,t12^2\,\alpha2 + s1\,t12^2\,\alpha2 - \\
m1^2\,s1\,t12^2\,\alpha2 + p12\,s1\,t12^2\,\alpha2 - 2\,m1\,p12\,s1\,t12^2\,\alpha2 + m1^2\,p12\,s1\,t12^2\,\alpha2 - s1\,t12^3\,\alpha2 + \\
m1\,s1\,t12^3\,\alpha2 + m1^2\,s1\,t12^3\,\alpha2 - m1^3\,s1\,t12^3\,\alpha2 - m1\,p12\,t22\,\alpha2 - m1\,p12\,s1\,t22\,\alpha2 - \\
2\,m1\,t12\,t22\,\alpha2 + 4\,m1^2\,t12\,t22\,\alpha2 + 2\,m1\,p12\,t12\,t22\,\alpha2 - 2\,m1^2\,p12\,t12\,t22\,\alpha2 + \\
2\,m1^2\,s1\,t12\,t22\,\alpha2 + 2\,m1\,p12\,s1\,t12\,t22\,\alpha2 - 2\,m1^2\,p12\,s1\,t12\,t22\,\alpha2 - m1\,s1\,t12^2\,t22\,\alpha2 - \\
2\,m1^2\,s1\,t12^2\,t22\,\alpha2 + 3\,m1^3\,s1\,t12^2\,t22\,\alpha2 - 2\,m1^2\,t22^2\,\alpha2 + m1^2\,p12\,t22^2\,\alpha2 - \\
m1^2\,s1\,t22^2\,\alpha2 + m1^2\,p12\,s1\,t22^2\,\alpha2 + m1^2\,s1\,t12\,t22^2\,\alpha2 - 3\,m1^3\,s1\,t12\,t22^2\,\alpha2 + \\
m1^3\,s1\,t22^3\,\alpha2 - p12\,t12\,\alpha1\,\alpha2 + m1\,p12\,t12\,\alpha1\,\alpha2 - p12\,s1\,t12\,\alpha1\,\alpha2 + m1\,p12\,s1\,t12\,\alpha1\,\alpha2 + \\
t12^2\,\alpha1\,\alpha2 - m1^2\,t12^2\,\alpha1\,\alpha2 + p12\,t12^2\,\alpha1\,\alpha2 - 2\,m1\,p12\,t12^2\,\alpha1\,\alpha2 + m1^2\,p12\,t12^2\,\alpha1\,\alpha2 + \\
s1\,t12^2\,\alpha1\,\alpha2 - m1^2\,s1\,t12^2\,\alpha1\,\alpha2 + p12\,s1\,t12^2\,\alpha1\,\alpha2 - 2\,m1\,p12\,s1\,t12^2\,\alpha1\,\alpha2 + \\
m1^2\,p12\,s1\,t12^2\,\alpha1\,\alpha2 - t12^3\,\alpha1\,\alpha2 + m1\,t12^3\,\alpha1\,\alpha2 + m1^2\,t12^3\,\alpha1\,\alpha2 - m1^3\,t12^3\,\alpha1\,\alpha2 - \\
s1\,t12^3\,\alpha1\,\alpha2 + m1\,s1\,t12^3\,\alpha1\,\alpha2 + m1^2\,s1\,t12^3\,\alpha1\,\alpha2 - m1^3\,s1\,t12^3\,\alpha1\,\alpha2 - m1\,p12\,t22\,\alpha1\,\alpha2 - \\
m1\,p12\,s1\,t22\,\alpha1\,\alpha2 + 2\,m1^2\,t12\,t22\,\alpha1\,\alpha2 + 2\,m1\,p12\,t12\,t22\,\alpha1\,\alpha2 - 2\,m1^2\,p12\,t12\,t22\,\alpha1\,\alpha2 + \\
2\,m1^2\,s1\,t12\,t22\,\alpha1\,\alpha2 + 2\,m1\,p12\,s1\,t12\,t22\,\alpha1\,\alpha2 - 2\,m1^2\,p12\,s1\,t12\,t22\,\alpha1\,\alpha2 - \\
m1\,t12^2\,t22\,\alpha1\,\alpha2 - 2\,m1^2\,t12^2\,t22\,\alpha1\,\alpha2 + 3\,m1^3\,t12^2\,t22\,\alpha1\,\alpha2 - m1\,s1\,t12^2\,t22\,\alpha1\,\alpha2 - \\
2\,m1^2\,s1\,t12^2\,t22\,\alpha1\,\alpha2 + 3\,m1^3\,s1\,t12^2\,t22\,\alpha1\,\alpha2 - m1^2\,t22^2\,\alpha1\,\alpha2 + m1^2\,p12\,t22^2\,\alpha1\,\alpha2 - \\
m1^2\,s1\,t22^2\,\alpha1\,\alpha2 + m1^2\,p12\,s1\,t22^2\,\alpha1\,\alpha2 + m1^2\,t12\,t22^2\,\alpha1\,\alpha2 - 3\,m1^3\,t12\,t22^2\,\alpha1\,\alpha2 + \\
m1^2\,s1\,t12\,t22^2\,\alpha1\,\alpha2 - 3\,m1^3\,s1\,t12\,t22^2\,\alpha1\,\alpha2 + m1^3\,t22^3\,\alpha1\,\alpha2 + m1^3\,s1\,t22^3\,\alpha1\,\alpha2) / \\
(2\,(1 + s1 - s1\,t12 + m1\,s1\,t12 - m1\,s1\,t22 + \alpha1 + s1\,\alpha1 - t12\,\alpha1 + m1\,t12\,\alpha1 - \\
s1\,t12\,\alpha1 + m1\,s1\,t12\,\alpha1 - m1\,t22\,\alpha1 - m1\,s1\,t22\,\alpha1)
$$

```
        (1 + s1 - s1 t12 + m1 s1 t12 - m1 s1 t22 + t12 α2 - m1 t12 α2 + m1 t22 α2))),
  ((-1 + p12) p12 r (1 + s1) (-t12 + m1 t12 - m1 t22) (1 - t12 + m1 t12 - m1 t22)
      (α1 + α2 + α1 α2)) /
    (2 (1 + s1 - s1 t12 + m1 s1 t12 - m1 s1 t22 + α1 + s1 α1 - t12 α1 + m1 t12 α1 -
          s1 t12 α1 + m1 s1 t12 α1 - m1 t22 α1 - m1 s1 t22 α1)
      (1 + s1 - s1 t12 + m1 s1 t12 - m1 s1 t22 + t12 α2 - m1 t12 α2 + m1 t22 α2)), 0,
  -((-2 m2 t12 - m2 s2 t12 - 3 m2^2 s2 t12^2 - m2^2 s2^2 t12^2 - m2^3 s2^2 t12^3 + 2 m2 t22 - s2 t22 +
        m2 s2 t22 - 2 m2 s2 t12 t22 + 6 m2^2 s2 t12 t22 - 2 m2 s2^2 t12 t22 + 2 m2^2 s2^2 t12 t22 -
        m2^2 s2^2 t12^2 t22 + 3 m2^3 s2^2 t12^2 t22 + s2 t22^2 + 2 m2 s2 t22^2 - 3 m2^2 s2 t22^2 - s2^2 t22^2 +
        2 m2 s2^2 t22^2 - m2^2 s2^2 t22^2 + m2 s2^2 t12 t22^2 + 2 m2^2 s2^2 t12 t22^2 - 3 m2^3 s2^2 t12 t22^2 +
        s2^2 t22^3 - m2 s2^2 t22^3 - m2^2 s2^2 t22^3 + m2^3 s2^2 t22^3 - m2 t12 α1 - m2 p12 t12 α1 -
        m2 p12 s2 t12 α1 + m2^2 t12^2 α1 + m2^2 p12 t12^2 α1 - m2^2 s2 t12^2 α1 + m2^2 p12 s2 t12^2 α1 +
        m2^3 s2 t12^3 α1 + t22 α1 + m2 t22 α1 - p12 t22 α1 + m2 p12 t22 α1 - p12 s2 t22 α1 +
        m2 p12 s2 t22 α1 - 2 m2^2 t12 t22 α1 + 2 m2 p12 t12 t22 α1 - 2 m2^2 p12 t12 t22 α1 +
        2 m2^2 s2 t12 t22 α1 + 2 m2 p12 s2 t12 t22 α1 - 2 m2^2 p12 s2 t12 t22 α1 + m2^2 s2 t12^2 t22 α1 -
        3 m2^3 s2 t12^2 t22 α1 - t22^2 α1 + m2^2 t22^2 α1 + p12 t22^2 α1 - 2 m2 p12 t22^2 α1 + m2^2 p12 t22^2 α1 +
        s2 t22^2 α1 - m2^2 s2 t22^2 α1 + p12 s2 t22^2 α1 - 2 m2 p12 s2 t22^2 α1 + m2^2 p12 s2 t22^2 α1 -
        m2 s2 t12 t22^2 α1 - 2 m2^2 s2 t12 t22^2 α1 + 3 m2^3 s2 t12 t22^2 α1 - s2 t22^3 α1 + m2 s2 t22^3 α1 +
        m2^2 s2 t22^3 α1 - m2^3 s2 t22^3 α1 - m2 p12 t12 α2 - m2 p12 s2 t12 α2 - 2 m2^2 t12^2 α2 +
        m2^2 p12 t12^2 α2 - 3 m2^2 s2 t12^2 α2 + m2^2 p12 s2 t12^2 α2 - m2^2 s2^2 t12^2 α2 - m2^3 s2 t12^3 α2 -
        m2^3 s2^2 t12^3 α2 - p12 t22 α2 + m2 p12 t22 α2 - p12 s2 t22 α2 + m2 p12 s2 t22 α2 -
        2 m2 t12 t22 α2 + 4 m2^2 t12 t22 α2 + 2 m2 p12 t12 t22 α2 - 2 m2^2 p12 t12 t22 α2 -
        4 m2 s2 t12 t22 α2 + 6 m2^2 s2 t12 t22 α2 + 2 m2 p12 s2 t12 t22 α2 - 2 m2^2 p12 s2 t12 t22 α2 -
        2 m2 s2^2 t12 t22 α2 + 2 m2^2 s2^2 t12 t22 α2 - m2^2 s2 t12^2 t22 α2 + 3 m2^3 s2 t12^2 t22 α2 -
        m2^2 s2^2 t12^2 t22 α2 + 3 m2^3 s2^2 t12^2 t22 α2 + 2 m2 t22^2 α2 - 2 m2^2 t22^2 α2 + p12 t22^2 α2 -
        2 m2 p12 t22^2 α2 + m2^2 p12 t22^2 α2 - s2 t22^2 α2 + 4 m2 s2 t22^2 α2 - 3 m2^2 s2 t22^2 α2 +
        p12 s2 t22^2 α2 - 2 m2 p12 s2 t22^2 α2 + m2^2 p12 s2 t22^2 α2 - s2^2 t22^2 α2 + 2 m2 s2^2 t22^2 α2 -
        m2^2 s2^2 t22^2 α2 + m2 s2 t12 t22^2 α2 + 2 m2^2 s2 t12 t22^2 α2 - 3 m2^3 s2 t12 t22^2 α2 +
        m2 s2^2 t12 t22^2 α2 + 2 m2^2 s2^2 t12 t22^2 α2 - 3 m2^3 s2^2 t12 t22^2 α2 + s2 t22^3 α2 - m2 s2 t22^3 α2 -
        m2^2 s2 t22^3 α2 + m2^3 s2 t22^3 α2 + s2^2 t22^3 α2 - m2 s2^2 t22^3 α2 - m2^2 s2^2 t22^3 α2 +
        m2^3 s2^2 t22^3 α2 - m2 p12 t12 α1 α2 - m2 p12 s2 t12 α1 α2 - m2^2 t12^2 α1 α2 + m2^2 p12 t12^2 α1 α2 -
        m2^2 s2 t12^2 α1 α2 + m2^2 p12 s2 t12^2 α1 α2 + m2^3 t12^3 α1 α2 + m2^3 s2 t12^3 α1 α2 - p12 t22 α1 α2 +
        m2 p12 t22 α1 α2 - p12 s2 t22 α1 α2 + m2 p12 s2 t22 α1 α2 + 2 m2^2 t12 t22 α1 α2 +
        2 m2 p12 t12 t22 α1 α2 - 2 m2^2 p12 t12 t22 α1 α2 + 2 m2^2 s2 t12 t22 α1 α2 +
        2 m2 p12 s2 t12 t22 α1 α2 - 2 m2^2 p12 s2 t12 t22 α1 α2 + m2^2 t12^2 t22 α1 α2 -
        3 m2^3 t12^2 t22 α1 α2 + m2^2 s2 t12^2 t22 α1 α2 - 3 m2^3 s2 t12^2 t22 α1 α2 + t22^2 α1 α2 -
        m2^2 t22^2 α1 α2 + p12 t22^2 α1 α2 - 2 m2 p12 t22^2 α1 α2 + m2^2 p12 t22^2 α1 α2 + s2 t22^2 α1 α2 -
        m2^2 s2 t22^2 α1 α2 + p12 s2 t22^2 α1 α2 - 2 m2 p12 s2 t22^2 α1 α2 + m2^2 p12 s2 t22^2 α1 α2 -
        m2 t12 t22^2 α1 α2 - 2 m2^2 t12 t22^2 α1 α2 + 3 m2^3 t12 t22^2 α1 α2 - m2 s2 t12 t22^2 α1 α2 -
        2 m2^2 s2 t12 t22^2 α1 α2 + 3 m2^3 s2 t12 t22^2 α1 α2 - t22^3 α1 α2 + m2 t22^3 α1 α2 + m2^2 t22^3 α1 α2 -
        m2^3 t22^3 α1 α2 - s2 t22^3 α1 α2 + m2 s2 t22^3 α1 α2 + m2^2 s2 t22^3 α1 α2 - m2^3 s2 t22^3 α1 α2) /
      (2 (-1 - m2 s2 t12 - s2 t22 + m2 s2 t22 - α1 + m2 t12 α1 + t22 α1 - m2 t22 α1)
        (-1 - m2 s2 t12 - s2 t22 + m2 s2 t22 - m2 t12 α2 -
          m2 s2 t12 α2 - t22 α2 + m2 t22 α2 - s2 t22 α2 + m2 s2 t22 α2))),
  ((-1 + p12) p12 r (1 + s2) (-1 + m2 t12 + t22 - m2 t22) (m2 t12 + t22 - m2 t22)
      (α1 + α2 + α1 α2)) /
    (2 (1 + m2 s2 t12 + s2 t22 - m2 s2 t22 + α1 - m2 t12 α1 - t22 α1 + m2 t22 α1)
      (1 + m2 s2 t12 + s2 t22 - m2 s2 t22 + m2 t12 α2 +
        m2 s2 t12 α2 + t22 α2 - m2 t22 α2 + s2 t22 α2 - m2 s2 t22 α2))}
```

```
Simplify[Solve[{((-1 + p12) p12 r (1 + s1) (-t12 + m1 t12 - m1 t22)
      (1 - t12 + m1 t12 - m1 t22) (α1 + α2 + α1 α2)), ((-1 + p12) p12 r (1 + s2)
      (-1 + m2 t12 + t22 - m2 t22) (m2 t12 + t22 - m2 t22) (α1 + α2 + α1 α2))} == 0, {t12, t22}]]
```

$\{\{t12 \to 0, t22 \to 0\}, \{t12 \to 1, t22 \to 1\}, \{t12 \to \frac{m1}{-1 + m1 + m2}, t22 \to \frac{-1 + m1}{-1 + m1 + m2}\}, \{t12 \to \frac{-1 + m2}{-1 + m1 + m2}, t22 \to \frac{m2}{-1 + m1 + m2}\}\}$

If 0<p12<1, the only admissible solutions are {t12 -> 0, t22 -> 0}, {t12 -> 1, t22 -> 1}.

Assume p12 = p22 = 0

```
FullSimplify[(itgenmale[s1, s2, α1, α2, m1, m2, r][{p12, t12, dd1, p22, t22, dd2}] /.
    {p12 → 0, dd1 → 0, p22 → 0, dd2 → 0}) - {0, t12, 0, 0, t22, 0}]
```

$\{0, \frac{1}{2}(-t12 - m1\,t12 + m1\,t22 + (t12 - m1\,t12 + m1\,t22) / (1 + \alpha1 + (-1 + m1)\,t12\,\alpha1 - m1\,t22\,\alpha1 + s1\,(1 + (-1 + m1)\,t12 - m1\,t22)\,(1 + \alpha1))), 0, 0, (m2^2\,(t12 - t22)^2\,(s2 - \alpha1) - (-1 + t22)\,t22\,(s2 - \alpha1) + m2\,(t12 - t22)\,(2 + s2 + \alpha1)) / (2\,(1 + (m2\,(t12 - t22) + t22)\,(s2 - \alpha1) + \alpha1)), 0\}$

```
FullSimplify[Numerator[Factor[-t12 - m1 t12 + m1 t22 + (t12 - m1 t12 + m1 t22) /
    (1 + α1 + (-1 + m1) t12 α1 - m1 t22 α1 + s1 (1 + (-1 + m1) t12 - m1 t22) (1 + α1))]]]
```

$(-1 + t12)\,t12\,(s1 + \alpha1 + s1\,\alpha1) - m1^2\,(t12 - t22)^2\,(s1 + \alpha1 + s1\,\alpha1) - m1\,(t12 - t22)\,(2 + s1 + \alpha1 + s1\,\alpha1)$

The first term is negative. Therefore, the above expression can be 0 only if the sum of the second and third is negative.
check when it is 0:

```
Simplify[
  Solve[m1^2 (t12 - t22)^2 (s1 + α1 + s1 α1) + m1 (t12 - t22) (2 + s1 + α1 + s1 α1) == 0, t22]]
```

$\{\{t22 \to t12\}, \{t22 \to (2 + \alpha1 + m1\,t12\,\alpha1 + s1\,(1 + m1\,t12)\,(1 + \alpha1)) / (m1\,(s1 + \alpha1 + s1\,\alpha1))\}\}$

Therefore the smallest value of t22 (from the second solution) occurs if t12 = 0:

```
Simplify[(2 + α1 + m1 t12 α1 + s1 (1 + m1 t12) (1 + α1)) / (m1 (s1 + α1 + s1 α1)) /. t12 → 0]
```

$$\frac{2 + s1 + \alpha1 + s1\,\alpha1}{m1\,(s1 + \alpha1 + s1\,\alpha1)}$$

This exceeds 1 for all reasonable migration rates, in particular if m1 > 1. Therefore, no solution other than {t12→0,t22→0} or {t12→1,t22→1} is possible.

Assume t12 = t22 = 0 (loss of trait allele T2)

```
Factor[(itgenmale[s1, s2, α1, α2, m1, m2, r][{p12, t12, dd1, p22, t22, dd2}] /.
    {t12 → 0, dd1 → 0, t22 → 0, dd2 → 0}) - {p12, 0, 0, p22, 0, 0}]
```

$\{-m1\,(p12 - p22), 0, 0, -m2\,(-p12 + p22), 0, 0\}$

Therefore, as expected p12 = p22 holds at these equilibria

Assume t12 = t22 = 1

```
Factor[(itgenmale[s1, s2, α1, α2, m1, m2, r][{p12, t12, dd1, p22, t22, dd2}] /.
    {t12 → 1, dd1 → 0, t22 → 1, dd2 → 0}) - {p12, 1, 0, p22, 1, 0}]
```

```
{-m1 (p12 - p22), 0, 0, -m2 (-p12 + p22), 0, 0}
```

Therefore, p12 = p22 holds at these equilibria

2.1.2 Jacobians and stability of equilibria, where one trait allele is fixed

Stability of equilibria at the edge t12 = t22 = 0

```
JacoT20 =
  Simplify[D[itgenmale[s1, s2, α1, α2, m1, m2, r][{p12, t12, dd1, p22, t22, dd2}],
     {{p12, t12, dd1, p22, t22, dd2}}] /. {t12 → 0, dd1 → 0, t22 → 0, dd2 → 0, p12 → p22}]
```

```
{{1 - m1, 0, ((-1 + m1) (s1 (1 + α1) - p22 α2 - α1 (-1 + p22 + p22 α2))) / (2 (1 + s1) (1 + α1)),
  m1, 0, (m1 (-s1 (1 + α1) + p22 α2 + α1 (-1 + p22 + p22 α2))) / (2 (1 + s1) (1 + α1))},
 {0, -(((-1 + m1) (2 + s1 + α1 + p22 α1 + s1 α1 + p22 α2 + p22 α1 α2)) / (2 (1 + s1) (1 + α1))),
  0, 0, (m1 (2 + s1 + α1 + p22 α1 + s1 α1 + p22 α2 + p22 α1 α2)) / (2 (1 + s1) (1 + α1)), 0},
 {0, ((-1 + m1) (-1 + p22) p22 r (α1 + α2 + α1 α2)) / (2 (1 + s1) (1 + α1)),
  ((-1 + m1) (-1 + r) (2 + s1 + α1 + p22 α1 + s1 α1 + p22 α2 + p22 α1 α2)) / (2 (1 + s1) (1 + α1)),
  0, -((m1 (-1 + p22) p22 r (α1 + α2 + α1 α2)) / (2 (1 + s1) (1 + α1))),
  -((m1 (-1 + r) (2 + s1 + α1 + p22 α1 + s1 α1 + p22 α2 + p22 α1 α2)) / (2 (1 + s1) (1 + α1)))},
 {m2, 0, (m2 (s2 + p22 α2 + α1 (-1 + p22 + p22 α2) + p22 s2 (α1 + α2 + α1 α2))) / (2 (1 + α1)),
  1 - m2, 0,
  -(((-1 + m2) (s2 + p22 α2 + α1 (-1 + p22 + p22 α2) + p22 s2 (α1 + α2 + α1 α2))) / (2 (1 + α1)))},
 {0, (m2 (2 + s2 + p22 α2 + α1 (1 + p22 + p22 α2) + p22 s2 (α1 + α2 + α1 α2))) / (2 (1 + α1)),
  0, 0, -(((-1 + m2) (2 + s2 + p22 α2 + α1 (1 + p22 + p22 α2) + p22 s2 (α1 + α2 + α1 α2))) /
      (2 (1 + α1))), 0}, {0, -((m2 (-1 + p22) p22 r (1 + s2) (α1 + α2 + α1 α2)) / (2 (1 + α1))),
  -((m2 (-1 + r) (2 + s2 + p22 α2 + α1 (1 + p22 + p22 α2) + p22 s2 (α1 + α2 + α1 α2))) /
      (2 (1 + α1))), 0, ((-1 + m2) (-1 + p22) p22 r (1 + s2) (α1 + α2 + α1 α2)) / (2 (1 + α1)),
  ((-1 + m2) (-1 + r) (2 + s2 + p22 α2 + α1 (1 + p22 + p22 α2) + p22 s2 (α1 + α2 + α1 α2))) /
   (2 (1 + α1))}}
```

Characteristic polynomial at this equilibrium:

```
cpolT20 = Simplify[Det[JacoT20 - x IdentityMatrix[6]]]
```

```
          1
--------------------------
16 (1 + s1)^2 (1 + α1)^4

 (-1 + x) (-1 + m1 + m2 + x) (-m1 m2 (2 + s1 + α1 + p22 α1 + s1 α1 + p22 α2 + p22 α1 α2)
       (2 + s2 + p22 α2 + α1 (1 + p22 + p22 α2) + p22 s2 (α1 + α2 + α1 α2)) +
     (-2 + 2 x - α1 - p22 α1 + 2 x α1 + s1 (-1 + 2 x) (1 + α1) - p22 α2 -
        p22 α1 α2 + m1 (2 + s1 + α1 + p22 α1 + s1 α1 + p22 α2 + p22 α1 α2))
      (-2 + 2 x - α1 - p22 α1 + 2 x α1 - p22 α2 - p22 α1 α2 - s2 (1 + p22 (α1 + α2 + α1 α2)) +
        m2 (2 + s2 + p22 α2 + α1 (1 + p22 + p22 α2) + p22 s2 (α1 + α2 + α1 α2))))
  (-m1 m2 (-1 + r)^2 (2 + s1 + α1 + p22 α1 + s1 α1 + p22 α2 + p22 α1 α2)
      (2 + s2 + p22 α2 + α1 (1 + p22 + p22 α2) + p22 s2 (α1 + α2 + α1 α2)) +
     (2 + s1 - 2 x - 2 s1 x + α1 + p22 α1 + s1 α1 - 2 x α1 - 2 s1 x α1 + p22 α2 +
        p22 α1 α2 + m1 (-1 + r) (2 + s1 + α1 + p22 α1 + s1 α1 + p22 α2 + p22 α1 α2) -
        r (2 + s1 + α1 + p22 α1 + s1 α1 + p22 α2 + p22 α1 α2))
      (2 + s2 - 2 x + α1 + p22 α1 + p22 s2 α1 - 2 x α1 + p22 α2 + p22 s2 α2 + p22 α1 α2 + p22 s2 α1 α2 +
        m2 (-1 + r) (2 + s2 + p22 α2 + α1 (1 + p22 + p22 α2) + p22 s2 (α1 + α2 + α1 α2)) -
        r (2 + s2 + p22 α2 + α1 (1 + p22 + p22 α2) + p22 s2 (α1 + α2 + α1 α2))))
```

There are four factors; x = 1 and x = 1 - m1 - m2 are always eigenvalues.
In addition, there is one factor without r, and one factor with r. Both factors are quadratic in x and given below:

```
cpolT20noR[x_, p22_] := (-m1 m2 (2 + s1 + α1 + p22 α1 + s1 α1 + p22 α2 + p22 α1 α2)
       (2 + p22 α2 + α1 (1 + p22 + p22 α2) + s2 (1 + p22 (α1 + α2 + α1 α2))) +
     (-2 + 2 x - α1 - p22 α1 + 2 x α1 + s1 (-1 + 2 x) (1 + α1) - p22 α2 -
        p22 α1 α2 + m1 (2 + s1 + α1 + p22 α1 + s1 α1 + p22 α2 + p22 α1 α2))
      (-2 + 2 x - α1 - p22 α1 + 2 x α1 - p22 α2 - p22 α1 α2 - s2 (1 + p22 (α1 + α2 + α1 α2)) +
        m2 (2 + s2 + p22 α2 + α1 (1 + p22 + p22 α2) + s2 p22 (α1 + α2 + α1 α2)))) ;
```

```
cpolT20R[x_, p22_] := (-m1 m2 (-1 + r)^2 (2 + s1 + α1 + p22 α1 + s1 α1 + p22 α2 + p22 α1 α2)
       (2 + p22 α2 + α1 (1 + p22 + p22 α2) + s2 (1 + p22 (α1 + α2 + α1 α2))) +
     (2 + s1 - 2 x - 2 s1 x + α1 + p22 α1 + s1 α1 - 2 x α1 - 2 s1 x α1 + p22 α2 +
        p22 α1 α2 + m1 (-1 + r) (2 + s1 + α1 + p22 α1 + s1 α1 + p22 α2 + p22 α1 α2) -
        r (2 + s1 + α1 + p22 α1 + s1 α1 + p22 α2 + p22 α1 α2))
      (2 + s2 - 2 x + α1 + p22 α1 + p22 s2 α1 - 2 x α1 + p22 α2 + p22 s2 α2 + p22 α1 α2 + p22 s2 α1
           α2 + m2 (-1 + r) (2 + p22 α2 + α1 (1 + p22 + p22 α2) + s2 (1 + p22 (α1 + α2 + α1 α2))) -
        r (2 + p22 α2 + α1 (1 + p22 + p22 α2) + s2 (1 + p22 (α1 + α2 + α1 α2)))));
```

```
Simplify[Series[cpolT20R[x, p22], {x, 0, 3}]]
```

```
- (-1 + m1 + m2) (-1 + r)^2 (2 + s1 + α1 + p22 α1 + s1 α1 + p22 α2 + p22 α1 α2)
    (2 + s2 + p22 α2 + α1 (1 + p22 + p22 α2) + p22 s2 (α1 + α2 + α1 α2)) +
  2 (-1 + r) (1 + α1) (- (-1 + m1) (2 + s1 + α1 + p22 α1 + s1 α1 + p22 α2 + p22 α1 α2) -
       (-1 + m2) (1 + s1) (2 + s2 + p22 α2 + α1 (1 + p22 + p22 α2) + p22 s2 (α1 + α2 + α1 α2)))
   x + 4 (1 + s1) (1 + α1)^2 x^2 + O[x]^4
```

```
Simplify[Series[cpolT20noR[x, p22], {x, 0, 3}]]
```

$$-(-1+m1+m2)(2+s1+\alpha1+p22\,\alpha1+s1\,\alpha1+p22\,\alpha2+p22\,\alpha1\,\alpha2)(2+s2+p22\,\alpha2+\alpha1(1+p22+p22\,\alpha2)+p22\,s2(\alpha1+\alpha2+\alpha1\,\alpha2))+2(1+\alpha1)((-1+m1)(2+s1+\alpha1+p22\,\alpha1+s1\,\alpha1+p22\,\alpha2+p22\,\alpha1\,\alpha2)+(-1+m2)(1+s1)(2+s2+p22\,\alpha2+\alpha1(1+p22+p22\,\alpha2)+p22\,s2(\alpha1+\alpha2+\alpha1\,\alpha2)))\,x+4(1+s1)(1+\alpha1)^2x^2+O[x]^4$$

Therefore, we find

```
Simplify[cpolT20R[x (1 - r), p22] - (1 - r)^2 cpolT20noR[x, p22]]
```

0

Therefore, cpolT20noR[x,p22] = 0 if and only if cpolT20R[(1-r)x,p22] = 0.
Hence, x is an eigenvalue resulting from cpolT20noR[x,p22] = 0 if and only if (1-r)x is an eigenvalue resulting from cpolT20R[x,p22] = 0.

As a consequence, an equilibrium p22 (=p12) at this edge (t12=t22=0, dd1=dd2=0) is unstable if and only if there exists and eigenvalue x>1 with cpolT20noR[x,p22] = 0.
Thus, a change of stability at this edge occurs at a point p22 if cpolT20noR[1,p22] = 0.

The curve of equilibria that connects the edges t12=t22=0 and t12=t22=1 has its endpoint at the value p22, at which the stability at this edge changes, i.e., where cpolT20noR[1,p22] = 0.
This is easily computed numerically.

Study eigenvalues at p22 = 0

By the above, it is sufficient to study the zeroes x resulting from cpolT20noR (x, 0) = 0

```
Simplify[Series[cpolT20noR[x, 0], {x, 0, 2}]]
```

$$-(-1+m1+m2)(2+s2+\alpha1)(2+s1+\alpha1+s1\,\alpha1)+2(1+\alpha1)(-4-3\,s1-s2-s1\,s2-2\,\alpha1-2\,s1\,\alpha1+m2(1+s1)(2+s2+\alpha1)+m1(2+s1+\alpha1+s1\,\alpha1))\,x+4(1+s1)(1+\alpha1)^2x^2+O[x]^3$$

The leading term is positive.

```
Simplify[cpolT20noR[0, 0]]
```

$$-(-1+m1+m2)(2+s2+\alpha1)(2+s1+\alpha1+s1\,\alpha1)$$

This is positive if m1 + m2 < 1, as we assume henceforth!
Therefore, there is one eigenvalue in (0,1) and one eigenvalue greater than 1 if and only if cpolT20noR[1, 0] < 0.

```
Simplify[cpolT20noR[1, 0]]
```

$$-m1\,m2(2+s2+\alpha1)(2+s1+\alpha1+s1\,\alpha1)+(-s2+\alpha1+m2(2+s2+\alpha1))(s1+\alpha1+s1\,\alpha1+m1(2+s1+\alpha1+s1\,\alpha1))$$

```
FullSimplify[Series[cpolT20noR[1, 0], {α1, 0, 3}]]
```

$(-(s1 + m1 (2 + s1)) s2 + m2 s1 (2 + s2)) + (2 m1 + 2 m2 + s1 + m1 s1 + 3 m2 s1 - (1 + m1 - m2) (1 + s1) s2) \alpha1 + (1 + m1 + m2) (1 + s1) \alpha1^2 + O[\alpha1]^4$

This is negative if $\alpha1 = 0$ and positive if $\alpha1$ is sufficiently large:

```
Simplify[cpolT20noR[1, 0] /. α1 → 0]
```

$-(s1 + m1 (2 + s1)) s2 + m2 s1 (2 + s2)$

The critical value $\alpha1crit$ of $\alpha1$ at which cpolT20noR[1,0]=0 is the second of the following solutions:

```
Simplify[Solve[cpolT20noR[1, 0] == 0, α1]]
```

$$\{\{\alpha1 \to -\frac{1}{2 (1 + m1 + m2) (1 + s1)} \Big(2 m1 + 2 m2 + s1 + m1 s1 + 3 m2 s1 - s2 - m1 s2 + m2 s2 - s1 s2 - m1 s1 s2 + m2 s1 s2 + \sqrt{\big(4 (1 + m1 + m2) (1 + s1) ((s1 + m1 (2 + s1)) s2 - m2 s1 (2 + s2)) + (s1 - s2 - s1 s2 + m1 (2 + s1 - s2 - s1 s2) + m2 (2 + s2 + s1 (3 + s2)))^2\big)}\Big)\},$$
$$\{\alpha1 \to \frac{1}{2 (1 + m1 + m2) (1 + s1)} \Big(-2 m1 - 2 m2 - s1 - m1 s1 - 3 m2 s1 + s2 + m1 s2 - m2 s2 + s1 s2 + m1 s1 s2 - m2 s1 s2 + \sqrt{\big(4 (1 + m1 + m2) (1 + s1) ((s1 + m1 (2 + s1)) s2 - m2 s1 (2 + s2)) + (s1 - s2 - s1 s2 + m1 (2 + s1 - s2 - s1 s2) + m2 (2 + s2 + s1 (3 + s2)))^2\big)}\Big)\}\}$$

The second solution is the bigger one, hence gives the zero.

$$\alpha1crit = \frac{1}{2 (1 + m1 + m2) (1 + s1)} \Big(-2 m1 - 2 m2 - s1 - m1 s1 - 3 m2 s1 + s2 + m1 s2 - m2 s2 + s1 s2 + m1 s1 s2 - m2 s1 s2 + \sqrt{\big(4 (1 + m1 + m2) (1 + s1) ((s1 + m1 (2 + s1)) s2 - m2 s1 (2 + s2)) + (s1 - s2 - s1 s2 + m1 (2 + s1 - s2 - s1 s2) + m2 (2 + s2 + s1 (3 + s2)))^2\big)}\Big);$$

```
Plot[α1crit /. {m1 → 0.1, m2 → 0.1, s2 → 0.05}, {s1, 0, 2}, PlotRange → All]
```

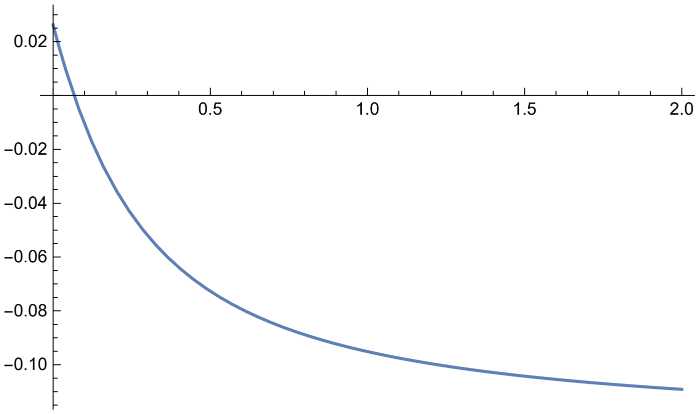

Therefore, if $\alpha1 < \alpha1crit$, then cpolT20noR[1, 0] < 0 and p22 = 0 is unstable.

Symmetric case :

```
Simplify[α1crit /. {s2 → s1, m2 → m1}]
```

$$\left(s1^2 - 4 m1 (1 + s1) + \sqrt{16 m1^2 (1 + s1)^2 + s1^2 (2 + s1)^2}\right) \Big/ (2 (1 + 2 m1) (1 + s1))$$

```
Simplify[
 Series[(s1^2 - 4 m1 (1 + s1) + √(16 m1^2 (1 + s1)^2 + s1^2 (2 + s1)^2)) / (2 (1 + 2 m1) (1 + s1)),
  {m1, 0, 1}], Assumptions → s1 > 0]
```

```
s1 - 2 (1 + s1) m1 + O[m1]^2
```

Therefore, cpolT20noR[1, 0] < 0 if and only if α1 <
(s1^2 - 4 m1 (1 + s1) + √(16 m1^2 (1 + s1)^2 + s1^2 (2 + s1)^2)) / (2 (1 + 2 m1) (1 + s1)) ≈
s1 - 2 m1 (1 + s1).
In this case p22 = 0 is unstable.

For the symmetric case, we also show that p22=0 is stable if α1>α1crit.
To show this, determine minimum of the polynomial and its value at the minimum

```
Simplify[Solve[D[cpolT20noR[x, 0] /. {s2 → s1, α2 → α1, m2 → m1}, x] == 0, x]]
```

```
{{x → -(((-1 + m1) (s1^2 + 2 (2 + α1) + 2 s1 (2 + α1))) / (4 (1 + s1) (1 + α1)))}}
```

```
Simplify[Reduce[(s1^2 + 2 (2 + α1) + 2 s1 (2 + α1)) / (4 (1 + s1) (1 + α1)) < 1, α1], Assumptions → {s1 > 0, α1 > 0}]
```

```
2 (1 + s1) α1 > s1^2
```

```
Simplify[
 cpolT20noR[-(((-1 + m1) (s1^2 + 2 (2 + α1) + 2 s1 (2 + α1))) / (4 (1 + s1) (1 + α1))), 0] /.
  {s2 → s1, α2 → α1, m2 → m1}]
```

```
-((s1^2 (2 + s1)^2 - 2 m1 s1^2 (2 + s1)^2 + m1^2 (s1^2 + 2 (2 + α1) + 2 s1 (2 + α1))^2) / (4 (1 + s1)))
```

```
Simplify[Reduce[
  -((s1^2 (2 + s1)^2 - 2 m1 s1^2 (2 + s1)^2 + m1^2 (s1^2 + 2 (2 + α1) + 2 s1 (2 + α1))^2) / (4 (1 + s1))) <
    0 && 0 < m1 < 1/2 && s1 > 0 && α1 > 0]]
```

```
α1 > 0 && s1 > 0 && 0 < m1 < 1/2
```

Therefore, the minimum is always negative!

It follows that both eigenvalues are less than one if and only if
α1 > Max[(s1^2 - 4 m1 (1 + s1) + √(16 m1^2 (1 + s1)^2 + s1^2 (2 + s1)^2)) / (2 (1 + 2 m1) (1 + s1)), s1^2/(2 (1+s1))]

```
Reduce[(s1^2 - 4 m1 (1 + s1) + √(16 m1^2 (1 + s1)^2 + s1^2 (2 + s1)^2)) / (2 (1 + 2 m1) (1 + s1)) <
   s1^2/(2 (1 + s1)) && 0 < m1 < 1/2 && s1 > 0]
```

```
False
```

It follows that both eigenvalues are less than one if and only if
α1 > α1crit =
(s1^2 - 4 m1 (1 + s1) + √(16 m1^2 (1 + s1)^2 + s1^2 (2 + s1)^2)) / (2 (1 + 2 m1) (1 + s1)) ≈
s1 - 2 m1 (1 + s1)

In summary, p12 = p22 = 0 is stable if and only if α1 > α1crit

Study eigenvalues at p22 = 1 (assuming symmetric parameters, i.e., s2=s1, α2=α1, m2=m1)

```
Simplify[cpolT20noR[x, 1] /. {s2 → s1, α2 → α1, m2 → m1}]
```

$(1+\alpha1)^2 \left(-m1^2 (2+s1+\alpha1)(2+s1+\alpha1+s1\,\alpha1) + (-2+2x+s1(-1+2x)-\alpha1+m1(2+s1+\alpha1)) (-2+2x-\alpha1-s1(1+\alpha1)+m1(2+s1+\alpha1+s1\,\alpha1))\right)$

```
Simplify[
 Series[(-m1^2 (2 + s1 + α1) (2 + s1 + α1 + s1 α1) + (-2 + 2 x + s1 (-1 + 2 x) - α1 + m1 (2 + s1 + α1))
     (-2 + 2 x - α1 - s1 (1 + α1) + m1 (2 + s1 + α1 + s1 α1))), {x, 0, 2}]]
```

$-(-1+2\,m1)\left(s1^2(1+\alpha1)+(2+\alpha1)^2+s1(2+\alpha1)^2\right)+ 2(-1+m1)\left(s1^2(1+\alpha1)+2(2+\alpha1)+2\,s1(2+\alpha1)\right)x+4(1+s1)x^2+O[x]^3$

The constant term is positive if m1 < 1/2; the linear term is negative; the leading term is positive. Throughout, we assume m1<1/2. Then the leading eigenvalue x is in (0,1) if and only if cpolT20noR[1,1]>0.

```
cpolT20P21noRsym[x_] := - (-1 + 2 m1) (s1^2 (1 + α1) + (2 + α1)^2 + s1 (2 + α1)^2) +
  2 (-1 + m1) (s1^2 (1 + α1) + 2 (2 + α1) + 2 s1 (2 + α1)) x + 4 (1 + s1) x^2
```

```
Simplify[Series[cpolT20P21noRsym[1], {α1, 0, 3}]]
```

$-s1^2+\left(-s1^2-4\,m1(1+s1)\right)\alpha1-(-1+2\,m1)(1+s1)\,\alpha1^2+O[\alpha1]^4$

This is negative for α1 = 0 and positive for sufficiently large α1 provided 0 < m1 < 1/2.

```
Simplify[Solve[cpolT20P21noRsym[1] == 0, α1]]
```

$\left\{\left\{\alpha1\to\left(-s1^2-4\,m1(1+s1)+\sqrt{\left(16\,m1^2(1+s1)^2+s1^2(2+s1)^2\right)}\right)\Big/\left(2(-1+2\,m1)(1+s1)\right)\right\},\left\{\alpha1\to-\left(\left(s1^2+4\,m1(1+s1)+\sqrt{\left(16\,m1^2(1+s1)^2+s1^2(2+s1)^2\right)}\right)\Big/\left(2(-1+2\,m1)(1+s1)\right)\right)\right\}\right\}$

The first solution is negative if m1 < 1/2. Thus cpolT20P21noRsym[1] > 0 if α1 > α1crit2, where

```
α1crit2 = (s1^2 + 4 m1 (1 + s1) + √(16 m1^2 (1 + s1)^2 + s1^2 (2 + s1)^2)) / (2 (1 - 2 m1) (1 + s1));
```

```
FullSimplify[α1crit2 /. m1 → 0, Assumptions → s1 ≥ 0]
```

s1

```
Simplify[Series[α1crit2, {m1, 0, 1}], Assumptions → s1 ≥ 0]
```

$s1+(2+2\,s1)\,m1+O[m1]^2$

Therefore,
p12 = p22 = 1 is stable if and only if α1 > α1crit2 = $s1+(2+2\,s1)\,m1+O[m1]^2$
Because α1crit2 > α1crit1, this implies stability of the whole edge.

Display parameter region in which p12=p22=0 (t12=t22=0) is stable (for symmetric parameters)

```
Simplify[cpolT20noR[x, 0] /. {s2 → s1, α2 → α1, m2 → m1}]
```

```
s1^2 (-1 + 2 x) (1 + α1) - (2 + α1 - 2 x (1 + α1))^2 - s1 (2 + α1 - 2 x (1 + α1))^2 - 2 m1
  (s1^2 (-1 + x) (1 + α1) + (2 + α1) (-2 - α1 + 2 x (1 + α1)) + s1 (2 + α1) (-2 - α1 + 2 x (1 + α1)))
```

The largest eigenvalue is the largest zero of the following polynomial (see above):

```
cpolT20P20noRsym[x_, s1_, α1_, m1_] := s1^2 (-1 + 2 x) (1 + α1) -
  (2 + α1 - 2 x (1 + α1))^2 - s1 (2 + α1 - 2 x (1 + α1))^2 - 2 m1 (s1^2 (-1 + x) (1 + α1) +
    (2 + α1) (-2 - α1 + 2 x (1 + α1)) + s1 (2 + α1) (-2 - α1 + 2 x (1 + α1)))
```

```
EVsT20P20[s1_, α1_, m1_, r_] := x /. NSolve[cpolT20P20noRsym[x, s1, α1, m1] == 0, x]
```

```
EVsT20P20[0.2, 0.1, 0.01, 0.5]
```

```
{0.869445, 1.03555}
```

The equilibrium p22 = 0 is stable in the blue region

```
RegionPlot[Abs[Max[EVsT20P20[10^s1, 10^a1, 0.01, 0.5]]] < 1,
 {s1, -3, 1}, {a1, -3, 4}, FrameLabel → {"Log10(s1)", "Log10(α1)"}]
```

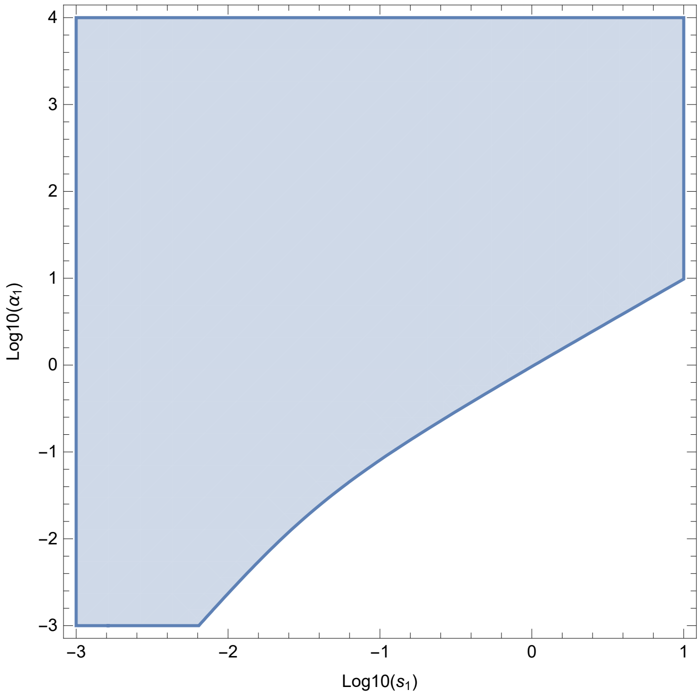

Display interval of p22 values on t12 = t22 = 0 edge that is stable

```
Simplify[cpolT20noR[x, p22] /. {α2 → α1, m2 → m1, s2 → s1}]
```

```
-m1^2 (s1 (1 + α1) + (2 + α1) (1 + p22 α1)) ((2 + α1) (1 + p22 α1) + s1 (1 + p22 α1 (2 + α1))) +
  (-2 + 2 x - α1 - 2 p22 α1 + 2 x α1 - p22 α1^2 + s1 (-1 + 2 x) (1 + α1) +
     m1 (s1 (1 + α1) + (2 + α1) (1 + p22 α1))) (-2 + 2 x - α1 - 2 p22 α1 + 2 x α1 - p22 α1^2 -
     s1 (1 + p22 α1 (2 + α1)) + m1 ((2 + α1) (1 + p22 α1) + s1 (1 + p22 α1 (2 + α1))))
```

```
cpolT20P2allnoRsym[p22_, x_, s1_, α1_, m1_] :=
 -m1^2 (s1 (1 + α1) + (2 + α1) (1 + p22 α1)) ((2 + α1) (1 + p22 α1) + s1 (1 + p22 α1 (2 + α1))) +
  (-2 + 2 x - α1 - 2 p22 α1 + 2 x α1 - p22 α1^2 + s1 (-1 + 2 x) (1 + α1) +
     m1 (s1 (1 + α1) + (2 + α1) (1 + p22 α1))) (-2 + 2 x - α1 - 2 p22 α1 + 2 x α1 - p22 α1^2 -
     s1 (1 + p22 α1 (2 + α1)) + m1 ((2 + α1) (1 + p22 α1) + s1 (1 + p22 α1 (2 + α1))))
```

```
EVsT20P2allnoRsym[p22_, s1_, α1_, m1_] :=
 x /. NSolve[cpolT20P2allnoRsym[p22, x, s1, α1, m1] == 0, x]
```

```
EVsT20P2allnoRsym[.1, 0.2, 3, 0.01]
```

```
{0.752229, 0.866834}
```

Below, the colored region is the region of stability

```
RegionPlot[Abs[Max[EVsT20P2allnoRsym[p22, 0.2, 10^a1, 0.01]]] < 1,
 {a1, -2, 3}, {p22, 0, 0.5}, FrameLabel → {"Log10(α₁)", "p22"}]
```

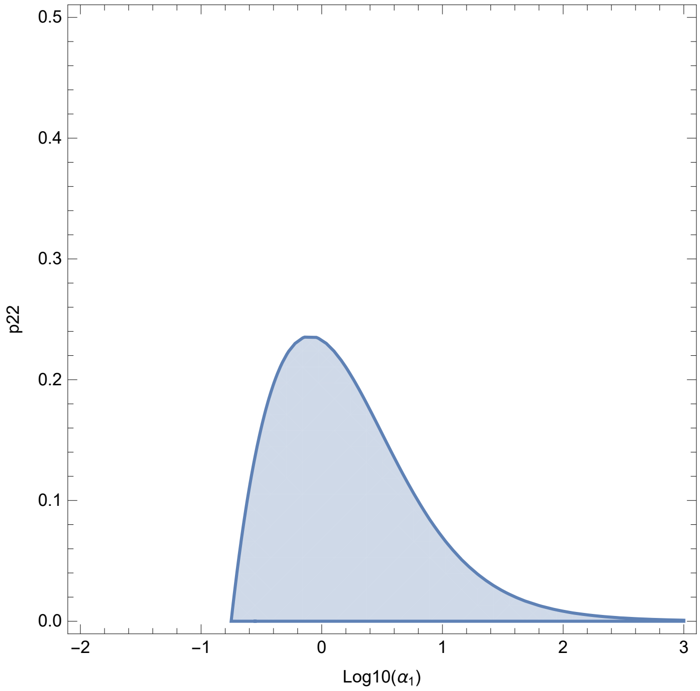

```
RegionPlot[{Abs[Max[EVsT20P2allnoRsym[p22, .05, 10^a1, 0.01]]] < 1,
  Abs[Max[EVsT20P2allnoRsym[p22, .2, 10^a1, 0.01]]] < 1,
  Abs[Max[EVsT20P2allnoRsym[p22, 1, 10^a1, 0.01]]] < 1},
 {a1, -2, 4}, {p22, 0, 0.4}, FrameLabel → {"Log10(α1)", "p22"}]
```

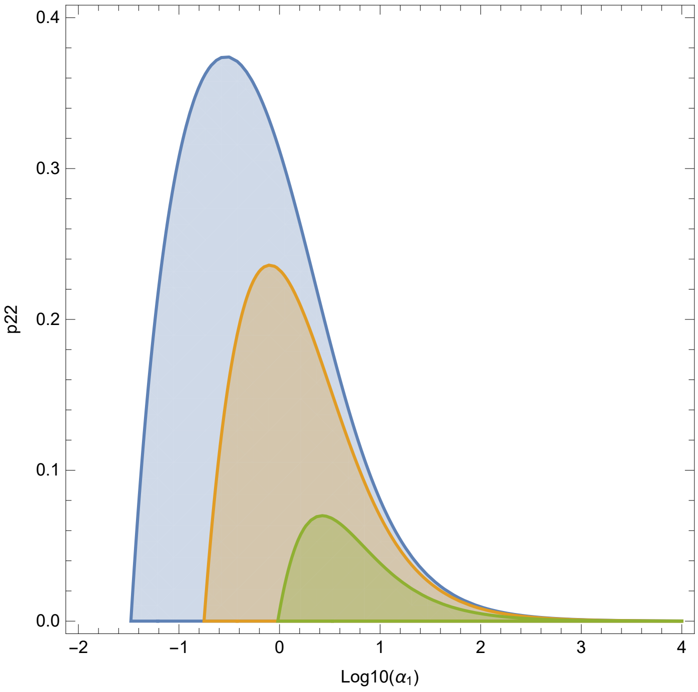

The region of stability decreases with increasing s

```
RegionPlot[{Abs[Max[EVsT20P2allnoRsym[p22, 0.2, 10^a1, 0.001]]] < 1,
  Abs[Max[EVsT20P2allnoRsym[p22, 0.2, 10^a1, 0.01]]] < 1,
  Abs[Max[EVsT20P2allnoRsym[p22, 0.2, 10^a1, 0.1]]] < 1},
 {a1, -2, 4}, {p22, 0, 0.4}, FrameLabel → {"Log10(α1)", "p22"}]
```

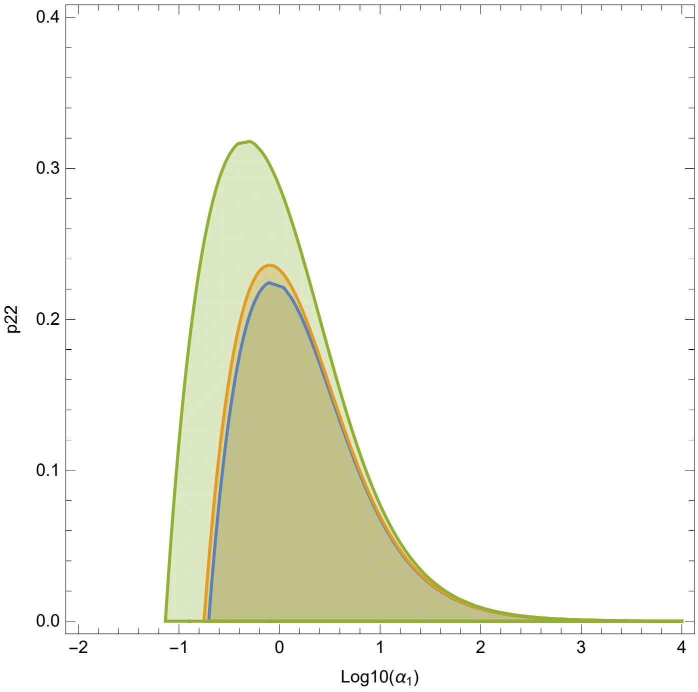

The region of stability increases with m!

Stability of equilibria at the edge t12 = t22 = 1 (only symmetric case)

An analogous analysis can be performed

```
JacoT21 =
  Simplify[D[itgenmale[s1, s2, α1, α2, m1, m2, r][{p12, t12, dd1, p22, t22, dd2}],
     {{p12, t12, dd1, p22, t22, dd2}}] /. {t12 → 1, dd1 → 0, t22 → 1, dd2 → 0, p12 → p22}]
```

```
{{1 - m1, 0, - (((-1 + m1) (p22 α2 + (-1 + p22) α1 (1 + α2) +
          s1 (-1 + (-1 + p22) α2 + (-1 + p22) α1 (1 + α2)))) / (2 (1 + α2))), m1, 0,
   (m1 (p22 α2 + (-1 + p22) α1 (1 + α2) + s1 (-1 + (-1 + p22) α2 + (-1 + p22) α1 (1 + α2)))) /
    (2 (1 + α2))},
 {0, ((-1 + m1) (-2 - 2 α2 + p22 α2 + (-1 + p22) α1 (1 + α2) +
        s1 (-1 + (-1 + p22) α2 + (-1 + p22) α1 (1 + α2)))) / (2 (1 + α2)),
  0, 0, - ((m1 (-2 - 2 α2 + p22 α2 + (-1 + p22) α1 (1 + α2) +
          s1 (-1 + (-1 + p22) α2 + (-1 + p22) α1 (1 + α2)))) / (2 (1 + α2))), 0},
 {0, - (((-1 + m1) (-1 + p22) p22 r (1 + s1) (α1 + α2 + α1 α2)) / (2 (1 + α2))),
  - (((-1 + m1) (-1 + r) (-2 - 2 α2 + p22 α2 + (-1 + p22) α1 (1 + α2) +
          s1 (-1 + (-1 + p22) α2 + (-1 + p22) α1 (1 + α2)))) / (2 (1 + α2))),
  0, (m1 (-1 + p22) p22 r (1 + s1) (α1 + α2 + α1 α2)) / (2 (1 + α2)),
  (m1 (-1 + r) (-2 - 2 α2 + p22 α2 + (-1 + p22) α1 (1 + α2) +
        s1 (-1 + (-1 + p22) α2 + (-1 + p22) α1 (1 + α2)))) / (2 (1 + α2))},
 {m2, 0, (m2 (p22 α2 + s2 (1 + α2) + (-1 + p22) α1 (1 + α2))) / (2 (1 + s2) (1 + α2)),
  1 - m2, 0,
  - (((-1 + m2) (p22 α2 + s2 (1 + α2) + (-1 + p22) α1 (1 + α2))) / (2 (1 + s2) (1 + α2)))},
 {0, (m2 (2 + 2 α2 - p22 α2 + s2 (1 + α2) - (-1 + p22) α1 (1 + α2))) / (2 (1 + s2) (1 + α2)), 0, 0,
  - (((-1 + m2) (2 + 2 α2 - p22 α2 + s2 (1 + α2) - (-1 + p22) α1 (1 + α2))) / (2 (1 + s2) (1 + α2))),
  0},
 {0, (m2 (-1 + p22) p22 r (α1 + α2 + α1 α2)) / (2 (1 + s2) (1 + α2)),
  - ((m2 (-1 + r) (2 + 2 α2 - p22 α2 + s2 (1 + α2) - (-1 + p22) α1 (1 + α2))) /
      (2 (1 + s2) (1 + α2))), 0,
  - (((-1 + m2) (-1 + p22) p22 r (α1 + α2 + α1 α2)) / (2 (1 + s2) (1 + α2))),
  ((-1 + m2) (-1 + r) (2 + 2 α2 - p22 α2 + s2 (1 + α2) - (-1 + p22) α1 (1 + α2))) /
   (2 (1 + s2) (1 + α2))}}
```

Characteristic polynomial at this equilibrium:

```
cpolT21 = Simplify[Det[JacoT21 - x IdentityMatrix[6]]]
```

```
              1
─────────────────────────────
16 (1 + s2)^2 (1 + α2)^4
 (-1 + x) (-1 + m1 + m2 + x) (m1 m2 (2 + 2 α2 - p22 α2 + s2 (1 + α2) - (-1 + p22) α1 (1 + α2)) (-2 -
        2 α2 + p22 α2 + (-1 + p22) α1 (1 + α2) + s1 (-1 + (-1 + p22) α2 + (-1 + p22) α1 (1 + α2))) +
     (-2 + 2 x - α1 + p22 α1 - 2 α2 + p22 α2 + 2 x α2 - α1 α2 + p22 α1 α2 + s2 (-1 + 2 x) (1 + α2) +
        m2 (2 + 2 α2 - p22 α2 + s2 (1 + α2) - (-1 + p22) α1 (1 + α2)))
      (-2 + 2 x - α1 + p22 α1 - 2 α2 + p22 α2 + 2 x α2 - α1 α2 + p22 α1 α2 +
        s1 (-1 + (-1 + p22) α2 + (-1 + p22) α1 (1 + α2)) + m1 (2 + 2 α2 - p22 α2 -
           (-1 + p22) α1 (1 + α2) + s1 (1 + α2 - p22 α2 - (-1 + p22) α1 (1 + α2)))))
  (m1 m2 (-1 + r)^2 (2 + 2 α2 - p22 α2 + s2 (1 + α2) - (-1 + p22) α1 (1 + α2)) (-2 - 2 α2 +
        p22 α2 + (-1 + p22) α1 (1 + α2) + s1 (-1 + (-1 + p22) α2 + (-1 + p22) α1 (1 + α2))) +
     (2 + s2 - 2 x - 2 s2 x + α1 - p22 α1 + 2 α2 - p22 α2 + s2 α2 - 2 x α2 - 2 s2 x α2 + α1 α2 -
        p22 α1 α2 + m2 (-1 + r) (2 + 2 α2 - p22 α2 + s2 (1 + α2) - (-1 + p22) α1 (1 + α2)) -
        r (2 + 2 α2 - p22 α2 + s2 (1 + α2) - (-1 + p22) α1 (1 + α2)))
      (2 + s1 - 2 x + α1 - p22 α1 + s1 α1 - p22 s1 α1 + 2 α2 - p22 α2 + s1 α2 - p22 s1 α2 - 2 x α2 +
        α1 α2 - p22 α1 α2 + s1 α1 α2 - p22 s1 α1 α2 - m1 (-1 + r) (-2 - 2 α2 + p22 α2 + (-1 + p22) α1
           (1 + α2) + s1 (-1 + (-1 + p22) α2 + (-1 + p22) α1 (1 + α2))) + r (-2 - 2 α2 + p22 α2 +
          (-1 + p22) α1 (1 + α2) + s1 (-1 + (-1 + p22) α2 + (-1 + p22) α1 (1 + α2)))))
```

There are four factors; x = 1 and x = 1 - m1 - m2 are always eigenvalues.
In addition, there is one factor without r, and one factor with r. Both factors are quadratic in x and given below:

```
cpolT21noR[x_, p22_] :=
  (m1 m2 (2 + 2 α2 - p22 α2 + s2 (1 + α2) - (-1 + p22) α1 (1 + α2)) (-2 - 2 α2 + p22 α2 +
        (-1 + p22) α1 (1 + α2) + s1 (-1 + (-1 + p22) α2 + (-1 + p22) α1 (1 + α2))) +
     (-2 + 2 x - α1 + p22 α1 - 2 α2 + p22 α2 + 2 x α2 - α1 α2 + p22 α1 α2 + s2 (-1 + 2 x) (1 + α2) +
        m2 (2 + 2 α2 - p22 α2 + s2 (1 + α2) - (-1 + p22) α1 (1 + α2)))
      (-2 + 2 x - α1 + p22 α1 - 2 α2 + p22 α2 + 2 x α2 - α1 α2 + p22 α1 α2 +
        s1 (-1 + (-1 + p22) α2 + (-1 + p22) α1 (1 + α2)) + m1 (2 + 2 α2 - p22 α2 -
           (-1 + p22) α1 (1 + α2) + s1 (1 + α2 - p22 α2 - (-1 + p22) α1 (1 + α2)))))
```

```
cpolT21R[x_, p22_] :=
  (m1 m2 (-1 + r)^2 (2 + 2 α2 - p22 α2 + s2 (1 + α2) - (-1 + p22) α1 (1 + α2)) (-2 - 2 α2 + p22 α2 +
        (-1 + p22) α1 (1 + α2) + s1 (-1 + (-1 + p22) α2 + (-1 + p22) α1 (1 + α2))) +
     (2 + s2 - 2 x - 2 s2 x + α1 - p22 α1 + 2 α2 - p22 α2 + s2 α2 - 2 x α2 - 2 s2 x α2 + α1 α2 -
        p22 α1 α2 + m2 (-1 + r) (2 + 2 α2 - p22 α2 + s2 (1 + α2) - (-1 + p22) α1 (1 + α2)) -
        r (2 + 2 α2 - p22 α2 + s2 (1 + α2) - (-1 + p22) α1 (1 + α2)))
      (2 + s1 - 2 x + α1 - p22 α1 + s1 α1 - p22 s1 α1 + 2 α2 - p22 α2 + s1 α2 - p22 s1 α2 - 2 x α2 +
        α1 α2 - p22 α1 α2 + s1 α1 α2 - p22 s1 α1 α2 - m1 (-1 + r) (-2 - 2 α2 + p22 α2 + (-1 + p22)
           α1 (1 + α2) + s1 (-1 + (-1 + p22) α2 + (-1 + p22) α1 (1 + α2))) + r (-2 - 2 α2 +
          p22 α2 + (-1 + p22) α1 (1 + α2) + s1 (-1 + (-1 + p22) α2 + (-1 + p22) α1 (1 + α2)))))
```

```
Simplify[Series[cpolT21R[x, p22], {x, 0, 3}]]
```

$$(-1+m1+m2)\,(-1+r)^2\,(2+2\,\alpha 2-p22\,\alpha 2+s2\,(1+\alpha 2)-(-1+p22)\,\alpha 1\,(1+\alpha 2))\,(-2-2\,\alpha 2+p22\,\alpha 2+(-1+p22)\,\alpha 1\,(1+\alpha 2)+s1\,(-1+(-1+p22)\,\alpha 2+(-1+p22)\,\alpha 1\,(1+\alpha 2)))+2\,(-1+r)\,(1+\alpha 2)\,(-(-1+m2)\,(2+2\,\alpha 2-p22\,\alpha 2+s2\,(1+\alpha 2)-(-1+p22)\,\alpha 1\,(1+\alpha 2))+(-1+m1)\,(1+s2)\,(-2-2\,\alpha 2+p22\,\alpha 2+(-1+p22)\,\alpha 1\,(1+\alpha 2)+s1\,(-1+(-1+p22)\,\alpha 2+(-1+p22)\,\alpha 1\,(1+\alpha 2))))\,x+4\,(1+s2)\,(1+\alpha 2)^2\,x^2+O[x]^4$$

```
Simplify[Series[cpolT21noR[x, p22], {x, 0, 3}]]
```

$$(-1+m1+m2)\,(2+2\,\alpha 2-p22\,\alpha 2+s2\,(1+\alpha 2)-(-1+p22)\,\alpha 1\,(1+\alpha 2))\,(-2-2\,\alpha 2+p22\,\alpha 2+(-1+p22)\,\alpha 1\,(1+\alpha 2)+s1\,(-1+(-1+p22)\,\alpha 2+(-1+p22)\,\alpha 1\,(1+\alpha 2)))+2\,(1+\alpha 2)\,((-1+m2)\,(2+2\,\alpha 2-p22\,\alpha 2+s2\,(1+\alpha 2)-(-1+p22)\,\alpha 1\,(1+\alpha 2))-(-1+m1)\,(1+s2)\,(-2-2\,\alpha 2+p22\,\alpha 2+(-1+p22)\,\alpha 1\,(1+\alpha 2)+s1\,(-1+(-1+p22)\,\alpha 2+(-1+p22)\,\alpha 1\,(1+\alpha 2))))\,x+4\,(1+s2)\,(1+\alpha 2)^2\,x^2+O[x]^4$$

Therefore, we find

```
Simplify[cpolT21R[x (1 - r), p22] - (1 - r)^2 cpolT21noR[x, p22]]
```

0

Therefore, cpolT21noR[x,p22] = 0 if and only if cpolT21R[(1-r)x,p22] = 0.
Hence, x is an eigenvalue resulting from cpolT21noR[x,p22] = 0 if and only if (1-r)x is an eigenvalue resulting from cpolT21R[x,p22] = 0.

As a consequence, an equilibrium p22 (=p12) at this edge (t12=t22=1, dd1=dd2=0) is unstable if and only if there exists and eigenvalue x>1 with cpolT21noR[x,p22] = 0.
Thus, a change of stability at this edge occurs at a point p22 if cpolT20noR[1,p22] = 0.

The curve of equilibria that connects the edges t12=t22=0 and t12=t22=1 has its endpoint at the value p22, at which the stability at this edge changes, i.e., where cpolT21noR[1,p22] = 0.
This is easily computed numerically.

Display interval of stability at the edge t12=t22=1

```
Simplify[cpolT21noR[x, p22] /. {α2 → α1, s2 → s1, m2 → m1}]
```

$$m1^2\,(s1\,(1+\alpha 1)-(2+\alpha 1)\,(-1+(-1+p22)\,\alpha 1))\,((2+\alpha 1)\,(-1+(-1+p22)\,\alpha 1)+s1\,(-1+2\,(-1+p22)\,\alpha 1+(-1+p22)\,\alpha 1^2))+(-2+2\,x-3\,\alpha 1+2\,p22\,\alpha 1+2\,x\,\alpha 1-\alpha 1^2+p22\,\alpha 1^2+m1\,s1\,(1+\alpha 1)+s1\,(-1+2\,x)\,(1+\alpha 1)-m1\,(2+\alpha 1)\,(-1+(-1+p22)\,\alpha 1))\,(-2+2\,x-3\,\alpha 1+2\,p22\,\alpha 1+2\,x\,\alpha 1-\alpha 1^2+p22\,\alpha 1^2+s1\,(-1+2\,(-1+p22)\,\alpha 1+(-1+p22)\,\alpha 1^2)+m1\,(-(2+\alpha 1)\,(-1+(-1+p22)\,\alpha 1)+s1\,(1-2\,(-1+p22)\,\alpha 1-(-1+p22)\,\alpha 1^2)))$$

```
cpolT21P2allnoRsym[p22_, x_, s1_, α1_, m1_] :=
 m1^2 (s1 (1 + α1) - (2 + α1) (-1 + (-1 + p22) α1))
   ((2 + α1) (-1 + (-1 + p22) α1) + s1 (-1 + 2 (-1 + p22) α1 + (-1 + p22) α1^2)) +
  (-2 + 2 x - 3 α1 + 2 p22 α1 + 2 x α1 - α1^2 + p22 α1^2 + m1 s1 (1 + α1) +
     s1 (-1 + 2 x) (1 + α1) - m1 (2 + α1) (-1 + (-1 + p22) α1)) (-2 + 2 x - 3 α1 +
     2 p22 α1 + 2 x α1 - α1^2 + p22 α1^2 + s1 (-1 + 2 (-1 + p22) α1 + (-1 + p22) α1^2) +
     m1 (-(2 + α1) (-1 + (-1 + p22) α1) + s1 (1 - 2 (-1 + p22) α1 - (-1 + p22) α1^2)))
```

```
EVsT21P2allnoRsym[p22_, s1_, α1_, m1_] :=
 x /. NSolve[cpolT21P2allnoRsym[p22, x, s1, α1, m1] == 0, x]
```

```
EVsT21P2allnoRsym[.1, 0.2, 3, 0.01]
```

```
{1.9895, 2.64907}
```

Below, the colored region is the region of stability

```
RegionPlot[Abs[Max[EVsT21P2allnoRsym[p22, 0.2, 10^a1, 0.01]]] < 1,
 {a1, -2, 3}, {p22, 0.7, 1}, FrameLabel → {"Log10(α1)", "p22"}]
```

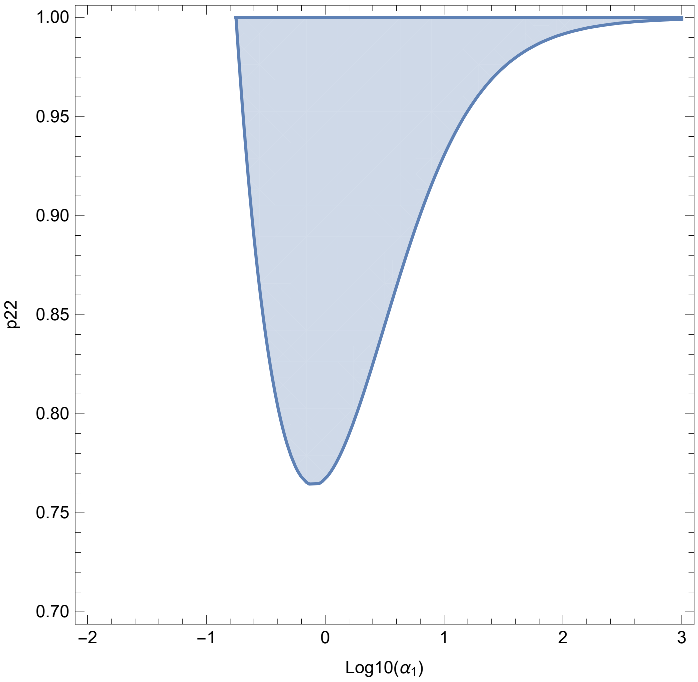

```
RegionPlot[{Abs[Max[EVsT21P2allnoRsym[p22, .05, 10^a1, 0.01]]] < 1,
  Abs[Max[EVsT21P2allnoRsym[p22, .2, 10^a1, 0.01]]] < 1,
  Abs[Max[EVsT21P2allnoRsym[p22, 1, 10^a1, 0.01]]] < 1},
 {a1, -2, 4}, {p22, 0.6, 1}, FrameLabel → {"Log10(α1)", "p22"}]
```

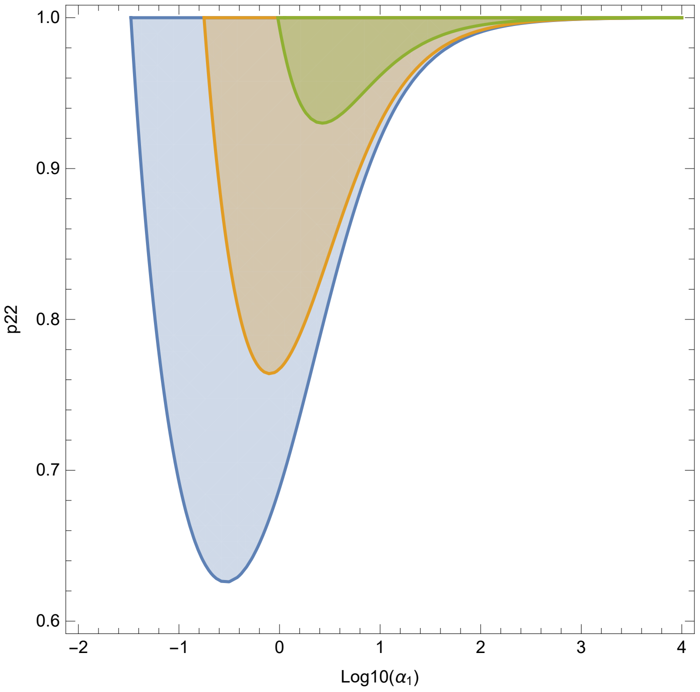

Note the 1-p22 gives the corresponding interval at t12 = t22 = 0 (because of symmetry)

2.2 General equilibrium conditions and computation of polymorphic equilibria

2.2.1 Basic definition

Solving deltagenmale = 0 yields the potential equilibria (conditional on admissibility)

```
deltagenmale =
 Simplify[itgenmale[s1, s2, α1, α2, m1, m2, r][{p12, t12, dd1, p22, t22, dd2}] -
   {p12, t12, dd1, p22, t22, dd2}]
```

```
deltagenmale =
  {-p12 + (dd1 (s1 + α1 + s1 α1) (-1 + s1 (-1 + t12) - t12 α2) + (-1 + m1)^2 m1 p12^2 (1 + s1)
        (t12 - t22) (α1 + α2 + α1 α2) + m1^3 p22 (t12 - t22) (s1^2 (t12 - t22) (1 + α1) +
          (-t12 + t22) α1 α2 + s1 (-t12 + t22) (α1 (-1 + α2) + α2) + p22 (α1 + α2 + α1 α2) +
         p22 s1 (α1 + α2 + α1 α2)) + m1 ((s1 + α1 + s1 α1) (dd2 (-1 + s1 (-1 + t12) -
               t12 α2) + dd1 (1 + s1 (1 - 2 t12 + t22) + 2 t12 α2 - t22 α2)) +
         p22 (2 + s1^2 (-1 + t12) (-2 + t12 + t22) (1 + α1) + dd1 α2 + 2 t12 α2 +
            dd1 α1 (1 + α2) - (-2 + t12 + t22) α1 (1 + t12 α2) + s1 (4 - t22 + 4 α1 +
               dd1 α1 - 2 t22 α1 + dd1 α2 + dd1 α1 α2 - t12^2 (α1 (-1 + α2) + α2) -
               t12 (3 + α1 (4 + t22 (-1 + α2) - 2 α2) + (-2 + t22) α2)))) +
      m1^2 ((dd1 - dd2) (t12 - t22) (s1 + α1 + s1 α1) (s1 - α2) - p22^2 (1 + s1)
```

```
(t12 - t22) (α1 + α2 + α1 α2) + p22 (-dd1 α1 + dd2 α1 + t12 α1 -
   t22 α1 - s1^2 (2 t12^2 - (-3 + t22) t22 - t12 (3 + t22)) (1 + α1) -
   dd1 α2 + dd2 α2 - 2 t12 α2 + 2 t22 α2 - dd1 α1 α2 + dd2 α1 α2 -
   2 t12 α1 α2 + 2 t12^2 α1 α2 + 2 t22 α1 α2 - t12 t22 α1 α2 - t22^2 α1 α2 +
   s1 (2 t12^2 (α1 (-1 + α2) + α2) - t22^2 (α1 (-1 + α2) + α2) +
      t22 (-3 + 2 α1 (-2 + α2) + 2 α2) - t12 (-3 + 2 α1 (-2 + α2) +
         t22 α1 (-1 + α2) + (2 + t22) α2) - (dd1 - dd2) (α1 + α2 + α1 α2)))) -
 (-1 + m1) p12 (2 + 2 α1 + dd1 α1 - dd1 m1 α1 + dd2 m1 α1 - 2 t12 α1 + m1 t12 α1 -
   m1 p22 t12 α1 + 2 m1^2 p22 t12 α1 - m1 t22 α1 + m1 p22 t22 α1 -
   2 m1^2 p22 t22 α1 + s1^2 (2 + (2 - 3 m1 + m1^2) t12^2 - 3 m1 t22 + m1^2 t22^2 +
      t12 (-4 - 2 m1^2 t22 + 3 m1 (1 + t22))) (1 + α1) + dd1 α2 - dd1 m1 α2 +
   dd2 m1 α2 + 2 t12 α2 - 2 m1 t12 α2 - m1 p22 t12 α2 + 2 m1^2 p22 t12 α2 +
   2 m1 t22 α2 + m1 p22 t22 α2 - 2 m1^2 p22 t22 α2 + dd1 α1 α2 - dd1 m1 α1 α2 +
   dd2 m1 α1 α2 + 2 t12 α1 α2 - 2 m1 t12 α1 α2 - m1 p22 t12 α1 α2 + 2 m1^2 p22 t12 α1 α2 -
   2 t12^2 α1 α2 + 3 m1 t12^2 α1 α2 - m1^2 t12^2 α1 α2 + 2 m1 t22 α1 α2 + m1 p22 t22 α1 α2 -
   2 m1^2 p22 t22 α1 α2 - 3 m1 t12 t22 α1 α2 + 2 m1^2 t12 t22 α1 α2 - m1^2 t22^2 α1 α2 +
   s1 (4 + 4 α1 + dd1 α1 + dd1 α2 + dd1 α1 α2 - (2 - 3 m1 + m1^2) t12^2 (α1 (-1 + α2) + α2) -
      m1^2 t22 (t22 (α1 (-1 + α2) + α2) + 2 p22 (α1 + α2 + α1 α2)) +
      m1 ((-dd1 + dd2) (α1 + α2 + α1 α2) + t22 (-3 + (2 + p22) α2 + α1 (-4 + p22 +
                 2 α2 + p22 α2))) + t12 (2 (-2 + α1 (-3 + α2) + α2) - m1 (-3 +
               (2 + p22 + 3 t22) α2 + α1 (-4 + p22 - 3 t22 + 2 α2 + p22 α2 + 3 t22 α2)) +
         2 m1^2 (t22 (α1 (-1 + α2) + α2) + p22 (α1 + α2 + α1 α2))))))) /
 (2 (1 + (1 + (-1 + m1) t12 - m1 t22) α1 + s1 (1 + (-1 + m1) t12 - m1 t22) (1 + α1))
   (1 + s1 + (-1 + m1) s1 t12 - m1 s1 t22 + m1 t22 α2 + t12 (α2 - m1 α2))),
-t12 - (((-1 + m1) t12 - m1 t22) (2 + s1^2 (1 + (-1 + m1) t12 - m1 t22)^2 (1 + α1) +
      p12 α2 - m1 p12 α2 + m1 p22 α2 + 2 t12 α2 - 2 m1 t12 α2 -
      p12 t12 α2 + 2 m1 p12 t12 α2 - m1^2 p12 t12 α2 - m1 p22 t12 α2 +
      m1^2 p22 t12 α2 + 2 m1 t22 α2 - m1 p12 t22 α2 + m1^2 p12 t22 α2 -
      m1^2 p22 t22 α2 - (1 + (-1 + m1) t12 - m1 t22) α1
       (-1 - t12 α2 + (-1 + m1) p12 (1 + α2) - m1 (p22 + p22 α2 - t12 α2 + t22 α2)) -
      s1 (1 + (-1 + m1) t12 - m1 t22) (-3 - p12 α2 + m1 p12 α2 - m1 p22 α2 -
         t12 α2 + m1 t12 α2 - m1 t22 α2 + α1 (-2 + t12 - t12 α2 + (-1 + m1) p12
               (1 + α2) - m1 (p22 + t12 - t22 + p22 α2 - t12 α2 + t22 α2)))))) /
 (2 (1 + (1 + (-1 + m1) t12 - m1 t22) α1 + s1 (1 + (-1 + m1) t12 - m1 t22) (1 + α1))
   (1 + s1 + (-1 + m1) s1 t12 - m1 s1 t22 + m1 t22 α2 + t12 (α2 - m1 α2))), 1/4
 (-4 dd1 - (-(((dd1 (-1 + m1) - dd2 m1 - p12 t12 + m1 p12 t12 - m1 p22 t22) (dd1 (-1 + m1) -
              dd2 m1 + t12 - m1 t12 - p12 t12 + m1 p12 t12 + m1 t22 - m1 p22 t22)) /
          (1 + (1 + (-1 + m1) t12 - m1 t22) α1 + s1 (1 + (-1 + m1) t12 - m1 t22) (1 + α1))) +
      (r (dd1 (-1 + m1) - dd2 m1 - p12 t12 + m1 p12 t12 - m1 p22 t22)
          (-1 + dd1 (-1 + m1) - dd2 m1 + p12 - m1 p12 + m1 p22 + t12 -
            m1 t12 - p12 t12 + m1 p12 t12 + m1 t22 - m1 p22 t22)) /
       (1 + (1 + (-1 + m1) t12 - m1 t22) α1 + s1 (1 + (-1 + m1) t12 - m1 t22) (1 + α1)) -
      ((dd1 (-1 + m1) - dd2 m1 + t12 - m1 t12 - p12 t12 + m1 p12 t12 + m1 t22 - m1 p22 t22)
          (-1 + dd1 (-1 + m1) - dd2 m1 + p12 - m1 p12 + m1 p22 + t12 -
            m1 t12 - p12 t12 + m1 p12 t12 + m1 t22 - m1 p22 t22)) /
       (1 + (1 + (-1 + m1) t12 - m1 t22) α1 + s1 (1 + (-1 + m1) t12 - m1 t22) (1 + α1)) +
      (2 (dd1 - dd1 m1 + dd2 m1 - t12 + m1 t12 + p12 t12 -
            m1 p12 t12 - m1 t22 + m1 p22 t22)^2) /
```

```
      (1 + (1 + (-1 + m1) t12 - m1 t22) α1 + s1 (1 + (-1 + m1) t12 - m1 t22) (1 + α1)) -
    ((-1 + r) (1 + s1) (dd1 (-1 + m1) - dd2 m1 + p12 - m1 p12 + m1 p22 -
          p12 t12 + m1 p12 t12 - m1 p22 t22) (dd1 (-1 + m1) - dd2 m1 + t12 -
          m1 t12 - p12 t12 + m1 p12 t12 + m1 t22 - m1 p22 t22) (1 + α1)) /
      (1 + (1 + (-1 + m1) t12 - m1 t22) α1 + s1 (1 + (-1 + m1) t12 - m1 t22) (1 + α1)) -
    ((1 + s1) (dd1 (-1 + m1) - dd2 m1 + t12 - m1 t12 - p12 t12 + m1 p12 t12 +
          m1 t22 - m1 p22 t22) (-1 + dd1 (-1 + m1) - dd2 m1 + p12 - m1 p12 + m1 p22 +
          t12 - m1 t12 - p12 t12 + m1 p12 t12 + m1 t22 - m1 p22 t22) (1 + α1)) /
      (1 + (1 + (-1 + m1) t12 - m1 t22) α1 + s1 (1 + (-1 + m1) t12 - m1 t22) (1 + α1)) +
    ((dd1 (-1 + m1) - dd2 m1 - p12 t12 + m1 p12 t12 - m1 p22 t22) (dd1 (-1 + m1) -
          dd2 m1 + t12 - m1 t12 - p12 t12 + m1 p12 t12 + m1 t22 - m1 p22 t22) (1 + α2)) /
      (-1 + s1 (-1 + t12 - m1 t12 + m1 t22) + (-1 + m1) t12 α2 - m1 t22 α2) +
    ((-1 + r) (dd1 (-1 + m1) - dd2 m1 + p12 - m1 p12 + m1 p22 - p12 t12 +
          m1 p12 t12 - m1 p22 t22) (dd1 (-1 + m1) - dd2 m1 + t12 -
          m1 t12 - p12 t12 + m1 p12 t12 + m1 t22 - m1 p22 t22) (1 + α2)) /
      (-1 + s1 (-1 + t12 - m1 t12 + m1 t22) + (-1 + m1) t12 α2 - m1 t22 α2) +
    (r (1 + s1) (dd1 (-1 + m1) - dd2 m1 - p12 t12 + m1 p12 t12 - m1 p22 t22)
        (-1 + dd1 (-1 + m1) - dd2 m1 + p12 - m1 p12 + m1 p22 + t12 -
          m1 t12 - p12 t12 + m1 p12 t12 + m1 t22 - m1 p22 t22)) /
      (1 + s1 + (-1 + m1) s1 t12 - m1 s1 t22 + m1 t22 α2 + t12 (α2 - m1 α2)))
  ((r (dd1 (-1 + m1) - dd2 m1 - p12 t12 + m1 p12 t12 - m1 p22 t22)
        (-1 + dd1 (-1 + m1) - dd2 m1 + p12 - m1 p12 + m1 p22 + t12 -
          m1 t12 - p12 t12 + m1 p12 t12 + m1 t22 - m1 p22 t22)) /
      (1 + (1 + (-1 + m1) t12 - m1 t22) α1 + s1 (1 + (-1 + m1) t12 - m1 t22) (1 + α1)) -
    ((-1 + r) (1 + s1) (dd1 (-1 + m1) - dd2 m1 + p12 - m1 p12 + m1 p22 -
          p12 t12 + m1 p12 t12 - m1 p22 t22) (dd1 (-1 + m1) - dd2 m1 + t12 -
          m1 t12 - p12 t12 + m1 p12 t12 + m1 t22 - m1 p22 t22) (1 + α1)) /
      (1 + (1 + (-1 + m1) t12 - m1 t22) α1 + s1 (1 + (-1 + m1) t12 - m1 t22) (1 + α1)) -
    ((1 + s1) (dd1 (-1 + m1) - dd2 m1 + p12 - m1 p12 + m1 p22 - p12 t12 + m1 p12 t12 -
          m1 p22 t22) (-1 + dd1 (-1 + m1) - dd2 m1 + p12 - m1 p12 + m1 p22 +
          t12 - m1 t12 - p12 t12 + m1 p12 t12 + m1 t22 - m1 p22 t22) (1 + α1)) /
      (1 + (1 + (-1 + m1) t12 - m1 t22) α1 + s1 (1 + (-1 + m1) t12 - m1 t22) (1 + α1)) +
    ((dd1 (-1 + m1) - dd2 m1 - p12 t12 + m1 p12 t12 - m1 p22 t22) (dd1 (-1 + m1) -
          dd2 m1 + p12 - m1 p12 + m1 p22 - p12 t12 + m1 p12 t12 - m1 p22 t22) (1 + α2)) /
      (-1 + s1 (-1 + t12 - m1 t12 + m1 t22) + (-1 + m1) t12 α2 - m1 t22 α2) +
    ((-1 + r) (dd1 (-1 + m1) - dd2 m1 + p12 - m1 p12 + m1 p22 - p12 t12 +
          m1 p12 t12 - m1 p22 t22) (dd1 (-1 + m1) - dd2 m1 + t12 -
          m1 t12 - p12 t12 + m1 p12 t12 + m1 t22 - m1 p22 t22) (1 + α2)) /
      (-1 + s1 (-1 + t12 - m1 t12 + m1 t22) + (-1 + m1) t12 α2 - m1 t22 α2) -
    ((1 + s1) (dd1 (-1 + m1) - dd2 m1 - p12 t12 + m1 p12 t12 - m1 p22 t22) (dd1 (-1 + m1) -
          dd2 m1 + p12 - m1 p12 + m1 p22 - p12 t12 + m1 p12 t12 - m1 p22 t22)) /
      (1 + s1 + (-1 + m1) s1 t12 - m1 s1 t22 + m1 t22 α2 + t12 (α2 - m1 α2)) +
    (r (1 + s1) (dd1 (-1 + m1) - dd2 m1 - p12 t12 + m1 p12 t12 - m1 p22 t22)
        (-1 + dd1 (-1 + m1) - dd2 m1 + p12 - m1 p12 + m1 p22 + t12 -
          m1 t12 - p12 t12 + m1 p12 t12 + m1 t22 - m1 p22 t22)) /
      (1 + s1 + (-1 + m1) s1 t12 - m1 s1 t22 + m1 t22 α2 + t12 (α2 - m1 α2)) -
    ((1 + s1) (dd1 (-1 + m1) - dd2 m1 + p12 - m1 p12 + m1 p22 - p12 t12 +
          m1 p12 t12 - m1 p22 t22) (-1 + dd1 (-1 + m1) - dd2 m1 + p12 - m1 p12 +
          m1 p22 + t12 - m1 t12 - p12 t12 + m1 p12 t12 + m1 t22 - m1 p22 t22)) /
      (1 + s1 + (-1 + m1) s1 t12 - m1 s1 t22 + m1 t22 α2 + t12 (α2 - m1 α2)) +
```

```
(2 (1 + s1) (dd1 - dd1 m1 + dd2 m1 - p12 + m1 p12 - m1 p22 +
      p12 t12 - m1 p12 t12 + m1 p22 t22)^2) /
   (1 + s1 + (-1 + m1) s1 t12 - m1 s1 t22 + m1 t22 α2 + t12 (α2 - m1 α2))) +
(-(((-1 + r) (dd1 (-1 + m1) - dd2 m1 - p12 t12 + m1 p12 t12 - m1 p22 t22)
        (-1 + dd1 (-1 + m1) - dd2 m1 + p12 - m1 p12 + m1 p22 + t12 -
          m1 t12 - p12 t12 + m1 p12 t12 + m1 t22 - m1 p22 t22)) /
      (1 + (1 + (-1 + m1) t12 - m1 t22) α1 + s1 (1 + (-1 + m1) t12 - m1 t22) (1 + α1))) -
   ((dd1 (-1 + m1) - dd2 m1 + t12 - m1 t12 - p12 t12 + m1 p12 t12 + m1 t22 - m1 p22 t22)
      (-1 + dd1 (-1 + m1) - dd2 m1 + p12 - m1 p12 + m1 p22 + t12 -
        m1 t12 - p12 t12 + m1 p12 t12 + m1 t22 - m1 p22 t22)) /
    (1 + (1 + (-1 + m1) t12 - m1 t22) α1 + s1 (1 + (-1 + m1) t12 - m1 t22) (1 + α1)) +
   (r (1 + s1) (dd1 (-1 + m1) - dd2 m1 + p12 - m1 p12 + m1 p22 - p12 t12 +
        m1 p12 t12 - m1 p22 t22) (dd1 (-1 + m1) - dd2 m1 + t12 -
        m1 t12 - p12 t12 + m1 p12 t12 + m1 t22 - m1 p22 t22) (1 + α1)) /
    (1 + (1 + (-1 + m1) t12 - m1 t22) α1 + s1 (1 + (-1 + m1) t12 - m1 t22) (1 + α1)) -
   ((1 + s1) (dd1 (-1 + m1) - dd2 m1 + p12 - m1 p12 + m1 p22 - p12 t12 + m1 p12 t12 -
        m1 p22 t22) (-1 + dd1 (-1 + m1) - dd2 m1 + p12 - m1 p12 + m1 p22 +
        t12 - m1 t12 - p12 t12 + m1 p12 t12 + m1 t22 - m1 p22 t22) (1 + α1)) /
    (1 + (1 + (-1 + m1) t12 - m1 t22) α1 + s1 (1 + (-1 + m1) t12 - m1 t22) (1 + α1)) -
   ((1 + s1) (dd1 (-1 + m1) - dd2 m1 + t12 - m1 t12 - p12 t12 + m1 p12 t12 +
        m1 t22 - m1 p22 t22) (-1 + dd1 (-1 + m1) - dd2 m1 + p12 - m1 p12 + m1 p22 +
        t12 - m1 t12 - p12 t12 + m1 p12 t12 + m1 t22 - m1 p22 t22) (1 + α1)) /
    (1 + (1 + (-1 + m1) t12 - m1 t22) α1 + s1 (1 + (-1 + m1) t12 - m1 t22) (1 + α1)) +
   (2 (1 + s1) (-1 + dd1 (-1 + m1) - dd2 m1 + p12 - m1 p12 + m1 p22 + t12 -
         m1 t12 - p12 t12 + m1 p12 t12 + m1 t22 - m1 p22 t22)^2 (1 + α1)) /
    (1 + (1 + (-1 + m1) t12 - m1 t22) α1 + s1 (1 + (-1 + m1) t12 - m1 t22) (1 + α1)) -
   ((-1 + r) (1 + s1) (dd1 (-1 + m1) - dd2 m1 - p12 t12 + m1 p12 t12 - m1 p22 t22)
      (-1 + dd1 (-1 + m1) - dd2 m1 + p12 - m1 p12 + m1 p22 + t12 -
        m1 t12 - p12 t12 + m1 p12 t12 + m1 t22 - m1 p22 t22)) /
    (1 + s1 + (-1 + m1) s1 t12 - m1 s1 t22 + m1 t22 α2 + t12 (α2 - m1 α2)) -
   ((1 + s1) (dd1 (-1 + m1) - dd2 m1 + p12 - m1 p12 + m1 p22 - p12 t12 +
        m1 p12 t12 - m1 p22 t22) (-1 + dd1 (-1 + m1) - dd2 m1 + p12 - m1 p12 +
        m1 p22 + t12 - m1 t12 - p12 t12 + m1 p12 t12 + m1 t22 - m1 p22 t22)) /
    (1 + s1 + (-1 + m1) s1 t12 - m1 s1 t22 + m1 t22 α2 + t12 (α2 - m1 α2)) +
   (r (dd1 (-1 + m1) - dd2 m1 + p12 - m1 p12 + m1 p22 - p12 t12 + m1 p12 t12 - m1 p22 t22)
      (dd1 (-1 + m1) - dd2 m1 + t12 - m1 t12 - p12 t12 + m1 p12 t12 + m1 t22 - m1 p22 t22)
      (1 + α2)) / (1 + s1 + (-1 + m1) s1 t12 - m1 s1 t22 + m1 t22 α2 + t12 (α2 - m1 α2)))
 (-(((dd1 (-1 + m1) - dd2 m1 - p12 t12 + m1 p12 t12 - m1 p22 t22) (dd1 (-1 + m1) -
          dd2 m1 + t12 - m1 t12 - p12 t12 + m1 p12 t12 + m1 t22 - m1 p22 t22)) /
      (1 + (1 + (-1 + m1) t12 - m1 t22) α1 + s1 (1 + (-1 + m1) t12 - m1 t22) (1 + α1))) -
   ((-1 + r) (dd1 (-1 + m1) - dd2 m1 - p12 t12 + m1 p12 t12 - m1 p22 t22)
      (-1 + dd1 (-1 + m1) - dd2 m1 + p12 - m1 p12 + m1 p22 + t12 -
        m1 t12 - p12 t12 + m1 p12 t12 + m1 t22 - m1 p22 t22)) /
    (1 + (1 + (-1 + m1) t12 - m1 t22) α1 + s1 (1 + (-1 + m1) t12 - m1 t22) (1 + α1)) +
   (r (1 + s1) (dd1 (-1 + m1) - dd2 m1 + p12 - m1 p12 + m1 p22 - p12 t12 +
        m1 p12 t12 - m1 p22 t22) (dd1 (-1 + m1) - dd2 m1 + t12 -
        m1 t12 - p12 t12 + m1 p12 t12 + m1 t22 - m1 p22 t22) (1 + α1)) /
    (1 + (1 + (-1 + m1) t12 - m1 t22) α1 + s1 (1 + (-1 + m1) t12 - m1 t22) (1 + α1)) +
```

```
((dd1 (-1 + m1) - dd2 m1 - p12 t12 + m1 p12 t12 - m1 p22 t22) (dd1 (-1 + m1) -
      dd2 m1 + p12 - m1 p12 + m1 p22 - p12 t12 + m1 p12 t12 - m1 p22 t22) (1 + α2)) /
  (-1 + s1 (-1 + t12 - m1 t12 + m1 t22) + (-1 + m1) t12 α2 - m1 t22 α2) +
 ((dd1 (-1 + m1) - dd2 m1 - p12 t12 + m1 p12 t12 - m1 p22 t22) (dd1 (-1 + m1) -
      dd2 m1 + t12 - m1 t12 - p12 t12 + m1 p12 t12 + m1 t22 - m1 p22 t22) (1 + α2)) /
  (-1 + s1 (-1 + t12 - m1 t12 + m1 t22) + (-1 + m1) t12 α2 - m1 t22 α2) -
 ((1 + s1) (dd1 (-1 + m1) - dd2 m1 - p12 t12 + m1 p12 t12 - m1 p22 t22) (dd1 (-1 + m1) -
      dd2 m1 + p12 - m1 p12 + m1 p22 - p12 t12 + m1 p12 t12 - m1 p22 t22)) /
  (1 + s1 + (-1 + m1) s1 t12 - m1 s1 t22 + m1 t22 α2 + t12 (α2 - m1 α2)) -
 ((-1 + r) (1 + s1) (dd1 (-1 + m1) - dd2 m1 - p12 t12 + m1 p12 t12 - m1 p22 t22)
    (-1 + dd1 (-1 + m1) - dd2 m1 + p12 - m1 p12 + m1 p22 + t12 -
      m1 t12 - p12 t12 + m1 p12 t12 + m1 t22 - m1 p22 t22)) /
  (1 + s1 + (-1 + m1) s1 t12 - m1 s1 t22 + m1 t22 α2 + t12 (α2 - m1 α2)) +
 (r (dd1 (-1 + m1) - dd2 m1 + p12 - m1 p12 + m1 p22 - p12 t12 + m1 p12 t12 - m1 p22 t22)
    (dd1 (-1 + m1) - dd2 m1 + t12 - m1 t12 - p12 t12 + m1 p12 t12 + m1 t22 - m1 p22 t22)
    (1 + α2)) / (1 + s1 + (-1 + m1) s1 t12 - m1 s1 t22 + m1 t22 α2 + t12 (α2 - m1 α2)) +
 (2 (dd1 - dd1 m1 + dd2 m1 + p12 t12 - m1 p12 t12 + m1 p22 t22)^2 (1 + α2)) /
  (1 + s1 + (-1 + m1) s1 t12 - m1 s1 t22 + m1 t22 α2 + t12 (α2 - m1 α2)))),
-p22 + (dd2 (s2 - α1) (1 + t22 α2 + s2 t22 (1 + α2)) - m2^3 (p12 - p22)
    (t12 - t22) (p12 α1 - p22 α1 + p12 α2 - p22 α2 + p12 α1 α2 -
      p22 α1 α2 + t12 α1 α2 - t22 α1 α2 - s2^2 (t12 - t22) (1 + α2) +
      s2 ((t12 - t22) (α1 - α2 + α1 α2) + p12 (α1 + α2 + α1 α2) - p22 (α1 + α2 + α1 α2))) +
   p22 (2 + dd2 α2 + 2 t22 α2 + 2 s2^2 t22^2 (1 + α2) + dd2 α1 (1 + α2) -
      2 (-1 + t22) α1 (1 + t22 α2) +
      s2 (-2 t22^2 (α1 - α2 + α1 α2) + dd2 (α1 + α2 + α1 α2) + 2 t22 (2 + α1 + α2 + α1 α2))) +
   m2^2 ((dd1 - dd2) (t12 - t22) (s2 - α1) (s2 + α2 + s2 α2) + p12^2 (1 + s2)
       (t12 - t22) (α1 + α2 + α1 α2) + 2 p22^2 (1 + s2) (t12 - t22) (α1 + α2 + α1 α2) +
      p12 (dd1 α1 - dd2 α1 - t12 α1 - 3 p22 t12 α1 + t22 α1 + 3 p22 t22 α1 + dd1 α2 -
         dd2 α2 + 2 t12 α2 - 3 p22 t12 α2 - 2 t22 α2 + 3 p22 t22 α2 + dd1 α1 α2 - dd2 α1 α2 +
         2 t12 α1 α2 - 3 p22 t12 α1 α2 - t12^2 α1 α2 - 2 t22 α1 α2 + 3 p22 t22 α1 α2 -
         t12 t22 α1 α2 + 2 t22^2 α1 α2 + s2^2 (t12^2 + t12 t22 - 2 t22^2) (1 + α2) -
         s2 (t12^2 (α1 - α2 + α1 α2) - 2 t22^2 (α1 - α2 + α1 α2) - (dd1 - dd2)
             (α1 + α2 + α1 α2) + t22 (3 + (2 - 3 p22) α2 - (-2 + 3 p22) α1 (1 + α2)) +
            t12 (-3 + (-2 + 3 p22 - t22) α2 + (-2 + 3 p22 + t22) α1 (1 + α2)))) +
      p22 (-dd1 α1 + dd2 α1 + t12 α1 - t22 α1 - dd1 α2 + dd2 α2 - 2 t12 α2 +
         2 t22 α2 - dd1 α1 α2 + dd2 α1 α2 - 2 t12 α1 α2 - t12^2 α1 α2 + 2 t22 α1 α2 +
         5 t12 t22 α1 α2 - 4 t22^2 α1 α2 + s2^2 (t12^2 - 5 t12 t22 + 4 t22^2) (1 + α2) -
         s2 (t12^2 (α1 - α2 + α1 α2) + 4 t22^2 (α1 - α2 + α1 α2) +
            (dd1 - dd2) (α1 + α2 + α1 α2) - t22 (3 + 2 α2 + 2 α1 (1 + α2)) +
            t12 (3 + 2 α2 + 5 t22 α2 - (-2 + 5 t22) α1 (1 + α2))))) +
   m2 (-p22^2 (1 + s2) (t12 - t22) (α1 + α2 + α1 α2) + (s2 - α1) (dd2 (-1 + t12 α2 -
            2 t22 α2 + s2 (t12 - 2 t22) (1 + α2)) + dd1 (1 + t22 α2 + s2 t22 (1 + α2))) +
      p22 (-2 - 2 α1 + dd1 α1 - 2 dd2 α1 - t12 α1 + 3 t22 α1 + dd1 α2 - 2 dd2 α2 +
         2 t12 α2 - 4 t22 α2 + dd1 α1 α2 - 2 dd2 α1 α2 + 2 t12 α1 α2 - 4 t22 α1 α2 -
         3 t12 t22 α1 α2 + 5 t22^2 α1 α2 + s2^2 (3 t12 - 5 t22) t22 (1 + α2) +
         s2 (5 t22^2 (α1 - α2 + α1 α2) + (dd1 - 2 dd2) (α1 + α2 + α1 α2) - t22 (7 + 4 α2 +
               4 α1 (1 + α2)) + t12 (3 + 2 α2 + 3 t22 α2 - (-2 + 3 t22) α1 (1 + α2)))) +
      p12 (2 + 2 α1 + dd2 α1 - t12 α1 + p22 t12 α1 - t22 α1 - p22 t22 α1 + dd2 α2 +
         p22 t12 α2 + 2 t22 α2 - p22 t22 α2 + dd2 α1 α2 + p22 t12 α1 α2 +
         2 t22 α1 α2 - p22 t22 α1 α2 - t12 t22 α1 α2 - t22^2 α1 α2 +
```

```
            s2^2 t22 (t12 + t22) (1 + α2) + s2 (-t22^2 (α1 - α2 + α1 α2) +
                dd2 (α1 + α2 + α1 α2) - t22 (-3 + (-2 + p22) α2 + (-2 + p22) α1 (1 + α2)) +
                t12 (1 - t22 (α1 - α2 + α1 α2) + p22 (α1 + α2 + α1 α2)))))) /
    (2 (1 + s2 t22 + m2 (t12 - t22) (s2 - α1) + α1 - t22 α1) (1 + t22 α2 +
        s2 t22 (1 + α2) + m2 (t12 - t22) (s2 + α2 + s2 α2))),
 -t22 + ((m2 (t12 - t22) + t22) (2 + m2 p12 α2 + p22 α2 - m2 p22 α2 + 2 m2 t12 α2 -
        m2^2 p12 t12 α2 - m2 p22 t12 α2 + m2^2 p22 t12 α2 +
        2 t22 α2 - 2 m2 t22 α2 - m2 p12 t22 α2 + m2^2 p12 t22 α2 -
        p22 t22 α2 + 2 m2 p22 t22 α2 - m2^2 p22 t22 α2 +
        s2^2 (m2^2 (t12 - t22)^2 + t22 (1 + t22) + m2 (t12 - t22) (1 + 2 t22)) (1 + α2) -
        (-1 + m2 (t12 - t22) + t22) α1
         (1 + p22 + p22 α2 + t22 α2 + m2 ((t12 - t22) α2 + p12 (1 + α2) - p22 (1 + α2))) -
        s2 (-1 - 3 t22 - p22 α1 - t22 α1 + p22 t22 α1 + t22^2 α1 - p22 α2 - 3 t22 α2 + p22 t22 α2 -
           t22^2 α2 - p22 α1 α2 - t22 α1 α2 + p22 t22 α1 α2 + t22^2 α1 α2 + m2^2 (t12 - t22)
            ((t12 - t22) (α1 - α2 + α1 α2) + p12 (α1 + α2 + α1 α2) - p22 (α1 + α2 + α1 α2)) +
           m2 (-2 t22^2 (α1 - α2 + α1 α2) - (p12 - p22) (α1 + α2 + α1 α2) + t22
                (3 + (3 + p12 - 2 p22) α2 + (1 + p12 - 2 p22) α1 (1 + α2)) + t12
                (-3 + (-3 + p22 - 2 t22) α2 + (-1 + p22 + 2 t22) α1 (1 + α2)))))) /
    (2 (1 + s2 t22 + m2 (t12 - t22) (s2 - α1) + α1 - t22 α1) (1 + t22 α2 +
        s2 t22 (1 + α2) + m2 (t12 - t22) (s2 + α2 + s2 α2))),
 1/4 (-4 dd2 - ((r (1 + s2) (dd2 + dd1 m2 - dd2 m2 + m2 p12 t12 + p22 t22 - m2 p22 t22)
           (1 + dd2 + dd1 m2 - dd2 m2 - m2 p12 - p22 + m2 p22 - m2 t12 +
             m2 p12 t12 - t22 + m2 t22 + p22 t22 - m2 p22 t22)) /
         (1 + s2 t22 + m2 (t12 - t22) (s2 - α1) + α1 - t22 α1) - ((-1 + r)
           (dd2 (-1 + m2) - dd1 m2 + m2 p12 + p22 - m2 p22 - m2 p12 t12 - p22 t22 + m2 p22 t22)
           (dd2 (-1 + m2) - dd1 m2 + m2 t12 - m2 p12 t12 + t22 - m2 t22 - p22 t22 + m2 p22 t22)
           (1 + α1)) / (1 + s2 t22 + m2 (t12 - t22) (s2 - α1) + α1 - t22 α1) -
        ((dd2 (-1 + m2) - dd1 m2 + m2 p12 + p22 - m2 p22 - m2 p12 t12 - p22 t22 + m2 p22 t22)
           (-1 + dd2 (-1 + m2) - dd1 m2 + m2 p12 + p22 - m2 p22 + m2 t12 -
             m2 p12 t12 + t22 - m2 t22 - p22 t22 + m2 p22 t22) (1 + α1)) /
         (1 + s2 t22 + m2 (t12 - t22) (s2 - α1) + α1 - t22 α1) + (2 (dd2 + dd1 m2 -
              dd2 m2 - m2 p12 - p22 + m2 p22 + m2 p12 t12 + p22 t22 - m2 p22 t22)^2) /
         (1 + t22 α2 + s2 t22 (1 + α2) + m2 (t12 - t22) (s2 + α2 + s2 α2)) -
        ((dd2 (-1 + m2) - dd1 m2 - m2 p12 t12 - p22 t22 + m2 p22 t22) (dd2 (-1 + m2) -
             dd1 m2 + m2 p12 + p22 - m2 p22 - m2 p12 t12 - p22 t22 + m2 p22 t22)) /
         (1 + t22 α2 + s2 t22 (1 + α2) + m2 (t12 - t22) (s2 + α2 + s2 α2)) +
        (r (dd2 (-1 + m2) - dd1 m2 - m2 p12 t12 - p22 t22 + m2 p22 t22)
           (-1 + dd2 (-1 + m2) - dd1 m2 + m2 p12 + p22 - m2 p22 + m2 t12 -
             m2 p12 t12 + t22 - m2 t22 - p22 t22 + m2 p22 t22)) /
         (1 + t22 α2 + s2 t22 (1 + α2) + m2 (t12 - t22) (s2 + α2 + s2 α2)) -
        ((dd2 (-1 + m2) - dd1 m2 + m2 p12 + p22 - m2 p22 - m2 p12 t12 - p22 t22 + m2 p22 t22)
           (-1 + dd2 (-1 + m2) - dd1 m2 + m2 p12 + p22 - m2 p22 + m2 t12 -
             m2 p12 t12 + t22 - m2 t22 - p22 t22 + m2 p22 t22)) /
         (1 + t22 α2 + s2 t22 (1 + α2) + m2 (t12 - t22) (s2 + α2 + s2 α2)) -
        ((1 + s2) (dd2 + dd1 m2 - dd2 m2 + m2 p12 t12 + p22 t22 - m2 p22 t22) (dd2 + dd1 m2 -
             dd2 m2 - m2 p12 - p22 + m2 p22 + m2 p12 t12 + p22 t22 - m2 p22 t22) (1 + α2)) /
         (1 + t22 α2 + s2 t22 (1 + α2) + m2 (t12 - t22) (s2 + α2 + s2 α2)) -
        ((-1 + r) (1 + s2) (dd2 + dd1 m2 - dd2 m2 - m2 p12 - p22 + m2 p22 +
```

```
            m2 p12 t12 + p22 t22 - m2 p22 t22) (dd2 + dd1 m2 - dd2 m2 - m2 t12 +
            m2 p12 t12 - t22 + m2 t22 + p22 t22 - m2 p22 t22) (1 + α2)) /
         (1 + t22 α2 + s2 t22 (1 + α2) + m2 (t12 - t22) (s2 + α2 + s2 α2)))
   (- (((1 + s2) (dd2 + dd1 m2 - dd2 m2 + m2 p12 t12 + p22 t22 - m2 p22 t22) (dd2 +
               dd1 m2 - dd2 m2 - m2 t12 + m2 p12 t12 - t22 + m2 t22 + p22 t22 - m2 p22 t22)) /
          (1 + s2 t22 + m2 (t12 - t22) (s2 - α1) + α1 - t22 α1)) +
       (2 (1 + s2) (dd2 + dd1 m2 - dd2 m2 - m2 t12 + m2 p12 t12 - t22 + m2 t22 + p22 t22 -
              m2 p22 t22)^2) / (1 + s2 t22 + m2 (t12 - t22) (s2 - α1) + α1 - t22 α1) +
       (r (1 + s2) (dd2 + dd1 m2 - dd2 m2 + m2 p12 t12 + p22 t22 - m2 p22 t22)
          (1 + dd2 + dd1 m2 - dd2 m2 - m2 p12 - p22 + m2 p22 - m2 t12 +
            m2 p12 t12 - t22 + m2 t22 + p22 t22 - m2 p22 t22)) /
         (1 + s2 t22 + m2 (t12 - t22) (s2 - α1) + α1 - t22 α1) -
       ((1 + s2) (dd2 (-1 + m2) - dd1 m2 + m2 t12 - m2 p12 t12 + t22 - m2 t22 -
            p22 t22 + m2 p22 t22) (-1 + dd2 (-1 + m2) - dd1 m2 + m2 p12 + p22 -
            m2 p22 + m2 t12 - m2 p12 t12 + t22 - m2 t22 - p22 t22 + m2 p22 t22)) /
         (1 + s2 t22 + m2 (t12 - t22) (s2 - α1) + α1 - t22 α1) - ((-1 + r)
          (dd2 (-1 + m2) - dd1 m2 + m2 p12 + p22 - m2 p22 - m2 p12 t12 - p22 t22 + m2 p22 t22)
          (dd2 (-1 + m2) - dd1 m2 + m2 t12 - m2 p12 t12 + t22 - m2 t22 - p22 t22 + m2 p22 t22)
          (1 + α1)) / (1 + s2 t22 + m2 (t12 - t22) (s2 - α1) + α1 - t22 α1) -
       ((dd2 (-1 + m2) - dd1 m2 + m2 t12 - m2 p12 t12 + t22 - m2 t22 - p22 t22 + m2 p22 t22)
          (-1 + dd2 (-1 + m2) - dd1 m2 + m2 p12 + p22 - m2 p22 + m2 t12 -
            m2 p12 t12 + t22 - m2 t22 - p22 t22 + m2 p22 t22) (1 + α1)) /
         (1 + s2 t22 + m2 (t12 - t22) (s2 - α1) + α1 - t22 α1) +
       (r (dd2 (-1 + m2) - dd1 m2 - m2 p12 t12 - p22 t22 + m2 p22 t22)
          (-1 + dd2 (-1 + m2) - dd1 m2 + m2 p12 + p22 - m2 p22 + m2 t12 -
            m2 p12 t12 + t22 - m2 t22 - p22 t22 + m2 p22 t22)) /
         (1 + t22 α2 + s2 t22 (1 + α2) + m2 (t12 - t22) (s2 + α2 + s2 α2)) -
       ((1 + s2) (dd2 + dd1 m2 - dd2 m2 + m2 p12 t12 + p22 t22 - m2 p22 t22) (dd2 + dd1 m2 -
            dd2 m2 - m2 t12 + m2 p12 t12 - t22 + m2 t22 + p22 t22 - m2 p22 t22) (1 + α2)) /
         (1 + t22 α2 + s2 t22 (1 + α2) + m2 (t12 - t22) (s2 + α2 + s2 α2)) -
       ((-1 + r) (1 + s2) (dd2 + dd1 m2 - dd2 m2 - m2 p12 - p22 + m2 p22 +
            m2 p12 t12 + p22 t22 - m2 p22 t22) (dd2 + dd1 m2 - dd2 m2 - m2 t12 +
            m2 p12 t12 - t22 + m2 t22 + p22 t22 - m2 p22 t22) (1 + α2)) /
         (1 + t22 α2 + s2 t22 (1 + α2) + m2 (t12 - t22) (s2 + α2 + s2 α2))) +
  (- (((-1 + r) (1 + s2) (dd2 + dd1 m2 - dd2 m2 + m2 p12 t12 + p22 t22 - m2 p22 t22)
            (1 + dd2 + dd1 m2 - dd2 m2 - m2 p12 - p22 + m2 p22 - m2 t12 +
              m2 p12 t12 - t22 + m2 t22 + p22 t22 - m2 p22 t22)) /
          (1 + s2 t22 + m2 (t12 - t22) (s2 - α1) + α1 - t22 α1)) -
       ((1 + s2) (dd2 (-1 + m2) - dd1 m2 + m2 t12 - m2 p12 t12 + t22 - m2 t22 -
            p22 t22 + m2 p22 t22) (-1 + dd2 (-1 + m2) - dd1 m2 + m2 p12 + p22 -
            m2 p22 + m2 t12 - m2 p12 t12 + t22 - m2 t22 - p22 t22 + m2 p22 t22)) /
         (1 + s2 t22 + m2 (t12 - t22) (s2 - α1) + α1 - t22 α1) +
       (r (dd2 (-1 + m2) - dd1 m2 + m2 p12 + p22 - m2 p22 - m2 p12 t12 - p22 t22 + m2 p22 t22)
          (dd2 (-1 + m2) - dd1 m2 + m2 t12 - m2 p12 t12 + t22 - m2 t22 - p22 t22 + m2 p22 t22)
          (1 + α1)) / (1 + s2 t22 + m2 (t12 - t22) (s2 - α1) + α1 - t22 α1) -
       ((dd2 (-1 + m2) - dd1 m2 + m2 p12 + p22 - m2 p22 - m2 p12 t12 - p22 t22 + m2 p22 t22)
          (-1 + dd2 (-1 + m2) - dd1 m2 + m2 p12 + p22 - m2 p22 + m2 t12 -
            m2 p12 t12 + t22 - m2 t22 - p22 t22 + m2 p22 t22) (1 + α1)) /
         (1 + s2 t22 + m2 (t12 - t22) (s2 - α1) + α1 - t22 α1) -
```

```
((dd2 (-1 + m2) - dd1 m2 + m2 t12 - m2 p12 t12 + t22 - m2 t22 - p22 t22 + m2 p22 t22)
    (-1 + dd2 (-1 + m2) - dd1 m2 + m2 p12 + p22 - m2 p22 + m2 t12 -
      m2 p12 t12 + t22 - m2 t22 - p22 t22 + m2 p22 t22) (1 + α1)) /
  (1 + s2 t22 + m2 (t12 - t22) (s2 - α1) + α1 - t22 α1) +
 (2 (-1 + dd2 (-1 + m2) - dd1 m2 + m2 p12 + p22 - m2 p22 + m2 t12 -
       m2 p12 t12 + t22 - m2 t22 - p22 t22 + m2 p22 t22)^2 (1 + α1)) /
  (1 + s2 t22 + m2 (t12 - t22) (s2 - α1) + α1 - t22 α1) -
 ((-1 + r) (dd2 (-1 + m2) - dd1 m2 - m2 p12 t12 - p22 t22 + m2 p22 t22)
    (-1 + dd2 (-1 + m2) - dd1 m2 + m2 p12 + p22 - m2 p22 + m2 t12 -
      m2 p12 t12 + t22 - m2 t22 - p22 t22 + m2 p22 t22)) /
  (1 + t22 α2 + s2 t22 (1 + α2) + m2 (t12 - t22) (s2 + α2 + s2 α2)) -
 ((dd2 (-1 + m2) - dd1 m2 + m2 p12 + p22 - m2 p22 - m2 p12 t12 - p22 t22 + m2 p22 t22)
    (-1 + dd2 (-1 + m2) - dd1 m2 + m2 p12 + p22 - m2 p22 + m2 t12 -
      m2 p12 t12 + t22 - m2 t22 - p22 t22 + m2 p22 t22)) /
  (1 + t22 α2 + s2 t22 (1 + α2) + m2 (t12 - t22) (s2 + α2 + s2 α2)) +
 (r (1 + s2) (dd2 + dd1 m2 - dd2 m2 - m2 p12 - p22 + m2 p22 + m2 p12 t12 +
      p22 t22 - m2 p22 t22) (dd2 + dd1 m2 - dd2 m2 - m2 t12 +
      m2 p12 t12 - t22 + m2 t22 + p22 t22 - m2 p22 t22) (1 + α2)) /
  (1 + t22 α2 + s2 t22 (1 + α2) + m2 (t12 - t22) (s2 + α2 + s2 α2)))
(-(((1 + s2) (dd2 + dd1 m2 - dd2 m2 + m2 p12 t12 + p22 t22 - m2 p22 t22) (dd2 +
        dd1 m2 - dd2 m2 - m2 t12 + m2 p12 t12 - t22 + m2 t22 + p22 t22 - m2 p22 t22)) /
    (1 + s2 t22 + m2 (t12 - t22) (s2 - α1) + α1 - t22 α1)) -
 ((-1 + r) (1 + s2) (dd2 + dd1 m2 - dd2 m2 + m2 p12 t12 + p22 t22 - m2 p22 t22)
    (1 + dd2 + dd1 m2 - dd2 m2 - m2 p12 - p22 + m2 p22 - m2 t12 +
      m2 p12 t12 - t22 + m2 t22 + p22 t22 - m2 p22 t22)) /
  (1 + s2 t22 + m2 (t12 - t22) (s2 - α1) + α1 - t22 α1) +
 (r (dd2 (-1 + m2) - dd1 m2 + m2 p12 + p22 - m2 p22 - m2 p12 t12 - p22 t22 + m2 p22 t22)
    (dd2 (-1 + m2) - dd1 m2 + m2 t12 - m2 p12 t12 + t22 - m2 t22 - p22 t22 + m2 p22 t22)
    (1 + α1)) / (1 + s2 t22 + m2 (t12 - t22) (s2 - α1) + α1 - t22 α1) -
 ((dd2 (-1 + m2) - dd1 m2 - m2 p12 t12 - p22 t22 + m2 p22 t22) (dd2 (-1 + m2) -
      dd1 m2 + m2 p12 + p22 - m2 p22 - m2 p12 t12 - p22 t22 + m2 p22 t22)) /
  (1 + t22 α2 + s2 t22 (1 + α2) + m2 (t12 - t22) (s2 + α2 + s2 α2)) -
 ((-1 + r) (dd2 (-1 + m2) - dd1 m2 - m2 p12 t12 - p22 t22 + m2 p22 t22)
    (-1 + dd2 (-1 + m2) - dd1 m2 + m2 p12 + p22 - m2 p22 + m2 t12 -
      m2 p12 t12 + t22 - m2 t22 - p22 t22 + m2 p22 t22)) /
  (1 + t22 α2 + s2 t22 (1 + α2) + m2 (t12 - t22) (s2 + α2 + s2 α2)) +
 (2 (1 + s2) (dd2 + dd1 m2 - dd2 m2 + m2 p12 t12 + p22 t22 - m2 p22 t22)^2 (1 + α2)) /
  (1 + t22 α2 + s2 t22 (1 + α2) + m2 (t12 - t22) (s2 + α2 + s2 α2)) -
 ((1 + s2) (dd2 + dd1 m2 - dd2 m2 + m2 p12 t12 + p22 t22 - m2 p22 t22) (dd2 + dd1 m2 -
      dd2 m2 - m2 p12 - p22 + m2 p22 + m2 p12 t12 + p22 t22 - m2 p22 t22) (1 + α2)) /
  (1 + t22 α2 + s2 t22 (1 + α2) + m2 (t12 - t22) (s2 + α2 + s2 α2)) -
 ((1 + s2) (dd2 + dd1 m2 - dd2 m2 + m2 p12 t12 + p22 t22 - m2 p22 t22) (dd2 + dd1 m2 -
      dd2 m2 - m2 t12 + m2 p12 t12 - t22 + m2 t22 + p22 t22 - m2 p22 t22) (1 + α2)) /
  (1 + t22 α2 + s2 t22 (1 + α2) + m2 (t12 - t22) (s2 + α2 + s2 α2)) +
 (r (1 + s2) (dd2 + dd1 m2 - dd2 m2 - m2 p12 - p22 + m2 p22 + m2 p12 t12 +
      p22 t22 - m2 p22 t22) (dd2 + dd1 m2 - dd2 m2 - m2 t12 +
      m2 p12 t12 - t22 + m2 t22 + p22 t22 - m2 p22 t22) (1 + α2)) /
  (1 + t22 α2 + s2 t22 (1 + α2) + m2 (t12 - t22) (s2 + α2 + s2 α2)))};
```

2.2.2 Structure of equilibrium conditions and reduction to two conditions

```
Coefficient[deltagenmale[[1]], r]
```

0

```
Coefficient[deltagenmale[[2]], r]
```

0

```
Exponent[deltagenmale[[3]], r]
```

2

This is not true because:

```
Factor[Coefficient[deltagenmale[[3]], r, 2]]
```

0

The recombination rate r occurs only linearly in deltagenmale[[3]] and deltagenmale[[6]].

The following gives the structure of the (numerator of the) components of deltagenmale

```
Exponent[Numerator[Factor[deltagenmale[[1]]]], {p12, t12, dd1, p22, t22, dd2}]
```

{2, 2, 1, 2, 2, 1}

```
Exponent[Numerator[Factor[deltagenmale[[2]]]], {p12, t12, dd1, p22, t22, dd2}]
```

{1, 3, 0, 1, 3, 0}

```
Exponent[Numerator[Factor[deltagenmale[[3]]]], {p12, t12, dd1, p22, t22, dd2}]
```

{3, 5, 2, 3, 5, 2}

```
Exponent[Numerator[Factor[deltagenmale[[4]]]], {p12, t12, dd1, p22, t22, dd2}]
```

{2, 2, 1, 2, 2, 1}

```
Exponent[Numerator[Factor[deltagenmale[[5]]]], {p12, t12, dd1, p22, t22, dd2}]
```

{1, 3, 0, 1, 3, 0}

```
Exponent[Numerator[Factor[deltagenmale[[6]]]], {p12, t12, dd1, p22, t22, dd2}]
```

{3, 5, 2, 3, 5, 2}

Therefore, deltagenmale[[2]] and deltagenmale[[5]] can be used to compute p12 and p22 (at equilibrium) as functions of (t12,t22):

```
Simplify[Solve[{deltagenmale[[2]], deltagenmale[[5]]} == 0, {p12, p22}]]
```

```
{{p12 → -((((-1 + m2) (1 + s2) (-1 + m2 (t12 - t22) + t22)
        (m2 (t12 - t22) + t22) (-(-1 + t12) t12 (s1 + α1 + s1 α1) +
          m1^2 (t12 - t22)^2 (s1 + α1 + s1 α1) + m1 (t12 - t22) (2 + s1 + α1 + s1 α1))
        (1 + s1 + (-1 + m1) s1 t12 - m1 s1 t22 + m1 t22 α2 + t12 (α2 - m1 α2)) -
      m1 (1 + s1) ((-1 + m1) t12 - m1 t22) (1 + (-1 + m1) t12 - m1 t22)
        (m2^2 (t12 - t22)^2 (s2 - α1) - (-1 + t22) t22 (s2 - α1) + m2 (t12 - t22) (2 + s2 + α1))
        (1 + t22 α2 + s2 t22 (1 + α2) + m2 (t12 - t22) (s2 + α2 + s2 α2))) / ((-1 + m1 + m2)
       (1 + s1) (1 + s2) (m2^2 (t12 - t22)^2 + (-1 + t22) t22 + m2 (t12 - t22) (-1 + 2 t22))
       ((-1 + m1)^2 t12^2 + m1 t22 (-1 + m1 t22) - (-1 + m1) t12 (-1 + 2 m1 t22))
       (α1 + α2 + α1 α2))),
  p22 → -((((-(-1 + t12) t12 (s1 + α1 + s1 α1) + m1^2 (t12 - t22)^2 (s1 + α1 + s1 α1) +
          m1 (t12 - t22) (2 + s1 + α1 + s1 α1))
        (1 + s1 + (-1 + m1) s1 t12 - m1 s1 t22 + m1 t22 α2 + t12 (α2 - m1 α2)) +
       ((-1 + m1) ((-1 + m2) (1 + s2) (-1 + m2 (t12 - t22) + t22)
            (m2 (t12 - t22) + t22) (-(-1 + t12) t12 (s1 + α1 + s1 α1) +
              m1^2 (t12 - t22)^2 (s1 + α1 + s1 α1) + m1 (t12 - t22) (2 + s1 + α1 + s1 α1))
            (1 + s1 + (-1 + m1) s1 t12 - m1 s1 t22 + m1 t22 α2 + t12 (α2 - m1 α2)) -
          m1 (1 + s1) ((-1 + m1) t12 - m1 t22) (1 + (-1 + m1) t12 - m1 t22) (m2^2 (t12 - t22)^2
              (s2 - α1) - (-1 + t22) t22 (s2 - α1) + m2 (t12 - t22) (2 + s2 + α1)) (1 +
              t22 α2 + s2 t22 (1 + α2) + m2 (t12 - t22) (s2 + α2 + s2 α2)))) / ((-1 + m1 + m2)
          (1 + s2) (m2^2 (t12 - t22)^2 + (-1 + t22) t22 + m2 (t12 - t22) (-1 + 2 t22)))) /
    (m1 (1 + s1) ((-1 + m1)^2 t12^2 + m1 t22 (-1 + m1 t22) - (-1 + m1) t12 (-1 + 2 m1 t22))
      (α1 + α2 + α1 α2)))}}
```

The following is (p12, p22) as a function of (t12, t22); it is independent of r and of (dd1, dd2):

```
p12p22genmt12 =
  {p12 → -(((-1 + m2) (1 + s2) (-1 + m2 (t12 - t22) + t22) (m2 (t12 - t22) + t22)
        (-(-1 + t12) t12 (s1 + α1 + s1 α1) + m1^2 (t12 - t22)^2 (s1 + α1 + s1 α1) +
          m1 (t12 - t22) (2 + s1 + α1 + s1 α1))
        (1 + s1 + (-1 + m1) s1 t12 - m1 s1 t22 + m1 t22 α2 + t12 (α2 - m1 α2)) -
       m1 (1 + s1) ((-1 + m1) t12 - m1 t22) (1 + (-1 + m1) t12 - m1 t22) (m2^2
           (t12 - t22)^2 (s2 - α1) - (-1 + t22) t22 (s2 - α1) + m2 (t12 - t22) (2 + s2 + α1))
        (1 + t22 α2 + s2 t22 (1 + α2) + m2 (t12 - t22) (s2 + α2 + s2 α2))) /
      ((-1 + m1 + m2) (1 + s1) (1 + s2) (m2^2 (t12 - t22)^2 + (-1 + t22) t22 +
          m2 (t12 - t22) (-1 + 2 t22)) ((-1 + m1)^2 t12^2 + m1 t22 (-1 + m1 t22) -
         (-1 + m1) t12 (-1 + 2 m1 t22)) (α1 + α2 + α1 α2))),
   p22 → -(((-(-1 + t12) t12 (s1 + α1 + s1 α1) + m1^2 (t12 - t22)^2 (s1 + α1 + s1 α1) +
          m1 (t12 - t22) (2 + s1 + α1 + s1 α1))
        (1 + s1 + (-1 + m1) s1 t12 - m1 s1 t22 + m1 t22 α2 + t12 (α2 - m1 α2)) +
       ((-1 + m1) ((-1 + m2) (1 + s2) (-1 + m2 (t12 - t22) + t22) (m2 (t12 - t22) + t22)
            (-(-1 + t12) t12 (s1 + α1 + s1 α1) + m1^2 (t12 - t22)^2 (s1 + α1 + s1 α1) +
              m1 (t12 - t22) (2 + s1 + α1 + s1 α1)) (1 + s1 + (-1 + m1) s1 t12 - m1 s1 t22 +
              m1 t22 α2 + t12 (α2 - m1 α2)) - m1 (1 + s1) ((-1 + m1) t12 - m1 t22)
            (1 + (-1 + m1) t12 - m1 t22) (m2^2 (t12 - t22)^2 (s2 - α1) - (-1 + t22) t22
               (s2 - α1) + m2 (t12 - t22) (2 + s2 + α1)) (1 + t22 α2 + s2 t22 (1 + α2) +
              m2 (t12 - t22) (s2 + α2 + s2 α2)))) / ((-1 + m1 + m2) (1 + s2)
          (m2^2 (t12 - t22)^2 + (-1 + t22) t22 + m2 (t12 - t22) (-1 + 2 t22)))) /
      (m1 (1 + s1) ((-1 + m1)^2 t12^2 + m1 t22 (-1 + m1 t22) - (-1 + m1) t12 (-1 + 2 m1 t22))
        (α1 + α2 + α1 α2))};
```

The structure of deltagenmale (see above) suggests that we can obtain (dd1, dd2) as functions of (t12, t22) by solving {deltagensym[[1]], deltagensym[[4]]} = 0:

```
delm1 = Factor[deltagenmale[[1]] /. p12p22genm12];
```

```
Exponent[Numerator[delm1], {t12, t22, dd1, dd2}]
```

```
{7, 7, 1, 1}
```

```
delm4 = Factor[deltagenmale[[4]] /. p12p22genmt12];
```

```
Exponent[Numerator[delm4], {t12, t22, dd1, dd2}]
```

```
{7, 7, 1, 1}
```

```
Simplify[Solve[{delm1, delm4} == 0, {dd1, dd2}]]
```

The following is (dd1, dd2) as a function of (t12, t22); it is independent of r:

```
dd12genmt12 =
  {dd1 → -(((-1 + t12) t12 (m2^3 (1 + s1) (t12 - t22)^3 ((-1 + m1)^2 t12^2 + m1 t22 (-1 + m1 t22) -
               (-1 + m1) t12 (-1 + 2 m1 t22)) (s2 - α1) (s2 + α2 + s2 α2) -
```

$$(-1+t22)\,t22\,(-m1^3\,(1+s2)\,(t12-t22)^3\,(s1+\alpha1+s1\,\alpha1)\,(s1-\alpha2)+$$
$$m1^2\,(t12-t22)^2\,(-2\,\alpha1+s1^2\,(1+s2)\,(-2+t12)\,(1+\alpha1)+2\,\alpha2+\alpha1\,\alpha2-$$
$$t12\,\alpha1\,\alpha2-t22\,\alpha1\,\alpha2+s2^2\,t22\,(1+\alpha2)+s2\,(1+(2+t22)\,\alpha2-$$
$$\alpha1\,(1+t22-\alpha2+t12\,\alpha2+t22\,\alpha2))+s1\,(-3+\alpha2-t12\,\alpha2+$$
$$s2^2\,t22\,(1+\alpha2)+\alpha1\,(-4+t12+\alpha2-t12\,\alpha2-t22\,\alpha2)+s2\,(-2+\alpha2-$$
$$t12\,\alpha2+t22\,\alpha2-\alpha1\,(3+t22+t12\,(-1+\alpha2)-\alpha2+t22\,\alpha2))))-$$
$$(-1+t12)\,t12\,(s1^2\,(1+s2)\,(-1+t12)\,(1+\alpha1)+(-t12+t22)\,\alpha1\,\alpha2-$$
$$s2^2\,t22\,(1+\alpha2)+s2\,(-1-t22\,\alpha2+\alpha1\,(-1+t22-t12\,\alpha2+t22\,\alpha2))-$$
$$s1\,(1+t12\,\alpha2+s2^2\,t22\,(1+\alpha2)+\alpha1\,(1+t12\,(-1+\alpha2)-t22\,\alpha2)+$$
$$s2\,(2+t12\,\alpha2+t22\,\alpha2-\alpha1\,(-2+t12+t22-t12\,\alpha2+t22\,\alpha2))))+$$
$$m1\,(t12-t22)\,(-2-2\,\alpha1+2\,t12\,\alpha1+s1^2\,(1+s2)\,(-1+t12^2)\,(1+\alpha1)-$$
$$2\,t12\,\alpha2-t12^2\,\alpha1\,\alpha2-t22\,\alpha1\,\alpha2+2\,t12\,t22\,\alpha1\,\alpha2-s2^2\,(-1+2\,t12)$$
$$t22\,(1+\alpha2)-s2\,(1-t22\,\alpha2+t12^2\,\alpha1\,\alpha2+\alpha1\,(1+t22+t22\,\alpha2)+$$
$$2\,t12\,(1+\alpha2-t22\,(\alpha1-\alpha2+\alpha1\,\alpha2)))-s1\,(3+3\,\alpha1+t22\,\alpha1\,\alpha2+$$
$$s2^2\,(-1+2\,t12)\,t22\,(1+\alpha2)+t12^2\,(\alpha1\,(-1+\alpha2)+\alpha2)-$$
$$2\,t12\,(1+\alpha1+t22\,\alpha1\,\alpha2)+s2\,(2+t12^2\,\alpha2-t22\,\alpha2+2\,t12\,t22\,\alpha2+$$
$$\alpha1\,(2+t22+t12^2\,(-1+\alpha2)+t22\,\alpha2-2\,t12\,t22\,(1+\alpha2))))))+$$
$$m2^2\,(t12-t22)^2\,(m1^3\,(1+s2)\,(t12-t22)^3\,(s1+\alpha1+s1\,\alpha1)\,(s1-\alpha2)+$$
$$(-1+t12)\,t12\,(-2\,\alpha1+s1^2\,(1+s2)\,(-1+t12)\,(1+\alpha1)+2\,\alpha2+\alpha1\,\alpha2-$$
$$t12\,\alpha1\,\alpha2-t22\,\alpha1\,\alpha2+s2^2\,(1+t22)\,(1+\alpha2)+s2\,(3+(3+\alpha1-t12\,\alpha1)\,\alpha2-$$
$$t22\,(\alpha1-\alpha2+\alpha1\,\alpha2))+s1\,(-1+2\,\alpha2-t12\,\alpha2+s2^2\,(1+t22)\,(1+\alpha2)+$$
$$\alpha1\,(-3+t12+\alpha2-t12\,\alpha2-t22\,\alpha2)-s2\,(-2+(-3+t12-t22)\,\alpha2+$$
$$\alpha1\,(1+t22+t12\,(-1+\alpha2)-\alpha2+t22\,\alpha2))))-m1^2\,(t12-t22)^2$$
$$(s1^2\,(1+s2)\,(-2+t12)\,(1+\alpha1)+(-t12+t22)\,\alpha1\,\alpha2-s2^2\,(1+t22)$$
$$(1+\alpha2)+s2\,(-3-(1+t22)\,\alpha2+\alpha1\,(-2+t22-t12\,\alpha2+t22\,\alpha2))-s1$$
$$(3+\alpha2+t12\,\alpha2+s2^2\,(1+t22)\,(1+\alpha2)+\alpha1\,(2+t12\,(-1+\alpha2)-t22\,\alpha2)+$$
$$s2\,(6+(2+t12+t22)\,\alpha2-\alpha1\,(-4+t12+t22-t12\,\alpha2+t22\,\alpha2))))-$$
$$m1\,(t12-t22)\,(-2-2\,t12\,\alpha1+s1^2\,(1+s2)\,(-1+t12^2)\,(1+\alpha1)-$$
$$2\,\alpha2+2\,t12\,\alpha2-\alpha1\,\alpha2+2\,t12\,\alpha1\,\alpha2-t12^2\,\alpha1\,\alpha2+t22\,\alpha1\,\alpha2-$$
$$2\,t12\,t22\,\alpha1\,\alpha2+s2^2\,(-1+2\,t12)\,(1+t22)\,(1+\alpha2)-$$
$$s2\,(5+3\,\alpha2+t22\,\alpha2+t12^2\,\alpha1\,\alpha2+\alpha1\,(2+\alpha2-t22\,(1+\alpha2))+$$
$$2\,t12\,(-3-(2+t22)\,\alpha2+(-1+t22)\,\alpha1\,(1+\alpha2)))+$$
$$s1\,(-3-\alpha1-2\,\alpha2-\alpha1\,\alpha2+t22\,\alpha1\,\alpha2+s2^2\,(-1+2\,t12)$$
$$(1+t22)\,(1+\alpha2)-t12^2\,(\alpha1\,(-1+\alpha2)+\alpha2)-$$
$$2\,t12\,(-1+\alpha1-2\,\alpha2+(-1+t22)\,\alpha1\,\alpha2)-s2\,(6+3\,\alpha2+t22\,\alpha2+$$
$$t12^2\,(\alpha1\,(-1+\alpha2)+\alpha2)+\alpha1\,(3+\alpha2-t22\,(1+\alpha2))+2\,t12$$
$$(-4-(3+t22)\,\alpha2+(-1+t22)\,\alpha1\,(1+\alpha2))))))+m2\,(t12-t22)$$
$$((-1+m1)^2\,(1+m1)\,(1+s2)\,t12^3\,(-1+2\,t22)\,(s1+\alpha1+s1\,\alpha1)\,(s1-\alpha2)+$$
$$m1\,t22\,(s2-4\,t22+2\,m1\,t22-6\,s2\,t22+m1\,s2\,t22-2\,s2^2\,t22+2\,m1\,s2\,t22^2+$$
$$s2^2\,t22^2+2\,m1\,s2^2\,t22^2-m1\,s2^2\,t22^3+s2\,\alpha1-2\,t22\,\alpha1-2\,s2\,t22\,\alpha1-$$
$$m1\,s2\,t22\,\alpha1+2\,m1\,t22^2\,\alpha1-s2\,t22^2\,\alpha1+2\,m1\,s2\,t22^2\,\alpha1+m1\,s2\,t22^3\,\alpha1-$$
$$s1^2\,(1+s2)\,(-1+2\,t22)\,(-1+m1\,t22)^2\,(1+\alpha1)-2\,t22\,\alpha2+2\,m1\,t22\,\alpha2-$$
$$4\,s2\,t22\,\alpha2+2\,m1\,s2\,t22\,\alpha2-2\,s2^2\,t22\,\alpha2-2\,m1\,t22^2\,\alpha2+s2\,t22^2\,\alpha2+$$
$$s2^2\,t22^2\,\alpha2+2\,m1\,s2^2\,t22^2\,\alpha2-m1\,s2\,t22^3\,\alpha2-m1\,s2^2\,t22^3\,\alpha2+$$
$$m1\,t22\,\alpha1\,\alpha2+m1\,s2\,t22\,\alpha1\,\alpha2-t22^2\,\alpha1\,\alpha2-2\,m1\,t22^2\,\alpha1\,\alpha2-$$
$$m1^2\,t22^2\,\alpha1\,\alpha2-s2\,t22^2\,\alpha1\,\alpha2-2\,m1\,s2\,t22^2\,\alpha1\,\alpha2-m1^2\,s2\,t22^2\,\alpha1\,\alpha2+$$
$$m1\,t22^3\,\alpha1\,\alpha2+2\,m1^2\,t22^3\,\alpha1\,\alpha2+m1\,s2\,t22^3\,\alpha1\,\alpha2+2\,m1^2\,s2\,t22^3\,\alpha1\,\alpha2+$$
$$s1\,(-1+m1\,t22)\,(-1-\alpha1-s2^2\,(-2+t22)\,t22\,(1+\alpha2)+t22^2\,(\alpha1\,\alpha2+2\,m1$$
$$(\alpha1\,(-1+\alpha2)+\alpha2))+t22\,(6+2\,\alpha2-m1\,\alpha2+\alpha1\,(4+m1-m1\,\alpha2))+$$

```
                    s2 (-2 (1 + α1) + t22^2 ((-1 + 2 m1) α2 + α1 (1 + 2 m1 (-1 + α2) + α2)) +
                        t22 (8 + 4 α2 - m1 α2 + α1 (4 + m1 - m1 α2))))) +
              t12 (-2 + 8 m1 t22 - 4 m1^2 t22 - 2 α1 + 2 t22 α1 + 4 m1 t22 α1 - 4 m1^2 t22^2 α1 +
                  s1^2 (1 + s2) (-1 + 2 t22) (1 + m1 + 3 m1^3 t22^2 - m1^2 t22 (4 + t22)) (1 + α1) -
                  2 t22 α2 + 4 m1 t22 α2 - 4 m1^2 t22 α2 + 4 m1^2 t22^2 α2 - 2 m1^2 t22 α1 α2 - t22^2
                   α1 α2 + m1 t22^2 α1 α2 + 3 m1^2 t22^2 α1 α2 + 3 m1^3 t22^2 α1 α2 + 2 m1 t22^3 α1 α2 -
                  6 m1^3 t22^3 α1 α2 + (-1 + m1) s2^2 (-2 + t22) t22 (-1 + 2 m1 t22) (1 + α2) -
                  s2 (1 + α1 + 3 m1^3 t22^2 (-1 + 2 t22) α1 α2 + t22 (2 - 2 α1 + 4 α2) +
                      m1^2 t22 (2 + t22 (4 + α1 (4 - 3 α2)) + 2 t22^2 (α1 - α2) + 2 α1 (-1 + α2) +
                          4 α2) + t22^2 (α1 - α2 + α1 α2) - m1 (-1 - α1 + 2 t22 (4 + α1 + 3 α2) +
                          2 t22^3 (α1 - α2 + α1 α2) + t22^2 (4 + α1 + 3 α2 + α1 α2))) +
                  s1 (-3 + 2 t22 - 3 α1 + 4 t22 α1 - 2 t22 α2 - t22^2 α1 α2 + s2^2 (-2 + t22)
                       t22 (1 + α2) - 3 m1^3 (1 + s2) t22^2 (-1 + 2 t22) (α1 (-1 + α2) + α2) -
                      s2 (2 + 4 t22 α2 - t22^2 α2 + α1 (2 - 4 t22 + t22^2 (1 + α2))) +
                      m1^2 t22 (-2 t22^2 (α1 - α2) + 2 s2^2 (-2 + t22) t22 (1 + α2) -
                          2 (-1 + α1 (-2 + α2) + α2) + t22 (-12 - α2 + α1 (-11 + 3 α2)) -
                          s2 (4 t22^2 (α1 - α2) + 2 (-2 + α1 (-3 + α2) + α2) +
                              t22 (16 + α1 (11 - 3 α2) + 5 α2))) + m1 (-1 - α1 + 2 t22^3 α1 α2 -
                          s2^2 t22 (-2 - 3 t22 + 2 t22^2) (1 + α2) + 2 t22 (4 + 3 α1 + α2) +
                          t22^2 (4 + (4 + α1) α2) + s2 (-2 (1 + α1) + 4 t22 (2 + α1 + α2) +
                              2 t22^3 (α1 - α2 + α1 α2) + t22^2 (8 + α1 + 7 α2 + α1 α2))))) -
              (-1 + m1) t12^2 (2 + s2 + 2 s2 t22 + 2 s2^2 t22 - s2^2 t22^2 + 2 α1 + s2 α1 -
                  2 t22 α1 - 2 s2 t22 α1 + s2 t22^2 α1 - 2 s1^2 (1 + s2) (-1 + 2 t22) (1 + α1) +
                  3 m1^2 (1 + s2) t22 (-1 + 2 t22) (s1 + α1 + s1 α1) (s1 - α2) + 2 t22 α2 +
                  4 s2 t22 α2 + 2 s2^2 t22 α2 - s2 t22^2 α2 - s2^2 t22^2 α2 - α1 α2 -
                  s2 α1 α2 + 2 t22 α1 α2 + 2 s2 t22 α1 α2 + t22^2 α1 α2 + s2 t22^2 α1 α2 -
                  s1 (-3 - 4 α1 + α2 + α1 α2 - t22^2 α1 α2 + s2^2 (-2 + t22) t22 (1 + α2) +
                      t22 (2 + 6 α1 - 4 α2 - 2 α1 α2) + s2 (-2 + α2 - 6 t22 α2 +
                          t22^2 α2 - α1 (3 + 2 t22 (-3 + α2) - α2 + t22^2 (1 + α2)))) +
                  m1 (-2 - 2 t22 α1 + s1^2 (1 + s2) (2 - 5 t22 + 2 t22^2) (1 + α1) -
                      2 α2 + 2 t22 α2 - α1 α2 + 3 t22 α1 α2 - 3 t22^2 α1 α2 +
                      s2^2 (-2 + t22) t22 (1 + α2) - s2 (1 + t22 (2 + α1 (2 - 3 α2)) +
                          α1 (-1 + α2) + 2 α2 + t22^2 (α1 - α2 + 3 α1 α2)) + s1 (1 + 2 α1 + t22^2
                           (α1 (2 - 3 α2) - 2 α2) - α2 - α1 α2 + s2^2 (-2 + t22) t22 (1 + α2) +
                          t22 (-6 + α2 + α1 (-7 + 3 α2)) - s2 (-2 + α1 (-3 + α2) + α2 + t22
                              (8 + α1 (7 - 3 α2) + α2) + t22^2 (α2 + α1 (-1 + 3 α2))))))))) /
          ((-1 + m1 + m2) (1 + s1) (1 + s2) (t12 - t22) (-1 + m2 (t12 - t22) + t22)
            (m2 (t12 - t22) + t22)
            ((-1 + m1) t12 - m1 t22)
            (1 + (-1 + m1) t12 - m1 t22)
            (α1 + α2 + α1 α2))),
    dd2 → -((((-1 + t22) t22 (m2^3 (1 + s1) (t12 - t22)^3 ((-1 + m1)^2 t12^2 + m1 t22 (-1 + m1 t22) -
                  (-1 + m1) t12 (-1 + 2 m1 t22)) (s2 - α1) (s2 + α2 + s2 α2) -
            (-1 + t22) t22 (-m1^3 (1 + s2) (t12 - t22)^3 (s1 + α1 + s1 α1) (s1 - α2) +
              m1^2 (t12 - t22)^2 (-2 α1 + s1^2 (1 + s2) (-2 + t12) (1 + α1) +
                  2 α2 + α1 α2 - t12 α1 α2 - t22 α1 α2 + s2^2 t22 (1 + α2) +
                  s2 (1 + (2 + t22) α2 - α1 (1 + t22 - α2 + t12 α2 + t22 α2)) +
                  s1 (-3 + α2 - t12 α2 + s2^2 t22 (1 + α2) +
                      α1 (-4 + t12 + α2 - t12 α2 - t22 α2) + s2 (-2 + α2 - t12 α2 +
```

$$
\begin{aligned}
& t22\,\alpha2 - \alpha1\,(3 + t22 + t12\,(-1 + \alpha2) - \alpha2 + t22\,\alpha2)))) - \\
& (-1 + t12)\,t12\,(s1^2\,(1 + s2)\,(-1 + t12)\,(1 + \alpha1) + (-t12 + t22)\,\alpha1\,\alpha2 - \\
& s2^2\,t22\,(1 + \alpha2) + s2\,(-1 - t22\,\alpha2 + \alpha1\,(-1 + t22 - t12\,\alpha2 + t22\,\alpha2)) - \\
& s1\,(1 + t12\,\alpha2 + s2^2\,t22\,(1 + \alpha2) + \alpha1\,(1 + t12\,(-1 + \alpha2) - t22\,\alpha2) + \\
& s2\,(2 + t12\,\alpha2 + t22\,\alpha2 - \alpha1\,(-2 + t12 + t22 - t12\,\alpha2 + t22\,\alpha2))))) + \\
& m1\,(t12 - t22)\,(-2 - 2\,\alpha1 + 2\,t12\,\alpha1 + s1^2\,(1 + s2)\,(-1 + t12^2)\,(1 + \alpha1) - \\
& 2\,t12\,\alpha2 - t12^2\,\alpha1\,\alpha2 - t22\,\alpha1\,\alpha2 + 2\,t12\,t22\,\alpha1\,\alpha2 - s2^2\,(-1 + 2\,t12) \\
& t22\,(1 + \alpha2) - s2\,(1 - t22\,\alpha2 + t12^2\,\alpha1\,\alpha2 + \alpha1\,(1 + t22 + t22\,\alpha2) + \\
& 2\,t12\,(1 + \alpha2 - t22\,(\alpha1 - \alpha2 + \alpha1\,\alpha2))) - s1\,(3 + 3\,\alpha1 + t22\,\alpha1\,\alpha2 + \\
& s2^2\,(-1 + 2\,t12)\,t22\,(1 + \alpha2) + t12^2\,(\alpha1\,(-1 + \alpha2) + \alpha2) - \\
& 2\,t12\,(1 + \alpha1 + t22\,\alpha1\,\alpha2) + s2\,(2 + t12^2\,\alpha2 - t22\,\alpha2 + 2\,t12\,t22\,\alpha2 + \\
& \alpha1\,(2 + t22 + t12^2\,(-1 + \alpha2) + t22\,\alpha2 - 2\,t12\,t22\,(1 + \alpha2)))))) + \\
& m2^2\,(t12 - t22)^2\,(m1^3\,(1 + s2)\,(t12 - t22)^3\,(s1 + \alpha1 + s1\,\alpha1)\,(s1 - \alpha2) + \\
& (-1 + t12)\,t12\,(-2\,\alpha1 + s1^2\,(1 + s2)\,(-1 + t12)\,(1 + \alpha1) + \\
& 2\,\alpha2 + \alpha1\,\alpha2 - t12\,\alpha1\,\alpha2 - t22\,\alpha1\,\alpha2 + s2^2\,(1 + t22)\,(1 + \alpha2) + \\
& s2\,(3 + (3 + \alpha1 - t12\,\alpha1)\,\alpha2 - t22\,(\alpha1 - \alpha2 + \alpha1\,\alpha2)) + \\
& s1\,(-1 + 2\,\alpha2 - t12\,\alpha2 + s2^2\,(1 + t22)\,(1 + \alpha2) + \\
& \alpha1\,(-3 + t12 + \alpha2 - t12\,\alpha2 - t22\,\alpha2) - s2\,(-2 + (-3 + t12 - t22)\,\alpha2 + \\
& \alpha1\,(1 + t22 + t12\,(-1 + \alpha2) - \alpha2 + t22\,\alpha2)))) - m1^2\,(t12 - t22)^2 \\
& (s1^2\,(1 + s2)\,(-2 + t12)\,(1 + \alpha1) + (-t12 + t22)\,\alpha1\,\alpha2 - s2^2\,(1 + t22) \\
& (1 + \alpha2) + s2\,(-3 - (1 + t22)\,\alpha2 + \alpha1\,(-2 + t22 - t12\,\alpha2 + t22\,\alpha2)) - s1 \\
& (3 + \alpha2 + t12\,\alpha2 + s2^2\,(1 + t22)\,(1 + \alpha2) + \alpha1\,(2 + t12\,(-1 + \alpha2) - t22\,\alpha2) + \\
& s2\,(6 + (2 + t12 + t22)\,\alpha2 - \alpha1\,(-4 + t12 + t22 - t12\,\alpha2 + t22\,\alpha2)))) - \\
& m1\,(t12 - t22)\,(-2 - 2\,t12\,\alpha1 + s1^2\,(1 + s2)\,(-1 + t12^2)\,(1 + \alpha1) - \\
& 2\,\alpha2 + 2\,t12\,\alpha2 - \alpha1\,\alpha2 + 2\,t12\,\alpha1\,\alpha2 - t12^2\,\alpha1\,\alpha2 + t22\,\alpha1\,\alpha2 - \\
& 2\,t12\,t22\,\alpha1\,\alpha2 + s2^2\,(-1 + 2\,t12)\,(1 + t22)\,(1 + \alpha2) - \\
& s2\,(5 + 3\,\alpha2 + t22\,\alpha2 + t12^2\,\alpha1\,\alpha2 + \alpha1\,(2 + \alpha2 - t22\,(1 + \alpha2)) + \\
& 2\,t12\,(-3 - (2 + t22)\,\alpha2 + (-1 + t22)\,\alpha1\,(1 + \alpha2))) + \\
& s1\,(-3 - \alpha1 - 2\,\alpha2 - \alpha1\,\alpha2 + t22\,\alpha1\,\alpha2 + s2^2\,(-1 + 2\,t12) \\
& (1 + t22)\,(1 + \alpha2) - t12^2\,(\alpha1\,(-1 + \alpha2) + \alpha2) - \\
& 2\,t12\,(-1 + \alpha1 - 2\,\alpha2 + (-1 + t22)\,\alpha1\,\alpha2) - s2\,(6 + 3\,\alpha2 + \\
& t22\,\alpha2 + t12^2\,(\alpha1\,(-1 + \alpha2) + \alpha2) + \alpha1\,(3 + \alpha2 - t22\,(1 + \alpha2)) + \\
& 2\,t12\,(-4 - (3 + t22)\,\alpha2 + (-1 + t22)\,\alpha1\,(1 + \alpha2)))))) + \\
& m2\,(t12 - t22)\,((-1 + m1)^2\,(1 + m1)\,(1 + s2)\,t12^3\,(-1 + 2\,t22)\,(s1 + \\
& \alpha1 + s1\,\alpha1)\,(s1 - \alpha2) + m1\,t22\,(s2 - 4\,t22 + 2\,m1\,t22 - 6\,s2\,t22 + \\
& m1\,s2\,t22 - 2\,s2^2\,t22 + 2\,m1\,s2\,t22^2 + s2^2\,t22^2 + 2\,m1\,s2^2\,t22^2 - \\
& m1\,s2^2\,t22^3 + s2\,\alpha1 - 2\,t22\,\alpha1 - 2\,s2\,t22\,\alpha1 - m1\,s2\,t22\,\alpha1 + \\
& 2\,m1\,t22^2\,\alpha1 - s2\,t22^2\,\alpha1 + 2\,m1\,s2\,t22^2\,\alpha1 + m1\,s2\,t22^3\,\alpha1 - \\
& s1^2\,(1 + s2)\,(-1 + 2\,t22)\,(-1 + m1\,t22)^2\,(1 + \alpha1) - 2\,t22\,\alpha2 + \\
& 2\,m1\,t22\,\alpha2 - 4\,s2\,t22\,\alpha2 + 2\,m1\,s2\,t22\,\alpha2 - 2\,s2^2\,t22\,\alpha2 - \\
& 2\,m1\,t22^2\,\alpha2 + s2\,t22^2\,\alpha2 + s2^2\,t22^2\,\alpha2 + 2\,m1\,s2^2\,t22^2\,\alpha2 - \\
& m1\,s2\,t22^3\,\alpha2 - m1\,s2^2\,t22^3\,\alpha2 + m1\,t22\,\alpha1\,\alpha2 + m1\,s2\,t22\,\alpha1\,\alpha2 - \\
& t22^2\,\alpha1\,\alpha2 - 2\,m1\,t22^2\,\alpha1\,\alpha2 - m1^2\,t22^2\,\alpha1\,\alpha2 - s2\,t22^2\,\alpha1\,\alpha2 - \\
& 2\,m1\,s2\,t22^2\,\alpha1\,\alpha2 - m1^2\,s2\,t22^2\,\alpha1\,\alpha2 + m1\,t22^3\,\alpha1\,\alpha2 + 2\,m1^2\,t22^3\,\alpha1\,\alpha2 + \\
& m1\,s2\,t22^3\,\alpha1\,\alpha2 + 2\,m1^2\,s2\,t22^3\,\alpha1\,\alpha2 + s1\,(-1 + m1\,t22)\,(-1 - \alpha1 - \\
& s2^2\,(-2 + t22)\,t22\,(1 + \alpha2) + t22^2\,(\alpha1\,\alpha2 + 2\,m1\,(\alpha1\,(-1 + \alpha2) + \alpha2)) + \\
& t22\,(6 + 2\,\alpha2 - m1\,\alpha2 + \alpha1\,(4 + m1 - m1\,\alpha2)) + s2 \\
& (-2\,(1 + \alpha1) + t22^2\,((-1 + 2\,m1)\,\alpha2 + \alpha1\,(1 + 2\,m1\,(-1 + \alpha2) + \alpha2)) + \\
& t22\,(8 + 4\,\alpha2 - m1\,\alpha2 + \alpha1\,(4 + m1 - m1\,\alpha2))))) +
\end{aligned}
$$

```
          t12 (-2 + 8 m1 t22 - 4 m1^2 t22 - 2 α1 + 2 t22 α1 + 4 m1 t22 α1 - 4 m1^2
              t22^2 α1 + s1^2 (1 + s2) (-1 + 2 t22) (1 + m1 + 3 m1^3 t22^2 - m1^2 t22 (4 + t22))
              (1 + α1) - 2 t22 α2 + 4 m1 t22 α2 - 4 m1^2 t22 α2 + 4 m1^2 t22^2 α2 -
             2 m1^2 t22 α1 α2 - t22^2 α1 α2 + m1 t22^2 α1 α2 + 3 m1^2 t22^2 α1 α2 +
             3 m1^3 t22^2 α1 α2 + 2 m1 t22^3 α1 α2 - 6 m1^3 t22^3 α1 α2 +
             (-1 + m1) s2^2 (-2 + t22) t22 (-1 + 2 m1 t22) (1 + α2) -
             s2 (1 + α1 + 3 m1^3 t22^2 (-1 + 2 t22) α1 α2 + t22 (2 - 2 α1 + 4 α2) +
                m1^2 t22 (2 + t22 (4 + α1 (4 - 3 α2)) + 2 t22^2 (α1 - α2) + 2 α1 (-1 + α2) +
                   4 α2) + t22^2 (α1 - α2 + α1 α2) - m1 (-1 - α1 + 2 t22 (4 + α1 + 3 α2) +
                   2 t22^3 (α1 - α2 + α1 α2) + t22^2 (4 + α1 + 3 α2 + α1 α2))) +
             s1 (-3 + 2 t22 - 3 α1 + 4 t22 α1 - 2 t22 α2 - t22^2 α1 α2 + s2^2 (-2 + t22)
                 t22 (1 + α2) - 3 m1^3 (1 + s2) t22^2 (-1 + 2 t22) (α1 (-1 + α2) + α2) -
                s2 (2 + 4 t22 α2 - t22^2 α2 + α1 (2 - 4 t22 + t22^2 (1 + α2))) +
                m1^2 t22 (-2 t22^2 (α1 - α2) + 2 s2^2 (-2 + t22) t22 (1 + α2) -
                   2 (-1 + α1 (-2 + α2) + α2) + t22 (-12 - α2 + α1 (-11 + 3 α2)) -
                   s2 (4 t22^2 (α1 - α2) + 2 (-2 + α1 (-3 + α2) + α2) +
                      t22 (16 + α1 (11 - 3 α2) + 5 α2))) + m1 (-1 - α1 + 2 t22^3 α1 α2 -
                   s2^2 t22 (-2 - 3 t22 + 2 t22^2) (1 + α2) + 2 t22 (4 + 3 α1 + α2) +
                   t22^2 (4 + (4 + α1) α2) + s2 (-2 (1 + α1) + 4 t22 (2 + α1 + α2) +
                      2 t22^3 (α1 - α2 + α1 α2) + t22^2 (8 + α1 + 7 α2 + α1 α2))))) -
          (-1 + m1) t12^2 (2 + s2 + 2 s2 t22 + 2 s2^2 t22 - s2^2 t22^2 + 2 α1 + s2 α1 -
             2 t22 α1 - 2 s2 t22 α1 + s2 t22^2 α1 - 2 s1^2 (1 + s2) (-1 + 2 t22) (1 + α1) +
             3 m1^2 (1 + s2) t22 (-1 + 2 t22) (s1 + α1 + s1 α1) (s1 - α2) + 2 t22 α2 +
             4 s2 t22 α2 + 2 s2^2 t22 α2 - s2 t22^2 α2 - s2^2 t22^2 α2 - α1 α2 -
             s2 α1 α2 + 2 t22 α1 α2 + 2 s2 t22 α1 α2 + t22^2 α1 α2 + s2 t22^2 α1 α2 -
             s1 (-3 - 4 α1 + α2 + α1 α2 - t22^2 α1 α2 + s2^2 (-2 + t22) t22 (1 + α2) +
                t22 (2 + 6 α1 - 4 α2 - 2 α1 α2) + s2 (-2 + α2 - 6 t22 α2 +
                   t22^2 α2 - α1 (3 + 2 t22 (-3 + α2) - α2 + t22^2 (1 + α2)))) +
             m1 (-2 - 2 t22 α1 + s1^2 (1 + s2) (2 - 5 t22 + 2 t22^2) (1 + α1) -
                2 α2 + 2 t22 α2 - α1 α2 + 3 t22 α1 α2 - 3 t22^2 α1 α2 +
                s2^2 (-2 + t22) t22 (1 + α2) - s2 (1 + t22 (2 + α1 (2 - 3 α2)) +
                   α1 (-1 + α2) + 2 α2 + t22^2 (α1 - α2 + 3 α1 α2)) + s1 (1 + 2 α1 + t22^2
                    (α1 (2 - 3 α2) - 2 α2) - α2 - α1 α2 + s2^2 (-2 + t22) t22 (1 + α2) +
                   t22 (-6 + α2 + α1 (-7 + 3 α2)) - s2 (-2 + α1 (-3 + α2) + α2 + t22
                       (8 + α1 (7 - 3 α2) + α2) + t22^2 (α2 + α1 (-1 + 3 α2)))))))) /
    ((-1 + m1 + m2) (1 + s1) (1 + s2) (t12 - t22) (-1 + m2 (t12 - t22) + t22)
      (m2 (t12 - t22) + t22)
      ((-1 + m1) t12 - m1 t22)
      (1 + (-1 + m1) t12 - m1 t22)
      (α1 + α2 + α1 α2))};
```

Therefore, p12p22genmt12 and dd12genmt12 give the potential equilibria as functions of (t12, t22).
We still need to invoke deltagenmale[[3]] and deltagenmale[[6]] to get the equilibria.

We know from above that deltagenmale[[6]] is linear in r and deltagenmale[[3]] is quadratic in r

```
factdelta3maler0 = Factor[Coefficient[deltagenmale[[3]], r, 0]]
```

```
Factor[factdelta3maler0 /. p12p22genmt12 /. dd12genmt12]
```

```
0
```

This took on the order of 60 CPU hours!

Therefore, the constant terms are zero!!! Hence there is a manifold, and the set of equilibria existing for r>0 is independent of r.

```
factdelta6maler0 = Factor[Coefficient[deltagenmale[[6]], r, 0]]
```

$$-\left(\left(-4\ dd1\ m2 + \cdots 21585 \cdots + 10\ m2^4\ p22\ s2^2\ t22^5\ \alpha1^2\ \alpha2^2 - 5\ m2^5\ p22\ s2^2\ t22^5\ \alpha1^2\ \alpha2^2 + m2^6\ p22\ s2^2\ t22^5\ \alpha1^2\ \alpha2^2\right) \Big/ \left(4\ (1 + \cdots 9 \cdots + m2\ t22\ \alpha1)^2\ (1 + m2\ s2\ t12 + \cdots 8 \cdots + s2\ t22\ \alpha2 - m2\ s2\ t22\ \alpha2)^2\right)\right)$$

large output | show less | show more | show all | set size limit...

Did not factor this one (too time consuming, and should also be 0 by symmetry)

```
factdelta3maler1 = Simplify[Numerator[Factor[Coefficient[deltagenmale[[3]], r, 1]]]]
```

Therefore, equilibria have to satisfy Coefficient[deltagenmale[[3]], r, 1] = 0 and Coefficient[delta-genmale[[6]], r, 1] = 0.
This shows that if r>0, then the equilibria are independent of r.
If r=0, there is a manifold of equilibria which can be parameterized by (t12,t22).

```
factdelta6maler1 = Factor[Coefficient[deltagenmale[[6]], r, 1]]
```

```
Factor[factdelta6maler1 /. p12p22genmt12 /. dd12genmt12]
```

```
factdelta3maler1 = Factor[Coefficient[deltagenmale[[3]], r, 1]]
```

```
Factor[factdelta3maler1 /. p12p22genmt12 /. dd12genmt12]
```

$$\left(-4\ m^3\ t12^5 + 4\ m^2\ s\ t12^5 - 20\ m^3\ s\ t12^5 + \cdots 14582 \cdots + 24\ m^8\ s^2\ t22^{12}\ \alpha^4 - 16\ m^9\ s^2\ t22^{12}\ \alpha^4 + 4\ m^{10}\ s^2\ t22^{12}\ \alpha^4\right) \Big/ \left(2 \cdots 10 \cdots (1 + s - s\ t12 + m\ s\ t12 - m\ s\ t22 + \alpha + s\ \alpha - t12\ \alpha + m\ t12\ \alpha - s\ t12\ \alpha + m\ s\ t12\ \alpha - m\ t22\ \alpha - m\ s\ t22\ \alpha)\right)$$

large output | show less | show more | show all | set size limit...

Next we show generic independence of the two remaining equilibrium conditions:

```
Factor[Numerator[Factor[factdelta3maler1 /. p12p22genmt12 /. dd12genmt12]] -
  Numerator[Factor[factdelta6maler1 /. p12p22genmt12 /. dd12genmt12]]]
```

Generically, this is not zero. Therefore, for t12 there will be finitely many solutions t22. Because this becomes too complicated, we assume symmetric parameters below.

2.2.3 Symmetric parameters

```
sympar = {s1 → s, s2 → s, m1 → m, m2 → m, α1 → α, α2 → α};
```

```
factdelta6msymr1 = Factor[Coefficient[deltagenmale[[6]], r, 1] /. sympar];
```

```
Factor[factdelta6msymr1 /. p12p22genmt12 /. dd12genmt12 /. sympar]
```

```
(≪14434≫ + 24 m^8 s^2 t22^12 α^4 - 16 m^9 s^2 t22^12 α^4 + 4 m^10 s^2 t22^12 α^4) / (2 ≪10≫
   (1 + m s t12 + s t22 - m s t22 + m t12 α + m s t12 α + t22 α - m t22 α + s t22 α - m s t22 α))

large output | show less | show more | show all | set size limit...
```

```
factdelta3msymr1 = Factor[Coefficient[deltagenmale[[3]], r, 1] /. sympar];
```

```
Factor[factdelta3msymr1 /. p12p22genmt12 /. dd12genmt12 /. sympar]
```

```
(-4 m^3 t12^5 + 4 m^2 s t12^5 - 20 m^3 s t12^5 +
   ≪14582≫ + 24 m^8 s^2 t22^12 α^4 - 16 m^9 s^2 t22^12 α^4 + 4 m^10 s^2 t22^12 α^4) /
 (2 ≪10≫ (1 + s - s t12 + m s t12 - m s t22 + α + s α - t12 α +
   m t12 α - s t12 α + m s t12 α - m t22 α - m s t22 α))

large output | show less | show more | show all | set size limit...
```

Next we show generic independence of the two remaining equilibrium conditions:

```
Factor[Numerator[Factor[factdelta3msymr1 /. p12p22genmt12 /. dd12genmt12 /. sympar]] -
  Numerator[Factor[factdelta6msymr1 /. p12p22genmt12 /. dd12genmt12 /. sympar]]]
```

```
- (-1 + 2 m) s (2 + s) (t12 - t22) (-1 + t12 + t22) (-t12 + m t12 - m t22)
 (1 - t12 + m t12 - m t22) (-1 + m t12 + t22 - m t22) (m t12 + t22 - m t22) (1 + α)
 (-2 m t12^2 - 2 m s t12^2 - 2 m^2 s t12^2 - m s^2 t12^2 - m^2 s^2 t12^2 + 2 m t12^3 - 4 m^2 t12^3 + 4 m^3 t12^3 +
   2 m s t12^3 - 2 m^2 s t12^3 + 4 m^3 s t12^3 + 2 m s^2 t12^3 + 2 m^2 s t12^4 - 8 m^3 s t12^4 + 6 m^4 s t12^4 -
   m s^2 t12^4 - 2 m^3 s^2 t12^4 + 3 m^4 s^2 t12^4 + m^2 s^2 t12^5 - 3 m^4 s^2 t12^5 + 2 m^5 s^2 t12^5 - 2 s t12 t22 +
   4 m^2 s t12 t22 - s^2 t12 t22 + 2 m^2 s^2 t12 t22 - 2 m t12^2 t22 + 12 m^2 t12^2 t22 - 12 m^3 t12^2 t22 +
   2 s t12^2 t22 - 2 m s t12^2 t22 + 10 m^2 s t12^2 t22 - 12 m^3 s t12^2 t22 + 2 s^2 t12^2 t22 - 2 m s^2 t12^2 t22 +
   2 m^2 s^2 t12^2 t22 - 12 m^2 s t12^3 t22 + 32 m^3 s t12^3 t22 - 24 m^4 s t12^3 t22 - s^2 t12^3 t22 -
   4 m^2 s^2 t12^3 t22 + 12 m^3 s^2 t12^3 t22 - 12 m^4 s^2 t12^3 t22 + 2 m s^2 t12^4 t22 - 3 m^2 s^2 t12^4 t22 -
   4 m^3 s^2 t12^4 t22 + 15 m^4 s^2 t12^4 t22 - 10 m^5 s^2 t12^4 t22 + 2 m t22^2 + 2 m s t22^2 - 2 m^2 s t22^2 +
   m s^2 t22^2 - m^2 s^2 t22^2 + 2 m t12 t22^2 - 12 m^2 t12 t22^2 + 12 m^3 t12 t22^2 + 2 s t12 t22^2 +
   2 m s t12 t22^2 - 14 m^2 s t12 t22^2 + 12 m^3 s t12 t22^2 + 2 m s^2 t12 t22^2 - 4 m^2 s^2 t12 t22^2 -
   2 s t12^2 t22^2 + 20 m^2 s t12^2 t22^2 - 48 m^3 s t12^2 t22^2 + 36 m^4 s t12^2 t22^2 - s^2 t12^2 t22^2 +
   10 m^2 s^2 t12^2 t22^2 - 24 m^3 s^2 t12^2 t22^2 + 18 m^4 s^2 t12^2 t22^2 + s^2 t12^3 t22^2 - 4 m s^2 t12^3 t22^2 +
   4 m^2 s^2 t12^3 t22^2 + 12 m^3 s^2 t12^3 t22^2 - 30 m^4 s^2 t12^3 t22^2 + 20 m^5 s^2 t12^3 t22^2 - 2 m t22^3 +
   4 m^2 t22^3 - 4 m^3 t22^3 - 2 m s t22^3 + 6 m^2 s t22^3 - 4 m^3 s t22^3 - 2 m s^2 t22^3 + 2 m^2 s^2 t22^3 -
   12 m^2 s t12 t22^3 + 32 m^3 s t12 t22^3 - 24 m^4 s t12 t22^3 + s^2 t12 t22^3 - 8 m^2 s^2 t12 t22^3 +
   20 m^3 s^2 t12 t22^3 - 12 m^4 s^2 t12 t22^3 - s^2 t12^2 t22^3 + 4 m s^2 t12^2 t22^3 - 4 m^2 s^2 t12^2 t22^3 -
   12 m^3 s^2 t12^2 t22^3 + 30 m^4 s^2 t12^2 t22^3 - 20 m^5 s^2 t12^2 t22^3 + 2 m^2 s t22^4 - 8 m^3 s t22^4 +
   6 m^4 s t22^4 + m s^2 t22^4 + 2 m^2 s^2 t22^4 - 6 m^3 s^2 t22^4 + 3 m^4 s^2 t22^4 - 2 m s^2 t12 t22^4 +
   3 m^2 s^2 t12 t22^4 + 4 m^3 s^2 t12 t22^4 - 15 m^4 s^2 t12 t22^4 + 10 m^5 s^2 t12 t22^4 - m^2 s^2 t22^5 +
   3 m^4 s^2 t22^5 - 2 m^5 s^2 t22^5 - 2 m t12^2 α - 2 m s t12^2 α - 2 m^2 s t12^2 α - m s^2 t12^2 α - m^2 s^2 t12^2 α +
```

$2 m\, t12^{3} \alpha - 4 m^{2} t12^{3} \alpha + 4 m^{3} t12^{3} \alpha + 2 m\, s\, t12^{3} \alpha - 2 m^{2} s\, t12^{3} \alpha + 4 m^{3} s\, t12^{3} \alpha + 2 m\, s^{2} t12^{3} \alpha +$
$2 m^{2} s\, t12^{4} \alpha - 8 m^{3} s\, t12^{4} \alpha + 6 m^{4} s\, t12^{4} \alpha - m\, s^{2} t12^{4} \alpha - 2 m^{3} s^{2} t12^{4} \alpha + 3 m^{4} s^{2} t12^{4} \alpha +$
$m^{2} s^{2} t12^{5} \alpha - 3 m^{4} s^{2} t12^{5} \alpha + 2 m^{5} s^{2} t12^{5} \alpha - 2 s\, t12\, t22 \alpha + 4 m^{2} s\, t12\, t22 \alpha - s^{2} t12\, t22 \alpha +$
$2 m^{2} s^{2} t12\, t22 \alpha - 2 m\, t12^{2} t22 \alpha + 12 m^{2} t12^{2} t22 \alpha - 12 m^{3} t12^{2} t22 \alpha + 2 s\, t12^{2} t22 \alpha -$
$2 m\, s\, t12^{2} t22 \alpha + 10 m^{2} s\, t12^{2} t22 \alpha - 12 m^{3} s\, t12^{2} t22 \alpha + 2 s^{2} t12^{2} t22 \alpha - 2 m\, s^{2} t12^{2} t22 \alpha +$
$2 m^{2} s^{2} t12^{2} t22 \alpha - 12 m^{2} s\, t12^{3} t22 \alpha + 32 m^{3} s\, t12^{3} t22 \alpha - 24 m^{4} s\, t12^{3} t22 \alpha - s^{2} t12^{3} t22 \alpha -$
$4 m^{2} s^{2} t12^{3} t22 \alpha + 12 m^{3} s^{2} t12^{3} t22 \alpha - 12 m^{4} s^{2} t12^{3} t22 \alpha + 2 m\, s^{2} t12^{4} t22 \alpha -$
$3 m^{2} s^{2} t12^{4} t22 \alpha - 4 m^{3} s^{2} t12^{4} t22 \alpha + 15 m^{4} s^{2} t12^{4} t22 \alpha - 10 m^{5} s^{2} t12^{4} t22 \alpha +$
$2 m\, t22^{2} \alpha + 2 m\, s\, t22^{2} \alpha - 2 m^{2} s\, t22^{2} \alpha + m\, s^{2} t22^{2} \alpha - m^{2} s^{2} t22^{2} \alpha + 2 m\, t12\, t22^{2} \alpha -$
$12 m^{2} t12\, t22^{2} \alpha + 12 m^{3} t12\, t22^{2} \alpha + 2 s\, t12\, t22^{2} \alpha + 2 m\, s\, t12\, t22^{2} \alpha - 14 m^{2} s\, t12\, t22^{2} \alpha +$
$12 m^{3} s\, t12\, t22^{2} \alpha + 2 m\, s^{2} t12\, t22^{2} \alpha - 4 m^{2} s^{2} t12\, t22^{2} \alpha - 2 s\, t12^{2} t22^{2} \alpha + 20 m^{2} s\, t12^{2} t22^{2} \alpha -$
$48 m^{3} s\, t12^{2} t22^{2} \alpha + 36 m^{4} s\, t12^{2} t22^{2} \alpha - s^{2} t12^{2} t22^{2} \alpha + 10 m^{2} s^{2} t12^{2} t22^{2} \alpha -$
$24 m^{3} s^{2} t12^{2} t22^{2} \alpha + 18 m^{4} s^{2} t12^{2} t22^{2} \alpha + s^{2} t12^{3} t22^{2} \alpha - 4 m\, s^{2} t12^{3} t22^{2} \alpha +$
$4 m^{2} s^{2} t12^{3} t22^{2} \alpha + 12 m^{3} s^{2} t12^{3} t22^{2} \alpha - 30 m^{4} s^{2} t12^{3} t22^{2} \alpha + 20 m^{5} s^{2} t12^{3} t22^{2} \alpha -$
$2 m\, t22^{3} \alpha + 4 m^{2} t22^{3} \alpha - 4 m^{3} t22^{3} \alpha - 2 m\, s\, t22^{3} \alpha + 6 m^{2} s\, t22^{3} \alpha - 4 m^{3} s\, t22^{3} \alpha -$
$2 m\, s^{2} t22^{3} \alpha + 2 m^{2} s^{2} t22^{3} \alpha - 12 m^{2} s\, t12\, t22^{3} \alpha + 32 m^{3} s\, t12\, t22^{3} \alpha - 24 m^{4} s\, t12\, t22^{3} \alpha +$
$s^{2} t12\, t22^{3} \alpha - 8 m^{2} s^{2} t12\, t22^{3} \alpha + 20 m^{3} s^{2} t12\, t22^{3} \alpha - 12 m^{4} s^{2} t12\, t22^{3} \alpha - s^{2} t12^{2} t22^{3} \alpha +$
$4 m\, s^{2} t12^{2} t22^{3} \alpha - 4 m^{2} s^{2} t12^{2} t22^{3} \alpha - 12 m^{3} s^{2} t12^{2} t22^{3} \alpha + 30 m^{4} s^{2} t12^{2} t22^{3} \alpha -$
$20 m^{5} s^{2} t12^{2} t22^{3} \alpha + 2 m^{2} s\, t22^{4} \alpha - 8 m^{3} s\, t22^{4} \alpha + 6 m^{4} s\, t22^{4} \alpha + m\, s^{2} t22^{4} \alpha + 2 m^{2} s^{2} t22^{4} \alpha -$
$6 m^{3} s^{2} t22^{4} \alpha + 3 m^{4} s^{2} t22^{4} \alpha - 2 m\, s^{2} t12\, t22^{4} \alpha + 3 m^{2} s^{2} t12\, t22^{4} \alpha + 4 m^{3} s^{2} t12\, t22^{4} \alpha -$
$15 m^{4} s^{2} t12\, t22^{4} \alpha + 10 m^{5} s^{2} t12\, t22^{4} \alpha - m^{2} s^{2} t22^{5} \alpha + 3 m^{4} s^{2} t22^{5} \alpha - 2 m^{5} s^{2} t22^{5} \alpha -$
$m\, t12^{3} \alpha^{2} - m^{2} t12^{3} \alpha^{2} + 2 m^{3} t12^{3} \alpha^{2} - m\, s\, t12^{3} \alpha^{2} - m^{2} s\, t12^{3} \alpha^{2} + 2 m^{3} s\, t12^{3} \alpha^{2} + m\, t12^{4} \alpha^{2} +$
$m^{2} t12^{4} \alpha^{2} - 2 m^{3} t12^{4} \alpha^{2} + m\, s\, t12^{4} \alpha^{2} + m^{2} s\, t12^{4} \alpha^{2} - 2 m^{3} s\, t12^{4} \alpha^{2} - m^{2} t12^{5} \alpha^{2} + 3 m^{4} t12^{5} \alpha^{2} -$
$2 m^{5} t12^{5} \alpha^{2} - m^{2} s\, t12^{5} \alpha^{2} + 3 m^{4} s\, t12^{5} \alpha^{2} - 2 m^{5} s\, t12^{5} \alpha^{2} - t12^{2} t22 \alpha^{2} + m\, t12^{2} t22 \alpha^{2} +$
$3 m^{2} t12^{2} t22 \alpha^{2} - 6 m^{3} t12^{2} t22 \alpha^{2} - s\, t12^{2} t22 \alpha^{2} + m\, s\, t12^{2} t22 \alpha^{2} + 3 m^{2} s\, t12^{2} t22 \alpha^{2} -$
$6 m^{3} s\, t12^{2} t22 \alpha^{2} + t12^{3} t22 \alpha^{2} - 2 m^{2} t12^{3} t22 \alpha^{2} + 4 m^{3} t12^{3} t22 \alpha^{2} + s\, t12^{3} t22 \alpha^{2} -$
$2 m^{2} s\, t12^{3} t22 \alpha^{2} + 4 m^{3} s\, t12^{3} t22 \alpha^{2} - 2 m\, t12^{4} t22 \alpha^{2} + 3 m^{2} t12^{4} t22 \alpha^{2} + 4 m^{3} t12^{4} t22 \alpha^{2} -$
$15 m^{4} t12^{4} t22 \alpha^{2} + 10 m^{5} t12^{4} t22 \alpha^{2} - 2 m\, s\, t12^{4} t22 \alpha^{2} + 3 m^{2} s\, t12^{4} t22 \alpha^{2} + 4 m^{3} s\, t12^{4} t22 \alpha^{2} -$
$15 m^{4} s\, t12^{4} t22 \alpha^{2} + 10 m^{5} s\, t12^{4} t22 \alpha^{2} + t12\, t22^{2} \alpha^{2} - m\, t12\, t22^{2} \alpha^{2} - 3 m^{2} t12\, t22^{2} \alpha^{2} +$
$6 m^{3} t12\, t22^{2} \alpha^{2} + s\, t12\, t22^{2} \alpha^{2} - m\, s\, t12\, t22^{2} \alpha^{2} - 3 m^{2} s\, t12\, t22^{2} \alpha^{2} + 6 m^{3} s\, t12\, t22^{2} \alpha^{2} -$
$t12^{3} t22^{2} \alpha^{2} + 4 m\, t12^{3} t22^{2} \alpha^{2} - 4 m^{2} t12^{3} t22^{2} \alpha^{2} - 12 m^{3} t12^{3} t22^{2} \alpha^{2} + 30 m^{4} t12^{3} t22^{2} \alpha^{2} -$
$20 m^{5} t12^{3} t22^{2} \alpha^{2} - s\, t12^{3} t22^{2} \alpha^{2} + 4 m\, s\, t12^{3} t22^{2} \alpha^{2} - 4 m^{2} s\, t12^{3} t22^{2} \alpha^{2} - 12 m^{3} s\, t12^{3} t22^{2} \alpha^{2} +$
$30 m^{4} s\, t12^{3} t22^{2} \alpha^{2} - 20 m^{5} s\, t12^{3} t22^{2} \alpha^{2} + m\, t22^{3} \alpha^{2} + m^{2} t22^{3} \alpha^{2} - 2 m^{3} t22^{3} \alpha^{2} + m\, s\, t22^{3} \alpha^{2} +$
$m^{2} s\, t22^{3} \alpha^{2} - 2 m^{3} s\, t22^{3} \alpha^{2} - t12\, t22^{3} \alpha^{2} + 2 m^{2} t12\, t22^{3} \alpha^{2} - 4 m^{3} t12\, t22^{3} \alpha^{2} - s\, t12\, t22^{3} \alpha^{2} +$
$2 m^{2} s\, t12\, t22^{3} \alpha^{2} - 4 m^{3} s\, t12\, t22^{3} \alpha^{2} + t12^{2} t22^{3} \alpha^{2} - 4 m\, t12^{2} t22^{3} \alpha^{2} + 4 m^{2} t12^{2} t22^{3} \alpha^{2} +$
$12 m^{3} t12^{2} t22^{3} \alpha^{2} - 30 m^{4} t12^{2} t22^{3} \alpha^{2} + 20 m^{5} t12^{2} t22^{3} \alpha^{2} + s\, t12^{2} t22^{3} \alpha^{2} - 4 m\, s\, t12^{2} t22^{3} \alpha^{2} +$
$4 m^{2} s\, t12^{2} t22^{3} \alpha^{2} + 12 m^{3} s\, t12^{2} t22^{3} \alpha^{2} - 30 m^{4} s\, t12^{2} t22^{3} \alpha^{2} + 20 m^{5} s\, t12^{2} t22^{3} \alpha^{2} -$
$m\, t22^{4} \alpha^{2} - m^{2} t22^{4} \alpha^{2} + 2 m^{3} t22^{4} \alpha^{2} - m\, s\, t22^{4} \alpha^{2} - m^{2} s\, t22^{4} \alpha^{2} + 2 m^{3} s\, t22^{4} \alpha^{2} + 2 m\, t12\, t22^{4} \alpha^{2} -$
$3 m^{2} t12\, t22^{4} \alpha^{2} - 4 m^{3} t12\, t22^{4} \alpha^{2} + 15 m^{4} t12\, t22^{4} \alpha^{2} - 10 m^{5} t12\, t22^{4} \alpha^{2} + 2 m\, s\, t12\, t22^{4} \alpha^{2} -$
$3 m^{2} s\, t12\, t22^{4} \alpha^{2} - 4 m^{3} s\, t12\, t22^{4} \alpha^{2} + 15 m^{4} s\, t12\, t22^{4} \alpha^{2} - 10 m^{5} s\, t12\, t22^{4} \alpha^{2} +$
$m^{2} t22^{5} \alpha^{2} - 3 m^{4} t22^{5} \alpha^{2} + 2 m^{5} t22^{5} \alpha^{2} + m^{2} s\, t22^{5} \alpha^{2} - 3 m^{4} s\, t22^{5} \alpha^{2} + 2 m^{5} s\, t22^{5} \alpha^{2})$

This equals 0 if t12 = t22 or if t12 = 1 - t22 (or if m=1/2). The linear factors depending on m do not give admissible nontrivial solutions (as is easily shown). The complicated factor can be solved for t12 or t22. Because this factor is a polynomial, generically, it has finitely many solutions for given t22 (or t12). Therefore, generically, there will be finitely many solutions of the equilibrium conditions (factdelta3maler1/.p12p22genmt12/.dd12genmt12)=0 and (factdelta6maler1/.p12p22genmt12/.dd12genmt12)=0, and not a curve or manifold (as when selection acts on both sexes). At least one equilibrium is symmetric, i.e., satisfied t12=1-t22.

```
Simplify[
 Numerator[Factor[factdelta3msymr1 /. p12p22genmt12 /. dd12genmt12 /. t12 → 1 - t22 /.
     sympar]] - Numerator[
   Factor[factdelta6msymr1 /. p12p22genmt12 /. dd12genmt12 /. t12 → 1 - t22 /. sympar]]]
```

0

Thus, if t12 = 1 - t22, the two conditions collapse to one, as it should be. Here is the polynomial in t22, whose zeros give rise to the potential equilibria (but not all solutions t22 may give rise to admissible equilibria)

```
symfacdelta3 =
 Simplify[factdelta3msymr1 /. p12p22genmt12 /. dd12genmt12 /. t12 → 1 - t22 /. sympar]
```

```
Exponent[Numerator[Factor[symfacdelta3]], t22]
```

7

This is a polynomial of degree 7. Hence, there may be up to 7 zeros (potentially giving rise to equilibria).
Study this polynomial:

```
Factor[Numerator[Factor[symfacdelta3]] /. t22 → 0]
```

$$
\begin{aligned}
m^2 \big(&4 - 24 m + 16 m^2 + 4 s - 16 m s - 44 m^2 s + 40 m^3 s + s^2 + 2 m s^2 - 47 m^2 s^2 - 12 m^3 s^2 + 32 m^4 s^2 + 2 m s^3 - \\
&8 m^2 s^3 - 30 m^3 s^3 + 12 m^4 s^3 + 8 m^5 s^3 + m^2 s^4 - 6 m^3 s^4 - 3 m^4 s^4 + 4 m^5 s^4 + 8 \alpha - 48 m \alpha + \\
&32 m^2 \alpha + 8 s \alpha - 32 m s \alpha - 88 m^2 s \alpha + 80 m^3 s \alpha + 2 s^2 \alpha + 4 m s^2 \alpha - 94 m^2 s^2 \alpha - 24 m^3 s^2 \alpha + \\
&64 m^4 s^2 \alpha + 4 m s^3 \alpha - 16 m^2 s^3 \alpha - 60 m^3 s^3 \alpha + 24 m^4 s^3 \alpha + 16 m^5 s^3 \alpha + 2 m^2 s^4 \alpha - 12 m^3 s^4 \alpha - \\
&6 m^4 s^4 \alpha + 8 m^5 s^4 \alpha + 5 \alpha^2 - 30 m \alpha^2 + 5 m^2 \alpha^2 + 28 m^3 \alpha^2 - 12 m^4 \alpha^2 + 5 s \alpha^2 - 20 m s \alpha^2 - \\
&83 m^2 s \alpha^2 + 94 m^3 s \alpha^2 - 4 m^4 s \alpha^2 - 8 m^5 s \alpha^2 + s^2 \alpha^2 + 5 m s^2 \alpha^2 - 81 m^2 s^2 \alpha^2 + 11 m^3 s^2 \alpha^2 + \\
&52 m^4 s^2 \alpha^2 - 12 m^5 s^2 \alpha^2 + 3 m s^3 \alpha^2 - 14 m^2 s^3 \alpha^2 - 33 m^3 s^3 \alpha^2 + 24 m^4 s^3 \alpha^2 + 4 m^5 s^3 \alpha^2 + \\
&m^2 s^4 \alpha^2 - 6 m^3 s^4 \alpha^2 - 3 m^4 s^4 \alpha^2 + 4 m^5 s^4 \alpha^2 + \alpha^3 - 6 m \alpha^3 - 11 m^2 \alpha^3 + 28 m^3 \alpha^3 - 12 m^4 \alpha^3 + s \alpha^3 - \\
&4 m s \alpha^3 - 39 m^2 s \alpha^3 + 54 m^3 s \alpha^3 - 4 m^4 s \alpha^3 - 8 m^5 s \alpha^3 + 3 m s^2 \alpha^3 - 34 m^2 s^2 \alpha^3 + 23 m^3 s^2 \alpha^3 + \\
&20 m^4 s^2 \alpha^3 - 12 m^5 s^2 \alpha^3 + m s^3 \alpha^3 - 6 m^2 s^3 \alpha^3 - 3 m^3 s^3 \alpha^3 + 12 m^4 s^3 \alpha^3 - 4 m^5 s^3 \alpha^3 - 4 m^2 \alpha^4 + \\
&8 m^3 \alpha^4 - 4 m^4 \alpha^4 - 8 m^2 s \alpha^4 + 16 m^3 s \alpha^4 - 8 m^4 s \alpha^4 - 4 m^2 s^2 \alpha^4 + 8 m^3 s^2 \alpha^4 - 4 m^4 s^2 \alpha^4\big)
\end{aligned}
$$

This is positive if m and s are small and α is not too large!

```
Factor[Numerator[Factor[symfacdelta3]] /. t22 → 1/2]
```

$$-\frac{1}{64} s^2 (2 + s)^2 (1 + \alpha)^2$$

This is always negative!

```
Factor[Numerator[Factor[symfacdelta3]] /. t22 → 1]
```

$$-m^2 \left(-4 + 24 m - 16 m^2 - 12 s + 80 m s - 108 m^2 s + 40 m^3 s - 13 s^2 + 94 m s^2 - 181 m^2 s^2 + 132 m^3 s^2 - 32 m^4 s^2 - 6 s^3 + 46 m s^3 - 110 m^2 s^3 + 114 m^3 s^3 - 52 m^4 s^3 + 8 m^5 s^3 - s^4 + 8 m s^4 - 22 m^2 s^4 + 28 m^3 s^4 - 17 m^4 s^4 + 4 m^5 s^4 - 8 \alpha + 48 m \alpha - 32 m^2 \alpha - 24 s \alpha + 160 m s \alpha - 216 m^2 s \alpha + 80 m^3 s \alpha - 26 s^2 \alpha + 188 m s^2 \alpha - 362 m^2 s^2 \alpha + 264 m^3 s^2 \alpha - 64 m^4 s^2 \alpha - 12 s^3 \alpha + 92 m s^3 \alpha - 220 m^2 s^3 \alpha + 228 m^3 s^3 \alpha - 104 m^4 s^3 \alpha + 16 m^5 s^3 \alpha - 2 s^4 \alpha + 16 m s^4 \alpha - 44 m^2 s^4 \alpha + 56 m^3 s^4 \alpha - 34 m^4 s^4 \alpha + 8 m^5 s^4 \alpha - 5 \alpha^2 + 30 m \alpha^2 - 5 m^2 \alpha^2 - 28 m^3 \alpha^2 + 12 m^4 \alpha^2 - 15 s \alpha^2 + 100 m s \alpha^2 - 103 m^2 s \alpha^2 - 18 m^3 s \alpha^2 + 44 m^4 s \alpha^2 - 8 m^5 s \alpha^2 - 16 s^2 \alpha^2 + 115 m s^2 \alpha^2 - 198 m^2 s^2 \alpha^2 + 103 m^3 s^2 \alpha^2 + 8 m^4 s^2 \alpha^2 - 12 m^5 s^2 \alpha^2 - 7 s^3 \alpha^2 + 53 m s^3 \alpha^2 - 121 m^2 s^3 \alpha^2 + 115 m^3 s^3 \alpha^2 - 44 m^4 s^3 \alpha^2 + 4 m^5 s^3 \alpha^2 - s^4 \alpha^2 + 8 m s^4 \alpha^2 - 22 m^2 s^4 \alpha^2 + 28 m^3 s^4 \alpha^2 - 17 m^4 s^4 \alpha^2 + 4 m^5 s^4 \alpha^2 - \alpha^3 + 6 m \alpha^3 + 11 m^2 \alpha^3 - 28 m^3 \alpha^3 + 12 m^4 \alpha^3 - 3 s \alpha^3 + 20 m s \alpha^3 + 5 m^2 s \alpha^3 - 58 m^3 s \alpha^3 + 44 m^4 s \alpha^3 - 8 m^5 s \alpha^3 - 3 s^2 \alpha^3 + 21 m s^2 \alpha^3 - 17 m^2 s^2 \alpha^3 - 29 m^3 s^2 \alpha^3 + 40 m^4 s^2 \alpha^3 - 12 m^5 s^2 \alpha^3 - s^3 \alpha^3 + 7 m s^3 \alpha^3 - 11 m^2 s^3 \alpha^3 + m^3 s^3 \alpha^3 + 8 m^4 s^3 \alpha^3 - 4 m^5 s^3 \alpha^3 + 4 m^2 \alpha^4 - 8 m^3 \alpha^4 + 4 m^4 \alpha^4 + 8 m^2 s \alpha^4 - 16 m^3 s \alpha^4 + 8 m^4 s \alpha^4 + 4 m^2 s^2 \alpha^4 - 8 m^3 s^2 \alpha^4 + 4 m^4 s^2 \alpha^4\right)$$

This is positive if m and s are small and α is not too large!

Therefore, if m and s are small and α is not too large, then there is one solution satisfying 0 < t22 < 0.5 and a second solution satisfying 0.5 < t22 < 1.

Numerical examples. Compute t22 for various parameter combinations; then compute (p22,t22,dd2):

```
Select[
 t22 /. NSolve[(symfacdelta3 /. {s → 0.2, m → 0.05, α → 1}) == 0, t22, Reals], 0 < # < 1 &]
```

```
{0.472115, 0.651443}
```

```
N[{p22, t22, dd2} /. p12p22genmt12 /. dd12genmt12 /. sympar /. {s → 0.2, m → 0.05, α → 1} /.
   t12 → 1 - t22 /. {t22 → %}]
```

```
{{0.336905, 0.525193}, {0.472115, 0.651443}, {1.45769, 0.0377726}}
```

Only second solution admissible

```
Select[
 t22 /. NSolve[(symfacdelta3 /. {s → 0.2, m → 0.05, α → 1}) == 0, t22, Reals], 0 < # < 1 &]
```

```
{0.472115, 0.651443}
```

```
N[{p22, t22, dd2} /. p12p22genmt12 /. dd12genmt12 /. sympar /. {s → 0.2, m → 0.05, α → 1} /.
   t12 → 1 - t22 /. {t22 → %}]
```

```
{{0.336905, 0.525193}, {0.472115, 0.651443}, {1.45769, 0.0377726}}
```

Only second solution admissible

```
Select[
 t22 /. NSolve[(symfacdelta3 /. {s → 0.2, m → 0.05, α → 68}) == 0, t22, Reals], 0 < # < 1 &]
```

```
{0.460396, 0.50709}
```

```
N[{p22, t22, dd2} /. p12p22genmt12 /. dd12genmt12 /. sympar /.
    {s → 0.2, m → 0.05, α → 68} /. t12 → 1 - t22 /. {t22 → %}]
```

```
{{0.449534, 0.505554}, {0.460396, 0.50709}, {0.316568, 0.195795}}
```

Only second solution admissible

```
Select[
 t22 /. NSolve[(symfacdelta3 /. {s → 0.2, m → 0.05, α → 69}) == 0, t22, Reals], 0 < # < 1 &]
{0.00142376, 0.459558, 0.507006}

N[{p22, t22, dd2} /. p12p22genmt12 /. dd12genmt12 /. sympar /.
    {s → 0.2, m → 0.05, α → 69} /. t12 → 1 - t22 /. {t22 → %}]
{{-0.122554, 0.448558, 0.505498},
 {0.00142376, 0.459558, 0.507006}, {0.00177527, 0.315918, 0.196143}}
```

Only third solution admissible

```
Select[
 t22 /. NSolve[(symfacdelta3 /. {s → 0.2, m → 0.05, α → 90}) == 0, t22, Reals], 0 < # < 1 &]
{0.0971816, 0.419647, 0.505647}

N[{p22, t22, dd2} /. p12p22genmt12 /. dd12genmt12 /. sympar /.
    {s → 0.2, m → 0.05, α → 90} /. t12 → 1 - t22 /. {t22 → %}]
{{0.0089782, 0.400929, 0.504566},
 {0.0971816, 0.419647, 0.505647}, {0.106949, 0.300276, 0.202146}}
```

Only third solution admissible

```
Select[
 t22 /. NSolve[(symfacdelta3 /. {s → 0.2, m → 0.05, α → 94.3}) == 0, t22, Reals], 0 < # < 1 &]
{0.161008, 0.39319, 0.505436, 0.99997}

N[{p22, t22, dd2} /. p12p22genmt12 /. dd12genmt12 /. sympar /.
    {s → 0.2, m → 0.05, α → 94.3} /. t12 → 1 - t22 /. {t22 → %}]
{{0.0877299, 0.369058, 0.504417, 1.12502},
 {0.161008, 0.39319, 0.505436, 0.99997}, {0.164285, 0.292497, 0.203138, 0.0000380353}}
```

Only third solution admissible

```
Select[t22 /. NSolve[(symfacdelta3 /. {s → 0.2, m → 0.05, α → 97.95}) == 0, t22, Reals],
 0 < # < 1 &]
{0.299276, 0.30306, 0.50527, 0.995122}

N[{p22, t22, dd2} /. p12p22genmt12 /. dd12genmt12 /. sympar /.
    {s → 0.2, m → 0.05, α → 97.95} /. t12 → 1 - t22 /. {t22 → %}]
{{0.255633, 0.260209, 0.504299, 1.11629},
 {0.299276, 0.30306, 0.50527, 0.995122}, {0.255308, 0.257172, 0.203932, 0.00604205}}
```

Only third solution admissible

```
Select[t22 /. NSolve[(symfacdelta3 /. {s → 0.2, m → 0.05, α → 97.96}) == 0, t22, Reals],
 0 < # < 1 &]
{0.505269, 0.995107}

N[{p22, t22, dd2} /. p12p22genmt12 /. dd12genmt12 /. sympar /.
    {s → 0.2, m → 0.05, α → 97.96} /. t12 → 1 - t22 /. {t22 → %}]
{{0.504299, 1.11627}, {0.505269, 0.995107}, {0.203934, 0.00606001}}
```

Only first solution admissible

```
Select[
 t22 /. NSolve[(symfacdelta3 /. {s → 0.2, m → 0.05, α → 100}) == 0, t22, Reals], 0 < # < 1 &]
{0.505181, 0.991945}
```

```
N[{p22, t22, dd2} /. p12p22genmt12 /. dd12genmt12 /. sympar /.
   {s → 0.2, m → 0.05, α → 100} /. t12 → 1 - t22 /. {t22 → %}]
{{0.504236, 1.11091}, {0.505181, 0.991945}, {0.20436, 0.00992283}}
```

Only first solution admissible

```
Select[
 t22 /. NSolve[(symfacdelta3 /. {s → 0.2, m → 0.05, α → 125}) == 0, t22, Reals], 0 < # < 1 &]
{0.504314, 0.747811}
```

```
N[{p22, t22, dd2} /. p12p22genmt12 /. dd12genmt12 /. sympar /.
   {s → 0.2, m → 0.05, α → 125} /. t12 → 1 - t22 /. {t22 → %}]
{{0.503603, 0.79881}, {0.504314, 0.747811}, {0.208752, 0.227402}}
```

Only first solution admissible

```
Select[
 t22 /. NSolve[(symfacdelta3 /. {s → 0.2, m → 0.05, α → 1000}) == 0, t22, Reals], 0 < # < 1 &]
{0.500721, 0.501315}
```

```
N[{p22, t22, dd2} /. p12p22genmt12 /. dd12genmt12 /. sympar /.
   {s → 0.2, m → 0.05, α → 1000} /. t12 → 1 - t22 /. {t22 → %}]
{{0.500676, 0.501401}, {0.500721, 0.501315}, {0.234556, 0.266461}}
```

Only first solution admissible

```
Select[
 t22 /. NSolve[(symfacdelta3 /. {s → 0.2, m → 0.01, α → 1000}) == 0, t22, Reals], 0 < # < 1 &]
{0.493048, 0.501857}
```

```
N[{p22, t22, dd2} /. p12p22genmt12 /. dd12genmt12 /. sympar /.
   {s → 0.2, m → 0.01, α → 1000} /. t12 → 1 - t22 /. {t22 → %}]
{{0.492591, 0.501742}, {0.493048, 0.501857}, {0.266387, 0.23455}}
```

Only second solution admissible

```
Select[t22 /. NSolve[(symfacdelta3 /. {s → 0.2, m → 0.01, α → 100 000}) == 0, t22, Reals],
 0 < # < 1 &]
{0.50004, 0.500054}
```

```
N[{p22, t22, dd2} /. p12p22genmt12 /. dd12genmt12 /. sympar /.
   {s → 0.2, m → 0.01, α → 100 000} /. t12 → 1 - t22 /. {t22 → %}]
{{0.500039, 0.500055}, {0.50004, 0.500054}, {0.248411, 0.251599}}
```

Only first solution admissible

2.2.4 Summary for symmetric parameters

In all cases, numerics suggests that only one (symmetric) equilibrium exists (see below), although there may be up to four solutions for t22 between 0 and 1.

If α is small enough such that there is one solution t22 with 0 < t22 < 0.5, and another with 0.5 < t22 < 1, then only the second gives rise to an (admissible) equilibrium.

If alpha is very large, then numerics suggests that there are between two and four solutions t22. Numerics suggests that there is exactly one admissible equilibrium, and it arises from the the value t22 that is closest to 0.5 (and >0.5).

All our numerical results from iteration of the recursion relations show that there is a single stable polymorphic equilibrium.

2.2.5 Efficient numerical computation of equilibria with symmetric parameters

One can use FindRoot or NSolve (the latter is efficient only in the symmetric case)

The following assumes symmetric parameters, but not t12 = 1 - t22:

```
Table[
 FindRoot[{factdelta3maler1 /. p12p22genmt12 /. dd12genmt12 /. sympar /. {s → 0.2, α → αα,
      m → 0.01}, factdelta6maler1 /. p12p22genmt12 /. dd12genmt12 /. sympar /.
     {s → 0.2, α → αα, m → 0.01}} == 0, {{t12, 0.45}, {t22, 0.6}}],
 {αα, {0.001, 0.01, 0.05, 0.1, 0.25, 0.5, 0.75, 1}}]
```

... FindRoot: Failed to converge to the requested accuracy or precision within 100 iterations.

... FindRoot: Failed to converge to the requested accuracy or precision within 100 iterations.

```
{{t12 → 0.098712, t22 → 0.902744}, {t12 → 0.0983662, t22 → 0.902383},
 {t12 → 0.0982358, t22 → 0.901764}, {t12 → 0.0989614, t22 → 0.901039},
 {t12 → 0.103525, t22 → 0.896475}, {t12 → 0.117885, t22 → 0.882115},
 {t12 → 0.139931, t22 → 0.860069}, {t12 → 0.168621, t22 → 0.831379}}
```

The following yields all possible values for t22 (by solving factdelta3maler1=0). Not all of them yield equilibria!

```
Table[
 Select[t22 /. NSolve[{factdelta3maler1 /. p12p22genmt12 /. dd12genmt12 /. t12 → 1 - t22 /.
       {s → 0.2, α → αα, m → 0.01}} == 0, t22, Reals],
  1 > # > 0 &], {αα, {0.001, 0.01, 0.05, 0.1, 0.25, 0.5, 0.75, 1}}]
```

```
{{0.476327, 0.90202}, {0.476327, 0.90201}, {0.47633, 0.901764}, {0.476339, 0.901039},
 {0.476396, 0.896475}, {0.476553, 0.882115}, {0.476755, 0.860069}, {0.47698, 0.831379}}
```

The above routine based on FindRoot yields always symmetric equilibrium. Assuming symmetry t12 = 1 - t22., we can use NSolve efficiently to obtain more accurate values in a shorter time.
The following computes the admissible polymorphic symmetric equilibria for the values α specified (in this first case : $\alpha \in$ {0.001, 0.01, 0.05, 0.1, 0.25, 0.5, 0.75, 1}):

```
params[αα_] := {s1 → 0.2, s2 → 0.2, m1 → 0.01, m2 → 0.01, α1 → αα, α2 → αα};
Table[t22val = Select[
   t22 /. NSolve[{factdelta3maler1 /. p12p22genmt12 /. dd12genmt12 /. t12 → 1 - t22 /.
        params[αα]} == 0, t22, Reals], 0 < # < 1 &];
 Flatten[Table[Select[{{p22, t22val[[i]], dd2}} /. p12p22genmt12 /. dd12genmt12 /.
       {t12 → 1 - t22val[[i]], t22 → t22val[[i]]} /. params[αα],
     0 < #[[1]] < 1 && 0 < #[[2]] < 1 && -Min[(1 - #[[1]]) (1 - #[[2]]), #[[1]] #[[2]]] ≤
        #[[3]] ≤ Min[#[[1]] (1 - #[[2]]), #[[2]] (1 - #[[1]])] &],
   {i, 1, Length[t22val]}], 2], {αα, {0.001, 0.01, 0.05, 0.1, 0.25, 0.5, 0.75, 1}}]
```

```
{{0.500093, 0.90202, 0.0000204374}, {0.500925, 0.90201, 0.00020348},
 {0.504535, 0.901764, 0.000999981}, {0.508844, 0.901039, 0.00196637},
 {0.520476, 0.896475, 0.00479307}, {0.535896, 0.882115, 0.00976864},
 {0.546767, 0.860069, 0.0156316}, {0.553442, 0.831379, 0.0226084}}
```

The following uses FindRoot (and can also detect asymmetric equilibria) and gives only (t12,t22) (further below, in the asymmetric case, there is a routine giving all coordinates):

```
Table[
 FindRoot[{factdelta3maler1 /. p12p22genmt12 /. dd12genmt12 /. sympar /. {s → 0.2, α → αα,
       m → 0.01}, factdelta6maler1 /. p12p22genmt12 /. dd12genmt12 /. sympar /.
      {s → 0.2, α → αα, m → 0.01}} == 0, {{t12, 0.4}, {t22, 0.59}}],
 {αα, {0.001, 0.01, 0.05, 0.1, 0.25, 0.5, 0.75, 1}}]
```

... FindRoot: Failed to converge to the requested accuracy or precision within 100 iterations.

... FindRoot: Failed to converge to the requested accuracy or precision within 100 iterations.

```
{{t12 → 0.0978138, t22 → 0.901853}, {t12 → 0.0978451, t22 → 0.901864},
 {t12 → 0.0982358, t22 → 0.901764}, {t12 → 0.0989614, t22 → 0.901039},
 {t12 → 0.103525, t22 → 0.896475}, {t12 → 0.117885, t22 → 0.882115},
 {t12 → 0.139931, t22 → 0.860069}, {t12 → 0.168621, t22 → 0.831379}}
```

```
params[αα_] := {s1 → 0.2, s2 → 0.2, m1 → 0.01, m2 → 0.01, α1 → αα, α2 → αα};
Table[t22val = Select[
   t22 /. NSolve[{factdelta3maler1 /. p12p22genmt12 /. dd12genmt12 /. t12 → 1 - t22 /.
        params[αα]} == 0, t22, Reals], 0 < # < 1 &];
 Flatten[Table[Select[{{p22, t22val[[i]], dd2}} /. p12p22genmt12 /. dd12genmt12 /.
       {t12 → 1 - t22val[[i]], t22 → t22val[[i]]} /. params[αα],
     0 < #[[1]] < 1 && 0 < #[[2]] < 1 && -Min[(1 - #[[1]]) (1 - #[[2]]), #[[1]] #[[2]]] ≤
        #[[3]] ≤ Min[#[[1]] (1 - #[[2]]), #[[2]] (1 - #[[1]])] &],
   {i, 1, Length[t22val]}], 2], {αα, 1, 10, 1}]
```

```
{{0.553442, 0.831379, 0.0226084}, {0.554653, 0.712148, 0.0528098},
 {0.54738, 0.646059, 0.0741773}, {0.541623, 0.611262, 0.088893},
 {0.537354, 0.590418, 0.0999034}, {0.534092, 0.576623, 0.108622},
 {0.531514, 0.566826, 0.11579}, {0.529416, 0.559502, 0.121844},
 {0.52767, 0.553812, 0.127059}, {0.526187, 0.549256, 0.131621}}
```

```
Table[
 FindRoot[{factdelta3maler1 /. p12p22genmt12 /. dd12genmt12 /. sympar /. {s → 0.2, α → αα,
      m → 0.01}, factdelta6maler1 /. p12p22genmt12 /. dd12genmt12 /. sympar /.
     {s → 0.2, α → αα, m → 0.01}} == 0, {{t12, 0.4}, {t22, 0.59}}], {αα, 1, 10, 1}]
```

```
{{t12 → 0.168621, t22 → 0.831379}, {t12 → 0.287852, t22 → 0.712148},
 {t12 → 0.353941, t22 → 0.646059}, {t12 → 0.388738, t22 → 0.611262},
 {t12 → 0.409582, t22 → 0.590418}, {t12 → 0.423377, t22 → 0.576623},
 {t12 → 0.433174, t22 → 0.566826}, {t12 → 0.440498, t22 → 0.559502},
 {t12 → 0.446188, t22 → 0.553812}, {t12 → 0.450744, t22 → 0.549256}}
```

```
params[αα_] := {s1 → 0.2, s2 → 0.2, m1 → 0.01, m2 → 0.01, α1 → αα, α2 → αα};
Table[t22val = Select[
   t22 /. NSolve[{factdelta3maler1 /. p12p22genmt12 /. dd12genmt12 /. t12 → 1 - t22 /.
        params[αα]} == 0, t22, Reals], 0 < # < 1 &];
 Flatten[Table[Select[{{p22, t22val[[i]], dd2}} /. p12p22genmt12 /. dd12genmt12 /.
      {t12 → 1 - t22val[[i]], t22 → t22val[[i]]} /. params[αα],
     0 < #[[1]] < 1 && 0 < #[[2]] < 1 && -Min[(1 - #[[1]]) (1 - #[[2]]), #[[1]] #[[2]]] ≤
       #[[3]] ≤ Min[#[[1]] (1 - #[[2]]), #[[2]] (1 - #[[1]])] &],
   {i, 1, Length[t22val]}], 2], {αα, 10, 100, 10}]
```

```
{{0.526187, 0.549256, 0.131621}, {0.518125, 0.5284, 0.159031},
 {0.514554, 0.521014, 0.172838}, {0.512429, 0.517089, 0.181619},
 {0.510983, 0.514603, 0.187862}, {0.509917, 0.512863, 0.192606},
 {0.50909, 0.511566, 0.196374}, {0.508425, 0.510555, 0.199465},
 {0.507875, 0.50974, 0.202061}, {0.507411, 0.509067, 0.204284}}
```

For larger α (and FindRoot), it is important to have initial values close to 0.5 (but not both at 0.5 because the denominator vanishes if t12=t22)

```
Table[FindRoot[
  {factdelta3maler1 /. p12p22genmt12 /. dd12genmt12 /. {s → 0.2, α → αα, m → 0.01},
    factdelta6maler1 /. p12p22genmt12 /. dd12genmt12 /. {s → 0.2, α → αα, m → 0.01}} == 0,
  {{t12, 0.49}, {t22, 0.52}}], {αα, 10, 100, 10}]
```

```
{{t12 → 0.450744, t22 → 0.549256}, {t12 → 0.4716, t22 → 0.5284},
 {t12 → 0.478986, t22 → 0.521014}, {t12 → 0.482911, t22 → 0.517089},
 {t12 → 0.485397, t22 → 0.514603}, {t12 → 0.487137, t22 → 0.512863},
 {t12 → 0.488434, t22 → 0.511566}, {t12 → 0.489445, t22 → 0.510555},
 {t12 → 0.49026, t22 → 0.50974}, {t12 → 0.490933, t22 → 0.509067}}
```

```
params[αα_] := {s1 → 0.2, s2 → 0.2, m1 → 0.01, m2 → 0.01, α1 → αα, α2 → αα};
Table[t22val = Select[
   t22 /. NSolve[{factdelta3maler1 /. p12p22genmt12 /. dd12genmt12 /. t12 → 1 - t22 /.
        params[αα]} == 0, t22, Reals], 0 < # < 1 &];
 Flatten[Table[Select[{{p22, t22val[[i]], dd2}} /. p12p22genmt12 /. dd12genmt12 /.
      {t12 → 1 - t22val[[i]], t22 → t22val[[i]]} /. params[αα],
     0 < #[[1]] < 1 && 0 < #[[2]] < 1 && -Min[(1 - #[[1]]) (1 - #[[2]]), #[[1]] #[[2]]] ≤
       #[[3]] ≤ Min[#[[1]] (1 - #[[2]]), #[[2]] (1 - #[[1]])] &],
   {i, 1, Length[t22val]}], 2], {αα, 100, 1000, 100}]
```

```
{{0.507411, 0.509067, 0.204284}, {0.504919, 0.505673, 0.216745},
 {0.503834, 0.504308, 0.222503}, {0.503198, 0.503537, 0.226006},
 {0.50277, 0.503031, 0.228428}, {0.502458, 0.502669, 0.230233},
 {0.502219, 0.502395, 0.231645}, {0.502029, 0.502178, 0.232789},
 {0.501872, 0.502003, 0.233741}, {0.501742, 0.501857, 0.23455}}
```

```
Table[
 FindRoot[{factdelta3maler1 /. p12p22genmt12 /. dd12genmt12 /. sympar /. {s → 0.2, α → αα,
      m → 0.01}, factdelta6maler1 /. p12p22genmt12 /. dd12genmt12 /. sympar /.
     {s → 0.2, α → αα, m → 0.01}} == 0,
  {{t12, 0.498}, {t22, 0.501}}], {αα, 100, 1000, 100}]
```

```
{{t12 → 0.490933, t22 → 0.509067}, {t12 → 0.494327, t22 → 0.505673},
 {t12 → 0.495692, t22 → 0.504308}, {t12 → 0.496463, t22 → 0.503537},
 {t12 → 0.496969, t22 → 0.503031}, {t12 → 0.497331, t22 → 0.502669},
 {t12 → 0.497605, t22 → 0.502395}, {t12 → 0.497822, t22 → 0.502178},
 {t12 → 0.497997, t22 → 0.502003}, {t12 → 0.498143, t22 → 0.501857}}
```

```
params[αα_] := {s1 → 0.2, s2 → 0.2, m1 → 0.01, m2 → 0.01, α1 → αα, α2 → αα};
Table[t22val = Select[
   t22 /. NSolve[{factdelta3maler1 /. p12p22genmt12 /. dd12genmt12 /. t12 → 1 - t22 /.
        params[αα]} == 0, t22, Reals], 0 < # < 1 &];
 Flatten[Table[Select[{{p22, t22val[[i]], dd2}} /. p12p22genmt12 /. dd12genmt12 /.
      {t12 → 1 - t22val[[i]], t22 → t22val[[i]]} /. params[αα],
     0 < #[[1]] < 1 && 0 < #[[2]] < 1 && -Min[(1 - #[[1]]) (1 - #[[2]]), #[[1]] #[[2]]] ≤
       #[[3]] ≤ Min[#[[1]] (1 - #[[2]]), #[[2]] (1 - #[[1]])] &],
   {i, 1, Length[t22val]}], 2], {αα, {1000, 10000, 100000}}]
```

```
{{0.501742, 0.501857, 0.23455},
 {0.500303, 0.500309, 0.245009}, {0.500039, 0.50004, 0.248411}}
```

The above shows that always a single admissible polymorphic equilibrium is found.

2.2.6 Asymmetric parameters

The above routine with FindRoot can also be used to find the equilibrium for asymmetric parameters (below, we also compute all coordinates and check admissibility):

```
params[αα_] := {s1 → 0.1, s2 → 0.2, m1 → 0.01, m2 → 0.01, α1 → 1.1 αα, α2 → αα};
Table[t12t22 = FindRoot[{factdelta3maler1 /. p12p22genmt12 /. dd12genmt12 /. params[αα],
     factdelta6maler1 /. p12p22genmt12 /. dd12genmt12 /. params[αα]} == 0,
   {{t12, 0.45}, {t22, 0.56}}]; Flatten[
  Select[{{p12, t12, dd1, p22, t22, dd2}} /. p12p22genmt12 /. dd12genmt12 /. t12t22 /.
    params[αα], 0 < #[[1]] < 1 && 0 < #[[2]] < 1 &&
     -Min[(1 - #[[1]]) (1 - #[[2]]), #[[1]] #[[2]]] ≤ #[[3]] ≤ Min[#[[1]] (1 - #[[2]]),
       #[[2]] (1 - #[[1]])] && 0 < #[[4]] < 1 && 0 < #[[5]] < 1 &], 1], {αα, 1, 10, 1}]
```

```
params[αα_] := {s1 → 0.1, s2 → 0.2, m1 → 0.01, m2 → 0.01, α1 → 10 αα, α2 → 10};
Table[t12t22 = FindRoot[{factdelta3maler1 /. p12p22genmt12 /. dd12genmt12 /. params[αα],
      factdelta6maler1 /. p12p22genmt12 /. dd12genmt12 /. params[αα]} == 0,
    {{t12, 0.45}, {t22, 0.56}}]; Flatten[
   Select[{{p12, t12, dd1, p22, t22, dd2}} /. p12p22genmt12 /. dd12genmt12 /. t12t22 /.
      params[αα],
     0 < #[[1]] < 1 && 0 < #[[2]] < 1 && -Min[(1 - #[[1]]) (1 - #[[2]]), #[[1]] #[[2]]] ≤
       #[[3]] ≤ Min[#[[1]] (1 - #[[2]]), #[[2]] (1 - #[[1]])] &&
      0 < #[[4]] < 1 && 0 < #[[5]] < 1 &], 1], {αα, 1, 2, 0.1}]

{{0.448264, 0.429257, 0.130693, 0.488207, 0.504134, 0.133354},
 {0.480468, 0.463931, 0.134674, 0.519532, 0.536069, 0.134674},
 {0.509607, 0.495146, 0.137072, 0.547686, 0.56459, 0.134798},
 {0.536013, 0.523308, 0.138245, 0.573052, 0.590143, 0.134043},
 {0.559995, 0.548783, 0.138479, 0.595974, 0.613117, 0.132654},
 {0.581829, 0.571893, 0.138, 0.616749, 0.633846, 0.130815},
 {0.601761, 0.592923, 0.136982, 0.635639, 0.652617, 0.128664},
 {0.620005, 0.612116, 0.135565, 0.652869, 0.669675, 0.126304},
 {0.636751, 0.629687, 0.133853, 0.668633, 0.685228, 0.123813},
 {0.652163, 0.645818, 0.131931, 0.6831, 0.699456, 0.121249},
 {0.666384, 0.66067, 0.12986, 0.696414, 0.712512, 0.118654}}
```

For large α, numerical problems may occur:

```
params[αα_] := {s1 → 0.2, s2 → 0.2, m1 → 0.01, m2 → 0.01, α1 → 1.1 αα, α2 → αα};
Table[t12t22 = FindRoot[{factdelta3maler1 /. p12p22genmt12 /. dd12genmt12 /. params[αα],
      factdelta6maler1 /. p12p22genmt12 /. dd12genmt12 /. params[αα]} == 0,
    {{t12, 0.49}, {t22, 0.51}}, MaxIterations → 200, PrecisionGoal → 8];
  Flatten[Select[{{p12, t12, dd1, p22, t22, dd2}} /. p12p22genmt12 /. dd12genmt12 /.
      t12t22 /. params[αα],
     0 < #[[1]] < 1 && 0 < #[[2]] < 1 && -Min[(1 - #[[1]]) (1 - #[[2]]), #[[1]] #[[2]]] ≤
       #[[3]] ≤ Min[#[[1]] (1 - #[[2]]), #[[2]] (1 - #[[1]])] &&
      0 < #[[4]] < 1 && 0 < #[[5]] < 1 &], 1], {αα, 100, 1000, 100}]
```

FindRoot: The line search decreased the step size to within tolerance specified by AccuracyGoal and PrecisionGoal but was unable to find a sufficient decrease in the merit function. You may need more than MachinePrecision digits of working precision to meet these tolerances.

FindRoot: Failed to converge to the requested accuracy or precision within 200 iterations.

FindRoot: The line search decreased the step size to within tolerance specified by AccuracyGoal and PrecisionGoal but was unable to find a sufficient decrease in the merit function. You may need more than MachinePrecision digits of working precision to meet these tolerances.

FindRoot: The line search decreased the step size to within tolerance specified by AccuracyGoal and PrecisionGoal but was unable to find a sufficient decrease in the merit function. You may need more than MachinePrecision digits of working precision to meet these tolerances.

General: Further output of FindRoot::lstol will be suppressed during this calculation.

```
{{0.518598, 0.517069, 0.205057, 0.53299, 0.534594, 0.204313},
 {0.520327, 0.519626, 0.217148, 0.529867, 0.530593, 0.216669},
 {0.521087, 0.520645, 0.222734, 0.528517, 0.528971, 0.222365},
 {}, {}, {}, {}, {}, {}, {}}
```

```
params[αα_] := {s1 → 0.2, s2 → 0.2, m1 → 0.01, m2 → 0.01, α1 → 1.1 αα, α2 → αα};
Table[t12t22 = FindRoot[{factdelta3maler1 /. p12p22genmt12 /. dd12genmt12 /. params[αα],
     factdelta6maler1 /. p12p22genmt12 /. dd12genmt12 /. params[αα]} == 0,
    {{t12, 0.52}, {t22, 0.53}}, MaxIterations → 200]; Flatten[
  Select[{{p12, t12, dd1, p22, t22, dd2}} /. p12p22genmt12 /. dd12genmt12 /. t12t22 /.
     params[αα],
    0 < #[[1]] < 1 && 0 < #[[2]] < 1 && -Min[(1 - #[[1]]) (1 - #[[2]]), #[[1]] #[[2]]] ≤
      #[[3]] ≤ Min[#[[1]] (1 - #[[2]]), #[[2]] (1 - #[[1]])] &&
     0 < #[[4]] < 1 && 0 < #[[5]] < 1 &], 1], {αα, 100, 1000, 100}]
{{0.518598, 0.517069, 0.205057, 0.53299, 0.534594, 0.204313},
 {0.520327, 0.519626, 0.217148, 0.529867, 0.530593, 0.216669},
 {0.521087, 0.520645, 0.222734, 0.528517, 0.528971, 0.222365},
 {0.521535, 0.521218, 0.226133, 0.527728, 0.528053, 0.225828},
 {0.521837, 0.521593, 0.228484, 0.527199, 0.527448, 0.228221}, {}, {}, {}, {}, {}}
```

3. Numerics

3.1 Checking convergence by iteration

The following routine is quite useful to check convergence with a limited output

```
testdif[n_][x_, y_] := If[Max[Abs[x - y]] < 10.^(-n), False, True]
```

In general, n = 10 worked very well (see also below)

```
tend1 = 200;
tend2 = 10;
res = Table[{0, 0, 0, 0, 0, 0}, {i, 1, tend2 + 1}];
res[[1]] = {.5, .5, 0, .5, .5, 0};
Table[res[[i + 1]] = NestWhile[Chop[itgenmale[0.2, 0.2, 10, 10, 0.01, 0.01, 0.5]],
    res[[i]], testdif[10], 2, tend1, -1], {i, 1, tend2}] // AbsoluteTiming
{1.20132, {{0.474167, 0.45117, 0.131649, 0.525833, 0.54883, 0.131649},
  {0.473819, 0.450751, 0.131622, 0.526181, 0.549249, 0.131622},
  {0.473813, 0.450744, 0.131621, 0.526187, 0.549256, 0.131621},
  {0.473813, 0.450744, 0.131621, 0.526187, 0.549256, 0.131621},
  {0.473813, 0.450744, 0.131621, 0.526187, 0.549256, 0.131621},
  {0.473813, 0.450744, 0.131621, 0.526187, 0.549256, 0.131621},
  {0.473813, 0.450744, 0.131621, 0.526187, 0.549256, 0.131621},
  {0.473813, 0.450744, 0.131621, 0.526187, 0.549256, 0.131621},
  {0.473813, 0.450744, 0.131621, 0.526187, 0.549256, 0.131621},
  {0.473813, 0.450744, 0.131621, 0.526187, 0.549256, 0.131621}}}
```

Starting with asymmetric initial conditions, if parameters are symmetric, is much slower:

```
tend1 = 5000;
tend2 = 10;
res = Table[{0, 0, 0, 0, 0, 0}, {i, 1, tend2 + 1}];
res[[1]] = {.45, .5, 0, .5, .52, 0};
Table[res[[i + 1]] = NestWhile[Chop[itgenmale[0.2, 0.2, 10, 10, 0.01, 0.01, 0.5]],
    res[[i]], UnsameQ, 2, tend1, -1], {i, 1, tend2}] // AbsoluteTiming
```

```
{75.0188, {{0.453186, 0.425906, 0.129647, 0.505515, 0.524363, 0.132868},
  {0.46466, 0.439722, 0.130835, 0.517025, 0.538224, 0.132265},
  {0.469754, 0.445856, 0.13129, 0.522126, 0.544366, 0.131924},
  {0.472013, 0.448576, 0.131478, 0.524387, 0.547088, 0.131759},
  {0.473015, 0.449783, 0.131558, 0.525389, 0.548295, 0.131683},
  {0.473459, 0.450317, 0.131593, 0.525833, 0.54883, 0.131649},
  {0.473656, 0.450555, 0.131609, 0.52603, 0.549067, 0.131633},
  {0.473744, 0.45066, 0.131616, 0.526117, 0.549173, 0.131627},
  {0.473782, 0.450706, 0.131619, 0.526156, 0.549219, 0.131624},
  {0.473799, 0.450727, 0.13162, 0.526173, 0.54924, 0.131622}}}
```

Convergence with asymmetric parameters is slow:

```
tend1 = 5000;
tend2 = 20;
res = Table[{0, 0, 0, 0, 0, 0}, {i, 1, tend2 + 1}];
res[[1]] = {.5, .5, 0, .5, .5, 0};
Table[res[[i + 1]] = NestWhile[Chop[itgenmale[0.2, 0.2, 10, 11, 0.01, 0.01, 0.5]],
    res[[i]], testdif[10], 2, tend1, -1], {i, 1, tend2}] // AbsoluteTiming
```

```
{109.315, {{0.458604, 0.438428, 0.133012, 0.509654, 0.533088, 0.134002},
  {0.450154, 0.428348, 0.13215, 0.501146, 0.522906, 0.134423},
  {0.446296, 0.423744, 0.131716, 0.497255, 0.518251, 0.134575},
  {0.444533, 0.421641, 0.131509, 0.495477, 0.516123, 0.134635},
  {0.443728, 0.42068, 0.131413, 0.494664, 0.51515, 0.134661},
  {0.44336, 0.420241, 0.131368, 0.494293, 0.514705, 0.134672},
  {0.443191, 0.42004, 0.131348, 0.494123, 0.514502, 0.134678},
  {0.443115, 0.419948, 0.131339, 0.494045, 0.514409, 0.13468},
  {0.443079, 0.419906, 0.131334, 0.49401, 0.514367, 0.134681},
  {0.443063, 0.419887, 0.131332, 0.493994, 0.514348, 0.134681},
  {0.443056, 0.419878, 0.131331, 0.493986, 0.514339, 0.134682},
  {0.443053, 0.419874, 0.131331, 0.493983, 0.514335, 0.134682},
  {0.443051, 0.419873, 0.131331, 0.493981, 0.514333, 0.134682},
  {0.44305, 0.419872, 0.131331, 0.493981, 0.514332, 0.134682},
  {0.44305, 0.419872, 0.131331, 0.49398, 0.514332, 0.134682},
  {0.44305, 0.419872, 0.131331, 0.49398, 0.514332, 0.134682},
  {0.44305, 0.419872, 0.131331, 0.49398, 0.514332, 0.134682},
  {0.44305, 0.419872, 0.131331, 0.49398, 0.514332, 0.134682},
  {0.44305, 0.419872, 0.131331, 0.49398, 0.514332, 0.134682},
  {0.44305, 0.419872, 0.131331, 0.49398, 0.514332, 0.134682}}}
```

The choice of n in testdif[n] influence accuracy and speed :

```
tend1 = 5000;
tend2 = 20;
res = Table[{0, 0, 0, 0, 0, 0}, {i, 1, tend2 + 1}];
res[[1]] = {.5, .5, 0, .5, .5, 0};
Table[res[[i + 1]] = NestWhile[Chop[itgenmale[0.2, 0.2, 10, 11, 0.01, 0.01, 0.5]],
    res[[i]], testdif[8], 2, tend1, -1], {i, 1, tend2}] // AbsoluteTiming
```

```
{60.4561, {{0.458604, 0.438428, 0.133012, 0.509654, 0.533088, 0.134002},
  {0.450154, 0.428348, 0.13215, 0.501146, 0.522906, 0.134423},
  {0.446296, 0.423744, 0.131716, 0.497255, 0.518251, 0.134575},
  {0.444533, 0.421641, 0.131509, 0.495477, 0.516123, 0.134635},
  {0.443728, 0.42068, 0.131413, 0.494664, 0.51515, 0.134661},
  {0.44336, 0.420241, 0.131368, 0.494293, 0.514705, 0.134672},
  {0.443191, 0.42004, 0.131348, 0.494123, 0.514502, 0.134678},
  {0.443115, 0.419948, 0.131339, 0.494045, 0.514409, 0.13468},
  {0.443103, 0.419934, 0.131337, 0.494033, 0.514395, 0.13468},
  {0.443103, 0.419934, 0.131337, 0.494033, 0.514395, 0.13468},
  {0.443103, 0.419934, 0.131337, 0.494033, 0.514395, 0.13468},
  {0.443103, 0.419934, 0.131337, 0.494033, 0.514395, 0.13468},
  {0.443103, 0.419934, 0.131337, 0.494033, 0.514395, 0.13468},
  {0.443103, 0.419934, 0.131337, 0.494033, 0.514395, 0.13468},
  {0.443103, 0.419934, 0.131337, 0.494033, 0.514395, 0.13468},
  {0.443103, 0.419934, 0.131337, 0.494033, 0.514395, 0.13468},
  {0.443103, 0.419934, 0.131337, 0.494033, 0.514395, 0.13468},
  {0.443103, 0.419934, 0.131337, 0.494033, 0.514395, 0.13468},
  {0.443103, 0.419934, 0.131337, 0.494033, 0.514395, 0.13468},
  {0.443103, 0.419934, 0.131337, 0.494033, 0.514395, 0.13468}}}
```

```
tend1 = 5000;
tend2 = 20;
res = Table[{0, 0, 0, 0, 0, 0}, {i, 1, tend2 + 1}];
res[[1]] = {.5, .5, 0, .5, .5, 0};
Table[res[[i + 1]] = NestWhile[Chop[itgenmale[0.2, 0.2, 10, 11, 0.01, 0.01, 0.5]],
    res[[i]], UnsameQ, 2, tend1, -1], {i, 1, tend2}] // AbsoluteTiming
```

```
{157.968, {{0.458604, 0.438428, 0.133012, 0.509654, 0.533088, 0.134002},
  {0.450154, 0.428348, 0.13215, 0.501146, 0.522906, 0.134423},
  {0.446296, 0.423744, 0.131716, 0.497255, 0.518251, 0.134575},
  {0.444533, 0.421641, 0.131509, 0.495477, 0.516123, 0.134635},
  {0.443728, 0.42068, 0.131413, 0.494664, 0.51515, 0.134661},
  {0.44336, 0.420241, 0.131368, 0.494293, 0.514705, 0.134672},
  {0.443191, 0.42004, 0.131348, 0.494123, 0.514502, 0.134678},
  {0.443115, 0.419948, 0.131339, 0.494045, 0.514409, 0.13468},
  {0.443079, 0.419906, 0.131334, 0.49401, 0.514367, 0.134681},
  {0.443063, 0.419887, 0.131332, 0.493994, 0.514348, 0.134681},
  {0.443056, 0.419878, 0.131331, 0.493986, 0.514339, 0.134682},
  {0.443053, 0.419874, 0.131331, 0.493983, 0.514335, 0.134682},
  {0.443051, 0.419873, 0.131331, 0.493981, 0.514333, 0.134682},
  {0.44305, 0.419872, 0.131331, 0.493981, 0.514332, 0.134682},
  {0.44305, 0.419871, 0.131331, 0.49398, 0.514332, 0.134682},
  {0.44305, 0.419871, 0.131331, 0.49398, 0.514331, 0.134682},
  {0.44305, 0.419871, 0.131331, 0.49398, 0.514331, 0.134682},
  {0.44305, 0.419871, 0.131331, 0.49398, 0.514331, 0.134682},
  {0.44305, 0.419871, 0.131331, 0.49398, 0.514331, 0.134682},
  {0.44305, 0.419871, 0.131331, 0.49398, 0.514331, 0.134682}}}
```

With symmetric initial conditions, convergence is fast, even if r is small:

```
tend1 = 500;
tend2 = 10;
res = Table[{0, 0, 0, 0, 0, 0}, {i, 1, tend2 + 1}];
res[[1]] = {.05, .05, .95 * .05, .95, .95, .95 * .05};
Table[res[[i + 1]] = NestWhile[Chop[itgenmale[0.2, 0.2, 10, 10, 0.01, 0.01, 0.01]],
    res[[i]], testdif[10], 2, tend1, -1], {i, 1, tend2}] // AbsoluteTiming
```

```
{5.05205, {{0.23517, 0.178562, 0.115365, 0.76483, 0.821438, 0.115365},
  {0.468958, 0.445133, 0.137371, 0.531042, 0.554867, 0.137371},
  {0.473533, 0.450421, 0.131995, 0.526467, 0.549579, 0.131995},
  {0.473796, 0.450723, 0.131645, 0.526204, 0.549277, 0.131645},
  {0.473812, 0.450742, 0.131623, 0.526188, 0.549258, 0.131623},
  {0.473813, 0.450744, 0.131621, 0.526187, 0.549256, 0.131621},
  {0.473813, 0.450744, 0.131621, 0.526187, 0.549256, 0.131621},
  {0.473813, 0.450744, 0.131621, 0.526187, 0.549256, 0.131621},
  {0.473813, 0.450744, 0.131621, 0.526187, 0.549256, 0.131621},
  {0.473813, 0.450744, 0.131621, 0.526187, 0.549256, 0.131621}}}
```

Extremely slow convergence with high, but not maximum, initial LD and asymmetric starting conditions:

```
tend1 = 20000;
tend2 = 10;
res = Table[{0, 0, 0, 0, 0, 0}, {i, 1, tend2 + 1}];
res[[1]] = {.05, .05, .95 * .05, .95, .95, .95 * .02};
Table[res[[i + 1]] = NestWhile[Chop[itgenmale[0.2, 0.2, 10, 10, 0.01, 0.01, 0.01]],
    res[[i]], testdif[10], 2, tend1, -1], {i, 1, tend2}] // AbsoluteTiming
```

```
{302.676, {{0.0759545, 0.000998444, 0.000427325, 0.0818185, 0.0145912, 0.0062369},
  {0.0940498, 0.0074737, 0.00336732, 0.115874, 0.0550877, 0.0240694},
  {0.256616, 0.189562, 0.078303, 0.303755, 0.281422, 0.104481},
  {0.370025, 0.325842, 0.115609, 0.42128, 0.422958, 0.129174},
  {0.424329, 0.391183, 0.126246, 0.476452, 0.489385, 0.132809},
  {0.450241, 0.422371, 0.12958, 0.502559, 0.520813, 0.132714},
  {0.462591, 0.437236, 0.130768, 0.514952, 0.535732, 0.13226},
  {0.468472, 0.444315, 0.131242, 0.520843, 0.542824, 0.131952},
  {0.471271, 0.447684, 0.131447, 0.523645, 0.546196, 0.131785},
  {0.472604, 0.449288, 0.13154, 0.524977, 0.5478, 0.1317}}}
```

Using FindRoot

3.2 3D Graphs

Here, we show by example how to produce the 3D graphs

3.2.1 Definitions

The following is itgenmale[s, s, α, α, m, m, r][{p12, t12, dd1, p22, t22, dd2}] (but slightly faster)

```
itfastsym[s_, α_, m_, r_][{p12_, t12_, dd1_, p22_, t22_, dd2_}] :=
 {((-1 + m)^2 m p12^2 (1 + s) (t12 - t22) α (2 + α) + dd1 (s + α + s α) (-1 + s (-1 + t12) - t12 α) +
     m^3 p22 (t12 - t22) (s^2 (t12 - t22) (1 + α) + α ((-t12 + t22) α + p22 (2 + α)) +
        s α ((-t12 + t22) α + p22 (2 + α))) + m ((s + α + s α)
```

$$(dd2\,(-1 + s\,(-1 + t12) - t12\,\alpha) + dd1\,(1 + s\,(1 - 2\,t12 + t22) + 2\,t12\,\alpha - t22\,\alpha)) +$$
$$p22\,(2 + (2 + 2\,dd1 + t12 - t22)\,\alpha + (dd1 - t12\,(-2 + t12 + t22))\,\alpha^2 +$$
$$s^2\,(-1 + t12)\,(-2 + t12 + t22)\,(1 + \alpha) - s\,(-4 + t22 - 4\,\alpha - 2\,dd1\,\alpha +$$
$$2\,t22\,\alpha - dd1\,\alpha^2 + t12^2\,\alpha^2 + t12\,(3 + 2\,\alpha + (-2 + t22)\,\alpha^2)))) +$$
$$m^2\,(-p22^2\,(1 + s)\,(t12 - t22)\,\alpha\,(2 + \alpha) + (dd1 - dd2)\,(t12 - t22)\,(s - \alpha)\,(s + \alpha + s\,\alpha) +$$
$$p22\,(-s^2\,(2\,t12^2 - (-3 + t22)\,t22 - t12\,(3 + t22))\,(1 + \alpha) - \alpha\,(dd1\,(2 + \alpha) -$$
$$dd2\,(2 + \alpha) - (t12 - t22)\,(-1 + (-2 + 2\,t12 + t22)\,\alpha)) + s\,(2\,t12^2\,\alpha^2 - t22^2\,\alpha^2 -$$
$$(dd1 - dd2)\,\alpha\,(2 + \alpha) + t22\,(-3 - 2\,\alpha + 2\,\alpha^2) + t12\,(3 + 2\,\alpha - (2 + t22)\,\alpha^2)))) -$$
$$(-1 + m)\,p12\,(2 + (2 - 2\,dd1\,(-1 + m) + 2\,dd2\,m - m\,t12 - 2\,m\,p22\,t12 +$$
$$4\,m^2\,p22\,t12 + m\,t22 + 2\,m\,p22\,t22 - 4\,m^2\,p22\,t22)\,\alpha +$$
$$(dd1 - dd1\,m + dd2\,m + 2\,t12 - 2\,m\,t12 - m\,p22\,t12 + 2\,m^2\,p22\,t12 - 2\,t12^2 + 3\,m\,t12^2 -$$
$$m^2\,t12^2 + 2\,m\,t22 + m\,p22\,t22 - 2\,m^2\,p22\,t22 - 3\,m\,t12\,t22 + 2\,m^2\,t12\,t22 - m^2\,t22^2)\,\alpha^2 +$$
$$s^2\,(2 + (2 - 3\,m + m^2)\,t12^2 - 3\,m\,t22 + m^2\,t22^2 + t12\,(-4 - 2\,m^2\,t22 + 3\,m\,(1 + t22)))$$
$$(1 + \alpha) +$$
$$s\,(4 + 4\,\alpha + 2\,dd1\,\alpha + dd1\,\alpha^2 - (2 - 3\,m + m^2)\,t12^2\,\alpha^2 - m^2\,t22\,\alpha\,(t22\,\alpha + 2\,p22\,(2 + \alpha)) +$$
$$m\,(-(dd1 - dd2)\,\alpha\,(2 + \alpha) + t22\,(-3 + 2\,(-1 + p22)\,\alpha + (2 + p22)\,\alpha^2)) +$$
$$t12\,(2\,(-2 - 2\,\alpha + \alpha^2) - m\,(-3 + 2\,(-1 + p22)\,\alpha + (2 + p22 + 3\,t22)\,\alpha^2) +$$
$$2\,m^2\,\alpha\,(t22\,\alpha + p22\,(2 + \alpha)))))) \Big/$$
$$(2\,(1 + (1 + (-1 + m)\,t12 - m\,t22)\,\alpha + s\,(1 + (-1 + m)\,t12 - m\,t22)\,(1 + \alpha))$$
$$(1 + s\,(1 + (-1 + m)\,t12 - m\,t22) + m\,t22\,\alpha + t12\,(\alpha - m\,\alpha))),$$
$$-(((-1 + m)\,t12 - m\,t22)\,(2 + p12\,\alpha - m\,p12\,\alpha + m\,p22\,\alpha + 2\,t12\,\alpha - 2\,m\,t12\,\alpha -$$
$$p12\,t12\,\alpha + 2\,m\,p12\,t12\,\alpha - m^2\,p12\,t12\,\alpha - m\,p22\,t12\,\alpha + m^2\,p22\,t12\,\alpha + 2\,m\,t22\,\alpha -$$
$$m\,p12\,t22\,\alpha + m^2\,p12\,t22\,\alpha - m^2\,p22\,t22\,\alpha + s^2\,(1 + (-1 + m)\,t12 - m\,t22)^2\,(1 + \alpha) -$$
$$s\,(1 + (-1 + m)\,t12 - m\,t22)\,(-3 + 2\,(-1 + (-1 + m)\,p12 - m\,p22)\,\alpha +$$
$$((-1 + m)\,p12 - t12 - m\,(p22 - t12 + t22))\,\alpha^2) - (1 + (-1 + m)\,t12 - m\,t22)$$
$$\alpha\,(-1 - t12\,\alpha + (-1 + m)\,p12\,(1 + \alpha) - m\,(p22 + p22\,\alpha - t12\,\alpha + t22\,\alpha)))) \Big/$$
$$(2\,(1 + (1 + (-1 + m)\,t12 - m\,t22)\,\alpha + s\,(1 + (-1 + m)\,t12 - m\,t22)\,(1 + \alpha))$$
$$(1 + s\,(1 + (-1 + m)\,t12 - m\,t22) + m\,t22\,\alpha + t12\,(\alpha - m\,\alpha))),$$
$$\frac{1}{4}\,(-(((dd1\,(-1 + m) - dd2\,m - p12\,t12 + m\,p12\,t12 - m\,p22\,t22)\,(dd1\,(-1 + m) -$$
$$dd2\,m + t12 - m\,t12 - p12\,t12 + m\,p12\,t12 + m\,t22 - m\,p22\,t22)\,(1 + \alpha)) /$$
$$(-1 + s\,(-1 + t12 - m\,t12 + m\,t22) + (-1 + m)\,t12\,\alpha - m\,t22\,\alpha) +$$
$$((-1 + r)\,(dd1\,(-1 + m) - dd2\,m + p12 - m\,p12 + m\,p22 - p12\,t12 + m\,p12\,t12 - m\,p22\,t22)$$
$$(dd1\,(-1 + m) - dd2\,m + t12 - m\,t12 - p12\,t12 + m\,p12\,t12 + m\,t22 - m\,p22\,t22)$$
$$(1 + \alpha)) / (-1 + s\,(-1 + t12 - m\,t12 + m\,t22) + (-1 + m)\,t12\,\alpha - m\,t22\,\alpha) -$$
$$((dd1\,(-1 + m) - dd2\,m - p12\,t12 + m\,p12\,t12 - m\,p22\,t22)$$
$$(dd1\,(-1 + m) - dd2\,m + t12 - m\,t12 - p12\,t12 + m\,p12\,t12 + m\,t22 - m\,p22\,t22)) /$$
$$(1 + (1 + (-1 + m)\,t12 - m\,t22)\,\alpha + s\,(1 + (-1 + m)\,t12 - m\,t22)\,(1 + \alpha)) +$$
$$(r\,(dd1\,(-1 + m) - dd2\,m - p12\,t12 + m\,p12\,t12 - m\,p22\,t22)\,(-1 + dd1\,(-1 + m) - dd2\,m +$$
$$p12 - m\,p12 + m\,p22 + t12 - m\,t12 - p12\,t12 + m\,p12\,t12 + m\,t22 - m\,p22\,t22)) /$$
$$(1 + (1 + (-1 + m)\,t12 - m\,t22)\,\alpha + s\,(1 + (-1 + m)\,t12 - m\,t22)\,(1 + \alpha)) -$$
$$((dd1\,(-1 + m) - dd2\,m + t12 - m\,t12 - p12\,t12 + m\,p12\,t12 + m\,t22 - m\,p22\,t22)$$
$$(-1 + dd1\,(-1 + m) - dd2\,m + p12 - m\,p12 + m\,p22 + t12 -$$
$$m\,t12 - p12\,t12 + m\,p12\,t12 + m\,t22 - m\,p22\,t22)) /$$
$$(1 + (1 + (-1 + m)\,t12 - m\,t22)\,\alpha + s\,(1 + (-1 + m)\,t12 - m\,t22)\,(1 + \alpha)) +$$
$$(2\,(dd1 - dd1\,m + dd2\,m - t12 + m\,t12 + p12\,t12 - m\,p12\,t12 - m\,t22 + m\,p22\,t22)^2) \Big/$$
$$(1 + (1 + (-1 + m)\,t12 - m\,t22)\,\alpha + s\,(1 + (-1 + m)\,t12 - m\,t22)\,(1 + \alpha)) -$$
$$((-1 + r)\,(1 + s)\,(dd1\,(-1 + m) - dd2\,m + p12 - m\,p12 + m\,p22 - p12\,t12 +$$
$$m\,p12\,t12 - m\,p22\,t22)\,(dd1\,(-1 + m) - dd2\,m + t12 -$$

```
            m t12 - p12 t12 + m p12 t12 + m t22 - m p22 t22) (1 + α)) /
        (1 + (1 + (-1 + m) t12 - m t22) α + s (1 + (-1 + m) t12 - m t22) (1 + α)) -
       ((1 + s) (dd1 (-1 + m) - dd2 m + t12 - m t12 - p12 t12 + m p12 t12 + m t22 - m p22 t22)
          (-1 + dd1 (-1 + m) - dd2 m + p12 - m p12 + m p22 + t12 -
            m t12 - p12 t12 + m p12 t12 + m t22 - m p22 t22) (1 + α)) /
        (1 + (1 + (-1 + m) t12 - m t22) α + s (1 + (-1 + m) t12 - m t22) (1 + α)) +
       (r (1 + s) (dd1 (-1 + m) - dd2 m - p12 t12 + m p12 t12 - m p22 t22) (-1 + dd1 (-1 + m) -
            dd2 m + p12 - m p12 + m p22 + t12 - m t12 - p12 t12 + m p12 t12 + m t22 -
            m p22 t22)) / (1 + s (1 + (-1 + m) t12 - m t22) + m t22 α + t12 (α - m α)))
  (((dd1 (-1 + m) - dd2 m - p12 t12 + m p12 t12 - m p22 t22) (dd1 (-1 + m) -
           dd2 m + p12 - m p12 + m p22 - p12 t12 + m p12 t12 - m p22 t22) (1 + α)) /
      (-1 + s (-1 + t12 - m t12 + m t22) + (-1 + m) t12 α - m t22 α) +
     ((-1 + r) (dd1 (-1 + m) - dd2 m + p12 - m p12 + m p22 - p12 t12 + m p12 t12 - m p22 t22)
         (dd1 (-1 + m) - dd2 m + t12 - m t12 - p12 t12 + m p12 t12 + m t22 - m p22 t22)
         (1 + α)) / (-1 + s (-1 + t12 - m t12 + m t22) + (-1 + m) t12 α - m t22 α) +
     (r (dd1 (-1 + m) - dd2 m - p12 t12 + m p12 t12 - m p22 t22) (-1 + dd1 (-1 + m) - dd2 m +
           p12 - m p12 + m p22 + t12 - m t12 - p12 t12 + m p12 t12 + m t22 - m p22 t22)) /
      (1 + (1 + (-1 + m) t12 - m t22) α + s (1 + (-1 + m) t12 - m t22) (1 + α)) - ((-1 + r)
         (1 + s) (dd1 (-1 + m) - dd2 m + p12 - m p12 + m p22 - p12 t12 + m p12 t12 - m p22 t22)
         (dd1 (-1 + m) - dd2 m + t12 - m t12 - p12 t12 + m p12 t12 + m t22 - m p22 t22)
         (1 + α)) / (1 + (1 + (-1 + m) t12 - m t22) α + s (1 + (-1 + m) t12 - m t22) (1 + α)) -
     ((1 + s) (dd1 (-1 + m) - dd2 m + p12 - m p12 + m p22 - p12 t12 + m p12 t12 - m p22 t22)
         (-1 + dd1 (-1 + m) - dd2 m + p12 - m p12 + m p22 + t12 -
           m t12 - p12 t12 + m p12 t12 + m t22 - m p22 t22) (1 + α)) /
      (1 + (1 + (-1 + m) t12 - m t22) α + s (1 + (-1 + m) t12 - m t22) (1 + α)) -
     ((1 + s) (dd1 (-1 + m) - dd2 m - p12 t12 + m p12 t12 - m p22 t22)
         (dd1 (-1 + m) - dd2 m + p12 - m p12 + m p22 - p12 t12 + m p12 t12 - m p22 t22)) /
      (1 + s (1 + (-1 + m) t12 - m t22) + m t22 α + t12 (α - m α)) +
     (r (1 + s) (dd1 (-1 + m) - dd2 m - p12 t12 + m p12 t12 - m p22 t22)
         (-1 + dd1 (-1 + m) - dd2 m + p12 - m p12 + m p22 + t12 - m t12 - p12 t12 + m p12 t12 +
           m t22 - m p22 t22)) / (1 + s (1 + (-1 + m) t12 - m t22) + m t22 α + t12 (α - m α)) -
     ((1 + s) (dd1 (-1 + m) - dd2 m + p12 - m p12 + m p22 - p12 t12 + m p12 t12 - m p22 t22)
         (-1 + dd1 (-1 + m) - dd2 m + p12 - m p12 + m p22 + t12 -
           m t12 - p12 t12 + m p12 t12 + m t22 - m p22 t22)) /
      (1 + s (1 + (-1 + m) t12 - m t22) + m t22 α + t12 (α - m α)) + (2 (1 + s)
         (dd1 - dd1 m + dd2 m - p12 + m p12 - m p22 + p12 t12 - m p12 t12 + m p22 t22)^2) /
      (1 + s (1 + (-1 + m) t12 - m t22) + m t22 α + t12 (α - m α))) +
  (- ((-1 + r) (dd1 (-1 + m) - dd2 m - p12 t12 + m p12 t12 - m p22 t22)
          (-1 + dd1 (-1 + m) - dd2 m + p12 - m p12 + m p22 + t12 -
            m t12 - p12 t12 + m p12 t12 + m t22 - m p22 t22)) /
      (1 + (1 + (-1 + m) t12 - m t22) α + s (1 + (-1 + m) t12 - m t22) (1 + α)) -
     ((dd1 (-1 + m) - dd2 m + t12 - m t12 - p12 t12 + m p12 t12 + m t22 - m p22 t22)
         (-1 + dd1 (-1 + m) - dd2 m + p12 - m p12 + m p22 + t12 -
           m t12 - p12 t12 + m p12 t12 + m t22 - m p22 t22)) /
      (1 + (1 + (-1 + m) t12 - m t22) α + s (1 + (-1 + m) t12 - m t22) (1 + α)) +
     (r (1 + s) (dd1 (-1 + m) - dd2 m + p12 - m p12 + m p22 - p12 t12 + m p12 t12 - m p22 t22)
         (dd1 (-1 + m) - dd2 m + t12 - m t12 - p12 t12 + m p12 t12 + m t22 - m p22 t22)
         (1 + α)) / (1 + (1 + (-1 + m) t12 - m t22) α + s (1 + (-1 + m) t12 - m t22) (1 + α)) -
     ((1 + s) (dd1 (-1 + m) - dd2 m + p12 - m p12 + m p22 - p12 t12 + m p12 t12 - m p22 t22)
```

```
(-1 + dd1 (-1 + m) - dd2 m + p12 - m p12 + m p22 + t12 -
    m t12 - p12 t12 + m p12 t12 + m t22 - m p22 t22) (1 + α)) /
  (1 + (1 + (-1 + m) t12 - m t22) α + s (1 + (-1 + m) t12 - m t22) (1 + α)) -
 ((1 + s) (dd1 (-1 + m) - dd2 m + t12 - m t12 - p12 t12 + m p12 t12 + m t22 - m p22 t22)
    (-1 + dd1 (-1 + m) - dd2 m + p12 - m p12 + m p22 + t12 -
      m t12 - p12 t12 + m p12 t12 + m t22 - m p22 t22) (1 + α)) /
  (1 + (1 + (-1 + m) t12 - m t22) α + s (1 + (-1 + m) t12 - m t22) (1 + α)) +
 (2 (1 + s) (-1 + dd1 (-1 + m) - dd2 m + p12 - m p12 + m p22 + t12 - m
        t12 - p12 t12 + m p12 t12 + m t22 - m p22 t22)^2 (1 + α)) /
  (1 + (1 + (-1 + m) t12 - m t22) α + s (1 + (-1 + m) t12 - m t22) (1 + α)) -
 ((-1 + r) (1 + s) (dd1 (-1 + m) - dd2 m - p12 t12 + m p12 t12 - m p22 t22)
    (-1 + dd1 (-1 + m) - dd2 m + p12 - m p12 + m p22 + t12 - m t12 - p12 t12 + m p12 t12 +
      m t22 - m p22 t22)) / (1 + s (1 + (-1 + m) t12 - m t22) + m t22 α + t12 (α - m α)) -
 ((1 + s) (dd1 (-1 + m) - dd2 m + p12 - m p12 + m p22 - p12 t12 + m p12 t12 - m p22 t22)
    (-1 + dd1 (-1 + m) - dd2 m + p12 - m p12 + m p22 + t12 - m t12 - p12 t12 + m p12 t12 +
      m t22 - m p22 t22)) / (1 + s (1 + (-1 + m) t12 - m t22) + m t22 α + t12 (α - m α)) +
 (r (dd1 (-1 + m) - dd2 m + p12 - m p12 + m p22 - p12 t12 + m p12 t12 - m p22 t22)
    (dd1 (-1 + m) - dd2 m + t12 - m t12 - p12 t12 + m p12 t12 + m t22 - m p22 t22)
    (1 + α)) / (1 + s (1 + (-1 + m) t12 - m t22) + m t22 α + t12 (α - m α)))
(((dd1 (-1 + m) - dd2 m - p12 t12 + m p12 t12 - m p22 t22) (dd1 (-1 + m) -
      dd2 m + p12 - m p12 + m p22 - p12 t12 + m p12 t12 - m p22 t22) (1 + α)) /
  (-1 + s (-1 + t12 - m t12 + m t22) + (-1 + m) t12 α - m t22 α) +
 ((dd1 (-1 + m) - dd2 m - p12 t12 + m p12 t12 - m p22 t22) (dd1 (-1 + m) -
      dd2 m + t12 - m t12 - p12 t12 + m p12 t12 + m t22 - m p22 t22) (1 + α)) /
  (-1 + s (-1 + t12 - m t12 + m t22) + (-1 + m) t12 α - m t22 α) -
 ((dd1 (-1 + m) - dd2 m - p12 t12 + m p12 t12 - m p22 t22)
    (dd1 (-1 + m) - dd2 m + t12 - m t12 - p12 t12 + m p12 t12 + m t22 - m p22 t22)) /
  (1 + (1 + (-1 + m) t12 - m t22) α + s (1 + (-1 + m) t12 - m t22) (1 + α)) -
 ((-1 + r) (dd1 (-1 + m) - dd2 m - p12 t12 + m p12 t12 - m p22 t22)
    (-1 + dd1 (-1 + m) - dd2 m + p12 - m p12 + m p22 + t12 -
      m t12 - p12 t12 + m p12 t12 + m t22 - m p22 t22)) /
  (1 + (1 + (-1 + m) t12 - m t22) α + s (1 + (-1 + m) t12 - m t22) (1 + α)) +
 (r (1 + s) (dd1 (-1 + m) - dd2 m + p12 - m p12 + m p22 - p12 t12 + m p12 t12 - m p22 t22)
    (dd1 (-1 + m) - dd2 m + t12 - m t12 - p12 t12 + m p12 t12 + m t22 - m p22 t22)
    (1 + α)) / (1 + (1 + (-1 + m) t12 - m t22) α + s (1 + (-1 + m) t12 - m t22) (1 + α)) -
 ((1 + s) (dd1 (-1 + m) - dd2 m - p12 t12 + m p12 t12 - m p22 t22)
    (dd1 (-1 + m) - dd2 m + p12 - m p12 + m p22 - p12 t12 + m p12 t12 - m p22 t22)) /
  (1 + s (1 + (-1 + m) t12 - m t22) + m t22 α + t12 (α - m α)) -
 ((-1 + r) (1 + s) (dd1 (-1 + m) - dd2 m - p12 t12 + m p12 t12 - m p22 t22)
    (-1 + dd1 (-1 + m) - dd2 m + p12 - m p12 + m p22 + t12 - m t12 - p12 t12 + m p12 t12 +
      m t22 - m p22 t22)) / (1 + s (1 + (-1 + m) t12 - m t22) + m t22 α + t12 (α - m α)) +
 (r (dd1 (-1 + m) - dd2 m + p12 - m p12 + m p22 - p12 t12 + m p12 t12 - m p22 t22)
    (dd1 (-1 + m) - dd2 m + t12 - m t12 - p12 t12 + m p12 t12 + m t22 - m p22 t22)
    (1 + α)) / (1 + s (1 + (-1 + m) t12 - m t22) + m t22 α + t12 (α - m α)) +
 (2 (dd1 - dd1 m + dd2 m + p12 t12 - m p12 t12 + m p22 t22)^2 (1 + α)) /
  (1 + s (1 + (-1 + m) t12 - m t22) + m t22 α + t12 (α - m α)))),
(dd2 (s - α) (1 + t22 α + s t22 (1 + α)) - m^3 (p12 - p22) (t12 - t22)
  (-s^2 (t12 - t22) (1 + α) + α ((t12 - t22) α + p12 (2 + α) - p22 (2 + α)) +
```

```
      s α ((t12 - t22) α + p12 (2 + α) - p22 (2 + α))) +
   p22 (2 + 2 (1 + dd2) α + (dd2 - 2 (-1 + t22) t22) α^2 + 2 s^2 t22^2 (1 + α) +
      s (-2 t22^2 α^2 + dd2 α (2 + α) + 2 t22 (2 + 2 α + α^2))) +
   m^2 (p12^2 (1 + s) (t12 - t22) α (2 + α) + 2 p22^2 (1 + s) (t12 - t22) α (2 + α) +
      (dd1 - dd2) (t12 - t22) (s - α) (s + α + s α) +
      p22 (s^2 (t12^2 - 5 t12 t22 + 4 t22^2) (1 + α) - α (dd1 (2 + α) - dd2 (2 + α) +
            (t12 - t22) (1 + (2 + t12 - 4 t22) α)) - s (t12^2 α^2 + 4 t22^2 α^2 +
            (dd1 - dd2) α (2 + α) - t22 (3 + 4 α + 2 α^2) + t12 (3 + 4 α + (2 - 5 t22) α^2))) +
      p12 (s^2 (t12^2 + t12 t22 - 2 t22^2) (1 + α) - s (t12^2 α^2 - 2 t22^2 α^2 -
            (dd1 - dd2) α (2 + α) + t22 (3 + (4 - 6 p22) α + (2 - 3 p22) α^2) +
            t12 (-3 + (-4 + 6 p22) α + (-2 + 3 p22 + t22) α^2)) - α (-dd1 (2 + α) +
            dd2 (2 + α) + (t12 - t22) (-1 + (-2 + t12 + 2 t22) α + 3 p22 (2 + α))))) +
   m (-p22^2 (1 + s) (t12 - t22) α (2 + α) + (s - α) (dd2 (-1 + t12 α - 2 t22 α +
            s (t12 - 2 t22) (1 + α)) + dd1 (1 + t22 α + s t22 (1 + α))) +
      p22 (-2 + (-2 + 2 dd1 - 4 dd2 + t12 - t22) α + (dd1 - 2 dd2 + 2 t12 - 4 t22 -
            3 t12 t22 + 5 t22^2) α^2 + s^2 (3 t12 - 5 t22) t22 (1 + α) + s (5 t22^2 α^2 +
            (dd1 - 2 dd2) α (2 + α) - t22 (7 + 8 α + 4 α^2) + t12 (3 + 4 α + (2 - 3 t22) α^2))) +
      p12 (2 + (2 + 2 dd2 + (-1 + 2 p22) t12 + t22 - 2 p22 t22) α +
         (dd2 + p22 (t12 - t22) - t22 (-2 + t12 + t22)) α^2 +
         s^2 t22 (t12 + t22) (1 + α) + s (-t22^2 α^2 + dd2 α (2 + α) +
            t22 (3 - 2 (-2 + p22) α - (-2 + p22) α^2) + t12 (1 - t22 α^2 + p22 α (2 + α)))))) /
  (2 (1 + s t22 + m (t12 - t22) (s - α) + α - t22 α) (1 + t22 α + s t22 (1 + α) +
      m (t12 - t22) (s + α + s α))),
 ((m (t12 - t22) + t22) (2 + (1 + 2 p22 - 2 m^2 (p12 - p22) (t12 - t22) +
         m (t12 - 2 p22 (1 + t12 - 2 t22) - 2 p12 (-1 + t22) - t22) + t22 - 2 p22 t22) α -
      (-1 + m (t12 - t22) + t22) (p22 + m (p12 - p22 + t12 - t22) + t22) α^2 +
      s^2 (m^2 (t12 - t22)^2 + t22 (1 + t22) + m (t12 - t22) (1 + 2 t22)) (1 + α) -
      s (-1 - 3 t22 - 2 p22 α - 4 t22 α + 2 p22 t22 α - p22 α^2 - t22 α^2 + p22 t22 α^2 +
         t22^2 α^2 + m^2 (t12 - t22) α ((t12 - t22) α + p12 (2 + α) - p22 (2 + α)) +
         m (-2 t22^2 α^2 - (p12 - p22) α (2 + α) + t22 (3 + 2 (2 + p12 - 2 p22) α + (1 + p12 -
                  2 p22) α^2) + t12 (-3 + 2 (-2 + p22) α + (-1 + p22 + 2 t22) α^2))))) /
  (2 (1 + s t22 + m (t12 - t22) (s - α) + α - t22 α) (1 + t22 α + s t22 (1 + α) +
      m (t12 - t22) (s + α + s α))),
 1/4 (-(((r (1 + s) (dd2 + dd1 m - dd2 m + m p12 t12 + p22 t22 - m p22 t22)
            (1 + dd2 + dd1 m - dd2 m - m p12 - p22 + m p22 - m t12 + m p12 t12 - t22 + m t22 +
               p22 t22 - m p22 t22)) / (1 + s t22 + m (t12 - t22) (s - α) + α - t22 α) -
         ((-1 + r) (dd2 (-1 + m) - dd1 m + m p12 + p22 - m p22 - m p12 t12 - p22 t22 + m p22 t22)
            (dd2 (-1 + m) - dd1 m + m t12 - m p12 t12 + t22 - m t22 - p22 t22 + m p22 t22)
            (1 + α)) / (1 + s t22 + m (t12 - t22) (s - α) + α - t22 α) -
         ((dd2 (-1 + m) - dd1 m + m p12 + p22 - m p22 - m p12 t12 - p22 t22 + m p22 t22)
            (-1 + dd2 (-1 + m) - dd1 m + m p12 + p22 - m p22 + m t12 - m p12 t12 + t22 - m t22 -
               p22 t22 + m p22 t22) (1 + α)) / (1 + s t22 + m (t12 - t22) (s - α) + α - t22 α) +
         (2 (dd2 + dd1 m - dd2 m - m p12 - p22 + m p22 + m p12 t12 + p22 t22 - m p22 t22)^2) /
          (1 + t22 α + s t22 (1 + α) + m (t12 - t22) (s + α + s α)) -
         ((dd2 (-1 + m) - dd1 m - m p12 t12 - p22 t22 + m p22 t22)
            (dd2 (-1 + m) - dd1 m + m p12 + p22 - m p22 - m p12 t12 - p22 t22 + m p22 t22)) /
          (1 + t22 α + s t22 (1 + α) + m (t12 - t22) (s + α + s α)) +
         (r (dd2 (-1 + m) - dd1 m - m p12 t12 - p22 t22 + m p22 t22) (-1 + dd2 (-1 + m) - dd1 m +
```

```
            m p12 + p22 - m p22 + m t12 - m p12 t12 + t22 - m t22 - p22 t22 + m p22 t22)) /
        (1 + t22 α + s t22 (1 + α) + m (t12 - t22) (s + α + s α)) -
      ((dd2 (-1 + m) - dd1 m + m p12 + p22 - m p22 - m p12 t12 - p22 t22 + m p22 t22)
          (-1 + dd2 (-1 + m) - dd1 m + m p12 + p22 - m p22 + m t12 - m p12 t12 + t22 - m t22 -
            p22 t22 + m p22 t22)) / (1 + t22 α + s t22 (1 + α) + m (t12 - t22) (s + α + s α)) -
      ((1 + s) (dd2 + dd1 m - dd2 m + m p12 t12 + p22 t22 - m p22 t22) (dd2 + dd1 m -
            dd2 m - m p12 - p22 + m p22 + m p12 t12 + p22 t22 - m p22 t22) (1 + α)) /
        (1 + t22 α + s t22 (1 + α) + m (t12 - t22) (s + α + s α)) - ((-1 + r) (1 + s)
          (dd2 + dd1 m - dd2 m - m p12 - p22 + m p22 + m p12 t12 + p22 t22 - m p22 t22)
          (dd2 + dd1 m - dd2 m - m t12 + m p12 t12 - t22 + m t22 + p22 t22 - m p22 t22)
          (1 + α)) / (1 + t22 α + s t22 (1 + α) + m (t12 - t22) (s + α + s α)))
  (-((1 + s) (dd2 + dd1 m - dd2 m + m p12 t12 + p22 t22 - m p22 t22)
          (dd2 + dd1 m - dd2 m - m t12 + m p12 t12 - t22 + m t22 + p22 t22 - m p22 t22)) /
      (1 + s t22 + m (t12 - t22) (s - α) + α - t22 α) + (2 (1 + s)
         (dd2 + dd1 m - dd2 m - m t12 + m p12 t12 - t22 + m t22 + p22 t22 - m p22 t22)^2) /
      (1 + s t22 + m (t12 - t22) (s - α) + α - t22 α) +
     (r (1 + s) (dd2 + dd1 m - dd2 m + m p12 t12 + p22 t22 - m p22 t22)
         (1 + dd2 + dd1 m - dd2 m - m p12 - p22 + m p22 - m t12 + m p12 t12 - t22 + m t22 +
           p22 t22 - m p22 t22)) / (1 + s t22 + m (t12 - t22) (s - α) + α - t22 α) -
     ((1 + s) (dd2 (-1 + m) - dd1 m + m t12 - m p12 t12 + t22 - m t22 - p22 t22 + m p22 t22)
         (-1 + dd2 (-1 + m) - dd1 m + m p12 + p22 - m p22 + m t12 - m p12 t12 + t22 - m t22 -
           p22 t22 + m p22 t22)) / (1 + s t22 + m (t12 - t22) (s - α) + α - t22 α) -
     ((-1 + r) (dd2 (-1 + m) - dd1 m + m p12 + p22 - m p22 - m p12 t12 - p22 t22 + m p22 t22)
         (dd2 (-1 + m) - dd1 m + m t12 - m p12 t12 + t22 - m t22 - p22 t22 + m p22 t22)
         (1 + α)) / (1 + s t22 + m (t12 - t22) (s - α) + α - t22 α) -
     ((dd2 (-1 + m) - dd1 m + m t12 - m p12 t12 + t22 - m t22 - p22 t22 + m p22 t22)
         (-1 + dd2 (-1 + m) - dd1 m + m p12 + p22 - m p22 + m t12 - m p12 t12 + t22 - m t22 -
           p22 t22 + m p22 t22) (1 + α)) / (1 + s t22 + m (t12 - t22) (s - α) + α - t22 α) +
     (r (dd2 (-1 + m) - dd1 m - m p12 t12 - p22 t22 + m p22 t22) (-1 + dd2 (-1 + m) - dd1 m +
           m p12 + p22 - m p22 + m t12 - m p12 t12 + t22 - m t22 - p22 t22 + m p22 t22)) /
      (1 + t22 α + s t22 (1 + α) + m (t12 - t22) (s + α + s α)) -
     ((1 + s) (dd2 + dd1 m - dd2 m + m p12 t12 + p22 t22 - m p22 t22)
         (dd2 + dd1 m - dd2 m - m t12 + m p12 t12 - t22 + m t22 + p22 t22 - m p22 t22) (1 + α)) /
      (1 + t22 α + s t22 (1 + α) + m (t12 - t22) (s + α + s α)) - ((-1 + r) (1 + s)
         (dd2 + dd1 m - dd2 m - m p12 - p22 + m p22 + m p12 t12 + p22 t22 - m p22 t22)
         (dd2 + dd1 m - dd2 m - m t12 + m p12 t12 - t22 + m t22 + p22 t22 - m p22 t22) (1 + α)) /
      (1 + t22 α + s t22 (1 + α) + m (t12 - t22) (s + α + s α))) +
 (-((-1 + r) (1 + s) (dd2 + dd1 m - dd2 m + m p12 t12 + p22 t22 - m p22 t22)
          (1 + dd2 + dd1 m - dd2 m - m p12 - p22 + m p22 - m t12 + m p12 t12 - t22 + m t22 +
            p22 t22 - m p22 t22)) / (1 + s t22 + m (t12 - t22) (s - α) + α - t22 α) -
     ((1 + s) (dd2 (-1 + m) - dd1 m + m t12 - m p12 t12 + t22 - m t22 - p22 t22 + m p22 t22)
         (-1 + dd2 (-1 + m) - dd1 m + m p12 + p22 - m p22 + m t12 - m p12 t12 + t22 - m t22 -
           p22 t22 + m p22 t22)) / (1 + s t22 + m (t12 - t22) (s - α) + α - t22 α) +
     (r (dd2 (-1 + m) - dd1 m + m p12 + p22 - m p22 - m p12 t12 - p22 t22 + m p22 t22)
         (dd2 (-1 + m) - dd1 m + m t12 - m p12 t12 + t22 - m t22 - p22 t22 + m p22 t22)
         (1 + α)) / (1 + s t22 + m (t12 - t22) (s - α) + α - t22 α) -
     ((dd2 (-1 + m) - dd1 m + m p12 + p22 - m p22 - m p12 t12 - p22 t22 + m p22 t22)
         (-1 + dd2 (-1 + m) - dd1 m + m p12 + p22 - m p22 + m t12 - m p12 t12 + t22 - m t22 -
           p22 t22 + m p22 t22) (1 + α)) / (1 + s t22 + m (t12 - t22) (s - α) + α - t22 α) -
```

```
((dd2 (-1 + m) - dd1 m + m t12 - m p12 t12 + t22 - m t22 - p22 t22 + m p22 t22)
    (-1 + dd2 (-1 + m) - dd1 m + m p12 + p22 - m p22 + m t12 - m p12 t12 + t22 - m t22 -
      p22 t22 + m p22 t22) (1 + α)) / (1 + s t22 + m (t12 - t22) (s - α) + α - t22 α) +
(2 (-1 + dd2 (-1 + m) - dd1 m + m p12 + p22 - m p22 + m t12 - m p12 t12 + t22 - m t22 - p22
        t22 + m p22 t22)^2 (1 + α)) / (1 + s t22 + m (t12 - t22) (s - α) + α - t22 α) -
((-1 + r) (dd2 (-1 + m) - dd1 m - m p12 t12 - p22 t22 + m p22 t22)
    (-1 + dd2 (-1 + m) - dd1 m + m p12 + p22 - m p22 + m t12 - m p12 t12 + t22 - m t22 -
      p22 t22 + m p22 t22)) / (1 + t22 α + s t22 (1 + α) + m (t12 - t22) (s + α + s α)) -
((dd2 (-1 + m) - dd1 m + m p12 + p22 - m p22 - m p12 t12 - p22 t22 + m p22 t22)
    (-1 + dd2 (-1 + m) - dd1 m + m p12 + p22 - m p22 + m t12 - m p12 t12 + t22 - m t22 -
      p22 t22 + m p22 t22)) / (1 + t22 α + s t22 (1 + α) + m (t12 - t22) (s + α + s α)) +
(r (1 + s) (dd2 + dd1 m - dd2 m - m p12 - p22 + m p22 + m p12 t12 + p22 t22 - m p22 t22)
    (dd2 + dd1 m - dd2 m - m t12 + m p12 t12 - t22 + m t22 + p22 t22 - m p22 t22) (1 + α)) /
  (1 + t22 α + s t22 (1 + α) + m (t12 - t22) (s + α + s α)))
(- ((1 + s) (dd2 + dd1 m - dd2 m + m p12 t12 + p22 t22 - m p22 t22)
      (dd2 + dd1 m - dd2 m - m t12 + m p12 t12 - t22 + m t22 + p22 t22 - m p22 t22)) /
  (1 + s t22 + m (t12 - t22) (s - α) + α - t22 α) -
((-1 + r) (1 + s) (dd2 + dd1 m - dd2 m + m p12 t12 + p22 t22 - m p22 t22)
    (1 + dd2 + dd1 m - dd2 m - m p12 - p22 + m p22 - m t12 + m p12 t12 - t22 + m t22 +
      p22 t22 - m p22 t22)) / (1 + s t22 + m (t12 - t22) (s - α) + α - t22 α) +
(r (dd2 (-1 + m) - dd1 m + m p12 + p22 - m p22 - m p12 t12 - p22 t22 + m p22 t22)
    (dd2 (-1 + m) - dd1 m + m t12 - m p12 t12 + t22 - m t22 - p22 t22 + m p22 t22)
    (1 + α)) / (1 + s t22 + m (t12 - t22) (s - α) + α - t22 α) -
((dd2 (-1 + m) - dd1 m - m p12 t12 - p22 t22 + m p22 t22)
    (dd2 (-1 + m) - dd1 m + m p12 + p22 - m p22 - m p12 t12 - p22 t22 + m p22 t22)) /
  (1 + t22 α + s t22 (1 + α) + m (t12 - t22) (s + α + s α)) -
((-1 + r) (dd2 (-1 + m) - dd1 m - m p12 t12 - p22 t22 + m p22 t22)
    (-1 + dd2 (-1 + m) - dd1 m + m p12 + p22 - m p22 + m t12 - m p12 t12 + t22 - m t22 -
      p22 t22 + m p22 t22)) / (1 + t22 α + s t22 (1 + α) + m (t12 - t22) (s + α + s α)) +
(2 (1 + s) (dd2 + dd1 m - dd2 m + m p12 t12 + p22 t22 - m p22 t22)^2 (1 + α)) /
  (1 + t22 α + s t22 (1 + α) + m (t12 - t22) (s + α + s α)) -
((1 + s) (dd2 + dd1 m - dd2 m + m p12 t12 + p22 t22 - m p22 t22)
    (dd2 + dd1 m - dd2 m - m p12 - p22 + m p22 + m p12 t12 + p22 t22 - m p22 t22) (1 + α)) /
  (1 + t22 α + s t22 (1 + α) + m (t12 - t22) (s + α + s α)) -
((1 + s) (dd2 + dd1 m - dd2 m + m p12 t12 + p22 t22 - m p22 t22)
    (dd2 + dd1 m - dd2 m - m t12 + m p12 t12 - t22 + m t22 + p22 t22 - m p22 t22) (1 + α)) /
  (1 + t22 α + s t22 (1 + α) + m (t12 - t22) (s + α + s α)) +
(r (1 + s) (dd2 + dd1 m - dd2 m - m p12 - p22 + m p22 + m p12 t12 + p22 t22 - m p22 t22)
    (dd2 + dd1 m - dd2 m - m t12 + m p12 t12 - t22 + m t22 + p22 t22 - m p22 t22) (1 + α)) /
  (1 + t22 α + s t22 (1 + α) + m (t12 - t22) (s + α + s α)))}
```

```
mdatsymfix1[p20_, t20_, dd0_, s_, α_, m_, r_, tend_] :=
 mdatsymfix1[p20, t20, dd0, s, α, m, r, tend] = PadRight[NestWhileList[
    itfastsym[s, α, m, r], {0, 0, 0, p2, t2, dd} /. {p2 → p20, t2 → t20, dd → dd0},
    Chop[#[[5]]] > 10^(-14) &, 1, tend], tend + 1, {{0, 0, 0, 0, 0, 0}}]
```

```
initvalD0 = {{0.99, 0.99, 0}, {0.95, 0.95, 0},
   {0.9, 0.9, 0}, {0.8, 0.8, 0}, {0.7, 0.7, 0}, {0.6, 0.6, 0}};
```

```
mtabdatfixD0[ss_, αα_, mm_, r_, tend_][nvals_] := Table[
  {n, Transpose[Table[mdatsymfix1[initvalD0[[i, 1]], initvalD0[[i, 2]], initvalD0[[i,
        3]], ss, αα, mm, r, tend][[n, 4 ;; 6]] // Chop, {i, 1, 6}]]}, {n, nvals}]
```

```
initphm = {{0.99, 0.99, 0.99 * 0.01}, {0.95, 0.95, 0.05 * 0.95}, {0.9, 0.9, 0.1 * 0.9},
   {0.8, 0.8, 0.2 * 0.8}, {0.7, 0.7, 0.3 * 0.7}, {0.6, 0.6, 0.4 * 0.6}};
```

```
mtabdatphmfix1[ss_, αα_, mm_, r_, tend_][nvals_] :=
 Table[{n, Transpose[Table[mdatsymfix1[initphm[[i, 1]], initphm[[i, 2]],
       initphm[[i, 3]], ss, αα, mm, r, tend][[n]] // Chop, {i, 1, 6}]]}, {n, nvals}]
```

```
initKPf = {{0.8695, 0.99, 0.0048}, {0.8279, 0.95, 0.0241}, {0.7766, 0.9, 0.0471},
   {0.6768, 0.8, 0.0471}, {0.5814, 0.7, 0.1123}, {0.4909, 0.6, 0.1261}};
```

```
mtabdatKPf[ss_, αα_, mm_, r_, tend_][nvals_] :=
 Table[{n, Transpose[Table[mdatsymfix1[initKPf[[i, 1]], initKPf[[i, 2]],
       initKPf[[i, 3]], ss, αα, mm, r, tend][[n]] // Chop, {i, 1, 6}]]}, {n, nvals}]
```

3.2.2 Example : Figure 5B (r = 0.1, phenotype matching initial conditions)

```
mtabdatphmfix1[0.2, 10, 0.01, 0.1, 1000][{1, 10, 100, 1000}] // TableForm //
 AbsoluteTiming
```

```
{7.87213,
        1     0       0       0      0      0      0
              0       0       0      0      0      0
              0       0       0      0      0      0
              0.99    0.95    0.9    0.8    0.7    0.6
              0.99    0.95    0.9    0.8    0.7    0.6
              0.0099  0.0475  0.09   0.16   0.21   0.24
        10    0.0224873 0.0221912 0.0216375 0.019817  0.0172123  0.014219
              0.0172485 0.0170147 0.0165398 0.0149046 0.0125822  0.00998798
              0.0140545 0.013815  0.0133707 0.011944  0.00998956 0.00784198
              0.975931  0.968589  0.954967  0.900276  0.799773   0.664923
              0.982095  0.978239  0.968191  0.915964  0.812708   0.673315
              0.0145323 0.0172381 0.0244119 0.0619359 0.127307   0.188458
        100   0.247323  0.237737  0.224909  0.197605  0.171399  0.139585
              0.18307   0.172009  0.157122  0.125222  0.0946422 0.0592968
              0.0894122 0.0844256 0.0775409 0.0622144 0.0469798 0.0293562
              0.744343  0.718285  0.681459  0.595573  0.503704  0.398806
              0.807472  0.777674  0.734793  0.632906  0.522758  0.396772
              0.0930016 0.102899  0.115244  0.135624  0.14348   0.134928
        1000  0.469672 0.452363 0.429076 0.377963 0.325665 0.263973
              0.445757 0.424917 0.396882 0.335359 0.27244  0.198303
              0.131297 0.129623 0.126567 0.116683 0.102201 0.0798341
              0.522044 0.504688 0.481238 0.429347 0.375613 0.311244
              0.544268 0.52337  0.495132 0.432642 0.367925 0.290385
              0.131917 0.132834 0.133248 0.130788 0.123352 0.108018
}
```

```
mtabdatphmfix1[0.2, 10, 0.01, 0.1, 100000][
  {1, 10, 100, 1000, 10000, 20000, 50000, 80000, 100000}] // TableForm
```

1	0	0	0	0	0	0
	0	0	0	0	0	0
	0	0	0	0	0	0
	0.99	0.95	0.9	0.8	0.7	0.6
	0.99	0.95	0.9	0.8	0.7	0.6
	0.0099	0.0475	0.09	0.16	0.21	0.24
10	0.0224873	0.0221912	0.0216375	0.019817	0.0172123	0.014219
	0.0172485	0.0170147	0.0165398	0.0149046	0.0125822	0.00998798
	0.0140545	0.013815	0.0133707	0.011944	0.00998956	0.00784198
	0.975931	0.968589	0.954967	0.900276	0.799773	0.664923
	0.982095	0.978239	0.968191	0.915964	0.812708	0.673315
	0.0145323	0.0172381	0.0244119	0.0619359	0.127307	0.188458
100	0.247323	0.237737	0.224909	0.197605	0.171399	0.139585
	0.18307	0.172009	0.157122	0.125222	0.0946422	0.0592968
	0.0894122	0.0844256	0.0775409	0.0622144	0.0469798	0.0293562
	0.744343	0.718285	0.681459	0.595573	0.503704	0.398806
	0.807472	0.777674	0.734793	0.632906	0.522758	0.396772
	0.0930016	0.102899	0.115244	0.135624	0.14348	0.134928
1000	0.469672	0.452363	0.429076	0.377963	0.325665	0.263973
	0.445757	0.424917	0.396882	0.335359	0.27244	0.198303
	0.131297	0.129623	0.126567	0.116683	0.102201	0.0798341
	0.522044	0.504688	0.481238	0.429347	0.375613	0.311244
	0.544268	0.52337	0.495132	0.432642	0.367925	0.290385
	0.131917	0.132834	0.133248	0.130788	0.123352	0.108018
10000	0.4725	0.467005	0.459595	0.443215	0.426191	0.405532
	0.449162	0.442547	0.433625	0.413904	0.393409	0.36854
	0.131521	0.131072	0.130384	0.128532	0.126125	0.122552
	0.524873	0.519374	0.511948	0.49549	0.478324	0.457408
	0.547675	0.541054	0.532112	0.512294	0.491624	0.466436
	0.131718	0.132091	0.132513	0.133109	0.133233	0.132696
20000	0.473447	0.471913	0.469843	0.465261	0.460488	0.454675
	0.450302	0.448455	0.445963	0.440447	0.4347	0.427701
	0.131594	0.131476	0.131311	0.130919	0.130472	0.129876
	0.52582	0.524286	0.522215	0.517627	0.512843	0.50701
	0.548815	0.546968	0.544474	0.53895	0.533189	0.526166
	0.131649	0.13176	0.131905	0.132199	0.132467	0.132741
50000	0.473805	0.473772	0.473727	0.473627	0.473523	0.473397
	0.450734	0.450694	0.45064	0.45052	0.450395	0.450242
	0.131621	0.131618	0.131615	0.131607	0.131599	0.13159
	0.526179	0.526146	0.526101	0.526001	0.525897	0.52577
	0.549247	0.549207	0.549152	0.549032	0.548907	0.548755
	0.131622	0.131624	0.131628	0.131635	0.131643	0.131652
80000	0.473813	0.473812	0.473811	0.473809	0.473807	0.473804
	0.450743	0.450743	0.450741	0.450739	0.450736	0.450733
	0.131621	0.131621	0.131621	0.131621	0.131621	0.131621
	0.526187	0.526186	0.526185	0.526183	0.526181	0.526178
	0.549256	0.549255	0.549254	0.549252	0.549249	0.549245
	0.131621	0.131621	0.131621	0.131622	0.131622	0.131622
100000	0.473813	0.473813	0.473813	0.473813	0.473813	0.473812
	0.450744	0.450744	0.450743	0.450743	0.450743	0.450743
	0.131621	0.131621	0.131621	0.131621	0.131621	0.131621
	0.526187	0.526187	0.526187	0.526187	0.526186	0.526186
	0.549256	0.549256	0.549256	0.549256	0.549256	0.549256
	0.131621	0.131621	0.131621	0.131621	0.131621	0.131621

```
params[αα_] := {s1 → 0.2, s2 → 0.2, m1 → 0.01, m2 → 0.01, α1 → αα, α2 → αα};
Table[t12t22 = FindRoot[{factdelta3maler1 /. p12p22genmt12 /. dd12genmt12 /. params[αα],
      factdelta6maler1 /. p12p22genmt12 /. dd12genmt12 /. params[αα]} == 0,
    {{t12, 0.45}, {t22, 0.56}}]; Flatten[
  Select[{{p12, t12, dd1, p22, t22, dd2}} /. p12p22genmt12 /. dd12genmt12 /. t12t22 /.
     params[αα], 0 < #[[1]] < 1 && 0 < #[[2]] < 1 &&
      -Min[(1 - #[[1]]) (1 - #[[2]]), #[[1]] #[[2]]] ≤ #[[3]] ≤ Min[#[[1]] (1 - #[[2]]),
        #[[2]] (1 - #[[1]])] && 0 < #[[4]] < 1 && 0 < #[[5]] < 1 &], 1], {αα, 10, 10}]
```

```
{{0.473813, 0.450744, 0.131621, 0.526187, 0.549256, 0.131621}}
```

```
mplotcor2br01endp =
  ListPointPlot3D[Table[ddat = mdatsymfix1[initphm[[i, 1]], initphm[[i, 2]],
       initphm[[i, 3]], 0.2, 10, 0.01, 0.1, 100000][[100000, 4 ;; 6]];
     {{ddat[[1]], ddat[[2]], ddat[[3]] / (√(ddat[[1]] (1 - ddat[[1]]) ddat[[2]] (1 -
              ddat[[2]])))}}, {i, 1, 6}], PlotStyle → {Directive[Red, PointSize[0.035]],
     Directive[Darker[Green], PointSize[0.035]], Directive[Magenta, PointSize[0.035]],
     Directive[Orange, PointSize[0.035]], Directive[Green, PointSize[0.035]],
     Directive[Blue, PointSize[0.035]]}, AxesLabel → {p2, t2, D}];
mplotcor2br01all = ListPointPlot3D[Table[Table[ddat = mdatsymfix1[initphm[[i, 1]],
        initphm[[i, 2]], initphm[[i, 3]], 0.2, 10, 0.01, 0.1, 100000][[n, 4 ;; 6]];
      {ddat[[1]], ddat[[2]], ddat[[3]] /
        (√(ddat[[1]] (1 - ddat[[1]]) ddat[[2]] (1 - ddat[[2]])))},
      {n, Flatten[{Join[Table[j, {j, 1, 1000}], Table[1000 + 10 j, {j, 1, 4900}]]}]}],
    {i, 1, 6}], PlotStyle → {Red, Darker[Green], Magenta, Orange, Green, Blue}];
mplotcor2br01 = Show[mplotcor2br01all, mplotcor2br01endp,
  ImageSize → 450, PlotRange → {{0, 1}, {0, 1}, {-0.005, 1.005}},
  AxesLabel → {Text[Style[p22, Black, 15]],
    Text[Style[t22, Black, 15]], Text[Style[D2,cor, Black, 15]]},
  LabelStyle → Directive[Black, FontFamily → "Helvetica", 12], BoxRatios → {1, 1, 0.6},
  ViewPoint → {3.4, -2.4, 1.6}, AxesEdge → {{0, 0}, {1, 0}, {1, 1}},
  Ticks → {{0, 0.5, 1}, {{0, "     0"}, 0.5, 1}, {0, 0.5, 1}}]
```

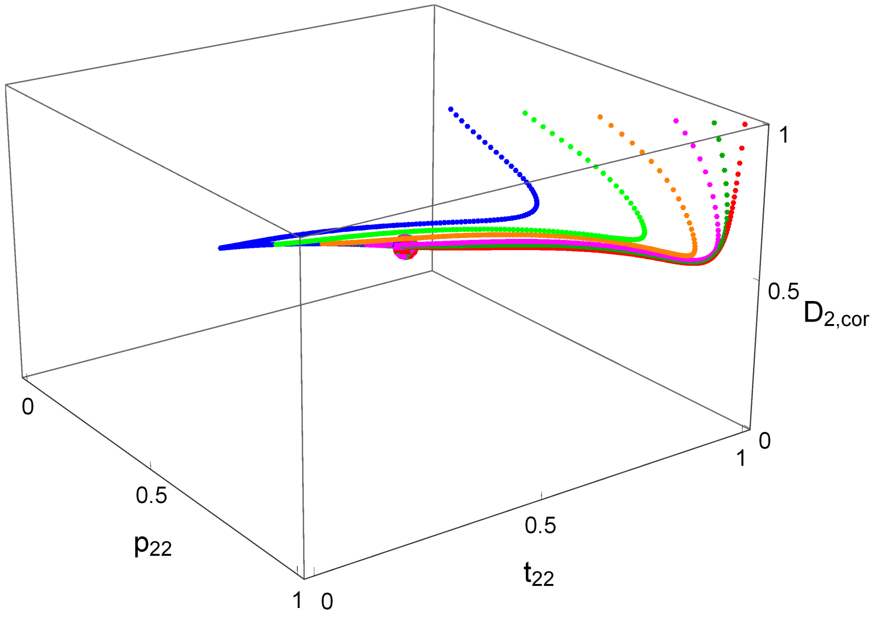

Supplement: Supplementary file 1 [file genes-09-00217-s001.zip › Servedio and Burger Supplementary Information proofs/Supplementary Materials File S4.pdf]
