# Supplementary material for: The Effects on Parapatric Divergence of Linkage between Preference and Trait Loci versus Pleiotropy
Source: Genes (Basel). 2018 Apr 17;9(4):217. doi: 10.3390/genes9040217 (PMC5924559; doi:10.3390/genes9040217)
Supplement: Supplementary file 1 [file genes-09-00217-s001.zip › Servedio and Burger Supplementary Information proofs/FigureS2.pdf]

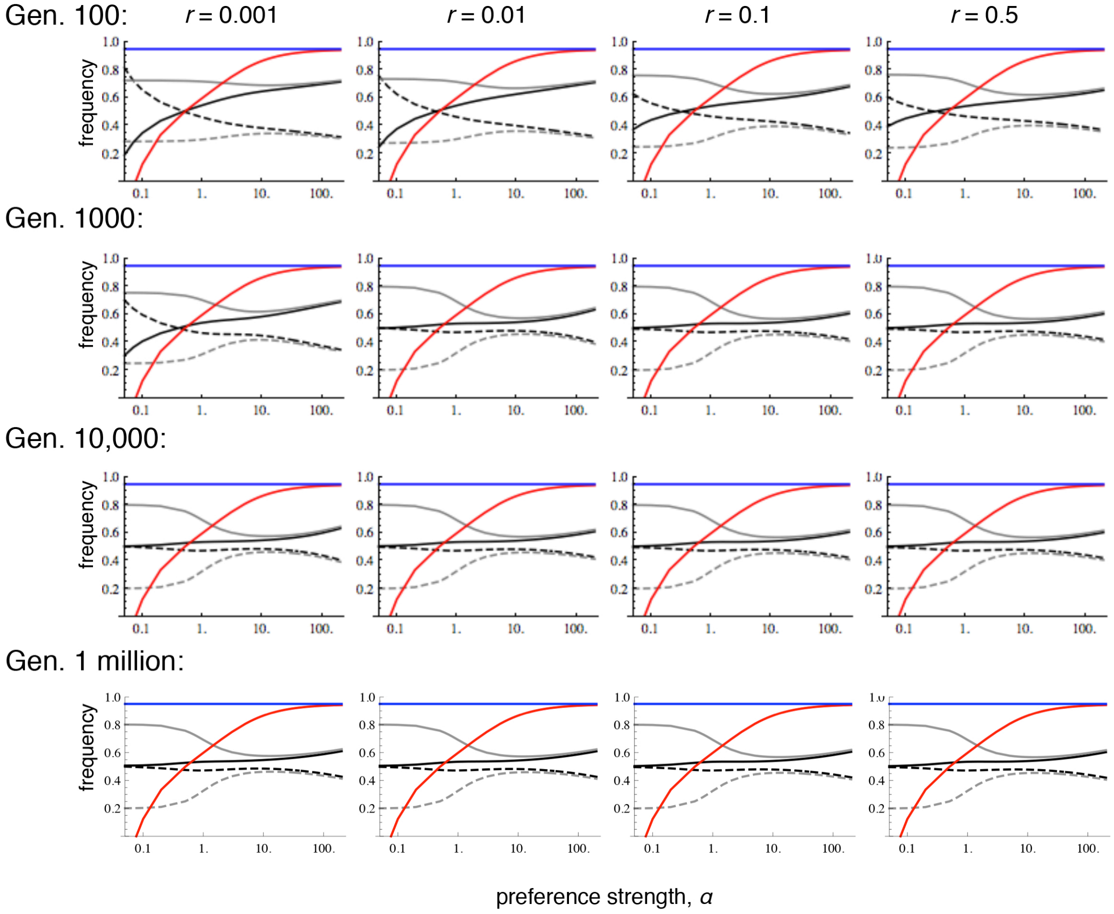

Figure S2. Preference and trait divergence at 100, 1000, 10,000, and 1 million generations after secondary contact when the trait is expressed in both sexes. The parameter values and legend follow Figure 1. Blue represents the starting conditions for t_2 (=0.95) in population 2 (compare to the grey solid lines), and Red lines represent the equilibrium starting conditions for p_2 in population 2 in allopatry given the blue value for t_2 (compare with the black solid lines for the endpoints for P_2). The starting conditions of population 1 (not shown) are t_2 = 0.07, yielding a p_2 roughly one minus the values in the red line.
