# Supplementary material for: The Effects on Parapatric Divergence of Linkage between Preference and Trait Loci versus Pleiotropy
Source: Genes (Basel). 2018 Apr 17;9(4):217. doi: 10.3390/genes9040217 (PMC5924559; doi:10.3390/genes9040217)
Supplement: Supplementary file 1 [file genes-09-00217-s001.zip › Servedio and Burger Supplementary Information proofs/FigureS3.pdf]

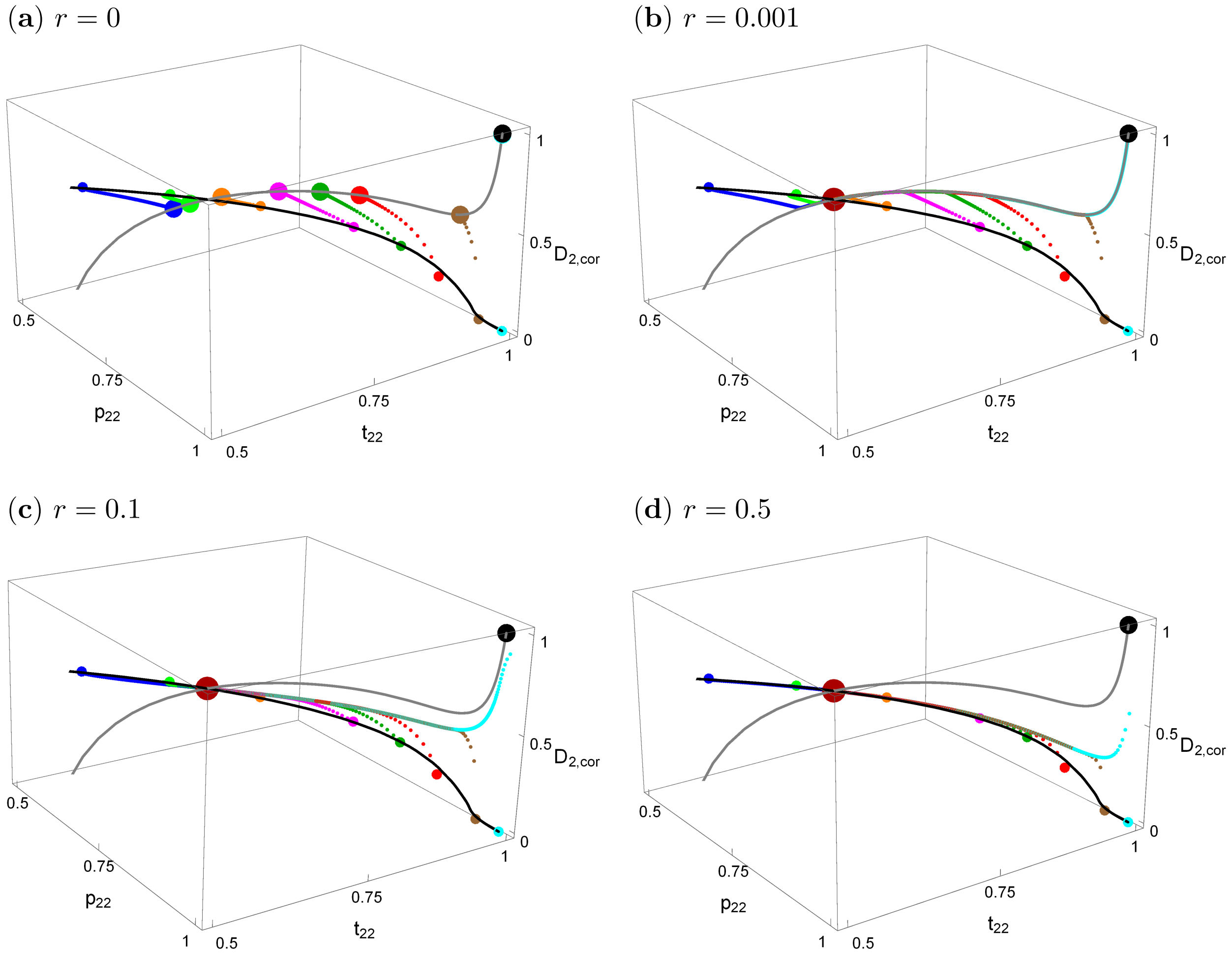

Figure S3. The effects of initial conditions and recombination rates on evolutionary trajectories with symmetric initial conditions, i.e., $p_{2,1} = 1 - p_{2,2}$, $t_{2,1} = 1 - t_{2,2}$, $D_1 = D_2$. The trait is expressed in both sexes and migration is symmetric ($m_1 = m_2 = 0.01$). The initial conditions are from the curve of stable equilibria in an isolated population and given below. The black curve shows all stable equilibria if initial conditions are arbitrary. The gray curve shows the stable equilibria for $r = 0$ and symmetric initial conditions. The big black symbol indicates the stable polymorphic equilibrium under phenotype matching. Small dots in different colors show evolutionary trajectories in population 2 starting from different initial values (given below). The initial values are indicated by slightly larger symbols. The big colored symbols in panel (**a**) show the equilibrium that is reached. The big dark-red symbol in panels (**b**), (**c**), and (**d**) shows the unique stable symmetric equilibrium for $r > 0$, to which all trajectories converge. It is at the intersection of the black and gray curves. Panels differ in their recombination rates. (**a**): $r = 0$; (**b**): $r = 0.001$, (**c**): $r = 0.1$, (**d**): $r = 0.5$. The other parameters are $s_1 = s_2 = 0.2$, $\alpha_1 = \alpha_2 = 10$, $m_1 = m_2 = 0.01$. The initial values are $(p_{2,2}, t_{2,2}, D_2) = (0.9999, 0.9999, 0)$ (cyan), $(0.95, 0.9999, 0)$ (brown), $(0.8695, 0.99, 0.0048)$ (red), $(0.8279, 0.95, 0.0241)$ (dark green), $(0.7766, 0.9, 0.0471)$ (magenta), $(0.6768, 0.8, 0.0471)$ (orange), $(0.5814, 0.7, 0.1123)$ (light green), $(0.4909, 0.6, 0.1261)$ (blue).
