# Supplementary material for: The Effects on Parapatric Divergence of Linkage between Preference and Trait Loci versus Pleiotropy
Source: Genes (Basel). 2018 Apr 17;9(4):217. doi: 10.3390/genes9040217 (PMC5924559; doi:10.3390/genes9040217)
Supplement: Supplementary file 1 [file genes-09-00217-s001.zip › Servedio and Burger Supplementary Information proofs/FigureS1.pdf]

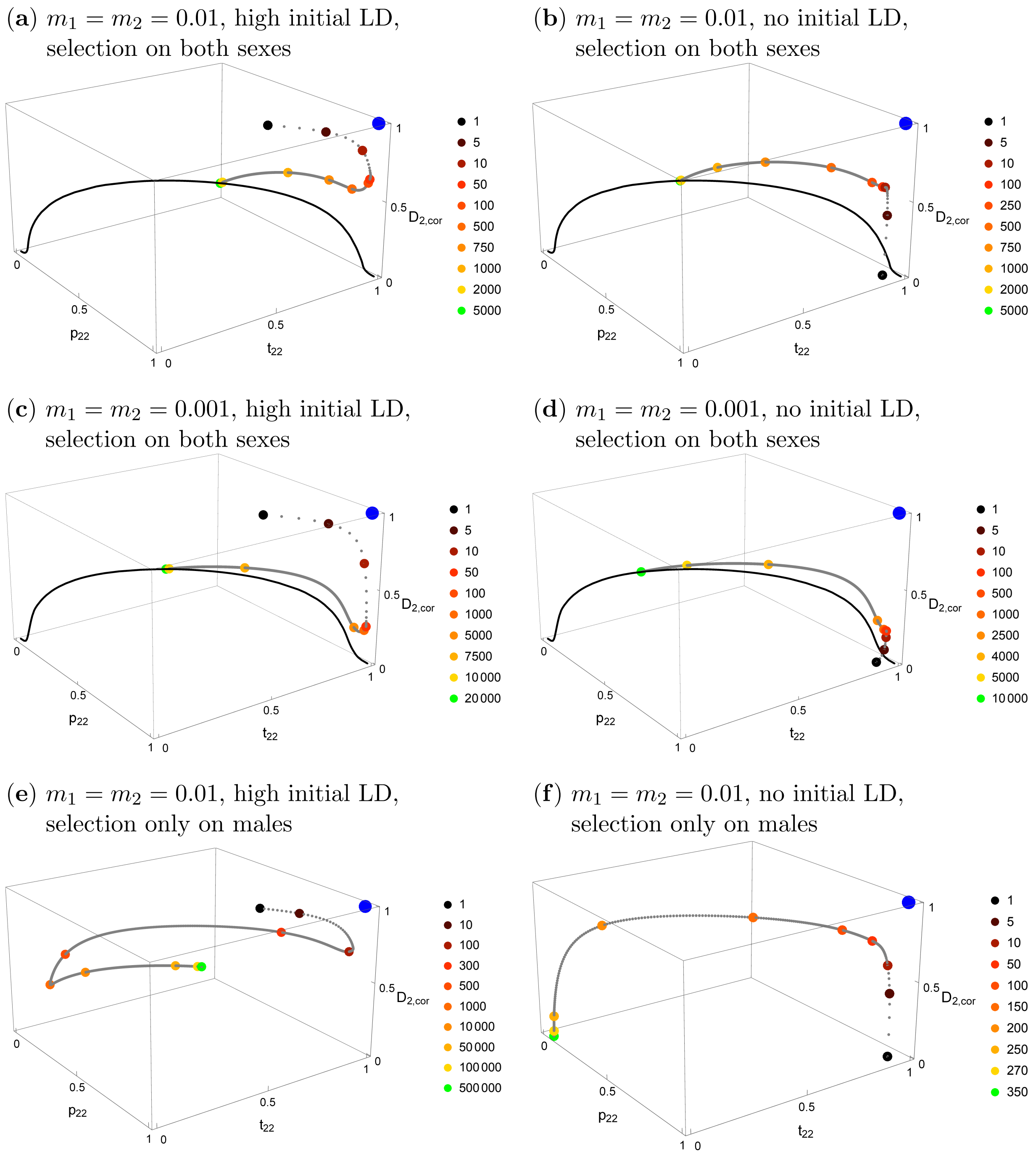


**Figure S1.** Detailed temporal evolution for selected initial conditions. Colored symbols show $(p_{2,2}, t_{2,2}, D_{2,\text{cor}})$ at the generation given in the legend. Gray dots show the complete trajectories. Linkage between trait and preference locus is tight ($r = 0.01$). The big blue symbol shows the stable polymorphic equilibrium under phenotype matching. Black curves show all stable equilibria for $r > 0$ when both sexes express the trait. Top row ($\mathbf{a}, \mathbf{b}$): both sexes express the trait and migration is symmetric ($m_1 = m_2 = 0.01$). Middle row ($\mathbf{c}, \mathbf{d}$): both sexes express the trait and migration is symmetric and very weak ($m_1 = m_2 = 0.001$). Bottom row ($\mathbf{e}, \mathbf{f}$): only males express the trait and migration is symmetric ($m_1 = m_2 = 0.01$). In all panels, population 1 is initially fixed for $P_1$ and $T_1$. Left and right columns differ by the initial conditions in population 2. Left column: $p_{2,2}(0) = t_{2,2}(0) = 0.7$, $D_2(0) = 0.2$ ($D_{2,\text{cor}}(0) = 0.952$); right column: $p_{2,2}(0) = t_{2,2}(0) = 0.95$, $D_2(0) = 0$. The other parameters are $s_1 = s_2 = 0.2$ and $\alpha_1 = \alpha_2 = 10$.
