# Supplementary material for: The Effects on Parapatric Divergence of Linkage between Preference and Trait Loci versus Pleiotropy
Source: Genes (Basel). 2018 Apr 17;9(4):217. doi: 10.3390/genes9040217 (PMC5924559; doi:10.3390/genes9040217)
Supplement: Supplementary file 1 [file genes-09-00217-s001.zip › Servedio and Burger Supplementary Information proofs/FigureS4.pdf]

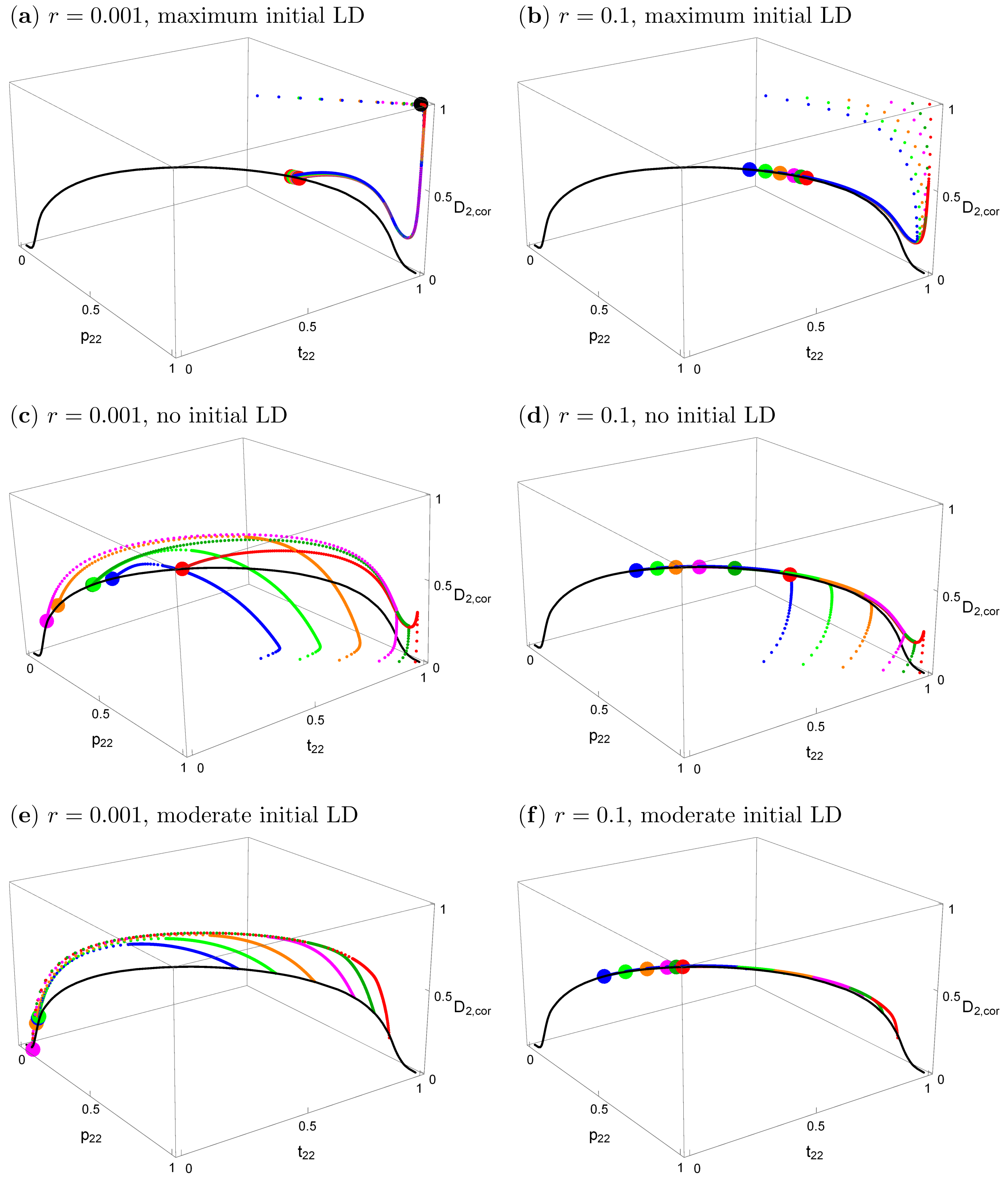

Figure S4. The effects of initial conditions and recombination rates on evolutionary trajectories when the trait is expressed in both sexes and migration is symmetric and very small, $m = 0.001$. Otherwise, this figure is analogous to Figure 3 (in which $m = 0.01$). The graphs in this figure look similar to those in Figure 3. However, often less divergence is maintained at equilibrium compared to $m = 0.01$. Most notably, the time course is about 3–10 times long than with $m = 0.01$. Especially, the trajectories that start with high divergence (red, dark green) hang around very high values of $p_{2,2} > 0.9$ and $t_{2,2} > 0.9$ for very long; typically 1000 generations for the higher recombination rate; and 20,000–50,000 for $r = 0.001$. During this time, only linkage disequilibrium changes (very slowly), and allele frequencies remain almost constant.
