# Supplementary material for: The Effects on Parapatric Divergence of Linkage between Preference and Trait Loci versus Pleiotropy
Source: Genes (Basel). 2018 Apr 17;9(4):217. doi: 10.3390/genes9040217 (PMC5924559; doi:10.3390/genes9040217)
Supplement: Supplementary file 1 [file genes-09-00217-s001.zip › Servedio and Burger Supplementary Information proofs/FigureS6.pdf]

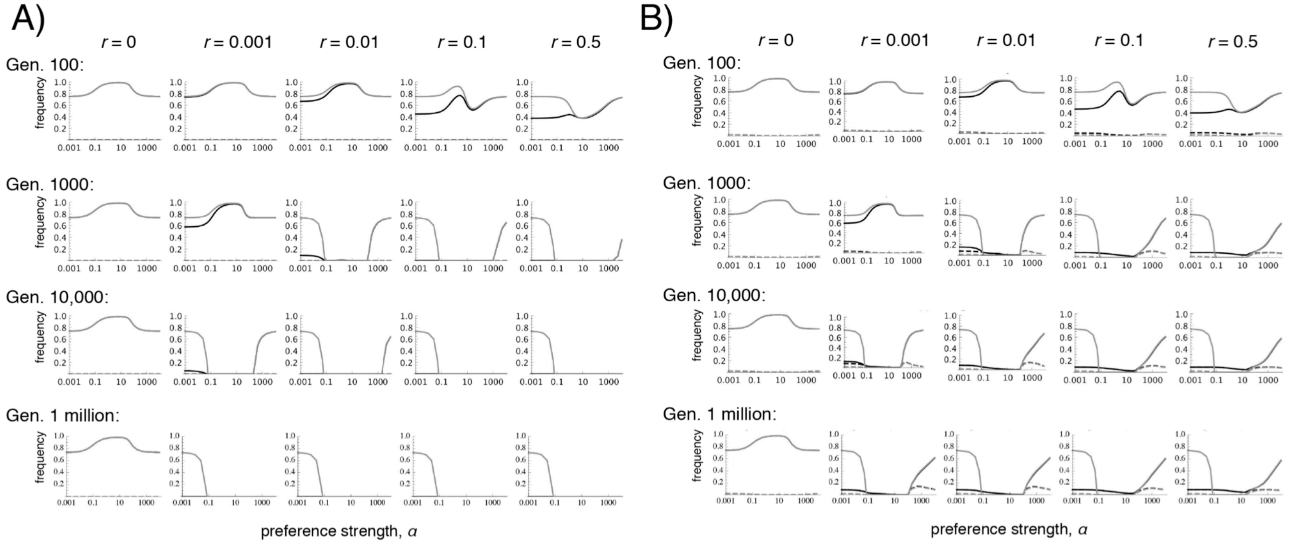

Figure S6. Preference and trait divergence at 100, 1000, 10,000, and 1 million generations after secondary contact when the trait is expressed in both sexes. The parameter values and legend follow Figure 1. a) Continent-island migration: $m_1 = 0$, $m_2 = 0.01$, b) asymmetric migration: $m_1 = 0.001$, $m_2 = 0.01$.
