# Supplementary material for: The Effects on Parapatric Divergence of Linkage between Preference and Trait Loci versus Pleiotropy
Source: Genes (Basel). 2018 Apr 17;9(4):217. doi: 10.3390/genes9040217 (PMC5924559; doi:10.3390/genes9040217)
Supplement: Supplementary file 1 [file genes-09-00217-s001.zip › Servedio and Burger Supplementary Information proofs/FigureS8.pdf]

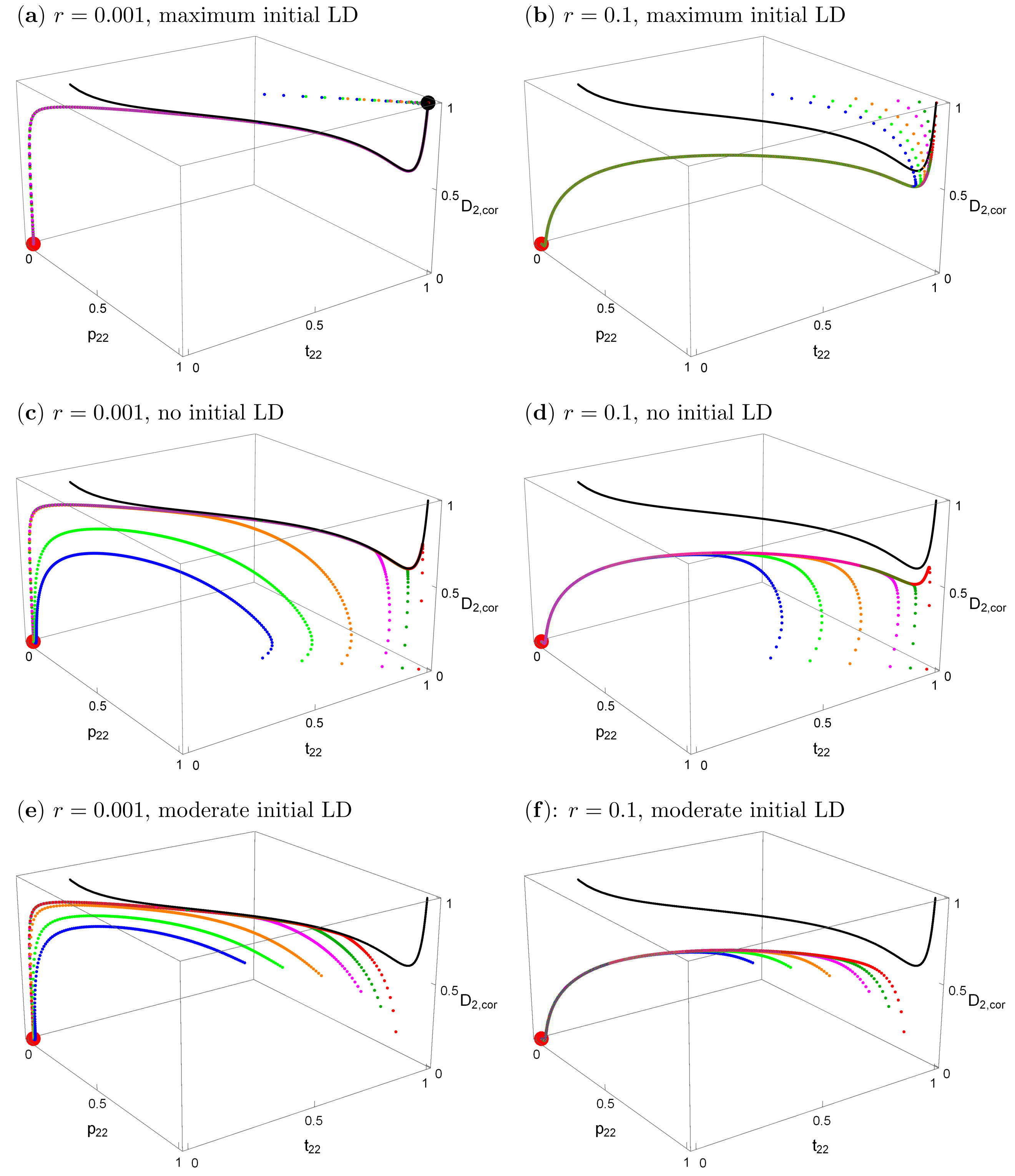

Figure S8. The effects of initial conditions and recombination rates on evolutionary trajectories when the trait is expressed in both sexes and there is continent-to-island migration. This figure is analogous to Figure 3 in the main text with the difference that here there is one-way migration into population 2, i.e., $m_1 = 0$ and $m_2 = 0.01$. In addition, here the black curve shows the equilibria for $r = 0$.
